# Pure and doped carbon quantum dots as fluorescent probes for the detection of phenol compounds and antibiotics in aquariums

**DOI:** 10.1038/s41598-023-39490-y

**Published:** 2023-08-08

**Authors:** Mahmoud A. Mousa, Hanaa H. Abdelrahman, Mamdouh A. Fahmy, Dina G. Ebrahim, Amira H. E. Moustafa

**Affiliations:** 1 Faculty of Science, Benha University, Banha, Egypt; 2 Faculty of Science, Alexandria University, Alexandria, Egypt; 3 Marine Chemistry Department, Environmental Division, National Institute of Oceanography and Fisheries (NIOF), Alexandria, Egypt

**Keywords:** Environmental sciences, Nanoscience and technology

## Abstract

The resulting antibiotic residue and organic chemicals from continuous climatic change, urbanization and increasing food demand have a detrimental impact on environmental and human health protection. So, we created a unique B, N-CQDs (Boron, Nitrogen doping carbon quantum dots) based fluorescent nanosensor to investigate novel sensing methodologies for the precise and concentrated identification of antibiotics and phenol derivatives substances to ensure that they are included in the permitted percentages. The as-prepared highly fluorescent B, N-CQDs had a limited range of sizes between 1 and 6 nm and average sizes of 2.5 nm in our study. The novel B, N-CQDs showed high sensitivity and selectivity for phenolic derivatives such as hydroquinone, resorcinol, and para aminophenol, as well as organic solvents such as hexane, with low detection limits of 0.05, 0.024, 0.032 and 0.013 µM respectively in an aqueous medium. The high fluorescence B, N-CQDs probes were examined using transmission electron microscopy (TEM), X-ray diffraction (XRD), Fourier-transform infrared spectroscopy (FTIR), and UV/VIS spectroscopy. The outcomes were compared to carbon quantum dots (CQDs) previously generated from Urea.

## Introduction

Climate change and global population expansion have increased the demand for food and medicine. Concerns about fisheries have led to the misuse of antibiotics as they have been overused. Antibiotics were used in around 63,000 tons in 2010, which is predicted to rise to 107,000 tons by 2030^1^. Antibiotics used in such large quantities in aquatic habitats may raise the risk of microorganisms that are resistant to the antibiotic. Over 700,000 people every year pass away from diseases brought on by bacteria resistant to antibiotics, making this a serious issue in our society. By 2050, if unchecked, this number might reach 10 million^2^.

Due to the discharge of contaminated wastewater from industrial, agricultural, and domestic activities, phenolic compounds exist in water bodies. These substances are well-recognized to be hazardous and negatively impact people and animals. Hydroquinone, one of the primary phenol derivatives, is a well-known hepatotoxic and carcinogenic chemical^3^. Recent research revealed hydroquinone could increase cancer risk by damaging DNA and impairing the immune system^4^. Amino phenols are potentially hazardous and mutagenic chemicals utilized or released in the industrial, medicinal, and agriculture sectors^5,6^. Para-aminophenols (PAP) are also used as a raw material for producing Paracetamol, one of the most commonly made drugs globally. They have been available for oral administration for a long time^7^. The amount of PAP in the world is increasing at a rate of 5% yearly. PAP induces nephrotoxicity and, similarly to Paracetamol, induces hepatotoxicity^8^. Furthermore, (PAP) adversely affects aquatic life^9,10^. So it is critical to seek highly efficient, environmentally friendly, and stable non-toxic technologies.

Due to their exceptional characteristics, such as superior non-toxicity and biocompatibility, mechanical strength, and thermal stability, carbon-based nanomaterials have recently attracted much research interest. These characteristics open up numerous possibilities in various fields of study^11,12^ Carbon quantum dots (CQDs) are zero-dimensional (0D) materials with an average size of less than 10 nm^13^. CQDs have a wide range of applications in the fields of health and environment^14^.

Due to the potential for new research about the mechanisms and enhanced future methods of detecting various ambient chemicals, CQD-based sensors have been the subject of substantial research. It was suggested to be a promising strategy for detecting pollutants and environmental monitoring^15,16^ Because of their high photo stability, biocompatibility, relative fluorescence strength, and controllable photoluminescence potential for surface functionalization^17^. Doping of CQDs with N-, P-, S-, and B- and other heteroatoms has been shown to alter their band gap and electron density, enhancing their quantum yield and extending their range of applications^18^. Doped CQDs successfully detect undesired pharmaceutics and environmental contaminants such as pesticides, antibiotics, phenolic chemicals, poly-aromatic hydrocarbons, and heavy metal ions^19^.

Most of the reported doped-CQDs are produced using two or more expensive reaction precursors, which may complicate purification^20^. Therefore, finding a simple, readily available, low-cost precursor to make two different kinds of heteroatom-doped CQDs is still a challenge. To further enhance the varieties and uses of CQDs, we intend to synthesize a novel unique CQDs doped with nitrogen and boron by a simple facile method for variable sensing applications.

Our research is concentrated on carbon quantum dots (CQDs) and how they may be used to detect various chemicals. This detection is significant for biological, pharmacological, and environmental goals. Advanced innovative dual-function optical sensors based on B, N-CQDs, are being investigated. We demonstrate how creative nanomaterial-based analytical biosensors can successfully detect, quantify, and confirm antibiotic residue’s presence to ensure it is within the permitted percentage.

Furthermore, demonstrate their ability to detect phenolic derivatives in aqueous solutions as chemical sensors. Their optical and chemical properties are reviewed in detail here, focusing on the advances toward creating luminescent nanoparticles with several sensing uses to advise developing more efficient, susceptible, and accurate sensors. It’s important to mention that this innovative technology doesn’t just recognize one type of antibiotic; it identifies multiple types and also determines the varied concentrations of each. This is what distinguishes our work and is not achieved by others. Finally, new viewpoints are presented to stress the numerous opportunities and obstacles that remain unsolved but are crucial in unlocking the full potential of these strong nanomaterials.

We describe the fluorescent B, N co-doping CQDs’ unprecedented sensing strategy, eliminating the need for time-consuming and costly traditional techniques. So, it helps to develop advanced eco-friendly sensors for various future applications. And provide a technique for creating dual-mode sensing systems based on brand-new stimuli-responsive materials for different analytical uses.

## Experimental section

### Chemicals

Citric acid monohydrate, Urea, trisodium citrate, and boric acid all were purchased from Sigma-Aldrich and Merck Chemical Company. Nitric acid (HNO_3_, 72%) and sodium hydroxide (NaOH, 99%) pellet extra pure from Oxford Lab Fine Chem. India, erythromycin, floroquin, and penicillin were obtained from (Medical Professions for veterinary products Co. *MUVCO*). Oxytetracycline hydrochloride was purchased from (Pharma SWEDE). The tested antibiotics were prepared by calculating the equivalent active materials and weighing the powder. Hydroquinone powder (+ 99%) was purchased from (Chemsavers, Egypt.), and p-aminophenol powder (99%) and resorcinol (99%) were purchased from (Sigma-Aldrich). All chemicals were marked as reagent grade and used as received without further purification. For all experiments, double-deionized water was used.

### Synthesis of Photo luminescent CQDs

A thermal decomposition procedure^21^ made CDs with minimal changes. To make translucent solutions, 40 mL of deionized water was mixed with 6 g citric acid and 12 g urea. In an electric oven, the transparent solution was heated to 200 °C and held at that temperature for 4 h. The combination was then allowed to cool naturally to ambient temperature, transforming from a colorless liquid to a black solid. The products were then dissolved in deionized water, and the CDs were filtered using a 0.45 μm membrane filter. Finally, a solution for CDs was found. More investigations and applications were performed on the created CDs.

### Synthesis of highly luminescence B, N –CQDs

The B and N co-doped CDs were created by combining previously reported procedures with minimal changes^22^. 3 g of tri-sodium citrate, 3 g of Urea, and 3 g of boric acid were mixed with 150 mL of deionized water and treated hydrothermally at 180 °C for four hours in a stainless steel autoclave with Teflon coating. The CDs were then filtered using a filter paper 0.45 µm membrane filter after the autoclave was cooled to room temperature under ambient conditions. Because of the final solution’s high intensity and quantum yield, 1 mL (13 mg mL^−1^) of the CD stock solution was diluted to 50 mL for further characterization and applications.

### Fluorescence measurements

First, stock solutions of various concentrations of the antibiotics: oxytetracycline hydrochloride, penicillin, erythromycin, and floroquine were made. The active ingredient concentrations of all antibiotics were kept constant to ensure that the results were accurate. An identical stock solution of toluene, acetonitrile, hexane, butane, THF, hydroquinone, resorcinol, and para aminophenol with concentrations ranging from 500 nM to 5 µM was made up.

A small amount of antibiotic solution (0.1 mL) was added to 0.1 mL of B, N-CQDs diluted solution and gently stirred for 5 min at room temperature. Then, using a Perkin Elmer model LS55 fluorescence spectrophotometer with a 330 nm excitation wavelength, the solution was ready for PL (Photoluminescence Spectroscopy) measurements. For the determination of phenol derivatives, the same procedure was used. All the measurements were performed under the same conditions and at room temperature.

### Calculation of the fluorescence quantum yield

The fluorescence quantum yield of B, N-CQDs has been reported in previous studies^23^. It was calculated from the following equation using Fluorescent as a standard whose quantum yield is about 0.95.

1 $${\text{Y}}_{{\text{X}}} = {\text{ Ys}}\left( {{\varvec{F}}x {\mathbf{A}}s {\varvec{\eta}}x / {\varvec{F}}s {\varvec{A}}x\user2{ \eta }s} \right)$$

where Y denotes the quantum yield, F_X_ and F_s_ denote the integrated fluorescence intensity, *A*_*X*_ and *As* indicate the absorbance, and η_x_ and η_s_ denote the refractive indices of the solvent of the CDs and the fluorescein solution, respectively. At 340 nm, the absorbance was measured, and the fluorescence spectrum was obtained at this wavelength.

### Characterization of carbon quantum dots (CQDs)

#### Instruments

To explore the crystalline structures of the produced materials, X-ray diffraction (XRD) patterns were recorded on an X-ray diffraction spectrophotometer utilizing Cu K radiation (*λ* = 1.5406, operating voltage 45 kV, and current 40 mA) at a step size of 0.02° and *2θ* range of 20–90°.

A transmission electron microscope (TEM) was used to examine nanoparticle morphology (JEM-1400 Plus, Japan). The sample was obtained by dissolving nanoparticles in distilled water, sonicating for 30 min to ensure complete dispersion, and then applying a single drop of nanoparticle solution on a carbon-coated copper grid (400 meshes) and drying at room temperature.

The FTIR spectra were captured with the help of (Nicolet 6700, Nicolet, United States. All spectra were obtained using pellets made by combining KBr (Sigma Aldrich) with pure powder of the produced nanoparticles in 4000–400 cm^−^ range. UV–Vis absorption spectrum was investigated utilizing the thermal scientific model EVO300.

## Results and discussion

### Morphological and structural characterization

**Figure 1:**
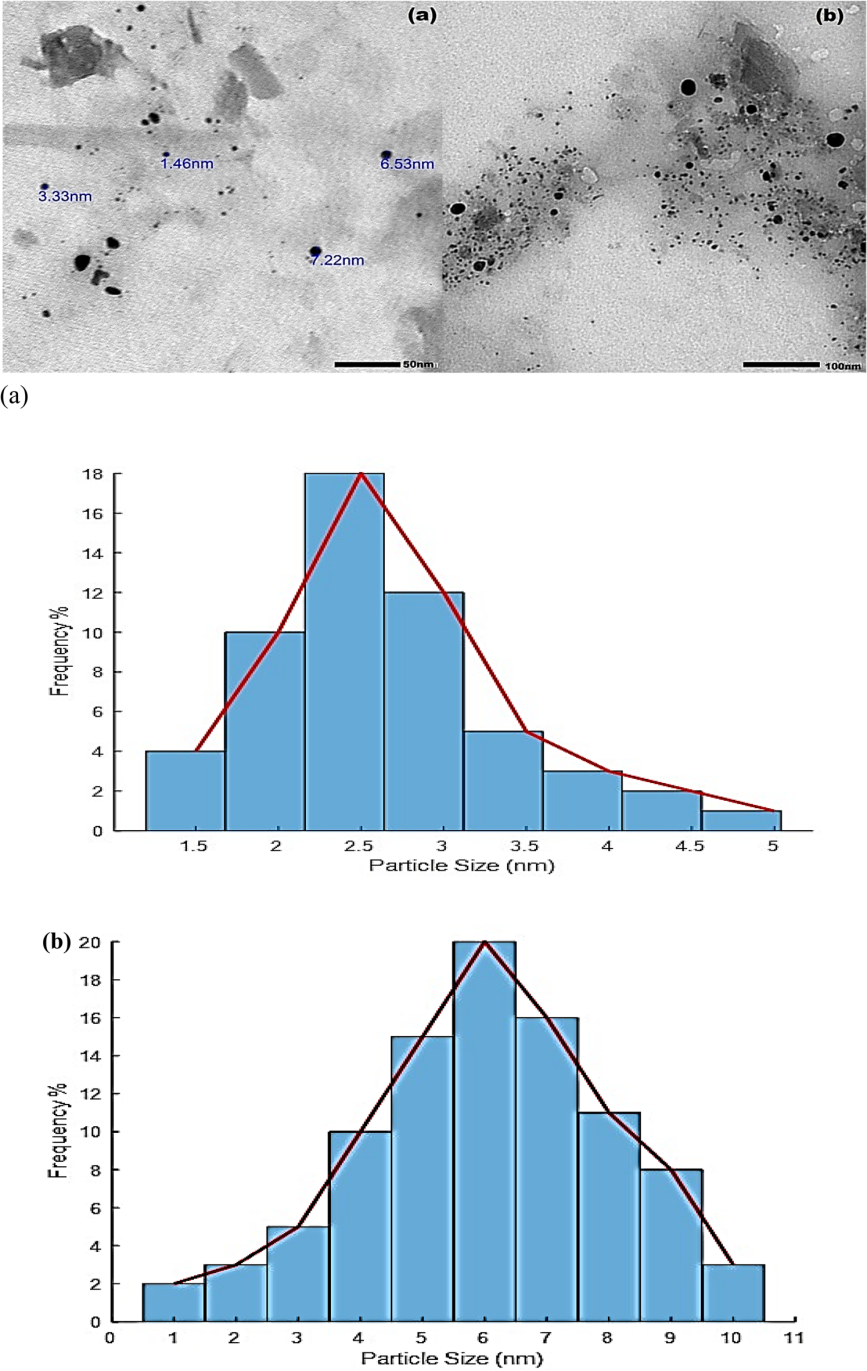
TEM images and histogram of (**a**) B, N doped CQDs (**b**) Carbon quantum dots (CQDs).

TEM was used to describe the size and morphology of the synthesized CQDs. TEM images and histogram in Fig. 1a,b Show that B, N-doped CQD, and CQDs exhibit uniform distribution with good dispersity of quasi-spheres morphological structures with average sizes of 2.5 and 6 nm, respectively.

### X-Ray diffraction (XRD)

**Figure 2:**
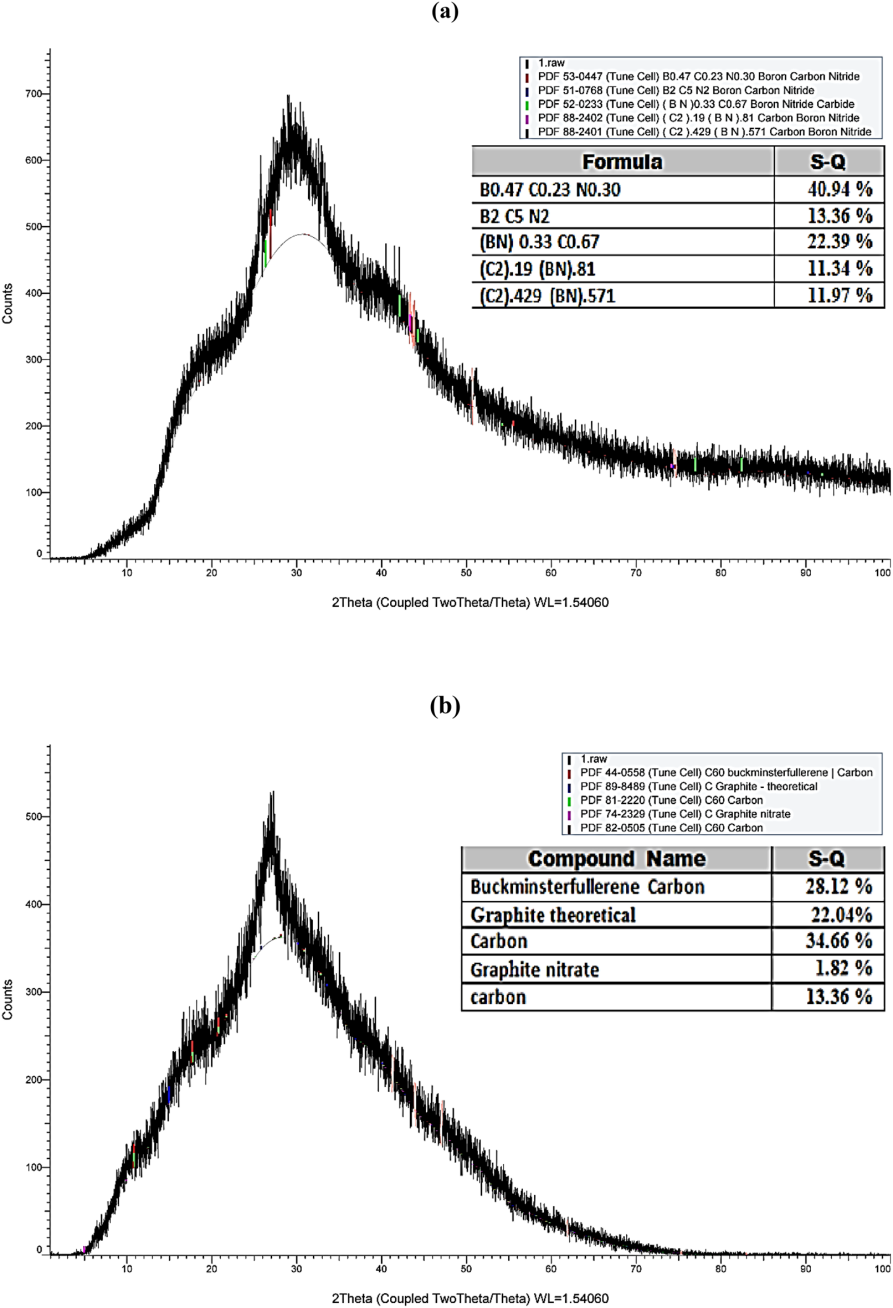
XRD pattern of (**a**) B, N doped CQDs (**b**) Carbon quantum dots (CQDs).

XRD pattern of the prepared CQDs and B, N co-doping CQDs are illustrated in Fig. 2a,b. Broad humped diffraction peaks centered at 27° and 30° for the doped and pure CQDs, respectively, reveal the amorphous structure of both materials, referring to the production of incredibly disorderly carbon atoms. The observed diffraction peaks correspond to the 3.2 and 2.8 Å interlayer spacing for CQD and B, N co-doping CQDs, respectively^24^ (Supplementary Information 1 & 2).

### FTIR analysis

**Figure 3:**
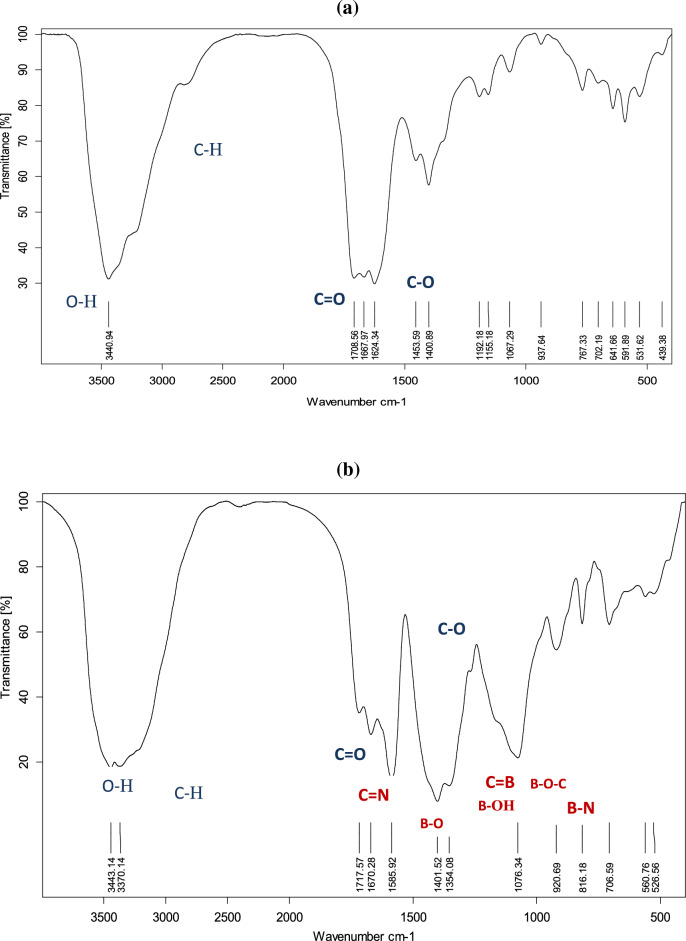
FTIR spectra of (**a**) Carbon quantum dots (CQDs) (**b**) B, N doped CQDs.

FTIR measurements were performed to investigate the functional group on the surface of the CQDs as prepared. FTIR analysis for the synthesized CQDs and B, N-doped CQDs were displayed in Fig. 3a,b. The spectra of the two samples show strong broadband at 3440 cm^−1^, comparable to –OH stretching vibrational and N–H bonds. C-H was responsible for the absorption peaks at 2771 cm^−1^. Strong bands detected at the wavenumber 1600–1750 cm^−1^ show the presence of C=O bonds on the surface of the particles, indicating the presence of the citrate molecule on the surface of the particles, which is what gives CDs their hydrophilic and water-soluble properties as well as increasing their stability. Bands at 1400 cm^−1^ and 1453 cm^−1^ were comparable to CN and –CO, respectively. The vibration peak of C=N was discovered at 1643 cm^−1^. On the other hand, the FTIR spectra of the synthesized B, N-doped CQDs showed differences from that of CQDs, such as B–O stretching vibration at 1405 cm^−1^ and absorption peak for B–O–C detected at 920 cm^−1^. B–OH bending and C=B stretching vibrations showed peaks at 1189 cm^−1^ and 1076 cm^−1^, respectively. However, the two peaks appeared at 1354 cm^−1^ and 816 cm^−1^. (in-plane B–N stretching vibration and out-of-plane B–N bending vibration, respectively) are connected to *sp*^2^-bonded B–N. All FTIR and XRD data successfully demonstrated the formation of B, N-doped CQDs, and the functional groups B–O, B–O–C, B–OH, C=B, C–N, N–H, and C=N confirm the doping, providing the high-performance and exceptional sensing properties.

### The Optical Properties of Carbon Quantum Dots

**Figure 4:**
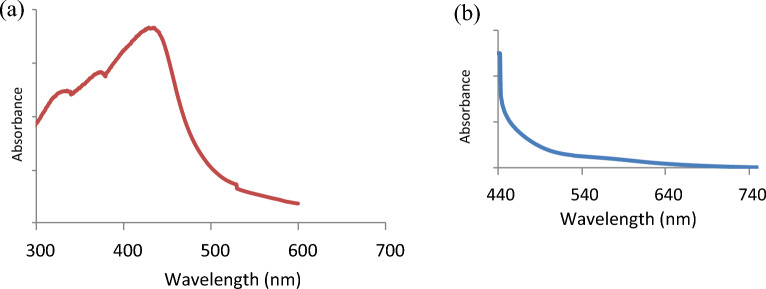
UV–VIS absorption spectra of (**a**) B, N co-doping CQDs (**b**) Carbon quantum dots CQDs.

UV–Vis absorption and photoluminescence spectra were used to evaluate the optical characteristics of the synthesized B, N CQDs, and CQDs from Urea. Deionized water diluted the samples to 0.2–0.05 mg/ml. The results obtained are illustrated in Fig. 4a,b. The UV–visible spectrum of the prepared CQDs in water shows three absorption bands^25^ at 320 nm, 360 nm, and 430 nm, which correspond to the π → π* transition of C=C or C=O bonds in which the orbital was *sp*^2^ hybridized and distinct bands at 350 nm and 340 nm was assigned to n → π* transition of edge functional groups electrons of B, O and N atoms^26^.

**Figure 5:**
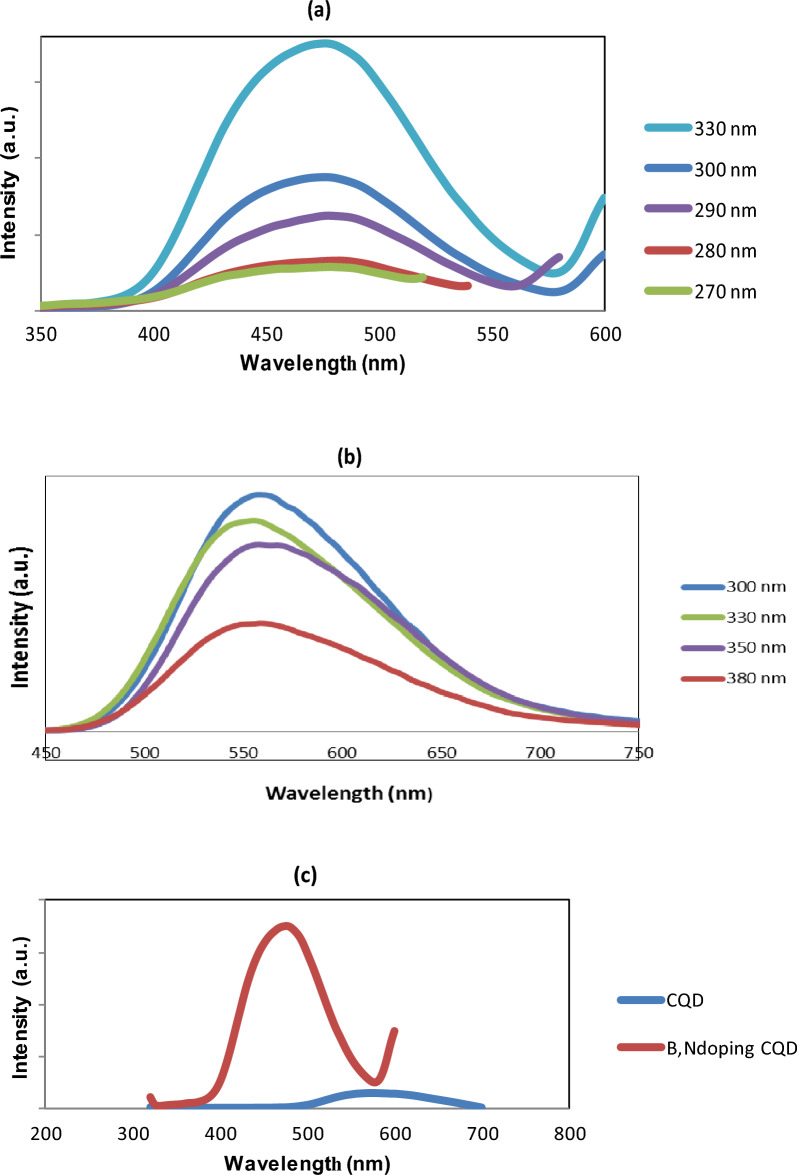
(**a**) Photoluminescence spectra of B, N-CQDs at different excitation wavelengths 270,280,290,300 and 330nm. (**b**) Photoluminescence spectra of CQDs at different excitation wavelengths 300,330,350, and 380 nm. (**c**) Comparison of fluorescence spectra of B, N-CQDs, and CQDs with an excitation wavelength of 330 nm.

These atoms have a stronger electronegativity than carbon; therefore, they inductively pull electrons out of π orbitals. The high carbon atoms conjugation facilitated the process. The extended absorption tail, which extends to 550 nm, is caused by transitions of low energy within the surface states caused by surface functional groups^27^. To further investigate the optical properties, the photoluminescence (PL) variation of B, N-CQDs, and CQDs from Urea with different stimulated wavelengths was explored to learn more about their PL features. The results are illustrated in Fig. 5. Exciting peaks are observed at 470 nm for all excited wavelengths for the as-prepared B, N-CQD. The intensity of the peaks decreases with decreasing the exciting wavelength from 330 to 270 nm, Fig. 5a.

On the other hand, CQDs prepared from Urea are illustrated in Fig. 5b. The intensity of the peaks decreases with the increase of the exciting wavelength from 300 to 380 nm; that means the intensity peaks get stronger as the exciting wavelength decreases. The band gap transition, which can regulate the fluorescence fluctuation of the manufactured CQDs, may cause the band gap to increase.

As previously observed, this intrinsic property is due to the variable particle sizes and wide distribution of emissive trap sites on as-prepared CQDs. Although the mechanism of CQDs photoluminescence is unknown, it is known to be significantly associated with the states, distribution, and association of functional groups on the surface^28^. Figure 5c compares the fluorescence spectra of CQDs and the doping CQDs at an excitation wavelength of 330 nm, which illustrates a significant red shift of fluorescence to the higher wavelength (lower energy) from 480 to 600 nm for the prepared CQDs from Urea. On the other hand, it is essential to mention that a Filter was used to reduce the fluorescence intensity of the co-doped CQDs 10 times to obtain these results. The results showed a high fluorescence for the B, N-CQDs. This evident variation confirms that the B, N-CQDs have unique fluorescence properties and can be used as a promising Nanoprobe for variable applications.

This study discussed comparisons between B, N-CQDs, and CQDs from Urea, confirming that B, N-CQDs are better. A tiny amount and little concentration were enough to measure and get the desired results. Highly cost preparations are not required, which has an economic impact in real life. So we can say that B, N doped CQDs are promising fluorescence probes to detect variable antibiotics in aqueous solutions.

### CQDs as fluorescence probes sensing variable antibiotics in aqueous solutions

**Figure 6:**
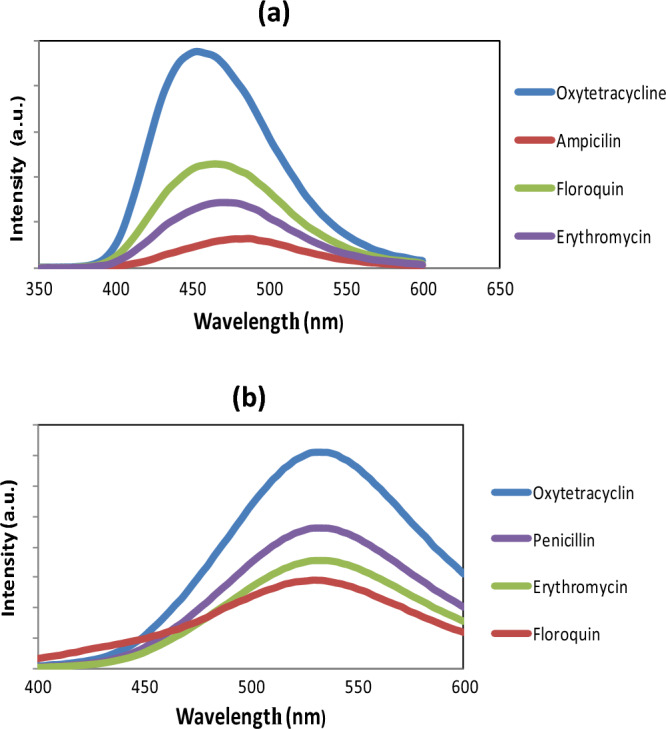
Representative fluorescence-quenching spectrum upon adding variable antibiotics for (**a**) B, N-CQDs, (**b**) CQDs.

The CQDs were utilized as fluorescence sensors for sensitive and selective determining of antibiotics, although to varying degrees, based on the benefits described above, which summarized the excellent photoluminescence performance of the as-prepared CQDs. Antibiotic residues in high amounts in aquaculture and drinking water pose a serious threat. The most often used antibiotics in Egyptian aquaculture are oxytetracycline hydrochloride, erythromycin, penicillin, and floriquin. These antibiotics were chosen as test subjects to study their light-sensing capabilities utilizing superior CQDs as a chemo-sensor. As a result, the fluorescence signal of the sensing system composed of CQDs and various antibiotics (A.B.) will be varied. The outcomes are described and depicted in Fig. 6.

With multiple antibiotics in the same analytical conditions, the fluorescence spectra of the B, N-CQD (A.B) detection system altered. penicillin and erythromycin have a higher fluorescence quenching response than the other antibiotics; however, they have varying degrees, Fig. 6a. In the case of CQDs, however, the results revealed that penicillin and oxytetracycline hydrochloride have a more significant fluorescence quenching effect than the other antibiotics, Fig. 6b. The results also demonstrated that the fluorescence quenching response of CQDs and the B, N co-doping CQDs sensing system could detect penicillin with exceptional sensitivity and selectivity. As a result, it is regarded as a new successful sensing probe that can distinguish between multiple types of antibiotics and one type and this makes our work distinct from the rest of the previous results.

**Figure 7:**
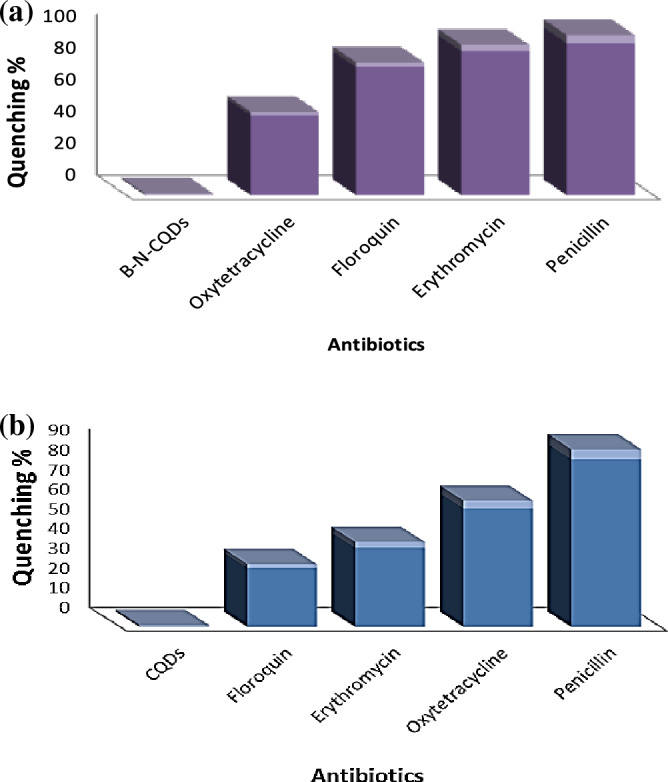
Quenching percentage diagram to demonstrate antibiotics selective efficiency for the investigated (**a**) B, N-CQDs, and (**b**) CQDs.

The results of the investigated B, N-CQD for various antibiotics are presented in Fig. 7a. the findings revealed that penicillin and erythromycin have a higher fluorescence quenching effect. According to the fluorescence quenching percentages plot, CQDs are more sensitive to detecting penicillin and erythromycin than any other antibiotic. CQDs made from Urea were more selective for penicillin and oxytetracycline hydrochloride Fig. 7b. It is crucial to note that, after comparing co-doped CQDs and CQDs, B, N-CQDs-based sensors will be more effective than CQDs and because of their specific, highly fluorescent properties (Filters were utilised to reduce the fluorescence intensity by ten fold).

### The detection mechanism and the fluorescence response of CQDs to analytes (antibiotics)

(a) Most studies believe that the carbon-core states, surface defect states, and molecular states mostly cause the complex fluorescence characteristic of CQDs.

- The carbon-core states caused by the separation of the valence and conduction band of the π-domain, mainly from the graphene core,
- The surface defect state is the primary fluorescence mechanism of the most studied CQDs produced by different surface functional groups. Combining these numerous functional groups produces excitation-dependent emission, multicolor emissions, and complicated radiative relaxation. Furthermore, heteroatomic doping with elements like N, S, and B enhances radiative recombination and increases the quantum yield of surface defect states^30^.
- The fluorophores or chromophores on the surface of CQDs cause the fluorescent molecular states. The molecular states were exhibited behavior identical of organic dyes, which frequently exhibit high quantum yield, apparentr, strong fluorescence, and weak photobleaching resistance.
  Fluorophores, as chromophores, are compounds that absorb light in the UV or visible spectrum. They re-emit some of the light as radiation, which this process is called fluorescence.
  - Fluorescence quenching that may be used for analyte detection can result from specific interactions of CQDs with various molecules, including metal ions, antibiotics, and proteins^31^.
- The quenching process can be static or dynamic, depending on the quenching system. The quencher and the fluorescence probe form a non-fluorescent connection during the static quenching procedure. The number of these complexes increases as the concentration of quencher molecules [Q] increases. However, quencher concentrations do not affect the corresponding fluorescence lifetime (fluorescence decay rate).
- A recommended quenching process for CQDs is static quenching, which happens when they come into contact with analytes and generate a new ground state of the analyte-CQDs complex due to a strong bonding connection. After the CQDs have been stimulated, the fluorescence can also be quenched by dynamic collision with the quencher, resulting in non-radiative energy transfer^32^.
- In dynamic quenching, the quencher molecule collides with the fluorophore while it is stimulated, causing the quencher concentrations to change. As a result, the change in K_SV_ and the associated lifetime value *T* decrease as [Q] quencher concentrations increase according to the following equation^33^:
  2 $${\varvec{K}}_{{{\varvec{SV}}}} = \, {\varvec{Kq}} \cdot \, {\varvec{T}}$$
  ***K***_***SV***_ is the Stern–Volmer constant, and ***T*** is the fluorescence decay rate.
  (b) Photo-induced electron transfer (PET) can also take place between the CQDs and the analyte. When analyte and electron-rich/poor CQDs interact, an electron may move from one structure to another. Because of this, the electrons are forced to take a different path (electron dropped from the lowest unoccupied molecular orbital (LUMO) to the highest occupied molecular orbital (HOMO) of the CQDs. PET may be divided into two categories: oxidative electron transfer, in which CQDs function as the electron donor, and reductive electron transfer, in which CQDs act as the electron receiver. The electron typically moves from the LUMO of the CQDs to the LUMO of the quencher during oxidative electron transfer. For reductive electron transfer from the quencher’s LUMO to the CQDs’ HOMO, both situations often result in reduced fluorescence^34^.

The detection of analytes did not only depend on a contact-based quenching process. There are further non-contact methods that may be used to detect a variety of analytes, including the inner filter effect (IFE), fluorescence and chemiluminescence resonance energy transfer (FRET and CRET), and others^35^.

### The effect of penicillin and erythromycin concentrations on the fluorescence spectra of the B, N-CQDs

**Figure 8:**
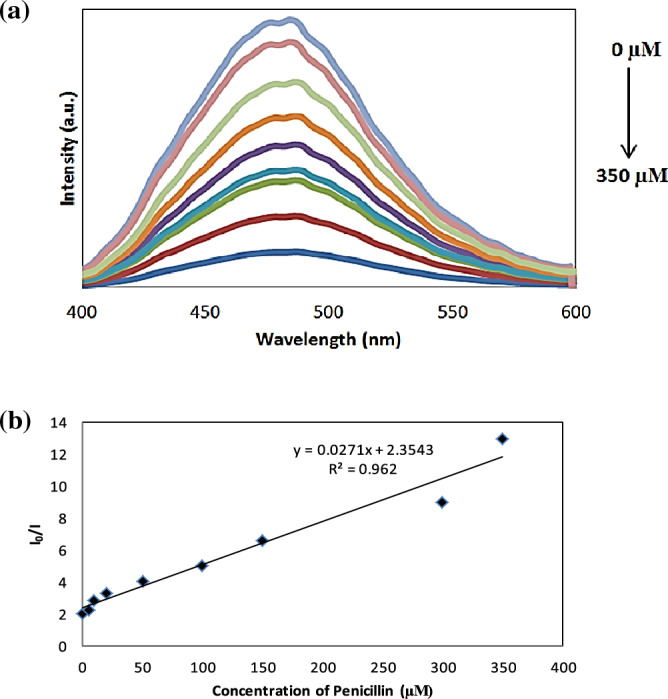
(**a**) PL spectra of B, N-CQDs upon the addition of different concentrations of Penicillin and (**b**) Plot of fluorescence intensity ratios of I_o_/I versus the concentration of Penicillin (µM) in the range from 0 µM to 350 µM.

Different concentrations of penicillin and erythromycin standard solutions were added to the B, N CQDs solutions under optimal experimental conditions. It’s important to mention that the PL measurements of the analyzed B and N-CQDs were examined using a filter. With varied penicillin concentrations, the detecting system altered. The penicillin concentrations in the 1 mL detection system were 0, 5.8, 10.3, 23.2, 47.68, 93.4, 143.04, 300, and 350 µM, as shown in Fig. 8a. When the penicillin concentration is raised under the same analytical conditions, the fluorescence intensity in the sensing system steadily decreases.

**Figure 9:**
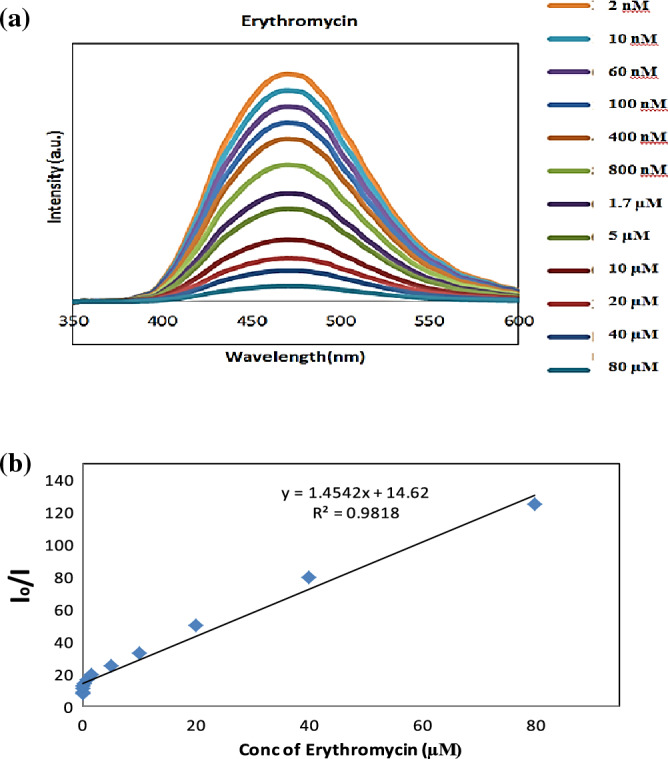
(**a**) FL spectra of B, N-CQDs upon the addition of different concentrations of Erythromycin and (**b**) Plot of fluorescence intensity ratios of I_o_/I versus the concentration of Erythromycin (µM) in the range from 2 nM to 80 µM.

On the other hand, Fig. 9a depicted the relation between erythromycin concentration and fluorescence intensity in a 1 mL detection system, with erythromycin concentrations of 2, 10, 60, 100, 400, 800 nM, 1.7, 5, 10, 20, 40 and 80 µM. Fluorescence quenching increases when concentration rises, according to the observations.

It’s crucial to note that penicillin and erythromycin quenched the fluorescence intensity of B, N CQDs, and the fluorescence quenching increased as antibiotic concentrations increased. Furthermore, this confirms B, N CQDs’ unrivaled sensitivity. Consequently, it’s considered a novel successful detecting probe with that has an excellent sensitivity to low concentrations of antibiotics (Supplementary Information 3).

The sensitivity of B, N-CQDs to antibiotics such as Penicillin and Erythromycin was tested under perfect experimental conditions, and the results are shown in Figs. 8b, 9b. The fluorescence intensity (I_o_/I) increases as the concentration of antibiotics increases, where I_o_ and I were the fluorescence intensity of B, and N CQDs in the absence and presence of antibiotics, respectively. The concentration ranges for penicillin and erythromycin were 0 to 350 µM and 2 nM to 60 µM, respectively. Within the limits of the linear regression equation, the plot revealed an excellent linear relationship and the linear correlation coefficient (R^2^) was 0.962 and 0.9818, respectively. Additionally, it was determined that the detection limit was 10 nM and 5 nM based on 3σ/S, where σ and S represent the standard deviation and slope of the curve, respectively. This work’s analytical performance is given in Table 1.

**Table 1:** B, N co-doping CQDs was quenched by penicillin and erythromycin.

Antibiotics	Linear regression equations	Correlation coefficient R^2^	Linear range	Limit of detection (LOD)
*Penicillin*	I_o_/I = 0.0271[Q] + 2.3543	0.9620	0–350 µM	10 µM
*Erythromycin*	I_o_/I = 1.4542[Q] + 14.62	0.9818	2 nM–80 µM	5 nM

The detection limit is defined by the equation LOD = 3δ/S where δ is the standard deviation (n = 10), and S is the slope of the calibration plot.

The synthesized B, N-CQDs provide active sites and a high surface area/volume ratio for the adsorption of antibiotic drugs. Additionally, it can alter the band gap, resulting in a rise in fluorescence that might be used to make sensors^36^.

It is generally known that the adsorption rate is most substantially influenced by the proportion of aromatic rings in antibiotics^37^. Antibiotics with various aromatic rings enhance their beinding capacity to B, N-CQD surfaces. This suggests that the number of aromatic rings an antibiotic possesses will affect how rapidly it can bind to carbon-based materials. As was previously mentioned, the main mechanisms by which antibiotics attach to B, N-CQDs is by π–π-interaction^38^. According to the reasons provided B, N-CQDs would be among the best dual-functional nanosensors for the foreseeable future.

Phenolic derivatives and phenolic compound pollution are two of the most prevalent challenges that the world’s population faces. Because of their toxicity and capacity to persist in the environment for extended periods, it is critical to monitor the amount of phenolic compounds in our environment. We were interested in employing CQDs to detect phenolic chemicals in this study.

**Figure 10:**
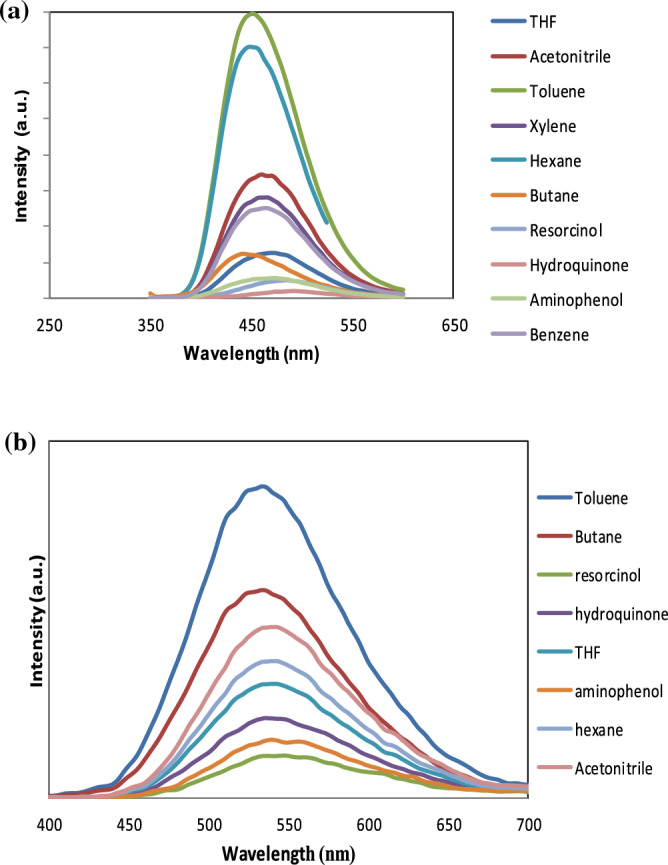
Representative fluorescence-quenching spectrum upon adding variable organic compounds and phenolic derivatives for (**a**) B, N-CQDs, and (**b**) Carbon quantum dots (CQDs).

**Figure 11:**
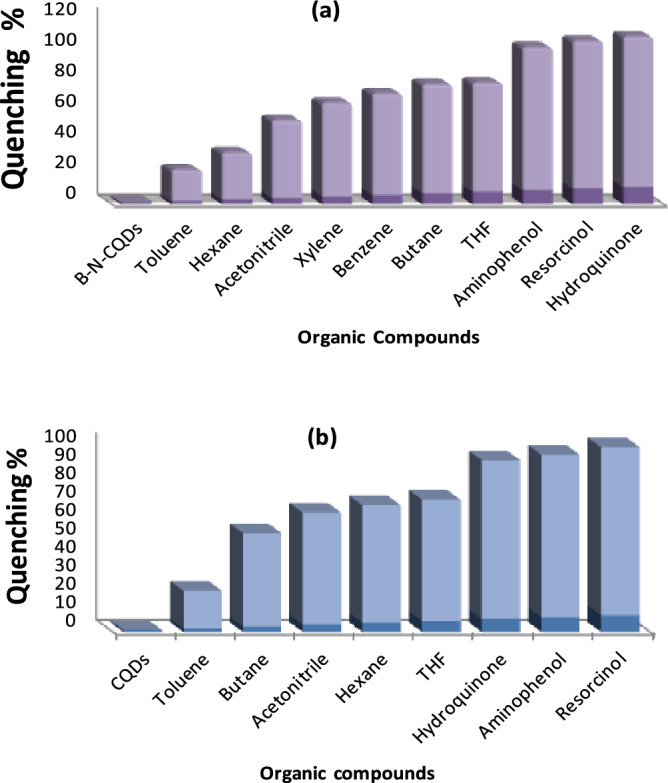
Quenching percentage diagram to demonstrate the selective efficiency of different organic compounds and phenolic derivatives for the investigated (**a**) B, N-CQDs (**b**) CQDs.

The unique CQDs studied to play a vital role in detecting phenol analogs (para-aminophenol, resorcinol, and hydroquinone), which are well-known contaminants. Figure 10 displays the findings for B, N-CQDs, and CQDs as promising sensors for phenol detection, and we assess their selectivity and sensitivity in various solvents. Figure 10a showed that the fluorescence intensity of B, N co-doping CQDs changed slightly with the addition of phenolic derivatives, indicating their high selectivity for hydroquinone detection over resorcinol and then aminophenol. CQDs have the maximum sensitivity for detecting phenol derivatives more than any other organic solvents under the same conditions. Figure 10b showed that the CQDs have more sensitivity to the phenolic compounds than any other organic solvent and are more selective to Resorcinol than others. According to the results, quenching percentage diagrams demonstrate the particular efficiency of the examined CQDs for a range of phenolic derivatives compounds (hydroquinone, resorcinol, and para-aminophenol). Because of their phenolic chemical structure, they had a higher fluorescence quenching response than the other organic solvents, implying that CQDs are more sensitive to detecting phenolic derivatives chemicals than any other organic solvent. In the case of B, N co-doping CQDs, Fig. 11a. Hydroquinone has a higher fluorescence quenching effect, according to the findings. CQDs, on the other hand, were more selective for Resorcinol Fig. 11b.

After comparing co-doping CQDs and CQDs, it is essential to mention that B, N-CQDs based sensors will be more affiant than CQDs because of their distinctive characteristic high fluorescence properties (filter was needed to decrease the fluorescence 10 times). So B, N-CQDs were expected as the most promising probe for phenols derivatives detection.

### Comparison of FT-IR data for B, N-CQDs with erythromycin, hydroquinone, and PAP (80 µM)

**Figure 12:**
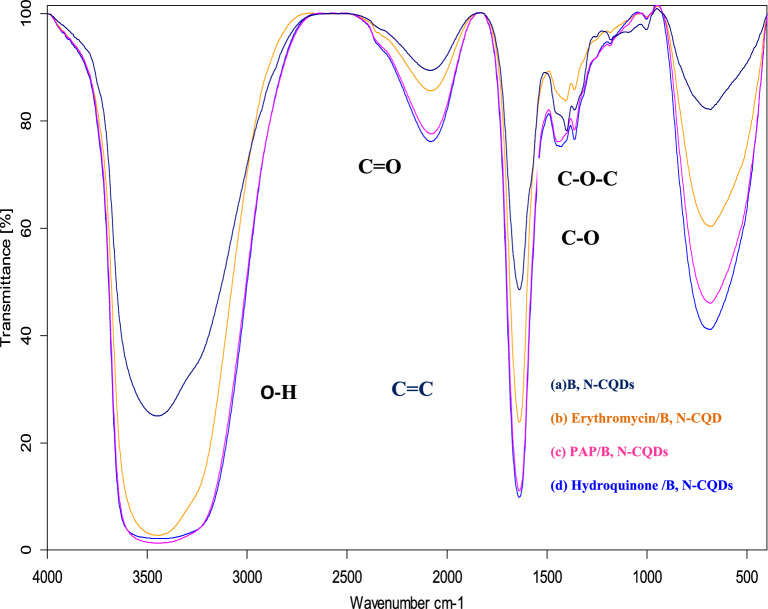
FT-IR spectra comparison of B, N-CQDs and different analytes.

The FT-IR spectrum displayed the comparison between pure B, N doping CQDs and different peaks that represent some functional groups located on the surface of B, N-CQDs with target analytes Fig. 12. Stretching vibration of C–OH (3400 cm^−1^) and C=C (1700 cm^−1^). Additionally, vibrational absorption bands corresponding to C=O and C–O–C were recorded at 1700 cm^−1^, 1500 cm^−1^, and 1250 cm^−1^^39^ .

Based on previous data, PL gives more accurate, precise, and detailed results than FTIR to study the CQDs fluorescence with different analytes, even with low concentrations (conc. < 80 µM), where fluorescence spectrometry is one of the most promising analytical methods.

### The effect of different phenolic derivatives concentrations on the fluorescence spectra of the B, N-CQDs

**Figure 13:**
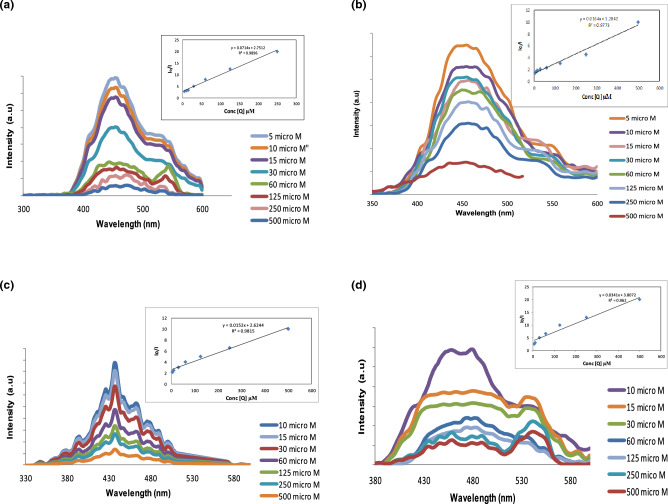
PL spectra of B, N-CQDs and fluorescence intensity ratios of I_o_/I versus the concentration in the range from 500 µM to 5µM of (**a**) Hydroquinone, (**b**) resorcinol, (**c**) Para aminophenol and (**d**) Hexane.

The detection of phenol derivatives chemicals was explored using the acquired B, N co-doping CQDs. For PL measurements, different concentrations of phenol derivatives ranging from 5 to 500 µM were introduced to an aqueous solution of B, N-CQDs. As shown in Fig. 13, at excitation wavelength 330 nm, the fluorescence intensity of the B, N-CQDs diminishes as the concentration of phenol derivatives compounds increases. This finding implies that phenol derivatives like hydroquinone, resorcinol, and para aminophenol can quench B, N-CQD fluorescence.

The connection between fluorescence quenching value (I_o_/I) and the concentration of phenol derivatives [Q] is shown in Fig. 13. (I_o_ and I are the fluorescence intensity of B, N-CQDs at 330 nm in the absence and the presence of phenol derivatives compounds). The Fluorescence plot (Fig. 13a–c) shows an excellent linear correlation with hydroquinone, resorcinol, and para aminophenol, with concentrations ranging from 5 µM to 500 µM. The corresponding regression coefficient (R^2^) value was 0.989, 0.977, and 0.981, respectively, confirming the linear relationship. The limit of detection (LOD) was calculated to be 0.05, 0.024, and 0.032 µM based on the formula LOD = 3δ/S, where S is the slope of the linear calibration plot, and δ is the standard deviation of the blank signal.

B, N co-doping CQDs were not limited to determining phenol derivatives. Still, it can also identify organic solvents, and it is clear from the plot in Fig. 13d that the quenching rises as the concentration of Hexane (an example of an organic solvent) increases. The plot demonstrated in Fig. 13d confirms the results. An excellent linear relationship can be observed with a linear range of 5-500 µM, an R^2^ value of 0.9707 and a limit of detection (LOD) is 0.013 µM. The complete data are summarized in Table 2.

**Table 2:** B, N co-doping CQDs quenched by hydroquinone, resorcinol, para aminophenol, and hexane.

Phenol derivatives & organic solvent	Linear regression equations	Correlation coefficient R^2^	Linear range µM	Limit of detection (LOD)
*Hydroquinone*	I_o_/I = 0.0714[Q] + 2.751	**0.9896**	5–500	0.050 µM
*Resorcinol*	I_o_/I = 0.0164[Q] + 1.2842	**0.9773**	5–500	0.024 µM
*Para aminophenol*	I_o_/I = 0.0152[Q] + 2.6244	**0.9815**	5–500	0.032 µM
*Hexane*	I_o_/I = 0.0341[Q] + 3.807	**0.962**	5–500	0.013 µM

The detection limit is defined by the equation LOD = 3δ/S where δ is the standard deviation (n = 10), and S is the slope of the calibration plot.

#### A mechanism for the fluorescence response of CQDs to phenolic derivatives

The multiple carboxyl groups on the surface of CQDs enable the formation of hydrogen bonds with phenol, resulting in remarkable selectivity and sensitivity for phenol detection. Density functional theory (DFT) and time-dependent density functional theory (TDDFT) research have shown that the hydrogen bonds (C=O•••H–O) between phenol and fluorescent CQDs are what cause the CQDs’ ability to quench fluorescence. Complex (CQDs @ phenol) was the hydrogen bond model that was the most stable. The O atom of CQDs and the H atom of phenol create a hydrogen bond, which allows for a remarkable sensitivity for phenol detection.

The analytical performance of this investigation was compared to prior detection methods for antibiotics and phenol derivatives chemicals, and the results were given in Tables 3, 4. The results revealed that this experiment’s detection limit is lower than most previously published approaches, the linear range is broad, and no sophisticated chemical modification is necessary. Our sensing device outperforms conventional fluorescent nanoprobes to detect antibiotics and phenol derivatives accurately. Consequently, it is classified as a dual-functional nanosensor. But the others didn’t have that powerful performance. The sensitivity of a probe for detecting phenolic derivatives, pesticides, and antibiotics, as well as variable drugs, is influenced by several factors. These factors include the surface functional groups on the CQDs and the quenching and binding constants of the target pesticides or antibiotics. Antibiotic sensors based on CQDs, on the other hand, have received the least attention and require additional investigation. The chemical interaction between CQDs and certain compounds is the leading cause of the fluorescence being quenched. CQD-based fluorescence sensors are effective in Antibiotic-detection and practical for phenol derivatives and pesticide detection.

**Table 3:** Comparison of sensing performance of variables Nanoprobes for detecting antibiotics.

Detection probe	Detected antibiotic	Method	Linear range (µM)	Limit of detection (LOD)	Year	References
A fiber optic SPR sensor (surface plasmon resonance)	Erythromycin	Molecular imprinted nanoparticles	0–50 µM	5.32 µM	2016	^40^
Rhombus porous carbon (RPC)	Penicillin	electrochemical	10^–8^ to 10^–5^ mg mL^−1^	2.68 * 10^–7^ mg/mL	2021	^41^
detection of H^+^ ions emerged by H^+^ generating enzymes	Penicillin	Photoelectrochemical Biosensors (PEC)			2021	^42^
CNT- Fe_3_O_4_@SiO_2_	Tetracycline	(AdSDPV) Adsorptive stripping Differential pulse Voltammetry	400–460 µM	400 nM	2021	^43^
Graphene-Au	Tetracycline	CV (cyclic voltammetry)	29–135 µM	16 nM	2021	^44^
Halide-directed Assembly of Mercury(II) Coordination Polymers	Penicillin	Electrochemical biosensor			2020	^45^
SiO_2_/Au	Erythromycin	Fluorescence		12 nM	2017	^46^
B, N co-doping CQDs	Penicillin & Erythromycin	Fluorescence	0–350 µM 2 nM-80 µM	10 nM 5 nM		This work

**Table 4:** Comparison of sensing performance of variables Nanoprobes for detecting some phenolic derivatives.

Detection probe	Detected analyte	Method	Detection range	LOD	Year	References
2-(phenyl azo) chromotropic acid-(CH–) conducting polymer (PCH/AGCE)	Hydroquinone	Electrochemical	0.066 μM	1–300 μM	2019	^47^
Carboxylated multi-walled carbon nanotubes (MWCNT-COOH)	Hydroquinone	Electrochemical	2–280 μM	0.017	2021	^48^
Diazo-coupling reaction	Paraaminophenol	Micro determination	0.0297–0.2229 µg/mL	0.0185 µg/mL	2019	^49^
Au gold nanoparticle@dithiooxamide functionalized reduced graphene oxide (AuNP@DFG)	Paraaminophenol	Electrochemical	0.1–130 μM	11 nM	2019	^50^
The peroxidase-like activity of Fe_3_O_4_@Au/MOF	Paraaminophenol	Colorimetric	0.5–20 μM	0.27 μM	2022	^51^
Carbon nanotubes (MWCNT)	Hydroquinone	Electrochemical square wave voltammetry (SWV)	1.0 × 10^–5^ M to 1.0 × 10^–3^ M	5.7 × 10^–6^ M	2017	^52^
Single-walled carbon nanotube	Hexane	Electrochemical			2019	^53^
B, N co-doping CQDs	Hydroquinone Resorcinol Para aminophenol Hexane		5–500 µM	0.05 µM 0.024 µM 0.032 µM 0.013 µM		This work

## Conclusion

In conclusion, we have successfully developed a unique technique for producing highly fluorescent dual functionalities probes and demonstrated their benefits in terms of simplicity, cost efficiency, and environmental friendliness. CQDs were synthesized using two distinct ways in this study. Synthesis of unique high-fluorescence B, N-CQD probes has also been a promising sensor. Both antibiotics and phenol derivatives may be detected using the suggested nanosensor. The detection is dependent on the quenching of fluorescence. The quenching percentages represent the sensitivity of the produced B, N-CQDs Nanoprobe, with the most sensitive antibiotics being Penicillin and Erythromycin. The task of the Nanoprobe under examination includes not only the detection of antibiotics but also the sensitive detection of dangerous phenolic derivatives like hydroquinone, resorcinol, and para aminophenol as organic solvents like Hexane and other comparable chemicals. The surface states of the carbon dots regulate the photoluminescence properties; therefore, the fluorescence can be attributed to them. The hydrogen bonds (CQO•••H–O) between phenolic compounds and fluorescent CQDs are responsible for the effect of CQDs on phenolic derivatives.

The results show that as concentration increases, the fluorescence of the high luminescence B, N-CQDs progressively diminishes, indicating that the current sensing device is susceptible. The experimental observations may be a significant step toward creating a promising probe that will serve as the optimal sensor. This new Fluorescence quenching-based method is sensitive, less time-consuming and requires no costly preparations to replace the widely used chromatographic detection method.

### Supplementary Information

Supplementary Information 1.

Supplementary Information 2.

Supplementary Information 3.

## Data Availability

The data used and analyzed during the current study are available from the corresponding authors upon reasonable request.
