# Supplementary material for: Pure and doped carbon quantum dots as fluorescent probes for the detection of phenol compounds and antibiotics in aquariums
Source: Sci Rep. 2023 Aug 8;13:12863. doi: 10.1038/s41598-023-39490-y (PMC10409781; doi:10.1038/s41598-023-39490-y)
Supplement: Supplementary file 1 — Supplementary Information 1. [file 41598_2023_39490_MOESM1_ESM.pdf]

Commander Sample ID (Coupled TwoTheta/Theta)

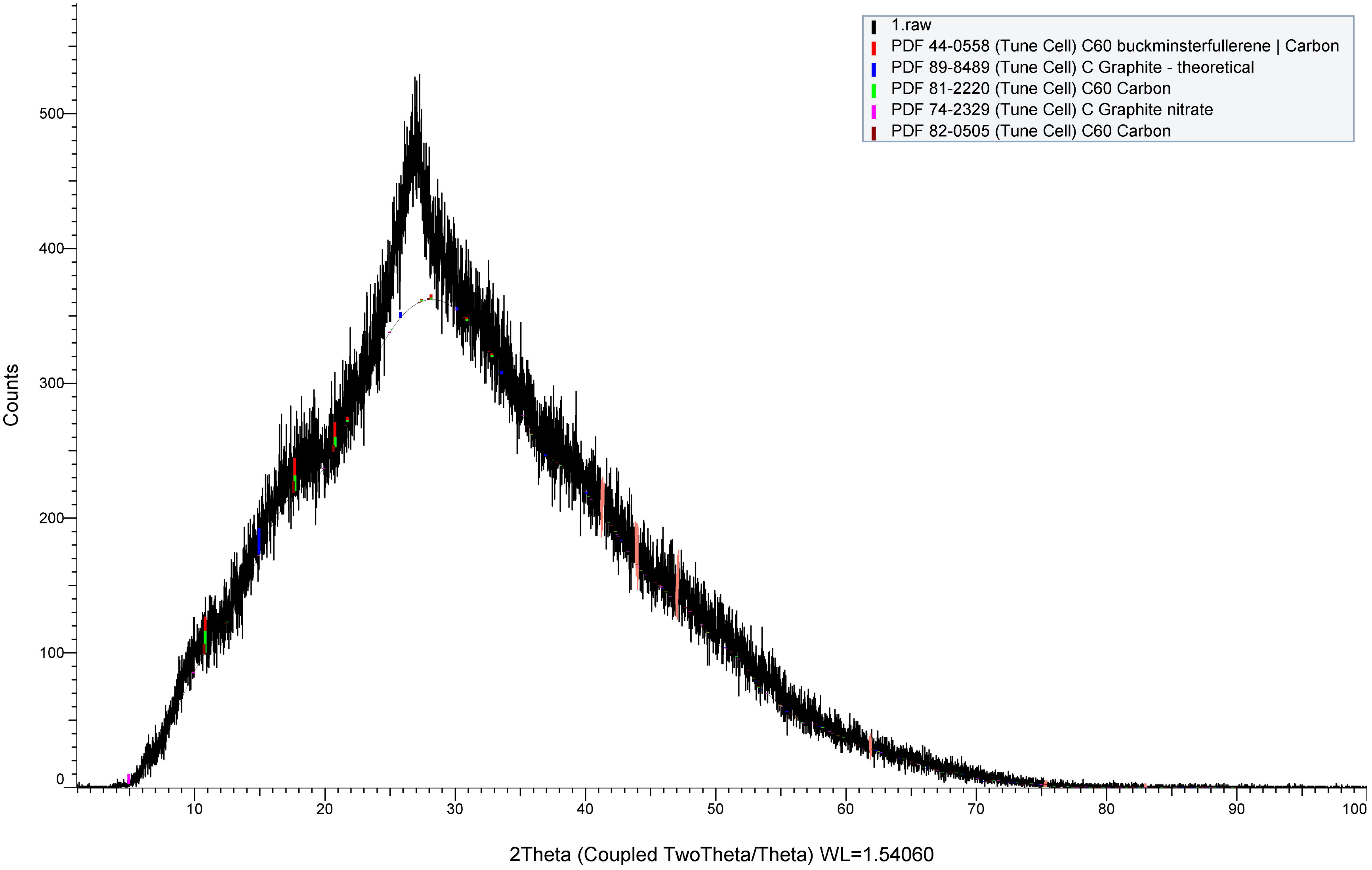

1.raw #1

X	d (Å)	Y (counts)	dY	Time (s)
0.998	88.43782	2.00	1.41	19.2
1.008	87.55197	0	0	19.2
1.018	86.68368	0	0	19.2
1.028	85.83245	0	0	19.2
1.039	84.99778	0	0	19.2
1.049	84.17918	0	0	19.2
1.059	83.37620	0	0	19.2
1.069	82.58840	0	0	19.2
1.079	81.81534	0	0	19.2
1.089	81.05662	0	0	19.2
1.099	80.31185	0	0	19.2
1.109	79.58064	0	0	19.2
1.119	78.86263	0	0	19.2
1.129	78.15745	0	0	19.2
1.140	77.46478	4.00	2.00	19.2
1.150	76.78427	0	0	19.2
1.160	76.11562	0	0	19.2
1.170	75.45852	0	0	19.2
1.180	74.81266	1.00	1.00	19.2
1.190	74.17776	0	0	19.2
1.200	73.55356	0	0	19.2
1.210	72.93977	0	0	19.2
1.220	72.33614	1.00	1.00	19.2
1.230	71.74241	0	0	19.2
1.240	71.15836	0	0	19.2
1.251	70.58374	0	0	19.2
1.261	70.01833	0	0	19.2
1.271	69.46190	0	0	19.2
1.281	68.91425	0	0	19.2
1.291	68.37517	0	0	19.2
1.301	67.84445	0	0	19.2
1.311	67.32191	0	0	19.2
1.321	66.80736	0	0	19.2
1.331	66.30062	0	0	19.2
1.341	65.80151	0	0	19.2
1.352	65.30985	0	0	19.2
1.362	64.82549	1.00	1.00	19.2
1.372	64.34826	2.00	1.41	19.2
1.382	63.87801	0	0	19.2
1.392	63.41457	0	0	19.2
1.402	62.95782	0	0	19.2
1.412	62.50760	0	0	19.2
1.422	62.06377	0	0	19.2
1.432	61.62621	0	0	19.2
1.442	61.19476	0	0	19.2
1.453	60.76932	1.00	1.00	19.2

1.463	60.34976	0	0	19.2
1.473	59.93594	1.00	1.00	19.2
1.483	59.52777	0	0	19.2
1.493	59.12512	0	0	19.2
1.503	58.72787	0	0	19.2
1.513	58.33594	1.00	1.00	19.2
1.523	57.94919	0	0	19.2
1.533	57.56755	0	0	19.2
1.543	57.19089	1.00	1.00	19.2
1.554	56.81914	1.00	1.00	19.2
1.564	56.45218	0	0	19.2
1.574	56.08994	0	0	19.2
1.584	55.73232	0	0	19.2
1.594	55.37922	0	0	19.2
1.604	55.03058	0	0	19.2
1.614	54.68630	0	0	19.2
1.624	54.34629	0	0	19.2
1.634	54.01050	1.00	1.00	19.2
1.644	53.67882	0	0	19.2
1.655	53.35119	0	0	19.2
1.665	53.02754	0	0	19.2
1.675	52.70780	0	0	19.2
1.685	52.39188	0	0	19.2
1.695	52.07974	1.00	1.00	19.2
1.705	51.77129	0	0	19.2
1.715	51.46647	0	0	19.2
1.725	51.16522	0	0	19.2
1.735	50.86748	0	0	19.2
1.745	50.57318	1.00	1.00	19.2
1.756	50.28227	0	0	19.2
1.766	49.99468	0	0	19.2
1.776	49.71037	1.00	1.00	19.2
1.786	49.42928	0	0	19.2
1.796	49.15134	0	0	19.2
1.806	48.87652	2.00	1.41	19.2
1.816	48.60475	0	0	19.2
1.826	48.33598	0	0	19.2
1.836	48.07018	0	0	19.2
1.846	47.80728	0	0	19.2
1.857	47.54724	0	0	19.2
1.867	47.29001	0	0	19.2
1.877	47.03556	0	0	19.2
1.887	46.78382	1.00	1.00	19.2
1.897	46.53477	0	0	19.2
1.907	46.28836	0	0	19.2
1.917	46.04454	3.00	1.73	19.2
1.927	45.80328	0	0	19.2
1.937	45.56453	0	0	19.2
1.947	45.32826	0	0	19.2
1.958	45.09443	0	0	19.2

1.968	44.86300	1.00	1.00	19.2
1.978	44.63393	0	0	19.2
1.988	44.40719	1.00	1.00	19.2
1.998	44.18274	0	0	19.2
2.008	43.96055	0	0	19.2
2.018	43.74058	1.00	1.00	19.2
2.028	43.52281	1.00	1.00	19.2
2.038	43.30719	0	0	19.2
2.048	43.09370	0	0	19.2
2.059	42.88230	1.00	1.00	19.2
2.069	42.67297	0	0	19.2
2.079	42.46567	1.00	1.00	19.2
2.089	42.26038	0	0	19.2
2.099	42.05706	0	0	19.2
2.109	41.85569	0	0	19.2
2.119	41.65624	0	0	19.2
2.129	41.45868	0	0	19.2
2.139	41.26299	0	0	19.2
2.149	41.06913	0	0	19.2
2.160	40.87709	0	0	19.2
2.170	40.68684	0	0	19.2
2.180	40.49835	0	0	19.2
2.190	40.31159	1.00	1.00	19.2
2.200	40.12656	1.00	1.00	19.2
2.210	39.94321	0	0	19.2
2.220	39.76154	0	0	19.2
2.230	39.58150	0	0	19.2
2.240	39.40309	0	0	19.2
2.250	39.22629	0	0	19.2
2.261	39.05106	0	0	19.2
2.271	38.87739	0	0	19.2
2.281	38.70526	0	0	19.2
2.291	38.53465	0	0	19.2
2.301	38.36553	1.00	1.00	19.2
2.311	38.19790	0	0	19.2
2.321	38.03172	0	0	19.2
2.331	37.86698	0	0	19.2
2.341	37.70367	0	0	19.2
2.351	37.54175	0	0	19.2
2.362	37.38122	0	0	19.2
2.372	37.22206	1.00	1.00	19.2
2.382	37.06425	0	0	19.2
2.392	36.90777	0	0	19.2
2.402	36.75261	0	0	19.2
2.412	36.59875	1.00	1.00	19.2
2.422	36.44617	0	0	19.2
2.432	36.29486	0	0	19.2
2.442	36.14479	0	0	19.2
2.452	35.99597	0	0	19.2
2.463	35.84836	0	0	19.2

2.473	35.70197	0	0	19.2
2.483	35.55676	1.00	1.00	19.2
2.493	35.41273	1.00	1.00	19.2
2.503	35.26986	0	0	19.2
2.513	35.12814	1.00	1.00	19.2
2.523	34.98755	0	0	19.2
2.533	34.84809	0	0	19.2
2.543	34.70973	0	0	19.2
2.553	34.57247	0	0	19.2
2.563	34.43629	0	0	19.2
2.574	34.30118	0	0	19.2
2.584	34.16713	0	0	19.2
2.594	34.03411	0	0	19.2
2.604	33.90214	0	0	19.2
2.614	33.77118	1.00	1.00	19.2
2.624	33.64122	0	0	19.2
2.634	33.51227	0	0	19.2
2.644	33.38430	1.00	1.00	19.2
2.654	33.25730	0	0	19.2
2.664	33.13127	0	0	19.2
2.675	33.00619	0	0	19.2
2.685	32.88205	1.00	1.00	19.2
2.695	32.75884	0	0	19.2
2.705	32.63655	0	0	19.2
2.715	32.51517	0	0	19.2
2.725	32.39469	0	0	19.2
2.735	32.27510	1.00	1.00	19.2
2.745	32.15639	0	0	19.2
2.755	32.03855	0	0	19.2
2.765	31.92158	0	0	19.2
2.776	31.80545	0	0	19.2
2.786	31.69016	0	0	19.2
2.796	31.57571	1.00	1.00	19.2
2.806	31.46208	1.00	1.00	19.2
2.816	31.34927	0	0	19.2
2.826	31.23726	0	0	19.2
2.836	31.12605	1.00	1.00	19.2
2.846	31.01563	1.00	1.00	19.2
2.856	30.90599	0	0	19.2
2.866	30.79713	0	0	19.2
2.877	30.68903	1.00	1.00	19.2
2.887	30.58168	0	0	19.2
2.897	30.47508	0	0	19.2
2.907	30.36923	0	0	19.2
2.917	30.26411	0	0	19.2
2.927	30.15971	1.00	1.00	19.2
2.937	30.05603	0	0	19.2
2.947	29.95306	0	0	19.2
2.957	29.85079	0	0	19.2
2.967	29.74923	0	0	19.2

2.978	29.64835	0	0	19.2
2.988	29.54815	0	0	19.2
2.998	29.44862	0	0	19.2
3.008	29.34977	0	0	19.2
3.018	29.25158	1.00	1.00	19.2
3.028	29.15404	0	0	19.2
3.038	29.05715	0	0	19.2
3.048	28.96090	1.00	1.00	19.2
3.058	28.86529	0	0	19.2
3.068	28.77031	0	0	19.2
3.079	28.67595	0	0	19.2
3.089	28.58221	1.00	1.00	19.2
3.099	28.48908	0	0	19.2
3.109	28.39655	0	0	19.2
3.119	28.30463	1.00	1.00	19.2
3.129	28.21330	0	0	19.2
3.139	28.12255	1.00	1.00	19.2
3.149	28.03239	0	0	19.2
3.159	27.94280	1.00	1.00	19.2
3.169	27.85379	0	0	19.2
3.180	27.76534	1.00	1.00	19.2
3.190	27.67745	0	0	19.2
3.200	27.59011	0	0	19.2
3.210	27.50333	1.00	1.00	19.2
3.220	27.41709	0	0	19.2
3.230	27.33139	0	0	19.2
3.240	27.24622	0	0	19.2
3.250	27.16158	0	0	19.2
3.260	27.07747	1.00	1.00	19.2
3.270	26.99387	0	0	19.2
3.281	26.91080	0	0	19.2
3.291	26.82823	0	0	19.2
3.301	26.74616	0	0	19.2
3.311	26.66460	0	0	19.2
3.321	26.58353	0	0	19.2
3.331	26.50296	0	0	19.2
3.341	26.42287	0	0	19.2
3.351	26.34327	0	0	19.2
3.361	26.26414	0	0	19.2
3.371	26.18549	0	0	19.2
3.382	26.10731	1.00	1.00	19.2
3.392	26.02959	1.00	1.00	19.2
3.402	25.95233	0	0	19.2
3.412	25.87554	1.00	1.00	19.2
3.422	25.79919	0	0	19.2
3.432	25.72330	1.00	1.00	19.2
3.442	25.64785	0	0	19.2
3.452	25.57284	0	0	19.2
3.462	25.49827	0	0	19.2
3.472	25.42413	1.00	1.00	19.2

3.483	25.35042	1.00	1.00	19.2
3.493	25.27714	1.00	1.00	19.2
3.503	25.20429	0	0	19.2
3.513	25.13185	0	0	19.2
3.523	25.05982	0	0	19.2
3.533	24.98821	2.00	1.41	19.2
3.543	24.91701	0	0	19.2
3.553	24.84621	0	0	19.2
3.563	24.77581	0	0	19.2
3.573	24.70581	0	0	19.2
3.584	24.63621	0	0	19.2
3.594	24.56700	0	0	19.2
3.604	24.49817	0	0	19.2
3.614	24.42973	0	0	19.2
3.624	24.36167	0	0	19.2
3.634	24.29399	0	0	19.2
3.644	24.22669	1.00	1.00	19.2
3.654	24.15975	1.00	1.00	19.2
3.664	24.09319	0	0	19.2
3.674	24.02699	1.00	1.00	19.2
3.685	23.96115	0	0	19.2
3.695	23.89568	0	0	19.2
3.705	23.83056	2.00	1.41	19.2
3.715	23.76580	0	0	19.2
3.725	23.70138	0	0	19.2
3.735	23.63732	1.00	1.00	19.2
3.745	23.57360	1.00	1.00	19.2
3.755	23.51022	0	0	19.2
3.765	23.44719	1.00	1.00	19.2
3.775	23.38449	3.00	1.73	19.2
3.786	23.32213	1.00	1.00	19.2
3.796	23.26009	0	0	19.2
3.806	23.19839	1.00	1.00	19.2
3.816	23.13701	1.00	1.00	19.2
3.826	23.07596	0	0	19.2
3.836	23.01523	0	0	19.2
3.846	22.95482	0	0	19.2
3.856	22.89472	1.00	1.00	19.2
3.866	22.83494	0	0	19.2
3.876	22.77547	1.00	1.00	19.2
3.886	22.71631	2.00	1.41	19.2
3.897	22.65746	1.00	1.00	19.2
3.907	22.59891	3.00	1.73	19.2
3.917	22.54066	0	0	19.2
3.927	22.48271	0	0	19.2
3.937	22.42506	0	0	19.2
3.947	22.36771	0	0	19.2
3.957	22.31065	0	0	19.2
3.967	22.25387	1.00	1.00	19.2
3.977	22.19739	2.00	1.41	19.2

3.987	22.14119	0	0	19.2
3.998	22.08528	2.00	1.41	19.2
4.008	22.02965	0	0	19.2
4.018	21.97430	1.00	1.00	19.2
4.028	21.91922	0	0	19.2
4.038	21.86442	0	0	19.2
4.048	21.80990	0	0	19.2
4.058	21.75564	1.00	1.00	19.2
4.068	21.70166	0	0	19.2
4.078	21.64794	6.00	2.45	19.2
4.088	21.59449	1.00	1.00	19.2
4.099	21.54130	0	0	19.2
4.109	21.48838	4.00	2.00	19.2
4.119	21.43571	0	0	19.2
4.129	21.38330	1.00	1.00	19.2
4.139	21.33115	1.00	1.00	19.2
4.149	21.27925	1.00	1.00	19.2
4.159	21.22760	1.00	1.00	19.2
4.169	21.17620	0	0	19.2
4.179	21.12506	1.00	1.00	19.2
4.189	21.07415	1.00	1.00	19.2
4.200	21.02350	4.00	2.00	19.2
4.210	20.97308	2.00	1.41	19.2
4.220	20.92291	4.00	2.00	19.2
4.230	20.87298	2.00	1.41	19.2
4.240	20.82328	1.00	1.00	19.2
4.250	20.77382	4.00	2.00	19.2
4.260	20.72460	2.00	1.41	19.2
4.270	20.67561	2.00	1.41	19.2
4.280	20.62685	0	0	19.2
4.290	20.57832	0	0	19.2
4.301	20.53001	1.00	1.00	19.2
4.311	20.48194	1.00	1.00	19.2
4.321	20.43409	1.00	1.00	19.2
4.331	20.38646	1.00	1.00	19.2
4.341	20.33905	1.00	1.00	19.2
4.351	20.29187	4.00	2.00	19.2
4.361	20.24490	0	0	19.2
4.371	20.19815	1.00	1.00	19.2
4.381	20.15161	1.00	1.00	19.2
4.391	20.10529	2.00	1.41	19.2
4.402	20.05918	1.00	1.00	19.2
4.412	20.01329	1.00	1.00	19.2
4.422	19.96760	2.00	1.41	19.2
4.432	19.92212	2.00	1.41	19.2
4.442	19.87685	2.00	1.41	19.2
4.452	19.83178	1.00	1.00	19.2
4.462	19.78692	3.00	1.73	19.2
4.472	19.74226	1.00	1.00	19.2
4.482	19.69780	1.00	1.00	19.2

4.492	19.65354	3.00	1.73	19.2
4.503	19.60948	1.00	1.00	19.2
4.513	19.56562	1.00	1.00	19.2
4.523	19.52195	3.00	1.73	19.2
4.533	19.47848	1.00	1.00	19.2
4.543	19.43520	1.00	1.00	19.2
4.553	19.39211	1.00	1.00	19.2
4.563	19.34921	6.00	2.45	19.2
4.573	19.30651	4.00	2.00	19.2
4.583	19.26399	3.00	1.73	19.2
4.593	19.22166	2.00	1.41	19.2
4.604	19.17951	4.00	2.00	19.2
4.614	19.13755	1.00	1.00	19.2
4.624	19.09577	2.00	1.41	19.2
4.634	19.05418	1.00	1.00	19.2
4.644	19.01276	4.00	2.00	19.2
4.654	18.97153	2.00	1.41	19.2
4.664	18.93047	3.00	1.73	19.2
4.674	18.88959	2.00	1.41	19.2
4.684	18.84889	3.00	1.73	19.2
4.694	18.80836	0	0	19.2
4.705	18.76801	1.00	1.00	19.2
4.715	18.72783	1.00	1.00	19.2
4.725	18.68782	2.00	1.41	19.2
4.735	18.64798	1.00	1.00	19.2
4.745	18.60831	3.00	1.73	19.2
4.755	18.56881	2.00	1.41	19.2
4.765	18.52948	1.00	1.00	19.2
4.775	18.49031	3.00	1.73	19.2
4.785	18.45131	2.00	1.41	19.2
4.795	18.41248	2.00	1.41	19.2
4.806	18.37381	3.00	1.73	19.2
4.816	18.33529	0	0	19.2
4.826	18.29695	3.00	1.73	19.2
4.836	18.25876	1.00	1.00	19.2
4.846	18.22073	2.00	1.41	19.2
4.856	18.18285	2.00	1.41	19.2
4.866	18.14514	5.00	2.24	19.2
4.876	18.10758	2.00	1.41	19.2
4.886	18.07018	1.00	1.00	19.2
4.896	18.03293	3.00	1.73	19.2
4.907	17.99584	4.00	2.00	19.2
4.917	17.95889	1.00	1.00	19.2
4.927	17.92210	5.00	2.24	19.2
4.937	17.88546	8.00	2.83	19.2
4.947	17.84897	2.00	1.41	19.2
4.957	17.81263	4.00	2.00	19.2
4.967	17.77643	6.00	2.45	19.2
4.977	17.74039	3.00	1.73	19.2
4.987	17.70449	1.00	1.00	19.2

4.997	17.66873	3.00	1.73	19.2
5.008	17.63312	4.00	2.00	19.2
5.018	17.59765	2.00	1.41	19.2
5.028	17.56232	2.00	1.41	19.2
5.038	17.52714	7.00	2.65	19.2
5.048	17.49209	3.00	1.73	19.2
5.058	17.45719	2.00	1.41	19.2
5.068	17.42243	4.00	2.00	19.2
5.078	17.38780	5.00	2.24	19.2
5.088	17.35331	2.00	1.41	19.2
5.098	17.31896	4.00	2.00	19.2
5.109	17.28474	3.00	1.73	19.2
5.119	17.25066	5.00	2.24	19.2
5.129	17.21672	2.00	1.41	19.2
5.139	17.18290	3.00	1.73	19.2
5.149	17.14922	3.00	1.73	19.2
5.159	17.11567	5.00	2.24	19.2
5.169	17.08226	6.00	2.45	19.2
5.179	17.04897	10.0	3.16	19.2
5.189	17.01581	7.00	2.65	19.2
5.199	16.98278	2.00	1.41	19.2
5.209	16.94988	4.00	2.00	19.2
5.220	16.91711	5.00	2.24	19.2
5.230	16.88446	1.00	1.00	19.2
5.240	16.85194	4.00	2.00	19.2
5.250	16.81955	8.00	2.83	19.2
5.260	16.78728	6.00	2.45	19.2
5.270	16.75513	9.00	3.00	19.2
5.280	16.72310	3.00	1.73	19.2
5.290	16.69120	4.00	2.00	19.2
5.300	16.65942	2.00	1.41	19.2
5.310	16.62776	5.00	2.24	19.2
5.321	16.59622	10.0	3.16	19.2
5.331	16.56480	3.00	1.73	19.2
5.341	16.53350	3.00	1.73	19.2
5.351	16.50232	8.00	2.83	19.2
5.361	16.47125	4.00	2.00	19.2
5.371	16.44031	5.00	2.24	19.2
5.381	16.40947	4.00	2.00	19.2
5.391	16.37876	4.00	2.00	19.2
5.401	16.34816	5.00	2.24	19.2
5.411	16.31767	5.00	2.24	19.2
5.422	16.28729	10.0	3.16	19.2
5.432	16.25703	11.0	3.32	19.2
5.442	16.22689	7.00	2.65	19.2
5.452	16.19685	4.00	2.00	19.2
5.462	16.16692	5.00	2.24	19.2
5.472	16.13711	5.00	2.24	19.2
5.482	16.10740	6.00	2.45	19.2
5.492	16.07781	2.00	1.41	19.2

5.502	16.04832	8.00	2.83	19.2
5.512	16.01894	1.00	1.00	19.2
5.523	15.98967	6.00	2.45	19.2
5.533	15.96051	10.0	3.16	19.2
5.543	15.93145	6.00	2.45	19.2
5.553	15.90249	8.00	2.83	19.2
5.563	15.87365	13.0	3.61	19.2
5.573	15.84490	7.00	2.65	19.2
5.583	15.81627	16.0	4.00	19.2
5.593	15.78773	13.0	3.61	19.2
5.603	15.75930	10.0	3.16	19.2
5.613	15.73097	5.00	2.24	19.2
5.624	15.70274	7.00	2.65	19.2
5.634	15.67461	13.0	3.61	19.2
5.644	15.64659	4.00	2.00	19.2
5.654	15.61866	5.00	2.24	19.2
5.664	15.59083	7.00	2.65	19.2
5.674	15.56311	4.00	2.00	19.2
5.684	15.53548	11.0	3.32	19.2
5.694	15.50795	4.00	2.00	19.2
5.704	15.48051	10.0	3.16	19.2
5.714	15.45318	4.00	2.00	19.2
5.725	15.42594	3.00	1.73	19.2
5.735	15.39879	10.0	3.16	19.2
5.745	15.37174	14.0	3.74	19.2
5.755	15.34479	8.00	2.83	19.2
5.765	15.31793	5.00	2.24	19.2
5.775	15.29117	13.0	3.61	19.2
5.785	15.26450	9.00	3.00	19.2
5.795	15.23792	16.0	4.00	19.2
5.805	15.21143	15.0	3.87	19.2
5.815	15.18504	9.00	3.00	19.2
5.826	15.15874	16.0	4.00	19.2
5.836	15.13252	14.0	3.74	19.2
5.846	15.10640	12.0	3.46	19.2
5.856	15.08037	14.0	3.74	19.2
5.866	15.05443	8.00	2.83	19.2
5.876	15.02858	16.0	4.00	19.2
5.886	15.00282	7.00	2.65	19.2
5.896	14.97714	13.0	3.61	19.2
5.906	14.95156	8.00	2.83	19.2
5.916	14.92606	11.0	3.32	19.2
5.927	14.90064	22.0	4.69	19.2
5.937	14.87532	12.0	3.46	19.2
5.947	14.85008	23.0	4.80	19.2
5.957	14.82492	20.0	4.47	19.2
5.967	14.79986	19.0	4.36	19.2
5.977	14.77487	10.0	3.16	19.2
5.987	14.74997	15.0	3.87	19.2
5.997	14.72516	13.0	3.61	19.2

6.007	14.70042	10.0	3.16	19.2
6.017	14.67577	12.0	3.46	19.2
6.028	14.65121	20.0	4.47	19.2
6.038	14.62672	20.0	4.47	19.2
6.048	14.60232	7.00	2.65	19.2
6.058	14.57800	21.0	4.58	19.2
6.068	14.55376	21.0	4.58	19.2
6.078	14.52960	14.0	3.74	19.2
6.088	14.50552	23.0	4.80	19.2
6.098	14.48152	17.0	4.12	19.2
6.108	14.45760	18.0	4.24	19.2
6.118	14.43376	22.0	4.69	19.2
6.129	14.40999	19.0	4.36	19.2
6.139	14.38631	25.0	5.00	19.2
6.149	14.36270	23.0	4.80	19.2
6.159	14.33917	19.0	4.36	19.2
6.169	14.31572	15.0	3.87	19.2
6.179	14.29234	16.0	4.00	19.2
6.189	14.26905	24.0	4.90	19.2
6.199	14.24582	19.0	4.36	19.2
6.209	14.22267	9.00	3.00	19.2
6.219	14.19960	25.0	5.00	19.2
6.230	14.17660	8.00	2.83	19.2
6.240	14.15368	30.0	5.48	19.2
6.250	14.13083	15.0	3.87	19.2
6.260	14.10806	21.0	4.58	19.2
6.270	14.08535	17.0	4.12	19.2
6.280	14.06273	20.0	4.47	19.2
6.290	14.04017	23.0	4.80	19.2
6.300	14.01769	24.0	4.90	19.2
6.310	13.99527	32.0	5.66	19.2
6.320	13.97293	20.0	4.47	19.2
6.331	13.95067	23.0	4.80	19.2
6.341	13.92847	25.0	5.00	19.2
6.351	13.90634	19.0	4.36	19.2
6.361	13.88428	19.0	4.36	19.2
6.371	13.86230	33.0	5.74	19.2
6.381	13.84038	18.0	4.24	19.2
6.391	13.81853	22.0	4.69	19.2
6.401	13.79675	19.0	4.36	19.2
6.411	13.77504	17.0	4.12	19.2
6.421	13.75340	10.0	3.16	19.2
6.431	13.73183	18.0	4.24	19.2
6.442	13.71032	22.0	4.69	19.2
6.452	13.68888	30.0	5.48	19.2
6.462	13.66751	19.0	4.36	19.2
6.472	13.64620	25.0	5.00	19.2
6.482	13.62497	39.0	6.24	19.2
6.492	13.60379	24.0	4.90	19.2
6.502	13.58269	19.0	4.36	19.2

6.512	13.56164	27.0	5.20	19.2
6.522	13.54067	32.0	5.66	19.2
6.532	13.51976	20.0	4.47	19.2
6.543	13.49891	28.0	5.29	19.2
6.553	13.47813	14.0	3.74	19.2
6.563	13.45741	20.0	4.47	19.2
6.573	13.43676	16.0	4.00	19.2
6.583	13.41616	32.0	5.66	19.2
6.593	13.39564	22.0	4.69	19.2
6.603	13.37517	17.0	4.12	19.2
6.613	13.35477	24.0	4.90	19.2
6.623	13.33443	20.0	4.47	19.2
6.633	13.31415	8.00	2.83	19.2
6.644	13.29393	31.0	5.57	19.2
6.654	13.27378	28.0	5.29	19.2
6.664	13.25368	22.0	4.69	19.2
6.674	13.23365	23.0	4.80	19.2
6.684	13.21368	27.0	5.20	19.2
6.694	13.19376	23.0	4.80	19.2
6.704	13.17391	26.0	5.10	19.2
6.714	13.15412	17.0	4.12	19.2
6.724	13.13438	27.0	5.20	19.2
6.734	13.11471	27.0	5.20	19.2
6.745	13.09510	16.0	4.00	19.2
6.755	13.07554	35.0	5.92	19.2
6.765	13.05604	23.0	4.80	19.2
6.775	13.03660	29.0	5.39	19.2
6.785	13.01722	17.0	4.12	19.2
6.795	12.99789	37.0	6.08	19.2
6.805	12.97863	26.0	5.10	19.2
6.815	12.95942	20.0	4.47	19.2
6.825	12.94026	28.0	5.29	19.2
6.835	12.92117	34.0	5.83	19.2
6.846	12.90213	31.0	5.57	19.2
6.856	12.88314	20.0	4.47	19.2
6.866	12.86422	22.0	4.69	19.2
6.876	12.84534	26.0	5.10	19.2
6.886	12.82653	18.0	4.24	19.2
6.896	12.80777	27.0	5.20	19.2
6.906	12.78906	19.0	4.36	19.2
6.916	12.77041	33.0	5.74	19.2
6.926	12.75181	33.0	5.74	19.2
6.936	12.73327	17.0	4.12	19.2
6.947	12.71478	25.0	5.00	19.2
6.957	12.69634	27.0	5.20	19.2
6.967	12.67796	32.0	5.66	19.2
6.977	12.65963	23.0	4.80	19.2
6.987	12.64135	31.0	5.57	19.2
6.997	12.62313	25.0	5.00	19.2
7.007	12.60496	46.0	6.78	19.2

7.017	12.58684	17.0	4.12	19.2
7.027	12.56877	32.0	5.66	19.2
7.037	12.55076	33.0	5.74	19.2
7.048	12.53280	29.0	5.39	19.2
7.058	12.51489	27.0	5.20	19.2
7.068	12.49703	40.0	6.32	19.2
7.078	12.47922	17.0	4.12	19.2
7.088	12.46146	26.0	5.10	19.2
7.098	12.44375	22.0	4.69	19.2
7.108	12.42609	22.0	4.69	19.2
7.118	12.40849	19.0	4.36	19.2
7.128	12.39093	32.0	5.66	19.2
7.138	12.37342	34.0	5.83	19.2
7.149	12.35596	30.0	5.48	19.2
7.159	12.33855	31.0	5.57	19.2
7.169	12.32119	33.0	5.74	19.2
7.179	12.30388	35.0	5.92	19.2
7.189	12.28662	28.0	5.29	19.2
7.199	12.26941	27.0	5.20	19.2
7.209	12.25224	37.0	6.08	19.2
7.219	12.23512	25.0	5.00	19.2
7.229	12.21805	23.0	4.80	19.2
7.239	12.20103	33.0	5.74	19.2
7.250	12.18406	37.0	6.08	19.2
7.260	12.16713	28.0	5.29	19.2
7.270	12.15025	36.0	6.00	19.2
7.280	12.13342	27.0	5.20	19.2
7.290	12.11663	41.0	6.40	19.2
7.300	12.09989	28.0	5.29	19.2
7.310	12.08320	30.0	5.48	19.2
7.320	12.06655	36.0	6.00	19.2
7.330	12.04995	35.0	5.92	19.2
7.340	12.03339	25.0	5.00	19.2
7.351	12.01688	33.0	5.74	19.2
7.361	12.00042	35.0	5.92	19.2
7.371	11.98400	32.0	5.66	19.2
7.381	11.96762	30.0	5.48	19.2
7.391	11.95129	39.0	6.24	19.2
7.401	11.93500	37.0	6.08	19.2
7.411	11.91876	34.0	5.83	19.2
7.421	11.90257	43.0	6.56	19.2
7.431	11.88641	32.0	5.66	19.2
7.441	11.87030	38.0	6.16	19.2
7.452	11.85424	35.0	5.92	19.2
7.462	11.83822	22.0	4.69	19.2
7.472	11.82224	45.0	6.71	19.2
7.482	11.80630	32.0	5.66	19.2
7.492	11.79041	43.0	6.56	19.2
7.502	11.77456	48.0	6.93	19.2
7.512	11.75875	36.0	6.00	19.2

7.522	11.74299	29.0	5.39	19.2
7.532	11.72727	33.0	5.74	19.2
7.542	11.71159	34.0	5.83	19.2
7.553	11.69595	26.0	5.10	19.2
7.563	11.68035	28.0	5.29	19.2
7.573	11.66480	25.0	5.00	19.2
7.583	11.64928	28.0	5.29	19.2
7.593	11.63381	45.0	6.71	19.2
7.603	11.61838	37.0	6.08	19.2
7.613	11.60299	36.0	6.00	19.2
7.623	11.58764	45.0	6.71	19.2
7.633	11.57233	34.0	5.83	19.2
7.643	11.55707	29.0	5.39	19.2
7.654	11.54184	21.0	4.58	19.2
7.664	11.52665	38.0	6.16	19.2
7.674	11.51150	51.0	7.14	19.2
7.684	11.49640	33.0	5.74	19.2
7.694	11.48133	43.0	6.56	19.2
7.704	11.46630	41.0	6.40	19.2
7.714	11.45131	32.0	5.66	19.2
7.724	11.43636	39.0	6.24	19.2
7.734	11.42145	46.0	6.78	19.2
7.744	11.40658	38.0	6.16	19.2
7.754	11.39175	40.0	6.32	19.2
7.765	11.37695	51.0	7.14	19.2
7.775	11.36220	61.0	7.81	19.2
7.785	11.34748	44.0	6.63	19.2
7.795	11.33280	28.0	5.29	19.2
7.805	11.31816	40.0	6.32	19.2
7.815	11.30355	44.0	6.63	19.2
7.825	11.28899	42.0	6.48	19.2
7.835	11.27446	47.0	6.86	19.2
7.845	11.25997	48.0	6.93	19.2
7.855	11.24552	56.0	7.48	19.2
7.866	11.23110	45.0	6.71	19.2
7.876	11.21672	45.0	6.71	19.2
7.886	11.20238	54.0	7.35	19.2
7.896	11.18807	40.0	6.32	19.2
7.906	11.17380	53.0	7.28	19.2
7.916	11.15957	41.0	6.40	19.2
7.926	11.14537	51.0	7.14	19.2
7.936	11.13121	48.0	6.93	19.2
7.946	11.11709	41.0	6.40	19.2
7.956	11.10300	50.0	7.07	19.2
7.967	11.08895	53.0	7.28	19.2
7.977	11.07493	57.0	7.55	19.2
7.987	11.06095	57.0	7.55	19.2
7.997	11.04700	48.0	6.93	19.2
8.007	11.03309	56.0	7.48	19.2
8.017	11.01922	50.0	7.07	19.2

8.027	11.00538	37.0	6.08	19.2
8.037	10.99157	38.0	6.16	19.2
8.047	10.97780	59.0	7.68	19.2
8.057	10.96406	48.0	6.93	19.2
8.068	10.95036	61.0	7.81	19.2
8.078	10.93669	53.0	7.28	19.2
8.088	10.92306	51.0	7.14	19.2
8.098	10.90946	43.0	6.56	19.2
8.108	10.89589	50.0	7.07	19.2
8.118	10.88236	49.0	7.00	19.2
8.128	10.86886	51.0	7.14	19.2
8.138	10.85539	52.0	7.21	19.2
8.148	10.84196	64.0	8.00	19.2
8.158	10.82856	59.0	7.68	19.2
8.169	10.81520	51.0	7.14	19.2
8.179	10.80187	54.0	7.35	19.2
8.189	10.78857	59.0	7.68	19.2
8.199	10.77530	48.0	6.93	19.2
8.209	10.76207	58.0	7.62	19.2
8.219	10.74887	49.0	7.00	19.2
8.229	10.73570	47.0	6.86	19.2
8.239	10.72256	50.0	7.07	19.2
8.249	10.70946	58.0	7.62	19.2
8.259	10.69638	52.0	7.21	19.2
8.270	10.68334	52.0	7.21	19.2
8.280	10.67033	66.0	8.12	19.2
8.290	10.65736	58.0	7.62	19.2
8.300	10.64441	58.0	7.62	19.2
8.310	10.63150	57.0	7.55	19.2
8.320	10.61862	48.0	6.93	19.2
8.330	10.60577	67.0	8.19	19.2
8.340	10.59295	58.0	7.62	19.2
8.350	10.58016	49.0	7.00	19.2
8.360	10.56740	49.0	7.00	19.2
8.371	10.55467	58.0	7.62	19.2
8.381	10.54197	64.0	8.00	19.2
8.391	10.52931	67.0	8.19	19.2
8.401	10.51667	67.0	8.19	19.2
8.411	10.50407	52.0	7.21	19.2
8.421	10.49149	67.0	8.19	19.2
8.431	10.47895	60.0	7.75	19.2
8.441	10.46643	63.0	7.94	19.2
8.451	10.45395	64.0	8.00	19.2
8.461	10.44150	47.0	6.86	19.2
8.472	10.42907	65.0	8.06	19.2
8.482	10.41668	65.0	8.06	19.2
8.492	10.40431	75.0	8.66	19.2
8.502	10.39197	68.0	8.25	19.2
8.512	10.37967	74.0	8.60	19.2
8.522	10.36739	66.0	8.12	19.2

8.532	10.35514	62.0	7.87	19.2
8.542	10.34292	72.0	8.49	19.2
8.552	10.33073	73.0	8.54	19.2
8.562	10.31857	55.0	7.42	19.2
8.573	10.30643	64.0	8.00	19.2
8.583	10.29433	63.0	7.94	19.2
8.593	10.28225	47.0	6.86	19.2
8.603	10.27020	68.0	8.25	19.2
8.613	10.25818	63.0	7.94	19.2
8.623	10.24619	68.0	8.25	19.2
8.633	10.23423	76.0	8.72	19.2
8.643	10.22229	80.0	8.94	19.2
8.653	10.21038	68.0	8.25	19.2
8.663	10.19850	51.0	7.14	19.2
8.674	10.18665	61.0	7.81	19.2
8.684	10.17483	84.0	9.17	19.2
8.694	10.16303	77.0	8.77	19.2
8.704	10.15126	64.0	8.00	19.2
8.714	10.13952	67.0	8.19	19.2
8.724	10.12780	59.0	7.68	19.2
8.734	10.11611	67.0	8.19	19.2
8.744	10.10445	57.0	7.55	19.2
8.754	10.09282	62.0	7.87	19.2
8.764	10.08121	76.0	8.72	19.2
8.775	10.06963	65.0	8.06	19.2
8.785	10.05808	67.0	8.19	19.2
8.795	10.04655	69.0	8.31	19.2
8.805	10.03505	97.0	9.85	19.2
8.815	10.02357	83.0	9.11	19.2
8.825	10.01213	75.0	8.66	19.2
8.835	10.00070	84.0	9.17	19.2
8.845	9.98931	94.0	9.70	19.2
8.855	9.97794	84.0	9.17	19.2
8.865	9.96660	65.0	8.06	19.2
8.876	9.95528	94.0	9.70	19.2
8.886	9.94398	79.0	8.89	19.2
8.896	9.93272	73.0	8.54	19.2
8.906	9.92148	83.0	9.11	19.2
8.916	9.91026	71.0	8.43	19.2
8.926	9.89907	70.0	8.37	19.2
8.936	9.88791	83.0	9.11	19.2
8.946	9.87677	77.0	8.77	19.2
8.956	9.86565	78.0	8.83	19.2
8.966	9.85456	80.0	8.94	19.2
8.977	9.84350	65.0	8.06	19.2
8.987	9.83246	89.0	9.43	19.2
8.997	9.82144	60.0	7.75	19.2
9.007	9.81045	56.0	7.48	19.2
9.017	9.79949	72.0	8.49	19.2
9.027	9.78855	78.0	8.83	19.2

9.037	9.77763	98.0	9.90	19.2
9.047	9.76674	71.0	8.43	19.2
9.057	9.75587	88.0	9.38	19.2
9.067	9.74503	82.0	9.06	19.2
9.077	9.73421	66.0	8.12	19.2
9.088	9.72341	70.0	8.37	19.2
9.098	9.71264	88.0	9.38	19.2
9.108	9.70190	85.0	9.22	19.2
9.118	9.69117	83.0	9.11	19.2
9.128	9.68047	87.0	9.33	19.2
9.138	9.66980	78.0	8.83	19.2
9.148	9.65914	71.0	8.43	19.2
9.158	9.64851	89.0	9.43	19.2
9.168	9.63791	93.0	9.64	19.2
9.178	9.62733	78.0	8.83	19.2
9.189	9.61677	95.0	9.75	19.2
9.199	9.60623	85.0	9.22	19.2
9.209	9.59572	81.0	9.00	19.2
9.219	9.58523	82.0	9.06	19.2
9.229	9.57476	90.0	9.49	19.2
9.239	9.56432	94.0	9.70	19.2
9.249	9.55390	91.0	9.54	19.2
9.259	9.54350	83.0	9.11	19.2
9.269	9.53313	88.0	9.38	19.2
9.279	9.52277	81.0	9.00	19.2
9.290	9.51244	78.0	8.83	19.2
9.300	9.50214	93.0	9.64	19.2
9.310	9.49185	82.0	9.06	19.2
9.320	9.48159	85.0	9.22	19.2
9.330	9.47135	81.0	9.00	19.2
9.340	9.46113	100	10.0	19.2
9.350	9.45093	78.0	8.83	19.2
9.360	9.44076	97.0	9.85	19.2
9.370	9.43061	93.0	9.64	19.2
9.380	9.42048	92.0	9.59	19.2
9.391	9.41037	93.0	9.64	19.2
9.401	9.40028	87.0	9.33	19.2
9.411	9.39021	93.0	9.64	19.2
9.421	9.38017	82.0	9.06	19.2
9.431	9.37015	102	10.1	19.2
9.441	9.36015	92.0	9.59	19.2
9.451	9.35017	104	10.2	19.2
9.461	9.34021	101	10.0	19.2
9.471	9.33027	101	10.0	19.2
9.481	9.32036	83.0	9.11	19.2
9.492	9.31046	94.0	9.70	19.2
9.502	9.30059	76.0	8.72	19.2
9.512	9.29074	108	10.4	19.2
9.522	9.28091	92.0	9.59	19.2
9.532	9.27110	92.0	9.59	19.2

9.542	9.26131	106	10.3	19.2
9.552	9.25154	83.0	9.11	19.2
9.562	9.24179	101	10.0	19.2
9.572	9.23206	107	10.3	19.2
9.582	9.22235	102	10.1	19.2
9.593	9.21267	83.0	9.11	19.2
9.603	9.20300	95.0	9.75	19.2
9.613	9.19335	90.0	9.49	19.2
9.623	9.18373	93.0	9.64	19.2
9.633	9.17412	110	10.5	19.2
9.643	9.16454	72.0	8.49	19.2
9.653	9.15497	81.0	9.00	19.2
9.663	9.14543	101	10.0	19.2
9.673	9.13590	94.0	9.70	19.2
9.683	9.12640	88.0	9.38	19.2
9.694	9.11691	94.0	9.70	19.2
9.704	9.10744	89.0	9.43	19.2
9.714	9.09800	94.0	9.70	19.2
9.724	9.08857	73.0	8.54	19.2
9.734	9.07916	98.0	9.90	19.2
9.744	9.06978	104	10.2	19.2
9.754	9.06041	91.0	9.54	19.2
9.764	9.05106	97.0	9.85	19.2
9.774	9.04173	97.0	9.85	19.2
9.784	9.03242	112	10.6	19.2
9.795	9.02313	103	10.1	19.2
9.805	9.01386	103	10.1	19.2
9.815	9.00461	97.0	9.85	19.2
9.825	8.99537	103	10.1	19.2
9.835	8.98616	96.0	9.80	19.2
9.845	8.97696	93.0	9.64	19.2
9.855	8.96779	81.0	9.00	19.2
9.865	8.95863	89.0	9.43	19.2
9.875	8.94949	110	10.5	19.2
9.885	8.94037	97.0	9.85	19.2
9.896	8.93127	96.0	9.80	19.2
9.906	8.92218	89.0	9.43	19.2
9.916	8.91312	126	11.2	19.2
9.926	8.90407	94.0	9.70	19.2
9.936	8.89505	94.0	9.70	19.2
9.946	8.88604	93.0	9.64	19.2
9.956	8.87705	82.0	9.06	19.2
9.966	8.86807	95.0	9.75	19.2
9.976	8.85912	98.0	9.90	19.2
9.986	8.85018	126	11.2	19.2
9.997	8.84126	105	10.2	19.2
10.007	8.83236	108	10.4	19.2
10.017	8.82348	107	10.3	19.2
10.027	8.81462	98.0	9.90	19.2
10.037	8.80577	97.0	9.85	19.2

10.047	8.79694	103	10.1	19.2
10.057	8.78813	102	10.1	19.2
10.067	8.77934	106	10.3	19.2
10.077	8.77056	98.0	9.90	19.2
10.087	8.76180	104	10.2	19.2
10.098	8.75306	108	10.4	19.2
10.108	8.74434	99.0	9.95	19.2
10.118	8.73563	99.0	9.95	19.2
10.128	8.72694	96.0	9.80	19.2
10.138	8.71827	93.0	9.64	19.2
10.148	8.70962	97.0	9.85	19.2
10.158	8.70098	93.0	9.64	19.2
10.168	8.69236	99.0	9.95	19.2
10.178	8.68376	97.0	9.85	19.2
10.188	8.67518	98.0	9.90	19.2
10.199	8.66661	121	11.0	19.2
10.209	8.65806	102	10.1	19.2
10.219	8.64952	92.0	9.59	19.2
10.229	8.64101	96.0	9.80	19.2
10.239	8.63251	108	10.4	19.2
10.249	8.62402	101	10.0	19.2
10.259	8.61555	118	10.9	19.2
10.269	8.60710	98.0	9.90	19.2
10.279	8.59867	111	10.5	19.2
10.289	8.59025	99.0	9.95	19.2
10.300	8.58185	112	10.6	19.2
10.310	8.57347	93.0	9.64	19.2
10.320	8.56510	114	10.7	19.2
10.330	8.55675	106	10.3	19.2
10.340	8.54841	91.0	9.54	19.2
10.350	8.54010	105	10.2	19.2
10.360	8.53179	104	10.2	19.2
10.370	8.52351	98.0	9.90	19.2
10.380	8.51524	105	10.2	19.2
10.390	8.50698	101	10.0	19.2
10.400	8.49875	94.0	9.70	19.2
10.411	8.49052	116	10.8	19.2
10.421	8.48232	116	10.8	19.2
10.431	8.47413	118	10.9	19.2
10.441	8.46595	111	10.5	19.2
10.451	8.45780	113	10.6	19.2
10.461	8.44965	107	10.3	19.2
10.471	8.44153	108	10.4	19.2
10.481	8.43341	99.0	9.95	19.2
10.491	8.42532	104	10.2	19.2
10.501	8.41724	102	10.1	19.2
10.512	8.40918	105	10.2	19.2
10.522	8.40113	123	11.1	19.2
10.532	8.39309	92.0	9.59	19.2
10.542	8.38507	110	10.5	19.2

10.552	8.37707	124	11.1	19.2
10.562	8.36908	115	10.7	19.2
10.572	8.36111	117	10.8	19.2
10.582	8.35316	107	10.3	19.2
10.592	8.34521	114	10.7	19.2
10.602	8.33729	130	11.4	19.2
10.613	8.32938	99.0	9.95	19.2
10.623	8.32148	116	10.8	19.2
10.633	8.31360	87.0	9.33	19.2
10.643	8.30573	112	10.6	19.2
10.653	8.29788	108	10.4	19.2
10.663	8.29005	128	11.3	19.2
10.673	8.28222	114	10.7	19.2
10.683	8.27442	106	10.3	19.2
10.693	8.26662	127	11.3	19.2
10.703	8.25885	125	11.2	19.2
10.714	8.25108	120	11.0	19.2
10.724	8.24334	116	10.8	19.2
10.734	8.23560	106	10.3	19.2
10.744	8.22788	124	11.1	19.2
10.754	8.22018	122	11.0	19.2
10.764	8.21249	124	11.1	19.2
10.774	8.20482	130	11.4	19.2
10.784	8.19715	103	10.1	19.2
10.794	8.18951	111	10.5	19.2
10.804	8.18188	99.0	9.95	19.2
10.815	8.17426	118	10.9	19.2
10.825	8.16665	141	11.9	19.2
10.835	8.15906	113	10.6	19.2
10.845	8.15149	128	11.3	19.2
10.855	8.14393	121	11.0	19.2
10.865	8.13638	118	10.9	19.2
10.875	8.12885	118	10.9	19.2
10.885	8.12133	113	10.6	19.2
10.895	8.11382	116	10.8	19.2
10.905	8.10633	111	10.5	19.2
10.916	8.09885	124	11.1	19.2
10.926	8.09139	85.0	9.22	19.2
10.936	8.08394	123	11.1	19.2
10.946	8.07650	119	10.9	19.2
10.956	8.06908	110	10.5	19.2
10.966	8.06167	118	10.9	19.2
10.976	8.05428	124	11.1	19.2
10.986	8.04690	97.0	9.85	19.2
10.996	8.03953	99.0	9.95	19.2
11.006	8.03218	110	10.5	19.2
11.017	8.02483	110	10.5	19.2
11.027	8.01751	90.0	9.49	19.2
11.037	8.01019	131	11.4	19.2
11.047	8.00289	127	11.3	19.2

11.057	7.99561	112	10.6	19.2
11.067	7.98833	108	10.4	19.2
11.077	7.98107	103	10.1	19.2
11.087	7.97382	89.0	9.43	19.2
11.097	7.96659	116	10.8	19.2
11.107	7.95937	128	11.3	19.2
11.118	7.95216	96.0	9.80	19.2
11.128	7.94497	122	11.0	19.2
11.138	7.93779	142	11.9	19.2
11.148	7.93062	114	10.7	19.2
11.158	7.92346	110	10.5	19.2
11.168	7.91632	117	10.8	19.2
11.178	7.90919	126	11.2	19.2
11.188	7.90207	123	11.1	19.2
11.198	7.89497	100	10.0	19.2
11.208	7.88788	138	11.7	19.2
11.219	7.88080	99.0	9.95	19.2
11.229	7.87374	142	11.9	19.2
11.239	7.86668	99.0	9.95	19.2
11.249	7.85964	109	10.4	19.2
11.259	7.85261	128	11.3	19.2
11.269	7.84560	105	10.2	19.2
11.279	7.83860	133	11.5	19.2
11.289	7.83161	115	10.7	19.2
11.299	7.82463	109	10.4	19.2
11.309	7.81767	122	11.0	19.2
11.320	7.81071	141	11.9	19.2
11.330	7.80377	117	10.8	19.2
11.340	7.79685	125	11.2	19.2
11.350	7.78993	128	11.3	19.2
11.360	7.78303	124	11.1	19.2
11.370	7.77614	116	10.8	19.2
11.380	7.76926	127	11.3	19.2
11.390	7.76239	116	10.8	19.2
11.400	7.75554	119	10.9	19.2
11.410	7.74870	122	11.0	19.2
11.421	7.74187	135	11.6	19.2
11.431	7.73505	117	10.8	19.2
11.441	7.72825	101	10.0	19.2
11.451	7.72145	123	11.1	19.2
11.461	7.71467	125	11.2	19.2
11.471	7.70790	113	10.6	19.2
11.481	7.70114	102	10.1	19.2
11.491	7.69440	116	10.8	19.2
11.501	7.68767	138	11.7	19.2
11.511	7.68094	91.0	9.54	19.2
11.522	7.67423	108	10.4	19.2
11.532	7.66754	113	10.6	19.2
11.542	7.66085	124	11.1	19.2
11.552	7.65417	100	10.0	19.2

11.562	7.64751	136	11.7	19.2
11.572	7.64086	137	11.7	19.2
11.582	7.63422	142	11.9	19.2
11.592	7.62759	110	10.5	19.2
11.602	7.62097	122	11.0	19.2
11.612	7.61437	100	10.0	19.2
11.623	7.60778	103	10.1	19.2
11.633	7.60119	128	11.3	19.2
11.643	7.59462	101	10.0	19.2
11.653	7.58806	116	10.8	19.2
11.663	7.58151	132	11.5	19.2
11.673	7.57498	102	10.1	19.2
11.683	7.56845	122	11.0	19.2
11.693	7.56194	114	10.7	19.2
11.703	7.55544	89.0	9.43	19.2
11.713	7.54894	110	10.5	19.2
11.723	7.54246	121	11.0	19.2
11.734	7.53599	119	10.9	19.2
11.744	7.52954	111	10.5	19.2
11.754	7.52309	125	11.2	19.2
11.764	7.51665	114	10.7	19.2
11.774	7.51023	116	10.8	19.2
11.784	7.50382	129	11.4	19.2
11.794	7.49741	112	10.6	19.2
11.804	7.49102	124	11.1	19.2
11.814	7.48464	117	10.8	19.2
11.824	7.47827	120	11.0	19.2
11.835	7.47191	120	11.0	19.2
11.845	7.46556	129	11.4	19.2
11.855	7.45923	127	11.3	19.2
11.865	7.45290	106	10.3	19.2
11.875	7.44658	120	11.0	19.2
11.885	7.44028	115	10.7	19.2
11.895	7.43398	115	10.7	19.2
11.905	7.42770	124	11.1	19.2
11.915	7.42143	131	11.4	19.2
11.925	7.41517	123	11.1	19.2
11.936	7.40891	131	11.4	19.2
11.946	7.40267	129	11.4	19.2
11.956	7.39644	138	11.7	19.2
11.966	7.39022	121	11.0	19.2
11.976	7.38401	131	11.4	19.2
11.986	7.37781	134	11.6	19.2
11.996	7.37163	100	10.0	19.2
12.006	7.36545	110	10.5	19.2
12.016	7.35928	112	10.6	19.2
12.026	7.35312	128	11.3	19.2
12.037	7.34698	117	10.8	19.2
12.047	7.34084	123	11.1	19.2
12.057	7.33471	120	11.0	19.2

12.067	7.32860	133	11.5	19.2
12.077	7.32249	117	10.8	19.2
12.087	7.31640	111	10.5	19.2
12.097	7.31031	139	11.8	19.2
12.107	7.30423	119	10.9	19.2
12.117	7.29817	132	11.5	19.2
12.127	7.29211	129	11.4	19.2
12.138	7.28607	133	11.5	19.2
12.148	7.28003	123	11.1	19.2
12.158	7.27401	108	10.4	19.2
12.168	7.26800	128	11.3	19.2
12.178	7.26199	129	11.4	19.2
12.188	7.25600	122	11.0	19.2
12.198	7.25001	130	11.4	19.2
12.208	7.24404	119	10.9	19.2
12.218	7.23807	100	10.0	19.2
12.228	7.23212	138	11.7	19.2
12.239	7.22617	99.0	9.95	19.2
12.249	7.22024	117	10.8	19.2
12.259	7.21431	128	11.3	19.2
12.269	7.20839	149	12.2	19.2
12.279	7.20249	129	11.4	19.2
12.289	7.19659	104	10.2	19.2
12.299	7.19071	154	12.4	19.2
12.309	7.18483	126	11.2	19.2
12.319	7.17896	118	10.9	19.2
12.329	7.17310	131	11.4	19.2
12.340	7.16726	109	10.4	19.2
12.350	7.16142	127	11.3	19.2
12.360	7.15559	110	10.5	19.2
12.370	7.14977	148	12.2	19.2
12.380	7.14396	120	11.0	19.2
12.390	7.13816	131	11.4	19.2
12.400	7.13237	128	11.3	19.2
12.410	7.12659	130	11.4	19.2
12.420	7.12081	115	10.7	19.2
12.430	7.11505	152	12.3	19.2
12.441	7.10930	128	11.3	19.2
12.451	7.10355	132	11.5	19.2
12.461	7.09782	136	11.7	19.2
12.471	7.09209	139	11.8	19.2
12.481	7.08638	122	11.0	19.2
12.491	7.08067	125	11.2	19.2
12.501	7.07497	125	11.2	19.2
12.511	7.06928	122	11.0	19.2
12.521	7.06361	132	11.5	19.2
12.531	7.05794	110	10.5	19.2
12.542	7.05227	118	10.9	19.2
12.552	7.04662	126	11.2	19.2
12.562	7.04098	138	11.7	19.2

12.572	7.03535	142	11.9	19.2
12.582	7.02972	135	11.6	19.2
12.592	7.02411	125	11.2	19.2
12.602	7.01850	121	11.0	19.2
12.612	7.01290	129	11.4	19.2
12.622	7.00732	134	11.6	19.2
12.632	7.00174	103	10.1	19.2
12.643	6.99617	130	11.4	19.2
12.653	6.99060	130	11.4	19.2
12.663	6.98505	145	12.0	19.2
12.673	6.97951	147	12.1	19.2
12.683	6.97397	141	11.9	19.2
12.693	6.96845	123	11.1	19.2
12.703	6.96293	131	11.4	19.2
12.713	6.95742	132	11.5	19.2
12.723	6.95192	138	11.7	19.2
12.733	6.94643	115	10.7	19.2
12.744	6.94095	130	11.4	19.2
12.754	6.93547	128	11.3	19.2
12.764	6.93001	127	11.3	19.2
12.774	6.92455	128	11.3	19.2
12.784	6.91910	110	10.5	19.2
12.794	6.91366	125	11.2	19.2
12.804	6.90823	156	12.5	19.2
12.814	6.90281	148	12.2	19.2
12.824	6.89740	169	13.0	19.2
12.834	6.89199	131	11.4	19.2
12.845	6.88660	132	11.5	19.2
12.855	6.88121	148	12.2	19.2
12.865	6.87583	132	11.5	19.2
12.875	6.87046	116	10.8	19.2
12.885	6.86510	116	10.8	19.2
12.895	6.85974	144	12.0	19.2
12.905	6.85440	134	11.6	19.2
12.915	6.84906	155	12.4	19.2
12.925	6.84373	130	11.4	19.2
12.935	6.83841	115	10.7	19.2
12.946	6.83310	140	11.8	19.2
12.956	6.82780	144	12.0	19.2
12.966	6.82250	167	12.9	19.2
12.976	6.81721	144	12.0	19.2
12.986	6.81193	134	11.6	19.2
12.996	6.80666	151	12.3	19.2
13.006	6.80140	144	12.0	19.2
13.016	6.79615	156	12.5	19.2
13.026	6.79090	149	12.2	19.2
13.036	6.78566	137	11.7	19.2
13.046	6.78043	136	11.7	19.2
13.057	6.77521	174	13.2	19.2
13.067	6.77000	163	12.8	19.2

13.077	6.76479	151	12.3	19.2
13.087	6.75959	143	12.0	19.2
13.097	6.75440	132	11.5	19.2
13.107	6.74922	132	11.5	19.2
13.117	6.74405	143	12.0	19.2
13.127	6.73888	164	12.8	19.2
13.137	6.73372	153	12.4	19.2
13.147	6.72857	136	11.7	19.2
13.158	6.72343	141	11.9	19.2
13.168	6.71830	132	11.5	19.2
13.178	6.71317	129	11.4	19.2
13.188	6.70805	162	12.7	19.2
13.198	6.70294	154	12.4	19.2
13.208	6.69784	147	12.1	19.2
13.218	6.69275	118	10.9	19.2
13.228	6.68766	147	12.1	19.2
13.238	6.68258	174	13.2	19.2
13.248	6.67751	126	11.2	19.2
13.259	6.67244	159	12.6	19.2
13.269	6.66739	160	12.6	19.2
13.279	6.66234	150	12.2	19.2
13.289	6.65730	136	11.7	19.2
13.299	6.65227	113	10.6	19.2
13.309	6.64724	154	12.4	19.2
13.319	6.64222	147	12.1	19.2
13.329	6.63721	139	11.8	19.2
13.339	6.63221	141	11.9	19.2
13.349	6.62722	154	12.4	19.2
13.360	6.62223	132	11.5	19.2
13.370	6.61725	129	11.4	19.2
13.380	6.61228	141	11.9	19.2
13.390	6.60731	144	12.0	19.2
13.400	6.60236	139	11.8	19.2
13.410	6.59741	157	12.5	19.2
13.420	6.59246	119	10.9	19.2
13.430	6.58753	128	11.3	19.2
13.440	6.58260	137	11.7	19.2
13.450	6.57768	135	11.6	19.2
13.461	6.57277	155	12.4	19.2
13.471	6.56787	160	12.6	19.2
13.481	6.56297	142	11.9	19.2
13.491	6.55808	162	12.7	19.2
13.501	6.55319	165	12.8	19.2
13.511	6.54832	143	12.0	19.2
13.521	6.54345	128	11.3	19.2
13.531	6.53859	142	11.9	19.2
13.541	6.53374	149	12.2	19.2
13.551	6.52889	147	12.1	19.2
13.562	6.52405	152	12.3	19.2
13.572	6.51922	142	11.9	19.2

13.582	6.51439	165	12.8	19.2
13.592	6.50957	148	12.2	19.2
13.602	6.50476	152	12.3	19.2
13.612	6.49996	145	12.0	19.2
13.622	6.49516	148	12.2	19.2
13.632	6.49038	121	11.0	19.2
13.642	6.48559	138	11.7	19.2
13.652	6.48082	166	12.9	19.2
13.663	6.47605	111	10.5	19.2
13.673	6.47129	163	12.8	19.2
13.683	6.46654	153	12.4	19.2
13.693	6.46179	147	12.1	19.2
13.703	6.45705	163	12.8	19.2
13.713	6.45232	145	12.0	19.2
13.723	6.44759	144	12.0	19.2
13.733	6.44287	147	12.1	19.2
13.743	6.43816	177	13.3	19.2
13.753	6.43346	159	12.6	19.2
13.764	6.42876	167	12.9	19.2
13.774	6.42407	157	12.5	19.2
13.784	6.41938	172	13.1	19.2
13.794	6.41471	146	12.1	19.2
13.804	6.41003	164	12.8	19.2
13.814	6.40537	193	13.9	19.2
13.824	6.40071	162	12.7	19.2
13.834	6.39606	157	12.5	19.2
13.844	6.39142	184	13.6	19.2
13.854	6.38678	141	11.9	19.2
13.865	6.38216	158	12.6	19.2
13.875	6.37753	143	12.0	19.2
13.885	6.37292	143	12.0	19.2
13.895	6.36831	166	12.9	19.2
13.905	6.36370	142	11.9	19.2
13.915	6.35911	156	12.5	19.2
13.925	6.35452	176	13.3	19.2
13.935	6.34994	165	12.8	19.2
13.945	6.34536	142	11.9	19.2
13.955	6.34079	170	13.0	19.2
13.966	6.33623	180	13.4	19.2
13.976	6.33167	143	12.0	19.2
13.986	6.32712	152	12.3	19.2
13.996	6.32258	173	13.2	19.2
14.006	6.31804	160	12.6	19.2
14.016	6.31351	152	12.3	19.2
14.026	6.30899	171	13.1	19.2
14.036	6.30447	149	12.2	19.2
14.046	6.29996	155	12.4	19.2
14.056	6.29546	168	13.0	19.2
14.067	6.29096	166	12.9	19.2
14.077	6.28647	153	12.4	19.2

14.087	6.28199	154	12.4	19.2
14.097	6.27751	174	13.2	19.2
14.107	6.27304	146	12.1	19.2
14.117	6.26857	196	14.0	19.2
14.127	6.26412	159	12.6	19.2
14.137	6.25966	149	12.2	19.2
14.147	6.25522	168	13.0	19.2
14.157	6.25078	150	12.2	19.2
14.168	6.24634	187	13.7	19.2
14.178	6.24192	153	12.4	19.2
14.188	6.23750	169	13.0	19.2
14.198	6.23308	174	13.2	19.2
14.208	6.22868	168	13.0	19.2
14.218	6.22427	154	12.4	19.2
14.228	6.21988	164	12.8	19.2
14.238	6.21549	164	12.8	19.2
14.248	6.21111	155	12.4	19.2
14.258	6.20673	188	13.7	19.2
14.269	6.20236	163	12.8	19.2
14.279	6.19800	199	14.1	19.2
14.289	6.19364	155	12.4	19.2
14.299	6.18929	173	13.2	19.2
14.309	6.18494	181	13.5	19.2
14.319	6.18060	160	12.6	19.2
14.329	6.17627	162	12.7	19.2
14.339	6.17194	148	12.2	19.2
14.349	6.16762	143	12.0	19.2
14.359	6.16330	170	13.0	19.2
14.369	6.15899	185	13.6	19.2
14.380	6.15469	170	13.0	19.2
14.390	6.15039	178	13.3	19.2
14.400	6.14610	154	12.4	19.2
14.410	6.14182	171	13.1	19.2
14.420	6.13754	173	13.2	19.2
14.430	6.13327	178	13.3	19.2
14.440	6.12900	169	13.0	19.2
14.450	6.12474	167	12.9	19.2
14.460	6.12048	154	12.4	19.2
14.470	6.11624	184	13.6	19.2
14.481	6.11199	156	12.5	19.2
14.491	6.10776	195	14.0	19.2
14.501	6.10352	158	12.6	19.2
14.511	6.09930	181	13.5	19.2
14.521	6.09508	194	13.9	19.2
14.531	6.09087	181	13.5	19.2
14.541	6.08666	196	14.0	19.2
14.551	6.08246	192	13.9	19.2
14.561	6.07826	202	14.2	19.2
14.571	6.07407	177	13.3	19.2
14.582	6.06989	186	13.6	19.2

14.592	6.06571	196	14.0	19.2
14.602	6.06154	171	13.1	19.2
14.612	6.05737	186	13.6	19.2
14.622	6.05321	188	13.7	19.2
14.632	6.04905	187	13.7	19.2
14.642	6.04490	178	13.3	19.2
14.652	6.04076	188	13.7	19.2
14.662	6.03662	169	13.0	19.2
14.672	6.03249	191	13.8	19.2
14.683	6.02836	169	13.0	19.2
14.693	6.02424	170	13.0	19.2
14.703	6.02013	184	13.6	19.2
14.713	6.01602	185	13.6	19.2
14.723	6.01191	177	13.3	19.2
14.733	6.00781	177	13.3	19.2
14.743	6.00372	187	13.7	19.2
14.753	5.99963	167	12.9	19.2
14.763	5.99555	181	13.5	19.2
14.773	5.99148	182	13.5	19.2
14.784	5.98741	175	13.2	19.2
14.794	5.98334	174	13.2	19.2
14.804	5.97928	171	13.1	19.2
14.814	5.97523	184	13.6	19.2
14.824	5.97118	197	14.0	19.2
14.834	5.96714	207	14.4	19.2
14.844	5.96310	175	13.2	19.2
14.854	5.95907	178	13.3	19.2
14.864	5.95504	153	12.4	19.2
14.874	5.95102	186	13.6	19.2
14.885	5.94701	188	13.7	19.2
14.895	5.94300	178	13.3	19.2
14.905	5.93899	184	13.6	19.2
14.915	5.93500	179	13.4	19.2
14.925	5.93100	194	13.9	19.2
14.935	5.92701	203	14.2	19.2
14.945	5.92303	162	12.7	19.2
14.955	5.91906	175	13.2	19.2
14.965	5.91508	191	13.8	19.2
14.975	5.91112	213	14.6	19.2
14.986	5.90716	200	14.1	19.2
14.996	5.90320	183	13.5	19.2
15.006	5.89925	191	13.8	19.2
15.016	5.89531	175	13.2	19.2
15.026	5.89137	176	13.3	19.2
15.036	5.88743	194	13.9	19.2
15.046	5.88350	197	14.0	19.2
15.056	5.87958	189	13.7	19.2
15.066	5.87566	179	13.4	19.2
15.076	5.87175	200	14.1	19.2
15.087	5.86784	199	14.1	19.2

15.097	5.86394	177	13.3	19.2
15.107	5.86004	196	14.0	19.2
15.117	5.85615	204	14.3	19.2
15.127	5.85226	205	14.3	19.2
15.137	5.84838	196	14.0	19.2
15.147	5.84450	174	13.2	19.2
15.157	5.84063	216	14.7	19.2
15.167	5.83676	169	13.0	19.2
15.177	5.83290	176	13.3	19.2
15.188	5.82905	183	13.5	19.2
15.198	5.82520	203	14.2	19.2
15.208	5.82135	201	14.2	19.2
15.218	5.81751	222	14.9	19.2
15.228	5.81367	202	14.2	19.2
15.238	5.80984	173	13.2	19.2
15.248	5.80602	190	13.8	19.2
15.258	5.80220	191	13.8	19.2
15.268	5.79838	183	13.5	19.2
15.278	5.79457	210	14.5	19.2
15.289	5.79077	199	14.1	19.2
15.299	5.78697	176	13.3	19.2
15.309	5.78317	196	14.0	19.2
15.319	5.77938	173	13.2	19.2
15.329	5.77560	189	13.7	19.2
15.339	5.77182	185	13.6	19.2
15.349	5.76804	174	13.2	19.2
15.359	5.76427	204	14.3	19.2
15.369	5.76051	198	14.1	19.2
15.379	5.75675	196	14.0	19.2
15.390	5.75299	206	14.4	19.2
15.400	5.74924	202	14.2	19.2
15.410	5.74550	204	14.3	19.2
15.420	5.74176	202	14.2	19.2
15.430	5.73802	181	13.5	19.2
15.440	5.73429	231	15.2	19.2
15.450	5.73057	232	15.2	19.2
15.460	5.72684	185	13.6	19.2
15.470	5.72313	191	13.8	19.2
15.480	5.71942	194	13.9	19.2
15.491	5.71571	187	13.7	19.2
15.501	5.71201	204	14.3	19.2
15.511	5.70831	211	14.5	19.2
15.521	5.70462	197	14.0	19.2
15.531	5.70094	179	13.4	19.2
15.541	5.69725	184	13.6	19.2
15.551	5.69358	171	13.1	19.2
15.561	5.68990	211	14.5	19.2
15.571	5.68624	182	13.5	19.2
15.581	5.68257	206	14.4	19.2
15.592	5.67892	231	15.2	19.2

15.602	5.67526	208	14.4	19.2
15.612	5.67161	202	14.2	19.2
15.622	5.66797	222	14.9	19.2
15.632	5.66433	197	14.0	19.2
15.642	5.66070	224	15.0	19.2
15.652	5.65707	204	14.3	19.2
15.662	5.65344	203	14.2	19.2
15.672	5.64982	209	14.5	19.2
15.682	5.64621	225	15.0	19.2
15.692	5.64259	221	14.9	19.2
15.703	5.63899	214	14.6	19.2
15.713	5.63539	232	15.2	19.2
15.723	5.63179	211	14.5	19.2
15.733	5.62820	216	14.7	19.2
15.743	5.62461	223	14.9	19.2
15.753	5.62103	205	14.3	19.2
15.763	5.61745	205	14.3	19.2
15.773	5.61387	183	13.5	19.2
15.783	5.61030	206	14.4	19.2
15.793	5.60674	196	14.0	19.2
15.804	5.60318	196	14.0	19.2
15.814	5.59962	179	13.4	19.2
15.824	5.59607	206	14.4	19.2
15.834	5.59253	195	14.0	19.2
15.844	5.58898	174	13.2	19.2
15.854	5.58545	217	14.7	19.2
15.864	5.58191	194	13.9	19.2
15.874	5.57838	209	14.5	19.2
15.884	5.57486	223	14.9	19.2
15.894	5.57134	209	14.5	19.2
15.905	5.56783	200	14.1	19.2
15.915	5.56432	212	14.6	19.2
15.925	5.56081	198	14.1	19.2
15.935	5.55731	219	14.8	19.2
15.945	5.55381	214	14.6	19.2
15.955	5.55032	201	14.2	19.2
15.965	5.54683	220	14.8	19.2
15.975	5.54335	199	14.1	19.2
15.985	5.53987	197	14.0	19.2
15.995	5.53639	213	14.6	19.2
16.006	5.53292	207	14.4	19.2
16.016	5.52945	210	14.5	19.2
16.026	5.52599	203	14.2	19.2
16.036	5.52253	209	14.5	19.2
16.046	5.51908	207	14.4	19.2
16.056	5.51563	197	14.0	19.2
16.066	5.51219	214	14.6	19.2
16.076	5.50875	169	13.0	19.2
16.086	5.50531	215	14.7	19.2
16.096	5.50188	194	13.9	19.2

16.107	5.49845	223	14.9	19.2
16.117	5.49503	205	14.3	19.2
16.127	5.49161	232	15.2	19.2
16.137	5.48820	237	15.4	19.2
16.147	5.48479	233	15.3	19.2
16.157	5.48138	213	14.6	19.2
16.167	5.47798	187	13.7	19.2
16.177	5.47458	212	14.6	19.2
16.187	5.47119	222	14.9	19.2
16.197	5.46780	195	14.0	19.2
16.208	5.46442	199	14.1	19.2
16.218	5.46104	221	14.9	19.2
16.228	5.45766	221	14.9	19.2
16.238	5.45429	218	14.8	19.2
16.248	5.45092	203	14.2	19.2
16.258	5.44756	235	15.3	19.2
16.268	5.44420	220	14.8	19.2
16.278	5.44085	213	14.6	19.2
16.288	5.43750	217	14.7	19.2
16.298	5.43415	210	14.5	19.2
16.309	5.43081	206	14.4	19.2
16.319	5.42747	222	14.9	19.2
16.329	5.42413	220	14.8	19.2
16.339	5.42080	221	14.9	19.2
16.349	5.41748	223	14.9	19.2
16.359	5.41416	213	14.6	19.2
16.369	5.41084	203	14.2	19.2
16.379	5.40753	194	13.9	19.2
16.389	5.40422	203	14.2	19.2
16.399	5.40091	210	14.5	19.2
16.410	5.39761	219	14.8	19.2
16.420	5.39431	222	14.9	19.2
16.430	5.39102	223	14.9	19.2
16.440	5.38773	234	15.3	19.2
16.450	5.38444	221	14.9	19.2
16.460	5.38116	225	15.0	19.2
16.470	5.37789	226	15.0	19.2
16.480	5.37461	215	14.7	19.2
16.490	5.37135	209	14.5	19.2
16.500	5.36808	247	15.7	19.2
16.511	5.36482	223	14.9	19.2
16.521	5.36156	224	15.0	19.2
16.531	5.35831	269	16.4	19.2
16.541	5.35506	213	14.6	19.2
16.551	5.35182	218	14.8	19.2
16.561	5.34858	232	15.2	19.2
16.571	5.34534	241	15.5	19.2
16.581	5.34211	222	14.9	19.2
16.591	5.33888	216	14.7	19.2
16.601	5.33565	233	15.3	19.2

16.612	5.33243	221	14.9	19.2
16.622	5.32921	205	14.3	19.2
16.632	5.32600	242	15.6	19.2
16.642	5.32279	214	14.6	19.2
16.652	5.31958	213	14.6	19.2
16.662	5.31638	227	15.1	19.2
16.672	5.31319	234	15.3	19.2
16.682	5.30999	277	16.6	19.2
16.692	5.30680	203	14.2	19.2
16.702	5.30362	225	15.0	19.2
16.713	5.30043	224	15.0	19.2
16.723	5.29726	238	15.4	19.2
16.733	5.29408	203	14.2	19.2
16.743	5.29091	222	14.9	19.2
16.753	5.28774	220	14.8	19.2
16.763	5.28458	217	14.7	19.2
16.773	5.28142	230	15.2	19.2
16.783	5.27827	234	15.3	19.2
16.793	5.27511	223	14.9	19.2
16.803	5.27197	243	15.6	19.2
16.814	5.26882	220	14.8	19.2
16.824	5.26568	226	15.0	19.2
16.834	5.26255	238	15.4	19.2
16.844	5.25941	203	14.2	19.2
16.854	5.25628	225	15.0	19.2
16.864	5.25316	249	15.8	19.2
16.874	5.25004	253	15.9	19.2
16.884	5.24692	221	14.9	19.2
16.894	5.24381	212	14.6	19.2
16.904	5.24070	213	14.6	19.2
16.915	5.23759	238	15.4	19.2
16.925	5.23449	226	15.0	19.2
16.935	5.23139	240	15.5	19.2
16.945	5.22829	211	14.5	19.2
16.955	5.22520	217	14.7	19.2
16.965	5.22211	215	14.7	19.2
16.975	5.21903	249	15.8	19.2
16.985	5.21595	212	14.6	19.2
16.995	5.21287	241	15.5	19.2
17.005	5.20980	246	15.7	19.2
17.015	5.20673	233	15.3	19.2
17.026	5.20366	221	14.9	19.2
17.036	5.20060	231	15.2	19.2
17.046	5.19754	269	16.4	19.2
17.056	5.19449	225	15.0	19.2
17.066	5.19144	244	15.6	19.2
17.076	5.18839	215	14.7	19.2
17.086	5.18535	224	15.0	19.2
17.096	5.18231	220	14.8	19.2
17.106	5.17927	226	15.0	19.2

17.116	5.17624	238	15.4	19.2
17.127	5.17321	226	15.0	19.2
17.137	5.17018	233	15.3	19.2
17.147	5.16716	216	14.7	19.2
17.157	5.16414	221	14.9	19.2
17.167	5.16112	229	15.1	19.2
17.177	5.15811	236	15.4	19.2
17.187	5.15510	216	14.7	19.2
17.197	5.15210	240	15.5	19.2
17.207	5.14910	221	14.9	19.2
17.217	5.14610	244	15.6	19.2
17.228	5.14311	214	14.6	19.2
17.238	5.14012	197	14.0	19.2
17.248	5.13713	242	15.6	19.2
17.258	5.13415	227	15.1	19.2
17.268	5.13117	222	14.9	19.2
17.278	5.12819	255	16.0	19.2
17.288	5.12522	228	15.1	19.2
17.298	5.12225	250	15.8	19.2
17.308	5.11928	248	15.7	19.2
17.318	5.11632	226	15.0	19.2
17.329	5.11336	222	14.9	19.2
17.339	5.11040	248	15.7	19.2
17.349	5.10745	243	15.6	19.2
17.359	5.10450	236	15.4	19.2
17.369	5.10156	238	15.4	19.2
17.379	5.09862	283	16.8	19.2
17.389	5.09568	223	14.9	19.2
17.399	5.09274	192	13.9	19.2
17.409	5.08981	244	15.6	19.2
17.419	5.08688	238	15.4	19.2
17.430	5.08396	248	15.7	19.2
17.440	5.08104	231	15.2	19.2
17.450	5.07812	237	15.4	19.2
17.460	5.07520	245	15.7	19.2
17.470	5.07229	221	14.9	19.2
17.480	5.06938	224	15.0	19.2
17.490	5.06648	245	15.7	19.2
17.500	5.06358	245	15.7	19.2
17.510	5.06068	215	14.7	19.2
17.520	5.05779	241	15.5	19.2
17.531	5.05490	240	15.5	19.2
17.541	5.05201	225	15.0	19.2
17.551	5.04912	212	14.6	19.2
17.561	5.04624	239	15.5	19.2
17.571	5.04337	224	15.0	19.2
17.581	5.04049	202	14.2	19.2
17.591	5.03762	214	14.6	19.2
17.601	5.03475	227	15.1	19.2
17.611	5.03189	253	15.9	19.2

17.621	5.02903	226	15.0	19.2
17.632	5.02617	221	14.9	19.2
17.642	5.02331	253	15.9	19.2
17.652	5.02046	234	15.3	19.2
17.662	5.01761	217	14.7	19.2
17.672	5.01477	220	14.8	19.2
17.682	5.01193	268	16.4	19.2
17.692	5.00909	242	15.6	19.2
17.702	5.00626	232	15.2	19.2
17.712	5.00342	225	15.0	19.2
17.722	5.00060	251	15.8	19.2
17.733	4.99777	232	15.2	19.2
17.743	4.99495	216	14.7	19.2
17.753	4.99213	225	15.0	19.2
17.763	4.98931	225	15.0	19.2
17.773	4.98650	225	15.0	19.2
17.783	4.98369	233	15.3	19.2
17.793	4.98089	243	15.6	19.2
17.803	4.97808	226	15.0	19.2
17.813	4.97528	226	15.0	19.2
17.823	4.97249	279	16.7	19.2
17.834	4.96969	232	15.2	19.2
17.844	4.96690	242	15.6	19.2
17.854	4.96412	217	14.7	19.2
17.864	4.96133	237	15.4	19.2
17.874	4.95855	273	16.5	19.2
17.884	4.95578	235	15.3	19.2
17.894	4.95300	234	15.3	19.2
17.904	4.95023	239	15.5	19.2
17.914	4.94746	251	15.8	19.2
17.924	4.94470	216	14.7	19.2
17.935	4.94194	214	14.6	19.2
17.945	4.93918	229	15.1	19.2
17.955	4.93642	262	16.2	19.2
17.965	4.93367	239	15.5	19.2
17.975	4.93092	253	15.9	19.2
17.985	4.92817	206	14.4	19.2
17.995	4.92543	239	15.5	19.2
18.005	4.92269	243	15.6	19.2
18.015	4.91995	228	15.1	19.2
18.025	4.91722	230	15.2	19.2
18.036	4.91449	249	15.8	19.2
18.046	4.91176	221	14.9	19.2
18.056	4.90904	254	15.9	19.2
18.066	4.90632	238	15.4	19.2
18.076	4.90360	255	16.0	19.2
18.086	4.90088	251	15.8	19.2
18.096	4.89817	268	16.4	19.2
18.106	4.89546	252	15.9	19.2
18.116	4.89275	247	15.7	19.2

18.126	4.89005	235	15.3	19.2
18.137	4.88735	233	15.3	19.2
18.147	4.88465	229	15.1	19.2
18.157	4.88196	260	16.1	19.2
18.167	4.87927	244	15.6	19.2
18.177	4.87658	249	15.8	19.2
18.187	4.87389	245	15.7	19.2
18.197	4.87121	238	15.4	19.2
18.207	4.86853	219	14.8	19.2
18.217	4.86586	290	17.0	19.2
18.227	4.86318	253	15.9	19.2
18.237	4.86051	225	15.0	19.2
18.248	4.85785	246	15.7	19.2
18.258	4.85518	246	15.7	19.2
18.268	4.85252	232	15.2	19.2
18.278	4.84986	238	15.4	19.2
18.288	4.84721	259	16.1	19.2
18.298	4.84455	223	14.9	19.2
18.308	4.84190	244	15.6	19.2
18.318	4.83926	224	15.0	19.2
18.328	4.83661	224	15.0	19.2
18.338	4.83397	248	15.7	19.2
18.349	4.83134	259	16.1	19.2
18.359	4.82870	215	14.7	19.2
18.369	4.82607	231	15.2	19.2
18.379	4.82344	237	15.4	19.2
18.389	4.82081	224	15.0	19.2
18.399	4.81819	243	15.6	19.2
18.409	4.81557	245	15.7	19.2
18.419	4.81295	278	16.7	19.2
18.429	4.81034	243	15.6	19.2
18.439	4.80772	253	15.9	19.2
18.450	4.80512	234	15.3	19.2
18.460	4.80251	234	15.3	19.2
18.470	4.79991	251	15.8	19.2
18.480	4.79731	215	14.7	19.2
18.490	4.79471	235	15.3	19.2
18.500	4.79211	237	15.4	19.2
18.510	4.78952	232	15.2	19.2
18.520	4.78693	228	15.1	19.2
18.530	4.78435	240	15.5	19.2
18.540	4.78176	251	15.8	19.2
18.551	4.77918	269	16.4	19.2
18.561	4.77661	224	15.0	19.2
18.571	4.77403	257	16.0	19.2
18.581	4.77146	245	15.7	19.2
18.591	4.76889	248	15.7	19.2
18.601	4.76632	239	15.5	19.2
18.611	4.76376	222	14.9	19.2
18.621	4.76120	270	16.4	19.2

18.631	4.75864	238	15.4	19.2
18.641	4.75608	213	14.6	19.2
18.652	4.75353	228	15.1	19.2
18.662	4.75098	241	15.5	19.2
18.672	4.74844	244	15.6	19.2
18.682	4.74589	239	15.5	19.2
18.692	4.74335	250	15.8	19.2
18.702	4.74081	258	16.1	19.2
18.712	4.73828	256	16.0	19.2
18.722	4.73574	237	15.4	19.2
18.732	4.73321	265	16.3	19.2
18.742	4.73068	261	16.2	19.2
18.753	4.72816	256	16.0	19.2
18.763	4.72564	227	15.1	19.2
18.773	4.72312	254	15.9	19.2
18.783	4.72060	229	15.1	19.2
18.793	4.71809	252	15.9	19.2
18.803	4.71558	269	16.4	19.2
18.813	4.71307	249	15.8	19.2
18.823	4.71056	259	16.1	19.2
18.833	4.70806	241	15.5	19.2
18.843	4.70556	281	16.8	19.2
18.854	4.70306	237	15.4	19.2
18.864	4.70056	223	14.9	19.2
18.874	4.69807	232	15.2	19.2
18.884	4.69558	262	16.2	19.2
18.894	4.69309	244	15.6	19.2
18.904	4.69061	235	15.3	19.2
18.914	4.68813	262	16.2	19.2
18.924	4.68565	261	16.2	19.2
18.934	4.68317	234	15.3	19.2
18.944	4.68070	240	15.5	19.2
18.955	4.67823	247	15.7	19.2
18.965	4.67576	262	16.2	19.2
18.975	4.67329	236	15.4	19.2
18.985	4.67083	239	15.5	19.2
18.995	4.66837	256	16.0	19.2
19.005	4.66591	227	15.1	19.2
19.015	4.66346	279	16.7	19.2
19.025	4.66100	256	16.0	19.2
19.035	4.65855	236	15.4	19.2
19.045	4.65611	253	15.9	19.2
19.056	4.65366	217	14.7	19.2
19.066	4.65122	231	15.2	19.2
19.076	4.64878	282	16.8	19.2
19.086	4.64634	217	14.7	19.2
19.096	4.64391	214	14.6	19.2
19.106	4.64148	268	16.4	19.2
19.116	4.63905	259	16.1	19.2
19.126	4.63662	243	15.6	19.2

19.136	4.63419	253	15.9	19.2
19.146	4.63177	252	15.9	19.2
19.157	4.62935	254	15.9	19.2
19.167	4.62694	295	17.2	19.2
19.177	4.62452	253	15.9	19.2
19.187	4.62211	226	15.0	19.2
19.197	4.61970	251	15.8	19.2
19.207	4.61730	244	15.6	19.2
19.217	4.61489	252	15.9	19.2
19.227	4.61249	289	17.0	19.2
19.237	4.61009	252	15.9	19.2
19.247	4.60770	239	15.5	19.2
19.258	4.60530	254	15.9	19.2
19.268	4.60291	267	16.3	19.2
19.278	4.60052	271	16.5	19.2
19.288	4.59814	272	16.5	19.2
19.298	4.59575	195	14.0	19.2
19.308	4.59337	246	15.7	19.2
19.318	4.59099	255	16.0	19.2
19.328	4.58862	274	16.6	19.2
19.338	4.58624	246	15.7	19.2
19.348	4.58387	231	15.2	19.2
19.359	4.58151	248	15.7	19.2
19.369	4.57914	236	15.4	19.2
19.379	4.57678	208	14.4	19.2
19.389	4.57441	231	15.2	19.2
19.399	4.57206	256	16.0	19.2
19.409	4.56970	254	15.9	19.2
19.419	4.56735	235	15.3	19.2
19.429	4.56499	273	16.5	19.2
19.439	4.56265	248	15.7	19.2
19.449	4.56030	242	15.6	19.2
19.460	4.55795	238	15.4	19.2
19.470	4.55561	236	15.4	19.2
19.480	4.55327	255	16.0	19.2
19.490	4.55094	267	16.3	19.2
19.500	4.54860	246	15.7	19.2
19.510	4.54627	281	16.8	19.2
19.520	4.54394	260	16.1	19.2
19.530	4.54162	227	15.1	19.2
19.540	4.53929	242	15.6	19.2
19.550	4.53697	258	16.1	19.2
19.560	4.53465	215	14.7	19.2
19.571	4.53233	215	14.7	19.2
19.581	4.53002	250	15.8	19.2
19.591	4.52770	251	15.8	19.2
19.601	4.52539	258	16.1	19.2
19.611	4.52309	245	15.7	19.2
19.621	4.52078	244	15.6	19.2
19.631	4.51848	251	15.8	19.2

19.641	4.51618	254	15.9	19.2
19.651	4.51388	254	15.9	19.2
19.661	4.51158	262	16.2	19.2
19.672	4.50929	252	15.9	19.2
19.682	4.50700	244	15.6	19.2
19.692	4.50471	237	15.4	19.2
19.702	4.50242	239	15.5	19.2
19.712	4.50014	248	15.7	19.2
19.722	4.49786	259	16.1	19.2
19.732	4.49558	249	15.8	19.2
19.742	4.49330	258	16.1	19.2
19.752	4.49103	249	15.8	19.2
19.762	4.48876	257	16.0	19.2
19.773	4.48649	249	15.8	19.2
19.783	4.48422	261	16.2	19.2
19.793	4.48195	241	15.5	19.2
19.803	4.47969	259	16.1	19.2
19.813	4.47743	263	16.2	19.2
19.823	4.47517	267	16.3	19.2
19.833	4.47291	244	15.6	19.2
19.843	4.47066	246	15.7	19.2
19.853	4.46841	245	15.7	19.2
19.863	4.46616	244	15.6	19.2
19.874	4.46391	241	15.5	19.2
19.884	4.46167	249	15.8	19.2
19.894	4.45943	241	15.5	19.2
19.904	4.45719	248	15.7	19.2
19.914	4.45495	241	15.5	19.2
19.924	4.45271	247	15.7	19.2
19.934	4.45048	232	15.2	19.2
19.944	4.44825	251	15.8	19.2
19.954	4.44602	217	14.7	19.2
19.964	4.44380	247	15.7	19.2
19.975	4.44157	265	16.3	19.2
19.985	4.43935	269	16.4	19.2
19.995	4.43713	215	14.7	19.2
20.005	4.43491	232	15.2	19.2
20.015	4.43270	266	16.3	19.2
20.025	4.43049	236	15.4	19.2
20.035	4.42827	223	14.9	19.2
20.045	4.42607	230	15.2	19.2
20.055	4.42386	258	16.1	19.2
20.065	4.42166	258	16.1	19.2
20.076	4.41945	261	16.2	19.2
20.086	4.41726	231	15.2	19.2
20.096	4.41506	260	16.1	19.2
20.106	4.41286	247	15.7	19.2
20.116	4.41067	267	16.3	19.2
20.126	4.40848	268	16.4	19.2
20.136	4.40629	250	15.8	19.2

20.146	4.40411	248	15.7	19.2
20.156	4.40192	250	15.8	19.2
20.166	4.39974	226	15.0	19.2
20.177	4.39756	236	15.4	19.2
20.187	4.39538	237	15.4	19.2
20.197	4.39321	248	15.7	19.2
20.207	4.39104	227	15.1	19.2
20.217	4.38887	251	15.8	19.2
20.227	4.38670	242	15.6	19.2
20.237	4.38453	236	15.4	19.2
20.247	4.38237	259	16.1	19.2
20.257	4.38020	246	15.7	19.2
20.267	4.37804	231	15.2	19.2
20.278	4.37589	225	15.0	19.2
20.288	4.37373	268	16.4	19.2
20.298	4.37158	266	16.3	19.2
20.308	4.36943	230	15.2	19.2
20.318	4.36728	257	16.0	19.2
20.328	4.36513	254	15.9	19.2
20.338	4.36299	245	15.7	19.2
20.348	4.36084	277	16.6	19.2
20.358	4.35870	277	16.6	19.2
20.368	4.35656	267	16.3	19.2
20.379	4.35443	248	15.7	19.2
20.389	4.35229	245	15.7	19.2
20.399	4.35016	265	16.3	19.2
20.409	4.34803	259	16.1	19.2
20.419	4.34590	244	15.6	19.2
20.429	4.34378	264	16.2	19.2
20.439	4.34165	281	16.8	19.2
20.449	4.33953	234	15.3	19.2
20.459	4.33741	254	15.9	19.2
20.469	4.33530	258	16.1	19.2
20.480	4.33318	280	16.7	19.2
20.490	4.33107	245	15.7	19.2
20.500	4.32896	229	15.1	19.2
20.510	4.32685	301	17.3	19.2
20.520	4.32474	280	16.7	19.2
20.530	4.32264	267	16.3	19.2
20.540	4.32054	263	16.2	19.2
20.550	4.31843	242	15.6	19.2
20.560	4.31634	249	15.8	19.2
20.570	4.31424	236	15.4	19.2
20.581	4.31215	246	15.7	19.2
20.591	4.31005	256	16.0	19.2
20.601	4.30796	263	16.2	19.2
20.611	4.30588	262	16.2	19.2
20.621	4.30379	246	15.7	19.2
20.631	4.30171	277	16.6	19.2
20.641	4.29962	242	15.6	19.2

20.651	4.29754	252	15.9	19.2
20.661	4.29547	232	15.2	19.2
20.671	4.29339	248	15.7	19.2
20.682	4.29132	270	16.4	19.2
20.692	4.28924	259	16.1	19.2
20.702	4.28717	271	16.5	19.2
20.712	4.28511	253	15.9	19.2
20.722	4.28304	257	16.0	19.2
20.732	4.28098	240	15.5	19.2
20.742	4.27892	285	16.9	19.2
20.752	4.27686	271	16.5	19.2
20.762	4.27480	226	15.0	19.2
20.772	4.27274	308	17.5	19.2
20.783	4.27069	262	16.2	19.2
20.793	4.26864	249	15.8	19.2
20.803	4.26659	281	16.8	19.2
20.813	4.26454	274	16.6	19.2
20.823	4.26250	245	15.7	19.2
20.833	4.26045	251	15.8	19.2
20.843	4.25841	252	15.9	19.2
20.853	4.25637	238	15.4	19.2
20.863	4.25433	263	16.2	19.2
20.873	4.25230	287	16.9	19.2
20.883	4.25026	256	16.0	19.2
20.894	4.24823	261	16.2	19.2
20.904	4.24620	254	15.9	19.2
20.914	4.24418	246	15.7	19.2
20.924	4.24215	252	15.9	19.2
20.934	4.24013	283	16.8	19.2
20.944	4.23810	260	16.1	19.2
20.954	4.23608	231	15.2	19.2
20.964	4.23407	281	16.8	19.2
20.974	4.23205	286	16.9	19.2
20.984	4.23004	267	16.3	19.2
20.995	4.22802	286	16.9	19.2
21.005	4.22601	266	16.3	19.2
21.015	4.22401	260	16.1	19.2
21.025	4.22200	238	15.4	19.2
21.035	4.22000	264	16.2	19.2
21.045	4.21799	288	17.0	19.2
21.055	4.21599	257	16.0	19.2
21.065	4.21399	267	16.3	19.2
21.075	4.21200	287	16.9	19.2
21.085	4.21000	254	15.9	19.2
21.096	4.20801	260	16.1	19.2
21.106	4.20602	284	16.9	19.2
21.116	4.20403	240	15.5	19.2
21.126	4.20204	275	16.6	19.2
21.136	4.20006	259	16.1	19.2
21.146	4.19808	288	17.0	19.2

21.156	4.19609	262	16.2	19.2
21.166	4.19412	299	17.3	19.2
21.176	4.19214	283	16.8	19.2
21.186	4.19016	250	15.8	19.2
21.197	4.18819	256	16.0	19.2
21.207	4.18622	274	16.6	19.2
21.217	4.18425	285	16.9	19.2
21.227	4.18228	263	16.2	19.2
21.237	4.18031	272	16.5	19.2
21.247	4.17835	255	16.0	19.2
21.257	4.17639	279	16.7	19.2
21.267	4.17443	253	15.9	19.2
21.277	4.17247	282	16.8	19.2
21.287	4.17051	252	15.9	19.2
21.298	4.16856	263	16.2	19.2
21.308	4.16660	265	16.3	19.2
21.318	4.16465	248	15.7	19.2
21.328	4.16270	265	16.3	19.2
21.338	4.16076	282	16.8	19.2
21.348	4.15881	249	15.8	19.2
21.358	4.15687	280	16.7	19.2
21.368	4.15492	270	16.4	19.2
21.378	4.15298	257	16.0	19.2
21.388	4.15105	317	17.8	19.2
21.399	4.14911	265	16.3	19.2
21.409	4.14718	294	17.1	19.2
21.419	4.14524	286	16.9	19.2
21.429	4.14331	287	16.9	19.2
21.439	4.14138	264	16.2	19.2
21.449	4.13946	263	16.2	19.2
21.459	4.13753	285	16.9	19.2
21.469	4.13561	269	16.4	19.2
21.479	4.13369	241	15.5	19.2
21.489	4.13177	270	16.4	19.2
21.500	4.12985	247	15.7	19.2
21.510	4.12793	280	16.7	19.2
21.520	4.12602	274	16.6	19.2
21.530	4.12410	309	17.6	19.2
21.540	4.12219	280	16.7	19.2
21.550	4.12028	277	16.6	19.2
21.560	4.11838	275	16.6	19.2
21.570	4.11647	292	17.1	19.2
21.580	4.11457	316	17.8	19.2
21.590	4.11267	276	16.6	19.2
21.601	4.11077	263	16.2	19.2
21.611	4.10887	253	15.9	19.2
21.621	4.10697	273	16.5	19.2
21.631	4.10508	269	16.4	19.2
21.641	4.10318	278	16.7	19.2
21.651	4.10129	284	16.9	19.2

21.661	4.09940	268	16.4	19.2
21.671	4.09752	258	16.1	19.2
21.681	4.09563	314	17.7	19.2
21.691	4.09375	249	15.8	19.2
21.702	4.09186	269	16.4	19.2
21.712	4.08998	272	16.5	19.2
21.722	4.08810	267	16.3	19.2
21.732	4.08623	302	17.4	19.2
21.742	4.08435	292	17.1	19.2
21.752	4.08248	284	16.9	19.2
21.762	4.08061	270	16.4	19.2
21.772	4.07874	284	16.9	19.2
21.782	4.07687	275	16.6	19.2
21.792	4.07500	295	17.2	19.2
21.803	4.07314	301	17.3	19.2
21.813	4.07127	283	16.8	19.2
21.823	4.06941	314	17.7	19.2
21.833	4.06755	252	15.9	19.2
21.843	4.06570	284	16.9	19.2
21.853	4.06384	312	17.7	19.2
21.863	4.06199	265	16.3	19.2
21.873	4.06013	270	16.4	19.2
21.883	4.05828	296	17.2	19.2
21.893	4.05643	279	16.7	19.2
21.904	4.05458	309	17.6	19.2
21.914	4.05274	294	17.1	19.2
21.924	4.05089	297	17.2	19.2
21.934	4.04905	299	17.3	19.2
21.944	4.04721	341	18.5	19.2
21.954	4.04537	268	16.4	19.2
21.964	4.04354	283	16.8	19.2
21.974	4.04170	253	15.9	19.2
21.984	4.03987	294	17.1	19.2
21.994	4.03803	251	15.8	19.2
22.005	4.03620	314	17.7	19.2
22.015	4.03437	278	16.7	19.2
22.025	4.03255	284	16.9	19.2
22.035	4.03072	313	17.7	19.2
22.045	4.02890	275	16.6	19.2
22.055	4.02708	305	17.5	19.2
22.065	4.02526	303	17.4	19.2
22.075	4.02344	266	16.3	19.2
22.085	4.02162	262	16.2	19.2
22.095	4.01980	283	16.8	19.2
22.106	4.01799	287	16.9	19.2
22.116	4.01618	261	16.2	19.2
22.126	4.01437	309	17.6	19.2
22.136	4.01256	307	17.5	19.2
22.146	4.01075	280	16.7	19.2
22.156	4.00895	305	17.5	19.2

22.166	4.00714	302	17.4	19.2
22.176	4.00534	310	17.6	19.2
22.186	4.00354	279	16.7	19.2
22.196	4.00174	280	16.7	19.2
22.206	3.99995	314	17.7	19.2
22.217	3.99815	285	16.9	19.2
22.227	3.99636	291	17.1	19.2
22.237	3.99456	293	17.1	19.2
22.247	3.99277	298	17.3	19.2
22.257	3.99098	258	16.1	19.2
22.267	3.98920	281	16.8	19.2
22.277	3.98741	305	17.5	19.2
22.287	3.98563	301	17.3	19.2
22.297	3.98385	325	18.0	19.2
22.307	3.98206	301	17.3	19.2
22.318	3.98029	270	16.4	19.2
22.328	3.97851	292	17.1	19.2
22.338	3.97673	309	17.6	19.2
22.348	3.97496	299	17.3	19.2
22.358	3.97319	273	16.5	19.2
22.368	3.97141	269	16.4	19.2
22.378	3.96964	284	16.9	19.2
22.388	3.96788	315	17.7	19.2
22.398	3.96611	313	17.7	19.2
22.408	3.96435	287	16.9	19.2
22.419	3.96258	301	17.3	19.2
22.429	3.96082	317	17.8	19.2
22.439	3.95906	323	18.0	19.2
22.449	3.95730	310	17.6	19.2
22.459	3.95555	303	17.4	19.2
22.469	3.95379	290	17.0	19.2
22.479	3.95204	309	17.6	19.2
22.489	3.95029	291	17.1	19.2
22.499	3.94854	269	16.4	19.2
22.509	3.94679	290	17.0	19.2
22.520	3.94504	300	17.3	19.2
22.530	3.94329	279	16.7	19.2
22.540	3.94155	306	17.5	19.2
22.550	3.93981	290	17.0	19.2
22.560	3.93807	277	16.6	19.2
22.570	3.93633	292	17.1	19.2
22.580	3.93459	287	16.9	19.2
22.590	3.93285	288	17.0	19.2
22.600	3.93112	294	17.1	19.2
22.610	3.92939	291	17.1	19.2
22.621	3.92765	304	17.4	19.2
22.631	3.92592	301	17.3	19.2
22.641	3.92420	302	17.4	19.2
22.651	3.92247	287	16.9	19.2
22.661	3.92074	321	17.9	19.2

22.671	3.91902	303	17.4	19.2
22.681	3.91730	295	17.2	19.2
22.691	3.91558	287	16.9	19.2
22.701	3.91386	290	17.0	19.2
22.711	3.91214	280	16.7	19.2
22.722	3.91042	291	17.1	19.2
22.732	3.90871	296	17.2	19.2
22.742	3.90700	298	17.3	19.2
22.752	3.90529	299	17.3	19.2
22.762	3.90358	322	17.9	19.2
22.772	3.90187	289	17.0	19.2
22.782	3.90016	319	17.9	19.2
22.792	3.89846	267	16.3	19.2
22.802	3.89675	283	16.8	19.2
22.812	3.89505	327	18.1	19.2
22.823	3.89335	310	17.6	19.2
22.833	3.89165	310	17.6	19.2
22.843	3.88995	284	16.9	19.2
22.853	3.88826	281	16.8	19.2
22.863	3.88656	293	17.1	19.2
22.873	3.88487	333	18.2	19.2
22.883	3.88318	296	17.2	19.2
22.893	3.88149	301	17.3	19.2
22.903	3.87980	331	18.2	19.2
22.913	3.87811	336	18.3	19.2
22.924	3.87642	291	17.1	19.2
22.934	3.87474	289	17.0	19.2
22.944	3.87306	325	18.0	19.2
22.954	3.87138	274	16.6	19.2
22.964	3.86970	312	17.7	19.2
22.974	3.86802	310	17.6	19.2
22.984	3.86634	338	18.4	19.2
22.994	3.86467	308	17.5	19.2
23.004	3.86299	254	15.9	19.2
23.014	3.86132	315	17.7	19.2
23.025	3.85965	301	17.3	19.2
23.035	3.85798	306	17.5	19.2
23.045	3.85631	314	17.7	19.2
23.055	3.85465	321	17.9	19.2
23.065	3.85298	352	18.8	19.2
23.075	3.85132	325	18.0	19.2
23.085	3.84965	329	18.1	19.2
23.095	3.84799	312	17.7	19.2
23.105	3.84633	313	17.7	19.2
23.115	3.84468	305	17.5	19.2
23.126	3.84302	299	17.3	19.2
23.136	3.84137	302	17.4	19.2
23.146	3.83971	324	18.0	19.2
23.156	3.83806	299	17.3	19.2
23.166	3.83641	293	17.1	19.2

23.176	3.83476	331	18.2	19.2
23.186	3.83311	294	17.1	19.2
23.196	3.83147	320	17.9	19.2
23.206	3.82982	333	18.2	19.2
23.216	3.82818	328	18.1	19.2
23.227	3.82654	367	19.2	19.2
23.237	3.82490	362	19.0	19.2
23.247	3.82326	326	18.1	19.2
23.257	3.82162	295	17.2	19.2
23.267	3.81999	300	17.3	19.2
23.277	3.81835	317	17.8	19.2
23.287	3.81672	295	17.2	19.2
23.297	3.81509	337	18.4	19.2
23.307	3.81346	310	17.6	19.2
23.317	3.81183	299	17.3	19.2
23.328	3.81020	314	17.7	19.2
23.338	3.80857	320	17.9	19.2
23.348	3.80695	305	17.5	19.2
23.358	3.80533	308	17.5	19.2
23.368	3.80371	304	17.4	19.2
23.378	3.80208	311	17.6	19.2
23.388	3.80047	300	17.3	19.2
23.398	3.79885	291	17.1	19.2
23.408	3.79723	302	17.4	19.2
23.418	3.79562	318	17.8	19.2
23.429	3.79400	338	18.4	19.2
23.439	3.79239	332	18.2	19.2
23.449	3.79078	306	17.5	19.2
23.459	3.78917	325	18.0	19.2
23.469	3.78756	342	18.5	19.2
23.479	3.78596	298	17.3	19.2
23.489	3.78435	306	17.5	19.2
23.499	3.78275	373	19.3	19.2
23.509	3.78115	296	17.2	19.2
23.519	3.77955	300	17.3	19.2
23.529	3.77795	302	17.4	19.2
23.540	3.77635	323	18.0	19.2
23.550	3.77475	325	18.0	19.2
23.560	3.77316	323	18.0	19.2
23.570	3.77156	332	18.2	19.2
23.580	3.76997	326	18.1	19.2
23.590	3.76838	324	18.0	19.2
23.600	3.76679	307	17.5	19.2
23.610	3.76520	310	17.6	19.2
23.620	3.76362	346	18.6	19.2
23.630	3.76203	315	17.7	19.2
23.641	3.76045	337	18.4	19.2
23.651	3.75886	333	18.2	19.2
23.661	3.75728	313	17.7	19.2
23.671	3.75570	351	18.7	19.2

23.681	3.75412	309	17.6	19.2
23.691	3.75254	331	18.2	19.2
23.701	3.75097	318	17.8	19.2
23.711	3.74939	326	18.1	19.2
23.721	3.74782	314	17.7	19.2
23.731	3.74625	341	18.5	19.2
23.742	3.74468	366	19.1	19.2
23.752	3.74311	358	18.9	19.2
23.762	3.74154	351	18.7	19.2
23.772	3.73997	333	18.2	19.2
23.782	3.73841	345	18.6	19.2
23.792	3.73684	324	18.0	19.2
23.802	3.73528	313	17.7	19.2
23.812	3.73372	316	17.8	19.2
23.822	3.73216	323	18.0	19.2
23.832	3.73060	332	18.2	19.2
23.843	3.72904	315	17.7	19.2
23.853	3.72749	374	19.3	19.2
23.863	3.72593	345	18.6	19.2
23.873	3.72438	354	18.8	19.2
23.883	3.72283	326	18.1	19.2
23.893	3.72128	336	18.3	19.2
23.903	3.71973	349	18.7	19.2
23.913	3.71818	341	18.5	19.2
23.923	3.71663	339	18.4	19.2
23.933	3.71509	347	18.6	19.2
23.944	3.71354	312	17.7	19.2
23.954	3.71200	352	18.8	19.2
23.964	3.71046	299	17.3	19.2
23.974	3.70892	314	17.7	19.2
23.984	3.70738	339	18.4	19.2
23.994	3.70584	301	17.3	19.2
24.004	3.70431	313	17.7	19.2
24.014	3.70277	345	18.6	19.2
24.024	3.70124	364	19.1	19.2
24.034	3.69970	330	18.2	19.2
24.045	3.69817	380	19.5	19.2
24.055	3.69664	293	17.1	19.2
24.065	3.69512	348	18.7	19.2
24.075	3.69359	303	17.4	19.2
24.085	3.69206	342	18.5	19.2
24.095	3.69054	375	19.4	19.2
24.105	3.68901	317	17.8	19.2
24.115	3.68749	319	17.9	19.2
24.125	3.68597	302	17.4	19.2
24.135	3.68445	350	18.7	19.2
24.146	3.68293	345	18.6	19.2
24.156	3.68142	312	17.7	19.2
24.166	3.67990	319	17.9	19.2
24.176	3.67839	295	17.2	19.2

24.186	3.67687	369	19.2	19.2
24.196	3.67536	337	18.4	19.2
24.206	3.67385	345	18.6	19.2
24.216	3.67234	341	18.5	19.2
24.226	3.67083	355	18.8	19.2
24.236	3.66933	317	17.8	19.2
24.247	3.66782	350	18.7	19.2
24.257	3.66632	387	19.7	19.2
24.267	3.66481	345	18.6	19.2
24.277	3.66331	385	19.6	19.2
24.287	3.66181	336	18.3	19.2
24.297	3.66031	348	18.7	19.2
24.307	3.65881	376	19.4	19.2
24.317	3.65732	339	18.4	19.2
24.327	3.65582	359	18.9	19.2
24.337	3.65433	336	18.3	19.2
24.348	3.65284	347	18.6	19.2
24.358	3.65134	341	18.5	19.2
24.368	3.64985	334	18.3	19.2
24.378	3.64836	339	18.4	19.2
24.388	3.64688	358	18.9	19.2
24.398	3.64539	347	18.6	19.2
24.408	3.64390	342	18.5	19.2
24.418	3.64242	345	18.6	19.2
24.428	3.64094	399	20.0	19.2
24.438	3.63946	378	19.4	19.2
24.449	3.63797	363	19.1	19.2
24.459	3.63650	333	18.2	19.2
24.469	3.63502	352	18.8	19.2
24.479	3.63354	387	19.7	19.2
24.489	3.63206	374	19.3	19.2
24.499	3.63059	341	18.5	19.2
24.509	3.62912	355	18.8	19.2
24.519	3.62765	342	18.5	19.2
24.529	3.62617	344	18.5	19.2
24.539	3.62471	357	18.9	19.2
24.550	3.62324	382	19.5	19.2
24.560	3.62177	306	17.5	19.2
24.570	3.62030	368	19.2	19.2
24.580	3.61884	351	18.7	19.2
24.590	3.61738	367	19.2	19.2
24.600	3.61591	355	18.8	19.2
24.610	3.61445	360	19.0	19.2
24.620	3.61299	350	18.7	19.2
24.630	3.61153	342	18.5	19.2
24.640	3.61008	390	19.7	19.2
24.651	3.60862	365	19.1	19.2
24.661	3.60717	363	19.1	19.2
24.671	3.60571	345	18.6	19.2
24.681	3.60426	358	18.9	19.2

24.691	3.60281	358	18.9	19.2
24.701	3.60136	379	19.5	19.2
24.711	3.59991	372	19.3	19.2
24.721	3.59846	373	19.3	19.2
24.731	3.59701	379	19.5	19.2
24.741	3.59557	390	19.7	19.2
24.752	3.59413	372	19.3	19.2
24.762	3.59268	345	18.6	19.2
24.772	3.59124	371	19.3	19.2
24.782	3.58980	357	18.9	19.2
24.792	3.58836	364	19.1	19.2
24.802	3.58692	359	18.9	19.2
24.812	3.58548	388	19.7	19.2
24.822	3.58405	395	19.9	19.2
24.832	3.58261	369	19.2	19.2
24.842	3.58118	359	18.9	19.2
24.852	3.57975	406	20.1	19.2
24.863	3.57832	356	18.9	19.2
24.873	3.57689	353	18.8	19.2
24.883	3.57546	363	19.1	19.2
24.893	3.57403	355	18.8	19.2
24.903	3.57260	379	19.5	19.2
24.913	3.57118	373	19.3	19.2
24.923	3.56975	378	19.4	19.2
24.933	3.56833	372	19.3	19.2
24.943	3.56691	372	19.3	19.2
24.953	3.56549	346	18.6	19.2
24.964	3.56407	382	19.5	19.2
24.974	3.56265	370	19.2	19.2
24.984	3.56123	383	19.6	19.2
24.994	3.55982	405	20.1	19.2
25.004	3.55840	370	19.2	19.2
25.014	3.55699	387	19.7	19.2
25.024	3.55558	397	19.9	19.2
25.034	3.55416	357	18.9	19.2
25.044	3.55275	382	19.5	19.2
25.054	3.55134	346	18.6	19.2
25.065	3.54994	381	19.5	19.2
25.075	3.54853	381	19.5	19.2
25.085	3.54712	363	19.1	19.2
25.095	3.54572	381	19.5	19.2
25.105	3.54432	386	19.6	19.2
25.115	3.54291	381	19.5	19.2
25.125	3.54151	402	20.0	19.2
25.135	3.54011	397	19.9	19.2
25.145	3.53871	381	19.5	19.2
25.155	3.53732	374	19.3	19.2
25.166	3.53592	388	19.7	19.2
25.176	3.53452	365	19.1	19.2
25.186	3.53313	393	19.8	19.2

25.196	3.53174	395	19.9	19.2
25.206	3.53034	397	19.9	19.2
25.216	3.52895	434	20.8	19.2
25.226	3.52756	378	19.4	19.2
25.236	3.52617	393	19.8	19.2
25.246	3.52479	363	19.1	19.2
25.256	3.52340	409	20.2	19.2
25.267	3.52201	414	20.3	19.2
25.277	3.52063	398	19.9	19.2
25.287	3.51925	382	19.5	19.2
25.297	3.51786	384	19.6	19.2
25.307	3.51648	438	20.9	19.2
25.317	3.51510	415	20.4	19.2
25.327	3.51372	426	20.6	19.2
25.337	3.51235	388	19.7	19.2
25.347	3.51097	401	20.0	19.2
25.357	3.50959	416	20.4	19.2
25.368	3.50822	403	20.1	19.2
25.378	3.50685	419	20.5	19.2
25.388	3.50548	420	20.5	19.2
25.398	3.50410	412	20.3	19.2
25.408	3.50273	400	20.0	19.2
25.418	3.50137	417	20.4	19.2
25.428	3.50000	420	20.5	19.2
25.438	3.49863	399	20.0	19.2
25.448	3.49727	416	20.4	19.2
25.458	3.49590	397	19.9	19.2
25.469	3.49454	395	19.9	19.2
25.479	3.49318	387	19.7	19.2
25.489	3.49181	404	20.1	19.2
25.499	3.49045	448	21.2	19.2
25.509	3.48909	397	19.9	19.2
25.519	3.48774	398	19.9	19.2
25.529	3.48638	390	19.7	19.2
25.539	3.48502	414	20.3	19.2
25.549	3.48367	381	19.5	19.2
25.559	3.48232	405	20.1	19.2
25.570	3.48096	410	20.2	19.2
25.580	3.47961	427	20.7	19.2
25.590	3.47826	367	19.2	19.2
25.600	3.47691	393	19.8	19.2
25.610	3.47556	403	20.1	19.2
25.620	3.47422	428	20.7	19.2
25.630	3.47287	399	20.0	19.2
25.640	3.47153	411	20.3	19.2
25.650	3.47018	405	20.1	19.2
25.660	3.46884	406	20.1	19.2
25.671	3.46750	407	20.2	19.2
25.681	3.46616	401	20.0	19.2
25.691	3.46482	415	20.4	19.2

25.701	3.46348	448	21.2	19.2
25.711	3.46214	421	20.5	19.2
25.721	3.46080	423	20.6	19.2
25.731	3.45947	419	20.5	19.2
25.741	3.45813	374	19.3	19.2
25.751	3.45680	415	20.4	19.2
25.761	3.45547	355	18.8	19.2
25.772	3.45414	396	19.9	19.2
25.782	3.45281	446	21.1	19.2
25.792	3.45148	412	20.3	19.2
25.802	3.45015	400	20.0	19.2
25.812	3.44882	438	20.9	19.2
25.822	3.44750	404	20.1	19.2
25.832	3.44617	414	20.3	19.2
25.842	3.44485	454	21.3	19.2
25.852	3.44353	417	20.4	19.2
25.862	3.44220	399	20.0	19.2
25.873	3.44088	440	21.0	19.2
25.883	3.43956	410	20.2	19.2
25.893	3.43824	405	20.1	19.2
25.903	3.43693	432	20.8	19.2
25.913	3.43561	396	19.9	19.2
25.923	3.43429	405	20.1	19.2
25.933	3.43298	447	21.1	19.2
25.943	3.43167	440	21.0	19.2
25.953	3.43035	437	20.9	19.2
25.963	3.42904	434	20.8	19.2
25.974	3.42773	414	20.3	19.2
25.984	3.42642	435	20.9	19.2
25.994	3.42511	437	20.9	19.2
26.004	3.42381	411	20.3	19.2
26.014	3.42250	464	21.5	19.2
26.024	3.42120	442	21.0	19.2
26.034	3.41989	409	20.2	19.2
26.044	3.41859	425	20.6	19.2
26.054	3.41729	457	21.4	19.2
26.064	3.41599	438	20.9	19.2
26.075	3.41468	413	20.3	19.2
26.085	3.41339	430	20.7	19.2
26.095	3.41209	454	21.3	19.2
26.105	3.41079	450	21.2	19.2
26.115	3.40949	420	20.5	19.2
26.125	3.40820	494	22.2	19.2
26.135	3.40691	419	20.5	19.2
26.145	3.40561	453	21.3	19.2
26.155	3.40432	450	21.2	19.2
26.165	3.40303	440	21.0	19.2
26.175	3.40174	477	21.8	19.2
26.186	3.40045	404	20.1	19.2
26.196	3.39916	468	21.6	19.2

26.206	3.39787	440	21.0	19.2
26.216	3.39659	449	21.2	19.2
26.226	3.39530	436	20.9	19.2
26.236	3.39402	466	21.6	19.2
26.246	3.39274	468	21.6	19.2
26.256	3.39145	445	21.1	19.2
26.266	3.39017	424	20.6	19.2
26.276	3.38889	456	21.4	19.2
26.287	3.38761	447	21.1	19.2
26.297	3.38634	490	22.1	19.2
26.307	3.38506	427	20.7	19.2
26.317	3.38378	444	21.1	19.2
26.327	3.38251	463	21.5	19.2
26.337	3.38123	454	21.3	19.2
26.347	3.37996	476	21.8	19.2
26.357	3.37869	437	20.9	19.2
26.367	3.37742	439	21.0	19.2
26.377	3.37615	475	21.8	19.2
26.388	3.37488	426	20.6	19.2
26.398	3.37361	431	20.8	19.2
26.408	3.37234	429	20.7	19.2
26.418	3.37108	476	21.8	19.2
26.428	3.36981	464	21.5	19.2
26.438	3.36855	464	21.5	19.2
26.448	3.36728	459	21.4	19.2
26.458	3.36602	455	21.3	19.2
26.468	3.36476	441	21.0	19.2
26.478	3.36350	472	21.7	19.2
26.489	3.36224	472	21.7	19.2
26.499	3.36098	450	21.2	19.2
26.509	3.35972	464	21.5	19.2
26.519	3.35847	478	21.9	19.2
26.529	3.35721	487	22.1	19.2
26.539	3.35596	480	21.9	19.2
26.549	3.35470	443	21.0	19.2
26.559	3.35345	473	21.7	19.2
26.569	3.35220	468	21.6	19.2
26.579	3.35095	451	21.2	19.2
26.590	3.34970	483	22.0	19.2
26.600	3.34845	454	21.3	19.2
26.610	3.34720	472	21.7	19.2
26.620	3.34595	498	22.3	19.2
26.630	3.34471	481	21.9	19.2
26.640	3.34346	450	21.2	19.2
26.650	3.34222	449	21.2	19.2
26.660	3.34097	501	22.4	19.2
26.670	3.33973	498	22.3	19.2
26.680	3.33849	484	22.0	19.2
26.691	3.33725	467	21.6	19.2
26.701	3.33601	473	21.7	19.2

26.711	3.33477	474	21.8	19.2
26.721	3.33354	451	21.2	19.2
26.731	3.33230	480	21.9	19.2
26.741	3.33106	514	22.7	19.2
26.751	3.32983	454	21.3	19.2
26.761	3.32860	431	20.8	19.2
26.771	3.32736	466	21.6	19.2
26.781	3.32613	431	20.8	19.2
26.792	3.32490	479	21.9	19.2
26.802	3.32367	447	21.1	19.2
26.812	3.32244	467	21.6	19.2
26.822	3.32121	457	21.4	19.2
26.832	3.31999	484	22.0	19.2
26.842	3.31876	467	21.6	19.2
26.852	3.31753	480	21.9	19.2
26.862	3.31631	460	21.4	19.2
26.872	3.31509	457	21.4	19.2
26.882	3.31386	468	21.6	19.2
26.893	3.31264	454	21.3	19.2
26.903	3.31142	484	22.0	19.2
26.913	3.31020	472	21.7	19.2
26.923	3.30898	527	23.0	19.2
26.933	3.30777	474	21.8	19.2
26.943	3.30655	466	21.6	19.2
26.953	3.30533	472	21.7	19.2
26.963	3.30412	487	22.1	19.2
26.973	3.30290	496	22.3	19.2
26.983	3.30169	505	22.5	19.2
26.994	3.30048	476	21.8	19.2
27.004	3.29927	447	21.1	19.2
27.014	3.29806	440	21.0	19.2
27.024	3.29685	460	21.4	19.2
27.034	3.29564	521	22.8	19.2
27.044	3.29443	476	21.8	19.2
27.054	3.29322	437	20.9	19.2
27.064	3.29202	524	22.9	19.2
27.074	3.29081	478	21.9	19.2
27.084	3.28961	477	21.8	19.2
27.095	3.28840	457	21.4	19.2
27.105	3.28720	483	22.0	19.2
27.115	3.28600	491	22.2	19.2
27.125	3.28480	463	21.5	19.2
27.135	3.28360	494	22.2	19.2
27.145	3.28240	461	21.5	19.2
27.155	3.28120	459	21.4	19.2
27.165	3.28001	467	21.6	19.2
27.175	3.27881	445	21.1	19.2
27.185	3.27762	493	22.2	19.2
27.196	3.27642	467	21.6	19.2
27.206	3.27523	466	21.6	19.2

27.216	3.27404	451	21.2	19.2
27.226	3.27284	471	21.7	19.2
27.236	3.27165	467	21.6	19.2
27.246	3.27046	445	21.1	19.2
27.256	3.26927	464	21.5	19.2
27.266	3.26809	456	21.4	19.2
27.276	3.26690	529	23.0	19.2
27.286	3.26571	463	21.5	19.2
27.297	3.26453	474	21.8	19.2
27.307	3.26334	472	21.7	19.2
27.317	3.26216	463	21.5	19.2
27.327	3.26098	459	21.4	19.2
27.337	3.25980	470	21.7	19.2
27.347	3.25861	462	21.5	19.2
27.357	3.25743	486	22.0	19.2
27.367	3.25626	434	20.8	19.2
27.377	3.25508	452	21.3	19.2
27.387	3.25390	483	22.0	19.2
27.398	3.25272	468	21.6	19.2
27.408	3.25155	489	22.1	19.2
27.418	3.25037	503	22.4	19.2
27.428	3.24920	467	21.6	19.2
27.438	3.24803	439	21.0	19.2
27.448	3.24685	440	21.0	19.2
27.458	3.24568	450	21.2	19.2
27.468	3.24451	443	21.0	19.2
27.478	3.24334	421	20.5	19.2
27.488	3.24217	439	21.0	19.2
27.498	3.24101	445	21.1	19.2
27.509	3.23984	441	21.0	19.2
27.519	3.23867	464	21.5	19.2
27.529	3.23751	462	21.5	19.2
27.539	3.23634	447	21.1	19.2
27.549	3.23518	456	21.4	19.2
27.559	3.23402	455	21.3	19.2
27.569	3.23286	451	21.2	19.2
27.579	3.23169	411	20.3	19.2
27.589	3.23053	449	21.2	19.2
27.599	3.22938	438	20.9	19.2
27.610	3.22822	478	21.9	19.2
27.620	3.22706	438	20.9	19.2
27.630	3.22590	439	21.0	19.2
27.640	3.22475	437	20.9	19.2
27.650	3.22359	456	21.4	19.2
27.660	3.22244	404	20.1	19.2
27.670	3.22128	430	20.7	19.2
27.680	3.22013	424	20.6	19.2
27.690	3.21898	394	19.8	19.2
27.700	3.21783	457	21.4	19.2
27.711	3.21668	421	20.5	19.2

27.721	3.21553	413	20.3	19.2
27.731	3.21438	402	20.0	19.2
27.741	3.21324	458	21.4	19.2
27.751	3.21209	408	20.2	19.2
27.761	3.21094	478	21.9	19.2
27.771	3.20980	441	21.0	19.2
27.781	3.20866	392	19.8	19.2
27.791	3.20751	423	20.6	19.2
27.801	3.20637	425	20.6	19.2
27.812	3.20523	446	21.1	19.2
27.822	3.20409	413	20.3	19.2
27.832	3.20295	452	21.3	19.2
27.842	3.20181	458	21.4	19.2
27.852	3.20067	408	20.2	19.2
27.862	3.19953	461	21.5	19.2
27.872	3.19840	441	21.0	19.2
27.882	3.19726	409	20.2	19.2
27.892	3.19613	412	20.3	19.2
27.902	3.19499	433	20.8	19.2
27.913	3.19386	416	20.4	19.2
27.923	3.19273	405	20.1	19.2
27.933	3.19160	425	20.6	19.2
27.943	3.19047	404	20.1	19.2
27.953	3.18934	461	21.5	19.2
27.963	3.18821	422	20.5	19.2
27.973	3.18708	407	20.2	19.2
27.983	3.18595	419	20.5	19.2
27.993	3.18483	439	21.0	19.2
28.003	3.18370	475	21.8	19.2
28.014	3.18258	407	20.2	19.2
28.024	3.18145	417	20.4	19.2
28.034	3.18033	428	20.7	19.2
28.044	3.17921	414	20.3	19.2
28.054	3.17808	422	20.5	19.2
28.064	3.17696	413	20.3	19.2
28.074	3.17584	380	19.5	19.2
28.084	3.17472	423	20.6	19.2
28.094	3.17361	378	19.4	19.2
28.104	3.17249	405	20.1	19.2
28.115	3.17137	439	21.0	19.2
28.125	3.17026	426	20.6	19.2
28.135	3.16914	415	20.4	19.2
28.145	3.16803	419	20.5	19.2
28.155	3.16691	381	19.5	19.2
28.165	3.16580	426	20.6	19.2
28.175	3.16469	415	20.4	19.2
28.185	3.16358	396	19.9	19.2
28.195	3.16247	422	20.5	19.2
28.205	3.16136	389	19.7	19.2
28.216	3.16025	399	20.0	19.2

28.226	3.15914	402	20.0	19.2
28.236	3.15804	425	20.6	19.2
28.246	3.15693	427	20.7	19.2
28.256	3.15582	418	20.4	19.2
28.266	3.15472	435	20.9	19.2
28.276	3.15362	398	19.9	19.2
28.286	3.15251	387	19.7	19.2
28.296	3.15141	419	20.5	19.2
28.306	3.15031	415	20.4	19.2
28.317	3.14921	413	20.3	19.2
28.327	3.14811	438	20.9	19.2
28.337	3.14701	416	20.4	19.2
28.347	3.14591	437	20.9	19.2
28.357	3.14481	414	20.3	19.2
28.367	3.14372	399	20.0	19.2
28.377	3.14262	404	20.1	19.2
28.387	3.14153	411	20.3	19.2
28.397	3.14043	382	19.5	19.2
28.407	3.13934	408	20.2	19.2
28.418	3.13825	405	20.1	19.2
28.428	3.13715	396	19.9	19.2
28.438	3.13606	370	19.2	19.2
28.448	3.13497	411	20.3	19.2
28.458	3.13388	398	19.9	19.2
28.468	3.13279	409	20.2	19.2
28.478	3.13171	438	20.9	19.2
28.488	3.13062	414	20.3	19.2
28.498	3.12953	400	20.0	19.2
28.508	3.12845	393	19.8	19.2
28.519	3.12736	413	20.3	19.2
28.529	3.12628	424	20.6	19.2
28.539	3.12519	406	20.1	19.2
28.549	3.12411	356	18.9	19.2
28.559	3.12303	396	19.9	19.2
28.569	3.12195	375	19.4	19.2
28.579	3.12087	420	20.5	19.2
28.589	3.11979	403	20.1	19.2
28.599	3.11871	414	20.3	19.2
28.609	3.11763	424	20.6	19.2
28.620	3.11655	416	20.4	19.2
28.630	3.11548	391	19.8	19.2
28.640	3.11440	432	20.8	19.2
28.650	3.11333	384	19.6	19.2
28.660	3.11225	451	21.2	19.2
28.670	3.11118	355	18.8	19.2
28.680	3.11011	400	20.0	19.2
28.690	3.10904	383	19.6	19.2
28.700	3.10796	407	20.2	19.2
28.710	3.10689	418	20.4	19.2
28.721	3.10582	398	19.9	19.2

28.731	3.10476	410	20.2	19.2
28.741	3.10369	378	19.4	19.2
28.751	3.10262	394	19.8	19.2
28.761	3.10155	437	20.9	19.2
28.771	3.10049	422	20.5	19.2
28.781	3.09942	394	19.8	19.2
28.791	3.09836	399	20.0	19.2
28.801	3.09730	376	19.4	19.2
28.811	3.09623	408	20.2	19.2
28.821	3.09517	388	19.7	19.2
28.832	3.09411	408	20.2	19.2
28.842	3.09305	399	20.0	19.2
28.852	3.09199	428	20.7	19.2
28.862	3.09093	420	20.5	19.2
28.872	3.08987	409	20.2	19.2
28.882	3.08881	421	20.5	19.2
28.892	3.08776	412	20.3	19.2
28.902	3.08670	401	20.0	19.2
28.912	3.08565	407	20.2	19.2
28.922	3.08459	392	19.8	19.2
28.933	3.08354	401	20.0	19.2
28.943	3.08249	390	19.7	19.2
28.953	3.08143	422	20.5	19.2
28.963	3.08038	389	19.7	19.2
28.973	3.07933	416	20.4	19.2
28.983	3.07828	441	21.0	19.2
28.993	3.07723	337	18.4	19.2
29.003	3.07618	392	19.8	19.2
29.013	3.07514	411	20.3	19.2
29.023	3.07409	411	20.3	19.2
29.034	3.07304	393	19.8	19.2
29.044	3.07200	400	20.0	19.2
29.054	3.07095	406	20.1	19.2
29.064	3.06991	379	19.5	19.2
29.074	3.06886	409	20.2	19.2
29.084	3.06782	393	19.8	19.2
29.094	3.06678	366	19.1	19.2
29.104	3.06574	394	19.8	19.2
29.114	3.06470	403	20.1	19.2
29.124	3.06366	384	19.6	19.2
29.135	3.06262	372	19.3	19.2
29.145	3.06158	395	19.9	19.2
29.155	3.06054	397	19.9	19.2
29.165	3.05951	388	19.7	19.2
29.175	3.05847	415	20.4	19.2
29.185	3.05743	417	20.4	19.2
29.195	3.05640	383	19.6	19.2
29.205	3.05537	434	20.8	19.2
29.215	3.05433	382	19.5	19.2
29.225	3.05330	378	19.4	19.2

29.236	3.05227	386	19.6	19.2
29.246	3.05124	396	19.9	19.2
29.256	3.05021	395	19.9	19.2
29.266	3.04918	396	19.9	19.2
29.276	3.04815	389	19.7	19.2
29.286	3.04712	389	19.7	19.2
29.296	3.04609	382	19.5	19.2
29.306	3.04507	388	19.7	19.2
29.316	3.04404	352	18.8	19.2
29.326	3.04301	355	18.8	19.2
29.337	3.04199	376	19.4	19.2
29.347	3.04097	385	19.6	19.2
29.357	3.03994	394	19.8	19.2
29.367	3.03892	404	20.1	19.2
29.377	3.03790	380	19.5	19.2
29.387	3.03688	380	19.5	19.2
29.397	3.03586	349	18.7	19.2
29.407	3.03484	372	19.3	19.2
29.417	3.03382	400	20.0	19.2
29.427	3.03280	381	19.5	19.2
29.438	3.03178	424	20.6	19.2
29.448	3.03077	374	19.3	19.2
29.458	3.02975	346	18.6	19.2
29.468	3.02874	378	19.4	19.2
29.478	3.02772	370	19.2	19.2
29.488	3.02671	397	19.9	19.2
29.498	3.02569	371	19.3	19.2
29.508	3.02468	398	19.9	19.2
29.518	3.02367	370	19.2	19.2
29.528	3.02266	393	19.8	19.2
29.539	3.02165	421	20.5	19.2
29.549	3.02064	383	19.6	19.2
29.559	3.01963	417	20.4	19.2
29.569	3.01862	379	19.5	19.2
29.579	3.01761	379	19.5	19.2
29.589	3.01661	368	19.2	19.2
29.599	3.01560	367	19.2	19.2
29.609	3.01459	385	19.6	19.2
29.619	3.01359	384	19.6	19.2
29.629	3.01258	411	20.3	19.2
29.640	3.01158	389	19.7	19.2
29.650	3.01058	371	19.3	19.2
29.660	3.00958	391	19.8	19.2
29.670	3.00857	360	19.0	19.2
29.680	3.00757	384	19.6	19.2
29.690	3.00657	382	19.5	19.2
29.700	3.00557	414	20.3	19.2
29.710	3.00458	357	18.9	19.2
29.720	3.00358	391	19.8	19.2
29.730	3.00258	395	19.9	19.2

29.741	3.00158	392	19.8	19.2
29.751	3.00059	378	19.4	19.2
29.761	2.99959	409	20.2	19.2
29.771	2.99860	373	19.3	19.2
29.781	2.99760	405	20.1	19.2
29.791	2.99661	377	19.4	19.2
29.801	2.99562	398	19.9	19.2
29.811	2.99463	384	19.6	19.2
29.821	2.99364	395	19.9	19.2
29.831	2.99264	381	19.5	19.2
29.842	2.99166	379	19.5	19.2
29.852	2.99067	342	18.5	19.2
29.862	2.98968	383	19.6	19.2
29.872	2.98869	423	20.6	19.2
29.882	2.98770	401	20.0	19.2
29.892	2.98672	370	19.2	19.2
29.902	2.98573	376	19.4	19.2
29.912	2.98475	344	18.5	19.2
29.922	2.98376	409	20.2	19.2
29.932	2.98278	383	19.6	19.2
29.943	2.98179	418	20.4	19.2
29.953	2.98081	395	19.9	19.2
29.963	2.97983	375	19.4	19.2
29.973	2.97885	365	19.1	19.2
29.983	2.97787	382	19.5	19.2
29.993	2.97689	357	18.9	19.2
30.003	2.97591	396	19.9	19.2
30.013	2.97493	373	19.3	19.2
30.023	2.97395	374	19.3	19.2
30.033	2.97298	370	19.2	19.2
30.044	2.97200	407	20.2	19.2
30.054	2.97102	377	19.4	19.2
30.064	2.97005	371	19.3	19.2
30.074	2.96908	364	19.1	19.2
30.084	2.96810	400	20.0	19.2
30.094	2.96713	377	19.4	19.2
30.104	2.96616	370	19.2	19.2
30.114	2.96518	353	18.8	19.2
30.124	2.96421	344	18.5	19.2
30.134	2.96324	342	18.5	19.2
30.144	2.96227	334	18.3	19.2
30.155	2.96130	376	19.4	19.2
30.165	2.96034	344	18.5	19.2
30.175	2.95937	360	19.0	19.2
30.185	2.95840	338	18.4	19.2
30.195	2.95743	380	19.5	19.2
30.205	2.95647	344	18.5	19.2
30.215	2.95550	407	20.2	19.2
30.225	2.95454	384	19.6	19.2
30.235	2.95357	351	18.7	19.2

30.245	2.95261	340	18.4	19.2
30.256	2.95165	372	19.3	19.2
30.266	2.95069	366	19.1	19.2
30.276	2.94973	374	19.3	19.2
30.286	2.94876	350	18.7	19.2
30.296	2.94780	359	18.9	19.2
30.306	2.94685	355	18.8	19.2
30.316	2.94589	374	19.3	19.2
30.326	2.94493	357	18.9	19.2
30.336	2.94397	348	18.7	19.2
30.346	2.94301	371	19.3	19.2
30.357	2.94206	384	19.6	19.2
30.367	2.94110	336	18.3	19.2
30.377	2.94015	367	19.2	19.2
30.387	2.93919	353	18.8	19.2
30.397	2.93824	386	19.6	19.2
30.407	2.93729	364	19.1	19.2
30.417	2.93634	360	19.0	19.2
30.427	2.93538	344	18.5	19.2
30.437	2.93443	402	20.0	19.2
30.447	2.93348	342	18.5	19.2
30.458	2.93253	357	18.9	19.2
30.468	2.93158	360	19.0	19.2
30.478	2.93064	377	19.4	19.2
30.488	2.92969	360	19.0	19.2
30.498	2.92874	328	18.1	19.2
30.508	2.92779	395	19.9	19.2
30.518	2.92685	332	18.2	19.2
30.528	2.92590	373	19.3	19.2
30.538	2.92496	400	20.0	19.2
30.548	2.92401	392	19.8	19.2
30.559	2.92307	338	18.4	19.2
30.569	2.92213	375	19.4	19.2
30.579	2.92119	327	18.1	19.2
30.589	2.92024	354	18.8	19.2
30.599	2.91930	368	19.2	19.2
30.609	2.91836	331	18.2	19.2
30.619	2.91742	321	17.9	19.2
30.629	2.91649	373	19.3	19.2
30.639	2.91555	356	18.9	19.2
30.649	2.91461	354	18.8	19.2
30.660	2.91367	376	19.4	19.2
30.670	2.91274	361	19.0	19.2
30.680	2.91180	362	19.0	19.2
30.690	2.91086	359	18.9	19.2
30.700	2.90993	338	18.4	19.2
30.710	2.90900	368	19.2	19.2
30.720	2.90806	377	19.4	19.2
30.730	2.90713	381	19.5	19.2
30.740	2.90620	361	19.0	19.2

30.750	2.90527	360	19.0	19.2
30.761	2.90434	356	18.9	19.2
30.771	2.90341	375	19.4	19.2
30.781	2.90248	377	19.4	19.2
30.791	2.90155	345	18.6	19.2
30.801	2.90062	331	18.2	19.2
30.811	2.89969	386	19.6	19.2
30.821	2.89876	391	19.8	19.2
30.831	2.89784	351	18.7	19.2
30.841	2.89691	362	19.0	19.2
30.851	2.89599	406	20.1	19.2
30.862	2.89506	374	19.3	19.2
30.872	2.89414	341	18.5	19.2
30.882	2.89321	354	18.8	19.2
30.892	2.89229	383	19.6	19.2
30.902	2.89137	356	18.9	19.2
30.912	2.89045	376	19.4	19.2
30.922	2.88953	331	18.2	19.2
30.932	2.88861	379	19.5	19.2
30.942	2.88769	342	18.5	19.2
30.952	2.88677	368	19.2	19.2
30.963	2.88585	371	19.3	19.2
30.973	2.88493	369	19.2	19.2
30.983	2.88401	340	18.4	19.2
30.993	2.88310	338	18.4	19.2
31.003	2.88218	380	19.5	19.2
31.013	2.88126	399	20.0	19.2
31.023	2.88035	380	19.5	19.2
31.033	2.87943	351	18.7	19.2
31.043	2.87852	391	19.8	19.2
31.053	2.87761	358	18.9	19.2
31.064	2.87670	364	19.1	19.2
31.074	2.87578	378	19.4	19.2
31.084	2.87487	353	18.8	19.2
31.094	2.87396	357	18.9	19.2
31.104	2.87305	355	18.8	19.2
31.114	2.87214	351	18.7	19.2
31.124	2.87123	349	18.7	19.2
31.134	2.87032	357	18.9	19.2
31.144	2.86942	328	18.1	19.2
31.154	2.86851	372	19.3	19.2
31.165	2.86760	355	18.8	19.2
31.175	2.86670	355	18.8	19.2
31.185	2.86579	376	19.4	19.2
31.195	2.86489	317	17.8	19.2
31.205	2.86398	390	19.7	19.2
31.215	2.86308	359	18.9	19.2
31.225	2.86218	385	19.6	19.2
31.235	2.86127	360	19.0	19.2
31.245	2.86037	371	19.3	19.2

31.255	2.85947	384	19.6	19.2
31.266	2.85857	343	18.5	19.2
31.276	2.85767	362	19.0	19.2
31.286	2.85677	344	18.5	19.2
31.296	2.85587	353	18.8	19.2
31.306	2.85497	333	18.2	19.2
31.316	2.85408	317	17.8	19.2
31.326	2.85318	357	18.9	19.2
31.336	2.85228	344	18.5	19.2
31.346	2.85139	373	19.3	19.2
31.356	2.85049	317	17.8	19.2
31.366	2.84960	332	18.2	19.2
31.377	2.84870	333	18.2	19.2
31.387	2.84781	349	18.7	19.2
31.397	2.84692	340	18.4	19.2
31.407	2.84602	354	18.8	19.2
31.417	2.84513	355	18.8	19.2
31.427	2.84424	328	18.1	19.2
31.437	2.84335	361	19.0	19.2
31.447	2.84246	350	18.7	19.2
31.457	2.84157	367	19.2	19.2
31.467	2.84068	334	18.3	19.2
31.478	2.83979	352	18.8	19.2
31.488	2.83890	349	18.7	19.2
31.498	2.83802	351	18.7	19.2
31.508	2.83713	352	18.8	19.2
31.518	2.83624	349	18.7	19.2
31.528	2.83536	320	17.9	19.2
31.538	2.83447	345	18.6	19.2
31.548	2.83359	338	18.4	19.2
31.558	2.83271	312	17.7	19.2
31.568	2.83182	340	18.4	19.2
31.579	2.83094	370	19.2	19.2
31.589	2.83006	363	19.1	19.2
31.599	2.82918	332	18.2	19.2
31.609	2.82830	385	19.6	19.2
31.619	2.82742	349	18.7	19.2
31.629	2.82654	335	18.3	19.2
31.639	2.82566	357	18.9	19.2
31.649	2.82478	358	18.9	19.2
31.659	2.82390	339	18.4	19.2
31.669	2.82302	355	18.8	19.2
31.680	2.82215	359	18.9	19.2
31.690	2.82127	361	19.0	19.2
31.700	2.82039	337	18.4	19.2
31.710	2.81952	346	18.6	19.2
31.720	2.81864	350	18.7	19.2
31.730	2.81777	383	19.6	19.2
31.740	2.81690	338	18.4	19.2
31.750	2.81602	345	18.6	19.2

31.760	2.81515	349	18.7	19.2
31.770	2.81428	347	18.6	19.2
31.781	2.81341	340	18.4	19.2
31.791	2.81254	345	18.6	19.2
31.801	2.81167	335	18.3	19.2
31.811	2.81080	336	18.3	19.2
31.821	2.80993	349	18.7	19.2
31.831	2.80906	372	19.3	19.2
31.841	2.80819	345	18.6	19.2
31.851	2.80732	382	19.5	19.2
31.861	2.80646	344	18.5	19.2
31.871	2.80559	341	18.5	19.2
31.882	2.80473	341	18.5	19.2
31.892	2.80386	347	18.6	19.2
31.902	2.80300	365	19.1	19.2
31.912	2.80213	370	19.2	19.2
31.922	2.80127	361	19.0	19.2
31.932	2.80040	357	18.9	19.2
31.942	2.79954	321	17.9	19.2
31.952	2.79868	308	17.5	19.2
31.962	2.79782	343	18.5	19.2
31.972	2.79696	328	18.1	19.2
31.983	2.79610	353	18.8	19.2
31.993	2.79524	350	18.7	19.2
32.003	2.79438	354	18.8	19.2
32.013	2.79352	341	18.5	19.2
32.023	2.79266	319	17.9	19.2
32.033	2.79181	342	18.5	19.2
32.043	2.79095	358	18.9	19.2
32.053	2.79009	313	17.7	19.2
32.063	2.78924	357	18.9	19.2
32.073	2.78838	362	19.0	19.2
32.084	2.78753	354	18.8	19.2
32.094	2.78667	348	18.7	19.2
32.104	2.78582	327	18.1	19.2
32.114	2.78497	354	18.8	19.2
32.124	2.78411	368	19.2	19.2
32.134	2.78326	370	19.2	19.2
32.144	2.78241	328	18.1	19.2
32.154	2.78156	324	18.0	19.2
32.164	2.78071	356	18.9	19.2
32.174	2.77986	360	19.0	19.2
32.185	2.77901	330	18.2	19.2
32.195	2.77816	335	18.3	19.2
32.205	2.77731	335	18.3	19.2
32.215	2.77647	334	18.3	19.2
32.225	2.77562	323	18.0	19.2
32.235	2.77477	319	17.9	19.2
32.245	2.77393	345	18.6	19.2
32.255	2.77308	300	17.3	19.2

32.265	2.77224	326	18.1	19.2
32.275	2.77139	362	19.0	19.2
32.286	2.77055	361	19.0	19.2
32.296	2.76970	333	18.2	19.2
32.306	2.76886	319	17.9	19.2
32.316	2.76802	361	19.0	19.2
32.326	2.76718	319	17.9	19.2
32.336	2.76634	338	18.4	19.2
32.346	2.76550	321	17.9	19.2
32.356	2.76466	331	18.2	19.2
32.366	2.76382	300	17.3	19.2
32.376	2.76298	342	18.5	19.2
32.387	2.76214	365	19.1	19.2
32.397	2.76130	320	17.9	19.2
32.407	2.76046	335	18.3	19.2
32.417	2.75963	328	18.1	19.2
32.427	2.75879	347	18.6	19.2
32.437	2.75795	358	18.9	19.2
32.447	2.75712	371	19.3	19.2
32.457	2.75628	323	18.0	19.2
32.467	2.75545	326	18.1	19.2
32.477	2.75462	332	18.2	19.2
32.488	2.75378	327	18.1	19.2
32.498	2.75295	332	18.2	19.2
32.508	2.75212	326	18.1	19.2
32.518	2.75129	337	18.4	19.2
32.528	2.75045	325	18.0	19.2
32.538	2.74962	311	17.6	19.2
32.548	2.74879	353	18.8	19.2
32.558	2.74796	326	18.1	19.2
32.568	2.74713	391	19.8	19.2
32.578	2.74631	316	17.8	19.2
32.589	2.74548	336	18.3	19.2
32.599	2.74465	334	18.3	19.2
32.609	2.74382	376	19.4	19.2
32.619	2.74300	340	18.4	19.2
32.629	2.74217	341	18.5	19.2
32.639	2.74135	331	18.2	19.2
32.649	2.74052	319	17.9	19.2
32.659	2.73970	316	17.8	19.2
32.669	2.73887	369	19.2	19.2
32.679	2.73805	326	18.1	19.2
32.689	2.73723	351	18.7	19.2
32.700	2.73640	341	18.5	19.2
32.710	2.73558	318	17.8	19.2
32.720	2.73476	305	17.5	19.2
32.730	2.73394	324	18.0	19.2
32.740	2.73312	310	17.6	19.2
32.750	2.73230	318	17.8	19.2
32.760	2.73148	336	18.3	19.2

32.770	2.73066	347	18.6	19.2
32.780	2.72984	342	18.5	19.2
32.790	2.72903	362	19.0	19.2
32.801	2.72821	321	17.9	19.2
32.811	2.72739	337	18.4	19.2
32.821	2.72658	351	18.7	19.2
32.831	2.72576	295	17.2	19.2
32.841	2.72495	314	17.7	19.2
32.851	2.72413	353	18.8	19.2
32.861	2.72332	316	17.8	19.2
32.871	2.72250	301	17.3	19.2
32.881	2.72169	354	18.8	19.2
32.891	2.72088	366	19.1	19.2
32.902	2.72007	346	18.6	19.2
32.912	2.71925	314	17.7	19.2
32.922	2.71844	374	19.3	19.2
32.932	2.71763	334	18.3	19.2
32.942	2.71682	327	18.1	19.2
32.952	2.71601	331	18.2	19.2
32.962	2.71520	340	18.4	19.2
32.972	2.71439	353	18.8	19.2
32.982	2.71359	339	18.4	19.2
32.992	2.71278	323	18.0	19.2
33.003	2.71197	324	18.0	19.2
33.013	2.71117	333	18.2	19.2
33.023	2.71036	311	17.6	19.2
33.033	2.70955	318	17.8	19.2
33.043	2.70875	318	17.8	19.2
33.053	2.70794	286	16.9	19.2
33.063	2.70714	324	18.0	19.2
33.073	2.70634	342	18.5	19.2
33.083	2.70553	341	18.5	19.2
33.093	2.70473	336	18.3	19.2
33.104	2.70393	352	18.8	19.2
33.114	2.70313	322	17.9	19.2
33.124	2.70233	331	18.2	19.2
33.134	2.70153	351	18.7	19.2
33.144	2.70072	342	18.5	19.2
33.154	2.69993	335	18.3	19.2
33.164	2.69913	325	18.0	19.2
33.174	2.69833	294	17.1	19.2
33.184	2.69753	320	17.9	19.2
33.194	2.69673	312	17.7	19.2
33.205	2.69593	343	18.5	19.2
33.215	2.69514	349	18.7	19.2
33.225	2.69434	335	18.3	19.2
33.235	2.69355	299	17.3	19.2
33.245	2.69275	336	18.3	19.2
33.255	2.69196	331	18.2	19.2
33.265	2.69116	363	19.1	19.2

33.275	2.69037	337	18.4	19.2
33.285	2.68958	324	18.0	19.2
33.295	2.68878	336	18.3	19.2
33.306	2.68799	342	18.5	19.2
33.316	2.68720	325	18.0	19.2
33.326	2.68641	318	17.8	19.2
33.336	2.68562	330	18.2	19.2
33.346	2.68483	362	19.0	19.2
33.356	2.68404	324	18.0	19.2
33.366	2.68325	327	18.1	19.2
33.376	2.68246	284	16.9	19.2
33.386	2.68167	337	18.4	19.2
33.396	2.68088	299	17.3	19.2
33.407	2.68009	342	18.5	19.2
33.417	2.67931	319	17.9	19.2
33.427	2.67852	343	18.5	19.2
33.437	2.67774	310	17.6	19.2
33.447	2.67695	316	17.8	19.2
33.457	2.67616	293	17.1	19.2
33.467	2.67538	299	17.3	19.2
33.477	2.67460	318	17.8	19.2
33.487	2.67381	315	17.7	19.2
33.497	2.67303	285	16.9	19.2
33.508	2.67225	325	18.0	19.2
33.518	2.67147	316	17.8	19.2
33.528	2.67068	321	17.9	19.2
33.538	2.66990	327	18.1	19.2
33.548	2.66912	333	18.2	19.2
33.558	2.66834	347	18.6	19.2
33.568	2.66756	338	18.4	19.2
33.578	2.66678	307	17.5	19.2
33.588	2.66600	320	17.9	19.2
33.598	2.66523	322	17.9	19.2
33.609	2.66445	293	17.1	19.2
33.619	2.66367	348	18.7	19.2
33.629	2.66289	309	17.6	19.2
33.639	2.66212	326	18.1	19.2
33.649	2.66134	294	17.1	19.2
33.659	2.66057	306	17.5	19.2
33.669	2.65979	353	18.8	19.2
33.679	2.65902	327	18.1	19.2
33.689	2.65824	304	17.4	19.2
33.699	2.65747	332	18.2	19.2
33.710	2.65670	326	18.1	19.2
33.720	2.65592	282	16.8	19.2
33.730	2.65515	323	18.0	19.2
33.740	2.65438	294	17.1	19.2
33.750	2.65361	365	19.1	19.2
33.760	2.65284	305	17.5	19.2
33.770	2.65207	304	17.4	19.2

33.780	2.65130	311	17.6	19.2
33.790	2.65053	308	17.5	19.2
33.800	2.64976	325	18.0	19.2
33.811	2.64899	304	17.4	19.2
33.821	2.64822	296	17.2	19.2
33.831	2.64746	301	17.3	19.2
33.841	2.64669	304	17.4	19.2
33.851	2.64592	305	17.5	19.2
33.861	2.64516	328	18.1	19.2
33.871	2.64439	334	18.3	19.2
33.881	2.64363	305	17.5	19.2
33.891	2.64286	302	17.4	19.2
33.901	2.64210	291	17.1	19.2
33.912	2.64133	316	17.8	19.2
33.922	2.64057	315	17.7	19.2
33.932	2.63981	295	17.2	19.2
33.942	2.63904	322	17.9	19.2
33.952	2.63828	333	18.2	19.2
33.962	2.63752	319	17.9	19.2
33.972	2.63676	294	17.1	19.2
33.982	2.63600	333	18.2	19.2
33.992	2.63524	319	17.9	19.2
34.002	2.63448	343	18.5	19.2
34.012	2.63372	311	17.6	19.2
34.023	2.63296	307	17.5	19.2
34.033	2.63220	353	18.8	19.2
34.043	2.63145	297	17.2	19.2
34.053	2.63069	288	17.0	19.2
34.063	2.62993	327	18.1	19.2
34.073	2.62917	308	17.5	19.2
34.083	2.62842	304	17.4	19.2
34.093	2.62766	335	18.3	19.2
34.103	2.62691	315	17.7	19.2
34.113	2.62615	335	18.3	19.2
34.124	2.62540	303	17.4	19.2
34.134	2.62465	312	17.7	19.2
34.144	2.62389	329	18.1	19.2
34.154	2.62314	285	16.9	19.2
34.164	2.62239	288	17.0	19.2
34.174	2.62164	297	17.2	19.2
34.184	2.62088	271	16.5	19.2
34.194	2.62013	292	17.1	19.2
34.204	2.61938	315	17.7	19.2
34.214	2.61863	327	18.1	19.2
34.225	2.61788	307	17.5	19.2
34.235	2.61713	306	17.5	19.2
34.245	2.61639	296	17.2	19.2
34.255	2.61564	346	18.6	19.2
34.265	2.61489	309	17.6	19.2
34.275	2.61414	303	17.4	19.2

34.285	2.61339	305	17.5	19.2
34.295	2.61265	314	17.7	19.2
34.305	2.61190	288	17.0	19.2
34.315	2.61116	277	16.6	19.2
34.326	2.61041	304	17.4	19.2
34.336	2.60967	301	17.3	19.2
34.346	2.60892	329	18.1	19.2
34.356	2.60818	317	17.8	19.2
34.366	2.60744	282	16.8	19.2
34.376	2.60669	312	17.7	19.2
34.386	2.60595	315	17.7	19.2
34.396	2.60521	272	16.5	19.2
34.406	2.60447	281	16.8	19.2
34.416	2.60373	319	17.9	19.2
34.427	2.60298	317	17.8	19.2
34.437	2.60224	296	17.2	19.2
34.447	2.60150	310	17.6	19.2
34.457	2.60077	295	17.2	19.2
34.467	2.60003	302	17.4	19.2
34.477	2.59929	306	17.5	19.2
34.487	2.59855	294	17.1	19.2
34.497	2.59781	284	16.9	19.2
34.507	2.59707	301	17.3	19.2
34.517	2.59634	295	17.2	19.2
34.528	2.59560	288	17.0	19.2
34.538	2.59487	313	17.7	19.2
34.548	2.59413	299	17.3	19.2
34.558	2.59340	291	17.1	19.2
34.568	2.59266	313	17.7	19.2
34.578	2.59193	280	16.7	19.2
34.588	2.59119	281	16.8	19.2
34.598	2.59046	279	16.7	19.2
34.608	2.58973	310	17.6	19.2
34.618	2.58899	282	16.8	19.2
34.629	2.58826	282	16.8	19.2
34.639	2.58753	299	17.3	19.2
34.649	2.58680	300	17.3	19.2
34.659	2.58607	298	17.3	19.2
34.669	2.58534	292	17.1	19.2
34.679	2.58461	321	17.9	19.2
34.689	2.58388	313	17.7	19.2
34.699	2.58315	306	17.5	19.2
34.709	2.58242	279	16.7	19.2
34.719	2.58169	285	16.9	19.2
34.730	2.58097	275	16.6	19.2
34.740	2.58024	278	16.7	19.2
34.750	2.57951	291	17.1	19.2
34.760	2.57879	248	15.7	19.2
34.770	2.57806	305	17.5	19.2
34.780	2.57734	285	16.9	19.2

34.790	2.57661	305	17.5	19.2
34.800	2.57589	287	16.9	19.2
34.810	2.57516	298	17.3	19.2
34.820	2.57444	304	17.4	19.2
34.831	2.57371	292	17.1	19.2
34.841	2.57299	292	17.1	19.2
34.851	2.57227	272	16.5	19.2
34.861	2.57155	278	16.7	19.2
34.871	2.57083	280	16.7	19.2
34.881	2.57010	282	16.8	19.2
34.891	2.56938	285	16.9	19.2
34.901	2.56866	259	16.1	19.2
34.911	2.56794	284	16.9	19.2
34.921	2.56722	292	17.1	19.2
34.932	2.56650	307	17.5	19.2
34.942	2.56579	277	16.6	19.2
34.952	2.56507	298	17.3	19.2
34.962	2.56435	293	17.1	19.2
34.972	2.56363	298	17.3	19.2
34.982	2.56292	280	16.7	19.2
34.992	2.56220	269	16.4	19.2
35.002	2.56148	284	16.9	19.2
35.012	2.56077	317	17.8	19.2
35.022	2.56005	329	18.1	19.2
35.033	2.55934	273	16.5	19.2
35.043	2.55862	295	17.2	19.2
35.053	2.55791	345	18.6	19.2
35.063	2.55719	294	17.1	19.2
35.073	2.55648	281	16.8	19.2
35.083	2.55577	271	16.5	19.2
35.093	2.55506	303	17.4	19.2
35.103	2.55434	272	16.5	19.2
35.113	2.55363	282	16.8	19.2
35.123	2.55292	301	17.3	19.2
35.134	2.55221	278	16.7	19.2
35.144	2.55150	273	16.5	19.2
35.154	2.55079	280	16.7	19.2
35.164	2.55008	290	17.0	19.2
35.174	2.54937	297	17.2	19.2
35.184	2.54866	273	16.5	19.2
35.194	2.54795	319	17.9	19.2
35.204	2.54725	295	17.2	19.2
35.214	2.54654	310	17.6	19.2
35.224	2.54583	308	17.5	19.2
35.235	2.54513	287	16.9	19.2
35.245	2.54442	300	17.3	19.2
35.255	2.54371	309	17.6	19.2
35.265	2.54301	306	17.5	19.2
35.275	2.54230	282	16.8	19.2
35.285	2.54160	285	16.9	19.2

35.295	2.54090	280	16.7	19.2
35.305	2.54019	289	17.0	19.2
35.315	2.53949	307	17.5	19.2
35.325	2.53879	282	16.8	19.2
35.335	2.53808	269	16.4	19.2
35.346	2.53738	289	17.0	19.2
35.356	2.53668	290	17.0	19.2
35.366	2.53598	287	16.9	19.2
35.376	2.53528	288	17.0	19.2
35.386	2.53458	297	17.2	19.2
35.396	2.53388	298	17.3	19.2
35.406	2.53318	284	16.9	19.2
35.416	2.53248	276	16.6	19.2
35.426	2.53178	294	17.1	19.2
35.436	2.53108	264	16.2	19.2
35.447	2.53038	281	16.8	19.2
35.457	2.52969	300	17.3	19.2
35.467	2.52899	264	16.2	19.2
35.477	2.52829	308	17.5	19.2
35.487	2.52759	317	17.8	19.2
35.497	2.52690	296	17.2	19.2
35.507	2.52620	301	17.3	19.2
35.517	2.52551	277	16.6	19.2
35.527	2.52481	261	16.2	19.2
35.537	2.52412	291	17.1	19.2
35.548	2.52342	285	16.9	19.2
35.558	2.52273	282	16.8	19.2
35.568	2.52204	317	17.8	19.2
35.578	2.52135	293	17.1	19.2
35.588	2.52065	275	16.6	19.2
35.598	2.51996	274	16.6	19.2
35.608	2.51927	286	16.9	19.2
35.618	2.51858	290	17.0	19.2
35.628	2.51789	257	16.0	19.2
35.638	2.51720	257	16.0	19.2
35.649	2.51651	310	17.6	19.2
35.659	2.51582	257	16.0	19.2
35.669	2.51513	285	16.9	19.2
35.679	2.51444	269	16.4	19.2
35.689	2.51375	288	17.0	19.2
35.699	2.51306	275	16.6	19.2
35.709	2.51238	273	16.5	19.2
35.719	2.51169	307	17.5	19.2
35.729	2.51100	289	17.0	19.2
35.739	2.51032	267	16.3	19.2
35.750	2.50963	276	16.6	19.2
35.760	2.50894	286	16.9	19.2
35.770	2.50826	263	16.2	19.2
35.780	2.50757	285	16.9	19.2
35.790	2.50689	277	16.6	19.2

35.800	2.50621	297	17.2	19.2
35.810	2.50552	277	16.6	19.2
35.820	2.50484	306	17.5	19.2
35.830	2.50416	252	15.9	19.2
35.840	2.50347	263	16.2	19.2
35.851	2.50279	291	17.1	19.2
35.861	2.50211	269	16.4	19.2
35.871	2.50143	286	16.9	19.2
35.881	2.50075	259	16.1	19.2
35.891	2.50007	286	16.9	19.2
35.901	2.49939	258	16.1	19.2
35.911	2.49871	299	17.3	19.2
35.921	2.49803	297	17.2	19.2
35.931	2.49735	282	16.8	19.2
35.941	2.49667	263	16.2	19.2
35.952	2.49599	258	16.1	19.2
35.962	2.49531	286	16.9	19.2
35.972	2.49464	287	16.9	19.2
35.982	2.49396	253	15.9	19.2
35.992	2.49328	295	17.2	19.2
36.002	2.49261	284	16.9	19.2
36.012	2.49193	265	16.3	19.2
36.022	2.49125	302	17.4	19.2
36.032	2.49058	298	17.3	19.2
36.042	2.48990	287	16.9	19.2
36.053	2.48923	280	16.7	19.2
36.063	2.48856	260	16.1	19.2
36.073	2.48788	279	16.7	19.2
36.083	2.48721	278	16.7	19.2
36.093	2.48654	288	17.0	19.2
36.103	2.48586	246	15.7	19.2
36.113	2.48519	269	16.4	19.2
36.123	2.48452	253	15.9	19.2
36.133	2.48385	277	16.6	19.2
36.143	2.48318	289	17.0	19.2
36.154	2.48251	265	16.3	19.2
36.164	2.48184	262	16.2	19.2
36.174	2.48117	266	16.3	19.2
36.184	2.48050	259	16.1	19.2
36.194	2.47983	269	16.4	19.2
36.204	2.47916	274	16.6	19.2
36.214	2.47849	279	16.7	19.2
36.224	2.47783	273	16.5	19.2
36.234	2.47716	265	16.3	19.2
36.244	2.47649	242	15.6	19.2
36.255	2.47582	261	16.2	19.2
36.265	2.47516	272	16.5	19.2
36.275	2.47449	279	16.7	19.2
36.285	2.47383	265	16.3	19.2
36.295	2.47316	287	16.9	19.2

36.305	2.47250	253	15.9	19.2
36.315	2.47183	279	16.7	19.2
36.325	2.47117	280	16.7	19.2
36.335	2.47050	250	15.8	19.2
36.345	2.46984	278	16.7	19.2
36.356	2.46918	259	16.1	19.2
36.366	2.46852	264	16.2	19.2
36.376	2.46785	249	15.8	19.2
36.386	2.46719	268	16.4	19.2
36.396	2.46653	259	16.1	19.2
36.406	2.46587	225	15.0	19.2
36.416	2.46521	263	16.2	19.2
36.426	2.46455	246	15.7	19.2
36.436	2.46389	231	15.2	19.2
36.446	2.46323	249	15.8	19.2
36.457	2.46257	261	16.2	19.2
36.467	2.46191	230	15.2	19.2
36.477	2.46125	251	15.8	19.2
36.487	2.46059	285	16.9	19.2
36.497	2.45994	267	16.3	19.2
36.507	2.45928	253	15.9	19.2
36.517	2.45862	258	16.1	19.2
36.527	2.45797	252	15.9	19.2
36.537	2.45731	249	15.8	19.2
36.547	2.45665	255	16.0	19.2
36.558	2.45600	272	16.5	19.2
36.568	2.45534	273	16.5	19.2
36.578	2.45469	269	16.4	19.2
36.588	2.45403	252	15.9	19.2
36.598	2.45338	257	16.0	19.2
36.608	2.45273	284	16.9	19.2
36.618	2.45207	254	15.9	19.2
36.628	2.45142	270	16.4	19.2
36.638	2.45077	282	16.8	19.2
36.648	2.45011	262	16.2	19.2
36.658	2.44946	235	15.3	19.2
36.669	2.44881	266	16.3	19.2
36.679	2.44816	271	16.5	19.2
36.689	2.44751	279	16.7	19.2
36.699	2.44686	239	15.5	19.2
36.709	2.44621	253	15.9	19.2
36.719	2.44556	244	15.6	19.2
36.729	2.44491	252	15.9	19.2
36.739	2.44426	258	16.1	19.2
36.749	2.44361	251	15.8	19.2
36.759	2.44296	267	16.3	19.2
36.770	2.44232	243	15.6	19.2
36.780	2.44167	257	16.0	19.2
36.790	2.44102	268	16.4	19.2
36.800	2.44038	236	15.4	19.2

36.810	2.43973	253	15.9	19.2
36.820	2.43908	282	16.8	19.2
36.830	2.43844	241	15.5	19.2
36.840	2.43779	257	16.0	19.2
36.850	2.43715	281	16.8	19.2
36.860	2.43650	242	15.6	19.2
36.871	2.43586	290	17.0	19.2
36.881	2.43522	245	15.7	19.2
36.891	2.43457	255	16.0	19.2
36.901	2.43393	259	16.1	19.2
36.911	2.43329	264	16.2	19.2
36.921	2.43264	260	16.1	19.2
36.931	2.43200	264	16.2	19.2
36.941	2.43136	263	16.2	19.2
36.951	2.43072	246	15.7	19.2
36.961	2.43008	254	15.9	19.2
36.972	2.42944	268	16.4	19.2
36.982	2.42880	286	16.9	19.2
36.992	2.42816	277	16.6	19.2
37.002	2.42752	270	16.4	19.2
37.012	2.42688	244	15.6	19.2
37.022	2.42624	273	16.5	19.2
37.032	2.42560	247	15.7	19.2
37.042	2.42496	258	16.1	19.2
37.052	2.42432	261	16.2	19.2
37.062	2.42369	274	16.6	19.2
37.073	2.42305	275	16.6	19.2
37.083	2.42241	258	16.1	19.2
37.093	2.42178	267	16.3	19.2
37.103	2.42114	235	15.3	19.2
37.113	2.42051	260	16.1	19.2
37.123	2.41987	242	15.6	19.2
37.133	2.41923	237	15.4	19.2
37.143	2.41860	271	16.5	19.2
37.153	2.41797	258	16.1	19.2
37.163	2.41733	237	15.4	19.2
37.174	2.41670	256	16.0	19.2
37.184	2.41607	278	16.7	19.2
37.194	2.41543	283	16.8	19.2
37.204	2.41480	270	16.4	19.2
37.214	2.41417	235	15.3	19.2
37.224	2.41354	250	15.8	19.2
37.234	2.41290	274	16.6	19.2
37.244	2.41227	265	16.3	19.2
37.254	2.41164	275	16.6	19.2
37.264	2.41101	261	16.2	19.2
37.275	2.41038	282	16.8	19.2
37.285	2.40975	231	15.2	19.2
37.295	2.40912	251	15.8	19.2
37.305	2.40849	274	16.6	19.2

37.315	2.40787	231	15.2	19.2
37.325	2.40724	259	16.1	19.2
37.335	2.40661	236	15.4	19.2
37.345	2.40598	249	15.8	19.2
37.355	2.40535	290	17.0	19.2
37.365	2.40473	268	16.4	19.2
37.376	2.40410	259	16.1	19.2
37.386	2.40347	271	16.5	19.2
37.396	2.40285	278	16.7	19.2
37.406	2.40222	278	16.7	19.2
37.416	2.40160	254	15.9	19.2
37.426	2.40097	243	15.6	19.2
37.436	2.40035	255	16.0	19.2
37.446	2.39972	251	15.8	19.2
37.456	2.39910	209	14.5	19.2
37.466	2.39848	259	16.1	19.2
37.477	2.39785	259	16.1	19.2
37.487	2.39723	255	16.0	19.2
37.497	2.39661	252	15.9	19.2
37.507	2.39599	273	16.5	19.2
37.517	2.39537	240	15.5	19.2
37.527	2.39474	230	15.2	19.2
37.537	2.39412	253	15.9	19.2
37.547	2.39350	259	16.1	19.2
37.557	2.39288	283	16.8	19.2
37.567	2.39226	298	17.3	19.2
37.578	2.39164	267	16.3	19.2
37.588	2.39102	251	15.8	19.2
37.598	2.39040	254	15.9	19.2
37.608	2.38978	263	16.2	19.2
37.618	2.38917	254	15.9	19.2
37.628	2.38855	235	15.3	19.2
37.638	2.38793	214	14.6	19.2
37.648	2.38731	266	16.3	19.2
37.658	2.38670	240	15.5	19.2
37.668	2.38608	252	15.9	19.2
37.679	2.38546	257	16.0	19.2
37.689	2.38485	239	15.5	19.2
37.699	2.38423	248	15.7	19.2
37.709	2.38362	226	15.0	19.2
37.719	2.38300	236	15.4	19.2
37.729	2.38239	274	16.6	19.2
37.739	2.38177	273	16.5	19.2
37.749	2.38116	253	15.9	19.2
37.759	2.38054	254	15.9	19.2
37.769	2.37993	229	15.1	19.2
37.780	2.37932	247	15.7	19.2
37.790	2.37871	278	16.7	19.2
37.800	2.37809	281	16.8	19.2
37.810	2.37748	282	16.8	19.2

37.820	2.37687	277	16.6	19.2
37.830	2.37626	238	15.4	19.2
37.840	2.37565	273	16.5	19.2
37.850	2.37504	260	16.1	19.2
37.860	2.37443	246	15.7	19.2
37.870	2.37382	245	15.7	19.2
37.881	2.37321	237	15.4	19.2
37.891	2.37260	249	15.8	19.2
37.901	2.37199	256	16.0	19.2
37.911	2.37138	245	15.7	19.2
37.921	2.37077	239	15.5	19.2
37.931	2.37016	247	15.7	19.2
37.941	2.36956	265	16.3	19.2
37.951	2.36895	272	16.5	19.2
37.961	2.36834	252	15.9	19.2
37.971	2.36773	246	15.7	19.2
37.981	2.36713	249	15.8	19.2
37.992	2.36652	256	16.0	19.2
38.002	2.36592	281	16.8	19.2
38.012	2.36531	250	15.8	19.2
38.022	2.36471	240	15.5	19.2
38.032	2.36410	246	15.7	19.2
38.042	2.36350	245	15.7	19.2
38.052	2.36289	281	16.8	19.2
38.062	2.36229	264	16.2	19.2
38.072	2.36169	205	14.3	19.2
38.082	2.36108	249	15.8	19.2
38.093	2.36048	287	16.9	19.2
38.103	2.35988	223	14.9	19.2
38.113	2.35927	272	16.5	19.2
38.123	2.35867	233	15.3	19.2
38.133	2.35807	271	16.5	19.2
38.143	2.35747	290	17.0	19.2
38.153	2.35687	271	16.5	19.2
38.163	2.35627	235	15.3	19.2
38.173	2.35567	271	16.5	19.2
38.183	2.35507	252	15.9	19.2
38.194	2.35447	225	15.0	19.2
38.204	2.35387	241	15.5	19.2
38.214	2.35327	243	15.6	19.2
38.224	2.35267	243	15.6	19.2
38.234	2.35207	238	15.4	19.2
38.244	2.35148	245	15.7	19.2
38.254	2.35088	240	15.5	19.2
38.264	2.35028	266	16.3	19.2
38.274	2.34969	241	15.5	19.2
38.284	2.34909	275	16.6	19.2
38.295	2.34849	239	15.5	19.2
38.305	2.34790	261	16.2	19.2
38.315	2.34730	251	15.8	19.2

38.325	2.34671	241	15.5	19.2
38.335	2.34611	262	16.2	19.2
38.345	2.34552	237	15.4	19.2
38.355	2.34492	242	15.6	19.2
38.365	2.34433	243	15.6	19.2
38.375	2.34373	254	15.9	19.2
38.385	2.34314	226	15.0	19.2
38.396	2.34255	244	15.6	19.2
38.406	2.34195	245	15.7	19.2
38.416	2.34136	234	15.3	19.2
38.426	2.34077	244	15.6	19.2
38.436	2.34018	244	15.6	19.2
38.446	2.33959	230	15.2	19.2
38.456	2.33900	241	15.5	19.2
38.466	2.33840	261	16.2	19.2
38.476	2.33781	241	15.5	19.2
38.486	2.33722	230	15.2	19.2
38.497	2.33663	245	15.7	19.2
38.507	2.33604	255	16.0	19.2
38.517	2.33545	254	15.9	19.2
38.527	2.33487	218	14.8	19.2
38.537	2.33428	258	16.1	19.2
38.547	2.33369	251	15.8	19.2
38.557	2.33310	230	15.2	19.2
38.567	2.33251	250	15.8	19.2
38.577	2.33193	261	16.2	19.2
38.587	2.33134	241	15.5	19.2
38.598	2.33075	270	16.4	19.2
38.608	2.33017	247	15.7	19.2
38.618	2.32958	255	16.0	19.2
38.628	2.32899	256	16.0	19.2
38.638	2.32841	227	15.1	19.2
38.648	2.32782	228	15.1	19.2
38.658	2.32724	238	15.4	19.2
38.668	2.32665	238	15.4	19.2
38.678	2.32607	247	15.7	19.2
38.688	2.32549	239	15.5	19.2
38.699	2.32490	232	15.2	19.2
38.709	2.32432	269	16.4	19.2
38.719	2.32374	239	15.5	19.2
38.729	2.32315	208	14.4	19.2
38.739	2.32257	255	16.0	19.2
38.749	2.32199	244	15.6	19.2
38.759	2.32141	222	14.9	19.2
38.769	2.32082	225	15.0	19.2
38.779	2.32024	253	15.9	19.2
38.789	2.31966	237	15.4	19.2
38.800	2.31908	244	15.6	19.2
38.810	2.31850	241	15.5	19.2
38.820	2.31792	226	15.0	19.2

38.830	2.31734	245	15.7	19.2
38.840	2.31676	244	15.6	19.2
38.850	2.31618	256	16.0	19.2
38.860	2.31561	257	16.0	19.2
38.870	2.31503	237	15.4	19.2
38.880	2.31445	234	15.3	19.2
38.890	2.31387	236	15.4	19.2
38.901	2.31329	236	15.4	19.2
38.911	2.31272	249	15.8	19.2
38.921	2.31214	253	15.9	19.2
38.931	2.31156	227	15.1	19.2
38.941	2.31099	228	15.1	19.2
38.951	2.31041	240	15.5	19.2
38.961	2.30983	241	15.5	19.2
38.971	2.30926	238	15.4	19.2
38.981	2.30868	206	14.4	19.2
38.991	2.30811	246	15.7	19.2
39.002	2.30754	227	15.1	19.2
39.012	2.30696	247	15.7	19.2
39.022	2.30639	233	15.3	19.2
39.032	2.30581	267	16.3	19.2
39.042	2.30524	270	16.4	19.2
39.052	2.30467	257	16.0	19.2
39.062	2.30410	241	15.5	19.2
39.072	2.30352	233	15.3	19.2
39.082	2.30295	237	15.4	19.2
39.092	2.30238	249	15.8	19.2
39.103	2.30181	231	15.2	19.2
39.113	2.30124	213	14.6	19.2
39.123	2.30067	214	14.6	19.2
39.133	2.30010	242	15.6	19.2
39.143	2.29953	244	15.6	19.2
39.153	2.29896	222	14.9	19.2
39.163	2.29839	221	14.9	19.2
39.173	2.29782	240	15.5	19.2
39.183	2.29725	234	15.3	19.2
39.193	2.29668	264	16.2	19.2
39.204	2.29611	231	15.2	19.2
39.214	2.29554	255	16.0	19.2
39.224	2.29497	238	15.4	19.2
39.234	2.29441	213	14.6	19.2
39.244	2.29384	232	15.2	19.2
39.254	2.29327	276	16.6	19.2
39.264	2.29271	241	15.5	19.2
39.274	2.29214	214	14.6	19.2
39.284	2.29157	230	15.2	19.2
39.294	2.29101	294	17.1	19.2
39.304	2.29044	228	15.1	19.2
39.315	2.28988	239	15.5	19.2
39.325	2.28931	224	15.0	19.2

39.335	2.28875	239	15.5	19.2
39.345	2.28818	239	15.5	19.2
39.355	2.28762	227	15.1	19.2
39.365	2.28706	256	16.0	19.2
39.375	2.28649	251	15.8	19.2
39.385	2.28593	233	15.3	19.2
39.395	2.28537	235	15.3	19.2
39.405	2.28481	227	15.1	19.2
39.416	2.28424	230	15.2	19.2
39.426	2.28368	251	15.8	19.2
39.436	2.28312	222	14.9	19.2
39.446	2.28256	240	15.5	19.2
39.456	2.28200	254	15.9	19.2
39.466	2.28144	241	15.5	19.2
39.476	2.28088	238	15.4	19.2
39.486	2.28032	236	15.4	19.2
39.496	2.27976	228	15.1	19.2
39.506	2.27920	247	15.7	19.2
39.517	2.27864	244	15.6	19.2
39.527	2.27808	228	15.1	19.2
39.537	2.27752	218	14.8	19.2
39.547	2.27696	232	15.2	19.2
39.557	2.27640	225	15.0	19.2
39.567	2.27585	229	15.1	19.2
39.577	2.27529	248	15.7	19.2
39.587	2.27473	227	15.1	19.2
39.597	2.27417	229	15.1	19.2
39.607	2.27362	227	15.1	19.2
39.618	2.27306	245	15.7	19.2
39.628	2.27251	187	13.7	19.2
39.638	2.27195	236	15.4	19.2
39.648	2.27139	232	15.2	19.2
39.658	2.27084	239	15.5	19.2
39.668	2.27028	226	15.0	19.2
39.678	2.26973	257	16.0	19.2
39.688	2.26918	227	15.1	19.2
39.698	2.26862	228	15.1	19.2
39.708	2.26807	244	15.6	19.2
39.719	2.26751	238	15.4	19.2
39.729	2.26696	241	15.5	19.2
39.739	2.26641	237	15.4	19.2
39.749	2.26586	242	15.6	19.2
39.759	2.26530	243	15.6	19.2
39.769	2.26475	212	14.6	19.2
39.779	2.26420	243	15.6	19.2
39.789	2.26365	231	15.2	19.2
39.799	2.26310	217	14.7	19.2
39.809	2.26255	232	15.2	19.2
39.820	2.26200	232	15.2	19.2
39.830	2.26145	216	14.7	19.2

39.840	2.26090	227	15.1	19.2
39.850	2.26035	238	15.4	19.2
39.860	2.25980	241	15.5	19.2
39.870	2.25925	225	15.0	19.2
39.880	2.25870	211	14.5	19.2
39.890	2.25815	224	15.0	19.2
39.900	2.25760	203	14.2	19.2
39.910	2.25705	232	15.2	19.2
39.921	2.25651	222	14.9	19.2
39.931	2.25596	212	14.6	19.2
39.941	2.25541	232	15.2	19.2
39.951	2.25486	235	15.3	19.2
39.961	2.25432	234	15.3	19.2
39.971	2.25377	232	15.2	19.2
39.981	2.25323	233	15.3	19.2
39.991	2.25268	231	15.2	19.2
40.001	2.25213	214	14.6	19.2
40.011	2.25159	233	15.3	19.2
40.022	2.25104	238	15.4	19.2
40.032	2.25050	227	15.1	19.2
40.042	2.24996	211	14.5	19.2
40.052	2.24941	236	15.4	19.2
40.062	2.24887	229	15.1	19.2
40.072	2.24832	243	15.6	19.2
40.082	2.24778	240	15.5	19.2
40.092	2.24724	233	15.3	19.2
40.102	2.24669	243	15.6	19.2
40.112	2.24615	233	15.3	19.2
40.123	2.24561	224	15.0	19.2
40.133	2.24507	221	14.9	19.2
40.143	2.24453	224	15.0	19.2
40.153	2.24399	228	15.1	19.2
40.163	2.24344	225	15.0	19.2
40.173	2.24290	232	15.2	19.2
40.183	2.24236	210	14.5	19.2
40.193	2.24182	226	15.0	19.2
40.203	2.24128	222	14.9	19.2
40.213	2.24074	240	15.5	19.2
40.224	2.24020	218	14.8	19.2
40.234	2.23967	206	14.4	19.2
40.244	2.23913	217	14.7	19.2
40.254	2.23859	201	14.2	19.2
40.264	2.23805	240	15.5	19.2
40.274	2.23751	203	14.2	19.2
40.284	2.23697	213	14.6	19.2
40.294	2.23644	227	15.1	19.2
40.304	2.23590	244	15.6	19.2
40.314	2.23536	237	15.4	19.2
40.325	2.23483	224	15.0	19.2
40.335	2.23429	234	15.3	19.2

40.345	2.23375	230	15.2	19.2
40.355	2.23322	225	15.0	19.2
40.365	2.23268	214	14.6	19.2
40.375	2.23215	234	15.3	19.2
40.385	2.23161	201	14.2	19.2
40.395	2.23108	221	14.9	19.2
40.405	2.23054	211	14.5	19.2
40.415	2.23001	192	13.9	19.2
40.426	2.22948	242	15.6	19.2
40.436	2.22894	209	14.5	19.2
40.446	2.22841	211	14.5	19.2
40.456	2.22788	223	14.9	19.2
40.466	2.22734	244	15.6	19.2
40.476	2.22681	251	15.8	19.2
40.486	2.22628	240	15.5	19.2
40.496	2.22575	250	15.8	19.2
40.506	2.22522	202	14.2	19.2
40.516	2.22468	223	14.9	19.2
40.527	2.22415	242	15.6	19.2
40.537	2.22362	231	15.2	19.2
40.547	2.22309	195	14.0	19.2
40.557	2.22256	218	14.8	19.2
40.567	2.22203	207	14.4	19.2
40.577	2.22150	236	15.4	19.2
40.587	2.22097	219	14.8	19.2
40.597	2.22044	241	15.5	19.2
40.607	2.21991	227	15.1	19.2
40.617	2.21939	250	15.8	19.2
40.627	2.21886	225	15.0	19.2
40.638	2.21833	233	15.3	19.2
40.648	2.21780	225	15.0	19.2
40.658	2.21727	236	15.4	19.2
40.668	2.21675	201	14.2	19.2
40.678	2.21622	223	14.9	19.2
40.688	2.21569	232	15.2	19.2
40.698	2.21517	213	14.6	19.2
40.708	2.21464	215	14.7	19.2
40.718	2.21411	203	14.2	19.2
40.728	2.21359	246	15.7	19.2
40.739	2.21306	207	14.4	19.2
40.749	2.21254	219	14.8	19.2
40.759	2.21201	228	15.1	19.2
40.769	2.21149	211	14.5	19.2
40.779	2.21096	222	14.9	19.2
40.789	2.21044	260	16.1	19.2
40.799	2.20992	187	13.7	19.2
40.809	2.20939	202	14.2	19.2
40.819	2.20887	218	14.8	19.2
40.829	2.20835	219	14.8	19.2
40.840	2.20782	226	15.0	19.2

40.850	2.20730	218	14.8	19.2
40.860	2.20678	214	14.6	19.2
40.870	2.20626	210	14.5	19.2
40.880	2.20573	187	13.7	19.2
40.890	2.20521	223	14.9	19.2
40.900	2.20469	232	15.2	19.2
40.910	2.20417	215	14.7	19.2
40.920	2.20365	192	13.9	19.2
40.930	2.20313	198	14.1	19.2
40.941	2.20261	207	14.4	19.2
40.951	2.20209	239	15.5	19.2
40.961	2.20157	237	15.4	19.2
40.971	2.20105	221	14.9	19.2
40.981	2.20053	202	14.2	19.2
40.991	2.20001	204	14.3	19.2
41.001	2.19949	204	14.3	19.2
41.011	2.19898	210	14.5	19.2
41.021	2.19846	203	14.2	19.2
41.031	2.19794	216	14.7	19.2
41.042	2.19742	225	15.0	19.2
41.052	2.19690	206	14.4	19.2
41.062	2.19639	204	14.3	19.2
41.072	2.19587	237	15.4	19.2
41.082	2.19535	220	14.8	19.2
41.092	2.19484	195	14.0	19.2
41.102	2.19432	213	14.6	19.2
41.112	2.19381	199	14.1	19.2
41.122	2.19329	229	15.1	19.2
41.132	2.19278	212	14.6	19.2
41.143	2.19226	232	15.2	19.2
41.153	2.19175	237	15.4	19.2
41.163	2.19123	221	14.9	19.2
41.173	2.19072	207	14.4	19.2
41.183	2.19020	211	14.5	19.2
41.193	2.18969	213	14.6	19.2
41.203	2.18918	196	14.0	19.2
41.213	2.18866	218	14.8	19.2
41.223	2.18815	225	15.0	19.2
41.233	2.18764	186	13.6	19.2
41.244	2.18713	228	15.1	19.2
41.254	2.18661	196	14.0	19.2
41.264	2.18610	212	14.6	19.2
41.274	2.18559	221	14.9	19.2
41.284	2.18508	226	15.0	19.2
41.294	2.18457	220	14.8	19.2
41.304	2.18406	191	13.8	19.2
41.314	2.18355	221	14.9	19.2
41.324	2.18304	218	14.8	19.2
41.334	2.18253	230	15.2	19.2
41.345	2.18202	212	14.6	19.2

41.355	2.18151	214	14.6	19.2
41.365	2.18100	219	14.8	19.2
41.375	2.18049	221	14.9	19.2
41.385	2.17998	222	14.9	19.2
41.395	2.17947	224	15.0	19.2
41.405	2.17896	210	14.5	19.2
41.415	2.17846	217	14.7	19.2
41.425	2.17795	226	15.0	19.2
41.435	2.17744	218	14.8	19.2
41.446	2.17693	208	14.4	19.2
41.456	2.17643	215	14.7	19.2
41.466	2.17592	202	14.2	19.2
41.476	2.17541	187	13.7	19.2
41.486	2.17491	211	14.5	19.2
41.496	2.17440	224	15.0	19.2
41.506	2.17389	209	14.5	19.2
41.516	2.17339	197	14.0	19.2
41.526	2.17288	213	14.6	19.2
41.536	2.17238	225	15.0	19.2
41.547	2.17187	213	14.6	19.2
41.557	2.17137	214	14.6	19.2
41.567	2.17087	208	14.4	19.2
41.577	2.17036	211	14.5	19.2
41.587	2.16986	217	14.7	19.2
41.597	2.16935	177	13.3	19.2
41.607	2.16885	194	13.9	19.2
41.617	2.16835	192	13.9	19.2
41.627	2.16785	229	15.1	19.2
41.637	2.16734	231	15.2	19.2
41.648	2.16684	244	15.6	19.2
41.658	2.16634	202	14.2	19.2
41.668	2.16584	205	14.3	19.2
41.678	2.16534	205	14.3	19.2
41.688	2.16483	204	14.3	19.2
41.698	2.16433	200	14.1	19.2
41.708	2.16383	189	13.7	19.2
41.718	2.16333	203	14.2	19.2
41.728	2.16283	220	14.8	19.2
41.738	2.16233	222	14.9	19.2
41.749	2.16183	186	13.6	19.2
41.759	2.16133	223	14.9	19.2
41.769	2.16083	209	14.5	19.2
41.779	2.16033	196	14.0	19.2
41.789	2.15984	206	14.4	19.2
41.799	2.15934	200	14.1	19.2
41.809	2.15884	204	14.3	19.2
41.819	2.15834	197	14.0	19.2
41.829	2.15784	216	14.7	19.2
41.839	2.15735	214	14.6	19.2
41.850	2.15685	233	15.3	19.2

41.860	2.15635	215	14.7	19.2
41.870	2.15585	187	13.7	19.2
41.880	2.15536	208	14.4	19.2
41.890	2.15486	212	14.6	19.2
41.900	2.15437	198	14.1	19.2
41.910	2.15387	219	14.8	19.2
41.920	2.15337	230	15.2	19.2
41.930	2.15288	233	15.3	19.2
41.940	2.15238	237	15.4	19.2
41.950	2.15189	195	14.0	19.2
41.961	2.15139	202	14.2	19.2
41.971	2.15090	189	13.7	19.2
41.981	2.15041	195	14.0	19.2
41.991	2.14991	192	13.9	19.2
42.001	2.14942	207	14.4	19.2
42.011	2.14893	188	13.7	19.2
42.021	2.14843	184	13.6	19.2
42.031	2.14794	216	14.7	19.2
42.041	2.14745	221	14.9	19.2
42.051	2.14695	208	14.4	19.2
42.062	2.14646	186	13.6	19.2
42.072	2.14597	199	14.1	19.2
42.082	2.14548	202	14.2	19.2
42.092	2.14499	211	14.5	19.2
42.102	2.14450	214	14.6	19.2
42.112	2.14400	208	14.4	19.2
42.122	2.14351	190	13.8	19.2
42.132	2.14302	200	14.1	19.2
42.142	2.14253	168	13.0	19.2
42.152	2.14204	205	14.3	19.2
42.163	2.14155	190	13.8	19.2
42.173	2.14106	207	14.4	19.2
42.183	2.14058	208	14.4	19.2
42.193	2.14009	196	14.0	19.2
42.203	2.13960	172	13.1	19.2
42.213	2.13911	199	14.1	19.2
42.223	2.13862	188	13.7	19.2
42.233	2.13813	195	14.0	19.2
42.243	2.13764	189	13.7	19.2
42.253	2.13716	182	13.5	19.2
42.264	2.13667	189	13.7	19.2
42.274	2.13618	202	14.2	19.2
42.284	2.13570	205	14.3	19.2
42.294	2.13521	208	14.4	19.2
42.304	2.13472	187	13.7	19.2
42.314	2.13424	188	13.7	19.2
42.324	2.13375	218	14.8	19.2
42.334	2.13327	175	13.2	19.2
42.344	2.13278	182	13.5	19.2
42.354	2.13229	185	13.6	19.2

42.365	2.13181	176	13.3	19.2
42.375	2.13132	185	13.6	19.2
42.385	2.13084	175	13.2	19.2
42.395	2.13036	191	13.8	19.2
42.405	2.12987	237	15.4	19.2
42.415	2.12939	201	14.2	19.2
42.425	2.12890	212	14.6	19.2
42.435	2.12842	211	14.5	19.2
42.445	2.12794	211	14.5	19.2
42.455	2.12746	169	13.0	19.2
42.466	2.12697	192	13.9	19.2
42.476	2.12649	176	13.3	19.2
42.486	2.12601	193	13.9	19.2
42.496	2.12553	227	15.1	19.2
42.506	2.12504	173	13.2	19.2
42.516	2.12456	193	13.9	19.2
42.526	2.12408	196	14.0	19.2
42.536	2.12360	158	12.6	19.2
42.546	2.12312	234	15.3	19.2
42.556	2.12264	175	13.2	19.2
42.567	2.12216	190	13.8	19.2
42.577	2.12168	196	14.0	19.2
42.587	2.12120	195	14.0	19.2
42.597	2.12072	192	13.9	19.2
42.607	2.12024	185	13.6	19.2
42.617	2.11976	196	14.0	19.2
42.627	2.11928	202	14.2	19.2
42.637	2.11880	197	14.0	19.2
42.647	2.11833	175	13.2	19.2
42.657	2.11785	187	13.7	19.2
42.668	2.11737	203	14.2	19.2
42.678	2.11689	173	13.2	19.2
42.688	2.11642	209	14.5	19.2
42.698	2.11594	187	13.7	19.2
42.708	2.11546	200	14.1	19.2
42.718	2.11498	188	13.7	19.2
42.728	2.11451	202	14.2	19.2
42.738	2.11403	182	13.5	19.2
42.748	2.11356	206	14.4	19.2
42.758	2.11308	216	14.7	19.2
42.769	2.11260	184	13.6	19.2
42.779	2.11213	200	14.1	19.2
42.789	2.11165	184	13.6	19.2
42.799	2.11118	194	13.9	19.2
42.809	2.11070	192	13.9	19.2
42.819	2.11023	195	14.0	19.2
42.829	2.10976	189	13.7	19.2
42.839	2.10928	181	13.5	19.2
42.849	2.10881	187	13.7	19.2
42.859	2.10833	213	14.6	19.2

42.870	2.10786	175	13.2	19.2
42.880	2.10739	177	13.3	19.2
42.890	2.10691	186	13.6	19.2
42.900	2.10644	184	13.6	19.2
42.910	2.10597	188	13.7	19.2
42.920	2.10550	182	13.5	19.2
42.930	2.10503	180	13.4	19.2
42.940	2.10455	170	13.0	19.2
42.950	2.10408	171	13.1	19.2
42.960	2.10361	206	14.4	19.2
42.971	2.10314	187	13.7	19.2
42.981	2.10267	187	13.7	19.2
42.991	2.10220	175	13.2	19.2
43.001	2.10173	203	14.2	19.2
43.011	2.10126	191	13.8	19.2
43.021	2.10079	177	13.3	19.2
43.031	2.10032	208	14.4	19.2
43.041	2.09985	193	13.9	19.2
43.051	2.09938	178	13.3	19.2
43.061	2.09891	200	14.1	19.2
43.072	2.09844	198	14.1	19.2
43.082	2.09797	177	13.3	19.2
43.092	2.09751	171	13.1	19.2
43.102	2.09704	177	13.3	19.2
43.112	2.09657	179	13.4	19.2
43.122	2.09610	190	13.8	19.2
43.132	2.09563	179	13.4	19.2
43.142	2.09517	190	13.8	19.2
43.152	2.09470	186	13.6	19.2
43.162	2.09423	185	13.6	19.2
43.172	2.09377	183	13.5	19.2
43.183	2.09330	168	13.0	19.2
43.193	2.09284	198	14.1	19.2
43.203	2.09237	179	13.4	19.2
43.213	2.09190	180	13.4	19.2
43.223	2.09144	188	13.7	19.2
43.233	2.09097	212	14.6	19.2
43.243	2.09051	192	13.9	19.2
43.253	2.09004	181	13.5	19.2
43.263	2.08958	173	13.2	19.2
43.273	2.08911	181	13.5	19.2
43.284	2.08865	192	13.9	19.2
43.294	2.08819	191	13.8	19.2
43.304	2.08772	200	14.1	19.2
43.314	2.08726	193	13.9	19.2
43.324	2.08680	173	13.2	19.2
43.334	2.08633	184	13.6	19.2
43.344	2.08587	210	14.5	19.2
43.354	2.08541	185	13.6	19.2
43.364	2.08495	179	13.4	19.2

43.374	2.08448	145	12.0	19.2
43.385	2.08402	222	14.9	19.2
43.395	2.08356	159	12.6	19.2
43.405	2.08310	197	14.0	19.2
43.415	2.08264	181	13.5	19.2
43.425	2.08218	177	13.3	19.2
43.435	2.08172	184	13.6	19.2
43.445	2.08126	193	13.9	19.2
43.455	2.08080	163	12.8	19.2
43.465	2.08034	188	13.7	19.2
43.475	2.07988	166	12.9	19.2
43.486	2.07942	188	13.7	19.2
43.496	2.07896	158	12.6	19.2
43.506	2.07850	147	12.1	19.2
43.516	2.07804	187	13.7	19.2
43.526	2.07758	202	14.2	19.2
43.536	2.07712	217	14.7	19.2
43.546	2.07666	183	13.5	19.2
43.556	2.07620	187	13.7	19.2
43.566	2.07575	184	13.6	19.2
43.576	2.07529	170	13.0	19.2
43.587	2.07483	202	14.2	19.2
43.597	2.07437	191	13.8	19.2
43.607	2.07392	182	13.5	19.2
43.617	2.07346	174	13.2	19.2
43.627	2.07300	178	13.3	19.2
43.637	2.07255	196	14.0	19.2
43.647	2.07209	163	12.8	19.2
43.657	2.07164	171	13.1	19.2
43.667	2.07118	189	13.7	19.2
43.677	2.07072	171	13.1	19.2
43.688	2.07027	190	13.8	19.2
43.698	2.06981	172	13.1	19.2
43.708	2.06936	193	13.9	19.2
43.718	2.06890	183	13.5	19.2
43.728	2.06845	182	13.5	19.2
43.738	2.06800	193	13.9	19.2
43.748	2.06754	176	13.3	19.2
43.758	2.06709	176	13.3	19.2
43.768	2.06663	167	12.9	19.2
43.778	2.06618	189	13.7	19.2
43.789	2.06573	180	13.4	19.2
43.799	2.06528	179	13.4	19.2
43.809	2.06482	179	13.4	19.2
43.819	2.06437	188	13.7	19.2
43.829	2.06392	197	14.0	19.2
43.839	2.06347	176	13.3	19.2
43.849	2.06301	167	12.9	19.2
43.859	2.06256	174	13.2	19.2
43.869	2.06211	184	13.6	19.2

43.879	2.06166	184	13.6	19.2
43.890	2.06121	163	12.8	19.2
43.900	2.06076	172	13.1	19.2
43.910	2.06031	157	12.5	19.2
43.920	2.05986	167	12.9	19.2
43.930	2.05941	164	12.8	19.2
43.940	2.05896	180	13.4	19.2
43.950	2.05851	196	14.0	19.2
43.960	2.05806	159	12.6	19.2
43.970	2.05761	171	13.1	19.2
43.980	2.05716	178	13.3	19.2
43.991	2.05671	173	13.2	19.2
44.001	2.05626	147	12.1	19.2
44.011	2.05581	195	14.0	19.2
44.021	2.05537	191	13.8	19.2
44.031	2.05492	176	13.3	19.2
44.041	2.05447	154	12.4	19.2
44.051	2.05402	178	13.3	19.2
44.061	2.05357	168	13.0	19.2
44.071	2.05313	181	13.5	19.2
44.081	2.05268	187	13.7	19.2
44.092	2.05223	170	13.0	19.2
44.102	2.05179	178	13.3	19.2
44.112	2.05134	155	12.4	19.2
44.122	2.05089	162	12.7	19.2
44.132	2.05045	181	13.5	19.2
44.142	2.05000	166	12.9	19.2
44.152	2.04956	167	12.9	19.2
44.162	2.04911	173	13.2	19.2
44.172	2.04867	157	12.5	19.2
44.182	2.04822	172	13.1	19.2
44.193	2.04778	175	13.2	19.2
44.203	2.04733	172	13.1	19.2
44.213	2.04689	174	13.2	19.2
44.223	2.04645	146	12.1	19.2
44.233	2.04600	192	13.9	19.2
44.243	2.04556	176	13.3	19.2
44.253	2.04511	182	13.5	19.2
44.263	2.04467	162	12.7	19.2
44.273	2.04423	167	12.9	19.2
44.283	2.04379	181	13.5	19.2
44.294	2.04334	178	13.3	19.2
44.304	2.04290	179	13.4	19.2
44.314	2.04246	145	12.0	19.2
44.324	2.04202	173	13.2	19.2
44.334	2.04157	161	12.7	19.2
44.344	2.04113	162	12.7	19.2
44.354	2.04069	166	12.9	19.2
44.364	2.04025	170	13.0	19.2
44.374	2.03981	189	13.7	19.2

44.384	2.03937	181	13.5	19.2
44.395	2.03893	144	12.0	19.2
44.405	2.03849	159	12.6	19.2
44.415	2.03805	140	11.8	19.2
44.425	2.03761	159	12.6	19.2
44.435	2.03717	166	12.9	19.2
44.445	2.03673	164	12.8	19.2
44.455	2.03629	159	12.6	19.2
44.465	2.03585	174	13.2	19.2
44.475	2.03541	169	13.0	19.2
44.485	2.03497	198	14.1	19.2
44.495	2.03454	195	14.0	19.2
44.506	2.03410	170	13.0	19.2
44.516	2.03366	196	14.0	19.2
44.526	2.03322	165	12.8	19.2
44.536	2.03278	161	12.7	19.2
44.546	2.03235	182	13.5	19.2
44.556	2.03191	167	12.9	19.2
44.566	2.03147	154	12.4	19.2
44.576	2.03104	181	13.5	19.2
44.586	2.03060	159	12.6	19.2
44.596	2.03016	163	12.8	19.2
44.607	2.02973	180	13.4	19.2
44.617	2.02929	169	13.0	19.2
44.627	2.02885	168	13.0	19.2
44.637	2.02842	164	12.8	19.2
44.647	2.02798	173	13.2	19.2
44.657	2.02755	154	12.4	19.2
44.667	2.02711	161	12.7	19.2
44.677	2.02668	164	12.8	19.2
44.687	2.02624	160	12.6	19.2
44.697	2.02581	178	13.3	19.2
44.708	2.02538	152	12.3	19.2
44.718	2.02494	165	12.8	19.2
44.728	2.02451	166	12.9	19.2
44.738	2.02407	167	12.9	19.2
44.748	2.02364	167	12.9	19.2
44.758	2.02321	155	12.4	19.2
44.768	2.02277	145	12.0	19.2
44.778	2.02234	165	12.8	19.2
44.788	2.02191	178	13.3	19.2
44.798	2.02148	163	12.8	19.2
44.809	2.02104	192	13.9	19.2
44.819	2.02061	166	12.9	19.2
44.829	2.02018	161	12.7	19.2
44.839	2.01975	169	13.0	19.2
44.849	2.01932	170	13.0	19.2
44.859	2.01889	156	12.5	19.2
44.869	2.01846	144	12.0	19.2
44.879	2.01802	176	13.3	19.2

44.889	2.01759	175	13.2	19.2
44.899	2.01716	163	12.8	19.2
44.910	2.01673	155	12.4	19.2
44.920	2.01630	162	12.7	19.2
44.930	2.01587	164	12.8	19.2
44.940	2.01544	153	12.4	19.2
44.950	2.01502	164	12.8	19.2
44.960	2.01459	165	12.8	19.2
44.970	2.01416	168	13.0	19.2
44.980	2.01373	157	12.5	19.2
44.990	2.01330	146	12.1	19.2
45.000	2.01287	187	13.7	19.2
45.011	2.01244	171	13.1	19.2
45.021	2.01202	172	13.1	19.2
45.031	2.01159	157	12.5	19.2
45.041	2.01116	158	12.6	19.2
45.051	2.01073	171	13.1	19.2
45.061	2.01031	164	12.8	19.2
45.071	2.00988	164	12.8	19.2
45.081	2.00945	131	11.4	19.2
45.091	2.00902	171	13.1	19.2
45.101	2.00860	161	12.7	19.2
45.112	2.00817	165	12.8	19.2
45.122	2.00775	157	12.5	19.2
45.132	2.00732	147	12.1	19.2
45.142	2.00689	158	12.6	19.2
45.152	2.00647	157	12.5	19.2
45.162	2.00604	173	13.2	19.2
45.172	2.00562	169	13.0	19.2
45.182	2.00519	182	13.5	19.2
45.192	2.00477	144	12.0	19.2
45.202	2.00435	141	11.9	19.2
45.213	2.00392	142	11.9	19.2
45.223	2.00350	170	13.0	19.2
45.233	2.00307	160	12.6	19.2
45.243	2.00265	146	12.1	19.2
45.253	2.00223	154	12.4	19.2
45.263	2.00180	161	12.7	19.2
45.273	2.00138	147	12.1	19.2
45.283	2.00096	156	12.5	19.2
45.293	2.00053	157	12.5	19.2
45.303	2.00011	162	12.7	19.2
45.314	1.99969	146	12.1	19.2
45.324	1.99927	158	12.6	19.2
45.334	1.99885	159	12.6	19.2
45.344	1.99842	156	12.5	19.2
45.354	1.99800	135	11.6	19.2
45.364	1.99758	163	12.8	19.2
45.374	1.99716	161	12.7	19.2
45.384	1.99674	134	11.6	19.2

45.394	1.99632	145	12.0	19.2
45.404	1.99590	150	12.2	19.2
45.415	1.99548	143	12.0	19.2
45.425	1.99506	156	12.5	19.2
45.435	1.99464	147	12.1	19.2
45.445	1.99422	159	12.6	19.2
45.455	1.99380	162	12.7	19.2
45.465	1.99338	179	13.4	19.2
45.475	1.99296	168	13.0	19.2
45.485	1.99254	167	12.9	19.2
45.495	1.99212	153	12.4	19.2
45.505	1.99170	138	11.7	19.2
45.516	1.99128	184	13.6	19.2
45.526	1.99087	150	12.2	19.2
45.536	1.99045	166	12.9	19.2
45.546	1.99003	156	12.5	19.2
45.556	1.98961	171	13.1	19.2
45.566	1.98919	167	12.9	19.2
45.576	1.98878	161	12.7	19.2
45.586	1.98836	171	13.1	19.2
45.596	1.98794	149	12.2	19.2
45.606	1.98753	169	13.0	19.2
45.617	1.98711	161	12.7	19.2
45.627	1.98669	167	12.9	19.2
45.637	1.98628	158	12.6	19.2
45.647	1.98586	147	12.1	19.2
45.657	1.98545	159	12.6	19.2
45.667	1.98503	159	12.6	19.2
45.677	1.98461	164	12.8	19.2
45.687	1.98420	151	12.3	19.2
45.697	1.98378	149	12.2	19.2
45.707	1.98337	153	12.4	19.2
45.718	1.98295	154	12.4	19.2
45.728	1.98254	156	12.5	19.2
45.738	1.98213	167	12.9	19.2
45.748	1.98171	150	12.2	19.2
45.758	1.98130	167	12.9	19.2
45.768	1.98088	167	12.9	19.2
45.778	1.98047	169	13.0	19.2
45.788	1.98006	173	13.2	19.2
45.798	1.97964	153	12.4	19.2
45.808	1.97923	156	12.5	19.2
45.818	1.97882	161	12.7	19.2
45.829	1.97841	174	13.2	19.2
45.839	1.97799	171	13.1	19.2
45.849	1.97758	139	11.8	19.2
45.859	1.97717	165	12.8	19.2
45.869	1.97676	184	13.6	19.2
45.879	1.97635	168	13.0	19.2
45.889	1.97593	151	12.3	19.2

45.899	1.97552	179	13.4	19.2
45.909	1.97511	161	12.7	19.2
45.919	1.97470	141	11.9	19.2
45.930	1.97429	170	13.0	19.2
45.940	1.97388	159	12.6	19.2
45.950	1.97347	193	13.9	19.2
45.960	1.97306	149	12.2	19.2
45.970	1.97265	158	12.6	19.2
45.980	1.97224	164	12.8	19.2
45.990	1.97183	133	11.5	19.2
46.000	1.97142	156	12.5	19.2
46.010	1.97101	161	12.7	19.2
46.020	1.97060	157	12.5	19.2
46.031	1.97019	161	12.7	19.2
46.041	1.96979	158	12.6	19.2
46.051	1.96938	156	12.5	19.2
46.061	1.96897	141	11.9	19.2
46.071	1.96856	129	11.4	19.2
46.081	1.96815	161	12.7	19.2
46.091	1.96774	174	13.2	19.2
46.101	1.96734	145	12.0	19.2
46.111	1.96693	147	12.1	19.2
46.121	1.96652	157	12.5	19.2
46.132	1.96612	135	11.6	19.2
46.142	1.96571	145	12.0	19.2
46.152	1.96530	168	13.0	19.2
46.162	1.96490	169	13.0	19.2
46.172	1.96449	159	12.6	19.2
46.182	1.96408	155	12.4	19.2
46.192	1.96368	155	12.4	19.2
46.202	1.96327	164	12.8	19.2
46.212	1.96287	145	12.0	19.2
46.222	1.96246	146	12.1	19.2
46.233	1.96206	152	12.3	19.2
46.243	1.96165	163	12.8	19.2
46.253	1.96125	163	12.8	19.2
46.263	1.96084	147	12.1	19.2
46.273	1.96044	159	12.6	19.2
46.283	1.96003	143	12.0	19.2
46.293	1.95963	128	11.3	19.2
46.303	1.95922	172	13.1	19.2
46.313	1.95882	139	11.8	19.2
46.323	1.95842	138	11.7	19.2
46.334	1.95801	173	13.2	19.2
46.344	1.95761	165	12.8	19.2
46.354	1.95721	153	12.4	19.2
46.364	1.95680	151	12.3	19.2
46.374	1.95640	143	12.0	19.2
46.384	1.95600	157	12.5	19.2
46.394	1.95560	134	11.6	19.2

46.404	1.95520	148	12.2	19.2
46.414	1.95479	158	12.6	19.2
46.424	1.95439	141	11.9	19.2
46.435	1.95399	140	11.8	19.2
46.445	1.95359	140	11.8	19.2
46.455	1.95319	180	13.4	19.2
46.465	1.95279	135	11.6	19.2
46.475	1.95239	149	12.2	19.2
46.485	1.95198	156	12.5	19.2
46.495	1.95158	138	11.7	19.2
46.505	1.95118	153	12.4	19.2
46.515	1.95078	168	13.0	19.2
46.525	1.95038	150	12.2	19.2
46.536	1.94998	180	13.4	19.2
46.546	1.94958	141	11.9	19.2
46.556	1.94919	183	13.5	19.2
46.566	1.94879	156	12.5	19.2
46.576	1.94839	145	12.0	19.2
46.586	1.94799	134	11.6	19.2
46.596	1.94759	150	12.2	19.2
46.606	1.94719	166	12.9	19.2
46.616	1.94679	158	12.6	19.2
46.626	1.94639	160	12.6	19.2
46.637	1.94600	167	12.9	19.2
46.647	1.94560	159	12.6	19.2
46.657	1.94520	150	12.2	19.2
46.667	1.94480	184	13.6	19.2
46.677	1.94441	125	11.2	19.2
46.687	1.94401	160	12.6	19.2
46.697	1.94361	160	12.6	19.2
46.707	1.94322	136	11.7	19.2
46.717	1.94282	156	12.5	19.2
46.727	1.94242	147	12.1	19.2
46.738	1.94203	140	11.8	19.2
46.748	1.94163	127	11.3	19.2
46.758	1.94123	132	11.5	19.2
46.768	1.94084	156	12.5	19.2
46.778	1.94044	143	12.0	19.2
46.788	1.94005	143	12.0	19.2
46.798	1.93965	173	13.2	19.2
46.808	1.93926	132	11.5	19.2
46.818	1.93886	139	11.8	19.2
46.828	1.93847	158	12.6	19.2
46.839	1.93807	156	12.5	19.2
46.849	1.93768	146	12.1	19.2
46.859	1.93729	148	12.2	19.2
46.869	1.93689	138	11.7	19.2
46.879	1.93650	131	11.4	19.2
46.889	1.93610	148	12.2	19.2
46.899	1.93571	128	11.3	19.2

46.909	1.93532	150	12.2	19.2
46.919	1.93493	149	12.2	19.2
46.929	1.93453	165	12.8	19.2
46.940	1.93414	138	11.7	19.2
46.950	1.93375	140	11.8	19.2
46.960	1.93335	145	12.0	19.2
46.970	1.93296	127	11.3	19.2
46.980	1.93257	144	12.0	19.2
46.990	1.93218	154	12.4	19.2
47.000	1.93179	158	12.6	19.2
47.010	1.93140	156	12.5	19.2
47.020	1.93100	133	11.5	19.2
47.030	1.93061	147	12.1	19.2
47.041	1.93022	157	12.5	19.2
47.051	1.92983	125	11.2	19.2
47.061	1.92944	128	11.3	19.2
47.071	1.92905	172	13.1	19.2
47.081	1.92866	149	12.2	19.2
47.091	1.92827	152	12.3	19.2
47.101	1.92788	140	11.8	19.2
47.111	1.92749	156	12.5	19.2
47.121	1.92710	165	12.8	19.2
47.131	1.92671	148	12.2	19.2
47.141	1.92632	168	13.0	19.2
47.152	1.92593	153	12.4	19.2
47.162	1.92554	176	13.3	19.2
47.172	1.92516	136	11.7	19.2
47.182	1.92477	167	12.9	19.2
47.192	1.92438	123	11.1	19.2
47.202	1.92399	140	11.8	19.2
47.212	1.92360	138	11.7	19.2
47.222	1.92321	137	11.7	19.2
47.232	1.92283	149	12.2	19.2
47.242	1.92244	162	12.7	19.2
47.253	1.92205	136	11.7	19.2
47.263	1.92167	147	12.1	19.2
47.273	1.92128	142	11.9	19.2
47.283	1.92089	138	11.7	19.2
47.293	1.92050	154	12.4	19.2
47.303	1.92012	147	12.1	19.2
47.313	1.91973	145	12.0	19.2
47.323	1.91935	136	11.7	19.2
47.333	1.91896	140	11.8	19.2
47.343	1.91857	147	12.1	19.2
47.354	1.91819	138	11.7	19.2
47.364	1.91780	176	13.3	19.2
47.374	1.91742	144	12.0	19.2
47.384	1.91703	141	11.9	19.2
47.394	1.91665	128	11.3	19.2
47.404	1.91626	164	12.8	19.2

47.414	1.91588	120	11.0	19.2
47.424	1.91549	152	12.3	19.2
47.434	1.91511	144	12.0	19.2
47.444	1.91473	159	12.6	19.2
47.455	1.91434	153	12.4	19.2
47.465	1.91396	139	11.8	19.2
47.475	1.91357	133	11.5	19.2
47.485	1.91319	161	12.7	19.2
47.495	1.91281	145	12.0	19.2
47.505	1.91242	155	12.4	19.2
47.515	1.91204	167	12.9	19.2
47.525	1.91166	150	12.2	19.2
47.535	1.91128	130	11.4	19.2
47.545	1.91089	132	11.5	19.2
47.556	1.91051	139	11.8	19.2
47.566	1.91013	149	12.2	19.2
47.576	1.90975	135	11.6	19.2
47.586	1.90937	159	12.6	19.2
47.596	1.90898	147	12.1	19.2
47.606	1.90860	157	12.5	19.2
47.616	1.90822	146	12.1	19.2
47.626	1.90784	124	11.1	19.2
47.636	1.90746	136	11.7	19.2
47.646	1.90708	133	11.5	19.2
47.657	1.90670	127	11.3	19.2
47.667	1.90632	154	12.4	19.2
47.677	1.90594	135	11.6	19.2
47.687	1.90556	141	11.9	19.2
47.697	1.90518	141	11.9	19.2
47.707	1.90480	145	12.0	19.2
47.717	1.90442	153	12.4	19.2
47.727	1.90404	149	12.2	19.2
47.737	1.90366	151	12.3	19.2
47.747	1.90328	150	12.2	19.2
47.758	1.90290	140	11.8	19.2
47.768	1.90252	144	12.0	19.2
47.778	1.90214	140	11.8	19.2
47.788	1.90177	149	12.2	19.2
47.798	1.90139	140	11.8	19.2
47.808	1.90101	161	12.7	19.2
47.818	1.90063	141	11.9	19.2
47.828	1.90025	140	11.8	19.2
47.838	1.89988	138	11.7	19.2
47.848	1.89950	124	11.1	19.2
47.859	1.89912	145	12.0	19.2
47.869	1.89874	148	12.2	19.2
47.879	1.89837	145	12.0	19.2
47.889	1.89799	153	12.4	19.2
47.899	1.89761	132	11.5	19.2
47.909	1.89724	140	11.8	19.2

47.919	1.89686	126	11.2	19.2
47.929	1.89649	135	11.6	19.2
47.939	1.89611	153	12.4	19.2
47.949	1.89573	134	11.6	19.2
47.960	1.89536	118	10.9	19.2
47.970	1.89498	143	12.0	19.2
47.980	1.89461	152	12.3	19.2
47.990	1.89423	137	11.7	19.2
48.000	1.89386	130	11.4	19.2
48.010	1.89348	130	11.4	19.2
48.020	1.89311	157	12.5	19.2
48.030	1.89273	141	11.9	19.2
48.040	1.89236	135	11.6	19.2
48.050	1.89198	125	11.2	19.2
48.061	1.89161	139	11.8	19.2
48.071	1.89124	130	11.4	19.2
48.081	1.89086	144	12.0	19.2
48.091	1.89049	109	10.4	19.2
48.101	1.89012	143	12.0	19.2
48.111	1.88974	108	10.4	19.2
48.121	1.88937	143	12.0	19.2
48.131	1.88900	141	11.9	19.2
48.141	1.88862	128	11.3	19.2
48.151	1.88825	148	12.2	19.2
48.162	1.88788	136	11.7	19.2
48.172	1.88751	119	10.9	19.2
48.182	1.88714	148	12.2	19.2
48.192	1.88676	132	11.5	19.2
48.202	1.88639	118	10.9	19.2
48.212	1.88602	144	12.0	19.2
48.222	1.88565	123	11.1	19.2
48.232	1.88528	144	12.0	19.2
48.242	1.88491	144	12.0	19.2
48.252	1.88454	131	11.4	19.2
48.263	1.88416	130	11.4	19.2
48.273	1.88379	146	12.1	19.2
48.283	1.88342	138	11.7	19.2
48.293	1.88305	140	11.8	19.2
48.303	1.88268	156	12.5	19.2
48.313	1.88231	152	12.3	19.2
48.323	1.88194	144	12.0	19.2
48.333	1.88157	128	11.3	19.2
48.343	1.88120	131	11.4	19.2
48.353	1.88084	136	11.7	19.2
48.364	1.88047	129	11.4	19.2
48.374	1.88010	143	12.0	19.2
48.384	1.87973	120	11.0	19.2
48.394	1.87936	120	11.0	19.2
48.404	1.87899	133	11.5	19.2
48.414	1.87862	131	11.4	19.2

48.424	1.87825	139	11.8	19.2
48.434	1.87789	137	11.7	19.2
48.444	1.87752	147	12.1	19.2
48.454	1.87715	167	12.9	19.2
48.464	1.87678	136	11.7	19.2
48.475	1.87642	135	11.6	19.2
48.485	1.87605	114	10.7	19.2
48.495	1.87568	143	12.0	19.2
48.505	1.87531	138	11.7	19.2
48.515	1.87495	150	12.2	19.2
48.525	1.87458	151	12.3	19.2
48.535	1.87421	143	12.0	19.2
48.545	1.87385	126	11.2	19.2
48.555	1.87348	114	10.7	19.2
48.565	1.87312	131	11.4	19.2
48.576	1.87275	129	11.4	19.2
48.586	1.87238	116	10.8	19.2
48.596	1.87202	140	11.8	19.2
48.606	1.87165	155	12.4	19.2
48.616	1.87129	131	11.4	19.2
48.626	1.87092	117	10.8	19.2
48.636	1.87056	144	12.0	19.2
48.646	1.87019	140	11.8	19.2
48.656	1.86983	130	11.4	19.2
48.666	1.86946	130	11.4	19.2
48.677	1.86910	152	12.3	19.2
48.687	1.86874	119	10.9	19.2
48.697	1.86837	106	10.3	19.2
48.707	1.86801	145	12.0	19.2
48.717	1.86765	135	11.6	19.2
48.727	1.86728	118	10.9	19.2
48.737	1.86692	127	11.3	19.2
48.747	1.86656	128	11.3	19.2
48.757	1.86619	125	11.2	19.2
48.767	1.86583	136	11.7	19.2
48.778	1.86547	115	10.7	19.2
48.788	1.86510	113	10.6	19.2
48.798	1.86474	120	11.0	19.2
48.808	1.86438	132	11.5	19.2
48.818	1.86402	115	10.7	19.2
48.828	1.86366	131	11.4	19.2
48.838	1.86329	131	11.4	19.2
48.848	1.86293	128	11.3	19.2
48.858	1.86257	122	11.0	19.2
48.868	1.86221	136	11.7	19.2
48.879	1.86185	142	11.9	19.2
48.889	1.86149	151	12.3	19.2
48.899	1.86113	133	11.5	19.2
48.909	1.86077	139	11.8	19.2
48.919	1.86040	135	11.6	19.2

48.929	1.86004	123	11.1	19.2
48.939	1.85968	130	11.4	19.2
48.949	1.85932	125	11.2	19.2
48.959	1.85896	119	10.9	19.2
48.969	1.85860	125	11.2	19.2
48.980	1.85825	129	11.4	19.2
48.990	1.85789	143	12.0	19.2
49.000	1.85753	129	11.4	19.2
49.010	1.85717	137	11.7	19.2
49.020	1.85681	111	10.5	19.2
49.030	1.85645	128	11.3	19.2
49.040	1.85609	124	11.1	19.2
49.050	1.85573	150	12.2	19.2
49.060	1.85537	152	12.3	19.2
49.070	1.85502	135	11.6	19.2
49.081	1.85466	125	11.2	19.2
49.091	1.85430	122	11.0	19.2
49.101	1.85394	153	12.4	19.2
49.111	1.85358	117	10.8	19.2
49.121	1.85323	152	12.3	19.2
49.131	1.85287	114	10.7	19.2
49.141	1.85251	107	10.3	19.2
49.151	1.85216	117	10.8	19.2
49.161	1.85180	124	11.1	19.2
49.171	1.85144	145	12.0	19.2
49.182	1.85108	121	11.0	19.2
49.192	1.85073	136	11.7	19.2
49.202	1.85037	140	11.8	19.2
49.212	1.85002	147	12.1	19.2
49.222	1.84966	115	10.7	19.2
49.232	1.84930	143	12.0	19.2
49.242	1.84895	134	11.6	19.2
49.252	1.84859	120	11.0	19.2
49.262	1.84824	119	10.9	19.2
49.272	1.84788	126	11.2	19.2
49.283	1.84753	130	11.4	19.2
49.293	1.84717	126	11.2	19.2
49.303	1.84682	128	11.3	19.2
49.313	1.84646	138	11.7	19.2
49.323	1.84611	117	10.8	19.2
49.333	1.84575	131	11.4	19.2
49.343	1.84540	127	11.3	19.2
49.353	1.84505	121	11.0	19.2
49.363	1.84469	91.0	9.54	19.2
49.373	1.84434	129	11.4	19.2
49.384	1.84399	126	11.2	19.2
49.394	1.84363	130	11.4	19.2
49.404	1.84328	113	10.6	19.2
49.414	1.84293	122	11.0	19.2
49.424	1.84257	135	11.6	19.2

49.434	1.84222	135	11.6	19.2
49.444	1.84187	122	11.0	19.2
49.454	1.84151	144	12.0	19.2
49.464	1.84116	126	11.2	19.2
49.474	1.84081	113	10.6	19.2
49.485	1.84046	124	11.1	19.2
49.495	1.84011	130	11.4	19.2
49.505	1.83975	91.0	9.54	19.2
49.515	1.83940	118	10.9	19.2
49.525	1.83905	129	11.4	19.2
49.535	1.83870	111	10.5	19.2
49.545	1.83835	125	11.2	19.2
49.555	1.83800	108	10.4	19.2
49.565	1.83765	112	10.6	19.2
49.575	1.83730	128	11.3	19.2
49.586	1.83695	135	11.6	19.2
49.596	1.83660	124	11.1	19.2
49.606	1.83625	139	11.8	19.2
49.616	1.83590	126	11.2	19.2
49.626	1.83555	134	11.6	19.2
49.636	1.83520	115	10.7	19.2
49.646	1.83485	118	10.9	19.2
49.656	1.83450	111	10.5	19.2
49.666	1.83415	111	10.5	19.2
49.676	1.83380	125	11.2	19.2
49.687	1.83345	109	10.4	19.2
49.697	1.83310	131	11.4	19.2
49.707	1.83275	137	11.7	19.2
49.717	1.83240	134	11.6	19.2
49.727	1.83205	127	11.3	19.2
49.737	1.83171	122	11.0	19.2
49.747	1.83136	113	10.6	19.2
49.757	1.83101	114	10.7	19.2
49.767	1.83066	118	10.9	19.2
49.777	1.83031	132	11.5	19.2
49.787	1.82997	133	11.5	19.2
49.798	1.82962	116	10.8	19.2
49.808	1.82927	100	10.0	19.2
49.818	1.82892	106	10.3	19.2
49.828	1.82858	111	10.5	19.2
49.838	1.82823	125	11.2	19.2
49.848	1.82788	123	11.1	19.2
49.858	1.82754	132	11.5	19.2
49.868	1.82719	116	10.8	19.2
49.878	1.82684	112	10.6	19.2
49.888	1.82650	122	11.0	19.2
49.899	1.82615	119	10.9	19.2
49.909	1.82581	132	11.5	19.2
49.919	1.82546	116	10.8	19.2
49.929	1.82511	108	10.4	19.2

49.939	1.82477	146	12.1	19.2
49.949	1.82442	119	10.9	19.2
49.959	1.82408	123	11.1	19.2
49.969	1.82373	131	11.4	19.2
49.979	1.82339	135	11.6	19.2
49.989	1.82304	143	12.0	19.2
50.000	1.82270	116	10.8	19.2
50.010	1.82235	127	11.3	19.2
50.020	1.82201	99.0	9.95	19.2
50.030	1.82167	109	10.4	19.2
50.040	1.82132	114	10.7	19.2
50.050	1.82098	114	10.7	19.2
50.060	1.82063	100	10.0	19.2
50.070	1.82029	115	10.7	19.2
50.080	1.81995	105	10.2	19.2
50.090	1.81960	117	10.8	19.2
50.101	1.81926	104	10.2	19.2
50.111	1.81892	96.0	9.80	19.2
50.121	1.81858	95.0	9.75	19.2
50.131	1.81823	127	11.3	19.2
50.141	1.81789	138	11.7	19.2
50.151	1.81755	125	11.2	19.2
50.161	1.81721	141	11.9	19.2
50.171	1.81686	128	11.3	19.2
50.181	1.81652	110	10.5	19.2
50.191	1.81618	126	11.2	19.2
50.202	1.81584	100	10.0	19.2
50.212	1.81550	115	10.7	19.2
50.222	1.81516	114	10.7	19.2
50.232	1.81481	137	11.7	19.2
50.242	1.81447	121	11.0	19.2
50.252	1.81413	125	11.2	19.2
50.262	1.81379	126	11.2	19.2
50.272	1.81345	127	11.3	19.2
50.282	1.81311	107	10.3	19.2
50.292	1.81277	118	10.9	19.2
50.303	1.81243	117	10.8	19.2
50.313	1.81209	126	11.2	19.2
50.323	1.81175	124	11.1	19.2
50.333	1.81141	129	11.4	19.2
50.343	1.81107	141	11.9	19.2
50.353	1.81073	141	11.9	19.2
50.363	1.81039	118	10.9	19.2
50.373	1.81005	130	11.4	19.2
50.383	1.80971	143	12.0	19.2
50.393	1.80937	129	11.4	19.2
50.404	1.80903	111	10.5	19.2
50.414	1.80870	115	10.7	19.2
50.424	1.80836	111	10.5	19.2
50.434	1.80802	130	11.4	19.2

50.444	1.80768	124	11.1	19.2
50.454	1.80734	130	11.4	19.2
50.464	1.80700	121	11.0	19.2
50.474	1.80667	137	11.7	19.2
50.484	1.80633	92.0	9.59	19.2
50.494	1.80599	115	10.7	19.2
50.505	1.80565	102	10.1	19.2
50.515	1.80532	103	10.1	19.2
50.525	1.80498	100	10.0	19.2
50.535	1.80464	116	10.8	19.2
50.545	1.80430	123	11.1	19.2
50.555	1.80397	92.0	9.59	19.2
50.565	1.80363	109	10.4	19.2
50.575	1.80330	136	11.7	19.2
50.585	1.80296	136	11.7	19.2
50.595	1.80262	127	11.3	19.2
50.606	1.80229	99.0	9.95	19.2
50.616	1.80195	120	11.0	19.2
50.626	1.80161	107	10.3	19.2
50.636	1.80128	128	11.3	19.2
50.646	1.80094	125	11.2	19.2
50.656	1.80061	112	10.6	19.2
50.666	1.80027	110	10.5	19.2
50.676	1.79994	121	11.0	19.2
50.686	1.79960	121	11.0	19.2
50.696	1.79927	120	11.0	19.2
50.707	1.79893	110	10.5	19.2
50.717	1.79860	115	10.7	19.2
50.727	1.79826	108	10.4	19.2
50.737	1.79793	110	10.5	19.2
50.747	1.79760	110	10.5	19.2
50.757	1.79726	116	10.8	19.2
50.767	1.79693	114	10.7	19.2
50.777	1.79659	124	11.1	19.2
50.787	1.79626	117	10.8	19.2
50.797	1.79593	105	10.2	19.2
50.808	1.79559	124	11.1	19.2
50.818	1.79526	120	11.0	19.2
50.828	1.79493	87.0	9.33	19.2
50.838	1.79460	101	10.0	19.2
50.848	1.79426	128	11.3	19.2
50.858	1.79393	128	11.3	19.2
50.868	1.79360	122	11.0	19.2
50.878	1.79327	109	10.4	19.2
50.888	1.79293	98.0	9.90	19.2
50.898	1.79260	112	10.6	19.2
50.909	1.79227	113	10.6	19.2
50.919	1.79194	100	10.0	19.2
50.929	1.79161	119	10.9	19.2
50.939	1.79127	116	10.8	19.2

50.949	1.79094	94.0	9.70	19.2
50.959	1.79061	121	11.0	19.2
50.969	1.79028	114	10.7	19.2
50.979	1.78995	133	11.5	19.2
50.989	1.78962	118	10.9	19.2
50.999	1.78929	100	10.0	19.2
51.010	1.78896	121	11.0	19.2
51.020	1.78863	93.0	9.64	19.2
51.030	1.78830	123	11.1	19.2
51.040	1.78797	115	10.7	19.2
51.050	1.78764	95.0	9.75	19.2
51.060	1.78731	134	11.6	19.2
51.070	1.78698	115	10.7	19.2
51.080	1.78665	122	11.0	19.2
51.090	1.78632	114	10.7	19.2
51.100	1.78599	99.0	9.95	19.2
51.110	1.78566	111	10.5	19.2
51.121	1.78533	110	10.5	19.2
51.131	1.78500	98.0	9.90	19.2
51.141	1.78467	113	10.6	19.2
51.151	1.78434	112	10.6	19.2
51.161	1.78402	109	10.4	19.2
51.171	1.78369	118	10.9	19.2
51.181	1.78336	116	10.8	19.2
51.191	1.78303	124	11.1	19.2
51.201	1.78270	94.0	9.70	19.2
51.211	1.78238	107	10.3	19.2
51.222	1.78205	110	10.5	19.2
51.232	1.78172	99.0	9.95	19.2
51.242	1.78139	106	10.3	19.2
51.252	1.78106	116	10.8	19.2
51.262	1.78074	100	10.0	19.2
51.272	1.78041	132	11.5	19.2
51.282	1.78008	99.0	9.95	19.2
51.292	1.77976	85.0	9.22	19.2
51.302	1.77943	109	10.4	19.2
51.312	1.77910	97.0	9.85	19.2
51.323	1.77878	118	10.9	19.2
51.333	1.77845	97.0	9.85	19.2
51.343	1.77813	108	10.4	19.2
51.353	1.77780	106	10.3	19.2
51.363	1.77747	106	10.3	19.2
51.373	1.77715	114	10.7	19.2
51.383	1.77682	114	10.7	19.2
51.393	1.77650	107	10.3	19.2
51.403	1.77617	108	10.4	19.2
51.413	1.77585	89.0	9.43	19.2
51.424	1.77552	103	10.1	19.2
51.434	1.77520	89.0	9.43	19.2
51.444	1.77487	110	10.5	19.2

51.454	1.77455	105	10.2	19.2
51.464	1.77422	98.0	9.90	19.2
51.474	1.77390	100	10.0	19.2
51.484	1.77357	103	10.1	19.2
51.494	1.77325	97.0	9.85	19.2
51.504	1.77293	115	10.7	19.2
51.514	1.77260	97.0	9.85	19.2
51.525	1.77228	98.0	9.90	19.2
51.535	1.77195	128	11.3	19.2
51.545	1.77163	111	10.5	19.2
51.555	1.77131	112	10.6	19.2
51.565	1.77098	127	11.3	19.2
51.575	1.77066	83.0	9.11	19.2
51.585	1.77034	111	10.5	19.2
51.595	1.77002	123	11.1	19.2
51.605	1.76969	103	10.1	19.2
51.615	1.76937	131	11.4	19.2
51.626	1.76905	106	10.3	19.2
51.636	1.76873	101	10.0	19.2
51.646	1.76840	101	10.0	19.2
51.656	1.76808	117	10.8	19.2
51.666	1.76776	106	10.3	19.2
51.676	1.76744	108	10.4	19.2
51.686	1.76712	111	10.5	19.2
51.696	1.76680	113	10.6	19.2
51.706	1.76647	115	10.7	19.2
51.716	1.76615	89.0	9.43	19.2
51.727	1.76583	102	10.1	19.2
51.737	1.76551	113	10.6	19.2
51.747	1.76519	107	10.3	19.2
51.757	1.76487	104	10.2	19.2
51.767	1.76455	68.0	8.25	19.2
51.777	1.76423	105	10.2	19.2
51.787	1.76391	107	10.3	19.2
51.797	1.76359	100	10.0	19.2
51.807	1.76327	100	10.0	19.2
51.817	1.76295	103	10.1	19.2
51.828	1.76263	112	10.6	19.2
51.838	1.76231	108	10.4	19.2
51.848	1.76199	96.0	9.80	19.2
51.858	1.76167	107	10.3	19.2
51.868	1.76135	83.0	9.11	19.2
51.878	1.76103	109	10.4	19.2
51.888	1.76071	118	10.9	19.2
51.898	1.76039	94.0	9.70	19.2
51.908	1.76007	101	10.0	19.2
51.918	1.75976	104	10.2	19.2
51.929	1.75944	110	10.5	19.2
51.939	1.75912	111	10.5	19.2
51.949	1.75880	100	10.0	19.2

51.959	1.75848	116	10.8	19.2
51.969	1.75816	104	10.2	19.2
51.979	1.75785	94.0	9.70	19.2
51.989	1.75753	92.0	9.59	19.2
51.999	1.75721	106	10.3	19.2
52.009	1.75689	89.0	9.43	19.2
52.019	1.75658	101	10.0	19.2
52.030	1.75626	93.0	9.64	19.2
52.040	1.75594	92.0	9.59	19.2
52.050	1.75563	89.0	9.43	19.2
52.060	1.75531	97.0	9.85	19.2
52.070	1.75499	122	11.0	19.2
52.080	1.75467	94.0	9.70	19.2
52.090	1.75436	95.0	9.75	19.2
52.100	1.75404	109	10.4	19.2
52.110	1.75373	98.0	9.90	19.2
52.120	1.75341	103	10.1	19.2
52.131	1.75309	98.0	9.90	19.2
52.141	1.75278	102	10.1	19.2
52.151	1.75246	111	10.5	19.2
52.161	1.75215	92.0	9.59	19.2
52.171	1.75183	105	10.2	19.2
52.181	1.75152	119	10.9	19.2
52.191	1.75120	104	10.2	19.2
52.201	1.75089	89.0	9.43	19.2
52.211	1.75057	105	10.2	19.2
52.221	1.75026	97.0	9.85	19.2
52.232	1.74994	86.0	9.27	19.2
52.242	1.74963	84.0	9.17	19.2
52.252	1.74931	109	10.4	19.2
52.262	1.74900	104	10.2	19.2
52.272	1.74868	111	10.5	19.2
52.282	1.74837	97.0	9.85	19.2
52.292	1.74806	85.0	9.22	19.2
52.302	1.74774	106	10.3	19.2
52.312	1.74743	72.0	8.49	19.2
52.322	1.74712	94.0	9.70	19.2
52.333	1.74680	91.0	9.54	19.2
52.343	1.74649	89.0	9.43	19.2
52.353	1.74618	82.0	9.06	19.2
52.363	1.74586	95.0	9.75	19.2
52.373	1.74555	104	10.2	19.2
52.383	1.74524	91.0	9.54	19.2
52.393	1.74492	102	10.1	19.2
52.403	1.74461	105	10.2	19.2
52.413	1.74430	108	10.4	19.2
52.423	1.74399	93.0	9.64	19.2
52.433	1.74367	90.0	9.49	19.2
52.444	1.74336	113	10.6	19.2
52.454	1.74305	93.0	9.64	19.2

52.464	1.74274	101	10.0	19.2
52.474	1.74243	73.0	8.54	19.2
52.484	1.74212	96.0	9.80	19.2
52.494	1.74180	100	10.0	19.2
52.504	1.74149	84.0	9.17	19.2
52.514	1.74118	79.0	8.89	19.2
52.524	1.74087	91.0	9.54	19.2
52.534	1.74056	84.0	9.17	19.2
52.545	1.74025	92.0	9.59	19.2
52.555	1.73994	84.0	9.17	19.2
52.565	1.73963	101	10.0	19.2
52.575	1.73932	87.0	9.33	19.2
52.585	1.73901	109	10.4	19.2
52.595	1.73870	87.0	9.33	19.2
52.605	1.73839	71.0	8.43	19.2
52.615	1.73808	107	10.3	19.2
52.625	1.73777	95.0	9.75	19.2
52.635	1.73746	93.0	9.64	19.2
52.646	1.73715	83.0	9.11	19.2
52.656	1.73684	92.0	9.59	19.2
52.666	1.73653	97.0	9.85	19.2
52.676	1.73622	97.0	9.85	19.2
52.686	1.73591	86.0	9.27	19.2
52.696	1.73560	93.0	9.64	19.2
52.706	1.73529	120	11.0	19.2
52.716	1.73499	84.0	9.17	19.2
52.726	1.73468	97.0	9.85	19.2
52.736	1.73437	87.0	9.33	19.2
52.747	1.73406	83.0	9.11	19.2
52.757	1.73375	87.0	9.33	19.2
52.767	1.73344	86.0	9.27	19.2
52.777	1.73314	94.0	9.70	19.2
52.787	1.73283	95.0	9.75	19.2
52.797	1.73252	88.0	9.38	19.2
52.807	1.73221	89.0	9.43	19.2
52.817	1.73191	97.0	9.85	19.2
52.827	1.73160	79.0	8.89	19.2
52.837	1.73129	108	10.4	19.2
52.848	1.73098	80.0	8.94	19.2
52.858	1.73068	93.0	9.64	19.2
52.868	1.73037	100	10.0	19.2
52.878	1.73006	96.0	9.80	19.2
52.888	1.72976	79.0	8.89	19.2
52.898	1.72945	100	10.0	19.2
52.908	1.72914	90.0	9.49	19.2
52.918	1.72884	99.0	9.95	19.2
52.928	1.72853	81.0	9.00	19.2
52.938	1.72823	86.0	9.27	19.2
52.949	1.72792	91.0	9.54	19.2
52.959	1.72761	85.0	9.22	19.2

52.969	1.72731	97.0	9.85	19.2
52.979	1.72700	91.0	9.54	19.2
52.989	1.72670	80.0	8.94	19.2
52.999	1.72639	88.0	9.38	19.2
53.009	1.72609	82.0	9.06	19.2
53.019	1.72578	81.0	9.00	19.2
53.029	1.72548	73.0	8.54	19.2
53.039	1.72517	99.0	9.95	19.2
53.050	1.72487	85.0	9.22	19.2
53.060	1.72456	89.0	9.43	19.2
53.070	1.72426	90.0	9.49	19.2
53.080	1.72396	75.0	8.66	19.2
53.090	1.72365	92.0	9.59	19.2
53.100	1.72335	105	10.2	19.2
53.110	1.72304	81.0	9.00	19.2
53.120	1.72274	65.0	8.06	19.2
53.130	1.72244	84.0	9.17	19.2
53.140	1.72213	74.0	8.60	19.2
53.151	1.72183	84.0	9.17	19.2
53.161	1.72153	90.0	9.49	19.2
53.171	1.72122	79.0	8.89	19.2
53.181	1.72092	86.0	9.27	19.2
53.191	1.72062	86.0	9.27	19.2
53.201	1.72031	77.0	8.77	19.2
53.211	1.72001	75.0	8.66	19.2
53.221	1.71971	85.0	9.22	19.2
53.231	1.71941	80.0	8.94	19.2
53.241	1.71910	83.0	9.11	19.2
53.252	1.71880	92.0	9.59	19.2
53.262	1.71850	79.0	8.89	19.2
53.272	1.71820	85.0	9.22	19.2
53.282	1.71790	71.0	8.43	19.2
53.292	1.71759	70.0	8.37	19.2
53.302	1.71729	72.0	8.49	19.2
53.312	1.71699	98.0	9.90	19.2
53.322	1.71669	71.0	8.43	19.2
53.332	1.71639	90.0	9.49	19.2
53.342	1.71609	77.0	8.77	19.2
53.353	1.71579	87.0	9.33	19.2
53.363	1.71548	82.0	9.06	19.2
53.373	1.71518	80.0	8.94	19.2
53.383	1.71488	91.0	9.54	19.2
53.393	1.71458	80.0	8.94	19.2
53.403	1.71428	97.0	9.85	19.2
53.413	1.71398	83.0	9.11	19.2
53.423	1.71368	64.0	8.00	19.2
53.433	1.71338	61.0	7.81	19.2
53.443	1.71308	81.0	9.00	19.2
53.454	1.71278	91.0	9.54	19.2
53.464	1.71248	91.0	9.54	19.2

53.474	1.71218	98.0	9.90	19.2
53.484	1.71188	96.0	9.80	19.2
53.494	1.71158	99.0	9.95	19.2
53.504	1.71128	90.0	9.49	19.2
53.514	1.71099	76.0	8.72	19.2
53.524	1.71069	77.0	8.77	19.2
53.534	1.71039	103	10.1	19.2
53.544	1.71009	90.0	9.49	19.2
53.555	1.70979	70.0	8.37	19.2
53.565	1.70949	119	10.9	19.2
53.575	1.70919	77.0	8.77	19.2
53.585	1.70889	83.0	9.11	19.2
53.595	1.70860	100	10.0	19.2
53.605	1.70830	75.0	8.66	19.2
53.615	1.70800	83.0	9.11	19.2
53.625	1.70770	77.0	8.77	19.2
53.635	1.70740	93.0	9.64	19.2
53.645	1.70711	85.0	9.22	19.2
53.656	1.70681	101	10.0	19.2
53.666	1.70651	82.0	9.06	19.2
53.676	1.70622	83.0	9.11	19.2
53.686	1.70592	88.0	9.38	19.2
53.696	1.70562	86.0	9.27	19.2
53.706	1.70532	78.0	8.83	19.2
53.716	1.70503	89.0	9.43	19.2
53.726	1.70473	97.0	9.85	19.2
53.736	1.70443	89.0	9.43	19.2
53.746	1.70414	75.0	8.66	19.2
53.756	1.70384	75.0	8.66	19.2
53.767	1.70355	66.0	8.12	19.2
53.777	1.70325	65.0	8.06	19.2
53.787	1.70295	90.0	9.49	19.2
53.797	1.70266	72.0	8.49	19.2
53.807	1.70236	65.0	8.06	19.2
53.817	1.70207	80.0	8.94	19.2
53.827	1.70177	95.0	9.75	19.2
53.837	1.70147	91.0	9.54	19.2
53.847	1.70118	85.0	9.22	19.2
53.857	1.70088	74.0	8.60	19.2
53.868	1.70059	86.0	9.27	19.2
53.878	1.70029	81.0	9.00	19.2
53.888	1.70000	83.0	9.11	19.2
53.898	1.69970	81.0	9.00	19.2
53.908	1.69941	91.0	9.54	19.2
53.918	1.69912	75.0	8.66	19.2
53.928	1.69882	72.0	8.49	19.2
53.938	1.69853	81.0	9.00	19.2
53.948	1.69823	76.0	8.72	19.2
53.958	1.69794	79.0	8.89	19.2
53.969	1.69765	64.0	8.00	19.2

53.979	1.69735	63.0	7.94	19.2
53.989	1.69706	68.0	8.25	19.2
53.999	1.69676	86.0	9.27	19.2
54.009	1.69647	80.0	8.94	19.2
54.019	1.69618	77.0	8.77	19.2
54.029	1.69588	78.0	8.83	19.2
54.039	1.69559	81.0	9.00	19.2
54.049	1.69530	80.0	8.94	19.2
54.059	1.69501	100	10.0	19.2
54.070	1.69471	72.0	8.49	19.2
54.080	1.69442	87.0	9.33	19.2
54.090	1.69413	84.0	9.17	19.2
54.100	1.69383	86.0	9.27	19.2
54.110	1.69354	70.0	8.37	19.2
54.120	1.69325	71.0	8.43	19.2
54.130	1.69296	101	10.0	19.2
54.140	1.69267	83.0	9.11	19.2
54.150	1.69237	74.0	8.60	19.2
54.160	1.69208	74.0	8.60	19.2
54.171	1.69179	79.0	8.89	19.2
54.181	1.69150	76.0	8.72	19.2
54.191	1.69121	79.0	8.89	19.2
54.201	1.69092	81.0	9.00	19.2
54.211	1.69063	79.0	8.89	19.2
54.221	1.69033	84.0	9.17	19.2
54.231	1.69004	83.0	9.11	19.2
54.241	1.68975	75.0	8.66	19.2
54.251	1.68946	79.0	8.89	19.2
54.261	1.68917	71.0	8.43	19.2
54.272	1.68888	83.0	9.11	19.2
54.282	1.68859	69.0	8.31	19.2
54.292	1.68830	82.0	9.06	19.2
54.302	1.68801	81.0	9.00	19.2
54.312	1.68772	79.0	8.89	19.2
54.322	1.68743	97.0	9.85	19.2
54.332	1.68714	80.0	8.94	19.2
54.342	1.68685	70.0	8.37	19.2
54.352	1.68656	80.0	8.94	19.2
54.362	1.68627	76.0	8.72	19.2
54.373	1.68598	103	10.1	19.2
54.383	1.68569	66.0	8.12	19.2
54.393	1.68540	75.0	8.66	19.2
54.403	1.68512	88.0	9.38	19.2
54.413	1.68483	79.0	8.89	19.2
54.423	1.68454	73.0	8.54	19.2
54.433	1.68425	63.0	7.94	19.2
54.443	1.68396	96.0	9.80	19.2
54.453	1.68367	82.0	9.06	19.2
54.463	1.68338	71.0	8.43	19.2
54.474	1.68310	81.0	9.00	19.2

54.484	1.68281	74.0	8.60	19.2
54.494	1.68252	86.0	9.27	19.2
54.504	1.68223	77.0	8.77	19.2
54.514	1.68194	73.0	8.54	19.2
54.524	1.68166	80.0	8.94	19.2
54.534	1.68137	89.0	9.43	19.2
54.544	1.68108	90.0	9.49	19.2
54.554	1.68079	72.0	8.49	19.2
54.564	1.68051	96.0	9.80	19.2
54.575	1.68022	81.0	9.00	19.2
54.585	1.67993	78.0	8.83	19.2
54.595	1.67965	73.0	8.54	19.2
54.605	1.67936	92.0	9.59	19.2
54.615	1.67907	74.0	8.60	19.2
54.625	1.67879	80.0	8.94	19.2
54.635	1.67850	64.0	8.00	19.2
54.645	1.67821	67.0	8.19	19.2
54.655	1.67793	74.0	8.60	19.2
54.665	1.67764	80.0	8.94	19.2
54.676	1.67735	75.0	8.66	19.2
54.686	1.67707	80.0	8.94	19.2
54.696	1.67678	89.0	9.43	19.2
54.706	1.67650	58.0	7.62	19.2
54.716	1.67621	84.0	9.17	19.2
54.726	1.67593	81.0	9.00	19.2
54.736	1.67564	78.0	8.83	19.2
54.746	1.67536	64.0	8.00	19.2
54.756	1.67507	67.0	8.19	19.2
54.766	1.67479	68.0	8.25	19.2
54.777	1.67450	81.0	9.00	19.2
54.787	1.67422	56.0	7.48	19.2
54.797	1.67393	68.0	8.25	19.2
54.807	1.67365	78.0	8.83	19.2
54.817	1.67336	65.0	8.06	19.2
54.827	1.67308	89.0	9.43	19.2
54.837	1.67279	63.0	7.94	19.2
54.847	1.67251	72.0	8.49	19.2
54.857	1.67222	63.0	7.94	19.2
54.867	1.67194	85.0	9.22	19.2
54.878	1.67166	72.0	8.49	19.2
54.888	1.67137	70.0	8.37	19.2
54.898	1.67109	57.0	7.55	19.2
54.908	1.67081	65.0	8.06	19.2
54.918	1.67052	67.0	8.19	19.2
54.928	1.67024	90.0	9.49	19.2
54.938	1.66996	70.0	8.37	19.2
54.948	1.66967	66.0	8.12	19.2
54.958	1.66939	80.0	8.94	19.2
54.968	1.66911	78.0	8.83	19.2
54.978	1.66883	77.0	8.77	19.2

54.989	1.66854	77.0	8.77	19.2
54.999	1.66826	74.0	8.60	19.2
55.009	1.66798	66.0	8.12	19.2
55.019	1.66770	56.0	7.48	19.2
55.029	1.66741	53.0	7.28	19.2
55.039	1.66713	75.0	8.66	19.2
55.049	1.66685	69.0	8.31	19.2
55.059	1.66657	61.0	7.81	19.2
55.069	1.66629	62.0	7.87	19.2
55.079	1.66600	69.0	8.31	19.2
55.090	1.66572	71.0	8.43	19.2
55.100	1.66544	54.0	7.35	19.2
55.110	1.66516	78.0	8.83	19.2
55.120	1.66488	76.0	8.72	19.2
55.130	1.66460	63.0	7.94	19.2
55.140	1.66432	81.0	9.00	19.2
55.150	1.66404	65.0	8.06	19.2
55.160	1.66375	67.0	8.19	19.2
55.170	1.66347	70.0	8.37	19.2
55.180	1.66319	76.0	8.72	19.2
55.191	1.66291	64.0	8.00	19.2
55.201	1.66263	69.0	8.31	19.2
55.211	1.66235	65.0	8.06	19.2
55.221	1.66207	85.0	9.22	19.2
55.231	1.66179	49.0	7.00	19.2
55.241	1.66151	71.0	8.43	19.2
55.251	1.66123	60.0	7.75	19.2
55.261	1.66095	62.0	7.87	19.2
55.271	1.66067	51.0	7.14	19.2
55.281	1.66039	72.0	8.49	19.2
55.292	1.66011	70.0	8.37	19.2
55.302	1.65984	87.0	9.33	19.2
55.312	1.65956	71.0	8.43	19.2
55.322	1.65928	56.0	7.48	19.2
55.332	1.65900	49.0	7.00	19.2
55.342	1.65872	58.0	7.62	19.2
55.352	1.65844	67.0	8.19	19.2
55.362	1.65816	76.0	8.72	19.2
55.372	1.65788	52.0	7.21	19.2
55.382	1.65761	83.0	9.11	19.2
55.393	1.65733	54.0	7.35	19.2
55.403	1.65705	58.0	7.62	19.2
55.413	1.65677	69.0	8.31	19.2
55.423	1.65649	66.0	8.12	19.2
55.433	1.65621	63.0	7.94	19.2
55.443	1.65594	59.0	7.68	19.2
55.453	1.65566	58.0	7.62	19.2
55.463	1.65538	56.0	7.48	19.2
55.473	1.65510	70.0	8.37	19.2
55.483	1.65483	39.0	6.24	19.2

55.494	1.65455	59.0	7.68	19.2
55.504	1.65427	55.0	7.42	19.2
55.514	1.65400	64.0	8.00	19.2
55.524	1.65372	70.0	8.37	19.2
55.534	1.65344	63.0	7.94	19.2
55.544	1.65316	57.0	7.55	19.2
55.554	1.65289	58.0	7.62	19.2
55.564	1.65261	63.0	7.94	19.2
55.574	1.65234	60.0	7.75	19.2
55.584	1.65206	65.0	8.06	19.2
55.595	1.65178	62.0	7.87	19.2
55.605	1.65151	54.0	7.35	19.2
55.615	1.65123	74.0	8.60	19.2
55.625	1.65095	55.0	7.42	19.2
55.635	1.65068	51.0	7.14	19.2
55.645	1.65040	63.0	7.94	19.2
55.655	1.65013	67.0	8.19	19.2
55.665	1.64985	69.0	8.31	19.2
55.675	1.64958	71.0	8.43	19.2
55.685	1.64930	52.0	7.21	19.2
55.696	1.64903	56.0	7.48	19.2
55.706	1.64875	67.0	8.19	19.2
55.716	1.64848	52.0	7.21	19.2
55.726	1.64820	52.0	7.21	19.2
55.736	1.64793	54.0	7.35	19.2
55.746	1.64765	70.0	8.37	19.2
55.756	1.64738	77.0	8.77	19.2
55.766	1.64710	50.0	7.07	19.2
55.776	1.64683	57.0	7.55	19.2
55.786	1.64656	57.0	7.55	19.2
55.797	1.64628	67.0	8.19	19.2
55.807	1.64601	61.0	7.81	19.2
55.817	1.64573	53.0	7.28	19.2
55.827	1.64546	50.0	7.07	19.2
55.837	1.64519	68.0	8.25	19.2
55.847	1.64491	60.0	7.75	19.2
55.857	1.64464	58.0	7.62	19.2
55.867	1.64437	69.0	8.31	19.2
55.877	1.64409	75.0	8.66	19.2
55.887	1.64382	65.0	8.06	19.2
55.898	1.64355	64.0	8.00	19.2
55.908	1.64327	66.0	8.12	19.2
55.918	1.64300	71.0	8.43	19.2
55.928	1.64273	68.0	8.25	19.2
55.938	1.64245	63.0	7.94	19.2
55.948	1.64218	59.0	7.68	19.2
55.958	1.64191	62.0	7.87	19.2
55.968	1.64164	46.0	6.78	19.2
55.978	1.64136	61.0	7.81	19.2
55.988	1.64109	61.0	7.81	19.2

55.999	1.64082	65.0	8.06	19.2
56.009	1.64055	76.0	8.72	19.2
56.019	1.64028	51.0	7.14	19.2
56.029	1.64001	64.0	8.00	19.2
56.039	1.63973	73.0	8.54	19.2
56.049	1.63946	52.0	7.21	19.2
56.059	1.63919	65.0	8.06	19.2
56.069	1.63892	62.0	7.87	19.2
56.079	1.63865	54.0	7.35	19.2
56.089	1.63838	56.0	7.48	19.2
56.100	1.63811	69.0	8.31	19.2
56.110	1.63784	58.0	7.62	19.2
56.120	1.63756	65.0	8.06	19.2
56.130	1.63729	70.0	8.37	19.2
56.140	1.63702	64.0	8.00	19.2
56.150	1.63675	58.0	7.62	19.2
56.160	1.63648	68.0	8.25	19.2
56.170	1.63621	62.0	7.87	19.2
56.180	1.63594	53.0	7.28	19.2
56.190	1.63567	59.0	7.68	19.2
56.201	1.63540	45.0	6.71	19.2
56.211	1.63513	66.0	8.12	19.2
56.221	1.63486	55.0	7.42	19.2
56.231	1.63459	61.0	7.81	19.2
56.241	1.63432	59.0	7.68	19.2
56.251	1.63405	66.0	8.12	19.2
56.261	1.63378	65.0	8.06	19.2
56.271	1.63351	64.0	8.00	19.2
56.281	1.63325	53.0	7.28	19.2
56.291	1.63298	66.0	8.12	19.2
56.301	1.63271	57.0	7.55	19.2
56.312	1.63244	55.0	7.42	19.2
56.322	1.63217	50.0	7.07	19.2
56.332	1.63190	64.0	8.00	19.2
56.342	1.63163	55.0	7.42	19.2
56.352	1.63136	68.0	8.25	19.2
56.362	1.63110	58.0	7.62	19.2
56.372	1.63083	63.0	7.94	19.2
56.382	1.63056	55.0	7.42	19.2
56.392	1.63029	52.0	7.21	19.2
56.402	1.63002	54.0	7.35	19.2
56.413	1.62976	51.0	7.14	19.2
56.423	1.62949	66.0	8.12	19.2
56.433	1.62922	55.0	7.42	19.2
56.443	1.62895	64.0	8.00	19.2
56.453	1.62868	73.0	8.54	19.2
56.463	1.62842	44.0	6.63	19.2
56.473	1.62815	56.0	7.48	19.2
56.483	1.62788	56.0	7.48	19.2
56.493	1.62762	53.0	7.28	19.2

56.503	1.62735	57.0	7.55	19.2
56.514	1.62708	53.0	7.28	19.2
56.524	1.62682	59.0	7.68	19.2
56.534	1.62655	58.0	7.62	19.2
56.544	1.62628	49.0	7.00	19.2
56.554	1.62602	56.0	7.48	19.2
56.564	1.62575	48.0	6.93	19.2
56.574	1.62548	75.0	8.66	19.2
56.584	1.62522	63.0	7.94	19.2
56.594	1.62495	49.0	7.00	19.2
56.604	1.62469	52.0	7.21	19.2
56.615	1.62442	57.0	7.55	19.2
56.625	1.62415	51.0	7.14	19.2
56.635	1.62389	62.0	7.87	19.2
56.645	1.62362	49.0	7.00	19.2
56.655	1.62336	54.0	7.35	19.2
56.665	1.62309	51.0	7.14	19.2
56.675	1.62283	51.0	7.14	19.2
56.685	1.62256	41.0	6.40	19.2
56.695	1.62230	44.0	6.63	19.2
56.705	1.62203	53.0	7.28	19.2
56.716	1.62177	62.0	7.87	19.2
56.726	1.62150	46.0	6.78	19.2
56.736	1.62124	65.0	8.06	19.2
56.746	1.62097	61.0	7.81	19.2
56.756	1.62071	40.0	6.32	19.2
56.766	1.62044	53.0	7.28	19.2
56.776	1.62018	53.0	7.28	19.2
56.786	1.61992	53.0	7.28	19.2
56.796	1.61965	46.0	6.78	19.2
56.806	1.61939	54.0	7.35	19.2
56.817	1.61912	71.0	8.43	19.2
56.827	1.61886	47.0	6.86	19.2
56.837	1.61860	67.0	8.19	19.2
56.847	1.61833	51.0	7.14	19.2
56.857	1.61807	57.0	7.55	19.2
56.867	1.61781	54.0	7.35	19.2
56.877	1.61754	63.0	7.94	19.2
56.887	1.61728	50.0	7.07	19.2
56.897	1.61702	63.0	7.94	19.2
56.907	1.61675	54.0	7.35	19.2
56.918	1.61649	45.0	6.71	19.2
56.928	1.61623	52.0	7.21	19.2
56.938	1.61596	40.0	6.32	19.2
56.948	1.61570	52.0	7.21	19.2
56.958	1.61544	54.0	7.35	19.2
56.968	1.61518	52.0	7.21	19.2
56.978	1.61491	53.0	7.28	19.2
56.988	1.61465	56.0	7.48	19.2
56.998	1.61439	46.0	6.78	19.2

57.008	1.61413	50.0	7.07	19.2
57.019	1.61387	58.0	7.62	19.2
57.029	1.61360	54.0	7.35	19.2
57.039	1.61334	50.0	7.07	19.2
57.049	1.61308	68.0	8.25	19.2
57.059	1.61282	46.0	6.78	19.2
57.069	1.61256	56.0	7.48	19.2
57.079	1.61230	47.0	6.86	19.2
57.089	1.61204	50.0	7.07	19.2
57.099	1.61177	41.0	6.40	19.2
57.109	1.61151	45.0	6.71	19.2
57.120	1.61125	73.0	8.54	19.2
57.130	1.61099	49.0	7.00	19.2
57.140	1.61073	58.0	7.62	19.2
57.150	1.61047	58.0	7.62	19.2
57.160	1.61021	53.0	7.28	19.2
57.170	1.60995	58.0	7.62	19.2
57.180	1.60969	45.0	6.71	19.2
57.190	1.60943	53.0	7.28	19.2
57.200	1.60917	47.0	6.86	19.2
57.210	1.60891	48.0	6.93	19.2
57.221	1.60865	40.0	6.32	19.2
57.231	1.60839	61.0	7.81	19.2
57.241	1.60813	66.0	8.12	19.2
57.251	1.60787	52.0	7.21	19.2
57.261	1.60761	60.0	7.75	19.2
57.271	1.60735	37.0	6.08	19.2
57.281	1.60709	63.0	7.94	19.2
57.291	1.60683	65.0	8.06	19.2
57.301	1.60657	42.0	6.48	19.2
57.311	1.60631	43.0	6.56	19.2
57.322	1.60605	54.0	7.35	19.2
57.332	1.60580	39.0	6.24	19.2
57.342	1.60554	61.0	7.81	19.2
57.352	1.60528	57.0	7.55	19.2
57.362	1.60502	54.0	7.35	19.2
57.372	1.60476	57.0	7.55	19.2
57.382	1.60450	53.0	7.28	19.2
57.392	1.60424	56.0	7.48	19.2
57.402	1.60399	50.0	7.07	19.2
57.412	1.60373	54.0	7.35	19.2
57.423	1.60347	65.0	8.06	19.2
57.433	1.60321	63.0	7.94	19.2
57.443	1.60295	50.0	7.07	19.2
57.453	1.60270	52.0	7.21	19.2
57.463	1.60244	59.0	7.68	19.2
57.473	1.60218	50.0	7.07	19.2
57.483	1.60192	49.0	7.00	19.2
57.493	1.60167	49.0	7.00	19.2
57.503	1.60141	55.0	7.42	19.2

57.513	1.60115	47.0	6.86	19.2
57.524	1.60089	53.0	7.28	19.2
57.534	1.60064	67.0	8.19	19.2
57.544	1.60038	44.0	6.63	19.2
57.554	1.60012	44.0	6.63	19.2
57.564	1.59987	51.0	7.14	19.2
57.574	1.59961	42.0	6.48	19.2
57.584	1.59935	56.0	7.48	19.2
57.594	1.59910	70.0	8.37	19.2
57.604	1.59884	66.0	8.12	19.2
57.614	1.59858	59.0	7.68	19.2
57.624	1.59833	44.0	6.63	19.2
57.635	1.59807	50.0	7.07	19.2
57.645	1.59782	49.0	7.00	19.2
57.655	1.59756	46.0	6.78	19.2
57.665	1.59730	66.0	8.12	19.2
57.675	1.59705	55.0	7.42	19.2
57.685	1.59679	55.0	7.42	19.2
57.695	1.59654	54.0	7.35	19.2
57.705	1.59628	39.0	6.24	19.2
57.715	1.59603	53.0	7.28	19.2
57.725	1.59577	57.0	7.55	19.2
57.736	1.59552	52.0	7.21	19.2
57.746	1.59526	48.0	6.93	19.2
57.756	1.59501	43.0	6.56	19.2
57.766	1.59475	54.0	7.35	19.2
57.776	1.59450	40.0	6.32	19.2
57.786	1.59424	56.0	7.48	19.2
57.796	1.59399	46.0	6.78	19.2
57.806	1.59373	43.0	6.56	19.2
57.816	1.59348	54.0	7.35	19.2
57.826	1.59323	50.0	7.07	19.2
57.837	1.59297	53.0	7.28	19.2
57.847	1.59272	46.0	6.78	19.2
57.857	1.59246	50.0	7.07	19.2
57.867	1.59221	51.0	7.14	19.2
57.877	1.59196	35.0	5.92	19.2
57.887	1.59170	54.0	7.35	19.2
57.897	1.59145	50.0	7.07	19.2
57.907	1.59119	59.0	7.68	19.2
57.917	1.59094	48.0	6.93	19.2
57.927	1.59069	53.0	7.28	19.2
57.938	1.59043	42.0	6.48	19.2
57.948	1.59018	55.0	7.42	19.2
57.958	1.58993	60.0	7.75	19.2
57.968	1.58968	39.0	6.24	19.2
57.978	1.58942	46.0	6.78	19.2
57.988	1.58917	61.0	7.81	19.2
57.998	1.58892	51.0	7.14	19.2
58.008	1.58866	49.0	7.00	19.2

58.018	1.58841	46.0	6.78	19.2
58.028	1.58816	49.0	7.00	19.2
58.039	1.58791	68.0	8.25	19.2
58.049	1.58765	62.0	7.87	19.2
58.059	1.58740	44.0	6.63	19.2
58.069	1.58715	48.0	6.93	19.2
58.079	1.58690	50.0	7.07	19.2
58.089	1.58665	41.0	6.40	19.2
58.099	1.58640	51.0	7.14	19.2
58.109	1.58614	43.0	6.56	19.2
58.119	1.58589	48.0	6.93	19.2
58.129	1.58564	45.0	6.71	19.2
58.140	1.58539	41.0	6.40	19.2
58.150	1.58514	46.0	6.78	19.2
58.160	1.58489	52.0	7.21	19.2
58.170	1.58464	54.0	7.35	19.2
58.180	1.58438	39.0	6.24	19.2
58.190	1.58413	43.0	6.56	19.2
58.200	1.58388	43.0	6.56	19.2
58.210	1.58363	50.0	7.07	19.2
58.220	1.58338	52.0	7.21	19.2
58.230	1.58313	52.0	7.21	19.2
58.241	1.58288	45.0	6.71	19.2
58.251	1.58263	37.0	6.08	19.2
58.261	1.58238	45.0	6.71	19.2
58.271	1.58213	41.0	6.40	19.2
58.281	1.58188	41.0	6.40	19.2
58.291	1.58163	44.0	6.63	19.2
58.301	1.58138	49.0	7.00	19.2
58.311	1.58113	42.0	6.48	19.2
58.321	1.58088	42.0	6.48	19.2
58.331	1.58063	44.0	6.63	19.2
58.342	1.58038	50.0	7.07	19.2
58.352	1.58013	45.0	6.71	19.2
58.362	1.57988	42.0	6.48	19.2
58.372	1.57963	44.0	6.63	19.2
58.382	1.57938	55.0	7.42	19.2
58.392	1.57913	43.0	6.56	19.2
58.402	1.57888	53.0	7.28	19.2
58.412	1.57864	49.0	7.00	19.2
58.422	1.57839	47.0	6.86	19.2
58.432	1.57814	43.0	6.56	19.2
58.443	1.57789	50.0	7.07	19.2
58.453	1.57764	38.0	6.16	19.2
58.463	1.57739	54.0	7.35	19.2
58.473	1.57714	44.0	6.63	19.2
58.483	1.57690	39.0	6.24	19.2
58.493	1.57665	47.0	6.86	19.2
58.503	1.57640	41.0	6.40	19.2
58.513	1.57615	52.0	7.21	19.2

58.523	1.57590	43.0	6.56	19.2
58.533	1.57566	48.0	6.93	19.2
58.544	1.57541	41.0	6.40	19.2
58.554	1.57516	39.0	6.24	19.2
58.564	1.57491	53.0	7.28	19.2
58.574	1.57467	43.0	6.56	19.2
58.584	1.57442	40.0	6.32	19.2
58.594	1.57417	47.0	6.86	19.2
58.604	1.57392	43.0	6.56	19.2
58.614	1.57368	44.0	6.63	19.2
58.624	1.57343	40.0	6.32	19.2
58.634	1.57318	44.0	6.63	19.2
58.645	1.57294	38.0	6.16	19.2
58.655	1.57269	39.0	6.24	19.2
58.665	1.57244	51.0	7.14	19.2
58.675	1.57220	38.0	6.16	19.2
58.685	1.57195	50.0	7.07	19.2
58.695	1.57170	44.0	6.63	19.2
58.705	1.57146	40.0	6.32	19.2
58.715	1.57121	40.0	6.32	19.2
58.725	1.57096	37.0	6.08	19.2
58.735	1.57072	48.0	6.93	19.2
58.746	1.57047	59.0	7.68	19.2
58.756	1.57023	45.0	6.71	19.2
58.766	1.56998	49.0	7.00	19.2
58.776	1.56973	54.0	7.35	19.2
58.786	1.56949	39.0	6.24	19.2
58.796	1.56924	54.0	7.35	19.2
58.806	1.56900	49.0	7.00	19.2
58.816	1.56875	42.0	6.48	19.2
58.826	1.56851	46.0	6.78	19.2
58.836	1.56826	43.0	6.56	19.2
58.847	1.56802	43.0	6.56	19.2
58.857	1.56777	42.0	6.48	19.2
58.867	1.56753	43.0	6.56	19.2
58.877	1.56728	34.0	5.83	19.2
58.887	1.56704	38.0	6.16	19.2
58.897	1.56679	51.0	7.14	19.2
58.907	1.56655	39.0	6.24	19.2
58.917	1.56630	48.0	6.93	19.2
58.927	1.56606	41.0	6.40	19.2
58.937	1.56582	36.0	6.00	19.2
58.947	1.56557	40.0	6.32	19.2
58.958	1.56533	42.0	6.48	19.2
58.968	1.56508	48.0	6.93	19.2
58.978	1.56484	43.0	6.56	19.2
58.988	1.56460	42.0	6.48	19.2
58.998	1.56435	44.0	6.63	19.2
59.008	1.56411	42.0	6.48	19.2
59.018	1.56386	44.0	6.63	19.2

59.028	1.56362	41.0	6.40	19.2
59.038	1.56338	33.0	5.74	19.2
59.048	1.56313	43.0	6.56	19.2
59.059	1.56289	39.0	6.24	19.2
59.069	1.56265	48.0	6.93	19.2
59.079	1.56241	47.0	6.86	19.2
59.089	1.56216	36.0	6.00	19.2
59.099	1.56192	39.0	6.24	19.2
59.109	1.56168	46.0	6.78	19.2
59.119	1.56143	33.0	5.74	19.2
59.129	1.56119	45.0	6.71	19.2
59.139	1.56095	41.0	6.40	19.2
59.149	1.56071	45.0	6.71	19.2
59.160	1.56046	58.0	7.62	19.2
59.170	1.56022	50.0	7.07	19.2
59.180	1.55998	43.0	6.56	19.2
59.190	1.55974	43.0	6.56	19.2
59.200	1.55950	39.0	6.24	19.2
59.210	1.55925	29.0	5.39	19.2
59.220	1.55901	46.0	6.78	19.2
59.230	1.55877	40.0	6.32	19.2
59.240	1.55853	47.0	6.86	19.2
59.250	1.55829	47.0	6.86	19.2
59.261	1.55805	33.0	5.74	19.2
59.271	1.55780	31.0	5.57	19.2
59.281	1.55756	52.0	7.21	19.2
59.291	1.55732	47.0	6.86	19.2
59.301	1.55708	36.0	6.00	19.2
59.311	1.55684	46.0	6.78	19.2
59.321	1.55660	54.0	7.35	19.2
59.331	1.55636	47.0	6.86	19.2
59.341	1.55612	39.0	6.24	19.2
59.351	1.55588	45.0	6.71	19.2
59.362	1.55564	32.0	5.66	19.2
59.372	1.55540	38.0	6.16	19.2
59.382	1.55515	38.0	6.16	19.2
59.392	1.55491	41.0	6.40	19.2
59.402	1.55467	34.0	5.83	19.2
59.412	1.55443	36.0	6.00	19.2
59.422	1.55419	41.0	6.40	19.2
59.432	1.55395	38.0	6.16	19.2
59.442	1.55371	42.0	6.48	19.2
59.452	1.55347	54.0	7.35	19.2
59.463	1.55323	35.0	5.92	19.2
59.473	1.55299	38.0	6.16	19.2
59.483	1.55276	43.0	6.56	19.2
59.493	1.55252	47.0	6.86	19.2
59.503	1.55228	38.0	6.16	19.2
59.513	1.55204	50.0	7.07	19.2
59.523	1.55180	41.0	6.40	19.2

59.533	1.55156	48.0	6.93	19.2
59.543	1.55132	40.0	6.32	19.2
59.553	1.55108	40.0	6.32	19.2
59.564	1.55084	51.0	7.14	19.2
59.574	1.55060	31.0	5.57	19.2
59.584	1.55036	39.0	6.24	19.2
59.594	1.55013	31.0	5.57	19.2
59.604	1.54989	38.0	6.16	19.2
59.614	1.54965	41.0	6.40	19.2
59.624	1.54941	26.0	5.10	19.2
59.634	1.54917	49.0	7.00	19.2
59.644	1.54893	40.0	6.32	19.2
59.654	1.54870	29.0	5.39	19.2
59.665	1.54846	35.0	5.92	19.2
59.675	1.54822	36.0	6.00	19.2
59.685	1.54798	38.0	6.16	19.2
59.695	1.54774	41.0	6.40	19.2
59.705	1.54751	26.0	5.10	19.2
59.715	1.54727	39.0	6.24	19.2
59.725	1.54703	37.0	6.08	19.2
59.735	1.54679	44.0	6.63	19.2
59.745	1.54656	40.0	6.32	19.2
59.755	1.54632	38.0	6.16	19.2
59.766	1.54608	43.0	6.56	19.2
59.776	1.54585	36.0	6.00	19.2
59.786	1.54561	40.0	6.32	19.2
59.796	1.54537	43.0	6.56	19.2
59.806	1.54513	34.0	5.83	19.2
59.816	1.54490	39.0	6.24	19.2
59.826	1.54466	47.0	6.86	19.2
59.836	1.54442	42.0	6.48	19.2
59.846	1.54419	36.0	6.00	19.2
59.856	1.54395	44.0	6.63	19.2
59.867	1.54372	44.0	6.63	19.2
59.877	1.54348	36.0	6.00	19.2
59.887	1.54324	40.0	6.32	19.2
59.897	1.54301	32.0	5.66	19.2
59.907	1.54277	40.0	6.32	19.2
59.917	1.54253	37.0	6.08	19.2
59.927	1.54230	44.0	6.63	19.2
59.937	1.54206	36.0	6.00	19.2
59.947	1.54183	40.0	6.32	19.2
59.957	1.54159	34.0	5.83	19.2
59.968	1.54136	42.0	6.48	19.2
59.978	1.54112	41.0	6.40	19.2
59.988	1.54089	31.0	5.57	19.2
59.998	1.54065	40.0	6.32	19.2
60.008	1.54042	36.0	6.00	19.2
60.018	1.54018	36.0	6.00	19.2
60.028	1.53995	36.0	6.00	19.2

60.038	1.53971	40.0	6.32	19.2
60.048	1.53948	41.0	6.40	19.2
60.058	1.53924	36.0	6.00	19.2
60.069	1.53901	35.0	5.92	19.2
60.079	1.53877	36.0	6.00	19.2
60.089	1.53854	34.0	5.83	19.2
60.099	1.53830	39.0	6.24	19.2
60.109	1.53807	35.0	5.92	19.2
60.119	1.53783	35.0	5.92	19.2
60.129	1.53760	43.0	6.56	19.2
60.139	1.53737	38.0	6.16	19.2
60.149	1.53713	28.0	5.29	19.2
60.159	1.53690	39.0	6.24	19.2
60.170	1.53666	44.0	6.63	19.2
60.180	1.53643	37.0	6.08	19.2
60.190	1.53620	36.0	6.00	19.2
60.200	1.53596	35.0	5.92	19.2
60.210	1.53573	32.0	5.66	19.2
60.220	1.53550	38.0	6.16	19.2
60.230	1.53526	36.0	6.00	19.2
60.240	1.53503	37.0	6.08	19.2
60.250	1.53480	47.0	6.86	19.2
60.260	1.53456	41.0	6.40	19.2
60.270	1.53433	41.0	6.40	19.2
60.281	1.53410	32.0	5.66	19.2
60.291	1.53387	55.0	7.42	19.2
60.301	1.53363	43.0	6.56	19.2
60.311	1.53340	36.0	6.00	19.2
60.321	1.53317	26.0	5.10	19.2
60.331	1.53293	42.0	6.48	19.2
60.341	1.53270	34.0	5.83	19.2
60.351	1.53247	36.0	6.00	19.2
60.361	1.53224	39.0	6.24	19.2
60.371	1.53201	34.0	5.83	19.2
60.382	1.53177	46.0	6.78	19.2
60.392	1.53154	27.0	5.20	19.2
60.402	1.53131	34.0	5.83	19.2
60.412	1.53108	32.0	5.66	19.2
60.422	1.53085	42.0	6.48	19.2
60.432	1.53061	37.0	6.08	19.2
60.442	1.53038	41.0	6.40	19.2
60.452	1.53015	39.0	6.24	19.2
60.462	1.52992	34.0	5.83	19.2
60.472	1.52969	39.0	6.24	19.2
60.483	1.52946	30.0	5.48	19.2
60.493	1.52923	43.0	6.56	19.2
60.503	1.52900	38.0	6.16	19.2
60.513	1.52876	42.0	6.48	19.2
60.523	1.52853	40.0	6.32	19.2
60.533	1.52830	34.0	5.83	19.2

60.543	1.52807	34.0	5.83	19.2
60.553	1.52784	34.0	5.83	19.2
60.563	1.52761	45.0	6.71	19.2
60.573	1.52738	39.0	6.24	19.2
60.584	1.52715	34.0	5.83	19.2
60.594	1.52692	38.0	6.16	19.2
60.604	1.52669	45.0	6.71	19.2
60.614	1.52646	34.0	5.83	19.2
60.624	1.52623	51.0	7.14	19.2
60.634	1.52600	39.0	6.24	19.2
60.644	1.52577	49.0	7.00	19.2
60.654	1.52554	38.0	6.16	19.2
60.664	1.52531	36.0	6.00	19.2
60.674	1.52508	31.0	5.57	19.2
60.685	1.52485	35.0	5.92	19.2
60.695	1.52462	38.0	6.16	19.2
60.705	1.52439	44.0	6.63	19.2
60.715	1.52416	38.0	6.16	19.2
60.725	1.52393	36.0	6.00	19.2
60.735	1.52370	36.0	6.00	19.2
60.745	1.52347	43.0	6.56	19.2
60.755	1.52324	33.0	5.74	19.2
60.765	1.52302	36.0	6.00	19.2
60.775	1.52279	44.0	6.63	19.2
60.786	1.52256	49.0	7.00	19.2
60.796	1.52233	28.0	5.29	19.2
60.806	1.52210	33.0	5.74	19.2
60.816	1.52187	34.0	5.83	19.2
60.826	1.52164	32.0	5.66	19.2
60.836	1.52141	38.0	6.16	19.2
60.846	1.52119	41.0	6.40	19.2
60.856	1.52096	38.0	6.16	19.2
60.866	1.52073	47.0	6.86	19.2
60.876	1.52050	28.0	5.29	19.2
60.887	1.52027	41.0	6.40	19.2
60.897	1.52005	32.0	5.66	19.2
60.907	1.51982	49.0	7.00	19.2
60.917	1.51959	38.0	6.16	19.2
60.927	1.51936	36.0	6.00	19.2
60.937	1.51913	35.0	5.92	19.2
60.947	1.51891	28.0	5.29	19.2
60.957	1.51868	41.0	6.40	19.2
60.967	1.51845	31.0	5.57	19.2
60.977	1.51823	36.0	6.00	19.2
60.988	1.51800	40.0	6.32	19.2
60.998	1.51777	30.0	5.48	19.2
61.008	1.51754	46.0	6.78	19.2
61.018	1.51732	33.0	5.74	19.2
61.028	1.51709	35.0	5.92	19.2
61.038	1.51686	33.0	5.74	19.2

61.048	1.51664	43.0	6.56	19.2
61.058	1.51641	46.0	6.78	19.2
61.068	1.51618	34.0	5.83	19.2
61.078	1.51596	32.0	5.66	19.2
61.089	1.51573	39.0	6.24	19.2
61.099	1.51550	35.0	5.92	19.2
61.109	1.51528	37.0	6.08	19.2
61.119	1.51505	29.0	5.39	19.2
61.129	1.51483	26.0	5.10	19.2
61.139	1.51460	31.0	5.57	19.2
61.149	1.51437	24.0	4.90	19.2
61.159	1.51415	28.0	5.29	19.2
61.169	1.51392	28.0	5.29	19.2
61.179	1.51370	34.0	5.83	19.2
61.190	1.51347	32.0	5.66	19.2
61.200	1.51324	37.0	6.08	19.2
61.210	1.51302	37.0	6.08	19.2
61.220	1.51279	30.0	5.48	19.2
61.230	1.51257	29.0	5.39	19.2
61.240	1.51234	43.0	6.56	19.2
61.250	1.51212	32.0	5.66	19.2
61.260	1.51189	36.0	6.00	19.2
61.270	1.51167	32.0	5.66	19.2
61.280	1.51144	40.0	6.32	19.2
61.291	1.51122	35.0	5.92	19.2
61.301	1.51099	30.0	5.48	19.2
61.311	1.51077	25.0	5.00	19.2
61.321	1.51054	38.0	6.16	19.2
61.331	1.51032	36.0	6.00	19.2
61.341	1.51010	41.0	6.40	19.2
61.351	1.50987	38.0	6.16	19.2
61.361	1.50965	29.0	5.39	19.2
61.371	1.50942	36.0	6.00	19.2
61.381	1.50920	29.0	5.39	19.2
61.392	1.50897	40.0	6.32	19.2
61.402	1.50875	34.0	5.83	19.2
61.412	1.50853	40.0	6.32	19.2
61.422	1.50830	28.0	5.29	19.2
61.432	1.50808	34.0	5.83	19.2
61.442	1.50786	32.0	5.66	19.2
61.452	1.50763	31.0	5.57	19.2
61.462	1.50741	27.0	5.20	19.2
61.472	1.50718	36.0	6.00	19.2
61.482	1.50696	29.0	5.39	19.2
61.493	1.50674	34.0	5.83	19.2
61.503	1.50651	35.0	5.92	19.2
61.513	1.50629	34.0	5.83	19.2
61.523	1.50607	27.0	5.20	19.2
61.533	1.50585	35.0	5.92	19.2
61.543	1.50562	42.0	6.48	19.2

61.553	1.50540	34.0	5.83	19.2
61.563	1.50518	39.0	6.24	19.2
61.573	1.50495	35.0	5.92	19.2
61.583	1.50473	30.0	5.48	19.2
61.593	1.50451	40.0	6.32	19.2
61.604	1.50429	32.0	5.66	19.2
61.614	1.50406	34.0	5.83	19.2
61.624	1.50384	34.0	5.83	19.2
61.634	1.50362	22.0	4.69	19.2
61.644	1.50340	29.0	5.39	19.2
61.654	1.50318	28.0	5.29	19.2
61.664	1.50295	27.0	5.20	19.2
61.674	1.50273	25.0	5.00	19.2
61.684	1.50251	33.0	5.74	19.2
61.694	1.50229	32.0	5.66	19.2
61.705	1.50207	24.0	4.90	19.2
61.715	1.50185	34.0	5.83	19.2
61.725	1.50162	22.0	4.69	19.2
61.735	1.50140	31.0	5.57	19.2
61.745	1.50118	30.0	5.48	19.2
61.755	1.50096	30.0	5.48	19.2
61.765	1.50074	26.0	5.10	19.2
61.775	1.50052	35.0	5.92	19.2
61.785	1.50030	26.0	5.10	19.2
61.795	1.50008	35.0	5.92	19.2
61.806	1.49985	38.0	6.16	19.2
61.816	1.49963	28.0	5.29	19.2
61.826	1.49941	28.0	5.29	19.2
61.836	1.49919	33.0	5.74	19.2
61.846	1.49897	28.0	5.29	19.2
61.856	1.49875	31.0	5.57	19.2
61.866	1.49853	21.0	4.58	19.2
61.876	1.49831	30.0	5.48	19.2
61.886	1.49809	28.0	5.29	19.2
61.896	1.49787	40.0	6.32	19.2
61.907	1.49765	34.0	5.83	19.2
61.917	1.49743	24.0	4.90	19.2
61.927	1.49721	29.0	5.39	19.2
61.937	1.49699	27.0	5.20	19.2
61.947	1.49677	35.0	5.92	19.2
61.957	1.49655	24.0	4.90	19.2
61.967	1.49633	34.0	5.83	19.2
61.977	1.49611	37.0	6.08	19.2
61.987	1.49589	43.0	6.56	19.2
61.997	1.49567	32.0	5.66	19.2
62.008	1.49545	29.0	5.39	19.2
62.018	1.49523	30.0	5.48	19.2
62.028	1.49501	32.0	5.66	19.2
62.038	1.49480	34.0	5.83	19.2
62.048	1.49458	33.0	5.74	19.2

62.058	1.49436	26.0	5.10	19.2
62.068	1.49414	23.0	4.80	19.2
62.078	1.49392	27.0	5.20	19.2
62.088	1.49370	31.0	5.57	19.2
62.098	1.49348	33.0	5.74	19.2
62.109	1.49326	31.0	5.57	19.2
62.119	1.49305	26.0	5.10	19.2
62.129	1.49283	29.0	5.39	19.2
62.139	1.49261	33.0	5.74	19.2
62.149	1.49239	36.0	6.00	19.2
62.159	1.49217	29.0	5.39	19.2
62.169	1.49195	15.0	3.87	19.2
62.179	1.49174	29.0	5.39	19.2
62.189	1.49152	28.0	5.29	19.2
62.199	1.49130	28.0	5.29	19.2
62.210	1.49108	28.0	5.29	19.2
62.220	1.49086	32.0	5.66	19.2
62.230	1.49065	29.0	5.39	19.2
62.240	1.49043	25.0	5.00	19.2
62.250	1.49021	32.0	5.66	19.2
62.260	1.48999	25.0	5.00	19.2
62.270	1.48978	29.0	5.39	19.2
62.280	1.48956	34.0	5.83	19.2
62.290	1.48934	31.0	5.57	19.2
62.300	1.48912	33.0	5.74	19.2
62.311	1.48891	32.0	5.66	19.2
62.321	1.48869	33.0	5.74	19.2
62.331	1.48847	32.0	5.66	19.2
62.341	1.48826	29.0	5.39	19.2
62.351	1.48804	27.0	5.20	19.2
62.361	1.48782	33.0	5.74	19.2
62.371	1.48761	33.0	5.74	19.2
62.381	1.48739	34.0	5.83	19.2
62.391	1.48717	34.0	5.83	19.2
62.401	1.48696	25.0	5.00	19.2
62.412	1.48674	37.0	6.08	19.2
62.422	1.48652	35.0	5.92	19.2
62.432	1.48631	21.0	4.58	19.2
62.442	1.48609	22.0	4.69	19.2
62.452	1.48588	42.0	6.48	19.2
62.462	1.48566	28.0	5.29	19.2
62.472	1.48544	28.0	5.29	19.2
62.482	1.48523	35.0	5.92	19.2
62.492	1.48501	27.0	5.20	19.2
62.502	1.48480	25.0	5.00	19.2
62.513	1.48458	32.0	5.66	19.2
62.523	1.48437	34.0	5.83	19.2
62.533	1.48415	36.0	6.00	19.2
62.543	1.48394	30.0	5.48	19.2
62.553	1.48372	28.0	5.29	19.2

62.563	1.48350	28.0	5.29	19.2
62.573	1.48329	30.0	5.48	19.2
62.583	1.48307	31.0	5.57	19.2
62.593	1.48286	28.0	5.29	19.2
62.603	1.48264	34.0	5.83	19.2
62.614	1.48243	30.0	5.48	19.2
62.624	1.48221	23.0	4.80	19.2
62.634	1.48200	35.0	5.92	19.2
62.644	1.48179	27.0	5.20	19.2
62.654	1.48157	37.0	6.08	19.2
62.664	1.48136	28.0	5.29	19.2
62.674	1.48114	37.0	6.08	19.2
62.684	1.48093	28.0	5.29	19.2
62.694	1.48071	31.0	5.57	19.2
62.704	1.48050	33.0	5.74	19.2
62.715	1.48029	25.0	5.00	19.2
62.725	1.48007	24.0	4.90	19.2
62.735	1.47986	29.0	5.39	19.2
62.745	1.47964	29.0	5.39	19.2
62.755	1.47943	24.0	4.90	19.2
62.765	1.47922	29.0	5.39	19.2
62.775	1.47900	32.0	5.66	19.2
62.785	1.47879	24.0	4.90	19.2
62.795	1.47857	24.0	4.90	19.2
62.805	1.47836	24.0	4.90	19.2
62.816	1.47815	38.0	6.16	19.2
62.826	1.47793	27.0	5.20	19.2
62.836	1.47772	21.0	4.58	19.2
62.846	1.47751	32.0	5.66	19.2
62.856	1.47730	26.0	5.10	19.2
62.866	1.47708	31.0	5.57	19.2
62.876	1.47687	25.0	5.00	19.2
62.886	1.47666	28.0	5.29	19.2
62.896	1.47644	29.0	5.39	19.2
62.906	1.47623	28.0	5.29	19.2
62.916	1.47602	30.0	5.48	19.2
62.927	1.47581	15.0	3.87	19.2
62.937	1.47559	25.0	5.00	19.2
62.947	1.47538	26.0	5.10	19.2
62.957	1.47517	37.0	6.08	19.2
62.967	1.47496	26.0	5.10	19.2
62.977	1.47474	29.0	5.39	19.2
62.987	1.47453	32.0	5.66	19.2
62.997	1.47432	28.0	5.29	19.2
63.007	1.47411	36.0	6.00	19.2
63.017	1.47390	32.0	5.66	19.2
63.028	1.47368	36.0	6.00	19.2
63.038	1.47347	25.0	5.00	19.2
63.048	1.47326	23.0	4.80	19.2
63.058	1.47305	37.0	6.08	19.2

63.068	1.47284	25.0	5.00	19.2
63.078	1.47263	32.0	5.66	19.2
63.088	1.47241	27.0	5.20	19.2
63.098	1.47220	24.0	4.90	19.2
63.108	1.47199	28.0	5.29	19.2
63.118	1.47178	25.0	5.00	19.2
63.129	1.47157	24.0	4.90	19.2
63.139	1.47136	37.0	6.08	19.2
63.149	1.47115	21.0	4.58	19.2
63.159	1.47094	24.0	4.90	19.2
63.169	1.47072	30.0	5.48	19.2
63.179	1.47051	27.0	5.20	19.2
63.189	1.47030	25.0	5.00	19.2
63.199	1.47009	22.0	4.69	19.2
63.209	1.46988	27.0	5.20	19.2
63.219	1.46967	24.0	4.90	19.2
63.230	1.46946	23.0	4.80	19.2
63.240	1.46925	31.0	5.57	19.2
63.250	1.46904	22.0	4.69	19.2
63.260	1.46883	18.0	4.24	19.2
63.270	1.46862	23.0	4.80	19.2
63.280	1.46841	22.0	4.69	19.2
63.290	1.46820	34.0	5.83	19.2
63.300	1.46799	26.0	5.10	19.2
63.310	1.46778	20.0	4.47	19.2
63.320	1.46757	33.0	5.74	19.2
63.331	1.46736	25.0	5.00	19.2
63.341	1.46715	23.0	4.80	19.2
63.351	1.46694	32.0	5.66	19.2
63.361	1.46673	25.0	5.00	19.2
63.371	1.46652	19.0	4.36	19.2
63.381	1.46631	25.0	5.00	19.2
63.391	1.46610	25.0	5.00	19.2
63.401	1.46590	23.0	4.80	19.2
63.411	1.46569	21.0	4.58	19.2
63.421	1.46548	24.0	4.90	19.2
63.432	1.46527	24.0	4.90	19.2
63.442	1.46506	28.0	5.29	19.2
63.452	1.46485	28.0	5.29	19.2
63.462	1.46464	26.0	5.10	19.2
63.472	1.46443	16.0	4.00	19.2
63.482	1.46422	24.0	4.90	19.2
63.492	1.46402	19.0	4.36	19.2
63.502	1.46381	37.0	6.08	19.2
63.512	1.46360	19.0	4.36	19.2
63.522	1.46339	28.0	5.29	19.2
63.533	1.46318	35.0	5.92	19.2
63.543	1.46297	19.0	4.36	19.2
63.553	1.46277	25.0	5.00	19.2
63.563	1.46256	20.0	4.47	19.2

63.573	1.46235	30.0	5.48	19.2
63.583	1.46214	23.0	4.80	19.2
63.593	1.46193	25.0	5.00	19.2
63.603	1.46173	35.0	5.92	19.2
63.613	1.46152	23.0	4.80	19.2
63.623	1.46131	26.0	5.10	19.2
63.634	1.46110	18.0	4.24	19.2
63.644	1.46090	21.0	4.58	19.2
63.654	1.46069	30.0	5.48	19.2
63.664	1.46048	27.0	5.20	19.2
63.674	1.46027	17.0	4.12	19.2
63.684	1.46007	19.0	4.36	19.2
63.694	1.45986	28.0	5.29	19.2
63.704	1.45965	23.0	4.80	19.2
63.714	1.45944	23.0	4.80	19.2
63.724	1.45924	27.0	5.20	19.2
63.735	1.45903	31.0	5.57	19.2
63.745	1.45882	25.0	5.00	19.2
63.755	1.45862	24.0	4.90	19.2
63.765	1.45841	23.0	4.80	19.2
63.775	1.45820	26.0	5.10	19.2
63.785	1.45800	19.0	4.36	19.2
63.795	1.45779	23.0	4.80	19.2
63.805	1.45758	21.0	4.58	19.2
63.815	1.45738	23.0	4.80	19.2
63.825	1.45717	25.0	5.00	19.2
63.836	1.45697	23.0	4.80	19.2
63.846	1.45676	26.0	5.10	19.2
63.856	1.45655	27.0	5.20	19.2
63.866	1.45635	24.0	4.90	19.2
63.876	1.45614	27.0	5.20	19.2
63.886	1.45594	22.0	4.69	19.2
63.896	1.45573	26.0	5.10	19.2
63.906	1.45552	25.0	5.00	19.2
63.916	1.45532	20.0	4.47	19.2
63.926	1.45511	30.0	5.48	19.2
63.937	1.45491	20.0	4.47	19.2
63.947	1.45470	19.0	4.36	19.2
63.957	1.45450	21.0	4.58	19.2
63.967	1.45429	18.0	4.24	19.2
63.977	1.45409	22.0	4.69	19.2
63.987	1.45388	22.0	4.69	19.2
63.997	1.45368	24.0	4.90	19.2
64.007	1.45347	29.0	5.39	19.2
64.017	1.45327	22.0	4.69	19.2
64.027	1.45306	21.0	4.58	19.2
64.038	1.45286	18.0	4.24	19.2
64.048	1.45265	13.0	3.61	19.2
64.058	1.45245	26.0	5.10	19.2
64.068	1.45224	29.0	5.39	19.2

64.078	1.45204	16.0	4.00	19.2
64.088	1.45183	27.0	5.20	19.2
64.098	1.45163	26.0	5.10	19.2
64.108	1.45142	20.0	4.47	19.2
64.118	1.45122	27.0	5.20	19.2
64.128	1.45102	13.0	3.61	19.2
64.139	1.45081	22.0	4.69	19.2
64.149	1.45061	24.0	4.90	19.2
64.159	1.45040	26.0	5.10	19.2
64.169	1.45020	21.0	4.58	19.2
64.179	1.45000	28.0	5.29	19.2
64.189	1.44979	28.0	5.29	19.2
64.199	1.44959	20.0	4.47	19.2
64.209	1.44939	31.0	5.57	19.2
64.219	1.44918	21.0	4.58	19.2
64.229	1.44898	30.0	5.48	19.2
64.239	1.44878	30.0	5.48	19.2
64.250	1.44857	17.0	4.12	19.2
64.260	1.44837	25.0	5.00	19.2
64.270	1.44817	17.0	4.12	19.2
64.280	1.44796	24.0	4.90	19.2
64.290	1.44776	20.0	4.47	19.2
64.300	1.44756	18.0	4.24	19.2
64.310	1.44735	20.0	4.47	19.2
64.320	1.44715	25.0	5.00	19.2
64.330	1.44695	22.0	4.69	19.2
64.340	1.44674	22.0	4.69	19.2
64.351	1.44654	28.0	5.29	19.2
64.361	1.44634	22.0	4.69	19.2
64.371	1.44614	20.0	4.47	19.2
64.381	1.44593	31.0	5.57	19.2
64.391	1.44573	22.0	4.69	19.2
64.401	1.44553	23.0	4.80	19.2
64.411	1.44533	26.0	5.10	19.2
64.421	1.44513	24.0	4.90	19.2
64.431	1.44492	22.0	4.69	19.2
64.441	1.44472	24.0	4.90	19.2
64.452	1.44452	29.0	5.39	19.2
64.462	1.44432	17.0	4.12	19.2
64.472	1.44412	26.0	5.10	19.2
64.482	1.44391	21.0	4.58	19.2
64.492	1.44371	14.0	3.74	19.2
64.502	1.44351	23.0	4.80	19.2
64.512	1.44331	25.0	5.00	19.2
64.522	1.44311	23.0	4.80	19.2
64.532	1.44291	21.0	4.58	19.2
64.542	1.44270	28.0	5.29	19.2
64.553	1.44250	27.0	5.20	19.2
64.563	1.44230	10.0	3.16	19.2
64.573	1.44210	17.0	4.12	19.2

64.583	1.44190	27.0	5.20	19.2
64.593	1.44170	22.0	4.69	19.2
64.603	1.44150	26.0	5.10	19.2
64.613	1.44130	29.0	5.39	19.2
64.623	1.44110	24.0	4.90	19.2
64.633	1.44089	23.0	4.80	19.2
64.643	1.44069	23.0	4.80	19.2
64.654	1.44049	32.0	5.66	19.2
64.664	1.44029	33.0	5.74	19.2
64.674	1.44009	25.0	5.00	19.2
64.684	1.43989	25.0	5.00	19.2
64.694	1.43969	21.0	4.58	19.2
64.704	1.43949	21.0	4.58	19.2
64.714	1.43929	27.0	5.20	19.2
64.724	1.43909	19.0	4.36	19.2
64.734	1.43889	25.0	5.00	19.2
64.744	1.43869	14.0	3.74	19.2
64.755	1.43849	20.0	4.47	19.2
64.765	1.43829	22.0	4.69	19.2
64.775	1.43809	26.0	5.10	19.2
64.785	1.43789	17.0	4.12	19.2
64.795	1.43769	22.0	4.69	19.2
64.805	1.43749	20.0	4.47	19.2
64.815	1.43729	19.0	4.36	19.2
64.825	1.43709	18.0	4.24	19.2
64.835	1.43689	16.0	4.00	19.2
64.845	1.43669	25.0	5.00	19.2
64.856	1.43649	18.0	4.24	19.2
64.866	1.43630	17.0	4.12	19.2
64.876	1.43610	21.0	4.58	19.2
64.886	1.43590	21.0	4.58	19.2
64.896	1.43570	27.0	5.20	19.2
64.906	1.43550	29.0	5.39	19.2
64.916	1.43530	25.0	5.00	19.2
64.926	1.43510	17.0	4.12	19.2
64.936	1.43490	31.0	5.57	19.2
64.946	1.43470	20.0	4.47	19.2
64.957	1.43450	22.0	4.69	19.2
64.967	1.43431	26.0	5.10	19.2
64.977	1.43411	22.0	4.69	19.2
64.987	1.43391	24.0	4.90	19.2
64.997	1.43371	22.0	4.69	19.2
65.007	1.43351	20.0	4.47	19.2
65.017	1.43331	28.0	5.29	19.2
65.027	1.43312	26.0	5.10	19.2
65.037	1.43292	12.0	3.46	19.2
65.047	1.43272	20.0	4.47	19.2
65.058	1.43252	30.0	5.48	19.2
65.068	1.43232	24.0	4.90	19.2
65.078	1.43213	20.0	4.47	19.2

65.088	1.43193	20.0	4.47	19.2
65.098	1.43173	28.0	5.29	19.2
65.108	1.43153	18.0	4.24	19.2
65.118	1.43134	17.0	4.12	19.2
65.128	1.43114	21.0	4.58	19.2
65.138	1.43094	18.0	4.24	19.2
65.148	1.43074	16.0	4.00	19.2
65.159	1.43055	26.0	5.10	19.2
65.169	1.43035	19.0	4.36	19.2
65.179	1.43015	18.0	4.24	19.2
65.189	1.42995	15.0	3.87	19.2
65.199	1.42976	16.0	4.00	19.2
65.209	1.42956	15.0	3.87	19.2
65.219	1.42936	11.0	3.32	19.2
65.229	1.42917	22.0	4.69	19.2
65.239	1.42897	25.0	5.00	19.2
65.249	1.42877	18.0	4.24	19.2
65.260	1.42858	25.0	5.00	19.2
65.270	1.42838	20.0	4.47	19.2
65.280	1.42818	19.0	4.36	19.2
65.290	1.42799	19.0	4.36	19.2
65.300	1.42779	21.0	4.58	19.2
65.310	1.42759	20.0	4.47	19.2
65.320	1.42740	16.0	4.00	19.2
65.330	1.42720	22.0	4.69	19.2
65.340	1.42700	18.0	4.24	19.2
65.350	1.42681	26.0	5.10	19.2
65.361	1.42661	25.0	5.00	19.2
65.371	1.42642	25.0	5.00	19.2
65.381	1.42622	22.0	4.69	19.2
65.391	1.42603	21.0	4.58	19.2
65.401	1.42583	19.0	4.36	19.2
65.411	1.42563	14.0	3.74	19.2
65.421	1.42544	27.0	5.20	19.2
65.431	1.42524	12.0	3.46	19.2
65.441	1.42505	14.0	3.74	19.2
65.451	1.42485	26.0	5.10	19.2
65.462	1.42466	20.0	4.47	19.2
65.472	1.42446	23.0	4.80	19.2
65.482	1.42427	27.0	5.20	19.2
65.492	1.42407	23.0	4.80	19.2
65.502	1.42388	12.0	3.46	19.2
65.512	1.42368	18.0	4.24	19.2
65.522	1.42349	17.0	4.12	19.2
65.532	1.42329	21.0	4.58	19.2
65.542	1.42310	26.0	5.10	19.2
65.552	1.42290	15.0	3.87	19.2
65.562	1.42271	21.0	4.58	19.2
65.573	1.42251	20.0	4.47	19.2
65.583	1.42232	23.0	4.80	19.2

65.593	1.42212	13.0	3.61	19.2
65.603	1.42193	15.0	3.87	19.2
65.613	1.42173	21.0	4.58	19.2
65.623	1.42154	26.0	5.10	19.2
65.633	1.42134	27.0	5.20	19.2
65.643	1.42115	19.0	4.36	19.2
65.653	1.42096	20.0	4.47	19.2
65.663	1.42076	19.0	4.36	19.2
65.674	1.42057	19.0	4.36	19.2
65.684	1.42037	22.0	4.69	19.2
65.694	1.42018	21.0	4.58	19.2
65.704	1.41999	25.0	5.00	19.2
65.714	1.41979	18.0	4.24	19.2
65.724	1.41960	17.0	4.12	19.2
65.734	1.41941	17.0	4.12	19.2
65.744	1.41921	24.0	4.90	19.2
65.754	1.41902	23.0	4.80	19.2
65.764	1.41882	17.0	4.12	19.2
65.775	1.41863	21.0	4.58	19.2
65.785	1.41844	14.0	3.74	19.2
65.795	1.41824	17.0	4.12	19.2
65.805	1.41805	22.0	4.69	19.2
65.815	1.41786	10.0	3.16	19.2
65.825	1.41767	16.0	4.00	19.2
65.835	1.41747	18.0	4.24	19.2
65.845	1.41728	16.0	4.00	19.2
65.855	1.41709	19.0	4.36	19.2
65.865	1.41689	20.0	4.47	19.2
65.876	1.41670	24.0	4.90	19.2
65.886	1.41651	31.0	5.57	19.2
65.896	1.41632	20.0	4.47	19.2
65.906	1.41612	20.0	4.47	19.2
65.916	1.41593	15.0	3.87	19.2
65.926	1.41574	13.0	3.61	19.2
65.936	1.41555	19.0	4.36	19.2
65.946	1.41535	19.0	4.36	19.2
65.956	1.41516	20.0	4.47	19.2
65.966	1.41497	16.0	4.00	19.2
65.977	1.41478	14.0	3.74	19.2
65.987	1.41458	15.0	3.87	19.2
65.997	1.41439	13.0	3.61	19.2
66.007	1.41420	11.0	3.32	19.2
66.017	1.41401	15.0	3.87	19.2
66.027	1.41382	20.0	4.47	19.2
66.037	1.41363	17.0	4.12	19.2
66.047	1.41343	22.0	4.69	19.2
66.057	1.41324	30.0	5.48	19.2
66.067	1.41305	20.0	4.47	19.2
66.078	1.41286	16.0	4.00	19.2
66.088	1.41267	19.0	4.36	19.2

66.098	1.41248	18.0	4.24	19.2
66.108	1.41228	18.0	4.24	19.2
66.118	1.41209	17.0	4.12	19.2
66.128	1.41190	22.0	4.69	19.2
66.138	1.41171	19.0	4.36	19.2
66.148	1.41152	21.0	4.58	19.2
66.158	1.41133	23.0	4.80	19.2
66.168	1.41114	17.0	4.12	19.2
66.179	1.41095	9.00	3.00	19.2
66.189	1.41076	15.0	3.87	19.2
66.199	1.41057	19.0	4.36	19.2
66.209	1.41038	26.0	5.10	19.2
66.219	1.41018	19.0	4.36	19.2
66.229	1.40999	19.0	4.36	19.2
66.239	1.40980	18.0	4.24	19.2
66.249	1.40961	22.0	4.69	19.2
66.259	1.40942	17.0	4.12	19.2
66.269	1.40923	16.0	4.00	19.2
66.280	1.40904	22.0	4.69	19.2
66.290	1.40885	15.0	3.87	19.2
66.300	1.40866	13.0	3.61	19.2
66.310	1.40847	24.0	4.90	19.2
66.320	1.40828	21.0	4.58	19.2
66.330	1.40809	10.0	3.16	19.2
66.340	1.40790	19.0	4.36	19.2
66.350	1.40771	16.0	4.00	19.2
66.360	1.40752	18.0	4.24	19.2
66.370	1.40733	28.0	5.29	19.2
66.381	1.40714	21.0	4.58	19.2
66.391	1.40695	17.0	4.12	19.2
66.401	1.40676	17.0	4.12	19.2
66.411	1.40658	18.0	4.24	19.2
66.421	1.40639	18.0	4.24	19.2
66.431	1.40620	17.0	4.12	19.2
66.441	1.40601	17.0	4.12	19.2
66.451	1.40582	18.0	4.24	19.2
66.461	1.40563	14.0	3.74	19.2
66.471	1.40544	19.0	4.36	19.2
66.482	1.40525	12.0	3.46	19.2
66.492	1.40506	17.0	4.12	19.2
66.502	1.40487	14.0	3.74	19.2
66.512	1.40468	25.0	5.00	19.2
66.522	1.40450	16.0	4.00	19.2
66.532	1.40431	14.0	3.74	19.2
66.542	1.40412	20.0	4.47	19.2
66.552	1.40393	21.0	4.58	19.2
66.562	1.40374	14.0	3.74	19.2
66.572	1.40355	17.0	4.12	19.2
66.583	1.40336	18.0	4.24	19.2
66.593	1.40318	20.0	4.47	19.2

66.603	1.40299	15.0	3.87	19.2
66.613	1.40280	20.0	4.47	19.2
66.623	1.40261	21.0	4.58	19.2
66.633	1.40242	19.0	4.36	19.2
66.643	1.40224	22.0	4.69	19.2
66.653	1.40205	13.0	3.61	19.2
66.663	1.40186	20.0	4.47	19.2
66.673	1.40167	15.0	3.87	19.2
66.684	1.40148	19.0	4.36	19.2
66.694	1.40130	20.0	4.47	19.2
66.704	1.40111	22.0	4.69	19.2
66.714	1.40092	18.0	4.24	19.2
66.724	1.40073	17.0	4.12	19.2
66.734	1.40055	14.0	3.74	19.2
66.744	1.40036	16.0	4.00	19.2
66.754	1.40017	19.0	4.36	19.2
66.764	1.39998	17.0	4.12	19.2
66.774	1.39980	14.0	3.74	19.2
66.784	1.39961	19.0	4.36	19.2
66.795	1.39942	20.0	4.47	19.2
66.805	1.39924	16.0	4.00	19.2
66.815	1.39905	15.0	3.87	19.2
66.825	1.39886	15.0	3.87	19.2
66.835	1.39867	20.0	4.47	19.2
66.845	1.39849	10.0	3.16	19.2
66.855	1.39830	16.0	4.00	19.2
66.865	1.39811	17.0	4.12	19.2
66.875	1.39793	20.0	4.47	19.2
66.885	1.39774	12.0	3.46	19.2
66.896	1.39755	18.0	4.24	19.2
66.906	1.39737	19.0	4.36	19.2
66.916	1.39718	13.0	3.61	19.2
66.926	1.39700	24.0	4.90	19.2
66.936	1.39681	12.0	3.46	19.2
66.946	1.39662	13.0	3.61	19.2
66.956	1.39644	17.0	4.12	19.2
66.966	1.39625	18.0	4.24	19.2
66.976	1.39606	19.0	4.36	19.2
66.986	1.39588	16.0	4.00	19.2
66.997	1.39569	21.0	4.58	19.2
67.007	1.39551	13.0	3.61	19.2
67.017	1.39532	13.0	3.61	19.2
67.027	1.39514	21.0	4.58	19.2
67.037	1.39495	20.0	4.47	19.2
67.047	1.39476	15.0	3.87	19.2
67.057	1.39458	17.0	4.12	19.2
67.067	1.39439	18.0	4.24	19.2
67.077	1.39421	10.0	3.16	19.2
67.087	1.39402	11.0	3.32	19.2
67.098	1.39384	13.0	3.61	19.2

67.108	1.39365	15.0	3.87	19.2
67.118	1.39347	22.0	4.69	19.2
67.128	1.39328	6.00	2.45	19.2
67.138	1.39310	12.0	3.46	19.2
67.148	1.39291	15.0	3.87	19.2
67.158	1.39273	16.0	4.00	19.2
67.168	1.39254	21.0	4.58	19.2
67.178	1.39236	19.0	4.36	19.2
67.188	1.39217	12.0	3.46	19.2
67.199	1.39199	14.0	3.74	19.2
67.209	1.39180	15.0	3.87	19.2
67.219	1.39162	17.0	4.12	19.2
67.229	1.39143	14.0	3.74	19.2
67.239	1.39125	18.0	4.24	19.2
67.249	1.39107	13.0	3.61	19.2
67.259	1.39088	19.0	4.36	19.2
67.269	1.39070	19.0	4.36	19.2
67.279	1.39051	13.0	3.61	19.2
67.289	1.39033	18.0	4.24	19.2
67.300	1.39014	9.00	3.00	19.2
67.310	1.38996	6.00	2.45	19.2
67.320	1.38978	14.0	3.74	19.2
67.330	1.38959	14.0	3.74	19.2
67.340	1.38941	17.0	4.12	19.2
67.350	1.38923	12.0	3.46	19.2
67.360	1.38904	10.0	3.16	19.2
67.370	1.38886	16.0	4.00	19.2
67.380	1.38867	8.00	2.83	19.2
67.390	1.38849	19.0	4.36	19.2
67.401	1.38831	19.0	4.36	19.2
67.411	1.38812	21.0	4.58	19.2
67.421	1.38794	18.0	4.24	19.2
67.431	1.38776	18.0	4.24	19.2
67.441	1.38757	8.00	2.83	19.2
67.451	1.38739	13.0	3.61	19.2
67.461	1.38721	15.0	3.87	19.2
67.471	1.38702	11.0	3.32	19.2
67.481	1.38684	17.0	4.12	19.2
67.491	1.38666	5.00	2.24	19.2
67.502	1.38648	10.0	3.16	19.2
67.512	1.38629	14.0	3.74	19.2
67.522	1.38611	13.0	3.61	19.2
67.532	1.38593	4.00	2.00	19.2
67.542	1.38574	15.0	3.87	19.2
67.552	1.38556	19.0	4.36	19.2
67.562	1.38538	18.0	4.24	19.2
67.572	1.38520	13.0	3.61	19.2
67.582	1.38501	17.0	4.12	19.2
67.592	1.38483	15.0	3.87	19.2
67.603	1.38465	13.0	3.61	19.2

67.613	1.38447	18.0	4.24	19.2
67.623	1.38429	16.0	4.00	19.2
67.633	1.38410	16.0	4.00	19.2
67.643	1.38392	14.0	3.74	19.2
67.653	1.38374	5.00	2.24	19.2
67.663	1.38356	22.0	4.69	19.2
67.673	1.38338	10.0	3.16	19.2
67.683	1.38319	11.0	3.32	19.2
67.693	1.38301	14.0	3.74	19.2
67.704	1.38283	14.0	3.74	19.2
67.714	1.38265	20.0	4.47	19.2
67.724	1.38247	17.0	4.12	19.2
67.734	1.38228	14.0	3.74	19.2
67.744	1.38210	15.0	3.87	19.2
67.754	1.38192	19.0	4.36	19.2
67.764	1.38174	21.0	4.58	19.2
67.774	1.38156	18.0	4.24	19.2
67.784	1.38138	12.0	3.46	19.2
67.794	1.38120	11.0	3.32	19.2
67.805	1.38102	13.0	3.61	19.2
67.815	1.38083	14.0	3.74	19.2
67.825	1.38065	15.0	3.87	19.2
67.835	1.38047	13.0	3.61	19.2
67.845	1.38029	8.00	2.83	19.2
67.855	1.38011	18.0	4.24	19.2
67.865	1.37993	17.0	4.12	19.2
67.875	1.37975	14.0	3.74	19.2
67.885	1.37957	12.0	3.46	19.2
67.895	1.37939	11.0	3.32	19.2
67.906	1.37921	13.0	3.61	19.2
67.916	1.37903	12.0	3.46	19.2
67.926	1.37885	10.0	3.16	19.2
67.936	1.37867	12.0	3.46	19.2
67.946	1.37849	10.0	3.16	19.2
67.956	1.37831	15.0	3.87	19.2
67.966	1.37813	11.0	3.32	19.2
67.976	1.37795	14.0	3.74	19.2
67.986	1.37777	18.0	4.24	19.2
67.996	1.37759	15.0	3.87	19.2
68.007	1.37741	16.0	4.00	19.2
68.017	1.37723	11.0	3.32	19.2
68.027	1.37705	9.00	3.00	19.2
68.037	1.37687	23.0	4.80	19.2
68.047	1.37669	8.00	2.83	19.2
68.057	1.37651	17.0	4.12	19.2
68.067	1.37633	11.0	3.32	19.2
68.077	1.37615	14.0	3.74	19.2
68.087	1.37597	19.0	4.36	19.2
68.097	1.37579	15.0	3.87	19.2
68.107	1.37561	11.0	3.32	19.2

68.118	1.37543	15.0	3.87	19.2
68.128	1.37525	10.0	3.16	19.2
68.138	1.37507	15.0	3.87	19.2
68.148	1.37489	19.0	4.36	19.2
68.158	1.37471	12.0	3.46	19.2
68.168	1.37453	18.0	4.24	19.2
68.178	1.37435	23.0	4.80	19.2
68.188	1.37418	11.0	3.32	19.2
68.198	1.37400	16.0	4.00	19.2
68.208	1.37382	18.0	4.24	19.2
68.219	1.37364	12.0	3.46	19.2
68.229	1.37346	12.0	3.46	19.2
68.239	1.37328	13.0	3.61	19.2
68.249	1.37310	11.0	3.32	19.2
68.259	1.37292	8.00	2.83	19.2
68.269	1.37275	11.0	3.32	19.2
68.279	1.37257	10.0	3.16	19.2
68.289	1.37239	7.00	2.65	19.2
68.299	1.37221	16.0	4.00	19.2
68.309	1.37203	14.0	3.74	19.2
68.320	1.37185	16.0	4.00	19.2
68.330	1.37168	14.0	3.74	19.2
68.340	1.37150	11.0	3.32	19.2
68.350	1.37132	10.0	3.16	19.2
68.360	1.37114	14.0	3.74	19.2
68.370	1.37096	12.0	3.46	19.2
68.380	1.37079	19.0	4.36	19.2
68.390	1.37061	12.0	3.46	19.2
68.400	1.37043	14.0	3.74	19.2
68.410	1.37025	7.00	2.65	19.2
68.421	1.37008	15.0	3.87	19.2
68.431	1.36990	7.00	2.65	19.2
68.441	1.36972	15.0	3.87	19.2
68.451	1.36954	13.0	3.61	19.2
68.461	1.36937	8.00	2.83	19.2
68.471	1.36919	15.0	3.87	19.2
68.481	1.36901	17.0	4.12	19.2
68.491	1.36883	15.0	3.87	19.2
68.501	1.36866	10.0	3.16	19.2
68.511	1.36848	12.0	3.46	19.2
68.522	1.36830	13.0	3.61	19.2
68.532	1.36812	12.0	3.46	19.2
68.542	1.36795	11.0	3.32	19.2
68.552	1.36777	10.0	3.16	19.2
68.562	1.36759	21.0	4.58	19.2
68.572	1.36742	16.0	4.00	19.2
68.582	1.36724	15.0	3.87	19.2
68.592	1.36706	8.00	2.83	19.2
68.602	1.36689	15.0	3.87	19.2
68.612	1.36671	15.0	3.87	19.2

68.623	1.36653	10.0	3.16	19.2
68.633	1.36636	7.00	2.65	19.2
68.643	1.36618	18.0	4.24	19.2
68.653	1.36601	14.0	3.74	19.2
68.663	1.36583	11.0	3.32	19.2
68.673	1.36565	8.00	2.83	19.2
68.683	1.36548	14.0	3.74	19.2
68.693	1.36530	12.0	3.46	19.2
68.703	1.36512	11.0	3.32	19.2
68.713	1.36495	15.0	3.87	19.2
68.724	1.36477	11.0	3.32	19.2
68.734	1.36460	12.0	3.46	19.2
68.744	1.36442	6.00	2.45	19.2
68.754	1.36424	12.0	3.46	19.2
68.764	1.36407	8.00	2.83	19.2
68.774	1.36389	15.0	3.87	19.2
68.784	1.36372	6.00	2.45	19.2
68.794	1.36354	8.00	2.83	19.2
68.804	1.36337	13.0	3.61	19.2
68.814	1.36319	12.0	3.46	19.2
68.825	1.36302	12.0	3.46	19.2
68.835	1.36284	16.0	4.00	19.2
68.845	1.36267	13.0	3.61	19.2
68.855	1.36249	16.0	4.00	19.2
68.865	1.36232	12.0	3.46	19.2
68.875	1.36214	23.0	4.80	19.2
68.885	1.36196	14.0	3.74	19.2
68.895	1.36179	8.00	2.83	19.2
68.905	1.36161	8.00	2.83	19.2
68.915	1.36144	11.0	3.32	19.2
68.926	1.36127	6.00	2.45	19.2
68.936	1.36109	11.0	3.32	19.2
68.946	1.36092	11.0	3.32	19.2
68.956	1.36074	16.0	4.00	19.2
68.966	1.36057	11.0	3.32	19.2
68.976	1.36039	11.0	3.32	19.2
68.986	1.36022	6.00	2.45	19.2
68.996	1.36004	14.0	3.74	19.2
69.006	1.35987	15.0	3.87	19.2
69.016	1.35969	12.0	3.46	19.2
69.027	1.35952	7.00	2.65	19.2
69.037	1.35935	10.0	3.16	19.2
69.047	1.35917	7.00	2.65	19.2
69.057	1.35900	15.0	3.87	19.2
69.067	1.35882	8.00	2.83	19.2
69.077	1.35865	18.0	4.24	19.2
69.087	1.35848	13.0	3.61	19.2
69.097	1.35830	8.00	2.83	19.2
69.107	1.35813	14.0	3.74	19.2
69.117	1.35795	13.0	3.61	19.2

69.128	1.35778	7.00	2.65	19.2
69.138	1.35761	9.00	3.00	19.2
69.148	1.35743	19.0	4.36	19.2
69.158	1.35726	4.00	2.00	19.2
69.168	1.35709	16.0	4.00	19.2
69.178	1.35691	14.0	3.74	19.2
69.188	1.35674	10.0	3.16	19.2
69.198	1.35657	12.0	3.46	19.2
69.208	1.35639	12.0	3.46	19.2
69.218	1.35622	16.0	4.00	19.2
69.229	1.35605	15.0	3.87	19.2
69.239	1.35587	16.0	4.00	19.2
69.249	1.35570	10.0	3.16	19.2
69.259	1.35553	3.00	1.73	19.2
69.269	1.35535	15.0	3.87	19.2
69.279	1.35518	13.0	3.61	19.2
69.289	1.35501	11.0	3.32	19.2
69.299	1.35484	5.00	2.24	19.2
69.309	1.35466	11.0	3.32	19.2
69.319	1.35449	14.0	3.74	19.2
69.330	1.35432	15.0	3.87	19.2
69.340	1.35414	12.0	3.46	19.2
69.350	1.35397	9.00	3.00	19.2
69.360	1.35380	12.0	3.46	19.2
69.370	1.35363	6.00	2.45	19.2
69.380	1.35345	15.0	3.87	19.2
69.390	1.35328	11.0	3.32	19.2
69.400	1.35311	7.00	2.65	19.2
69.410	1.35294	5.00	2.24	19.2
69.420	1.35277	9.00	3.00	19.2
69.430	1.35259	5.00	2.24	19.2
69.441	1.35242	11.0	3.32	19.2
69.451	1.35225	5.00	2.24	19.2
69.461	1.35208	10.0	3.16	19.2
69.471	1.35191	12.0	3.46	19.2
69.481	1.35173	10.0	3.16	19.2
69.491	1.35156	9.00	3.00	19.2
69.501	1.35139	9.00	3.00	19.2
69.511	1.35122	8.00	2.83	19.2
69.521	1.35105	7.00	2.65	19.2
69.531	1.35088	16.0	4.00	19.2
69.542	1.35070	7.00	2.65	19.2
69.552	1.35053	10.0	3.16	19.2
69.562	1.35036	9.00	3.00	19.2
69.572	1.35019	8.00	2.83	19.2
69.582	1.35002	14.0	3.74	19.2
69.592	1.34985	10.0	3.16	19.2
69.602	1.34968	11.0	3.32	19.2
69.612	1.34951	13.0	3.61	19.2
69.622	1.34933	9.00	3.00	19.2

69.632	1.34916	9.00	3.00	19.2
69.643	1.34899	13.0	3.61	19.2
69.653	1.34882	11.0	3.32	19.2
69.663	1.34865	14.0	3.74	19.2
69.673	1.34848	7.00	2.65	19.2
69.683	1.34831	7.00	2.65	19.2
69.693	1.34814	6.00	2.45	19.2
69.703	1.34797	6.00	2.45	19.2
69.713	1.34780	12.0	3.46	19.2
69.723	1.34763	7.00	2.65	19.2
69.733	1.34746	12.0	3.46	19.2
69.744	1.34729	16.0	4.00	19.2
69.754	1.34712	7.00	2.65	19.2
69.764	1.34695	10.0	3.16	19.2
69.774	1.34678	8.00	2.83	19.2
69.784	1.34660	11.0	3.32	19.2
69.794	1.34643	11.0	3.32	19.2
69.804	1.34626	11.0	3.32	19.2
69.814	1.34609	12.0	3.46	19.2
69.824	1.34592	10.0	3.16	19.2
69.834	1.34575	13.0	3.61	19.2
69.845	1.34558	11.0	3.32	19.2
69.855	1.34542	8.00	2.83	19.2
69.865	1.34525	11.0	3.32	19.2
69.875	1.34508	11.0	3.32	19.2
69.885	1.34491	6.00	2.45	19.2
69.895	1.34474	12.0	3.46	19.2
69.905	1.34457	4.00	2.00	19.2
69.915	1.34440	10.0	3.16	19.2
69.925	1.34423	10.0	3.16	19.2
69.935	1.34406	13.0	3.61	19.2
69.946	1.34389	14.0	3.74	19.2
69.956	1.34372	10.0	3.16	19.2
69.966	1.34355	7.00	2.65	19.2
69.976	1.34338	8.00	2.83	19.2
69.986	1.34321	6.00	2.45	19.2
69.996	1.34304	8.00	2.83	19.2
70.006	1.34287	6.00	2.45	19.2
70.016	1.34271	10.0	3.16	19.2
70.026	1.34254	15.0	3.87	19.2
70.036	1.34237	8.00	2.83	19.2
70.047	1.34220	8.00	2.83	19.2
70.057	1.34203	7.00	2.65	19.2
70.067	1.34186	8.00	2.83	19.2
70.077	1.34169	6.00	2.45	19.2
70.087	1.34152	9.00	3.00	19.2
70.097	1.34136	4.00	2.00	19.2
70.107	1.34119	9.00	3.00	19.2
70.117	1.34102	8.00	2.83	19.2
70.127	1.34085	11.0	3.32	19.2

70.137	1.34068	15.0	3.87	19.2
70.148	1.34051	10.0	3.16	19.2
70.158	1.34034	9.00	3.00	19.2
70.168	1.34018	16.0	4.00	19.2
70.178	1.34001	9.00	3.00	19.2
70.188	1.33984	9.00	3.00	19.2
70.198	1.33967	14.0	3.74	19.2
70.208	1.33950	7.00	2.65	19.2
70.218	1.33934	12.0	3.46	19.2
70.228	1.33917	10.0	3.16	19.2
70.238	1.33900	12.0	3.46	19.2
70.249	1.33883	4.00	2.00	19.2
70.259	1.33867	5.00	2.24	19.2
70.269	1.33850	4.00	2.00	19.2
70.279	1.33833	4.00	2.00	19.2
70.289	1.33816	9.00	3.00	19.2
70.299	1.33800	10.0	3.16	19.2
70.309	1.33783	4.00	2.00	19.2
70.319	1.33766	9.00	3.00	19.2
70.329	1.33749	16.0	4.00	19.2
70.339	1.33733	8.00	2.83	19.2
70.350	1.33716	8.00	2.83	19.2
70.360	1.33699	7.00	2.65	19.2
70.370	1.33682	7.00	2.65	19.2
70.380	1.33666	7.00	2.65	19.2
70.390	1.33649	9.00	3.00	19.2
70.400	1.33632	8.00	2.83	19.2
70.410	1.33616	10.0	3.16	19.2
70.420	1.33599	4.00	2.00	19.2
70.430	1.33582	13.0	3.61	19.2
70.440	1.33566	3.00	1.73	19.2
70.451	1.33549	6.00	2.45	19.2
70.461	1.33532	9.00	3.00	19.2
70.471	1.33516	11.0	3.32	19.2
70.481	1.33499	9.00	3.00	19.2
70.491	1.33482	8.00	2.83	19.2
70.501	1.33466	4.00	2.00	19.2
70.511	1.33449	13.0	3.61	19.2
70.521	1.33432	7.00	2.65	19.2
70.531	1.33416	10.0	3.16	19.2
70.541	1.33399	9.00	3.00	19.2
70.552	1.33382	9.00	3.00	19.2
70.562	1.33366	7.00	2.65	19.2
70.572	1.33349	7.00	2.65	19.2
70.582	1.33333	13.0	3.61	19.2
70.592	1.33316	7.00	2.65	19.2
70.602	1.33299	6.00	2.45	19.2
70.612	1.33283	6.00	2.45	19.2
70.622	1.33266	7.00	2.65	19.2
70.632	1.33250	13.0	3.61	19.2

70.642	1.33233	3.00	1.73	19.2
70.653	1.33217	1.00	1.00	19.2
70.663	1.33200	6.00	2.45	19.2
70.673	1.33183	11.0	3.32	19.2
70.683	1.33167	7.00	2.65	19.2
70.693	1.33150	6.00	2.45	19.2
70.703	1.33134	5.00	2.24	19.2
70.713	1.33117	6.00	2.45	19.2
70.723	1.33101	7.00	2.65	19.2
70.733	1.33084	8.00	2.83	19.2
70.743	1.33068	6.00	2.45	19.2
70.753	1.33051	8.00	2.83	19.2
70.764	1.33035	5.00	2.24	19.2
70.774	1.33018	8.00	2.83	19.2
70.784	1.33002	9.00	3.00	19.2
70.794	1.32985	7.00	2.65	19.2
70.804	1.32969	8.00	2.83	19.2
70.814	1.32952	12.0	3.46	19.2
70.824	1.32936	12.0	3.46	19.2
70.834	1.32919	4.00	2.00	19.2
70.844	1.32903	5.00	2.24	19.2
70.854	1.32886	6.00	2.45	19.2
70.865	1.32870	4.00	2.00	19.2
70.875	1.32853	4.00	2.00	19.2
70.885	1.32837	8.00	2.83	19.2
70.895	1.32820	3.00	1.73	19.2
70.905	1.32804	10.0	3.16	19.2
70.915	1.32788	7.00	2.65	19.2
70.925	1.32771	9.00	3.00	19.2
70.935	1.32755	8.00	2.83	19.2
70.945	1.32738	10.0	3.16	19.2
70.955	1.32722	8.00	2.83	19.2
70.966	1.32705	8.00	2.83	19.2
70.976	1.32689	7.00	2.65	19.2
70.986	1.32673	10.0	3.16	19.2
70.996	1.32656	4.00	2.00	19.2
71.006	1.32640	6.00	2.45	19.2
71.016	1.32623	6.00	2.45	19.2
71.026	1.32607	9.00	3.00	19.2
71.036	1.32591	9.00	3.00	19.2
71.046	1.32574	6.00	2.45	19.2
71.056	1.32558	11.0	3.32	19.2
71.067	1.32542	7.00	2.65	19.2
71.077	1.32525	6.00	2.45	19.2
71.087	1.32509	7.00	2.65	19.2
71.097	1.32493	4.00	2.00	19.2
71.107	1.32476	10.0	3.16	19.2
71.117	1.32460	7.00	2.65	19.2
71.127	1.32444	8.00	2.83	19.2
71.137	1.32427	4.00	2.00	19.2

71.147	1.32411	7.00	2.65	19.2
71.157	1.32395	6.00	2.45	19.2
71.168	1.32378	6.00	2.45	19.2
71.178	1.32362	7.00	2.65	19.2
71.188	1.32346	11.0	3.32	19.2
71.198	1.32329	4.00	2.00	19.2
71.208	1.32313	3.00	1.73	19.2
71.218	1.32297	7.00	2.65	19.2
71.228	1.32281	9.00	3.00	19.2
71.238	1.32264	10.0	3.16	19.2
71.248	1.32248	7.00	2.65	19.2
71.258	1.32232	10.0	3.16	19.2
71.269	1.32216	12.0	3.46	19.2
71.279	1.32199	9.00	3.00	19.2
71.289	1.32183	7.00	2.65	19.2
71.299	1.32167	7.00	2.65	19.2
71.309	1.32151	5.00	2.24	19.2
71.319	1.32134	8.00	2.83	19.2
71.329	1.32118	4.00	2.00	19.2
71.339	1.32102	8.00	2.83	19.2
71.349	1.32086	7.00	2.65	19.2
71.359	1.32069	7.00	2.65	19.2
71.370	1.32053	6.00	2.45	19.2
71.380	1.32037	7.00	2.65	19.2
71.390	1.32021	11.0	3.32	19.2
71.400	1.32005	3.00	1.73	19.2
71.410	1.31988	9.00	3.00	19.2
71.420	1.31972	7.00	2.65	19.2
71.430	1.31956	9.00	3.00	19.2
71.440	1.31940	7.00	2.65	19.2
71.450	1.31924	5.00	2.24	19.2
71.460	1.31908	11.0	3.32	19.2
71.471	1.31891	13.0	3.61	19.2
71.481	1.31875	6.00	2.45	19.2
71.491	1.31859	10.0	3.16	19.2
71.501	1.31843	6.00	2.45	19.2
71.511	1.31827	5.00	2.24	19.2
71.521	1.31811	5.00	2.24	19.2
71.531	1.31795	7.00	2.65	19.2
71.541	1.31778	9.00	3.00	19.2
71.551	1.31762	6.00	2.45	19.2
71.561	1.31746	8.00	2.83	19.2
71.572	1.31730	6.00	2.45	19.2
71.582	1.31714	7.00	2.65	19.2
71.592	1.31698	6.00	2.45	19.2
71.602	1.31682	1.00	1.00	19.2
71.612	1.31666	6.00	2.45	19.2
71.622	1.31650	5.00	2.24	19.2
71.632	1.31634	7.00	2.65	19.2
71.642	1.31617	9.00	3.00	19.2

71.652	1.31601	11.0	3.32	19.2
71.662	1.31585	8.00	2.83	19.2
71.673	1.31569	3.00	1.73	19.2
71.683	1.31553	11.0	3.32	19.2
71.693	1.31537	9.00	3.00	19.2
71.703	1.31521	3.00	1.73	19.2
71.713	1.31505	4.00	2.00	19.2
71.723	1.31489	9.00	3.00	19.2
71.733	1.31473	8.00	2.83	19.2
71.743	1.31457	11.0	3.32	19.2
71.753	1.31441	5.00	2.24	19.2
71.763	1.31425	4.00	2.00	19.2
71.774	1.31409	4.00	2.00	19.2
71.784	1.31393	4.00	2.00	19.2
71.794	1.31377	7.00	2.65	19.2
71.804	1.31361	6.00	2.45	19.2
71.814	1.31345	5.00	2.24	19.2
71.824	1.31329	11.0	3.32	19.2
71.834	1.31313	6.00	2.45	19.2
71.844	1.31297	4.00	2.00	19.2
71.854	1.31281	4.00	2.00	19.2
71.864	1.31265	7.00	2.65	19.2
71.875	1.31249	2.00	1.41	19.2
71.885	1.31233	5.00	2.24	19.2
71.895	1.31217	9.00	3.00	19.2
71.905	1.31201	6.00	2.45	19.2
71.915	1.31185	2.00	1.41	19.2
71.925	1.31169	6.00	2.45	19.2
71.935	1.31154	11.0	3.32	19.2
71.945	1.31138	8.00	2.83	19.2
71.955	1.31122	5.00	2.24	19.2
71.965	1.31106	11.0	3.32	19.2
71.976	1.31090	5.00	2.24	19.2
71.986	1.31074	5.00	2.24	19.2
71.996	1.31058	8.00	2.83	19.2
72.006	1.31042	10.0	3.16	19.2
72.016	1.31026	5.00	2.24	19.2
72.026	1.31010	5.00	2.24	19.2
72.036	1.30994	3.00	1.73	19.2
72.046	1.30979	3.00	1.73	19.2
72.056	1.30963	4.00	2.00	19.2
72.066	1.30947	6.00	2.45	19.2
72.076	1.30931	8.00	2.83	19.2
72.087	1.30915	4.00	2.00	19.2
72.097	1.30899	7.00	2.65	19.2
72.107	1.30883	11.0	3.32	19.2
72.117	1.30868	6.00	2.45	19.2
72.127	1.30852	6.00	2.45	19.2
72.137	1.30836	4.00	2.00	19.2
72.147	1.30820	6.00	2.45	19.2

72.157	1.30804	6.00	2.45	19.2
72.167	1.30788	7.00	2.65	19.2
72.177	1.30773	3.00	1.73	19.2
72.188	1.30757	7.00	2.65	19.2
72.198	1.30741	5.00	2.24	19.2
72.208	1.30725	4.00	2.00	19.2
72.218	1.30709	5.00	2.24	19.2
72.228	1.30694	2.00	1.41	19.2
72.238	1.30678	4.00	2.00	19.2
72.248	1.30662	8.00	2.83	19.2
72.258	1.30646	4.00	2.00	19.2
72.268	1.30631	9.00	3.00	19.2
72.278	1.30615	2.00	1.41	19.2
72.289	1.30599	2.00	1.41	19.2
72.299	1.30583	3.00	1.73	19.2
72.309	1.30567	5.00	2.24	19.2
72.319	1.30552	5.00	2.24	19.2
72.329	1.30536	7.00	2.65	19.2
72.339	1.30520	6.00	2.45	19.2
72.349	1.30505	8.00	2.83	19.2
72.359	1.30489	5.00	2.24	19.2
72.369	1.30473	7.00	2.65	19.2
72.379	1.30457	4.00	2.00	19.2
72.390	1.30442	4.00	2.00	19.2
72.400	1.30426	2.00	1.41	19.2
72.410	1.30410	6.00	2.45	19.2
72.420	1.30395	4.00	2.00	19.2
72.430	1.30379	1.00	1.00	19.2
72.440	1.30363	6.00	2.45	19.2
72.450	1.30347	4.00	2.00	19.2
72.460	1.30332	12.0	3.46	19.2
72.470	1.30316	9.00	3.00	19.2
72.480	1.30300	7.00	2.65	19.2
72.491	1.30285	6.00	2.45	19.2
72.501	1.30269	8.00	2.83	19.2
72.511	1.30253	4.00	2.00	19.2
72.521	1.30238	9.00	3.00	19.2
72.531	1.30222	8.00	2.83	19.2
72.541	1.30207	5.00	2.24	19.2
72.551	1.30191	4.00	2.00	19.2
72.561	1.30175	3.00	1.73	19.2
72.571	1.30160	4.00	2.00	19.2
72.581	1.30144	6.00	2.45	19.2
72.592	1.30128	3.00	1.73	19.2
72.602	1.30113	6.00	2.45	19.2
72.612	1.30097	3.00	1.73	19.2
72.622	1.30082	9.00	3.00	19.2
72.632	1.30066	5.00	2.24	19.2
72.642	1.30050	6.00	2.45	19.2
72.652	1.30035	3.00	1.73	19.2

72.662	1.30019	5.00	2.24	19.2
72.672	1.30004	4.00	2.00	19.2
72.682	1.29988	5.00	2.24	19.2
72.693	1.29972	2.00	1.41	19.2
72.703	1.29957	4.00	2.00	19.2
72.713	1.29941	2.00	1.41	19.2
72.723	1.29926	6.00	2.45	19.2
72.733	1.29910	5.00	2.24	19.2
72.743	1.29895	3.00	1.73	19.2
72.753	1.29879	2.00	1.41	19.2
72.763	1.29864	7.00	2.65	19.2
72.773	1.29848	5.00	2.24	19.2
72.783	1.29833	7.00	2.65	19.2
72.794	1.29817	10.0	3.16	19.2
72.804	1.29802	3.00	1.73	19.2
72.814	1.29786	10.0	3.16	19.2
72.824	1.29771	5.00	2.24	19.2
72.834	1.29755	3.00	1.73	19.2
72.844	1.29740	9.00	3.00	19.2
72.854	1.29724	5.00	2.24	19.2
72.864	1.29709	5.00	2.24	19.2
72.874	1.29693	7.00	2.65	19.2
72.884	1.29678	4.00	2.00	19.2
72.895	1.29662	4.00	2.00	19.2
72.905	1.29647	7.00	2.65	19.2
72.915	1.29631	6.00	2.45	19.2
72.925	1.29616	7.00	2.65	19.2
72.935	1.29600	3.00	1.73	19.2
72.945	1.29585	4.00	2.00	19.2
72.955	1.29569	4.00	2.00	19.2
72.965	1.29554	2.00	1.41	19.2
72.975	1.29538	2.00	1.41	19.2
72.985	1.29523	4.00	2.00	19.2
72.996	1.29508	3.00	1.73	19.2
73.006	1.29492	4.00	2.00	19.2
73.016	1.29477	5.00	2.24	19.2
73.026	1.29461	3.00	1.73	19.2
73.036	1.29446	5.00	2.24	19.2
73.046	1.29431	7.00	2.65	19.2
73.056	1.29415	3.00	1.73	19.2
73.066	1.29400	6.00	2.45	19.2
73.076	1.29384	2.00	1.41	19.2
73.086	1.29369	7.00	2.65	19.2
73.097	1.29354	2.00	1.41	19.2
73.107	1.29338	3.00	1.73	19.2
73.117	1.29323	4.00	2.00	19.2
73.127	1.29307	3.00	1.73	19.2
73.137	1.29292	3.00	1.73	19.2
73.147	1.29277	3.00	1.73	19.2
73.157	1.29261	4.00	2.00	19.2

73.167	1.29246	5.00	2.24	19.2
73.177	1.29231	8.00	2.83	19.2
73.187	1.29215	4.00	2.00	19.2
73.198	1.29200	3.00	1.73	19.2
73.208	1.29185	3.00	1.73	19.2
73.218	1.29169	5.00	2.24	19.2
73.228	1.29154	3.00	1.73	19.2
73.238	1.29139	2.00	1.41	19.2
73.248	1.29123	7.00	2.65	19.2
73.258	1.29108	2.00	1.41	19.2
73.268	1.29093	3.00	1.73	19.2
73.278	1.29077	3.00	1.73	19.2
73.288	1.29062	6.00	2.45	19.2
73.299	1.29047	2.00	1.41	19.2
73.309	1.29032	3.00	1.73	19.2
73.319	1.29016	3.00	1.73	19.2
73.329	1.29001	4.00	2.00	19.2
73.339	1.28986	6.00	2.45	19.2
73.349	1.28971	4.00	2.00	19.2
73.359	1.28955	4.00	2.00	19.2
73.369	1.28940	4.00	2.00	19.2
73.379	1.28925	2.00	1.41	19.2
73.389	1.28910	3.00	1.73	19.2
73.399	1.28894	4.00	2.00	19.2
73.410	1.28879	4.00	2.00	19.2
73.420	1.28864	4.00	2.00	19.2
73.430	1.28849	4.00	2.00	19.2
73.440	1.28833	9.00	3.00	19.2
73.450	1.28818	3.00	1.73	19.2
73.460	1.28803	7.00	2.65	19.2
73.470	1.28788	4.00	2.00	19.2
73.480	1.28772	1.00	1.00	19.2
73.490	1.28757	3.00	1.73	19.2
73.500	1.28742	5.00	2.24	19.2
73.511	1.28727	4.00	2.00	19.2
73.521	1.28712	5.00	2.24	19.2
73.531	1.28697	7.00	2.65	19.2
73.541	1.28681	4.00	2.00	19.2
73.551	1.28666	2.00	1.41	19.2
73.561	1.28651	1.00	1.00	19.2
73.571	1.28636	2.00	1.41	19.2
73.581	1.28621	4.00	2.00	19.2
73.591	1.28606	5.00	2.24	19.2
73.601	1.28590	3.00	1.73	19.2
73.612	1.28575	7.00	2.65	19.2
73.622	1.28560	6.00	2.45	19.2
73.632	1.28545	5.00	2.24	19.2
73.642	1.28530	2.00	1.41	19.2
73.652	1.28515	6.00	2.45	19.2
73.662	1.28500	3.00	1.73	19.2

73.672	1.28484	5.00	2.24	19.2
73.682	1.28469	6.00	2.45	19.2
73.692	1.28454	3.00	1.73	19.2
73.702	1.28439	5.00	2.24	19.2
73.713	1.28424	4.00	2.00	19.2
73.723	1.28409	0	0	19.2
73.733	1.28394	5.00	2.24	19.2
73.743	1.28379	3.00	1.73	19.2
73.753	1.28364	6.00	2.45	19.2
73.763	1.28349	6.00	2.45	19.2
73.773	1.28334	5.00	2.24	19.2
73.783	1.28318	3.00	1.73	19.2
73.793	1.28303	3.00	1.73	19.2
73.803	1.28288	5.00	2.24	19.2
73.814	1.28273	4.00	2.00	19.2
73.824	1.28258	6.00	2.45	19.2
73.834	1.28243	4.00	2.00	19.2
73.844	1.28228	8.00	2.83	19.2
73.854	1.28213	2.00	1.41	19.2
73.864	1.28198	4.00	2.00	19.2
73.874	1.28183	4.00	2.00	19.2
73.884	1.28168	1.00	1.00	19.2
73.894	1.28153	4.00	2.00	19.2
73.904	1.28138	2.00	1.41	19.2
73.915	1.28123	2.00	1.41	19.2
73.925	1.28108	5.00	2.24	19.2
73.935	1.28093	5.00	2.24	19.2
73.945	1.28078	3.00	1.73	19.2
73.955	1.28063	3.00	1.73	19.2
73.965	1.28048	4.00	2.00	19.2
73.975	1.28033	3.00	1.73	19.2
73.985	1.28018	6.00	2.45	19.2
73.995	1.28003	4.00	2.00	19.2
74.005	1.27988	3.00	1.73	19.2
74.016	1.27973	7.00	2.65	19.2
74.026	1.27958	3.00	1.73	19.2
74.036	1.27943	2.00	1.41	19.2
74.046	1.27928	3.00	1.73	19.2
74.056	1.27913	5.00	2.24	19.2
74.066	1.27898	3.00	1.73	19.2
74.076	1.27883	5.00	2.24	19.2
74.086	1.27868	7.00	2.65	19.2
74.096	1.27854	7.00	2.65	19.2
74.106	1.27839	1.00	1.00	19.2
74.117	1.27824	3.00	1.73	19.2
74.127	1.27809	6.00	2.45	19.2
74.137	1.27794	5.00	2.24	19.2
74.147	1.27779	1.00	1.00	19.2
74.157	1.27764	3.00	1.73	19.2
74.167	1.27749	6.00	2.45	19.2

74.177	1.27734	3.00	1.73	19.2
74.187	1.27719	7.00	2.65	19.2
74.197	1.27704	3.00	1.73	19.2
74.207	1.27690	5.00	2.24	19.2
74.218	1.27675	3.00	1.73	19.2
74.228	1.27660	6.00	2.45	19.2
74.238	1.27645	4.00	2.00	19.2
74.248	1.27630	2.00	1.41	19.2
74.258	1.27615	2.00	1.41	19.2
74.268	1.27600	4.00	2.00	19.2
74.278	1.27586	7.00	2.65	19.2
74.288	1.27571	3.00	1.73	19.2
74.298	1.27556	3.00	1.73	19.2
74.308	1.27541	1.00	1.00	19.2
74.319	1.27526	6.00	2.45	19.2
74.329	1.27511	2.00	1.41	19.2
74.339	1.27497	3.00	1.73	19.2
74.349	1.27482	2.00	1.41	19.2
74.359	1.27467	2.00	1.41	19.2
74.369	1.27452	7.00	2.65	19.2
74.379	1.27437	3.00	1.73	19.2
74.389	1.27423	3.00	1.73	19.2
74.399	1.27408	3.00	1.73	19.2
74.409	1.27393	4.00	2.00	19.2
74.420	1.27378	1.00	1.00	19.2
74.430	1.27363	4.00	2.00	19.2
74.440	1.27349	2.00	1.41	19.2
74.450	1.27334	2.00	1.41	19.2
74.460	1.27319	3.00	1.73	19.2
74.470	1.27304	2.00	1.41	19.2
74.480	1.27290	2.00	1.41	19.2
74.490	1.27275	4.00	2.00	19.2
74.500	1.27260	1.00	1.00	19.2
74.510	1.27245	2.00	1.41	19.2
74.521	1.27231	2.00	1.41	19.2
74.531	1.27216	3.00	1.73	19.2
74.541	1.27201	4.00	2.00	19.2
74.551	1.27186	6.00	2.45	19.2
74.561	1.27172	1.00	1.00	19.2
74.571	1.27157	7.00	2.65	19.2
74.581	1.27142	3.00	1.73	19.2
74.591	1.27127	4.00	2.00	19.2
74.601	1.27113	3.00	1.73	19.2
74.611	1.27098	1.00	1.00	19.2
74.622	1.27083	2.00	1.41	19.2
74.632	1.27069	3.00	1.73	19.2
74.642	1.27054	5.00	2.24	19.2
74.652	1.27039	2.00	1.41	19.2
74.662	1.27025	2.00	1.41	19.2
74.672	1.27010	5.00	2.24	19.2

74.682	1.26995	1.00	1.00	19.2
74.692	1.26981	1.00	1.00	19.2
74.702	1.26966	6.00	2.45	19.2
74.712	1.26951	2.00	1.41	19.2
74.722	1.26937	2.00	1.41	19.2
74.733	1.26922	2.00	1.41	19.2
74.743	1.26907	3.00	1.73	19.2
74.753	1.26893	2.00	1.41	19.2
74.763	1.26878	4.00	2.00	19.2
74.773	1.26863	5.00	2.24	19.2
74.783	1.26849	4.00	2.00	19.2
74.793	1.26834	3.00	1.73	19.2
74.803	1.26820	3.00	1.73	19.2
74.813	1.26805	1.00	1.00	19.2
74.823	1.26790	3.00	1.73	19.2
74.834	1.26776	4.00	2.00	19.2
74.844	1.26761	0	0	19.2
74.854	1.26746	2.00	1.41	19.2
74.864	1.26732	4.00	2.00	19.2
74.874	1.26717	4.00	2.00	19.2
74.884	1.26703	5.00	2.24	19.2
74.894	1.26688	1.00	1.00	19.2
74.904	1.26674	4.00	2.00	19.2
74.914	1.26659	4.00	2.00	19.2
74.924	1.26644	5.00	2.24	19.2
74.935	1.26630	4.00	2.00	19.2
74.945	1.26615	1.00	1.00	19.2
74.955	1.26601	2.00	1.41	19.2
74.965	1.26586	0	0	19.2
74.975	1.26572	5.00	2.24	19.2
74.985	1.26557	2.00	1.41	19.2
74.995	1.26543	1.00	1.00	19.2
75.005	1.26528	1.00	1.00	19.2
75.015	1.26513	4.00	2.00	19.2
75.025	1.26499	7.00	2.65	19.2
75.036	1.26484	3.00	1.73	19.2
75.046	1.26470	3.00	1.73	19.2
75.056	1.26455	3.00	1.73	19.2
75.066	1.26441	5.00	2.24	19.2
75.076	1.26426	5.00	2.24	19.2
75.086	1.26412	4.00	2.00	19.2
75.096	1.26397	2.00	1.41	19.2
75.106	1.26383	1.00	1.00	19.2
75.116	1.26368	4.00	2.00	19.2
75.126	1.26354	6.00	2.45	19.2
75.137	1.26339	7.00	2.65	19.2
75.147	1.26325	5.00	2.24	19.2
75.157	1.26311	1.00	1.00	19.2
75.167	1.26296	2.00	1.41	19.2
75.177	1.26282	3.00	1.73	19.2

75.187	1.26267	5.00	2.24	19.2
75.197	1.26253	5.00	2.24	19.2
75.207	1.26238	1.00	1.00	19.2
75.217	1.26224	2.00	1.41	19.2
75.227	1.26209	1.00	1.00	19.2
75.238	1.26195	2.00	1.41	19.2
75.248	1.26181	4.00	2.00	19.2
75.258	1.26166	1.00	1.00	19.2
75.268	1.26152	3.00	1.73	19.2
75.278	1.26137	3.00	1.73	19.2
75.288	1.26123	2.00	1.41	19.2
75.298	1.26108	1.00	1.00	19.2
75.308	1.26094	3.00	1.73	19.2
75.318	1.26080	3.00	1.73	19.2
75.328	1.26065	2.00	1.41	19.2
75.339	1.26051	4.00	2.00	19.2
75.349	1.26036	3.00	1.73	19.2
75.359	1.26022	3.00	1.73	19.2
75.369	1.26008	5.00	2.24	19.2
75.379	1.25993	2.00	1.41	19.2
75.389	1.25979	1.00	1.00	19.2
75.399	1.25965	5.00	2.24	19.2
75.409	1.25950	1.00	1.00	19.2
75.419	1.25936	2.00	1.41	19.2
75.429	1.25921	2.00	1.41	19.2
75.440	1.25907	1.00	1.00	19.2
75.450	1.25893	1.00	1.00	19.2
75.460	1.25878	4.00	2.00	19.2
75.470	1.25864	2.00	1.41	19.2
75.480	1.25850	2.00	1.41	19.2
75.490	1.25835	1.00	1.00	19.2
75.500	1.25821	4.00	2.00	19.2
75.510	1.25807	2.00	1.41	19.2
75.520	1.25793	2.00	1.41	19.2
75.530	1.25778	1.00	1.00	19.2
75.541	1.25764	4.00	2.00	19.2
75.551	1.25750	1.00	1.00	19.2
75.561	1.25735	3.00	1.73	19.2
75.571	1.25721	1.00	1.00	19.2
75.581	1.25707	2.00	1.41	19.2
75.591	1.25692	2.00	1.41	19.2
75.601	1.25678	2.00	1.41	19.2
75.611	1.25664	2.00	1.41	19.2
75.621	1.25650	3.00	1.73	19.2
75.631	1.25635	0	0	19.2
75.642	1.25621	2.00	1.41	19.2
75.652	1.25607	0	0	19.2
75.662	1.25593	2.00	1.41	19.2
75.672	1.25578	2.00	1.41	19.2
75.682	1.25564	3.00	1.73	19.2

75.692	1.25550	3.00	1.73	19.2
75.702	1.25536	2.00	1.41	19.2
75.712	1.25521	3.00	1.73	19.2
75.722	1.25507	4.00	2.00	19.2
75.732	1.25493	5.00	2.24	19.2
75.743	1.25479	2.00	1.41	19.2
75.753	1.25464	1.00	1.00	19.2
75.763	1.25450	3.00	1.73	19.2
75.773	1.25436	0	0	19.2
75.783	1.25422	3.00	1.73	19.2
75.793	1.25408	2.00	1.41	19.2
75.803	1.25393	2.00	1.41	19.2
75.813	1.25379	3.00	1.73	19.2
75.823	1.25365	0	0	19.2
75.833	1.25351	0	0	19.2
75.844	1.25337	3.00	1.73	19.2
75.854	1.25322	1.00	1.00	19.2
75.864	1.25308	3.00	1.73	19.2
75.874	1.25294	1.00	1.00	19.2
75.884	1.25280	6.00	2.45	19.2
75.894	1.25266	0	0	19.2
75.904	1.25252	5.00	2.24	19.2
75.914	1.25238	1.00	1.00	19.2
75.924	1.25223	4.00	2.00	19.2
75.934	1.25209	3.00	1.73	19.2
75.945	1.25195	0	0	19.2
75.955	1.25181	1.00	1.00	19.2
75.965	1.25167	0	0	19.2
75.975	1.25153	3.00	1.73	19.2
75.985	1.25139	0	0	19.2
75.995	1.25124	3.00	1.73	19.2
76.005	1.25110	1.00	1.00	19.2
76.015	1.25096	4.00	2.00	19.2
76.025	1.25082	2.00	1.41	19.2
76.035	1.25068	2.00	1.41	19.2
76.045	1.25054	0	0	19.2
76.056	1.25040	4.00	2.00	19.2
76.066	1.25026	6.00	2.45	19.2
76.076	1.25012	3.00	1.73	19.2
76.086	1.24998	3.00	1.73	19.2
76.096	1.24983	3.00	1.73	19.2
76.106	1.24969	1.00	1.00	19.2
76.116	1.24955	1.00	1.00	19.2
76.126	1.24941	3.00	1.73	19.2
76.136	1.24927	3.00	1.73	19.2
76.146	1.24913	2.00	1.41	19.2
76.157	1.24899	1.00	1.00	19.2
76.167	1.24885	1.00	1.00	19.2
76.177	1.24871	0	0	19.2
76.187	1.24857	1.00	1.00	19.2

76.197	1.24843	2.00	1.41	19.2
76.207	1.24829	1.00	1.00	19.2
76.217	1.24815	1.00	1.00	19.2
76.227	1.24801	4.00	2.00	19.2
76.237	1.24787	3.00	1.73	19.2
76.247	1.24773	0	0	19.2
76.258	1.24759	3.00	1.73	19.2
76.268	1.24745	2.00	1.41	19.2
76.278	1.24731	1.00	1.00	19.2
76.288	1.24717	0	0	19.2
76.298	1.24703	3.00	1.73	19.2
76.308	1.24689	1.00	1.00	19.2
76.318	1.24675	3.00	1.73	19.2
76.328	1.24661	3.00	1.73	19.2
76.338	1.24647	2.00	1.41	19.2
76.348	1.24633	1.00	1.00	19.2
76.359	1.24619	0	0	19.2
76.369	1.24605	0	0	19.2
76.379	1.24591	0	0	19.2
76.389	1.24577	3.00	1.73	19.2
76.399	1.24563	2.00	1.41	19.2
76.409	1.24549	2.00	1.41	19.2
76.419	1.24535	4.00	2.00	19.2
76.429	1.24521	1.00	1.00	19.2
76.439	1.24507	4.00	2.00	19.2
76.449	1.24493	1.00	1.00	19.2
76.460	1.24479	3.00	1.73	19.2
76.470	1.24466	1.00	1.00	19.2
76.480	1.24452	1.00	1.00	19.2
76.490	1.24438	6.00	2.45	19.2
76.500	1.24424	2.00	1.41	19.2
76.510	1.24410	4.00	2.00	19.2
76.520	1.24396	3.00	1.73	19.2
76.530	1.24382	2.00	1.41	19.2
76.540	1.24368	5.00	2.24	19.2
76.550	1.24354	1.00	1.00	19.2
76.561	1.24340	4.00	2.00	19.2
76.571	1.24327	1.00	1.00	19.2
76.581	1.24313	2.00	1.41	19.2
76.591	1.24299	2.00	1.41	19.2
76.601	1.24285	2.00	1.41	19.2
76.611	1.24271	1.00	1.00	19.2
76.621	1.24257	3.00	1.73	19.2
76.631	1.24243	2.00	1.41	19.2
76.641	1.24229	3.00	1.73	19.2
76.651	1.24216	2.00	1.41	19.2
76.662	1.24202	5.00	2.24	19.2
76.672	1.24188	0	0	19.2
76.682	1.24174	3.00	1.73	19.2
76.692	1.24160	1.00	1.00	19.2

76.702	1.24146	1.00	1.00	19.2
76.712	1.24133	0	0	19.2
76.722	1.24119	2.00	1.41	19.2
76.732	1.24105	2.00	1.41	19.2
76.742	1.24091	0	0	19.2
76.752	1.24077	1.00	1.00	19.2
76.763	1.24064	1.00	1.00	19.2
76.773	1.24050	1.00	1.00	19.2
76.783	1.24036	2.00	1.41	19.2
76.793	1.24022	2.00	1.41	19.2
76.803	1.24008	0	0	19.2
76.813	1.23995	3.00	1.73	19.2
76.823	1.23981	2.00	1.41	19.2
76.833	1.23967	2.00	1.41	19.2
76.843	1.23953	2.00	1.41	19.2
76.853	1.23939	4.00	2.00	19.2
76.864	1.23926	0	0	19.2
76.874	1.23912	0	0	19.2
76.884	1.23898	4.00	2.00	19.2
76.894	1.23884	2.00	1.41	19.2
76.904	1.23871	0	0	19.2
76.914	1.23857	1.00	1.00	19.2
76.924	1.23843	1.00	1.00	19.2
76.934	1.23829	1.00	1.00	19.2
76.944	1.23816	2.00	1.41	19.2
76.954	1.23802	1.00	1.00	19.2
76.965	1.23788	1.00	1.00	19.2
76.975	1.23775	1.00	1.00	19.2
76.985	1.23761	2.00	1.41	19.2
76.995	1.23747	5.00	2.24	19.2
77.005	1.23733	0	0	19.2
77.015	1.23720	4.00	2.00	19.2
77.025	1.23706	4.00	2.00	19.2
77.035	1.23692	2.00	1.41	19.2
77.045	1.23679	2.00	1.41	19.2
77.055	1.23665	3.00	1.73	19.2
77.066	1.23651	1.00	1.00	19.2
77.076	1.23638	0	0	19.2
77.086	1.23624	3.00	1.73	19.2
77.096	1.23610	2.00	1.41	19.2
77.106	1.23596	1.00	1.00	19.2
77.116	1.23583	0	0	19.2
77.126	1.23569	3.00	1.73	19.2
77.136	1.23556	0	0	19.2
77.146	1.23542	2.00	1.41	19.2
77.156	1.23528	3.00	1.73	19.2
77.167	1.23515	1.00	1.00	19.2
77.177	1.23501	1.00	1.00	19.2
77.187	1.23487	1.00	1.00	19.2
77.197	1.23474	0	0	19.2

77.207	1.23460	1.00	1.00	19.2
77.217	1.23446	0	0	19.2
77.227	1.23433	2.00	1.41	19.2
77.237	1.23419	1.00	1.00	19.2
77.247	1.23406	0	0	19.2
77.257	1.23392	0	0	19.2
77.268	1.23378	1.00	1.00	19.2
77.278	1.23365	0	0	19.2
77.288	1.23351	2.00	1.41	19.2
77.298	1.23338	2.00	1.41	19.2
77.308	1.23324	0	0	19.2
77.318	1.23310	2.00	1.41	19.2
77.328	1.23297	1.00	1.00	19.2
77.338	1.23283	3.00	1.73	19.2
77.348	1.23270	0	0	19.2
77.358	1.23256	0	0	19.2
77.368	1.23242	1.00	1.00	19.2
77.379	1.23229	3.00	1.73	19.2
77.389	1.23215	3.00	1.73	19.2
77.399	1.23202	2.00	1.41	19.2
77.409	1.23188	5.00	2.24	19.2
77.419	1.23175	0	0	19.2
77.429	1.23161	2.00	1.41	19.2
77.439	1.23148	1.00	1.00	19.2
77.449	1.23134	1.00	1.00	19.2
77.459	1.23121	1.00	1.00	19.2
77.469	1.23107	3.00	1.73	19.2
77.480	1.23093	1.00	1.00	19.2
77.490	1.23080	2.00	1.41	19.2
77.500	1.23066	1.00	1.00	19.2
77.510	1.23053	1.00	1.00	19.2
77.520	1.23039	0	0	19.2
77.530	1.23026	2.00	1.41	19.2
77.540	1.23012	1.00	1.00	19.2
77.550	1.22999	1.00	1.00	19.2
77.560	1.22985	2.00	1.41	19.2
77.570	1.22972	2.00	1.41	19.2
77.581	1.22958	3.00	1.73	19.2
77.591	1.22945	1.00	1.00	19.2
77.601	1.22932	4.00	2.00	19.2
77.611	1.22918	1.00	1.00	19.2
77.621	1.22905	0	0	19.2
77.631	1.22891	0	0	19.2
77.641	1.22878	1.00	1.00	19.2
77.651	1.22864	1.00	1.00	19.2
77.661	1.22851	1.00	1.00	19.2
77.671	1.22837	2.00	1.41	19.2
77.682	1.22824	1.00	1.00	19.2
77.692	1.22810	1.00	1.00	19.2
77.702	1.22797	0	0	19.2

77.712	1.22784	2.00	1.41	19.2
77.722	1.22770	1.00	1.00	19.2
77.732	1.22757	1.00	1.00	19.2
77.742	1.22743	1.00	1.00	19.2
77.752	1.22730	2.00	1.41	19.2
77.762	1.22716	1.00	1.00	19.2
77.772	1.22703	1.00	1.00	19.2
77.783	1.22690	2.00	1.41	19.2
77.793	1.22676	0	0	19.2
77.803	1.22663	0	0	19.2
77.813	1.22649	3.00	1.73	19.2
77.823	1.22636	1.00	1.00	19.2
77.833	1.22623	1.00	1.00	19.2
77.843	1.22609	2.00	1.41	19.2
77.853	1.22596	3.00	1.73	19.2
77.863	1.22582	1.00	1.00	19.2
77.873	1.22569	0	0	19.2
77.884	1.22556	2.00	1.41	19.2
77.894	1.22542	0	0	19.2
77.904	1.22529	0	0	19.2
77.914	1.22516	2.00	1.41	19.2
77.924	1.22502	1.00	1.00	19.2
77.934	1.22489	1.00	1.00	19.2
77.944	1.22476	1.00	1.00	19.2
77.954	1.22462	0	0	19.2
77.964	1.22449	2.00	1.41	19.2
77.974	1.22436	1.00	1.00	19.2
77.985	1.22422	1.00	1.00	19.2
77.995	1.22409	2.00	1.41	19.2
78.005	1.22396	2.00	1.41	19.2
78.015	1.22382	1.00	1.00	19.2
78.025	1.22369	0	0	19.2
78.035	1.22356	2.00	1.41	19.2
78.045	1.22342	2.00	1.41	19.2
78.055	1.22329	3.00	1.73	19.2
78.065	1.22316	1.00	1.00	19.2
78.075	1.22302	1.00	1.00	19.2
78.086	1.22289	1.00	1.00	19.2
78.096	1.22276	1.00	1.00	19.2
78.106	1.22263	3.00	1.73	19.2
78.116	1.22249	0	0	19.2
78.126	1.22236	2.00	1.41	19.2
78.136	1.22223	2.00	1.41	19.2
78.146	1.22210	3.00	1.73	19.2
78.156	1.22196	0	0	19.2
78.166	1.22183	2.00	1.41	19.2
78.176	1.22170	3.00	1.73	19.2
78.187	1.22156	0	0	19.2
78.197	1.22143	1.00	1.00	19.2
78.207	1.22130	6.00	2.45	19.2

78.217	1.22117	1.00	1.00	19.2
78.227	1.22104	2.00	1.41	19.2
78.237	1.22090	1.00	1.00	19.2
78.247	1.22077	0	0	19.2
78.257	1.22064	2.00	1.41	19.2
78.267	1.22051	0	0	19.2
78.277	1.22037	2.00	1.41	19.2
78.288	1.22024	1.00	1.00	19.2
78.298	1.22011	1.00	1.00	19.2
78.308	1.21998	2.00	1.41	19.2
78.318	1.21985	2.00	1.41	19.2
78.328	1.21971	1.00	1.00	19.2
78.338	1.21958	1.00	1.00	19.2
78.348	1.21945	2.00	1.41	19.2
78.358	1.21932	1.00	1.00	19.2
78.368	1.21919	1.00	1.00	19.2
78.378	1.21905	0	0	19.2
78.389	1.21892	2.00	1.41	19.2
78.399	1.21879	0	0	19.2
78.409	1.21866	1.00	1.00	19.2
78.419	1.21853	2.00	1.41	19.2
78.429	1.21840	3.00	1.73	19.2
78.439	1.21826	0	0	19.2
78.449	1.21813	2.00	1.41	19.2
78.459	1.21800	2.00	1.41	19.2
78.469	1.21787	3.00	1.73	19.2
78.479	1.21774	0	0	19.2
78.490	1.21761	2.00	1.41	19.2
78.500	1.21748	2.00	1.41	19.2
78.510	1.21734	3.00	1.73	19.2
78.520	1.21721	1.00	1.00	19.2
78.530	1.21708	1.00	1.00	19.2
78.540	1.21695	1.00	1.00	19.2
78.550	1.21682	1.00	1.00	19.2
78.560	1.21669	1.00	1.00	19.2
78.570	1.21656	2.00	1.41	19.2
78.580	1.21643	1.00	1.00	19.2
78.591	1.21630	0	0	19.2
78.601	1.21616	1.00	1.00	19.2
78.611	1.21603	2.00	1.41	19.2
78.621	1.21590	0	0	19.2
78.631	1.21577	0	0	19.2
78.641	1.21564	1.00	1.00	19.2
78.651	1.21551	0	0	19.2
78.661	1.21538	3.00	1.73	19.2
78.671	1.21525	1.00	1.00	19.2
78.681	1.21512	0	0	19.2
78.691	1.21499	0	0	19.2
78.702	1.21486	1.00	1.00	19.2
78.712	1.21473	0	0	19.2

78.722	1.21460	0	0	19.2
78.732	1.21446	2.00	1.41	19.2
78.742	1.21433	0	0	19.2
78.752	1.21420	0	0	19.2
78.762	1.21407	2.00	1.41	19.2
78.772	1.21394	0	0	19.2
78.782	1.21381	1.00	1.00	19.2
78.792	1.21368	2.00	1.41	19.2
78.803	1.21355	2.00	1.41	19.2
78.813	1.21342	3.00	1.73	19.2
78.823	1.21329	0	0	19.2
78.833	1.21316	0	0	19.2
78.843	1.21303	2.00	1.41	19.2
78.853	1.21290	0	0	19.2
78.863	1.21277	2.00	1.41	19.2
78.873	1.21264	1.00	1.00	19.2
78.883	1.21251	0	0	19.2
78.893	1.21238	0	0	19.2
78.904	1.21225	2.00	1.41	19.2
78.914	1.21212	2.00	1.41	19.2
78.924	1.21199	0	0	19.2
78.934	1.21186	1.00	1.00	19.2
78.944	1.21173	1.00	1.00	19.2
78.954	1.21160	2.00	1.41	19.2
78.964	1.21147	2.00	1.41	19.2
78.974	1.21134	0	0	19.2
78.984	1.21121	2.00	1.41	19.2
78.994	1.21109	0	0	19.2
79.005	1.21096	0	0	19.2
79.015	1.21083	0	0	19.2
79.025	1.21070	1.00	1.00	19.2
79.035	1.21057	1.00	1.00	19.2
79.045	1.21044	0	0	19.2
79.055	1.21031	1.00	1.00	19.2
79.065	1.21018	0	0	19.2
79.075	1.21005	0	0	19.2
79.085	1.20992	1.00	1.00	19.2
79.095	1.20979	1.00	1.00	19.2
79.106	1.20966	3.00	1.73	19.2
79.116	1.20953	2.00	1.41	19.2
79.126	1.20941	1.00	1.00	19.2
79.136	1.20928	1.00	1.00	19.2
79.146	1.20915	1.00	1.00	19.2
79.156	1.20902	1.00	1.00	19.2
79.166	1.20889	1.00	1.00	19.2
79.176	1.20876	0	0	19.2
79.186	1.20863	0	0	19.2
79.196	1.20850	1.00	1.00	19.2
79.207	1.20837	0	0	19.2
79.217	1.20825	2.00	1.41	19.2

79.227	1.20812	0	0	19.2
79.237	1.20799	2.00	1.41	19.2
79.247	1.20786	0	0	19.2
79.257	1.20773	1.00	1.00	19.2
79.267	1.20760	0	0	19.2
79.277	1.20747	2.00	1.41	19.2
79.287	1.20735	0	0	19.2
79.297	1.20722	0	0	19.2
79.308	1.20709	0	0	19.2
79.318	1.20696	1.00	1.00	19.2
79.328	1.20683	1.00	1.00	19.2
79.338	1.20670	1.00	1.00	19.2
79.348	1.20658	3.00	1.73	19.2
79.358	1.20645	1.00	1.00	19.2
79.368	1.20632	2.00	1.41	19.2
79.378	1.20619	2.00	1.41	19.2
79.388	1.20606	1.00	1.00	19.2
79.398	1.20594	0	0	19.2
79.409	1.20581	1.00	1.00	19.2
79.419	1.20568	1.00	1.00	19.2
79.429	1.20555	2.00	1.41	19.2
79.439	1.20542	0	0	19.2
79.449	1.20530	0	0	19.2
79.459	1.20517	0	0	19.2
79.469	1.20504	0	0	19.2
79.479	1.20491	0	0	19.2
79.489	1.20478	1.00	1.00	19.2
79.499	1.20466	1.00	1.00	19.2
79.510	1.20453	2.00	1.41	19.2
79.520	1.20440	2.00	1.41	19.2
79.530	1.20427	1.00	1.00	19.2
79.540	1.20415	2.00	1.41	19.2
79.550	1.20402	1.00	1.00	19.2
79.560	1.20389	0	0	19.2
79.570	1.20376	0	0	19.2
79.580	1.20364	0	0	19.2
79.590	1.20351	0	0	19.2
79.600	1.20338	1.00	1.00	19.2
79.611	1.20325	1.00	1.00	19.2
79.621	1.20313	0	0	19.2
79.631	1.20300	1.00	1.00	19.2
79.641	1.20287	0	0	19.2
79.651	1.20275	0	0	19.2
79.661	1.20262	2.00	1.41	19.2
79.671	1.20249	0	0	19.2
79.681	1.20236	1.00	1.00	19.2
79.691	1.20224	1.00	1.00	19.2
79.701	1.20211	1.00	1.00	19.2
79.712	1.20198	2.00	1.41	19.2
79.722	1.20186	0	0	19.2

79.732	1.20173	2.00	1.41	19.2
79.742	1.20160	2.00	1.41	19.2
79.752	1.20148	1.00	1.00	19.2
79.762	1.20135	3.00	1.73	19.2
79.772	1.20122	2.00	1.41	19.2
79.782	1.20110	0	0	19.2
79.792	1.20097	0	0	19.2
79.802	1.20084	2.00	1.41	19.2
79.813	1.20072	1.00	1.00	19.2
79.823	1.20059	0	0	19.2
79.833	1.20046	1.00	1.00	19.2
79.843	1.20034	1.00	1.00	19.2
79.853	1.20021	1.00	1.00	19.2
79.863	1.20008	0	0	19.2
79.873	1.19996	2.00	1.41	19.2
79.883	1.19983	0	0	19.2
79.893	1.19971	2.00	1.41	19.2
79.903	1.19958	0	0	19.2
79.913	1.19945	1.00	1.00	19.2
79.924	1.19933	1.00	1.00	19.2
79.934	1.19920	2.00	1.41	19.2
79.944	1.19908	2.00	1.41	19.2
79.954	1.19895	1.00	1.00	19.2
79.964	1.19882	0	0	19.2
79.974	1.19870	2.00	1.41	19.2
79.984	1.19857	3.00	1.73	19.2
79.994	1.19845	1.00	1.00	19.2
80.004	1.19832	2.00	1.41	19.2
80.014	1.19819	0	0	19.2
80.025	1.19807	1.00	1.00	19.2
80.035	1.19794	2.00	1.41	19.2
80.045	1.19782	3.00	1.73	19.2
80.055	1.19769	0	0	19.2
80.065	1.19756	1.00	1.00	19.2
80.075	1.19744	0	0	19.2
80.085	1.19731	0	0	19.2
80.095	1.19719	2.00	1.41	19.2
80.105	1.19706	1.00	1.00	19.2
80.115	1.19694	0	0	19.2
80.126	1.19681	2.00	1.41	19.2
80.136	1.19669	1.00	1.00	19.2
80.146	1.19656	1.00	1.00	19.2
80.156	1.19644	1.00	1.00	19.2
80.166	1.19631	1.00	1.00	19.2
80.176	1.19618	0	0	19.2
80.186	1.19606	0	0	19.2
80.196	1.19593	0	0	19.2
80.206	1.19581	1.00	1.00	19.2
80.216	1.19568	0	0	19.2
80.227	1.19556	1.00	1.00	19.2

80.237	1.19543	0	0	19.2
80.247	1.19531	0	0	19.2
80.257	1.19518	4.00	2.00	19.2
80.267	1.19506	1.00	1.00	19.2
80.277	1.19493	1.00	1.00	19.2
80.287	1.19481	1.00	1.00	19.2
80.297	1.19468	2.00	1.41	19.2
80.307	1.19456	1.00	1.00	19.2
80.317	1.19443	0	0	19.2
80.328	1.19431	1.00	1.00	19.2
80.338	1.19419	3.00	1.73	19.2
80.348	1.19406	1.00	1.00	19.2
80.358	1.19394	0	0	19.2
80.368	1.19381	1.00	1.00	19.2
80.378	1.19369	1.00	1.00	19.2
80.388	1.19356	1.00	1.00	19.2
80.398	1.19344	1.00	1.00	19.2
80.408	1.19331	3.00	1.73	19.2
80.418	1.19319	0	0	19.2
80.429	1.19306	0	0	19.2
80.439	1.19294	1.00	1.00	19.2
80.449	1.19282	1.00	1.00	19.2
80.459	1.19269	0	0	19.2
80.469	1.19257	0	0	19.2
80.479	1.19244	0	0	19.2
80.489	1.19232	1.00	1.00	19.2
80.499	1.19220	0	0	19.2
80.509	1.19207	0	0	19.2
80.519	1.19195	0	0	19.2
80.530	1.19182	1.00	1.00	19.2
80.540	1.19170	5.00	2.24	19.2
80.550	1.19158	0	0	19.2
80.560	1.19145	2.00	1.41	19.2
80.570	1.19133	2.00	1.41	19.2
80.580	1.19120	3.00	1.73	19.2
80.590	1.19108	1.00	1.00	19.2
80.600	1.19096	1.00	1.00	19.2
80.610	1.19083	0	0	19.2
80.620	1.19071	1.00	1.00	19.2
80.631	1.19058	1.00	1.00	19.2
80.641	1.19046	4.00	2.00	19.2
80.651	1.19034	0	0	19.2
80.661	1.19021	1.00	1.00	19.2
80.671	1.19009	2.00	1.41	19.2
80.681	1.18997	1.00	1.00	19.2
80.691	1.18984	1.00	1.00	19.2
80.701	1.18972	0	0	19.2
80.711	1.18960	1.00	1.00	19.2
80.721	1.18947	3.00	1.73	19.2
80.732	1.18935	1.00	1.00	19.2

80.742	1.18923	0	0	19.2
80.752	1.18910	2.00	1.41	19.2
80.762	1.18898	0	0	19.2
80.772	1.18886	0	0	19.2
80.782	1.18873	1.00	1.00	19.2
80.792	1.18861	3.00	1.73	19.2
80.802	1.18849	1.00	1.00	19.2
80.812	1.18836	1.00	1.00	19.2
80.822	1.18824	0	0	19.2
80.833	1.18812	1.00	1.00	19.2
80.843	1.18800	1.00	1.00	19.2
80.853	1.18787	0	0	19.2
80.863	1.18775	0	0	19.2
80.873	1.18763	0	0	19.2
80.883	1.18750	0	0	19.2
80.893	1.18738	2.00	1.41	19.2
80.903	1.18726	1.00	1.00	19.2
80.913	1.18714	0	0	19.2
80.923	1.18701	3.00	1.73	19.2
80.934	1.18689	0	0	19.2
80.944	1.18677	1.00	1.00	19.2
80.954	1.18665	1.00	1.00	19.2
80.964	1.18652	0	0	19.2
80.974	1.18640	1.00	1.00	19.2
80.984	1.18628	1.00	1.00	19.2
80.994	1.18616	1.00	1.00	19.2
81.004	1.18603	1.00	1.00	19.2
81.014	1.18591	0	0	19.2
81.024	1.18579	0	0	19.2
81.035	1.18567	0	0	19.2
81.045	1.18554	0	0	19.2
81.055	1.18542	0	0	19.2
81.065	1.18530	2.00	1.41	19.2
81.075	1.18518	0	0	19.2
81.085	1.18506	0	0	19.2
81.095	1.18493	2.00	1.41	19.2
81.105	1.18481	0	0	19.2
81.115	1.18469	3.00	1.73	19.2
81.125	1.18457	0	0	19.2
81.136	1.18444	1.00	1.00	19.2
81.146	1.18432	0	0	19.2
81.156	1.18420	1.00	1.00	19.2
81.166	1.18408	0	0	19.2
81.176	1.18396	1.00	1.00	19.2
81.186	1.18384	0	0	19.2
81.196	1.18371	1.00	1.00	19.2
81.206	1.18359	0	0	19.2
81.216	1.18347	3.00	1.73	19.2
81.226	1.18335	1.00	1.00	19.2
81.236	1.18323	0	0	19.2

81.247	1.18311	0	0	19.2
81.257	1.18298	2.00	1.41	19.2
81.267	1.18286	2.00	1.41	19.2
81.277	1.18274	1.00	1.00	19.2
81.287	1.18262	0	0	19.2
81.297	1.18250	0	0	19.2
81.307	1.18238	0	0	19.2
81.317	1.18226	0	0	19.2
81.327	1.18213	1.00	1.00	19.2
81.337	1.18201	1.00	1.00	19.2
81.348	1.18189	1.00	1.00	19.2
81.358	1.18177	0	0	19.2
81.368	1.18165	0	0	19.2
81.378	1.18153	0	0	19.2
81.388	1.18141	1.00	1.00	19.2
81.398	1.18129	0	0	19.2
81.408	1.18117	1.00	1.00	19.2
81.418	1.18104	1.00	1.00	19.2
81.428	1.18092	0	0	19.2
81.438	1.18080	1.00	1.00	19.2
81.449	1.18068	1.00	1.00	19.2
81.459	1.18056	3.00	1.73	19.2
81.469	1.18044	0	0	19.2
81.479	1.18032	0	0	19.2
81.489	1.18020	0	0	19.2
81.499	1.18008	1.00	1.00	19.2
81.509	1.17996	2.00	1.41	19.2
81.519	1.17984	1.00	1.00	19.2
81.529	1.17972	2.00	1.41	19.2
81.539	1.17960	1.00	1.00	19.2
81.550	1.17947	1.00	1.00	19.2
81.560	1.17935	2.00	1.41	19.2
81.570	1.17923	2.00	1.41	19.2
81.580	1.17911	0	0	19.2
81.590	1.17899	0	0	19.2
81.600	1.17887	0	0	19.2
81.610	1.17875	2.00	1.41	19.2
81.620	1.17863	0	0	19.2
81.630	1.17851	1.00	1.00	19.2
81.640	1.17839	1.00	1.00	19.2
81.651	1.17827	1.00	1.00	19.2
81.661	1.17815	2.00	1.41	19.2
81.671	1.17803	0	0	19.2
81.681	1.17791	3.00	1.73	19.2
81.691	1.17779	1.00	1.00	19.2
81.701	1.17767	1.00	1.00	19.2
81.711	1.17755	0	0	19.2
81.721	1.17743	1.00	1.00	19.2
81.731	1.17731	1.00	1.00	19.2
81.741	1.17719	2.00	1.41	19.2

81.752	1.17707	1.00	1.00	19.2
81.762	1.17695	1.00	1.00	19.2
81.772	1.17683	0	0	19.2
81.782	1.17671	1.00	1.00	19.2
81.792	1.17659	1.00	1.00	19.2
81.802	1.17647	1.00	1.00	19.2
81.812	1.17635	1.00	1.00	19.2
81.822	1.17623	0	0	19.2
81.832	1.17611	1.00	1.00	19.2
81.842	1.17599	0	0	19.2
81.853	1.17587	1.00	1.00	19.2
81.863	1.17575	1.00	1.00	19.2
81.873	1.17563	0	0	19.2
81.883	1.17552	1.00	1.00	19.2
81.893	1.17540	0	0	19.2
81.903	1.17528	0	0	19.2
81.913	1.17516	0	0	19.2
81.923	1.17504	1.00	1.00	19.2
81.933	1.17492	4.00	2.00	19.2
81.943	1.17480	0	0	19.2
81.954	1.17468	1.00	1.00	19.2
81.964	1.17456	2.00	1.41	19.2
81.974	1.17444	1.00	1.00	19.2
81.984	1.17432	0	0	19.2
81.994	1.17420	1.00	1.00	19.2
82.004	1.17408	2.00	1.41	19.2
82.014	1.17397	1.00	1.00	19.2
82.024	1.17385	1.00	1.00	19.2
82.034	1.17373	0	0	19.2
82.044	1.17361	3.00	1.73	19.2
82.055	1.17349	0	0	19.2
82.065	1.17337	0	0	19.2
82.075	1.17325	0	0	19.2
82.085	1.17313	0	0	19.2
82.095	1.17301	1.00	1.00	19.2
82.105	1.17290	1.00	1.00	19.2
82.115	1.17278	3.00	1.73	19.2
82.125	1.17266	1.00	1.00	19.2
82.135	1.17254	2.00	1.41	19.2
82.145	1.17242	2.00	1.41	19.2
82.156	1.17230	2.00	1.41	19.2
82.166	1.17218	2.00	1.41	19.2
82.176	1.17207	2.00	1.41	19.2
82.186	1.17195	0	0	19.2
82.196	1.17183	1.00	1.00	19.2
82.206	1.17171	3.00	1.73	19.2
82.216	1.17159	0	0	19.2
82.226	1.17147	4.00	2.00	19.2
82.236	1.17136	0	0	19.2
82.246	1.17124	0	0	19.2

82.257	1.17112	3.00	1.73	19.2
82.267	1.17100	0	0	19.2
82.277	1.17088	0	0	19.2
82.287	1.17076	2.00	1.41	19.2
82.297	1.17065	2.00	1.41	19.2
82.307	1.17053	0	0	19.2
82.317	1.17041	1.00	1.00	19.2
82.327	1.17029	3.00	1.73	19.2
82.337	1.17017	0	0	19.2
82.347	1.17006	1.00	1.00	19.2
82.358	1.16994	2.00	1.41	19.2
82.368	1.16982	0	0	19.2
82.378	1.16970	0	0	19.2
82.388	1.16959	1.00	1.00	19.2
82.398	1.16947	0	0	19.2
82.408	1.16935	1.00	1.00	19.2
82.418	1.16923	1.00	1.00	19.2
82.428	1.16911	0	0	19.2
82.438	1.16900	1.00	1.00	19.2
82.448	1.16888	2.00	1.41	19.2
82.459	1.16876	1.00	1.00	19.2
82.469	1.16864	0	0	19.2
82.479	1.16853	0	0	19.2
82.489	1.16841	0	0	19.2
82.499	1.16829	1.00	1.00	19.2
82.509	1.16817	1.00	1.00	19.2
82.519	1.16806	1.00	1.00	19.2
82.529	1.16794	0	0	19.2
82.539	1.16782	0	0	19.2
82.549	1.16771	0	0	19.2
82.559	1.16759	0	0	19.2
82.570	1.16747	0	0	19.2
82.580	1.16735	0	0	19.2
82.590	1.16724	0	0	19.2
82.600	1.16712	1.00	1.00	19.2
82.610	1.16700	2.00	1.41	19.2
82.620	1.16689	0	0	19.2
82.630	1.16677	2.00	1.41	19.2
82.640	1.16665	1.00	1.00	19.2
82.650	1.16653	0	0	19.2
82.660	1.16642	1.00	1.00	19.2
82.671	1.16630	2.00	1.41	19.2
82.681	1.16618	0	0	19.2
82.691	1.16607	2.00	1.41	19.2
82.701	1.16595	1.00	1.00	19.2
82.711	1.16583	0	0	19.2
82.721	1.16572	1.00	1.00	19.2
82.731	1.16560	0	0	19.2
82.741	1.16548	3.00	1.73	19.2
82.751	1.16537	1.00	1.00	19.2

82.761	1.16525	3.00	1.73	19.2
82.772	1.16513	0	0	19.2
82.782	1.16502	0	0	19.2
82.792	1.16490	0	0	19.2
82.802	1.16478	1.00	1.00	19.2
82.812	1.16467	0	0	19.2
82.822	1.16455	1.00	1.00	19.2
82.832	1.16444	0	0	19.2
82.842	1.16432	1.00	1.00	19.2
82.852	1.16420	2.00	1.41	19.2
82.862	1.16409	1.00	1.00	19.2
82.873	1.16397	0	0	19.2
82.883	1.16385	0	0	19.2
82.893	1.16374	1.00	1.00	19.2
82.903	1.16362	0	0	19.2
82.913	1.16351	0	0	19.2
82.923	1.16339	2.00	1.41	19.2
82.933	1.16327	0	0	19.2
82.943	1.16316	1.00	1.00	19.2
82.953	1.16304	0	0	19.2
82.963	1.16293	0	0	19.2
82.974	1.16281	3.00	1.73	19.2
82.984	1.16269	1.00	1.00	19.2
82.994	1.16258	0	0	19.2
83.004	1.16246	1.00	1.00	19.2
83.014	1.16235	1.00	1.00	19.2
83.024	1.16223	0	0	19.2
83.034	1.16211	2.00	1.41	19.2
83.044	1.16200	0	0	19.2
83.054	1.16188	1.00	1.00	19.2
83.064	1.16177	0	0	19.2
83.075	1.16165	0	0	19.2
83.085	1.16154	0	0	19.2
83.095	1.16142	0	0	19.2
83.105	1.16131	0	0	19.2
83.115	1.16119	0	0	19.2
83.125	1.16107	0	0	19.2
83.135	1.16096	0	0	19.2
83.145	1.16084	0	0	19.2
83.155	1.16073	0	0	19.2
83.165	1.16061	1.00	1.00	19.2
83.176	1.16050	0	0	19.2
83.186	1.16038	0	0	19.2
83.196	1.16027	0	0	19.2
83.206	1.16015	1.00	1.00	19.2
83.216	1.16004	1.00	1.00	19.2
83.226	1.15992	0	0	19.2
83.236	1.15981	0	0	19.2
83.246	1.15969	1.00	1.00	19.2
83.256	1.15958	0	0	19.2

83.266	1.15946	0	0	19.2
83.277	1.15935	1.00	1.00	19.2
83.287	1.15923	1.00	1.00	19.2
83.297	1.15912	1.00	1.00	19.2
83.307	1.15900	0	0	19.2
83.317	1.15889	0	0	19.2
83.327	1.15877	1.00	1.00	19.2
83.337	1.15866	0	0	19.2
83.347	1.15854	0	0	19.2
83.357	1.15843	0	0	19.2
83.367	1.15831	0	0	19.2
83.378	1.15820	0	0	19.2
83.388	1.15808	2.00	1.41	19.2
83.398	1.15797	0	0	19.2
83.408	1.15786	0	0	19.2
83.418	1.15774	1.00	1.00	19.2
83.428	1.15763	1.00	1.00	19.2
83.438	1.15751	0	0	19.2
83.448	1.15740	0	0	19.2
83.458	1.15728	2.00	1.41	19.2
83.468	1.15717	1.00	1.00	19.2
83.479	1.15706	0	0	19.2
83.489	1.15694	1.00	1.00	19.2
83.499	1.15683	1.00	1.00	19.2
83.509	1.15671	1.00	1.00	19.2
83.519	1.15660	1.00	1.00	19.2
83.529	1.15648	0	0	19.2
83.539	1.15637	0	0	19.2
83.549	1.15626	1.00	1.00	19.2
83.559	1.15614	1.00	1.00	19.2
83.569	1.15603	0	0	19.2
83.580	1.15591	1.00	1.00	19.2
83.590	1.15580	0	0	19.2
83.600	1.15569	0	0	19.2
83.610	1.15557	1.00	1.00	19.2
83.620	1.15546	1.00	1.00	19.2
83.630	1.15534	1.00	1.00	19.2
83.640	1.15523	0	0	19.2
83.650	1.15512	1.00	1.00	19.2
83.660	1.15500	1.00	1.00	19.2
83.670	1.15489	0	0	19.2
83.681	1.15478	0	0	19.2
83.691	1.15466	1.00	1.00	19.2
83.701	1.15455	1.00	1.00	19.2
83.711	1.15443	0	0	19.2
83.721	1.15432	0	0	19.2
83.731	1.15421	0	0	19.2
83.741	1.15409	0	0	19.2
83.751	1.15398	0	0	19.2
83.761	1.15387	2.00	1.41	19.2

83.771	1.15375	0	0	19.2
83.782	1.15364	0	0	19.2
83.792	1.15353	1.00	1.00	19.2
83.802	1.15341	0	0	19.2
83.812	1.15330	2.00	1.41	19.2
83.822	1.15319	0	0	19.2
83.832	1.15307	0	0	19.2
83.842	1.15296	1.00	1.00	19.2
83.852	1.15285	0	0	19.2
83.862	1.15273	0	0	19.2
83.872	1.15262	0	0	19.2
83.882	1.15251	0	0	19.2
83.893	1.15240	1.00	1.00	19.2
83.903	1.15228	2.00	1.41	19.2
83.913	1.15217	0	0	19.2
83.923	1.15206	0	0	19.2
83.933	1.15194	0	0	19.2
83.943	1.15183	2.00	1.41	19.2
83.953	1.15172	0	0	19.2
83.963	1.15161	0	0	19.2
83.973	1.15149	1.00	1.00	19.2
83.983	1.15138	1.00	1.00	19.2
83.994	1.15127	2.00	1.41	19.2
84.004	1.15115	0	0	19.2
84.014	1.15104	0	0	19.2
84.024	1.15093	1.00	1.00	19.2
84.034	1.15082	0	0	19.2
84.044	1.15070	0	0	19.2
84.054	1.15059	2.00	1.41	19.2
84.064	1.15048	1.00	1.00	19.2
84.074	1.15037	0	0	19.2
84.084	1.15025	1.00	1.00	19.2
84.095	1.15014	0	0	19.2
84.105	1.15003	0	0	19.2
84.115	1.14992	0	0	19.2
84.125	1.14980	1.00	1.00	19.2
84.135	1.14969	0	0	19.2
84.145	1.14958	2.00	1.41	19.2
84.155	1.14947	2.00	1.41	19.2
84.165	1.14936	1.00	1.00	19.2
84.175	1.14924	2.00	1.41	19.2
84.185	1.14913	1.00	1.00	19.2
84.196	1.14902	1.00	1.00	19.2
84.206	1.14891	1.00	1.00	19.2
84.216	1.14879	0	0	19.2
84.226	1.14868	1.00	1.00	19.2
84.236	1.14857	0	0	19.2
84.246	1.14846	0	0	19.2
84.256	1.14835	0	0	19.2
84.266	1.14824	2.00	1.41	19.2

84.276	1.14812	0	0	19.2
84.286	1.14801	0	0	19.2
84.297	1.14790	0	0	19.2
84.307	1.14779	0	0	19.2
84.317	1.14768	2.00	1.41	19.2
84.327	1.14756	2.00	1.41	19.2
84.337	1.14745	1.00	1.00	19.2
84.347	1.14734	1.00	1.00	19.2
84.357	1.14723	0	0	19.2
84.367	1.14712	0	0	19.2
84.377	1.14701	1.00	1.00	19.2
84.387	1.14689	1.00	1.00	19.2
84.398	1.14678	1.00	1.00	19.2
84.408	1.14667	0	0	19.2
84.418	1.14656	1.00	1.00	19.2
84.428	1.14645	0	0	19.2
84.438	1.14634	0	0	19.2
84.448	1.14623	0	0	19.2
84.458	1.14612	1.00	1.00	19.2
84.468	1.14600	1.00	1.00	19.2
84.478	1.14589	0	0	19.2
84.488	1.14578	0	0	19.2
84.499	1.14567	1.00	1.00	19.2
84.509	1.14556	0	0	19.2
84.519	1.14545	0	0	19.2
84.529	1.14534	0	0	19.2
84.539	1.14523	0	0	19.2
84.549	1.14511	0	0	19.2
84.559	1.14500	0	0	19.2
84.569	1.14489	2.00	1.41	19.2
84.579	1.14478	1.00	1.00	19.2
84.589	1.14467	0	0	19.2
84.600	1.14456	0	0	19.2
84.610	1.14445	0	0	19.2
84.620	1.14434	0	0	19.2
84.630	1.14423	0	0	19.2
84.640	1.14412	1.00	1.00	19.2
84.650	1.14401	0	0	19.2
84.660	1.14390	1.00	1.00	19.2
84.670	1.14378	0	0	19.2
84.680	1.14367	1.00	1.00	19.2
84.690	1.14356	0	0	19.2
84.701	1.14345	0	0	19.2
84.711	1.14334	0	0	19.2
84.721	1.14323	1.00	1.00	19.2
84.731	1.14312	0	0	19.2
84.741	1.14301	1.00	1.00	19.2
84.751	1.14290	0	0	19.2
84.761	1.14279	0	0	19.2
84.771	1.14268	1.00	1.00	19.2

84.781	1.14257	0	0	19.2
84.791	1.14246	0	0	19.2
84.802	1.14235	1.00	1.00	19.2
84.812	1.14224	2.00	1.41	19.2
84.822	1.14213	1.00	1.00	19.2
84.832	1.14202	0	0	19.2
84.842	1.14191	0	0	19.2
84.852	1.14180	2.00	1.41	19.2
84.862	1.14169	0	0	19.2
84.872	1.14158	0	0	19.2
84.882	1.14147	0	0	19.2
84.892	1.14136	0	0	19.2
84.903	1.14125	0	0	19.2
84.913	1.14114	0	0	19.2
84.923	1.14103	0	0	19.2
84.933	1.14092	0	0	19.2
84.943	1.14081	0	0	19.2
84.953	1.14070	0	0	19.2
84.963	1.14059	0	0	19.2
84.973	1.14048	1.00	1.00	19.2
84.983	1.14037	1.00	1.00	19.2
84.993	1.14026	0	0	19.2
85.004	1.14015	0	0	19.2
85.014	1.14004	1.00	1.00	19.2
85.024	1.13993	2.00	1.41	19.2
85.034	1.13982	0	0	19.2
85.044	1.13971	0	0	19.2
85.054	1.13960	2.00	1.41	19.2
85.064	1.13949	0	0	19.2
85.074	1.13938	0	0	19.2
85.084	1.13927	2.00	1.41	19.2
85.094	1.13916	1.00	1.00	19.2
85.105	1.13906	0	0	19.2
85.115	1.13895	0	0	19.2
85.125	1.13884	1.00	1.00	19.2
85.135	1.13873	0	0	19.2
85.145	1.13862	1.00	1.00	19.2
85.155	1.13851	2.00	1.41	19.2
85.165	1.13840	0	0	19.2
85.175	1.13829	1.00	1.00	19.2
85.185	1.13818	0	0	19.2
85.195	1.13807	2.00	1.41	19.2
85.205	1.13796	0	0	19.2
85.216	1.13785	1.00	1.00	19.2
85.226	1.13774	0	0	19.2
85.236	1.13764	1.00	1.00	19.2
85.246	1.13753	0	0	19.2
85.256	1.13742	0	0	19.2
85.266	1.13731	2.00	1.41	19.2
85.276	1.13720	0	0	19.2

85.286	1.13709	0	0	19.2
85.296	1.13698	0	0	19.2
85.306	1.13687	0	0	19.2
85.317	1.13677	0	0	19.2
85.327	1.13666	0	0	19.2
85.337	1.13655	0	0	19.2
85.347	1.13644	0	0	19.2
85.357	1.13633	0	0	19.2
85.367	1.13622	0	0	19.2
85.377	1.13611	1.00	1.00	19.2
85.387	1.13600	0	0	19.2
85.397	1.13590	0	0	19.2
85.407	1.13579	0	0	19.2
85.418	1.13568	0	0	19.2
85.428	1.13557	1.00	1.00	19.2
85.438	1.13546	1.00	1.00	19.2
85.448	1.13535	0	0	19.2
85.458	1.13525	0	0	19.2
85.468	1.13514	0	0	19.2
85.478	1.13503	2.00	1.41	19.2
85.488	1.13492	0	0	19.2
85.498	1.13481	0	0	19.2
85.508	1.13470	1.00	1.00	19.2
85.519	1.13460	0	0	19.2
85.529	1.13449	0	0	19.2
85.539	1.13438	1.00	1.00	19.2
85.549	1.13427	1.00	1.00	19.2
85.559	1.13416	2.00	1.41	19.2
85.569	1.13406	0	0	19.2
85.579	1.13395	0	0	19.2
85.589	1.13384	3.00	1.73	19.2
85.599	1.13373	0	0	19.2
85.609	1.13362	1.00	1.00	19.2
85.620	1.13352	0	0	19.2
85.630	1.13341	0	0	19.2
85.640	1.13330	3.00	1.73	19.2
85.650	1.13319	0	0	19.2
85.660	1.13309	0	0	19.2
85.670	1.13298	0	0	19.2
85.680	1.13287	0	0	19.2
85.690	1.13276	0	0	19.2
85.700	1.13265	0	0	19.2
85.710	1.13255	0	0	19.2
85.721	1.13244	0	0	19.2
85.731	1.13233	0	0	19.2
85.741	1.13222	0	0	19.2
85.751	1.13212	0	0	19.2
85.761	1.13201	2.00	1.41	19.2
85.771	1.13190	1.00	1.00	19.2
85.781	1.13179	1.00	1.00	19.2

85.791	1.13169	0	0	19.2
85.801	1.13158	2.00	1.41	19.2
85.811	1.13147	1.00	1.00	19.2
85.822	1.13137	0	0	19.2
85.832	1.13126	1.00	1.00	19.2
85.842	1.13115	1.00	1.00	19.2
85.852	1.13104	0	0	19.2
85.862	1.13094	0	0	19.2
85.872	1.13083	1.00	1.00	19.2
85.882	1.13072	1.00	1.00	19.2
85.892	1.13062	1.00	1.00	19.2
85.902	1.13051	0	0	19.2
85.912	1.13040	2.00	1.41	19.2
85.923	1.13029	0	0	19.2
85.933	1.13019	0	0	19.2
85.943	1.13008	1.00	1.00	19.2
85.953	1.12997	1.00	1.00	19.2
85.963	1.12987	0	0	19.2
85.973	1.12976	1.00	1.00	19.2
85.983	1.12965	0	0	19.2
85.993	1.12955	0	0	19.2
86.003	1.12944	0	0	19.2
86.013	1.12933	1.00	1.00	19.2
86.024	1.12923	0	0	19.2
86.034	1.12912	0	0	19.2
86.044	1.12901	2.00	1.41	19.2
86.054	1.12891	1.00	1.00	19.2
86.064	1.12880	2.00	1.41	19.2
86.074	1.12869	1.00	1.00	19.2
86.084	1.12859	1.00	1.00	19.2
86.094	1.12848	1.00	1.00	19.2
86.104	1.12837	1.00	1.00	19.2
86.114	1.12827	1.00	1.00	19.2
86.125	1.12816	0	0	19.2
86.135	1.12805	1.00	1.00	19.2
86.145	1.12795	0	0	19.2
86.155	1.12784	1.00	1.00	19.2
86.165	1.12774	0	0	19.2
86.175	1.12763	1.00	1.00	19.2
86.185	1.12752	0	0	19.2
86.195	1.12742	0	0	19.2
86.205	1.12731	0	0	19.2
86.215	1.12720	0	0	19.2
86.226	1.12710	1.00	1.00	19.2
86.236	1.12699	0	0	19.2
86.246	1.12689	0	0	19.2
86.256	1.12678	1.00	1.00	19.2
86.266	1.12667	1.00	1.00	19.2
86.276	1.12657	0	0	19.2
86.286	1.12646	2.00	1.41	19.2

86.296	1.12636	1.00	1.00	19.2
86.306	1.12625	1.00	1.00	19.2
86.316	1.12614	0	0	19.2
86.327	1.12604	0	0	19.2
86.337	1.12593	0	0	19.2
86.347	1.12583	0	0	19.2
86.357	1.12572	1.00	1.00	19.2
86.367	1.12562	1.00	1.00	19.2
86.377	1.12551	1.00	1.00	19.2
86.387	1.12540	0	0	19.2
86.397	1.12530	0	0	19.2
86.407	1.12519	0	0	19.2
86.417	1.12509	3.00	1.73	19.2
86.428	1.12498	1.00	1.00	19.2
86.438	1.12488	0	0	19.2
86.448	1.12477	3.00	1.73	19.2
86.458	1.12467	1.00	1.00	19.2
86.468	1.12456	2.00	1.41	19.2
86.478	1.12445	0	0	19.2
86.488	1.12435	0	0	19.2
86.498	1.12424	0	0	19.2
86.508	1.12414	0	0	19.2
86.518	1.12403	2.00	1.41	19.2
86.528	1.12393	0	0	19.2
86.539	1.12382	0	0	19.2
86.549	1.12372	1.00	1.00	19.2
86.559	1.12361	0	0	19.2
86.569	1.12351	0	0	19.2
86.579	1.12340	2.00	1.41	19.2
86.589	1.12330	0	0	19.2
86.599	1.12319	0	0	19.2
86.609	1.12309	1.00	1.00	19.2
86.619	1.12298	0	0	19.2
86.629	1.12288	0	0	19.2
86.640	1.12277	1.00	1.00	19.2
86.650	1.12267	0	0	19.2
86.660	1.12256	1.00	1.00	19.2
86.670	1.12246	1.00	1.00	19.2
86.680	1.12235	0	0	19.2
86.690	1.12225	1.00	1.00	19.2
86.700	1.12214	1.00	1.00	19.2
86.710	1.12204	0	0	19.2
86.720	1.12193	2.00	1.41	19.2
86.730	1.12183	1.00	1.00	19.2
86.741	1.12172	1.00	1.00	19.2
86.751	1.12162	0	0	19.2
86.761	1.12151	0	0	19.2
86.771	1.12141	1.00	1.00	19.2
86.781	1.12131	0	0	19.2
86.791	1.12120	1.00	1.00	19.2

86.801	1.12110	3.00	1.73	19.2
86.811	1.12099	1.00	1.00	19.2
86.821	1.12089	1.00	1.00	19.2
86.831	1.12078	1.00	1.00	19.2
86.842	1.12068	1.00	1.00	19.2
86.852	1.12057	0	0	19.2
86.862	1.12047	0	0	19.2
86.872	1.12037	0	0	19.2
86.882	1.12026	0	0	19.2
86.892	1.12016	0	0	19.2
86.902	1.12005	0	0	19.2
86.912	1.11995	1.00	1.00	19.2
86.922	1.11985	0	0	19.2
86.932	1.11974	1.00	1.00	19.2
86.943	1.11964	1.00	1.00	19.2
86.953	1.11953	0	0	19.2
86.963	1.11943	1.00	1.00	19.2
86.973	1.11932	1.00	1.00	19.2
86.983	1.11922	1.00	1.00	19.2
86.993	1.11912	2.00	1.41	19.2
87.003	1.11901	1.00	1.00	19.2
87.013	1.11891	2.00	1.41	19.2
87.023	1.11881	0	0	19.2
87.033	1.11870	0	0	19.2
87.044	1.11860	1.00	1.00	19.2
87.054	1.11849	0	0	19.2
87.064	1.11839	1.00	1.00	19.2
87.074	1.11829	0	0	19.2
87.084	1.11818	0	0	19.2
87.094	1.11808	2.00	1.41	19.2
87.104	1.11798	0	0	19.2
87.114	1.11787	0	0	19.2
87.124	1.11777	0	0	19.2
87.134	1.11766	1.00	1.00	19.2
87.145	1.11756	0	0	19.2
87.155	1.11746	0	0	19.2
87.165	1.11735	0	0	19.2
87.175	1.11725	0	0	19.2
87.185	1.11715	0	0	19.2
87.195	1.11704	1.00	1.00	19.2
87.205	1.11694	1.00	1.00	19.2
87.215	1.11684	0	0	19.2
87.225	1.11673	0	0	19.2
87.235	1.11663	0	0	19.2
87.246	1.11653	1.00	1.00	19.2
87.256	1.11642	0	0	19.2
87.266	1.11632	0	0	19.2
87.276	1.11622	2.00	1.41	19.2
87.286	1.11611	2.00	1.41	19.2
87.296	1.11601	0	0	19.2

87.306	1.11591	0	0	19.2
87.316	1.11580	1.00	1.00	19.2
87.326	1.11570	0	0	19.2
87.336	1.11560	0	0	19.2
87.347	1.11550	0	0	19.2
87.357	1.11539	1.00	1.00	19.2
87.367	1.11529	1.00	1.00	19.2
87.377	1.11519	1.00	1.00	19.2
87.387	1.11508	1.00	1.00	19.2
87.397	1.11498	1.00	1.00	19.2
87.407	1.11488	2.00	1.41	19.2
87.417	1.11478	0	0	19.2
87.427	1.11467	0	0	19.2
87.437	1.11457	0	0	19.2
87.448	1.11447	1.00	1.00	19.2
87.458	1.11436	0	0	19.2
87.468	1.11426	0	0	19.2
87.478	1.11416	1.00	1.00	19.2
87.488	1.11406	2.00	1.41	19.2
87.498	1.11395	1.00	1.00	19.2
87.508	1.11385	0	0	19.2
87.518	1.11375	1.00	1.00	19.2
87.528	1.11365	1.00	1.00	19.2
87.538	1.11354	2.00	1.41	19.2
87.549	1.11344	0	0	19.2
87.559	1.11334	0	0	19.2
87.569	1.11324	0	0	19.2
87.579	1.11313	0	0	19.2
87.589	1.11303	0	0	19.2
87.599	1.11293	0	0	19.2
87.609	1.11283	3.00	1.73	19.2
87.619	1.11273	0	0	19.2
87.629	1.11262	1.00	1.00	19.2
87.639	1.11252	0	0	19.2
87.650	1.11242	0	0	19.2
87.660	1.11232	2.00	1.41	19.2
87.670	1.11221	0	0	19.2
87.680	1.11211	0	0	19.2
87.690	1.11201	0	0	19.2
87.700	1.11191	0	0	19.2
87.710	1.11181	0	0	19.2
87.720	1.11170	1.00	1.00	19.2
87.730	1.11160	1.00	1.00	19.2
87.740	1.11150	1.00	1.00	19.2
87.751	1.11140	0	0	19.2
87.761	1.11130	1.00	1.00	19.2
87.771	1.11120	0	0	19.2
87.781	1.11109	0	0	19.2
87.791	1.11099	1.00	1.00	19.2
87.801	1.11089	0	0	19.2

87.811	1.11079	1.00	1.00	19.2
87.821	1.11069	1.00	1.00	19.2
87.831	1.11058	1.00	1.00	19.2
87.841	1.11048	0	0	19.2
87.851	1.11038	0	0	19.2
87.862	1.11028	0	0	19.2
87.872	1.11018	1.00	1.00	19.2
87.882	1.11008	0	0	19.2
87.892	1.10998	0	0	19.2
87.902	1.10987	0	0	19.2
87.912	1.10977	0	0	19.2
87.922	1.10967	1.00	1.00	19.2
87.932	1.10957	0	0	19.2
87.942	1.10947	2.00	1.41	19.2
87.952	1.10937	0	0	19.2
87.963	1.10927	0	0	19.2
87.973	1.10916	0	0	19.2
87.983	1.10906	0	0	19.2
87.993	1.10896	0	0	19.2
88.003	1.10886	2.00	1.41	19.2
88.013	1.10876	0	0	19.2
88.023	1.10866	1.00	1.00	19.2
88.033	1.10856	0	0	19.2
88.043	1.10846	0	0	19.2
88.053	1.10835	0	0	19.2
88.064	1.10825	0	0	19.2
88.074	1.10815	0	0	19.2
88.084	1.10805	0	0	19.2
88.094	1.10795	1.00	1.00	19.2
88.104	1.10785	0	0	19.2
88.114	1.10775	0	0	19.2
88.124	1.10765	0	0	19.2
88.134	1.10755	0	0	19.2
88.144	1.10745	0	0	19.2
88.154	1.10735	1.00	1.00	19.2
88.165	1.10724	1.00	1.00	19.2
88.175	1.10714	1.00	1.00	19.2
88.185	1.10704	1.00	1.00	19.2
88.195	1.10694	0	0	19.2
88.205	1.10684	1.00	1.00	19.2
88.215	1.10674	0	0	19.2
88.225	1.10664	0	0	19.2
88.235	1.10654	0	0	19.2
88.245	1.10644	1.00	1.00	19.2
88.255	1.10634	0	0	19.2
88.266	1.10624	1.00	1.00	19.2
88.276	1.10614	1.00	1.00	19.2
88.286	1.10604	0	0	19.2
88.296	1.10594	1.00	1.00	19.2
88.306	1.10584	0	0	19.2

88.316	1.10574	0	0	19.2
88.326	1.10564	0	0	19.2
88.336	1.10554	0	0	19.2
88.346	1.10544	0	0	19.2
88.356	1.10534	0	0	19.2
88.367	1.10523	0	0	19.2
88.377	1.10513	0	0	19.2
88.387	1.10503	1.00	1.00	19.2
88.397	1.10493	0	0	19.2
88.407	1.10483	2.00	1.41	19.2
88.417	1.10473	0	0	19.2
88.427	1.10463	0	0	19.2
88.437	1.10453	3.00	1.73	19.2
88.447	1.10443	0	0	19.2
88.457	1.10433	0	0	19.2
88.468	1.10423	0	0	19.2
88.478	1.10413	0	0	19.2
88.488	1.10403	1.00	1.00	19.2
88.498	1.10393	0	0	19.2
88.508	1.10383	1.00	1.00	19.2
88.518	1.10373	1.00	1.00	19.2
88.528	1.10363	0	0	19.2
88.538	1.10354	0	0	19.2
88.548	1.10344	0	0	19.2
88.558	1.10334	1.00	1.00	19.2
88.569	1.10324	1.00	1.00	19.2
88.579	1.10314	2.00	1.41	19.2
88.589	1.10304	3.00	1.73	19.2
88.599	1.10294	0	0	19.2
88.609	1.10284	1.00	1.00	19.2
88.619	1.10274	2.00	1.41	19.2
88.629	1.10264	0	0	19.2
88.639	1.10254	0	0	19.2
88.649	1.10244	2.00	1.41	19.2
88.659	1.10234	1.00	1.00	19.2
88.670	1.10224	0	0	19.2
88.680	1.10214	1.00	1.00	19.2
88.690	1.10204	1.00	1.00	19.2
88.700	1.10194	2.00	1.41	19.2
88.710	1.10184	0	0	19.2
88.720	1.10174	0	0	19.2
88.730	1.10164	0	0	19.2
88.740	1.10154	0	0	19.2
88.750	1.10145	0	0	19.2
88.760	1.10135	0	0	19.2
88.771	1.10125	0	0	19.2
88.781	1.10115	1.00	1.00	19.2
88.791	1.10105	0	0	19.2
88.801	1.10095	0	0	19.2
88.811	1.10085	1.00	1.00	19.2

88.821	1.10075	0	0	19.2
88.831	1.10065	0	0	19.2
88.841	1.10055	0	0	19.2
88.851	1.10045	1.00	1.00	19.2
88.861	1.10036	0	0	19.2
88.872	1.10026	2.00	1.41	19.2
88.882	1.10016	2.00	1.41	19.2
88.892	1.10006	0	0	19.2
88.902	1.09996	0	0	19.2
88.912	1.09986	0	0	19.2
88.922	1.09976	1.00	1.00	19.2
88.932	1.09966	0	0	19.2
88.942	1.09957	1.00	1.00	19.2
88.952	1.09947	0	0	19.2
88.962	1.09937	0	0	19.2
88.973	1.09927	1.00	1.00	19.2
88.983	1.09917	1.00	1.00	19.2
88.993	1.09907	0	0	19.2
89.003	1.09897	3.00	1.73	19.2
89.013	1.09888	0	0	19.2
89.023	1.09878	0	0	19.2
89.033	1.09868	1.00	1.00	19.2
89.043	1.09858	0	0	19.2
89.053	1.09848	1.00	1.00	19.2
89.063	1.09838	0	0	19.2
89.074	1.09828	1.00	1.00	19.2
89.084	1.09819	0	0	19.2
89.094	1.09809	0	0	19.2
89.104	1.09799	0	0	19.2
89.114	1.09789	0	0	19.2
89.124	1.09779	0	0	19.2
89.134	1.09769	0	0	19.2
89.144	1.09760	1.00	1.00	19.2
89.154	1.09750	0	0	19.2
89.164	1.09740	0	0	19.2
89.174	1.09730	2.00	1.41	19.2
89.185	1.09720	0	0	19.2
89.195	1.09711	0	0	19.2
89.205	1.09701	0	0	19.2
89.215	1.09691	0	0	19.2
89.225	1.09681	0	0	19.2
89.235	1.09671	0	0	19.2
89.245	1.09662	0	0	19.2
89.255	1.09652	0	0	19.2
89.265	1.09642	0	0	19.2
89.275	1.09632	1.00	1.00	19.2
89.286	1.09622	1.00	1.00	19.2
89.296	1.09613	0	0	19.2
89.306	1.09603	2.00	1.41	19.2
89.316	1.09593	0	0	19.2

89.326	1.09583	0	0	19.2
89.336	1.09574	0	0	19.2
89.346	1.09564	0	0	19.2
89.356	1.09554	0	0	19.2
89.366	1.09544	1.00	1.00	19.2
89.376	1.09534	0	0	19.2
89.387	1.09525	0	0	19.2
89.397	1.09515	0	0	19.2
89.407	1.09505	0	0	19.2
89.417	1.09495	0	0	19.2
89.427	1.09486	0	0	19.2
89.437	1.09476	1.00	1.00	19.2
89.447	1.09466	0	0	19.2
89.457	1.09456	0	0	19.2
89.467	1.09447	0	0	19.2
89.477	1.09437	0	0	19.2
89.488	1.09427	1.00	1.00	19.2
89.498	1.09418	1.00	1.00	19.2
89.508	1.09408	0	0	19.2
89.518	1.09398	1.00	1.00	19.2
89.528	1.09388	1.00	1.00	19.2
89.538	1.09379	0	0	19.2
89.548	1.09369	1.00	1.00	19.2
89.558	1.09359	0	0	19.2
89.568	1.09350	0	0	19.2
89.578	1.09340	0	0	19.2
89.589	1.09330	0	0	19.2
89.599	1.09320	0	0	19.2
89.609	1.09311	0	0	19.2
89.619	1.09301	0	0	19.2
89.629	1.09291	0	0	19.2
89.639	1.09282	0	0	19.2
89.649	1.09272	0	0	19.2
89.659	1.09262	1.00	1.00	19.2
89.669	1.09253	0	0	19.2
89.679	1.09243	0	0	19.2
89.690	1.09233	0	0	19.2
89.700	1.09224	0	0	19.2
89.710	1.09214	1.00	1.00	19.2
89.720	1.09204	0	0	19.2
89.730	1.09194	0	0	19.2
89.740	1.09185	0	0	19.2
89.750	1.09175	0	0	19.2
89.760	1.09166	0	0	19.2
89.770	1.09156	0	0	19.2
89.780	1.09146	0	0	19.2
89.791	1.09137	0	0	19.2
89.801	1.09127	0	0	19.2
89.811	1.09117	0	0	19.2
89.821	1.09108	0	0	19.2

89.831	1.09098	0	0	19.2
89.841	1.09088	1.00	1.00	19.2
89.851	1.09079	0	0	19.2
89.861	1.09069	0	0	19.2
89.871	1.09059	1.00	1.00	19.2
89.881	1.09050	0	0	19.2
89.892	1.09040	0	0	19.2
89.902	1.09030	0	0	19.2
89.912	1.09021	0	0	19.2
89.922	1.09011	1.00	1.00	19.2
89.932	1.09002	1.00	1.00	19.2
89.942	1.08992	0	0	19.2
89.952	1.08982	0	0	19.2
89.962	1.08973	1.00	1.00	19.2
89.972	1.08963	0	0	19.2
89.982	1.08954	0	0	19.2
89.993	1.08944	0	0	19.2
90.003	1.08934	0	0	19.2
90.013	1.08925	1.00	1.00	19.2
90.023	1.08915	0	0	19.2
90.033	1.08906	1.00	1.00	19.2
90.043	1.08896	1.00	1.00	19.2
90.053	1.08886	0	0	19.2
90.063	1.08877	1.00	1.00	19.2
90.073	1.08867	1.00	1.00	19.2
90.083	1.08858	0	0	19.2
90.094	1.08848	0	0	19.2
90.104	1.08838	0	0	19.2
90.114	1.08829	1.00	1.00	19.2
90.124	1.08819	0	0	19.2
90.134	1.08810	0	0	19.2
90.144	1.08800	0	0	19.2
90.154	1.08791	0	0	19.2
90.164	1.08781	0	0	19.2
90.174	1.08772	2.00	1.41	19.2
90.184	1.08762	2.00	1.41	19.2
90.195	1.08752	1.00	1.00	19.2
90.205	1.08743	1.00	1.00	19.2
90.215	1.08733	0	0	19.2
90.225	1.08724	2.00	1.41	19.2
90.235	1.08714	0	0	19.2
90.245	1.08705	0	0	19.2
90.255	1.08695	0	0	19.2
90.265	1.08686	0	0	19.2
90.275	1.08676	0	0	19.2
90.285	1.08667	0	0	19.2
90.296	1.08657	0	0	19.2
90.306	1.08647	0	0	19.2
90.316	1.08638	0	0	19.2
90.326	1.08628	1.00	1.00	19.2

90.336	1.08619	0	0	19.2
90.346	1.08609	0	0	19.2
90.356	1.08600	1.00	1.00	19.2
90.366	1.08590	0	0	19.2
90.376	1.08581	3.00	1.73	19.2
90.386	1.08571	0	0	19.2
90.397	1.08562	0	0	19.2
90.407	1.08552	0	0	19.2
90.417	1.08543	1.00	1.00	19.2
90.427	1.08533	0	0	19.2
90.437	1.08524	0	0	19.2
90.447	1.08514	1.00	1.00	19.2
90.457	1.08505	0	0	19.2
90.467	1.08495	0	0	19.2
90.477	1.08486	1.00	1.00	19.2
90.487	1.08476	0	0	19.2
90.497	1.08467	0	0	19.2
90.508	1.08458	1.00	1.00	19.2
90.518	1.08448	0	0	19.2
90.528	1.08439	1.00	1.00	19.2
90.538	1.08429	1.00	1.00	19.2
90.548	1.08420	0	0	19.2
90.558	1.08410	0	0	19.2
90.568	1.08401	0	0	19.2
90.578	1.08391	0	0	19.2
90.588	1.08382	0	0	19.2
90.598	1.08372	0	0	19.2
90.609	1.08363	2.00	1.41	19.2
90.619	1.08353	0	0	19.2
90.629	1.08344	0	0	19.2
90.639	1.08335	0	0	19.2
90.649	1.08325	2.00	1.41	19.2
90.659	1.08316	0	0	19.2
90.669	1.08306	2.00	1.41	19.2
90.679	1.08297	1.00	1.00	19.2
90.689	1.08287	0	0	19.2
90.699	1.08278	0	0	19.2
90.710	1.08269	0	0	19.2
90.720	1.08259	0	0	19.2
90.730	1.08250	2.00	1.41	19.2
90.740	1.08240	0	0	19.2
90.750	1.08231	0	0	19.2
90.760	1.08221	1.00	1.00	19.2
90.770	1.08212	0	0	19.2
90.780	1.08203	1.00	1.00	19.2
90.790	1.08193	1.00	1.00	19.2
90.800	1.08184	0	0	19.2
90.811	1.08174	2.00	1.41	19.2
90.821	1.08165	0	0	19.2
90.831	1.08156	0	0	19.2

90.841	1.08146	0	0	19.2
90.851	1.08137	1.00	1.00	19.2
90.861	1.08127	0	0	19.2
90.871	1.08118	0	0	19.2
90.881	1.08109	0	0	19.2
90.891	1.08099	0	0	19.2
90.901	1.08090	0	0	19.2
90.912	1.08081	1.00	1.00	19.2
90.922	1.08071	1.00	1.00	19.2
90.932	1.08062	0	0	19.2
90.942	1.08052	0	0	19.2
90.952	1.08043	0	0	19.2
90.962	1.08034	0	0	19.2
90.972	1.08024	0	0	19.2
90.982	1.08015	1.00	1.00	19.2
90.992	1.08006	1.00	1.00	19.2
91.002	1.07996	0	0	19.2
91.013	1.07987	0	0	19.2
91.023	1.07978	1.00	1.00	19.2
91.033	1.07968	0	0	19.2
91.043	1.07959	0	0	19.2
91.053	1.07950	0	0	19.2
91.063	1.07940	0	0	19.2
91.073	1.07931	0	0	19.2
91.083	1.07922	0	0	19.2
91.093	1.07912	1.00	1.00	19.2
91.103	1.07903	0	0	19.2
91.114	1.07894	0	0	19.2
91.124	1.07884	0	0	19.2
91.134	1.07875	0	0	19.2
91.144	1.07866	0	0	19.2
91.154	1.07856	3.00	1.73	19.2
91.164	1.07847	0	0	19.2
91.174	1.07838	0	0	19.2
91.184	1.07828	0	0	19.2
91.194	1.07819	0	0	19.2
91.204	1.07810	1.00	1.00	19.2
91.215	1.07800	0	0	19.2
91.225	1.07791	1.00	1.00	19.2
91.235	1.07782	0	0	19.2
91.245	1.07772	0	0	19.2
91.255	1.07763	1.00	1.00	19.2
91.265	1.07754	1.00	1.00	19.2
91.275	1.07745	0	0	19.2
91.285	1.07735	1.00	1.00	19.2
91.295	1.07726	1.00	1.00	19.2
91.305	1.07717	1.00	1.00	19.2
91.316	1.07707	0	0	19.2
91.326	1.07698	2.00	1.41	19.2
91.336	1.07689	0	0	19.2

91.346	1.07680	1.00	1.00	19.2
91.356	1.07670	0	0	19.2
91.366	1.07661	0	0	19.2
91.376	1.07652	0	0	19.2
91.386	1.07643	0	0	19.2
91.396	1.07633	0	0	19.2
91.406	1.07624	0	0	19.2
91.417	1.07615	0	0	19.2
91.427	1.07606	0	0	19.2
91.437	1.07596	0	0	19.2
91.447	1.07587	0	0	19.2
91.457	1.07578	0	0	19.2
91.467	1.07569	0	0	19.2
91.477	1.07559	0	0	19.2
91.487	1.07550	0	0	19.2
91.497	1.07541	0	0	19.2
91.507	1.07532	0	0	19.2
91.518	1.07522	0	0	19.2
91.528	1.07513	1.00	1.00	19.2
91.538	1.07504	0	0	19.2
91.548	1.07495	1.00	1.00	19.2
91.558	1.07486	0	0	19.2
91.568	1.07476	0	0	19.2
91.578	1.07467	0	0	19.2
91.588	1.07458	1.00	1.00	19.2
91.598	1.07449	0	0	19.2
91.608	1.07439	0	0	19.2
91.619	1.07430	1.00	1.00	19.2
91.629	1.07421	0	0	19.2
91.639	1.07412	0	0	19.2
91.649	1.07403	0	0	19.2
91.659	1.07393	0	0	19.2
91.669	1.07384	0	0	19.2
91.679	1.07375	0	0	19.2
91.689	1.07366	1.00	1.00	19.2
91.699	1.07357	1.00	1.00	19.2
91.709	1.07348	1.00	1.00	19.2
91.719	1.07338	0	0	19.2
91.730	1.07329	1.00	1.00	19.2
91.740	1.07320	0	0	19.2
91.750	1.07311	0	0	19.2
91.760	1.07302	2.00	1.41	19.2
91.770	1.07292	0	0	19.2
91.780	1.07283	0	0	19.2
91.790	1.07274	0	0	19.2
91.800	1.07265	0	0	19.2
91.810	1.07256	0	0	19.2
91.820	1.07247	0	0	19.2
91.831	1.07238	0	0	19.2
91.841	1.07228	0	0	19.2

91.851	1.07219	0	0	19.2
91.861	1.07210	0	0	19.2
91.871	1.07201	0	0	19.2
91.881	1.07192	1.00	1.00	19.2
91.891	1.07183	0	0	19.2
91.901	1.07173	0	0	19.2
91.911	1.07164	0	0	19.2
91.921	1.07155	1.00	1.00	19.2
91.932	1.07146	0	0	19.2
91.942	1.07137	1.00	1.00	19.2
91.952	1.07128	0	0	19.2
91.962	1.07119	0	0	19.2
91.972	1.07110	0	0	19.2
91.982	1.07100	0	0	19.2
91.992	1.07091	0	0	19.2
92.002	1.07082	1.00	1.00	19.2
92.012	1.07073	0	0	19.2
92.022	1.07064	1.00	1.00	19.2
92.033	1.07055	0	0	19.2
92.043	1.07046	0	0	19.2
92.053	1.07037	0	0	19.2
92.063	1.07028	1.00	1.00	19.2
92.073	1.07019	0	0	19.2
92.083	1.07009	0	0	19.2
92.093	1.07000	0	0	19.2
92.103	1.06991	0	0	19.2
92.113	1.06982	0	0	19.2
92.123	1.06973	0	0	19.2
92.134	1.06964	0	0	19.2
92.144	1.06955	0	0	19.2
92.154	1.06946	1.00	1.00	19.2
92.164	1.06937	2.00	1.41	19.2
92.174	1.06928	1.00	1.00	19.2
92.184	1.06919	0	0	19.2
92.194	1.06910	0	0	19.2
92.204	1.06900	0	0	19.2
92.214	1.06891	1.00	1.00	19.2
92.224	1.06882	1.00	1.00	19.2
92.235	1.06873	0	0	19.2
92.245	1.06864	1.00	1.00	19.2
92.255	1.06855	0	0	19.2
92.265	1.06846	0	0	19.2
92.275	1.06837	0	0	19.2
92.285	1.06828	0	0	19.2
92.295	1.06819	0	0	19.2
92.305	1.06810	0	0	19.2
92.315	1.06801	0	0	19.2
92.325	1.06792	1.00	1.00	19.2
92.336	1.06783	0	0	19.2
92.346	1.06774	1.00	1.00	19.2

92.356	1.06765	2.00	1.41	19.2
92.366	1.06756	0	0	19.2
92.376	1.06747	0	0	19.2
92.386	1.06738	0	0	19.2
92.396	1.06729	0	0	19.2
92.406	1.06720	0	0	19.2
92.416	1.06711	0	0	19.2
92.426	1.06702	1.00	1.00	19.2
92.437	1.06693	0	0	19.2
92.447	1.06684	1.00	1.00	19.2
92.457	1.06675	0	0	19.2
92.467	1.06666	0	0	19.2
92.477	1.06657	0	0	19.2
92.487	1.06648	0	0	19.2
92.497	1.06639	0	0	19.2
92.507	1.06630	0	0	19.2
92.517	1.06621	0	0	19.2
92.527	1.06612	0	0	19.2
92.538	1.06603	1.00	1.00	19.2
92.548	1.06594	0	0	19.2
92.558	1.06585	0	0	19.2
92.568	1.06576	1.00	1.00	19.2
92.578	1.06567	0	0	19.2
92.588	1.06558	0	0	19.2
92.598	1.06549	0	0	19.2
92.608	1.06540	0	0	19.2
92.618	1.06531	1.00	1.00	19.2
92.628	1.06522	1.00	1.00	19.2
92.639	1.06513	0	0	19.2
92.649	1.06504	0	0	19.2
92.659	1.06495	1.00	1.00	19.2
92.669	1.06486	0	0	19.2
92.679	1.06477	0	0	19.2
92.689	1.06468	1.00	1.00	19.2
92.699	1.06459	0	0	19.2
92.709	1.06450	0	0	19.2
92.719	1.06441	0	0	19.2
92.729	1.06432	0	0	19.2
92.740	1.06423	0	0	19.2
92.750	1.06414	0	0	19.2
92.760	1.06405	1.00	1.00	19.2
92.770	1.06396	0	0	19.2
92.780	1.06388	0	0	19.2
92.790	1.06379	0	0	19.2
92.800	1.06370	0	0	19.2
92.810	1.06361	1.00	1.00	19.2
92.820	1.06352	1.00	1.00	19.2
92.830	1.06343	1.00	1.00	19.2
92.841	1.06334	0	0	19.2
92.851	1.06325	0	0	19.2

92.861	1.06316	0	0	19.2
92.871	1.06307	0	0	19.2
92.881	1.06298	0	0	19.2
92.891	1.06289	0	0	19.2
92.901	1.06281	0	0	19.2
92.911	1.06272	0	0	19.2
92.921	1.06263	0	0	19.2
92.931	1.06254	0	0	19.2
92.942	1.06245	0	0	19.2
92.952	1.06236	0	0	19.2
92.962	1.06227	1.00	1.00	19.2
92.972	1.06218	0	0	19.2
92.982	1.06209	0	0	19.2
92.992	1.06200	1.00	1.00	19.2
93.002	1.06192	0	0	19.2
93.012	1.06183	0	0	19.2
93.022	1.06174	0	0	19.2
93.032	1.06165	0	0	19.2
93.042	1.06156	1.00	1.00	19.2
93.053	1.06147	0	0	19.2
93.063	1.06138	1.00	1.00	19.2
93.073	1.06129	0	0	19.2
93.083	1.06121	0	0	19.2
93.093	1.06112	0	0	19.2
93.103	1.06103	0	0	19.2
93.113	1.06094	0	0	19.2
93.123	1.06085	1.00	1.00	19.2
93.133	1.06076	0	0	19.2
93.143	1.06067	0	0	19.2
93.154	1.06059	0	0	19.2
93.164	1.06050	0	0	19.2
93.174	1.06041	0	0	19.2
93.184	1.06032	1.00	1.00	19.2
93.194	1.06023	1.00	1.00	19.2
93.204	1.06014	0	0	19.2
93.214	1.06006	0	0	19.2
93.224	1.05997	0	0	19.2
93.234	1.05988	1.00	1.00	19.2
93.244	1.05979	0	0	19.2
93.255	1.05970	0	0	19.2
93.265	1.05961	0	0	19.2
93.275	1.05953	0	0	19.2
93.285	1.05944	0	0	19.2
93.295	1.05935	0	0	19.2
93.305	1.05926	0	0	19.2
93.315	1.05917	0	0	19.2
93.325	1.05909	1.00	1.00	19.2
93.335	1.05900	0	0	19.2
93.345	1.05891	0	0	19.2
93.356	1.05882	0	0	19.2

93.366	1.05873	0	0	19.2
93.376	1.05865	0	0	19.2
93.386	1.05856	1.00	1.00	19.2
93.396	1.05847	0	0	19.2
93.406	1.05838	0	0	19.2
93.416	1.05829	1.00	1.00	19.2
93.426	1.05821	1.00	1.00	19.2
93.436	1.05812	0	0	19.2
93.446	1.05803	0	0	19.2
93.457	1.05794	0	0	19.2
93.467	1.05785	0	0	19.2
93.477	1.05777	1.00	1.00	19.2
93.487	1.05768	0	0	19.2
93.497	1.05759	0	0	19.2
93.507	1.05750	0	0	19.2
93.517	1.05742	0	0	19.2
93.527	1.05733	0	0	19.2
93.537	1.05724	0	0	19.2
93.547	1.05715	0	0	19.2
93.558	1.05707	0	0	19.2
93.568	1.05698	0	0	19.2
93.578	1.05689	0	0	19.2
93.588	1.05680	0	0	19.2
93.598	1.05672	1.00	1.00	19.2
93.608	1.05663	0	0	19.2
93.618	1.05654	0	0	19.2
93.628	1.05645	0	0	19.2
93.638	1.05637	0	0	19.2
93.648	1.05628	0	0	19.2
93.659	1.05619	0	0	19.2
93.669	1.05610	0	0	19.2
93.679	1.05602	0	0	19.2
93.689	1.05593	0	0	19.2
93.699	1.05584	1.00	1.00	19.2
93.709	1.05576	0	0	19.2
93.719	1.05567	1.00	1.00	19.2
93.729	1.05558	0	0	19.2
93.739	1.05549	0	0	19.2
93.749	1.05541	1.00	1.00	19.2
93.760	1.05532	0	0	19.2
93.770	1.05523	0	0	19.2
93.780	1.05515	0	0	19.2
93.790	1.05506	0	0	19.2
93.800	1.05497	0	0	19.2
93.810	1.05488	0	0	19.2
93.820	1.05480	0	0	19.2
93.830	1.05471	0	0	19.2
93.840	1.05462	1.00	1.00	19.2
93.850	1.05454	0	0	19.2
93.861	1.05445	0	0	19.2

93.871	1.05436	0	0	19.2
93.881	1.05428	0	0	19.2
93.891	1.05419	0	0	19.2
93.901	1.05410	1.00	1.00	19.2
93.911	1.05402	0	0	19.2
93.921	1.05393	2.00	1.41	19.2
93.931	1.05384	0	0	19.2
93.941	1.05376	0	0	19.2
93.951	1.05367	0	0	19.2
93.962	1.05358	0	0	19.2
93.972	1.05350	0	0	19.2
93.982	1.05341	0	0	19.2
93.992	1.05332	1.00	1.00	19.2
94.002	1.05324	0	0	19.2
94.012	1.05315	0	0	19.2
94.022	1.05306	3.00	1.73	19.2
94.032	1.05298	1.00	1.00	19.2
94.042	1.05289	0	0	19.2
94.052	1.05280	0	0	19.2
94.063	1.05272	2.00	1.41	19.2
94.073	1.05263	0	0	19.2
94.083	1.05254	1.00	1.00	19.2
94.093	1.05246	0	0	19.2
94.103	1.05237	0	0	19.2
94.113	1.05229	0	0	19.2
94.123	1.05220	1.00	1.00	19.2
94.133	1.05211	0	0	19.2
94.143	1.05203	0	0	19.2
94.153	1.05194	0	0	19.2
94.164	1.05185	0	0	19.2
94.174	1.05177	0	0	19.2
94.184	1.05168	0	0	19.2
94.194	1.05160	0	0	19.2
94.204	1.05151	0	0	19.2
94.214	1.05142	0	0	19.2
94.224	1.05134	0	0	19.2
94.234	1.05125	0	0	19.2
94.244	1.05116	0	0	19.2
94.254	1.05108	0	0	19.2
94.265	1.05099	1.00	1.00	19.2
94.275	1.05091	0	0	19.2
94.285	1.05082	0	0	19.2
94.295	1.05074	1.00	1.00	19.2
94.305	1.05065	0	0	19.2
94.315	1.05056	0	0	19.2
94.325	1.05048	0	0	19.2
94.335	1.05039	0	0	19.2
94.345	1.05031	0	0	19.2
94.355	1.05022	0	0	19.2
94.365	1.05013	0	0	19.2

94.376	1.05005	0	0	19.2
94.386	1.04996	0	0	19.2
94.396	1.04988	0	0	19.2
94.406	1.04979	1.00	1.00	19.2
94.416	1.04971	0	0	19.2
94.426	1.04962	1.00	1.00	19.2
94.436	1.04953	0	0	19.2
94.446	1.04945	3.00	1.73	19.2
94.456	1.04936	0	0	19.2
94.466	1.04928	2.00	1.41	19.2
94.477	1.04919	1.00	1.00	19.2
94.487	1.04911	0	0	19.2
94.497	1.04902	0	0	19.2
94.507	1.04894	2.00	1.41	19.2
94.517	1.04885	0	0	19.2
94.527	1.04876	0	0	19.2
94.537	1.04868	1.00	1.00	19.2
94.547	1.04859	0	0	19.2
94.557	1.04851	0	0	19.2
94.567	1.04842	0	0	19.2
94.578	1.04834	1.00	1.00	19.2
94.588	1.04825	0	0	19.2
94.598	1.04817	0	0	19.2
94.608	1.04808	0	0	19.2
94.618	1.04800	0	0	19.2
94.628	1.04791	1.00	1.00	19.2
94.638	1.04783	1.00	1.00	19.2
94.648	1.04774	0	0	19.2
94.658	1.04766	0	0	19.2
94.668	1.04757	0	0	19.2
94.679	1.04749	0	0	19.2
94.689	1.04740	0	0	19.2
94.699	1.04732	0	0	19.2
94.709	1.04723	1.00	1.00	19.2
94.719	1.04715	0	0	19.2
94.729	1.04706	1.00	1.00	19.2
94.739	1.04698	0	0	19.2
94.749	1.04689	0	0	19.2
94.759	1.04681	0	0	19.2
94.769	1.04672	0	0	19.2
94.780	1.04664	0	0	19.2
94.790	1.04655	1.00	1.00	19.2
94.800	1.04647	0	0	19.2
94.810	1.04638	0	0	19.2
94.820	1.04630	0	0	19.2
94.830	1.04621	0	0	19.2
94.840	1.04613	0	0	19.2
94.850	1.04604	0	0	19.2
94.860	1.04596	2.00	1.41	19.2
94.870	1.04587	0	0	19.2

94.881	1.04579	0	0	19.2
94.891	1.04570	0	0	19.2
94.901	1.04562	0	0	19.2
94.911	1.04554	0	0	19.2
94.921	1.04545	1.00	1.00	19.2
94.931	1.04537	0	0	19.2
94.941	1.04528	1.00	1.00	19.2
94.951	1.04520	1.00	1.00	19.2
94.961	1.04511	0	0	19.2
94.971	1.04503	0	0	19.2
94.982	1.04494	0	0	19.2
94.992	1.04486	0	0	19.2
95.002	1.04478	0	0	19.2
95.012	1.04469	0	0	19.2
95.022	1.04461	2.00	1.41	19.2
95.032	1.04452	0	0	19.2
95.042	1.04444	0	0	19.2
95.052	1.04435	0	0	19.2
95.062	1.04427	0	0	19.2
95.072	1.04419	1.00	1.00	19.2
95.083	1.04410	0	0	19.2
95.093	1.04402	0	0	19.2
95.103	1.04393	0	0	19.2
95.113	1.04385	0	0	19.2
95.123	1.04376	0	0	19.2
95.133	1.04368	1.00	1.00	19.2
95.143	1.04360	0	0	19.2
95.153	1.04351	1.00	1.00	19.2
95.163	1.04343	0	0	19.2
95.173	1.04334	1.00	1.00	19.2
95.184	1.04326	0	0	19.2
95.194	1.04318	1.00	1.00	19.2
95.204	1.04309	0	0	19.2
95.214	1.04301	0	0	19.2
95.224	1.04292	0	0	19.2
95.234	1.04284	0	0	19.2
95.244	1.04276	1.00	1.00	19.2
95.254	1.04267	0	0	19.2
95.264	1.04259	0	0	19.2
95.274	1.04251	0	0	19.2
95.285	1.04242	0	0	19.2
95.295	1.04234	0	0	19.2
95.305	1.04225	0	0	19.2
95.315	1.04217	0	0	19.2
95.325	1.04209	0	0	19.2
95.335	1.04200	0	0	19.2
95.345	1.04192	0	0	19.2
95.355	1.04184	0	0	19.2
95.365	1.04175	0	0	19.2
95.375	1.04167	0	0	19.2

95.386	1.04158	0	0	19.2
95.396	1.04150	0	0	19.2
95.406	1.04142	1.00	1.00	19.2
95.416	1.04133	0	0	19.2
95.426	1.04125	0	0	19.2
95.436	1.04117	1.00	1.00	19.2
95.446	1.04108	1.00	1.00	19.2
95.456	1.04100	1.00	1.00	19.2
95.466	1.04092	0	0	19.2
95.476	1.04083	0	0	19.2
95.487	1.04075	0	0	19.2
95.497	1.04067	1.00	1.00	19.2
95.507	1.04058	0	0	19.2
95.517	1.04050	1.00	1.00	19.2
95.527	1.04042	1.00	1.00	19.2
95.537	1.04033	1.00	1.00	19.2
95.547	1.04025	0	0	19.2
95.557	1.04017	0	0	19.2
95.567	1.04008	0	0	19.2
95.577	1.04000	0	0	19.2
95.588	1.03992	1.00	1.00	19.2
95.598	1.03984	0	0	19.2
95.608	1.03975	0	0	19.2
95.618	1.03967	0	0	19.2
95.628	1.03959	0	0	19.2
95.638	1.03950	0	0	19.2
95.648	1.03942	0	0	19.2
95.658	1.03934	0	0	19.2
95.668	1.03925	1.00	1.00	19.2
95.678	1.03917	1.00	1.00	19.2
95.688	1.03909	0	0	19.2
95.699	1.03901	0	0	19.2
95.709	1.03892	0	0	19.2
95.719	1.03884	0	0	19.2
95.729	1.03876	0	0	19.2
95.739	1.03867	0	0	19.2
95.749	1.03859	0	0	19.2
95.759	1.03851	1.00	1.00	19.2
95.769	1.03843	0	0	19.2
95.779	1.03834	0	0	19.2
95.789	1.03826	0	0	19.2
95.800	1.03818	0	0	19.2
95.810	1.03809	0	0	19.2
95.820	1.03801	0	0	19.2
95.830	1.03793	1.00	1.00	19.2
95.840	1.03785	0	0	19.2
95.850	1.03776	0	0	19.2
95.860	1.03768	0	0	19.2
95.870	1.03760	0	0	19.2
95.880	1.03752	0	0	19.2

95.890	1.03743	1.00	1.00	19.2
95.901	1.03735	1.00	1.00	19.2
95.911	1.03727	1.00	1.00	19.2
95.921	1.03719	0	0	19.2
95.931	1.03710	0	0	19.2
95.941	1.03702	0	0	19.2
95.951	1.03694	0	0	19.2
95.961	1.03686	0	0	19.2
95.971	1.03677	0	0	19.2
95.981	1.03669	0	0	19.2
95.991	1.03661	0	0	19.2
96.002	1.03653	0	0	19.2
96.012	1.03645	0	0	19.2
96.022	1.03636	0	0	19.2
96.032	1.03628	0	0	19.2
96.042	1.03620	1.00	1.00	19.2
96.052	1.03612	0	0	19.2
96.062	1.03603	0	0	19.2
96.072	1.03595	0	0	19.2
96.082	1.03587	0	0	19.2
96.092	1.03579	1.00	1.00	19.2
96.103	1.03571	1.00	1.00	19.2
96.113	1.03562	1.00	1.00	19.2
96.123	1.03554	0	0	19.2
96.133	1.03546	0	0	19.2
96.143	1.03538	0	0	19.2
96.153	1.03530	0	0	19.2
96.163	1.03521	0	0	19.2
96.173	1.03513	0	0	19.2
96.183	1.03505	1.00	1.00	19.2
96.193	1.03497	0	0	19.2
96.204	1.03489	0	0	19.2
96.214	1.03481	0	0	19.2
96.224	1.03472	0	0	19.2
96.234	1.03464	0	0	19.2
96.244	1.03456	0	0	19.2
96.254	1.03448	0	0	19.2
96.264	1.03440	1.00	1.00	19.2
96.274	1.03431	0	0	19.2
96.284	1.03423	0	0	19.2
96.294	1.03415	0	0	19.2
96.305	1.03407	1.00	1.00	19.2
96.315	1.03399	0	0	19.2
96.325	1.03391	0	0	19.2
96.335	1.03383	0	0	19.2
96.345	1.03374	1.00	1.00	19.2
96.355	1.03366	0	0	19.2
96.365	1.03358	1.00	1.00	19.2
96.375	1.03350	0	0	19.2
96.385	1.03342	0	0	19.2

96.395	1.03334	0	0	19.2
96.406	1.03325	0	0	19.2
96.416	1.03317	0	0	19.2
96.426	1.03309	1.00	1.00	19.2
96.436	1.03301	0	0	19.2
96.446	1.03293	0	0	19.2
96.456	1.03285	0	0	19.2
96.466	1.03277	0	0	19.2
96.476	1.03269	0	0	19.2
96.486	1.03260	0	0	19.2
96.496	1.03252	0	0	19.2
96.507	1.03244	1.00	1.00	19.2
96.517	1.03236	1.00	1.00	19.2
96.527	1.03228	0	0	19.2
96.537	1.03220	0	0	19.2
96.547	1.03212	1.00	1.00	19.2
96.557	1.03204	1.00	1.00	19.2
96.567	1.03195	0	0	19.2
96.577	1.03187	0	0	19.2
96.587	1.03179	0	0	19.2
96.597	1.03171	0	0	19.2
96.608	1.03163	0	0	19.2
96.618	1.03155	0	0	19.2
96.628	1.03147	0	0	19.2
96.638	1.03139	1.00	1.00	19.2
96.648	1.03131	1.00	1.00	19.2
96.658	1.03123	0	0	19.2
96.668	1.03115	0	0	19.2
96.678	1.03106	0	0	19.2
96.688	1.03098	0	0	19.2
96.698	1.03090	0	0	19.2
96.709	1.03082	0	0	19.2
96.719	1.03074	0	0	19.2
96.729	1.03066	0	0	19.2
96.739	1.03058	1.00	1.00	19.2
96.749	1.03050	0	0	19.2
96.759	1.03042	0	0	19.2
96.769	1.03034	0	0	19.2
96.779	1.03026	1.00	1.00	19.2
96.789	1.03018	0	0	19.2
96.799	1.03010	0	0	19.2
96.810	1.03001	1.00	1.00	19.2
96.820	1.02993	0	0	19.2
96.830	1.02985	1.00	1.00	19.2
96.840	1.02977	0	0	19.2
96.850	1.02969	0	0	19.2
96.860	1.02961	0	0	19.2
96.870	1.02953	0	0	19.2
96.880	1.02945	0	0	19.2
96.890	1.02937	0	0	19.2

96.900	1.02929	0	0	19.2
96.911	1.02921	0	0	19.2
96.921	1.02913	0	0	19.2
96.931	1.02905	0	0	19.2
96.941	1.02897	0	0	19.2
96.951	1.02889	0	0	19.2
96.961	1.02881	1.00	1.00	19.2
96.971	1.02873	0	0	19.2
96.981	1.02865	0	0	19.2
96.991	1.02857	0	0	19.2
97.001	1.02849	0	0	19.2
97.011	1.02841	0	0	19.2
97.022	1.02833	0	0	19.2
97.032	1.02825	0	0	19.2
97.042	1.02817	0	0	19.2
97.052	1.02809	0	0	19.2
97.062	1.02801	0	0	19.2
97.072	1.02793	0	0	19.2
97.082	1.02785	0	0	19.2
97.092	1.02777	0	0	19.2
97.102	1.02769	0	0	19.2
97.112	1.02761	0	0	19.2
97.123	1.02753	0	0	19.2
97.133	1.02745	0	0	19.2
97.143	1.02737	0	0	19.2
97.153	1.02729	0	0	19.2
97.163	1.02721	0	0	19.2
97.173	1.02713	0	0	19.2
97.183	1.02705	0	0	19.2
97.193	1.02697	0	0	19.2
97.203	1.02689	1.00	1.00	19.2
97.213	1.02681	0	0	19.2
97.224	1.02673	0	0	19.2
97.234	1.02665	0	0	19.2
97.244	1.02657	0	0	19.2
97.254	1.02649	1.00	1.00	19.2
97.264	1.02641	0	0	19.2
97.274	1.02633	0	0	19.2
97.284	1.02625	1.00	1.00	19.2
97.294	1.02617	0	0	19.2
97.304	1.02609	0	0	19.2
97.314	1.02601	0	0	19.2
97.325	1.02593	0	0	19.2
97.335	1.02585	0	0	19.2
97.345	1.02577	0	0	19.2
97.355	1.02569	0	0	19.2
97.365	1.02561	0	0	19.2
97.375	1.02553	0	0	19.2
97.385	1.02546	0	0	19.2
97.395	1.02538	0	0	19.2

97.405	1.02530	0	0	19.2
97.415	1.02522	0	0	19.2
97.426	1.02514	0	0	19.2
97.436	1.02506	1.00	1.00	19.2
97.446	1.02498	0	0	19.2
97.456	1.02490	0	0	19.2
97.466	1.02482	0	0	19.2
97.476	1.02474	1.00	1.00	19.2
97.486	1.02466	0	0	19.2
97.496	1.02458	0	0	19.2
97.506	1.02450	0	0	19.2
97.516	1.02442	1.00	1.00	19.2
97.527	1.02435	0	0	19.2
97.537	1.02427	0	0	19.2
97.547	1.02419	0	0	19.2
97.557	1.02411	0	0	19.2
97.567	1.02403	0	0	19.2
97.577	1.02395	1.00	1.00	19.2
97.587	1.02387	0	0	19.2
97.597	1.02379	0	0	19.2
97.607	1.02371	1.00	1.00	19.2
97.617	1.02363	0	0	19.2
97.628	1.02355	0	0	19.2
97.638	1.02348	3.00	1.73	19.2
97.648	1.02340	0	0	19.2
97.658	1.02332	0	0	19.2
97.668	1.02324	0	0	19.2
97.678	1.02316	2.00	1.41	19.2
97.688	1.02308	1.00	1.00	19.2
97.698	1.02300	0	0	19.2
97.708	1.02292	1.00	1.00	19.2
97.718	1.02285	0	0	19.2
97.729	1.02277	0	0	19.2
97.739	1.02269	0	0	19.2
97.749	1.02261	0	0	19.2
97.759	1.02253	0	0	19.2
97.769	1.02245	0	0	19.2
97.779	1.02237	0	0	19.2
97.789	1.02229	0	0	19.2
97.799	1.02222	1.00	1.00	19.2
97.809	1.02214	0	0	19.2
97.819	1.02206	0	0	19.2
97.830	1.02198	0	0	19.2
97.840	1.02190	0	0	19.2
97.850	1.02182	1.00	1.00	19.2
97.860	1.02174	0	0	19.2
97.870	1.02167	0	0	19.2
97.880	1.02159	0	0	19.2
97.890	1.02151	0	0	19.2
97.900	1.02143	0	0	19.2

97.910	1.02135	0	0	19.2
97.920	1.02127	0	0	19.2
97.931	1.02120	0	0	19.2
97.941	1.02112	0	0	19.2
97.951	1.02104	0	0	19.2
97.961	1.02096	0	0	19.2
97.971	1.02088	0	0	19.2
97.981	1.02080	0	0	19.2
97.991	1.02073	0	0	19.2
98.001	1.02065	0	0	19.2
98.011	1.02057	0	0	19.2
98.021	1.02049	0	0	19.2
98.032	1.02041	0	0	19.2
98.042	1.02034	0	0	19.2
98.052	1.02026	2.00	1.41	19.2
98.062	1.02018	0	0	19.2
98.072	1.02010	0	0	19.2
98.082	1.02002	0	0	19.2
98.092	1.01995	1.00	1.00	19.2
98.102	1.01987	1.00	1.00	19.2
98.112	1.01979	0	0	19.2
98.122	1.01971	0	0	19.2
98.133	1.01963	0	0	19.2
98.143	1.01956	0	0	19.2
98.153	1.01948	0	0	19.2
98.163	1.01940	0	0	19.2
98.173	1.01932	0	0	19.2
98.183	1.01924	0	0	19.2
98.193	1.01917	0	0	19.2
98.203	1.01909	0	0	19.2
98.213	1.01901	0	0	19.2
98.223	1.01893	0	0	19.2
98.234	1.01885	0	0	19.2
98.244	1.01878	0	0	19.2
98.254	1.01870	0	0	19.2
98.264	1.01862	0	0	19.2
98.274	1.01854	0	0	19.2
98.284	1.01847	0	0	19.2
98.294	1.01839	0	0	19.2
98.304	1.01831	0	0	19.2
98.314	1.01823	0	0	19.2
98.324	1.01816	0	0	19.2
98.334	1.01808	0	0	19.2
98.345	1.01800	1.00	1.00	19.2
98.355	1.01792	0	0	19.2
98.365	1.01785	0	0	19.2
98.375	1.01777	0	0	19.2
98.385	1.01769	1.00	1.00	19.2
98.395	1.01761	0	0	19.2
98.405	1.01754	0	0	19.2

98.415	1.01746	0	0	19.2
98.425	1.01738	1.00	1.00	19.2
98.435	1.01730	0	0	19.2
98.446	1.01723	0	0	19.2
98.456	1.01715	0	0	19.2
98.466	1.01707	0	0	19.2
98.476	1.01699	0	0	19.2
98.486	1.01692	0	0	19.2
98.496	1.01684	2.00	1.41	19.2
98.506	1.01676	1.00	1.00	19.2
98.516	1.01669	0	0	19.2
98.526	1.01661	0	0	19.2
98.536	1.01653	0	0	19.2
98.547	1.01645	0	0	19.2
98.557	1.01638	0	0	19.2
98.567	1.01630	1.00	1.00	19.2
98.577	1.01622	0	0	19.2
98.587	1.01615	1.00	1.00	19.2
98.597	1.01607	1.00	1.00	19.2
98.607	1.01599	0	0	19.2
98.617	1.01592	1.00	1.00	19.2
98.627	1.01584	0	0	19.2
98.637	1.01576	0	0	19.2
98.648	1.01568	0	0	19.2
98.658	1.01561	0	0	19.2
98.668	1.01553	0	0	19.2
98.678	1.01545	0	0	19.2
98.688	1.01538	0	0	19.2
98.698	1.01530	0	0	19.2
98.708	1.01522	0	0	19.2
98.718	1.01515	0	0	19.2
98.728	1.01507	0	0	19.2
98.738	1.01499	0	0	19.2
98.749	1.01492	0	0	19.2
98.759	1.01484	0	0	19.2
98.769	1.01476	0	0	19.2
98.779	1.01469	0	0	19.2
98.789	1.01461	1.00	1.00	19.2
98.799	1.01453	0	0	19.2
98.809	1.01446	0	0	19.2
98.819	1.01438	1.00	1.00	19.2
98.829	1.01430	0	0	19.2
98.839	1.01423	1.00	1.00	19.2
98.850	1.01415	0	0	19.2
98.860	1.01407	0	0	19.2
98.870	1.01400	0	0	19.2
98.880	1.01392	1.00	1.00	19.2
98.890	1.01384	0	0	19.2
98.900	1.01377	0	0	19.2
98.910	1.01369	0	0	19.2

98.920	1.01361	0	0	19.2
98.930	1.01354	0	0	19.2
98.940	1.01346	0	0	19.2
98.951	1.01339	1.00	1.00	19.2
98.961	1.01331	0	0	19.2
98.971	1.01323	1.00	1.00	19.2
98.981	1.01316	0	0	19.2
98.991	1.01308	0	0	19.2
99.001	1.01300	0	0	19.2
99.011	1.01293	0	0	19.2
99.021	1.01285	0	0	19.2
99.031	1.01277	0	0	19.2
99.041	1.01270	1.00	1.00	19.2
99.052	1.01262	0	0	19.2
99.062	1.01255	0	0	19.2
99.072	1.01247	0	0	19.2
99.082	1.01239	0	0	19.2
99.092	1.01232	0	0	19.2
99.102	1.01224	0	0	19.2
99.112	1.01217	1.00	1.00	19.2
99.122	1.01209	1.00	1.00	19.2
99.132	1.01201	0	0	19.2
99.142	1.01194	0	0	19.2
99.153	1.01186	1.00	1.00	19.2
99.163	1.01179	0	0	19.2
99.173	1.01171	0	0	19.2
99.183	1.01163	0	0	19.2
99.193	1.01156	1.00	1.00	19.2
99.203	1.01148	0	0	19.2
99.213	1.01141	0	0	19.2
99.223	1.01133	0	0	19.2
99.233	1.01125	0	0	19.2
99.243	1.01118	0	0	19.2
99.254	1.01110	0	0	19.2
99.264	1.01103	0	0	19.2
99.274	1.01095	0	0	19.2
99.284	1.01088	1.00	1.00	19.2
99.294	1.01080	0	0	19.2
99.304	1.01072	0	0	19.2
99.314	1.01065	0	0	19.2
99.324	1.01057	0	0	19.2
99.334	1.01050	0	0	19.2
99.344	1.01042	0	0	19.2
99.355	1.01035	0	0	19.2
99.365	1.01027	0	0	19.2
99.375	1.01020	0	0	19.2
99.385	1.01012	1.00	1.00	19.2
99.395	1.01004	0	0	19.2
99.405	1.00997	0	0	19.2
99.415	1.00989	0	0	19.2

99.425	1.00982	0	0	19.2
99.435	1.00974	0	0	19.2
99.445	1.00967	0	0	19.2
99.456	1.00959	0	0	19.2
99.466	1.00952	0	0	19.2
99.476	1.00944	1.00	1.00	19.2
99.486	1.00937	0	0	19.2
99.496	1.00929	0	0	19.2
99.506	1.00922	0	0	19.2
99.516	1.00914	0	0	19.2
99.526	1.00906	0	0	19.2
99.536	1.00899	0	0	19.2
99.546	1.00891	0	0	19.2
99.557	1.00884	0	0	19.2
99.567	1.00876	0	0	19.2
99.577	1.00869	0	0	19.2
99.587	1.00861	0	0	19.2
99.597	1.00854	0	0	19.2
99.607	1.00846	0	0	19.2
99.617	1.00839	0	0	19.2
99.627	1.00831	0	0	19.2
99.637	1.00824	1.00	1.00	19.2
99.647	1.00816	0	0	19.2
99.657	1.00809	0	0	19.2
99.668	1.00801	1.00	1.00	19.2
99.678	1.00794	0	0	19.2
99.688	1.00786	0	0	19.2
99.698	1.00779	0	0	19.2
99.708	1.00771	0	0	19.2
99.718	1.00764	0	0	19.2
99.728	1.00756	1.00	1.00	19.2
99.738	1.00749	0	0	19.2
99.748	1.00741	0	0	19.2
99.758	1.00734	0	0	19.2
99.769	1.00726	0	0	19.2
99.779	1.00719	0	0	19.2
99.789	1.00711	0	0	19.2
99.799	1.00704	0	0	19.2
99.809	1.00697	0	0	19.2
99.819	1.00689	0	0	19.2
99.829	1.00682	0	0	19.2
99.839	1.00674	0	0	19.2
99.849	1.00667	0	0	19.2
99.859	1.00659	0	0	19.2
99.870	1.00652	0	0	19.2
99.880	1.00644	0	0	19.2
99.890	1.00637	0	0	19.2
99.900	1.00629	0	0	19.2
99.910	1.00622	1.00	1.00	19.2
99.920	1.00614	0	0	19.2

99.930	1.00607	0	0	19.2
99.940	1.00600	0	0	19.2
99.950	1.00592	0	0	19.2
99.960	1.00585	0	0	19.2
99.971	1.00577	0	0	19.2
99.981	1.00570	1.00	1.00	19.2
99.991	1.00562	0	0	19.2
100.001	1.00555	0	0	19.2

2Theta

Show	Icon	Color	Index	Name	Parent	Scan	Pattern #
Yes			0	PDF 44-0558 (Tune Cell)	Pattern List #3	1.raw #1	PDF 44-0558 (Tune Cell)
Yes			1	PDF 89-8489 (Tune Cell)	Pattern List #3	1.raw #1	PDF 89-8489 (Tune Cell)
Yes			2	PDF 81-2220 (Tune Cell)	Pattern List #3	1.raw #1	PDF 81-2220 (Tune Cell)
Yes			3	PDF 74-2329 (Tune Cell)	Pattern List #3	1.raw #1	PDF 74-2329 (Tune Cell)
Yes			4	PDF 82-0505 (Tune Cell)	Pattern List #3	1.raw #1	PDF 82-0505 (Tune Cell)

Compound Name	Formula	Quality	Y-Scale	I/Ic DB	I/Ic User	S-Q	Added Reference
buckminsterfullerene Carbon	C60	Star (*)	15.7302 %	2.200		28.12 %	
Graphite - theoretical	C	Hypothetical	11.4317 %	2.040		22.04 %	
Carbon	C60	Calculated	9.6063 %	1.090		34.66 %	
Graphite nitrate	C	Calculated	4.6503 %	10.040		1.82 %	
Carbon	C60	Calculated	5.0936 %	1.500		13.36 %	

d x by	Scan WL	Wavelength	System	Space Group	a	b	c	alpha	beta
1.0000	Yes	1.54060	Cubic	F (0)	14.16600				
1.0000	Yes	1.54060	Orthorhombic	Cmmm (65)	4.52500	5.33400	5.92500		
1.0000	Yes	1.54060	Cubic	Fm-3m (225)	14.16000				
1.0000	Yes	1.54060	Rhombo.H.axes	R-3m (166)	2.46000		53.50000		
1.0000	Yes	1.54060	Cubic	Fm-3 (202)	14.26000				

gamma	Z	Volume	Density	Cell Tuned	F (N)
	4	2842.77		Automatic	F29= 32.2(0.0210, 42)
	16	143.01	2.231	Automatic	F30= 421.7(0.0020, 35)
	2	2839.16	0.505	Automatic	F30= 999.9(0.0000, 30)
	24	280.38	1.707	Automatic	F30= 503.2(0.0018, 33)
	4	2899.74	1.650	Automatic	F30= 999.9(0.0000, 30)

Pattern: PDF 44-0558 (Tune Cell)   Radiation: 1.54060   Quality: User modified

**Formula**	C60
**Name**	Carbon
**Name (mineral)**	
**Name (common)**	buckminsterfullerene

**Lattice:**	Cubic	**Mol. weight =**	720.66
**S.G.:**	F (0)	**Volume [CD] =**	2842.77
		**Dx =**	
		**Dm =**	
		**I/Icor =**	2.200

**a =**	14.16600	**alpha =**	
**b =**		**beta =**	
**c =**		**gamma =**	
**a/b =**	1.00000	**Z =**	4
**c/b =**	1.00000		

Color: Dark brown
Sample Source Or Locality: Sample from Terrasimco, Inc
Additional Pattern: To replace 43-995

Primary Reference
Publication: Powder Diffraction
Detail: volume 9, page 93 (1994)
Authors: McCready, D., Alnajjar, M.

**Radiation:**	CuKa1	**Filter:**	M
**Wavelength:**	1.54060	**d-spacing:**	
**SS/FOM:**	32.2 (0.021,42)		

d	2θ	I	h	k	l
8.17874	10.809	27	1	1	1
5.00844	17.694	24	2	2	0
4.27121	20.780	19	3	1	1
4.08937	21.715	4	2	2	2
3.24990	27.422	2	3	3	1
3.16761	28.149	4	4	2	0
2.89162	30.899	3	4	2	2
2.72625	32.825	2	5	1	1
2.50422	35.829	0	4	4	0
2.39449	37.531	0	5	3	1
2.23984	40.230	0	6	2	0
2.16029	41.780	0	5	3	3
2.13560	42.286	0	6	2	2
2.04469	44.263	0	4	4	4
1.98364	45.701	1	7	1	1
1.96447	46.172	0	6	4	0
1.89301	48.023	0	6	4	2
1.84426	49.376	0	7	3	1
1.71788	53.282	0	8	2	0
1.66948	54.955	0	6	6	0
1.63575	56.188	0	7	5	1
1.58381	58.203	0	8	4	0
1.55492	59.392	0	9	1	1
1.54564	59.784	0	8	4	2
1.51010	61.341	0	6	6	4
1.48500	62.493	0	9	3	1
1.31528	71.699	0	10	4	0
1.29317	73.120	0	10	4	2
1.27731	74.180	0	11	1	1

Pattern: PDF 89-8489 (Tune Cell) Radiation: 1.54060 Quality: User modified

**Formula**	C
**Name**	Carbon
**Name (mineral)**	Graphite - theoretical
**Name (common)**	

**Lattice:**	Orthorhombic	**Mol. weight =**	12.01
**S.G.:**	Cmmm (65)	**Volume [CD] =**	143.01
		**Dx =**	
		**Dm =**	
		**I/Icor =**	2.040

a =	4.52500	alpha =	
b =	5.33400	beta =	
c =	5.92500	gamma =	
a/b =	0.84833	Z =	16
c/b =	1.11080		

ICSD Collection Code: 088812
Hypothetical Structure: Structure calculated theoretically
Remark From ICSD/CSD: REM K Phase gra_crbl33_bo
Test From ICSD: No R value given
Test From ICSD: At least one TF missing
Article Title: Possible 3D carbon structures as progressive intermediates in graphite to diamond phase transition

Structure
Publication: J. Solid State Chem.
Detail: volume 148, page 278 (1999)
Authors: Fayos, J.
Primary Reference
Publication: Calculated from ICSD using POWD-12++

**Radiation:**	CuKa1	**Filter:**	Not specified
**Wavelength:**	1.54060	**d-spacing:**	
**SS/FOM:**	421.7 (0.002,35)		

d	2θ	I	h	k	l
5.92500	14.940	19	0	0	1
3.45061	25.798	4	1	1	0
2.96250	30.142	3	0	0	2
2.66700	33.575	3	0	2	0
2.43198	36.932	1	0	2	1
2.24774	40.083	1	1	1	2
2.11364	42.746	1	2	0	1
1.97500	45.912	1	0	0	3
1.79810	50.732	1	2	0	2
1.72531	53.035	0	2	2	0
1.71409	53.409	0	1	1	3
1.65484	55.483	1	1	3	0
1.65651	55.422	1	2	2	1
1.59384	57.802	0	1	3	1
1.58718	58.068	0	0	2	3
1.49090	62.218	0	2	2	2
1.48787	62.359	0	2	0	3
1.48125	62.669	0	0	0	4
1.45142	64.108	0	3	1	0
1.44472	64.442	0	1	3	2
1.40974	66.243	0	3	1	1
1.36114	68.933	0	1	1	4
1.33350	70.571	0	0	4	0
1.30340	72.455	0	3	1	2
1.29934	72.717	0	2	2	3
1.29493	73.005	0	0	2	4
1.26843	74.787	0	1	3	3
1.23928	76.862	0	2	0	4
1.21599	78.614	0	0	4	2
1.18500	81.090	0	0	0	5
1.16956	82.390	0	3	1	3
1.14881	84.215	0	2	4	0
1.13125	85.832	0	4	0	0
1.12780	86.158	0	2	4	1
1.12387	86.534	0	2	2	4
1.12075	86.834	0	1	1	5
1.11118	87.772	0	4	0	1
1.10369	88.523	0	1	3	4

Pattern: PDF 81-2220 (Tune Cell) Radiation: 1.54060 Quality: User modified

Formula C60
Name Carbon
Name (mineral)
Name (common)

Lattice: Cubic
S.G.: Fm-3m (225)

Mol. weight = 720.66
Volume [CD] = 2839.16
Dx =
Dm =
I/Icor = 1.090

a = 14.16000
b =
c =
a/b = 1.00000
c/b = 1.00000

alpha =
beta =
gamma =
Z = 2

ICSD Collection Code: 073661
Temperature Factor: ATF
Remark From ICSD/CSD: REM REF
Remark From ICSD/CSD: REM F Refinement on data taken from Phys. Rev. Lett., 69 2943-2946 (1992)
Test From ICSD: Calcul. formula slightly deviates from input
Test From ICSD: At least one SOF implausible
Temperature Factor: ITF
Remark From ICSD/CSD: REM TEM Mentioned
Article Title: Structure of C60: Partial orientational order in the room-temperature modification of C60

Structure
Publication: Acta Crystallogr., Sec. B: Structural Science
Detail: volume 49, page 832 (1993)
Authors: Buergi, H.- B., Restori, R., Schwarzenbach, D.
Primary Reference
Publication: Calculated from ICSD using POWD-12++

Radiation: CuKa1
Wavelength: 1.54060
SS/FOM: 999.9 (0,30)

Filter: Not specified
d-spacing:

d	2θ	I	h	k	l
8.17528	10.813	16	1	1	1
7.08000	12.492	0	2	0	0
5.00632	17.702	12	2	2	0
4.26940	20.789	8	3	1	1
4.08764	21.724	1	2	2	2
3.54000	25.136	0	4	0	0
3.24853	27.434	1	3	3	1
3.16627	28.161	1	4	2	0
2.89040	30.913	2	4	2	2
2.72509	32.839	1	5	1	1
2.50316	35.845	0	4	4	0
2.39348	37.548	0	5	3	1
2.36000	38.101	0	4	4	2
2.23889	40.248	0	6	2	0
2.15938	41.798	0	5	3	3
2.13470	42.304	0	6	2	2
2.04382	44.283	0	4	4	4
1.98280	45.721	0	7	1	1
1.96364	46.193	0	6	4	0
1.89221	48.044	0	6	4	2
1.84347	49.398	0	7	3	1
1.77000	51.596	0	8	0	0
1.72992	52.883	0	7	3	3
1.71715	53.307	0	8	2	0
1.66877	54.980	0	8	2	2
1.63506	56.213	0	7	5	1
1.62426	56.621	0	6	6	2
1.58314	58.230	0	8	4	0
1.55426	59.419	0	7	5	3
1.54498	59.812	0	8	4	2
1.50946	61.370	0	6	6	4
1.48437	62.522	0	9	3	1
1.44520	64.418	0	8	4	4
1.42313	65.540	0	9	3	3
1.41600	65.912	0	10	0	0
1.38850	67.390	0	8	6	2
1.36890	68.488	0	9	5	1
1.36255	68.852	0	10	2	2
1.32043	71.376	0	9	5	3
1.31472	71.734	0	8	6	4
1.29263	73.156	0	10	4	2
1.27676	74.216	0	7	7	5
1.25158	75.971	0	8	8	0
1.23716	77.017	0	11	3	1
1.23247	77.365	0	8	8	2
1.21421	78.752	0	8	6	6
1.20104	79.787	0	11	3	3
1.19674	80.131	0	10	6	2
1.18000	81.506	0	8	8	4
1.16790	82.533	0	11	5	1
1.16394	82.875	0	12	2	0

1.14853	84.240	0	12	2	2
1.13736	85.262	0	11	5	3
1.11945	86.961	0	12	4	0
1.10910	87.979	0	9	9	1
1.10571	88.319	0	12	4	2
1.09247	89.675	0	10	8	2

Pattern: PDF 74-2329 (Tune Cell) Radiation: 1.54060 Quality: User modified

**Formula**	C
**Name**	Carbon
**Name (mineral)**	Graphite nitrate
**Name (common)**	

**Lattice:**	Rhombo.H.axes	**Mol. weight =**	12.01
**S.G.:**	R-3m (166)	**Volume [CD] =**	280.38
		**Dx =**	
		**Dm =**	
		**I/Icor =**	10.040

**a =**	2.46000	**alpha =**	
**b =**		**beta =**	
**c =**	53.50000	**gamma =**	
**a/b =**	1.00000	**Z =**	24
**c/b =**	21.74797		

ICSD Collection Code: 028418
Test From ICSD: No R value given
Test From ICSD: At least one TF missing
Remark From ICSD/CSD: Other atoms not determined
Article Title: Order-disorder transformations in graphite nitrates

Structure
Publication: Proc. R. Soc. London, Ser. A
Detail: volume 291, page 324 (1966)
Authors: Nixon, D.E., Parry, G.S., Ubbelohde, A.R.
Primary Reference
Publication: Calculated from ICSD using POWD-12++

**Radiation:**	CuKa1	**Filter:**	Not specified
**Wavelength:**	1.54060	**d-spacing:**	
**SS/FOM:**	503.2 (0.0018,33)		

d	2θ	I	h	k	l
17.83333	4.951	8	0	0	3
8.91667	9.912	1	0	0	6
5.94444	14.891	0	0	0	9
4.45833	19.899	0	0	0	12
3.56667	24.945	1	0	0	15
2.97222	30.041	0	0	0	18
2.54762	35.199	0	0	0	21
2.22917	40.431	0	0	0	24
2.12874	42.429	0	1	0	1
2.12370	42.534	0	0	1	2
2.08941	43.267	0	0	1	5
2.05219	44.093	0	1	0	7
2.02991	44.602	0	0	1	8
1.97927	45.808	0	1	0	10
1.98148	45.753	0	0	0	27
1.95142	46.499	0	0	1	11
1.89195	48.051	0	1	0	13
1.86079	48.908	0	0	1	14
1.79673	50.773	0	1	0	16
1.78333	51.182	0	0	0	30
1.76420	51.778	0	0	1	17
1.69894	53.924	0	1	0	19
1.66649	55.062	0	0	1	20
1.62121	56.737	0	0	0	33
1.60246	57.462	0	1	0	22
1.57106	58.721	0	0	1	23
1.50981	61.354	0	1	0	25
1.48005	62.725	0	0	1	26
1.42243	65.577	0	1	0	28
1.39461	67.055	0	0	1	29
1.37179	68.323	0	0	0	39
1.34101	70.118	0	1	0	31
1.31523	71.701	0	0	1	32
1.27381	74.418	0	0	0	42
1.26572	74.975	0	1	0	34
1.24196	76.666	0	0	1	35
1.23000	77.549	0	1	1	0
1.22708	77.768	0	1	1	3
1.21846	78.424	0	1	1	6
1.20449	79.513	0	1	1	9
1.19641	80.158	0	1	0	37
1.18889	80.769	0	0	0	45
1.18570	81.031	0	1	1	12
1.17458	81.962	0	0	1	38
1.16280	82.975	0	1	1	15
1.13653	85.339	0	1	1	18
1.11458	87.436	0	0	0	48
1.11274	87.618	0	0	1	41
1.10766	88.123	0	1	1	21

Pattern: PDF 82-0505 (Tune Cell)   Radiation: 1.54060   Quality: User modified

Formula C60
Name Carbon
Name (mineral)
Name (common)

Lattice: Cubic
S.G.: Fm-3 (202)

Mol. weight = 720.66
Volume [CD] = 2899.74
Dx =
Dm =
I/Icor = 1.500

a = 14.26000
b =
c =
a/b = 1.00000
c/b = 1.00000

alpha =
beta =
gamma =
Z = 4

ICSD Collection Code: 074523
Temperature Factor: ITF
Article Title: Disorder and the molecular packing of C60 buckminsterfullerene:a direct electron-crystallographic analysis

Structure
Publication: Acta Crystallogr., Sec. A: Found. Crystallogr.
Detail: volume 50, page 344 (1994)
Authors: Dorset, D.L., McCourt, M.P.
Primary Reference
Publication: Calculated from ICSD using POWD-12++

Radiation: CuKa1
Wavelength: 1.54060
SS/FOM: 999.9 (0,30)

Filter: Not specified
d-spacing:

d	2θ	I	h	k	l
8.23302	10.737	8	1	1	1
7.13000	12.404	0	2	0	0
5.04167	17.577	9	2	2	0
4.29955	20.641	5	3	1	1
4.11651	21.570	1	2	2	2
3.56500	24.957	0	4	0	0
3.27147	27.237	0	3	3	1
3.18863	27.959	1	0	2	4
2.91081	30.690	1	4	2	2
2.74434	32.602	1	3	3	3
2.52084	35.585	0	4	4	0
2.41038	37.275	0	1	3	5
2.37667	37.823	0	6	0	0
2.25470	39.954	0	0	2	6
2.17463	41.491	0	5	3	3
2.14978	41.994	0	6	2	2
2.05825	43.956	0	4	4	4
1.99680	45.383	0	7	1	1
1.97751	45.851	0	6	4	0
1.90557	47.686	0	2	4	6
1.85649	49.029	0	1	3	7
1.78250	51.208	0	8	0	0
1.74214	52.483	0	7	3	3
1.72928	52.904	0	0	2	8
1.68056	54.563	0	8	2	2
1.64660	55.785	0	7	5	1
1.63573	56.188	0	6	6	2
1.59432	57.783	0	0	4	8
1.56524	58.961	0	7	5	3
1.55589	59.351	0	8	4	2
1.52012	60.893	0	6	6	4
1.49485	62.035	0	9	3	1
1.45541	63.912	0	8	4	4
1.43318	65.024	0	7	7	1
1.42600	65.392	0	8	6	0
1.39831	66.855	0	8	6	2
1.37857	67.941	0	9	5	1
1.37217	68.302	0	10	2	2
1.32975	70.800	0	3	5	9
1.32401	71.154	0	0	4	10
1.30175	72.561	0	10	4	2
1.28578	73.610	0	11	1	1
1.26042	75.345	0	8	8	0
1.24590	76.379	0	1	3	11
1.24117	76.723	0	10	4	4
1.22278	78.094	0	10	6	0
1.20952	79.117	0	11	3	3
1.20519	79.457	0	2	6	10
1.18833	80.815	0	8	8	4
1.17615	81.830	0	11	5	1
1.17216	82.167	0	0	2	12

1.15664	83.515	0	12	2	2
1.14539	84.524	0	3	5	11
1.12735	86.201	0	12	4	0
1.11693	87.206	0	9	9	1
1.11352	87.541	0	8	8	6
1.10018	88.879	0	10	8	2
1.09049	89.882	0	11	5	5
