# Supplementary material for: Pure and doped carbon quantum dots as fluorescent probes for the detection of phenol compounds and antibiotics in aquariums
Source: Sci Rep. 2023 Aug 8;13:12863. doi: 10.1038/s41598-023-39490-y (PMC10409781; doi:10.1038/s41598-023-39490-y)
Supplement: Supplementary file 2 — Supplementary Information 2. [file 41598_2023_39490_MOESM2_ESM.pdf]

Commander Sample ID (Coupled TwoTheta/Theta)

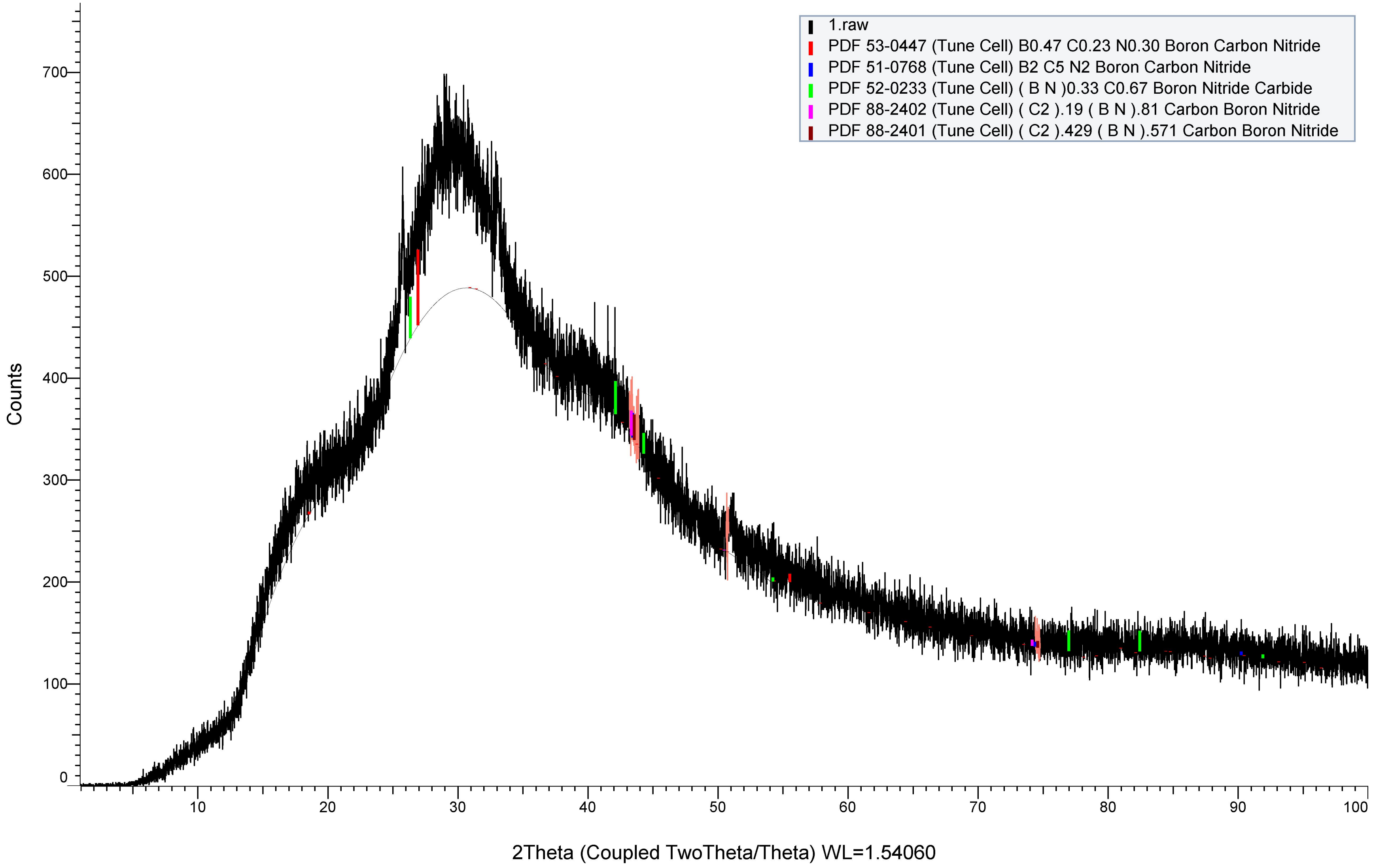

1.raw #1

X	d (Å)	Y (counts)	dY	Time (s)
0.998	88.43782	1.00	1.00	19.2
1.008	87.55197	1.00	1.00	19.2
1.018	86.68368	0	0	19.2
1.028	85.83245	0	0	19.2
1.039	84.99778	0	0	19.2
1.049	84.17918	1.00	1.00	19.2
1.059	83.37620	1.00	1.00	19.2
1.069	82.58840	0	0	19.2
1.079	81.81534	0	0	19.2
1.089	81.05662	0	0	19.2
1.099	80.31185	0	0	19.2
1.109	79.58064	0	0	19.2
1.119	78.86263	0	0	19.2
1.129	78.15745	0	0	19.2
1.140	77.46478	0	0	19.2
1.150	76.78427	0	0	19.2
1.160	76.11562	0	0	19.2
1.170	75.45852	0	0	19.2
1.180	74.81266	0	0	19.2
1.190	74.17776	1.00	1.00	19.2
1.200	73.55356	2.00	1.41	19.2
1.210	72.93977	0	0	19.2
1.220	72.33614	0	0	19.2
1.230	71.74241	1.00	1.00	19.2
1.240	71.15836	1.00	1.00	19.2
1.251	70.58374	0	0	19.2
1.261	70.01833	0	0	19.2
1.271	69.46190	1.00	1.00	19.2
1.281	68.91425	1.00	1.00	19.2
1.291	68.37517	0	0	19.2
1.301	67.84445	0	0	19.2
1.311	67.32191	1.00	1.00	19.2
1.321	66.80736	0	0	19.2
1.331	66.30062	1.00	1.00	19.2
1.341	65.80151	1.00	1.00	19.2
1.352	65.30985	0	0	19.2
1.362	64.82549	0	0	19.2
1.372	64.34826	0	0	19.2
1.382	63.87801	1.00	1.00	19.2
1.392	63.41457	0	0	19.2
1.402	62.95782	0	0	19.2
1.412	62.50760	0	0	19.2
1.422	62.06377	1.00	1.00	19.2
1.432	61.62621	1.00	1.00	19.2
1.442	61.19476	1.00	1.00	19.2
1.453	60.76932	1.00	1.00	19.2

1.463	60.34976	0	0	19.2
1.473	59.93594	0	0	19.2
1.483	59.52777	0	0	19.2
1.493	59.12512	0	0	19.2
1.503	58.72787	0	0	19.2
1.513	58.33594	0	0	19.2
1.523	57.94919	1.00	1.00	19.2
1.533	57.56755	0	0	19.2
1.543	57.19089	1.00	1.00	19.2
1.554	56.81914	1.00	1.00	19.2
1.564	56.45218	1.00	1.00	19.2
1.574	56.08994	0	0	19.2
1.584	55.73232	1.00	1.00	19.2
1.594	55.37922	0	0	19.2
1.604	55.03058	0	0	19.2
1.614	54.68630	0	0	19.2
1.624	54.34629	0	0	19.2
1.634	54.01050	0	0	19.2
1.644	53.67882	2.00	1.41	19.2
1.655	53.35119	0	0	19.2
1.665	53.02754	0	0	19.2
1.675	52.70780	1.00	1.00	19.2
1.685	52.39188	0	0	19.2
1.695	52.07974	0	0	19.2
1.705	51.77129	0	0	19.2
1.715	51.46647	0	0	19.2
1.725	51.16522	0	0	19.2
1.735	50.86748	2.00	1.41	19.2
1.745	50.57318	0	0	19.2
1.756	50.28227	0	0	19.2
1.766	49.99468	0	0	19.2
1.776	49.71037	1.00	1.00	19.2
1.786	49.42928	0	0	19.2
1.796	49.15134	1.00	1.00	19.2
1.806	48.87652	0	0	19.2
1.816	48.60475	0	0	19.2
1.826	48.33598	0	0	19.2
1.836	48.07018	1.00	1.00	19.2
1.846	47.80728	0	0	19.2
1.857	47.54724	1.00	1.00	19.2
1.867	47.29001	0	0	19.2
1.877	47.03556	1.00	1.00	19.2
1.887	46.78382	0	0	19.2
1.897	46.53477	0	0	19.2
1.907	46.28836	0	0	19.2
1.917	46.04454	0	0	19.2
1.927	45.80328	0	0	19.2
1.937	45.56453	0	0	19.2
1.947	45.32826	0	0	19.2
1.958	45.09443	0	0	19.2

1.968	44.86300	1.00	1.00	19.2
1.978	44.63393	0	0	19.2
1.988	44.40719	0	0	19.2
1.998	44.18274	0	0	19.2
2.008	43.96055	0	0	19.2
2.018	43.74058	0	0	19.2
2.028	43.52281	0	0	19.2
2.038	43.30719	0	0	19.2
2.048	43.09370	0	0	19.2
2.059	42.88230	0	0	19.2
2.069	42.67297	0	0	19.2
2.079	42.46567	0	0	19.2
2.089	42.26038	0	0	19.2
2.099	42.05706	0	0	19.2
2.109	41.85569	0	0	19.2
2.119	41.65624	0	0	19.2
2.129	41.45868	1.00	1.00	19.2
2.139	41.26299	2.00	1.41	19.2
2.149	41.06913	0	0	19.2
2.160	40.87709	0	0	19.2
2.170	40.68684	0	0	19.2
2.180	40.49835	1.00	1.00	19.2
2.190	40.31159	1.00	1.00	19.2
2.200	40.12656	0	0	19.2
2.210	39.94321	0	0	19.2
2.220	39.76154	2.00	1.41	19.2
2.230	39.58150	0	0	19.2
2.240	39.40309	0	0	19.2
2.250	39.22629	1.00	1.00	19.2
2.261	39.05106	1.00	1.00	19.2
2.271	38.87739	1.00	1.00	19.2
2.281	38.70526	0	0	19.2
2.291	38.53465	0	0	19.2
2.301	38.36553	0	0	19.2
2.311	38.19790	1.00	1.00	19.2
2.321	38.03172	0	0	19.2
2.331	37.86698	0	0	19.2
2.341	37.70367	1.00	1.00	19.2
2.351	37.54175	0	0	19.2
2.362	37.38122	1.00	1.00	19.2
2.372	37.22206	0	0	19.2
2.382	37.06425	2.00	1.41	19.2
2.392	36.90777	0	0	19.2
2.402	36.75261	0	0	19.2
2.412	36.59875	0	0	19.2
2.422	36.44617	0	0	19.2
2.432	36.29486	0	0	19.2
2.442	36.14479	0	0	19.2
2.452	35.99597	2.00	1.41	19.2
2.463	35.84836	0	0	19.2

2.473	35.70197	0	0	19.2
2.483	35.55676	1.00	1.00	19.2
2.493	35.41273	0	0	19.2
2.503	35.26986	0	0	19.2
2.513	35.12814	1.00	1.00	19.2
2.523	34.98755	0	0	19.2
2.533	34.84809	1.00	1.00	19.2
2.543	34.70973	2.00	1.41	19.2
2.553	34.57247	0	0	19.2
2.563	34.43629	0	0	19.2
2.574	34.30118	0	0	19.2
2.584	34.16713	0	0	19.2
2.594	34.03411	1.00	1.00	19.2
2.604	33.90214	0	0	19.2
2.614	33.77118	0	0	19.2
2.624	33.64122	1.00	1.00	19.2
2.634	33.51227	1.00	1.00	19.2
2.644	33.38430	0	0	19.2
2.654	33.25730	2.00	1.41	19.2
2.664	33.13127	1.00	1.00	19.2
2.675	33.00619	0	0	19.2
2.685	32.88205	0	0	19.2
2.695	32.75884	0	0	19.2
2.705	32.63655	0	0	19.2
2.715	32.51517	0	0	19.2
2.725	32.39469	1.00	1.00	19.2
2.735	32.27510	1.00	1.00	19.2
2.745	32.15639	0	0	19.2
2.755	32.03855	1.00	1.00	19.2
2.765	31.92158	1.00	1.00	19.2
2.776	31.80545	0	0	19.2
2.786	31.69016	0	0	19.2
2.796	31.57571	1.00	1.00	19.2
2.806	31.46208	0	0	19.2
2.816	31.34927	0	0	19.2
2.826	31.23726	1.00	1.00	19.2
2.836	31.12605	0	0	19.2
2.846	31.01563	1.00	1.00	19.2
2.856	30.90599	0	0	19.2
2.866	30.79713	0	0	19.2
2.877	30.68903	0	0	19.2
2.887	30.58168	0	0	19.2
2.897	30.47508	0	0	19.2
2.907	30.36923	0	0	19.2
2.917	30.26411	1.00	1.00	19.2
2.927	30.15971	0	0	19.2
2.937	30.05603	2.00	1.41	19.2
2.947	29.95306	0	0	19.2
2.957	29.85079	0	0	19.2
2.967	29.74923	0	0	19.2

2.978	29.64835	0	0	19.2
2.988	29.54815	1.00	1.00	19.2
2.998	29.44862	0	0	19.2
3.008	29.34977	0	0	19.2
3.018	29.25158	1.00	1.00	19.2
3.028	29.15404	2.00	1.41	19.2
3.038	29.05715	0	0	19.2
3.048	28.96090	1.00	1.00	19.2
3.058	28.86529	0	0	19.2
3.068	28.77031	2.00	1.41	19.2
3.079	28.67595	0	0	19.2
3.089	28.58221	0	0	19.2
3.099	28.48908	0	0	19.2
3.109	28.39655	1.00	1.00	19.2
3.119	28.30463	0	0	19.2
3.129	28.21330	0	0	19.2
3.139	28.12255	2.00	1.41	19.2
3.149	28.03239	0	0	19.2
3.159	27.94280	1.00	1.00	19.2
3.169	27.85379	0	0	19.2
3.180	27.76534	0	0	19.2
3.190	27.67745	0	0	19.2
3.200	27.59011	0	0	19.2
3.210	27.50333	0	0	19.2
3.220	27.41709	0	0	19.2
3.230	27.33139	0	0	19.2
3.240	27.24622	0	0	19.2
3.250	27.16158	1.00	1.00	19.2
3.260	27.07747	1.00	1.00	19.2
3.270	26.99387	1.00	1.00	19.2
3.281	26.91080	2.00	1.41	19.2
3.291	26.82823	0	0	19.2
3.301	26.74616	0	0	19.2
3.311	26.66460	1.00	1.00	19.2
3.321	26.58353	0	0	19.2
3.331	26.50296	0	0	19.2
3.341	26.42287	1.00	1.00	19.2
3.351	26.34327	0	0	19.2
3.361	26.26414	0	0	19.2
3.371	26.18549	1.00	1.00	19.2
3.382	26.10731	0	0	19.2
3.392	26.02959	0	0	19.2
3.402	25.95233	1.00	1.00	19.2
3.412	25.87554	0	0	19.2
3.422	25.79919	2.00	1.41	19.2
3.432	25.72330	0	0	19.2
3.442	25.64785	1.00	1.00	19.2
3.452	25.57284	0	0	19.2
3.462	25.49827	0	0	19.2
3.472	25.42413	3.00	1.73	19.2

3.483	25.35042	0	0	19.2
3.493	25.27714	0	0	19.2
3.503	25.20429	0	0	19.2
3.513	25.13185	0	0	19.2
3.523	25.05982	0	0	19.2
3.533	24.98821	0	0	19.2
3.543	24.91701	0	0	19.2
3.553	24.84621	0	0	19.2
3.563	24.77581	0	0	19.2
3.573	24.70581	1.00	1.00	19.2
3.584	24.63621	0	0	19.2
3.594	24.56700	0	0	19.2
3.604	24.49817	0	0	19.2
3.614	24.42973	0	0	19.2
3.624	24.36167	0	0	19.2
3.634	24.29399	0	0	19.2
3.644	24.22669	0	0	19.2
3.654	24.15975	0	0	19.2
3.664	24.09319	0	0	19.2
3.674	24.02699	0	0	19.2
3.685	23.96115	0	0	19.2
3.695	23.89568	1.00	1.00	19.2
3.705	23.83056	3.00	1.73	19.2
3.715	23.76580	0	0	19.2
3.725	23.70138	0	0	19.2
3.735	23.63732	0	0	19.2
3.745	23.57360	0	0	19.2
3.755	23.51022	0	0	19.2
3.765	23.44719	0	0	19.2
3.775	23.38449	1.00	1.00	19.2
3.786	23.32213	0	0	19.2
3.796	23.26009	0	0	19.2
3.806	23.19839	2.00	1.41	19.2
3.816	23.13701	1.00	1.00	19.2
3.826	23.07596	0	0	19.2
3.836	23.01523	1.00	1.00	19.2
3.846	22.95482	0	0	19.2
3.856	22.89472	0	0	19.2
3.866	22.83494	1.00	1.00	19.2
3.876	22.77547	1.00	1.00	19.2
3.886	22.71631	0	0	19.2
3.897	22.65746	3.00	1.73	19.2
3.907	22.59891	0	0	19.2
3.917	22.54066	0	0	19.2
3.927	22.48271	1.00	1.00	19.2
3.937	22.42506	0	0	19.2
3.947	22.36771	1.00	1.00	19.2
3.957	22.31065	2.00	1.41	19.2
3.967	22.25387	0	0	19.2
3.977	22.19739	0	0	19.2

3.987	22.14119	0	0	19.2
3.998	22.08528	0	0	19.2
4.008	22.02965	3.00	1.73	19.2
4.018	21.97430	1.00	1.00	19.2
4.028	21.91922	2.00	1.41	19.2
4.038	21.86442	0	0	19.2
4.048	21.80990	0	0	19.2
4.058	21.75564	2.00	1.41	19.2
4.068	21.70166	0	0	19.2
4.078	21.64794	1.00	1.00	19.2
4.088	21.59449	2.00	1.41	19.2
4.099	21.54130	2.00	1.41	19.2
4.109	21.48838	0	0	19.2
4.119	21.43571	1.00	1.00	19.2
4.129	21.38330	0	0	19.2
4.139	21.33115	0	0	19.2
4.149	21.27925	0	0	19.2
4.159	21.22760	0	0	19.2
4.169	21.17620	0	0	19.2
4.179	21.12506	0	0	19.2
4.189	21.07415	1.00	1.00	19.2
4.200	21.02350	2.00	1.41	19.2
4.210	20.97308	1.00	1.00	19.2
4.220	20.92291	0	0	19.2
4.230	20.87298	1.00	1.00	19.2
4.240	20.82328	1.00	1.00	19.2
4.250	20.77382	1.00	1.00	19.2
4.260	20.72460	1.00	1.00	19.2
4.270	20.67561	0	0	19.2
4.280	20.62685	0	0	19.2
4.290	20.57832	0	0	19.2
4.301	20.53001	1.00	1.00	19.2
4.311	20.48194	1.00	1.00	19.2
4.321	20.43409	1.00	1.00	19.2
4.331	20.38646	0	0	19.2
4.341	20.33905	0	0	19.2
4.351	20.29187	1.00	1.00	19.2
4.361	20.24490	0	0	19.2
4.371	20.19815	3.00	1.73	19.2
4.381	20.15161	3.00	1.73	19.2
4.391	20.10529	0	0	19.2
4.402	20.05918	0	0	19.2
4.412	20.01329	1.00	1.00	19.2
4.422	19.96760	0	0	19.2
4.432	19.92212	0	0	19.2
4.442	19.87685	1.00	1.00	19.2
4.452	19.83178	0	0	19.2
4.462	19.78692	1.00	1.00	19.2
4.472	19.74226	1.00	1.00	19.2
4.482	19.69780	0	0	19.2

4.492	19.65354	1.00	1.00	19.2
4.503	19.60948	0	0	19.2
4.513	19.56562	0	0	19.2
4.523	19.52195	1.00	1.00	19.2
4.533	19.47848	2.00	1.41	19.2
4.543	19.43520	0	0	19.2
4.553	19.39211	2.00	1.41	19.2
4.563	19.34921	2.00	1.41	19.2
4.573	19.30651	0	0	19.2
4.583	19.26399	1.00	1.00	19.2
4.593	19.22166	0	0	19.2
4.604	19.17951	0	0	19.2
4.614	19.13755	1.00	1.00	19.2
4.624	19.09577	2.00	1.41	19.2
4.634	19.05418	1.00	1.00	19.2
4.644	19.01276	1.00	1.00	19.2
4.654	18.97153	0	0	19.2
4.664	18.93047	0	0	19.2
4.674	18.88959	3.00	1.73	19.2
4.684	18.84889	1.00	1.00	19.2
4.694	18.80836	2.00	1.41	19.2
4.705	18.76801	2.00	1.41	19.2
4.715	18.72783	0	0	19.2
4.725	18.68782	2.00	1.41	19.2
4.735	18.64798	0	0	19.2
4.745	18.60831	4.00	2.00	19.2
4.755	18.56881	1.00	1.00	19.2
4.765	18.52948	1.00	1.00	19.2
4.775	18.49031	0	0	19.2
4.785	18.45131	3.00	1.73	19.2
4.795	18.41248	2.00	1.41	19.2
4.806	18.37381	2.00	1.41	19.2
4.816	18.33529	2.00	1.41	19.2
4.826	18.29695	1.00	1.00	19.2
4.836	18.25876	0	0	19.2
4.846	18.22073	3.00	1.73	19.2
4.856	18.18285	2.00	1.41	19.2
4.866	18.14514	1.00	1.00	19.2
4.876	18.10758	4.00	2.00	19.2
4.886	18.07018	1.00	1.00	19.2
4.896	18.03293	3.00	1.73	19.2
4.907	17.99584	1.00	1.00	19.2
4.917	17.95889	3.00	1.73	19.2
4.927	17.92210	4.00	2.00	19.2
4.937	17.88546	1.00	1.00	19.2
4.947	17.84897	3.00	1.73	19.2
4.957	17.81263	1.00	1.00	19.2
4.967	17.77643	1.00	1.00	19.2
4.977	17.74039	1.00	1.00	19.2
4.987	17.70449	4.00	2.00	19.2

4.997	17.66873	2.00	1.41	19.2
5.008	17.63312	4.00	2.00	19.2
5.018	17.59765	1.00	1.00	19.2
5.028	17.56232	3.00	1.73	19.2
5.038	17.52714	1.00	1.00	19.2
5.048	17.49209	3.00	1.73	19.2
5.058	17.45719	3.00	1.73	19.2
5.068	17.42243	4.00	2.00	19.2
5.078	17.38780	2.00	1.41	19.2
5.088	17.35331	0	0	19.2
5.098	17.31896	3.00	1.73	19.2
5.109	17.28474	2.00	1.41	19.2
5.119	17.25066	2.00	1.41	19.2
5.129	17.21672	0	0	19.2
5.139	17.18290	1.00	1.00	19.2
5.149	17.14922	4.00	2.00	19.2
5.159	17.11567	2.00	1.41	19.2
5.169	17.08226	2.00	1.41	19.2
5.179	17.04897	4.00	2.00	19.2
5.189	17.01581	0	0	19.2
5.199	16.98278	4.00	2.00	19.2
5.209	16.94988	5.00	2.24	19.2
5.220	16.91711	2.00	1.41	19.2
5.230	16.88446	4.00	2.00	19.2
5.240	16.85194	2.00	1.41	19.2
5.250	16.81955	5.00	2.24	19.2
5.260	16.78728	4.00	2.00	19.2
5.270	16.75513	2.00	1.41	19.2
5.280	16.72310	0	0	19.2
5.290	16.69120	3.00	1.73	19.2
5.300	16.65942	4.00	2.00	19.2
5.310	16.62776	2.00	1.41	19.2
5.321	16.59622	5.00	2.24	19.2
5.331	16.56480	0	0	19.2
5.341	16.53350	5.00	2.24	19.2
5.351	16.50232	3.00	1.73	19.2
5.361	16.47125	4.00	2.00	19.2
5.371	16.44031	4.00	2.00	19.2
5.381	16.40947	3.00	1.73	19.2
5.391	16.37876	2.00	1.41	19.2
5.401	16.34816	1.00	1.00	19.2
5.411	16.31767	4.00	2.00	19.2
5.422	16.28729	4.00	2.00	19.2
5.432	16.25703	4.00	2.00	19.2
5.442	16.22689	3.00	1.73	19.2
5.452	16.19685	7.00	2.65	19.2
5.462	16.16692	3.00	1.73	19.2
5.472	16.13711	2.00	1.41	19.2
5.482	16.10740	2.00	1.41	19.2
5.492	16.07781	3.00	1.73	19.2

5.502	16.04832	4.00	2.00	19.2
5.512	16.01894	3.00	1.73	19.2
5.523	15.98967	4.00	2.00	19.2
5.533	15.96051	3.00	1.73	19.2
5.543	15.93145	1.00	1.00	19.2
5.553	15.90249	7.00	2.65	19.2
5.563	15.87365	5.00	2.24	19.2
5.573	15.84490	6.00	2.45	19.2
5.583	15.81627	7.00	2.65	19.2
5.593	15.78773	7.00	2.65	19.2
5.603	15.75930	1.00	1.00	19.2
5.613	15.73097	3.00	1.73	19.2
5.624	15.70274	2.00	1.41	19.2
5.634	15.67461	3.00	1.73	19.2
5.644	15.64659	5.00	2.24	19.2
5.654	15.61866	4.00	2.00	19.2
5.664	15.59083	4.00	2.00	19.2
5.674	15.56311	3.00	1.73	19.2
5.684	15.53548	6.00	2.45	19.2
5.694	15.50795	5.00	2.24	19.2
5.704	15.48051	6.00	2.45	19.2
5.714	15.45318	9.00	3.00	19.2
5.725	15.42594	5.00	2.24	19.2
5.735	15.39879	2.00	1.41	19.2
5.745	15.37174	3.00	1.73	19.2
5.755	15.34479	6.00	2.45	19.2
5.765	15.31793	5.00	2.24	19.2
5.775	15.29117	1.00	1.00	19.2
5.785	15.26450	2.00	1.41	19.2
5.795	15.23792	16.0	4.00	19.2
5.805	15.21143	2.00	1.41	19.2
5.815	15.18504	2.00	1.41	19.2
5.826	15.15874	0	0	19.2
5.836	15.13252	4.00	2.00	19.2
5.846	15.10640	8.00	2.83	19.2
5.856	15.08037	5.00	2.24	19.2
5.866	15.05443	5.00	2.24	19.2
5.876	15.02858	2.00	1.41	19.2
5.886	15.00282	8.00	2.83	19.2
5.896	14.97714	7.00	2.65	19.2
5.906	14.95156	4.00	2.00	19.2
5.916	14.92606	2.00	1.41	19.2
5.927	14.90064	2.00	1.41	19.2
5.937	14.87532	8.00	2.83	19.2
5.947	14.85008	8.00	2.83	19.2
5.957	14.82492	6.00	2.45	19.2
5.967	14.79986	6.00	2.45	19.2
5.977	14.77487	7.00	2.65	19.2
5.987	14.74997	7.00	2.65	19.2
5.997	14.72516	5.00	2.24	19.2

6.007	14.70042	2.00	1.41	19.2
6.017	14.67577	5.00	2.24	19.2
6.028	14.65121	3.00	1.73	19.2
6.038	14.62672	12.0	3.46	19.2
6.048	14.60232	4.00	2.00	19.2
6.058	14.57800	6.00	2.45	19.2
6.068	14.55376	8.00	2.83	19.2
6.078	14.52960	8.00	2.83	19.2
6.088	14.50552	9.00	3.00	19.2
6.098	14.48152	2.00	1.41	19.2
6.108	14.45760	5.00	2.24	19.2
6.118	14.43376	7.00	2.65	19.2
6.129	14.40999	4.00	2.00	19.2
6.139	14.38631	3.00	1.73	19.2
6.149	14.36270	5.00	2.24	19.2
6.159	14.33917	8.00	2.83	19.2
6.169	14.31572	4.00	2.00	19.2
6.179	14.29234	10.0	3.16	19.2
6.189	14.26905	8.00	2.83	19.2
6.199	14.24582	6.00	2.45	19.2
6.209	14.22267	9.00	3.00	19.2
6.219	14.19960	10.0	3.16	19.2
6.230	14.17660	10.0	3.16	19.2
6.240	14.15368	13.0	3.61	19.2
6.250	14.13083	5.00	2.24	19.2
6.260	14.10806	1.00	1.00	19.2
6.270	14.08535	2.00	1.41	19.2
6.280	14.06273	7.00	2.65	19.2
6.290	14.04017	5.00	2.24	19.2
6.300	14.01769	10.0	3.16	19.2
6.310	13.99527	4.00	2.00	19.2
6.320	13.97293	13.0	3.61	19.2
6.331	13.95067	5.00	2.24	19.2
6.341	13.92847	5.00	2.24	19.2
6.351	13.90634	8.00	2.83	19.2
6.361	13.88428	6.00	2.45	19.2
6.371	13.86230	14.0	3.74	19.2
6.381	13.84038	11.0	3.32	19.2
6.391	13.81853	14.0	3.74	19.2
6.401	13.79675	8.00	2.83	19.2
6.411	13.77504	9.00	3.00	19.2
6.421	13.75340	9.00	3.00	19.2
6.431	13.73183	5.00	2.24	19.2
6.442	13.71032	10.0	3.16	19.2
6.452	13.68888	7.00	2.65	19.2
6.462	13.66751	4.00	2.00	19.2
6.472	13.64620	14.0	3.74	19.2
6.482	13.62497	10.0	3.16	19.2
6.492	13.60379	8.00	2.83	19.2
6.502	13.58269	9.00	3.00	19.2

6.512	13.56164	9.00	3.00	19.2
6.522	13.54067	10.0	3.16	19.2
6.532	13.51976	5.00	2.24	19.2
6.543	13.49891	7.00	2.65	19.2
6.553	13.47813	15.0	3.87	19.2
6.563	13.45741	18.0	4.24	19.2
6.573	13.43676	8.00	2.83	19.2
6.583	13.41616	9.00	3.00	19.2
6.593	13.39564	6.00	2.45	19.2
6.603	13.37517	6.00	2.45	19.2
6.613	13.35477	13.0	3.61	19.2
6.623	13.33443	9.00	3.00	19.2
6.633	13.31415	10.0	3.16	19.2
6.644	13.29393	14.0	3.74	19.2
6.654	13.27378	4.00	2.00	19.2
6.664	13.25368	17.0	4.12	19.2
6.674	13.23365	23.0	4.80	19.2
6.684	13.21368	8.00	2.83	19.2
6.694	13.19376	8.00	2.83	19.2
6.704	13.17391	14.0	3.74	19.2
6.714	13.15412	15.0	3.87	19.2
6.724	13.13438	8.00	2.83	19.2
6.734	13.11471	7.00	2.65	19.2
6.745	13.09510	18.0	4.24	19.2
6.755	13.07554	8.00	2.83	19.2
6.765	13.05604	12.0	3.46	19.2
6.775	13.03660	16.0	4.00	19.2
6.785	13.01722	4.00	2.00	19.2
6.795	12.99789	16.0	4.00	19.2
6.805	12.97863	8.00	2.83	19.2
6.815	12.95942	13.0	3.61	19.2
6.825	12.94026	14.0	3.74	19.2
6.835	12.92117	7.00	2.65	19.2
6.846	12.90213	9.00	3.00	19.2
6.856	12.88314	14.0	3.74	19.2
6.866	12.86422	5.00	2.24	19.2
6.876	12.84534	19.0	4.36	19.2
6.886	12.82653	15.0	3.87	19.2
6.896	12.80777	11.0	3.32	19.2
6.906	12.78906	11.0	3.32	19.2
6.916	12.77041	4.00	2.00	19.2
6.926	12.75181	12.0	3.46	19.2
6.936	12.73327	12.0	3.46	19.2
6.947	12.71478	8.00	2.83	19.2
6.957	12.69634	17.0	4.12	19.2
6.967	12.67796	10.0	3.16	19.2
6.977	12.65963	22.0	4.69	19.2
6.987	12.64135	17.0	4.12	19.2
6.997	12.62313	13.0	3.61	19.2
7.007	12.60496	13.0	3.61	19.2

7.017	12.58684	10.0	3.16	19.2
7.027	12.56877	8.00	2.83	19.2
7.037	12.55076	22.0	4.69	19.2
7.048	12.53280	7.00	2.65	19.2
7.058	12.51489	7.00	2.65	19.2
7.068	12.49703	12.0	3.46	19.2
7.078	12.47922	10.0	3.16	19.2
7.088	12.46146	12.0	3.46	19.2
7.098	12.44375	17.0	4.12	19.2
7.108	12.42609	13.0	3.61	19.2
7.118	12.40849	11.0	3.32	19.2
7.128	12.39093	16.0	4.00	19.2
7.138	12.37342	11.0	3.32	19.2
7.149	12.35596	9.00	3.00	19.2
7.159	12.33855	10.0	3.16	19.2
7.169	12.32119	8.00	2.83	19.2
7.179	12.30388	14.0	3.74	19.2
7.189	12.28662	9.00	3.00	19.2
7.199	12.26941	11.0	3.32	19.2
7.209	12.25224	10.0	3.16	19.2
7.219	12.23512	11.0	3.32	19.2
7.229	12.21805	16.0	4.00	19.2
7.239	12.20103	9.00	3.00	19.2
7.250	12.18406	11.0	3.32	19.2
7.260	12.16713	13.0	3.61	19.2
7.270	12.15025	6.00	2.45	19.2
7.280	12.13342	10.0	3.16	19.2
7.290	12.11663	13.0	3.61	19.2
7.300	12.09989	14.0	3.74	19.2
7.310	12.08320	6.00	2.45	19.2
7.320	12.06655	12.0	3.46	19.2
7.330	12.04995	13.0	3.61	19.2
7.340	12.03339	22.0	4.69	19.2
7.351	12.01688	8.00	2.83	19.2
7.361	12.00042	14.0	3.74	19.2
7.371	11.98400	13.0	3.61	19.2
7.381	11.96762	10.0	3.16	19.2
7.391	11.95129	8.00	2.83	19.2
7.401	11.93500	15.0	3.87	19.2
7.411	11.91876	8.00	2.83	19.2
7.421	11.90257	6.00	2.45	19.2
7.431	11.88641	19.0	4.36	19.2
7.441	11.87030	15.0	3.87	19.2
7.452	11.85424	16.0	4.00	19.2
7.462	11.83822	11.0	3.32	19.2
7.472	11.82224	21.0	4.58	19.2
7.482	11.80630	4.00	2.00	19.2
7.492	11.79041	11.0	3.32	19.2
7.502	11.77456	16.0	4.00	19.2
7.512	11.75875	7.00	2.65	19.2

7.522	11.74299	19.0	4.36	19.2
7.532	11.72727	25.0	5.00	19.2
7.542	11.71159	23.0	4.80	19.2
7.553	11.69595	20.0	4.47	19.2
7.563	11.68035	10.0	3.16	19.2
7.573	11.66480	12.0	3.46	19.2
7.583	11.64928	24.0	4.90	19.2
7.593	11.63381	12.0	3.46	19.2
7.603	11.61838	20.0	4.47	19.2
7.613	11.60299	25.0	5.00	19.2
7.623	11.58764	18.0	4.24	19.2
7.633	11.57233	20.0	4.47	19.2
7.643	11.55707	16.0	4.00	19.2
7.654	11.54184	24.0	4.90	19.2
7.664	11.52665	15.0	3.87	19.2
7.674	11.51150	18.0	4.24	19.2
7.684	11.49640	15.0	3.87	19.2
7.694	11.48133	23.0	4.80	19.2
7.704	11.46630	27.0	5.20	19.2
7.714	11.45131	13.0	3.61	19.2
7.724	11.43636	11.0	3.32	19.2
7.734	11.42145	26.0	5.10	19.2
7.744	11.40658	12.0	3.46	19.2
7.754	11.39175	12.0	3.46	19.2
7.765	11.37695	18.0	4.24	19.2
7.775	11.36220	18.0	4.24	19.2
7.785	11.34748	22.0	4.69	19.2
7.795	11.33280	11.0	3.32	19.2
7.805	11.31816	18.0	4.24	19.2
7.815	11.30355	18.0	4.24	19.2
7.825	11.28899	15.0	3.87	19.2
7.835	11.27446	23.0	4.80	19.2
7.845	11.25997	18.0	4.24	19.2
7.855	11.24552	25.0	5.00	19.2
7.866	11.23110	17.0	4.12	19.2
7.876	11.21672	26.0	5.10	19.2
7.886	11.20238	25.0	5.00	19.2
7.896	11.18807	29.0	5.39	19.2
7.906	11.17380	18.0	4.24	19.2
7.916	11.15957	25.0	5.00	19.2
7.926	11.14537	23.0	4.80	19.2
7.936	11.13121	22.0	4.69	19.2
7.946	11.11709	22.0	4.69	19.2
7.956	11.10300	15.0	3.87	19.2
7.967	11.08895	23.0	4.80	19.2
7.977	11.07493	27.0	5.20	19.2
7.987	11.06095	22.0	4.69	19.2
7.997	11.04700	25.0	5.00	19.2
8.007	11.03309	24.0	4.90	19.2
8.017	11.01922	20.0	4.47	19.2

8.027	11.00538	12.0	3.46	19.2
8.037	10.99157	15.0	3.87	19.2
8.047	10.97780	20.0	4.47	19.2
8.057	10.96406	25.0	5.00	19.2
8.068	10.95036	19.0	4.36	19.2
8.078	10.93669	11.0	3.32	19.2
8.088	10.92306	26.0	5.10	19.2
8.098	10.90946	20.0	4.47	19.2
8.108	10.89589	24.0	4.90	19.2
8.118	10.88236	17.0	4.12	19.2
8.128	10.86886	19.0	4.36	19.2
8.138	10.85539	23.0	4.80	19.2
8.148	10.84196	26.0	5.10	19.2
8.158	10.82856	36.0	6.00	19.2
8.169	10.81520	16.0	4.00	19.2
8.179	10.80187	21.0	4.58	19.2
8.189	10.78857	22.0	4.69	19.2
8.199	10.77530	20.0	4.47	19.2
8.209	10.76207	24.0	4.90	19.2
8.219	10.74887	26.0	5.10	19.2
8.229	10.73570	22.0	4.69	19.2
8.239	10.72256	26.0	5.10	19.2
8.249	10.70946	28.0	5.29	19.2
8.259	10.69638	33.0	5.74	19.2
8.270	10.68334	29.0	5.39	19.2
8.280	10.67033	23.0	4.80	19.2
8.290	10.65736	22.0	4.69	19.2
8.300	10.64441	25.0	5.00	19.2
8.310	10.63150	18.0	4.24	19.2
8.320	10.61862	23.0	4.80	19.2
8.330	10.60577	32.0	5.66	19.2
8.340	10.59295	33.0	5.74	19.2
8.350	10.58016	16.0	4.00	19.2
8.360	10.56740	22.0	4.69	19.2
8.371	10.55467	40.0	6.32	19.2
8.381	10.54197	26.0	5.10	19.2
8.391	10.52931	23.0	4.80	19.2
8.401	10.51667	21.0	4.58	19.2
8.411	10.50407	26.0	5.10	19.2
8.421	10.49149	20.0	4.47	19.2
8.431	10.47895	31.0	5.57	19.2
8.441	10.46643	25.0	5.00	19.2
8.451	10.45395	19.0	4.36	19.2
8.461	10.44150	19.0	4.36	19.2
8.472	10.42907	18.0	4.24	19.2
8.482	10.41668	26.0	5.10	19.2
8.492	10.40431	29.0	5.39	19.2
8.502	10.39197	18.0	4.24	19.2
8.512	10.37967	24.0	4.90	19.2
8.522	10.36739	26.0	5.10	19.2

8.532	10.35514	22.0	4.69	19.2
8.542	10.34292	21.0	4.58	19.2
8.552	10.33073	21.0	4.58	19.2
8.562	10.31857	21.0	4.58	19.2
8.573	10.30643	35.0	5.92	19.2
8.583	10.29433	32.0	5.66	19.2
8.593	10.28225	21.0	4.58	19.2
8.603	10.27020	29.0	5.39	19.2
8.613	10.25818	25.0	5.00	19.2
8.623	10.24619	32.0	5.66	19.2
8.633	10.23423	17.0	4.12	19.2
8.643	10.22229	27.0	5.20	19.2
8.653	10.21038	27.0	5.20	19.2
8.663	10.19850	32.0	5.66	19.2
8.674	10.18665	16.0	4.00	19.2
8.684	10.17483	26.0	5.10	19.2
8.694	10.16303	40.0	6.32	19.2
8.704	10.15126	19.0	4.36	19.2
8.714	10.13952	31.0	5.57	19.2
8.724	10.12780	28.0	5.29	19.2
8.734	10.11611	27.0	5.20	19.2
8.744	10.10445	23.0	4.80	19.2
8.754	10.09282	30.0	5.48	19.2
8.764	10.08121	32.0	5.66	19.2
8.775	10.06963	27.0	5.20	19.2
8.785	10.05808	21.0	4.58	19.2
8.795	10.04655	35.0	5.92	19.2
8.805	10.03505	24.0	4.90	19.2
8.815	10.02357	33.0	5.74	19.2
8.825	10.01213	33.0	5.74	19.2
8.835	10.00070	35.0	5.92	19.2
8.845	9.98931	30.0	5.48	19.2
8.855	9.97794	30.0	5.48	19.2
8.865	9.96660	29.0	5.39	19.2
8.876	9.95528	22.0	4.69	19.2
8.886	9.94398	28.0	5.29	19.2
8.896	9.93272	16.0	4.00	19.2
8.906	9.92148	23.0	4.80	19.2
8.916	9.91026	33.0	5.74	19.2
8.926	9.89907	44.0	6.63	19.2
8.936	9.88791	35.0	5.92	19.2
8.946	9.87677	26.0	5.10	19.2
8.956	9.86565	28.0	5.29	19.2
8.966	9.85456	24.0	4.90	19.2
8.977	9.84350	30.0	5.48	19.2
8.987	9.83246	27.0	5.20	19.2
8.997	9.82144	34.0	5.83	19.2
9.007	9.81045	21.0	4.58	19.2
9.017	9.79949	36.0	6.00	19.2
9.027	9.78855	23.0	4.80	19.2

9.037	9.77763	35.0	5.92	19.2
9.047	9.76674	27.0	5.20	19.2
9.057	9.75587	34.0	5.83	19.2
9.067	9.74503	42.0	6.48	19.2
9.077	9.73421	33.0	5.74	19.2
9.088	9.72341	25.0	5.00	19.2
9.098	9.71264	27.0	5.20	19.2
9.108	9.70190	42.0	6.48	19.2
9.118	9.69117	30.0	5.48	19.2
9.128	9.68047	39.0	6.24	19.2
9.138	9.66980	21.0	4.58	19.2
9.148	9.65914	33.0	5.74	19.2
9.158	9.64851	31.0	5.57	19.2
9.168	9.63791	34.0	5.83	19.2
9.178	9.62733	37.0	6.08	19.2
9.189	9.61677	29.0	5.39	19.2
9.199	9.60623	22.0	4.69	19.2
9.209	9.59572	33.0	5.74	19.2
9.219	9.58523	38.0	6.16	19.2
9.229	9.57476	31.0	5.57	19.2
9.239	9.56432	43.0	6.56	19.2
9.249	9.55390	31.0	5.57	19.2
9.259	9.54350	40.0	6.32	19.2
9.269	9.53313	32.0	5.66	19.2
9.279	9.52277	36.0	6.00	19.2
9.290	9.51244	33.0	5.74	19.2
9.300	9.50214	29.0	5.39	19.2
9.310	9.49185	35.0	5.92	19.2
9.320	9.48159	28.0	5.29	19.2
9.330	9.47135	33.0	5.74	19.2
9.340	9.46113	34.0	5.83	19.2
9.350	9.45093	32.0	5.66	19.2
9.360	9.44076	33.0	5.74	19.2
9.370	9.43061	33.0	5.74	19.2
9.380	9.42048	30.0	5.48	19.2
9.391	9.41037	31.0	5.57	19.2
9.401	9.40028	28.0	5.29	19.2
9.411	9.39021	40.0	6.32	19.2
9.421	9.38017	39.0	6.24	19.2
9.431	9.37015	40.0	6.32	19.2
9.441	9.36015	24.0	4.90	19.2
9.451	9.35017	27.0	5.20	19.2
9.461	9.34021	23.0	4.80	19.2
9.471	9.33027	42.0	6.48	19.2
9.481	9.32036	42.0	6.48	19.2
9.492	9.31046	36.0	6.00	19.2
9.502	9.30059	31.0	5.57	19.2
9.512	9.29074	39.0	6.24	19.2
9.522	9.28091	51.0	7.14	19.2
9.532	9.27110	31.0	5.57	19.2

9.542	9.26131	35.0	5.92	19.2
9.552	9.25154	35.0	5.92	19.2
9.562	9.24179	28.0	5.29	19.2
9.572	9.23206	33.0	5.74	19.2
9.582	9.22235	39.0	6.24	19.2
9.593	9.21267	42.0	6.48	19.2
9.603	9.20300	31.0	5.57	19.2
9.613	9.19335	36.0	6.00	19.2
9.623	9.18373	37.0	6.08	19.2
9.633	9.17412	43.0	6.56	19.2
9.643	9.16454	55.0	7.42	19.2
9.653	9.15497	40.0	6.32	19.2
9.663	9.14543	34.0	5.83	19.2
9.673	9.13590	49.0	7.00	19.2
9.683	9.12640	57.0	7.55	19.2
9.694	9.11691	39.0	6.24	19.2
9.704	9.10744	50.0	7.07	19.2
9.714	9.09800	30.0	5.48	19.2
9.724	9.08857	43.0	6.56	19.2
9.734	9.07916	38.0	6.16	19.2
9.744	9.06978	20.0	4.47	19.2
9.754	9.06041	46.0	6.78	19.2
9.764	9.05106	38.0	6.16	19.2
9.774	9.04173	36.0	6.00	19.2
9.784	9.03242	41.0	6.40	19.2
9.795	9.02313	37.0	6.08	19.2
9.805	9.01386	41.0	6.40	19.2
9.815	9.00461	26.0	5.10	19.2
9.825	8.99537	38.0	6.16	19.2
9.835	8.98616	34.0	5.83	19.2
9.845	8.97696	28.0	5.29	19.2
9.855	8.96779	31.0	5.57	19.2
9.865	8.95863	31.0	5.57	19.2
9.875	8.94949	43.0	6.56	19.2
9.885	8.94037	33.0	5.74	19.2
9.896	8.93127	29.0	5.39	19.2
9.906	8.92218	34.0	5.83	19.2
9.916	8.91312	37.0	6.08	19.2
9.926	8.90407	52.0	7.21	19.2
9.936	8.89505	51.0	7.14	19.2
9.946	8.88604	48.0	6.93	19.2
9.956	8.87705	41.0	6.40	19.2
9.966	8.86807	41.0	6.40	19.2
9.976	8.85912	39.0	6.24	19.2
9.986	8.85018	40.0	6.32	19.2
9.997	8.84126	25.0	5.00	19.2
10.007	8.83236	42.0	6.48	19.2
10.017	8.82348	44.0	6.63	19.2
10.027	8.81462	40.0	6.32	19.2
10.037	8.80577	42.0	6.48	19.2

10.047	8.79694	39.0	6.24	19.2
10.057	8.78813	49.0	7.00	19.2
10.067	8.77934	39.0	6.24	19.2
10.077	8.77056	43.0	6.56	19.2
10.087	8.76180	34.0	5.83	19.2
10.098	8.75306	38.0	6.16	19.2
10.108	8.74434	31.0	5.57	19.2
10.118	8.73563	53.0	7.28	19.2
10.128	8.72694	42.0	6.48	19.2
10.138	8.71827	41.0	6.40	19.2
10.148	8.70962	40.0	6.32	19.2
10.158	8.70098	50.0	7.07	19.2
10.168	8.69236	28.0	5.29	19.2
10.178	8.68376	37.0	6.08	19.2
10.188	8.67518	33.0	5.74	19.2
10.199	8.66661	45.0	6.71	19.2
10.209	8.65806	51.0	7.14	19.2
10.219	8.64952	49.0	7.00	19.2
10.229	8.64101	46.0	6.78	19.2
10.239	8.63251	35.0	5.92	19.2
10.249	8.62402	41.0	6.40	19.2
10.259	8.61555	45.0	6.71	19.2
10.269	8.60710	39.0	6.24	19.2
10.279	8.59867	42.0	6.48	19.2
10.289	8.59025	52.0	7.21	19.2
10.300	8.58185	44.0	6.63	19.2
10.310	8.57347	44.0	6.63	19.2
10.320	8.56510	60.0	7.75	19.2
10.330	8.55675	40.0	6.32	19.2
10.340	8.54841	41.0	6.40	19.2
10.350	8.54010	36.0	6.00	19.2
10.360	8.53179	32.0	5.66	19.2
10.370	8.52351	54.0	7.35	19.2
10.380	8.51524	40.0	6.32	19.2
10.390	8.50698	47.0	6.86	19.2
10.400	8.49875	44.0	6.63	19.2
10.411	8.49052	45.0	6.71	19.2
10.421	8.48232	37.0	6.08	19.2
10.431	8.47413	38.0	6.16	19.2
10.441	8.46595	45.0	6.71	19.2
10.451	8.45780	50.0	7.07	19.2
10.461	8.44965	62.0	7.87	19.2
10.471	8.44153	35.0	5.92	19.2
10.481	8.43341	39.0	6.24	19.2
10.491	8.42532	42.0	6.48	19.2
10.501	8.41724	53.0	7.28	19.2
10.512	8.40918	57.0	7.55	19.2
10.522	8.40113	41.0	6.40	19.2
10.532	8.39309	34.0	5.83	19.2
10.542	8.38507	44.0	6.63	19.2

10.552	8.37707	32.0	5.66	19.2
10.562	8.36908	41.0	6.40	19.2
10.572	8.36111	46.0	6.78	19.2
10.582	8.35316	55.0	7.42	19.2
10.592	8.34521	36.0	6.00	19.2
10.602	8.33729	42.0	6.48	19.2
10.613	8.32938	47.0	6.86	19.2
10.623	8.32148	43.0	6.56	19.2
10.633	8.31360	44.0	6.63	19.2
10.643	8.30573	51.0	7.14	19.2
10.653	8.29788	40.0	6.32	19.2
10.663	8.29005	37.0	6.08	19.2
10.673	8.28222	46.0	6.78	19.2
10.683	8.27442	42.0	6.48	19.2
10.693	8.26662	42.0	6.48	19.2
10.703	8.25885	43.0	6.56	19.2
10.714	8.25108	51.0	7.14	19.2
10.724	8.24334	33.0	5.74	19.2
10.734	8.23560	62.0	7.87	19.2
10.744	8.22788	42.0	6.48	19.2
10.754	8.22018	52.0	7.21	19.2
10.764	8.21249	37.0	6.08	19.2
10.774	8.20482	64.0	8.00	19.2
10.784	8.19715	39.0	6.24	19.2
10.794	8.18951	49.0	7.00	19.2
10.804	8.18188	41.0	6.40	19.2
10.815	8.17426	51.0	7.14	19.2
10.825	8.16665	52.0	7.21	19.2
10.835	8.15906	42.0	6.48	19.2
10.845	8.15149	53.0	7.28	19.2
10.855	8.14393	53.0	7.28	19.2
10.865	8.13638	41.0	6.40	19.2
10.875	8.12885	42.0	6.48	19.2
10.885	8.12133	49.0	7.00	19.2
10.895	8.11382	49.0	7.00	19.2
10.905	8.10633	42.0	6.48	19.2
10.916	8.09885	42.0	6.48	19.2
10.926	8.09139	45.0	6.71	19.2
10.936	8.08394	50.0	7.07	19.2
10.946	8.07650	46.0	6.78	19.2
10.956	8.06908	51.0	7.14	19.2
10.966	8.06167	57.0	7.55	19.2
10.976	8.05428	51.0	7.14	19.2
10.986	8.04690	60.0	7.75	19.2
10.996	8.03953	50.0	7.07	19.2
11.006	8.03218	43.0	6.56	19.2
11.017	8.02483	57.0	7.55	19.2
11.027	8.01751	38.0	6.16	19.2
11.037	8.01019	46.0	6.78	19.2
11.047	8.00289	50.0	7.07	19.2

11.057	7.99561	42.0	6.48	19.2
11.067	7.98833	51.0	7.14	19.2
11.077	7.98107	49.0	7.00	19.2
11.087	7.97382	55.0	7.42	19.2
11.097	7.96659	41.0	6.40	19.2
11.107	7.95937	56.0	7.48	19.2
11.118	7.95216	48.0	6.93	19.2
11.128	7.94497	48.0	6.93	19.2
11.138	7.93779	43.0	6.56	19.2
11.148	7.93062	44.0	6.63	19.2
11.158	7.92346	57.0	7.55	19.2
11.168	7.91632	38.0	6.16	19.2
11.178	7.90919	38.0	6.16	19.2
11.188	7.90207	63.0	7.94	19.2
11.198	7.89497	47.0	6.86	19.2
11.208	7.88788	49.0	7.00	19.2
11.219	7.88080	51.0	7.14	19.2
11.229	7.87374	60.0	7.75	19.2
11.239	7.86668	48.0	6.93	19.2
11.249	7.85964	63.0	7.94	19.2
11.259	7.85261	56.0	7.48	19.2
11.269	7.84560	43.0	6.56	19.2
11.279	7.83860	59.0	7.68	19.2
11.289	7.83161	47.0	6.86	19.2
11.299	7.82463	47.0	6.86	19.2
11.309	7.81767	64.0	8.00	19.2
11.320	7.81071	45.0	6.71	19.2
11.330	7.80377	50.0	7.07	19.2
11.340	7.79685	45.0	6.71	19.2
11.350	7.78993	57.0	7.55	19.2
11.360	7.78303	47.0	6.86	19.2
11.370	7.77614	51.0	7.14	19.2
11.380	7.76926	42.0	6.48	19.2
11.390	7.76239	49.0	7.00	19.2
11.400	7.75554	59.0	7.68	19.2
11.410	7.74870	46.0	6.78	19.2
11.421	7.74187	55.0	7.42	19.2
11.431	7.73505	43.0	6.56	19.2
11.441	7.72825	48.0	6.93	19.2
11.451	7.72145	67.0	8.19	19.2
11.461	7.71467	42.0	6.48	19.2
11.471	7.70790	49.0	7.00	19.2
11.481	7.70114	50.0	7.07	19.2
11.491	7.69440	54.0	7.35	19.2
11.501	7.68767	49.0	7.00	19.2
11.511	7.68094	45.0	6.71	19.2
11.522	7.67423	43.0	6.56	19.2
11.532	7.66754	59.0	7.68	19.2
11.542	7.66085	50.0	7.07	19.2
11.552	7.65417	50.0	7.07	19.2

11.562	7.64751	45.0	6.71	19.2
11.572	7.64086	43.0	6.56	19.2
11.582	7.63422	44.0	6.63	19.2
11.592	7.62759	62.0	7.87	19.2
11.602	7.62097	47.0	6.86	19.2
11.612	7.61437	55.0	7.42	19.2
11.623	7.60778	46.0	6.78	19.2
11.633	7.60119	62.0	7.87	19.2
11.643	7.59462	70.0	8.37	19.2
11.653	7.58806	73.0	8.54	19.2
11.663	7.58151	54.0	7.35	19.2
11.673	7.57498	37.0	6.08	19.2
11.683	7.56845	65.0	8.06	19.2
11.693	7.56194	50.0	7.07	19.2
11.703	7.55544	56.0	7.48	19.2
11.713	7.54894	64.0	8.00	19.2
11.723	7.54246	61.0	7.81	19.2
11.734	7.53599	64.0	8.00	19.2
11.744	7.52954	54.0	7.35	19.2
11.754	7.52309	60.0	7.75	19.2
11.764	7.51665	57.0	7.55	19.2
11.774	7.51023	51.0	7.14	19.2
11.784	7.50382	48.0	6.93	19.2
11.794	7.49741	57.0	7.55	19.2
11.804	7.49102	56.0	7.48	19.2
11.814	7.48464	74.0	8.60	19.2
11.824	7.47827	63.0	7.94	19.2
11.835	7.47191	53.0	7.28	19.2
11.845	7.46556	55.0	7.42	19.2
11.855	7.45923	60.0	7.75	19.2
11.865	7.45290	59.0	7.68	19.2
11.875	7.44658	68.0	8.25	19.2
11.885	7.44028	62.0	7.87	19.2
11.895	7.43398	65.0	8.06	19.2
11.905	7.42770	59.0	7.68	19.2
11.915	7.42143	36.0	6.00	19.2
11.925	7.41517	43.0	6.56	19.2
11.936	7.40891	56.0	7.48	19.2
11.946	7.40267	55.0	7.42	19.2
11.956	7.39644	65.0	8.06	19.2
11.966	7.39022	62.0	7.87	19.2
11.976	7.38401	60.0	7.75	19.2
11.986	7.37781	57.0	7.55	19.2
11.996	7.37163	71.0	8.43	19.2
12.006	7.36545	50.0	7.07	19.2
12.016	7.35928	62.0	7.87	19.2
12.026	7.35312	62.0	7.87	19.2
12.037	7.34698	54.0	7.35	19.2
12.047	7.34084	50.0	7.07	19.2
12.057	7.33471	83.0	9.11	19.2

12.067	7.32860	54.0	7.35	19.2
12.077	7.32249	62.0	7.87	19.2
12.087	7.31640	52.0	7.21	19.2
12.097	7.31031	59.0	7.68	19.2
12.107	7.30423	67.0	8.19	19.2
12.117	7.29817	61.0	7.81	19.2
12.127	7.29211	51.0	7.14	19.2
12.138	7.28607	67.0	8.19	19.2
12.148	7.28003	67.0	8.19	19.2
12.158	7.27401	63.0	7.94	19.2
12.168	7.26800	62.0	7.87	19.2
12.178	7.26199	57.0	7.55	19.2
12.188	7.25600	53.0	7.28	19.2
12.198	7.25001	52.0	7.21	19.2
12.208	7.24404	72.0	8.49	19.2
12.218	7.23807	58.0	7.62	19.2
12.228	7.23212	72.0	8.49	19.2
12.239	7.22617	70.0	8.37	19.2
12.249	7.22024	70.0	8.37	19.2
12.259	7.21431	68.0	8.25	19.2
12.269	7.20839	70.0	8.37	19.2
12.279	7.20249	70.0	8.37	19.2
12.289	7.19659	58.0	7.62	19.2
12.299	7.19071	61.0	7.81	19.2
12.309	7.18483	77.0	8.77	19.2
12.319	7.17896	55.0	7.42	19.2
12.329	7.17310	68.0	8.25	19.2
12.340	7.16726	61.0	7.81	19.2
12.350	7.16142	71.0	8.43	19.2
12.360	7.15559	61.0	7.81	19.2
12.370	7.14977	72.0	8.49	19.2
12.380	7.14396	65.0	8.06	19.2
12.390	7.13816	60.0	7.75	19.2
12.400	7.13237	62.0	7.87	19.2
12.410	7.12659	67.0	8.19	19.2
12.420	7.12081	55.0	7.42	19.2
12.430	7.11505	63.0	7.94	19.2
12.441	7.10930	57.0	7.55	19.2
12.451	7.10355	59.0	7.68	19.2
12.461	7.09782	71.0	8.43	19.2
12.471	7.09209	73.0	8.54	19.2
12.481	7.08638	73.0	8.54	19.2
12.491	7.08067	64.0	8.00	19.2
12.501	7.07497	71.0	8.43	19.2
12.511	7.06928	53.0	7.28	19.2
12.521	7.06361	66.0	8.12	19.2
12.531	7.05794	74.0	8.60	19.2
12.542	7.05227	65.0	8.06	19.2
12.552	7.04662	45.0	6.71	19.2
12.562	7.04098	63.0	7.94	19.2

12.572	7.03535	67.0	8.19	19.2
12.582	7.02972	71.0	8.43	19.2
12.592	7.02411	74.0	8.60	19.2
12.602	7.01850	71.0	8.43	19.2
12.612	7.01290	77.0	8.77	19.2
12.622	7.00732	64.0	8.00	19.2
12.632	7.00174	87.0	9.33	19.2
12.643	6.99617	70.0	8.37	19.2
12.653	6.99060	72.0	8.49	19.2
12.663	6.98505	77.0	8.77	19.2
12.673	6.97951	54.0	7.35	19.2
12.683	6.97397	70.0	8.37	19.2
12.693	6.96845	61.0	7.81	19.2
12.703	6.96293	81.0	9.00	19.2
12.713	6.95742	76.0	8.72	19.2
12.723	6.95192	65.0	8.06	19.2
12.733	6.94643	70.0	8.37	19.2
12.744	6.94095	64.0	8.00	19.2
12.754	6.93547	67.0	8.19	19.2
12.764	6.93001	74.0	8.60	19.2
12.774	6.92455	74.0	8.60	19.2
12.784	6.91910	80.0	8.94	19.2
12.794	6.91366	92.0	9.59	19.2
12.804	6.90823	75.0	8.66	19.2
12.814	6.90281	57.0	7.55	19.2
12.824	6.89740	68.0	8.25	19.2
12.834	6.89199	70.0	8.37	19.2
12.845	6.88660	77.0	8.77	19.2
12.855	6.88121	68.0	8.25	19.2
12.865	6.87583	70.0	8.37	19.2
12.875	6.87046	72.0	8.49	19.2
12.885	6.86510	68.0	8.25	19.2
12.895	6.85974	83.0	9.11	19.2
12.905	6.85440	72.0	8.49	19.2
12.915	6.84906	87.0	9.33	19.2
12.925	6.84373	81.0	9.00	19.2
12.935	6.83841	77.0	8.77	19.2
12.946	6.83310	67.0	8.19	19.2
12.956	6.82780	82.0	9.06	19.2
12.966	6.82250	88.0	9.38	19.2
12.976	6.81721	70.0	8.37	19.2
12.986	6.81193	74.0	8.60	19.2
12.996	6.80666	96.0	9.80	19.2
13.006	6.80140	84.0	9.17	19.2
13.016	6.79615	77.0	8.77	19.2
13.026	6.79090	69.0	8.31	19.2
13.036	6.78566	67.0	8.19	19.2
13.046	6.78043	91.0	9.54	19.2
13.057	6.77521	78.0	8.83	19.2
13.067	6.77000	96.0	9.80	19.2

13.077	6.76479	89.0	9.43	19.2
13.087	6.75959	74.0	8.60	19.2
13.097	6.75440	83.0	9.11	19.2
13.107	6.74922	90.0	9.49	19.2
13.117	6.74405	91.0	9.54	19.2
13.127	6.73888	76.0	8.72	19.2
13.137	6.73372	64.0	8.00	19.2
13.147	6.72857	74.0	8.60	19.2
13.158	6.72343	85.0	9.22	19.2
13.168	6.71830	72.0	8.49	19.2
13.178	6.71317	94.0	9.70	19.2
13.188	6.70805	89.0	9.43	19.2
13.198	6.70294	84.0	9.17	19.2
13.208	6.69784	70.0	8.37	19.2
13.218	6.69275	79.0	8.89	19.2
13.228	6.68766	83.0	9.11	19.2
13.238	6.68258	103	10.1	19.2
13.248	6.67751	100	10.0	19.2
13.259	6.67244	90.0	9.49	19.2
13.269	6.66739	84.0	9.17	19.2
13.279	6.66234	112	10.6	19.2
13.289	6.65730	82.0	9.06	19.2
13.299	6.65227	100	10.0	19.2
13.309	6.64724	67.0	8.19	19.2
13.319	6.64222	89.0	9.43	19.2
13.329	6.63721	98.0	9.90	19.2
13.339	6.63221	105	10.2	19.2
13.349	6.62722	101	10.0	19.2
13.360	6.62223	101	10.0	19.2
13.370	6.61725	88.0	9.38	19.2
13.380	6.61228	79.0	8.89	19.2
13.390	6.60731	89.0	9.43	19.2
13.400	6.60236	99.0	9.95	19.2
13.410	6.59741	107	10.3	19.2
13.420	6.59246	90.0	9.49	19.2
13.430	6.58753	107	10.3	19.2
13.440	6.58260	97.0	9.85	19.2
13.450	6.57768	104	10.2	19.2
13.461	6.57277	120	11.0	19.2
13.471	6.56787	113	10.6	19.2
13.481	6.56297	100	10.0	19.2
13.491	6.55808	100	10.0	19.2
13.501	6.55319	119	10.9	19.2
13.511	6.54832	103	10.1	19.2
13.521	6.54345	103	10.1	19.2
13.531	6.53859	90.0	9.49	19.2
13.541	6.53374	94.0	9.70	19.2
13.551	6.52889	107	10.3	19.2
13.562	6.52405	123	11.1	19.2
13.572	6.51922	109	10.4	19.2

13.582	6.51439	102	10.1	19.2
13.592	6.50957	106	10.3	19.2
13.602	6.50476	113	10.6	19.2
13.612	6.49996	113	10.6	19.2
13.622	6.49516	107	10.3	19.2
13.632	6.49038	107	10.3	19.2
13.642	6.48559	107	10.3	19.2
13.652	6.48082	95.0	9.75	19.2
13.663	6.47605	110	10.5	19.2
13.673	6.47129	101	10.0	19.2
13.683	6.46654	100	10.0	19.2
13.693	6.46179	114	10.7	19.2
13.703	6.45705	126	11.2	19.2
13.713	6.45232	127	11.3	19.2
13.723	6.44759	106	10.3	19.2
13.733	6.44287	124	11.1	19.2
13.743	6.43816	110	10.5	19.2
13.753	6.43346	86.0	9.27	19.2
13.764	6.42876	126	11.2	19.2
13.774	6.42407	130	11.4	19.2
13.784	6.41938	102	10.1	19.2
13.794	6.41471	109	10.4	19.2
13.804	6.41003	113	10.6	19.2
13.814	6.40537	122	11.0	19.2
13.824	6.40071	118	10.9	19.2
13.834	6.39606	124	11.1	19.2
13.844	6.39142	111	10.5	19.2
13.854	6.38678	116	10.8	19.2
13.865	6.38216	142	11.9	19.2
13.875	6.37753	117	10.8	19.2
13.885	6.37292	128	11.3	19.2
13.895	6.36831	119	10.9	19.2
13.905	6.36370	142	11.9	19.2
13.915	6.35911	121	11.0	19.2
13.925	6.35452	114	10.7	19.2
13.935	6.34994	131	11.4	19.2
13.945	6.34536	122	11.0	19.2
13.955	6.34079	122	11.0	19.2
13.966	6.33623	109	10.4	19.2
13.976	6.33167	112	10.6	19.2
13.986	6.32712	126	11.2	19.2
13.996	6.32258	125	11.2	19.2
14.006	6.31804	125	11.2	19.2
14.016	6.31351	133	11.5	19.2
14.026	6.30899	117	10.8	19.2
14.036	6.30447	113	10.6	19.2
14.046	6.29996	137	11.7	19.2
14.056	6.29546	108	10.4	19.2
14.067	6.29096	115	10.7	19.2
14.077	6.28647	125	11.2	19.2

14.087	6.28199	127	11.3	19.2
14.097	6.27751	146	12.1	19.2
14.107	6.27304	120	11.0	19.2
14.117	6.26857	132	11.5	19.2
14.127	6.26412	129	11.4	19.2
14.137	6.25966	146	12.1	19.2
14.147	6.25522	144	12.0	19.2
14.157	6.25078	142	11.9	19.2
14.168	6.24634	111	10.5	19.2
14.178	6.24192	136	11.7	19.2
14.188	6.23750	156	12.5	19.2
14.198	6.23308	148	12.2	19.2
14.208	6.22868	143	12.0	19.2
14.218	6.22427	145	12.0	19.2
14.228	6.21988	165	12.8	19.2
14.238	6.21549	152	12.3	19.2
14.248	6.21111	122	11.0	19.2
14.258	6.20673	144	12.0	19.2
14.269	6.20236	125	11.2	19.2
14.279	6.19800	148	12.2	19.2
14.289	6.19364	144	12.0	19.2
14.299	6.18929	139	11.8	19.2
14.309	6.18494	115	10.7	19.2
14.319	6.18060	159	12.6	19.2
14.329	6.17627	141	11.9	19.2
14.339	6.17194	144	12.0	19.2
14.349	6.16762	134	11.6	19.2
14.359	6.16330	160	12.6	19.2
14.369	6.15899	128	11.3	19.2
14.380	6.15469	122	11.0	19.2
14.390	6.15039	146	12.1	19.2
14.400	6.14610	141	11.9	19.2
14.410	6.14182	157	12.5	19.2
14.420	6.13754	139	11.8	19.2
14.430	6.13327	148	12.2	19.2
14.440	6.12900	141	11.9	19.2
14.450	6.12474	155	12.4	19.2
14.460	6.12048	148	12.2	19.2
14.470	6.11624	146	12.1	19.2
14.481	6.11199	170	13.0	19.2
14.491	6.10776	151	12.3	19.2
14.501	6.10352	160	12.6	19.2
14.511	6.09930	160	12.6	19.2
14.521	6.09508	162	12.7	19.2
14.531	6.09087	150	12.2	19.2
14.541	6.08666	149	12.2	19.2
14.551	6.08246	147	12.1	19.2
14.561	6.07826	186	13.6	19.2
14.571	6.07407	142	11.9	19.2
14.582	6.06989	168	13.0	19.2

14.592	6.06571	149	12.2	19.2
14.602	6.06154	149	12.2	19.2
14.612	6.05737	155	12.4	19.2
14.622	6.05321	158	12.6	19.2
14.632	6.04905	157	12.5	19.2
14.642	6.04490	180	13.4	19.2
14.652	6.04076	175	13.2	19.2
14.662	6.03662	132	11.5	19.2
14.672	6.03249	145	12.0	19.2
14.683	6.02836	146	12.1	19.2
14.693	6.02424	161	12.7	19.2
14.703	6.02013	163	12.8	19.2
14.713	6.01602	168	13.0	19.2
14.723	6.01191	162	12.7	19.2
14.733	6.00781	164	12.8	19.2
14.743	6.00372	151	12.3	19.2
14.753	5.99963	168	13.0	19.2
14.763	5.99555	157	12.5	19.2
14.773	5.99148	198	14.1	19.2
14.784	5.98741	161	12.7	19.2
14.794	5.98334	157	12.5	19.2
14.804	5.97928	136	11.7	19.2
14.814	5.97523	180	13.4	19.2
14.824	5.97118	185	13.6	19.2
14.834	5.96714	196	14.0	19.2
14.844	5.96310	171	13.1	19.2
14.854	5.95907	153	12.4	19.2
14.864	5.95504	184	13.6	19.2
14.874	5.95102	170	13.0	19.2
14.885	5.94701	163	12.8	19.2
14.895	5.94300	186	13.6	19.2
14.905	5.93899	195	14.0	19.2
14.915	5.93500	162	12.7	19.2
14.925	5.93100	162	12.7	19.2
14.935	5.92701	146	12.1	19.2
14.945	5.92303	198	14.1	19.2
14.955	5.91906	158	12.6	19.2
14.965	5.91508	155	12.4	19.2
14.975	5.91112	188	13.7	19.2
14.986	5.90716	179	13.4	19.2
14.996	5.90320	170	13.0	19.2
15.006	5.89925	205	14.3	19.2
15.016	5.89531	184	13.6	19.2
15.026	5.89137	183	13.5	19.2
15.036	5.88743	172	13.1	19.2
15.046	5.88350	176	13.3	19.2
15.056	5.87958	163	12.8	19.2
15.066	5.87566	176	13.3	19.2
15.076	5.87175	194	13.9	19.2
15.087	5.86784	179	13.4	19.2

15.097	5.86394	162	12.7	19.2
15.107	5.86004	164	12.8	19.2
15.117	5.85615	189	13.7	19.2
15.127	5.85226	167	12.9	19.2
15.137	5.84838	160	12.6	19.2
15.147	5.84450	186	13.6	19.2
15.157	5.84063	163	12.8	19.2
15.167	5.83676	183	13.5	19.2
15.177	5.83290	165	12.8	19.2
15.188	5.82905	212	14.6	19.2
15.198	5.82520	182	13.5	19.2
15.208	5.82135	197	14.0	19.2
15.218	5.81751	186	13.6	19.2
15.228	5.81367	183	13.5	19.2
15.238	5.80984	193	13.9	19.2
15.248	5.80602	209	14.5	19.2
15.258	5.80220	189	13.7	19.2
15.268	5.79838	188	13.7	19.2
15.278	5.79457	182	13.5	19.2
15.289	5.79077	190	13.8	19.2
15.299	5.78697	214	14.6	19.2
15.309	5.78317	184	13.6	19.2
15.319	5.77938	193	13.9	19.2
15.329	5.77560	189	13.7	19.2
15.339	5.77182	188	13.7	19.2
15.349	5.76804	209	14.5	19.2
15.359	5.76427	214	14.6	19.2
15.369	5.76051	207	14.4	19.2
15.379	5.75675	168	13.0	19.2
15.390	5.75299	182	13.5	19.2
15.400	5.74924	205	14.3	19.2
15.410	5.74550	202	14.2	19.2
15.420	5.74176	188	13.7	19.2
15.430	5.73802	194	13.9	19.2
15.440	5.73429	189	13.7	19.2
15.450	5.73057	200	14.1	19.2
15.460	5.72684	188	13.7	19.2
15.470	5.72313	213	14.6	19.2
15.480	5.71942	210	14.5	19.2
15.491	5.71571	194	13.9	19.2
15.501	5.71201	193	13.9	19.2
15.511	5.70831	229	15.1	19.2
15.521	5.70462	238	15.4	19.2
15.531	5.70094	224	15.0	19.2
15.541	5.69725	197	14.0	19.2
15.551	5.69358	219	14.8	19.2
15.561	5.68990	214	14.6	19.2
15.571	5.68624	203	14.2	19.2
15.581	5.68257	206	14.4	19.2
15.592	5.67892	208	14.4	19.2

15.602	5.67526	197	14.0	19.2
15.612	5.67161	226	15.0	19.2
15.622	5.66797	207	14.4	19.2
15.632	5.66433	208	14.4	19.2
15.642	5.66070	184	13.6	19.2
15.652	5.65707	195	14.0	19.2
15.662	5.65344	210	14.5	19.2
15.672	5.64982	227	15.1	19.2
15.682	5.64621	190	13.8	19.2
15.692	5.64259	195	14.0	19.2
15.703	5.63899	211	14.5	19.2
15.713	5.63539	188	13.7	19.2
15.723	5.63179	204	14.3	19.2
15.733	5.62820	226	15.0	19.2
15.743	5.62461	195	14.0	19.2
15.753	5.62103	205	14.3	19.2
15.763	5.61745	234	15.3	19.2
15.773	5.61387	225	15.0	19.2
15.783	5.61030	206	14.4	19.2
15.793	5.60674	211	14.5	19.2
15.804	5.60318	205	14.3	19.2
15.814	5.59962	201	14.2	19.2
15.824	5.59607	223	14.9	19.2
15.834	5.59253	244	15.6	19.2
15.844	5.58898	233	15.3	19.2
15.854	5.58545	213	14.6	19.2
15.864	5.58191	221	14.9	19.2
15.874	5.57838	232	15.2	19.2
15.884	5.57486	226	15.0	19.2
15.894	5.57134	222	14.9	19.2
15.905	5.56783	218	14.8	19.2
15.915	5.56432	196	14.0	19.2
15.925	5.56081	223	14.9	19.2
15.935	5.55731	249	15.8	19.2
15.945	5.55381	214	14.6	19.2
15.955	5.55032	208	14.4	19.2
15.965	5.54683	256	16.0	19.2
15.975	5.54335	230	15.2	19.2
15.985	5.53987	230	15.2	19.2
15.995	5.53639	232	15.2	19.2
16.006	5.53292	237	15.4	19.2
16.016	5.52945	209	14.5	19.2
16.026	5.52599	214	14.6	19.2
16.036	5.52253	201	14.2	19.2
16.046	5.51908	212	14.6	19.2
16.056	5.51563	212	14.6	19.2
16.066	5.51219	208	14.4	19.2
16.076	5.50875	247	15.7	19.2
16.086	5.50531	209	14.5	19.2
16.096	5.50188	238	15.4	19.2

16.107	5.49845	247	15.7	19.2
16.117	5.49503	235	15.3	19.2
16.127	5.49161	222	14.9	19.2
16.137	5.48820	217	14.7	19.2
16.147	5.48479	221	14.9	19.2
16.157	5.48138	229	15.1	19.2
16.167	5.47798	230	15.2	19.2
16.177	5.47458	228	15.1	19.2
16.187	5.47119	252	15.9	19.2
16.197	5.46780	236	15.4	19.2
16.208	5.46442	241	15.5	19.2
16.218	5.46104	243	15.6	19.2
16.228	5.45766	251	15.8	19.2
16.238	5.45429	246	15.7	19.2
16.248	5.45092	253	15.9	19.2
16.258	5.44756	201	14.2	19.2
16.268	5.44420	246	15.7	19.2
16.278	5.44085	252	15.9	19.2
16.288	5.43750	216	14.7	19.2
16.298	5.43415	229	15.1	19.2
16.309	5.43081	258	16.1	19.2
16.319	5.42747	233	15.3	19.2
16.329	5.42413	218	14.8	19.2
16.339	5.42080	205	14.3	19.2
16.349	5.41748	230	15.2	19.2
16.359	5.41416	232	15.2	19.2
16.369	5.41084	203	14.2	19.2
16.379	5.40753	248	15.7	19.2
16.389	5.40422	270	16.4	19.2
16.399	5.40091	231	15.2	19.2
16.410	5.39761	220	14.8	19.2
16.420	5.39431	245	15.7	19.2
16.430	5.39102	227	15.1	19.2
16.440	5.38773	224	15.0	19.2
16.450	5.38444	234	15.3	19.2
16.460	5.38116	219	14.8	19.2
16.470	5.37789	221	14.9	19.2
16.480	5.37461	249	15.8	19.2
16.490	5.37135	258	16.1	19.2
16.500	5.36808	220	14.8	19.2
16.511	5.36482	243	15.6	19.2
16.521	5.36156	264	16.2	19.2
16.531	5.35831	257	16.0	19.2
16.541	5.35506	252	15.9	19.2
16.551	5.35182	269	16.4	19.2
16.561	5.34858	282	16.8	19.2
16.571	5.34534	251	15.8	19.2
16.581	5.34211	251	15.8	19.2
16.591	5.33888	239	15.5	19.2
16.601	5.33565	245	15.7	19.2

16.612	5.33243	225	15.0	19.2
16.622	5.32921	251	15.8	19.2
16.632	5.32600	264	16.2	19.2
16.642	5.32279	274	16.6	19.2
16.652	5.31958	266	16.3	19.2
16.662	5.31638	251	15.8	19.2
16.672	5.31319	267	16.3	19.2
16.682	5.30999	255	16.0	19.2
16.692	5.30680	233	15.3	19.2
16.702	5.30362	263	16.2	19.2
16.713	5.30043	292	17.1	19.2
16.723	5.29726	247	15.7	19.2
16.733	5.29408	258	16.1	19.2
16.743	5.29091	252	15.9	19.2
16.753	5.28774	288	17.0	19.2
16.763	5.28458	239	15.5	19.2
16.773	5.28142	245	15.7	19.2
16.783	5.27827	226	15.0	19.2
16.793	5.27511	238	15.4	19.2
16.803	5.27197	251	15.8	19.2
16.814	5.26882	255	16.0	19.2
16.824	5.26568	274	16.6	19.2
16.834	5.26255	219	14.8	19.2
16.844	5.25941	248	15.7	19.2
16.854	5.25628	244	15.6	19.2
16.864	5.25316	251	15.8	19.2
16.874	5.25004	270	16.4	19.2
16.884	5.24692	252	15.9	19.2
16.894	5.24381	267	16.3	19.2
16.904	5.24070	222	14.9	19.2
16.915	5.23759	266	16.3	19.2
16.925	5.23449	262	16.2	19.2
16.935	5.23139	267	16.3	19.2
16.945	5.22829	261	16.2	19.2
16.955	5.22520	235	15.3	19.2
16.965	5.22211	266	16.3	19.2
16.975	5.21903	253	15.9	19.2
16.985	5.21595	244	15.6	19.2
16.995	5.21287	259	16.1	19.2
17.005	5.20980	269	16.4	19.2
17.015	5.20673	267	16.3	19.2
17.026	5.20366	284	16.9	19.2
17.036	5.20060	252	15.9	19.2
17.046	5.19754	266	16.3	19.2
17.056	5.19449	280	16.7	19.2
17.066	5.19144	257	16.0	19.2
17.076	5.18839	267	16.3	19.2
17.086	5.18535	249	15.8	19.2
17.096	5.18231	275	16.6	19.2
17.106	5.17927	281	16.8	19.2

17.116	5.17624	247	15.7	19.2
17.127	5.17321	294	17.1	19.2
17.137	5.17018	237	15.4	19.2
17.147	5.16716	259	16.1	19.2
17.157	5.16414	265	16.3	19.2
17.167	5.16112	261	16.2	19.2
17.177	5.15811	261	16.2	19.2
17.187	5.15510	219	14.8	19.2
17.197	5.15210	244	15.6	19.2
17.207	5.14910	247	15.7	19.2
17.217	5.14610	279	16.7	19.2
17.228	5.14311	261	16.2	19.2
17.238	5.14012	261	16.2	19.2
17.248	5.13713	282	16.8	19.2
17.258	5.13415	279	16.7	19.2
17.268	5.13117	239	15.5	19.2
17.278	5.12819	275	16.6	19.2
17.288	5.12522	263	16.2	19.2
17.298	5.12225	278	16.7	19.2
17.308	5.11928	262	16.2	19.2
17.318	5.11632	248	15.7	19.2
17.329	5.11336	254	15.9	19.2
17.339	5.11040	280	16.7	19.2
17.349	5.10745	281	16.8	19.2
17.359	5.10450	283	16.8	19.2
17.369	5.10156	280	16.7	19.2
17.379	5.09862	261	16.2	19.2
17.389	5.09568	280	16.7	19.2
17.399	5.09274	270	16.4	19.2
17.409	5.08981	268	16.4	19.2
17.419	5.08688	272	16.5	19.2
17.430	5.08396	255	16.0	19.2
17.440	5.08104	256	16.0	19.2
17.450	5.07812	269	16.4	19.2
17.460	5.07520	269	16.4	19.2
17.470	5.07229	269	16.4	19.2
17.480	5.06938	286	16.9	19.2
17.490	5.06648	272	16.5	19.2
17.500	5.06358	294	17.1	19.2
17.510	5.06068	245	15.7	19.2
17.520	5.05779	312	17.7	19.2
17.531	5.05490	259	16.1	19.2
17.541	5.05201	271	16.5	19.2
17.551	5.04912	296	17.2	19.2
17.561	5.04624	303	17.4	19.2
17.571	5.04337	283	16.8	19.2
17.581	5.04049	271	16.5	19.2
17.591	5.03762	280	16.7	19.2
17.601	5.03475	280	16.7	19.2
17.611	5.03189	319	17.9	19.2

17.621	5.02903	284	16.9	19.2
17.632	5.02617	294	17.1	19.2
17.642	5.02331	291	17.1	19.2
17.652	5.02046	257	16.0	19.2
17.662	5.01761	294	17.1	19.2
17.672	5.01477	279	16.7	19.2
17.682	5.01193	302	17.4	19.2
17.692	5.00909	268	16.4	19.2
17.702	5.00626	272	16.5	19.2
17.712	5.00342	310	17.6	19.2
17.722	5.00060	270	16.4	19.2
17.733	4.99777	295	17.2	19.2
17.743	4.99495	282	16.8	19.2
17.753	4.99213	314	17.7	19.2
17.763	4.98931	258	16.1	19.2
17.773	4.98650	295	17.2	19.2
17.783	4.98369	262	16.2	19.2
17.793	4.98089	274	16.6	19.2
17.803	4.97808	289	17.0	19.2
17.813	4.97528	284	16.9	19.2
17.823	4.97249	291	17.1	19.2
17.834	4.96969	268	16.4	19.2
17.844	4.96690	284	16.9	19.2
17.854	4.96412	302	17.4	19.2
17.864	4.96133	279	16.7	19.2
17.874	4.95855	279	16.7	19.2
17.884	4.95578	295	17.2	19.2
17.894	4.95300	298	17.3	19.2
17.904	4.95023	272	16.5	19.2
17.914	4.94746	309	17.6	19.2
17.924	4.94470	293	17.1	19.2
17.935	4.94194	304	17.4	19.2
17.945	4.93918	276	16.6	19.2
17.955	4.93642	295	17.2	19.2
17.965	4.93367	296	17.2	19.2
17.975	4.93092	266	16.3	19.2
17.985	4.92817	285	16.9	19.2
17.995	4.92543	290	17.0	19.2
18.005	4.92269	290	17.0	19.2
18.015	4.91995	294	17.1	19.2
18.025	4.91722	284	16.9	19.2
18.036	4.91449	291	17.1	19.2
18.046	4.91176	295	17.2	19.2
18.056	4.90904	274	16.6	19.2
18.066	4.90632	280	16.7	19.2
18.076	4.90360	297	17.2	19.2
18.086	4.90088	287	16.9	19.2
18.096	4.89817	309	17.6	19.2
18.106	4.89546	296	17.2	19.2
18.116	4.89275	280	16.7	19.2

18.126	4.89005	301	17.3	19.2
18.137	4.88735	287	16.9	19.2
18.147	4.88465	312	17.7	19.2
18.157	4.88196	287	16.9	19.2
18.167	4.87927	306	17.5	19.2
18.177	4.87658	254	15.9	19.2
18.187	4.87389	322	17.9	19.2
18.197	4.87121	276	16.6	19.2
18.207	4.86853	285	16.9	19.2
18.217	4.86586	276	16.6	19.2
18.227	4.86318	259	16.1	19.2
18.237	4.86051	262	16.2	19.2
18.248	4.85785	288	17.0	19.2
18.258	4.85518	317	17.8	19.2
18.268	4.85252	306	17.5	19.2
18.278	4.84986	297	17.2	19.2
18.288	4.84721	313	17.7	19.2
18.298	4.84455	305	17.5	19.2
18.308	4.84190	282	16.8	19.2
18.318	4.83926	312	17.7	19.2
18.328	4.83661	297	17.2	19.2
18.338	4.83397	294	17.1	19.2
18.349	4.83134	281	16.8	19.2
18.359	4.82870	286	16.9	19.2
18.369	4.82607	294	17.1	19.2
18.379	4.82344	315	17.7	19.2
18.389	4.82081	266	16.3	19.2
18.399	4.81819	330	18.2	19.2
18.409	4.81557	338	18.4	19.2
18.419	4.81295	268	16.4	19.2
18.429	4.81034	268	16.4	19.2
18.439	4.80772	314	17.7	19.2
18.450	4.80512	320	17.9	19.2
18.460	4.80251	292	17.1	19.2
18.470	4.79991	304	17.4	19.2
18.480	4.79731	318	17.8	19.2
18.490	4.79471	260	16.1	19.2
18.500	4.79211	302	17.4	19.2
18.510	4.78952	300	17.3	19.2
18.520	4.78693	288	17.0	19.2
18.530	4.78435	294	17.1	19.2
18.540	4.78176	282	16.8	19.2
18.551	4.77918	301	17.3	19.2
18.561	4.77661	289	17.0	19.2
18.571	4.77403	294	17.1	19.2
18.581	4.77146	287	16.9	19.2
18.591	4.76889	325	18.0	19.2
18.601	4.76632	297	17.2	19.2
18.611	4.76376	280	16.7	19.2
18.621	4.76120	279	16.7	19.2

18.631	4.75864	287	16.9	19.2
18.641	4.75608	316	17.8	19.2
18.652	4.75353	302	17.4	19.2
18.662	4.75098	268	16.4	19.2
18.672	4.74844	315	17.7	19.2
18.682	4.74589	294	17.1	19.2
18.692	4.74335	310	17.6	19.2
18.702	4.74081	299	17.3	19.2
18.712	4.73828	279	16.7	19.2
18.722	4.73574	283	16.8	19.2
18.732	4.73321	310	17.6	19.2
18.742	4.73068	303	17.4	19.2
18.753	4.72816	316	17.8	19.2
18.763	4.72564	297	17.2	19.2
18.773	4.72312	314	17.7	19.2
18.783	4.72060	298	17.3	19.2
18.793	4.71809	301	17.3	19.2
18.803	4.71558	310	17.6	19.2
18.813	4.71307	290	17.0	19.2
18.823	4.71056	298	17.3	19.2
18.833	4.70806	281	16.8	19.2
18.843	4.70556	301	17.3	19.2
18.854	4.70306	289	17.0	19.2
18.864	4.70056	313	17.7	19.2
18.874	4.69807	308	17.5	19.2
18.884	4.69558	313	17.7	19.2
18.894	4.69309	279	16.7	19.2
18.904	4.69061	293	17.1	19.2
18.914	4.68813	329	18.1	19.2
18.924	4.68565	293	17.1	19.2
18.934	4.68317	311	17.6	19.2
18.944	4.68070	297	17.2	19.2
18.955	4.67823	291	17.1	19.2
18.965	4.67576	273	16.5	19.2
18.975	4.67329	324	18.0	19.2
18.985	4.67083	316	17.8	19.2
18.995	4.66837	278	16.7	19.2
19.005	4.66591	292	17.1	19.2
19.015	4.66346	338	18.4	19.2
19.025	4.66100	261	16.2	19.2
19.035	4.65855	304	17.4	19.2
19.045	4.65611	308	17.5	19.2
19.056	4.65366	305	17.5	19.2
19.066	4.65122	317	17.8	19.2
19.076	4.64878	299	17.3	19.2
19.086	4.64634	305	17.5	19.2
19.096	4.64391	303	17.4	19.2
19.106	4.64148	327	18.1	19.2
19.116	4.63905	312	17.7	19.2
19.126	4.63662	296	17.2	19.2

19.136	4.63419	312	17.7	19.2
19.146	4.63177	320	17.9	19.2
19.157	4.62935	298	17.3	19.2
19.167	4.62694	304	17.4	19.2
19.177	4.62452	329	18.1	19.2
19.187	4.62211	302	17.4	19.2
19.197	4.61970	284	16.9	19.2
19.207	4.61730	279	16.7	19.2
19.217	4.61489	304	17.4	19.2
19.227	4.61249	310	17.6	19.2
19.237	4.61009	320	17.9	19.2
19.247	4.60770	298	17.3	19.2
19.258	4.60530	283	16.8	19.2
19.268	4.60291	303	17.4	19.2
19.278	4.60052	290	17.0	19.2
19.288	4.59814	276	16.6	19.2
19.298	4.59575	309	17.6	19.2
19.308	4.59337	284	16.9	19.2
19.318	4.59099	306	17.5	19.2
19.328	4.58862	306	17.5	19.2
19.338	4.58624	322	17.9	19.2
19.348	4.58387	286	16.9	19.2
19.359	4.58151	314	17.7	19.2
19.369	4.57914	299	17.3	19.2
19.379	4.57678	281	16.8	19.2
19.389	4.57441	317	17.8	19.2
19.399	4.57206	337	18.4	19.2
19.409	4.56970	312	17.7	19.2
19.419	4.56735	346	18.6	19.2
19.429	4.56499	311	17.6	19.2
19.439	4.56265	293	17.1	19.2
19.449	4.56030	309	17.6	19.2
19.460	4.55795	344	18.5	19.2
19.470	4.55561	312	17.7	19.2
19.480	4.55327	310	17.6	19.2
19.490	4.55094	307	17.5	19.2
19.500	4.54860	304	17.4	19.2
19.510	4.54627	294	17.1	19.2
19.520	4.54394	290	17.0	19.2
19.530	4.54162	265	16.3	19.2
19.540	4.53929	312	17.7	19.2
19.550	4.53697	294	17.1	19.2
19.560	4.53465	302	17.4	19.2
19.571	4.53233	302	17.4	19.2
19.581	4.53002	313	17.7	19.2
19.591	4.52770	264	16.2	19.2
19.601	4.52539	332	18.2	19.2
19.611	4.52309	284	16.9	19.2
19.621	4.52078	303	17.4	19.2
19.631	4.51848	313	17.7	19.2

19.641	4.51618	297	17.2	19.2
19.651	4.51388	294	17.1	19.2
19.661	4.51158	309	17.6	19.2
19.672	4.50929	335	18.3	19.2
19.682	4.50700	292	17.1	19.2
19.692	4.50471	348	18.7	19.2
19.702	4.50242	336	18.3	19.2
19.712	4.50014	286	16.9	19.2
19.722	4.49786	274	16.6	19.2
19.732	4.49558	329	18.1	19.2
19.742	4.49330	300	17.3	19.2
19.752	4.49103	287	16.9	19.2
19.762	4.48876	288	17.0	19.2
19.773	4.48649	348	18.7	19.2
19.783	4.48422	286	16.9	19.2
19.793	4.48195	308	17.5	19.2
19.803	4.47969	269	16.4	19.2
19.813	4.47743	269	16.4	19.2
19.823	4.47517	319	17.9	19.2
19.833	4.47291	303	17.4	19.2
19.843	4.47066	318	17.8	19.2
19.853	4.46841	295	17.2	19.2
19.863	4.46616	329	18.1	19.2
19.874	4.46391	280	16.7	19.2
19.884	4.46167	327	18.1	19.2
19.894	4.45943	314	17.7	19.2
19.904	4.45719	303	17.4	19.2
19.914	4.45495	313	17.7	19.2
19.924	4.45271	319	17.9	19.2
19.934	4.45048	329	18.1	19.2
19.944	4.44825	314	17.7	19.2
19.954	4.44602	276	16.6	19.2
19.964	4.44380	339	18.4	19.2
19.975	4.44157	313	17.7	19.2
19.985	4.43935	296	17.2	19.2
19.995	4.43713	290	17.0	19.2
20.005	4.43491	335	18.3	19.2
20.015	4.43270	336	18.3	19.2
20.025	4.43049	325	18.0	19.2
20.035	4.42827	302	17.4	19.2
20.045	4.42607	295	17.2	19.2
20.055	4.42386	322	17.9	19.2
20.065	4.42166	294	17.1	19.2
20.076	4.41945	330	18.2	19.2
20.086	4.41726	317	17.8	19.2
20.096	4.41506	325	18.0	19.2
20.106	4.41286	301	17.3	19.2
20.116	4.41067	328	18.1	19.2
20.126	4.40848	295	17.2	19.2
20.136	4.40629	310	17.6	19.2

20.146	4.40411	297	17.2	19.2
20.156	4.40192	335	18.3	19.2
20.166	4.39974	306	17.5	19.2
20.177	4.39756	304	17.4	19.2
20.187	4.39538	311	17.6	19.2
20.197	4.39321	293	17.1	19.2
20.207	4.39104	344	18.5	19.2
20.217	4.38887	342	18.5	19.2
20.227	4.38670	322	17.9	19.2
20.237	4.38453	308	17.5	19.2
20.247	4.38237	306	17.5	19.2
20.257	4.38020	302	17.4	19.2
20.267	4.37804	330	18.2	19.2
20.278	4.37589	307	17.5	19.2
20.288	4.37373	322	17.9	19.2
20.298	4.37158	311	17.6	19.2
20.308	4.36943	295	17.2	19.2
20.318	4.36728	307	17.5	19.2
20.328	4.36513	302	17.4	19.2
20.338	4.36299	310	17.6	19.2
20.348	4.36084	283	16.8	19.2
20.358	4.35870	349	18.7	19.2
20.368	4.35656	340	18.4	19.2
20.379	4.35443	320	17.9	19.2
20.389	4.35229	301	17.3	19.2
20.399	4.35016	299	17.3	19.2
20.409	4.34803	320	17.9	19.2
20.419	4.34590	332	18.2	19.2
20.429	4.34378	293	17.1	19.2
20.439	4.34165	311	17.6	19.2
20.449	4.33953	304	17.4	19.2
20.459	4.33741	307	17.5	19.2
20.469	4.33530	327	18.1	19.2
20.480	4.33318	306	17.5	19.2
20.490	4.33107	315	17.7	19.2
20.500	4.32896	328	18.1	19.2
20.510	4.32685	342	18.5	19.2
20.520	4.32474	309	17.6	19.2
20.530	4.32264	302	17.4	19.2
20.540	4.32054	319	17.9	19.2
20.550	4.31843	339	18.4	19.2
20.560	4.31634	336	18.3	19.2
20.570	4.31424	305	17.5	19.2
20.581	4.31215	341	18.5	19.2
20.591	4.31005	280	16.7	19.2
20.601	4.30796	333	18.2	19.2
20.611	4.30588	312	17.7	19.2
20.621	4.30379	306	17.5	19.2
20.631	4.30171	302	17.4	19.2
20.641	4.29962	349	18.7	19.2

20.651	4.29754	355	18.8	19.2
20.661	4.29547	284	16.9	19.2
20.671	4.29339	326	18.1	19.2
20.682	4.29132	303	17.4	19.2
20.692	4.28924	324	18.0	19.2
20.702	4.28717	323	18.0	19.2
20.712	4.28511	302	17.4	19.2
20.722	4.28304	311	17.6	19.2
20.732	4.28098	315	17.7	19.2
20.742	4.27892	309	17.6	19.2
20.752	4.27686	336	18.3	19.2
20.762	4.27480	296	17.2	19.2
20.772	4.27274	317	17.8	19.2
20.783	4.27069	311	17.6	19.2
20.793	4.26864	304	17.4	19.2
20.803	4.26659	303	17.4	19.2
20.813	4.26454	332	18.2	19.2
20.823	4.26250	349	18.7	19.2
20.833	4.26045	310	17.6	19.2
20.843	4.25841	325	18.0	19.2
20.853	4.25637	301	17.3	19.2
20.863	4.25433	339	18.4	19.2
20.873	4.25230	313	17.7	19.2
20.883	4.25026	315	17.7	19.2
20.894	4.24823	287	16.9	19.2
20.904	4.24620	344	18.5	19.2
20.914	4.24418	316	17.8	19.2
20.924	4.24215	304	17.4	19.2
20.934	4.24013	309	17.6	19.2
20.944	4.23810	342	18.5	19.2
20.954	4.23608	328	18.1	19.2
20.964	4.23407	319	17.9	19.2
20.974	4.23205	328	18.1	19.2
20.984	4.23004	323	18.0	19.2
20.995	4.22802	332	18.2	19.2
21.005	4.22601	306	17.5	19.2
21.015	4.22401	315	17.7	19.2
21.025	4.22200	313	17.7	19.2
21.035	4.22000	292	17.1	19.2
21.045	4.21799	311	17.6	19.2
21.055	4.21599	332	18.2	19.2
21.065	4.21399	310	17.6	19.2
21.075	4.21200	304	17.4	19.2
21.085	4.21000	298	17.3	19.2
21.096	4.20801	291	17.1	19.2
21.106	4.20602	296	17.2	19.2
21.116	4.20403	342	18.5	19.2
21.126	4.20204	300	17.3	19.2
21.136	4.20006	329	18.1	19.2
21.146	4.19808	329	18.1	19.2

21.156	4.19609	305	17.5	19.2
21.166	4.19412	310	17.6	19.2
21.176	4.19214	320	17.9	19.2
21.186	4.19016	278	16.7	19.2
21.197	4.18819	317	17.8	19.2
21.207	4.18622	334	18.3	19.2
21.217	4.18425	349	18.7	19.2
21.227	4.18228	314	17.7	19.2
21.237	4.18031	349	18.7	19.2
21.247	4.17835	367	19.2	19.2
21.257	4.17639	346	18.6	19.2
21.267	4.17443	302	17.4	19.2
21.277	4.17247	336	18.3	19.2
21.287	4.17051	313	17.7	19.2
21.298	4.16856	321	17.9	19.2
21.308	4.16660	338	18.4	19.2
21.318	4.16465	315	17.7	19.2
21.328	4.16270	321	17.9	19.2
21.338	4.16076	314	17.7	19.2
21.348	4.15881	316	17.8	19.2
21.358	4.15687	327	18.1	19.2
21.368	4.15492	306	17.5	19.2
21.378	4.15298	317	17.8	19.2
21.388	4.15105	324	18.0	19.2
21.399	4.14911	334	18.3	19.2
21.409	4.14718	329	18.1	19.2
21.419	4.14524	328	18.1	19.2
21.429	4.14331	332	18.2	19.2
21.439	4.14138	304	17.4	19.2
21.449	4.13946	313	17.7	19.2
21.459	4.13753	349	18.7	19.2
21.469	4.13561	313	17.7	19.2
21.479	4.13369	353	18.8	19.2
21.489	4.13177	357	18.9	19.2
21.500	4.12985	314	17.7	19.2
21.510	4.12793	328	18.1	19.2
21.520	4.12602	323	18.0	19.2
21.530	4.12410	318	17.8	19.2
21.540	4.12219	316	17.8	19.2
21.550	4.12028	316	17.8	19.2
21.560	4.11838	321	17.9	19.2
21.570	4.11647	319	17.9	19.2
21.580	4.11457	342	18.5	19.2
21.590	4.11267	309	17.6	19.2
21.601	4.11077	289	17.0	19.2
21.611	4.10887	318	17.8	19.2
21.621	4.10697	308	17.5	19.2
21.631	4.10508	326	18.1	19.2
21.641	4.10318	309	17.6	19.2
21.651	4.10129	360	19.0	19.2

21.661	4.09940	290	17.0	19.2
21.671	4.09752	321	17.9	19.2
21.681	4.09563	296	17.2	19.2
21.691	4.09375	320	17.9	19.2
21.702	4.09186	336	18.3	19.2
21.712	4.08998	377	19.4	19.2
21.722	4.08810	319	17.9	19.2
21.732	4.08623	321	17.9	19.2
21.742	4.08435	307	17.5	19.2
21.752	4.08248	347	18.6	19.2
21.762	4.08061	358	18.9	19.2
21.772	4.07874	337	18.4	19.2
21.782	4.07687	301	17.3	19.2
21.792	4.07500	326	18.1	19.2
21.803	4.07314	322	17.9	19.2
21.813	4.07127	335	18.3	19.2
21.823	4.06941	320	17.9	19.2
21.833	4.06755	353	18.8	19.2
21.843	4.06570	323	18.0	19.2
21.853	4.06384	336	18.3	19.2
21.863	4.06199	317	17.8	19.2
21.873	4.06013	329	18.1	19.2
21.883	4.05828	339	18.4	19.2
21.893	4.05643	332	18.2	19.2
21.904	4.05458	319	17.9	19.2
21.914	4.05274	296	17.2	19.2
21.924	4.05089	302	17.4	19.2
21.934	4.04905	317	17.8	19.2
21.944	4.04721	325	18.0	19.2
21.954	4.04537	304	17.4	19.2
21.964	4.04354	323	18.0	19.2
21.974	4.04170	341	18.5	19.2
21.984	4.03987	326	18.1	19.2
21.994	4.03803	307	17.5	19.2
22.005	4.03620	332	18.2	19.2
22.015	4.03437	327	18.1	19.2
22.025	4.03255	305	17.5	19.2
22.035	4.03072	304	17.4	19.2
22.045	4.02890	319	17.9	19.2
22.055	4.02708	357	18.9	19.2
22.065	4.02526	370	19.2	19.2
22.075	4.02344	353	18.8	19.2
22.085	4.02162	352	18.8	19.2
22.095	4.01980	317	17.8	19.2
22.106	4.01799	335	18.3	19.2
22.116	4.01618	313	17.7	19.2
22.126	4.01437	343	18.5	19.2
22.136	4.01256	326	18.1	19.2
22.146	4.01075	355	18.8	19.2
22.156	4.00895	321	17.9	19.2

22.166	4.00714	335	18.3	19.2
22.176	4.00534	337	18.4	19.2
22.186	4.00354	304	17.4	19.2
22.196	4.00174	344	18.5	19.2
22.206	3.99995	333	18.2	19.2
22.217	3.99815	355	18.8	19.2
22.227	3.99636	300	17.3	19.2
22.237	3.99456	342	18.5	19.2
22.247	3.99277	360	19.0	19.2
22.257	3.99098	347	18.6	19.2
22.267	3.98920	334	18.3	19.2
22.277	3.98741	320	17.9	19.2
22.287	3.98563	323	18.0	19.2
22.297	3.98385	382	19.5	19.2
22.307	3.98206	354	18.8	19.2
22.318	3.98029	388	19.7	19.2
22.328	3.97851	336	18.3	19.2
22.338	3.97673	312	17.7	19.2
22.348	3.97496	318	17.8	19.2
22.358	3.97319	333	18.2	19.2
22.368	3.97141	365	19.1	19.2
22.378	3.96964	349	18.7	19.2
22.388	3.96788	340	18.4	19.2
22.398	3.96611	358	18.9	19.2
22.408	3.96435	309	17.6	19.2
22.419	3.96258	321	17.9	19.2
22.429	3.96082	327	18.1	19.2
22.439	3.95906	349	18.7	19.2
22.449	3.95730	346	18.6	19.2
22.459	3.95555	364	19.1	19.2
22.469	3.95379	353	18.8	19.2
22.479	3.95204	322	17.9	19.2
22.489	3.95029	346	18.6	19.2
22.499	3.94854	361	19.0	19.2
22.509	3.94679	326	18.1	19.2
22.520	3.94504	325	18.0	19.2
22.530	3.94329	348	18.7	19.2
22.540	3.94155	338	18.4	19.2
22.550	3.93981	347	18.6	19.2
22.560	3.93807	353	18.8	19.2
22.570	3.93633	343	18.5	19.2
22.580	3.93459	342	18.5	19.2
22.590	3.93285	350	18.7	19.2
22.600	3.93112	331	18.2	19.2
22.610	3.92939	335	18.3	19.2
22.621	3.92765	309	17.6	19.2
22.631	3.92592	327	18.1	19.2
22.641	3.92420	326	18.1	19.2
22.651	3.92247	362	19.0	19.2
22.661	3.92074	361	19.0	19.2

22.671	3.91902	310	17.6	19.2
22.681	3.91730	341	18.5	19.2
22.691	3.91558	324	18.0	19.2
22.701	3.91386	325	18.0	19.2
22.711	3.91214	340	18.4	19.2
22.722	3.91042	342	18.5	19.2
22.732	3.90871	319	17.9	19.2
22.742	3.90700	327	18.1	19.2
22.752	3.90529	318	17.8	19.2
22.762	3.90358	365	19.1	19.2
22.772	3.90187	354	18.8	19.2
22.782	3.90016	349	18.7	19.2
22.792	3.89846	337	18.4	19.2
22.802	3.89675	355	18.8	19.2
22.812	3.89505	360	19.0	19.2
22.823	3.89335	315	17.7	19.2
22.833	3.89165	351	18.7	19.2
22.843	3.88995	340	18.4	19.2
22.853	3.88826	392	19.8	19.2
22.863	3.88656	356	18.9	19.2
22.873	3.88487	329	18.1	19.2
22.883	3.88318	386	19.6	19.2
22.893	3.88149	340	18.4	19.2
22.903	3.87980	335	18.3	19.2
22.913	3.87811	356	18.9	19.2
22.924	3.87642	357	18.9	19.2
22.934	3.87474	365	19.1	19.2
22.944	3.87306	376	19.4	19.2
22.954	3.87138	334	18.3	19.2
22.964	3.86970	402	20.0	19.2
22.974	3.86802	365	19.1	19.2
22.984	3.86634	350	18.7	19.2
22.994	3.86467	359	18.9	19.2
23.004	3.86299	366	19.1	19.2
23.014	3.86132	376	19.4	19.2
23.025	3.85965	343	18.5	19.2
23.035	3.85798	378	19.4	19.2
23.045	3.85631	332	18.2	19.2
23.055	3.85465	381	19.5	19.2
23.065	3.85298	332	18.2	19.2
23.075	3.85132	362	19.0	19.2
23.085	3.84965	340	18.4	19.2
23.095	3.84799	370	19.2	19.2
23.105	3.84633	374	19.3	19.2
23.115	3.84468	359	18.9	19.2
23.126	3.84302	322	17.9	19.2
23.136	3.84137	380	19.5	19.2
23.146	3.83971	352	18.8	19.2
23.156	3.83806	382	19.5	19.2
23.166	3.83641	351	18.7	19.2

23.176	3.83476	350	18.7	19.2
23.186	3.83311	380	19.5	19.2
23.196	3.83147	386	19.6	19.2
23.206	3.82982	359	18.9	19.2
23.216	3.82818	332	18.2	19.2
23.227	3.82654	357	18.9	19.2
23.237	3.82490	357	18.9	19.2
23.247	3.82326	369	19.2	19.2
23.257	3.82162	356	18.9	19.2
23.267	3.81999	356	18.9	19.2
23.277	3.81835	399	20.0	19.2
23.287	3.81672	368	19.2	19.2
23.297	3.81509	368	19.2	19.2
23.307	3.81346	355	18.8	19.2
23.317	3.81183	338	18.4	19.2
23.328	3.81020	329	18.1	19.2
23.338	3.80857	354	18.8	19.2
23.348	3.80695	342	18.5	19.2
23.358	3.80533	357	18.9	19.2
23.368	3.80371	355	18.8	19.2
23.378	3.80208	391	19.8	19.2
23.388	3.80047	328	18.1	19.2
23.398	3.79885	365	19.1	19.2
23.408	3.79723	352	18.8	19.2
23.418	3.79562	374	19.3	19.2
23.429	3.79400	327	18.1	19.2
23.439	3.79239	345	18.6	19.2
23.449	3.79078	363	19.1	19.2
23.459	3.78917	376	19.4	19.2
23.469	3.78756	330	18.2	19.2
23.479	3.78596	366	19.1	19.2
23.489	3.78435	373	19.3	19.2
23.499	3.78275	368	19.2	19.2
23.509	3.78115	377	19.4	19.2
23.519	3.77955	354	18.8	19.2
23.529	3.77795	377	19.4	19.2
23.540	3.77635	355	18.8	19.2
23.550	3.77475	399	20.0	19.2
23.560	3.77316	359	18.9	19.2
23.570	3.77156	367	19.2	19.2
23.580	3.76997	384	19.6	19.2
23.590	3.76838	352	18.8	19.2
23.600	3.76679	368	19.2	19.2
23.610	3.76520	367	19.2	19.2
23.620	3.76362	378	19.4	19.2
23.630	3.76203	355	18.8	19.2
23.641	3.76045	352	18.8	19.2
23.651	3.75886	373	19.3	19.2
23.661	3.75728	333	18.2	19.2
23.671	3.75570	359	18.9	19.2

23.681	3.75412	333	18.2	19.2
23.691	3.75254	364	19.1	19.2
23.701	3.75097	372	19.3	19.2
23.711	3.74939	370	19.2	19.2
23.721	3.74782	372	19.3	19.2
23.731	3.74625	378	19.4	19.2
23.742	3.74468	350	18.7	19.2
23.752	3.74311	390	19.7	19.2
23.762	3.74154	377	19.4	19.2
23.772	3.73997	351	18.7	19.2
23.782	3.73841	399	20.0	19.2
23.792	3.73684	363	19.1	19.2
23.802	3.73528	385	19.6	19.2
23.812	3.73372	361	19.0	19.2
23.822	3.73216	359	18.9	19.2
23.832	3.73060	382	19.5	19.2
23.843	3.72904	399	20.0	19.2
23.853	3.72749	392	19.8	19.2
23.863	3.72593	374	19.3	19.2
23.873	3.72438	381	19.5	19.2
23.883	3.72283	386	19.6	19.2
23.893	3.72128	374	19.3	19.2
23.903	3.71973	396	19.9	19.2
23.913	3.71818	393	19.8	19.2
23.923	3.71663	375	19.4	19.2
23.933	3.71509	367	19.2	19.2
23.944	3.71354	360	19.0	19.2
23.954	3.71200	390	19.7	19.2
23.964	3.71046	341	18.5	19.2
23.974	3.70892	353	18.8	19.2
23.984	3.70738	360	19.0	19.2
23.994	3.70584	362	19.0	19.2
24.004	3.70431	383	19.6	19.2
24.014	3.70277	385	19.6	19.2
24.024	3.70124	351	18.7	19.2
24.034	3.69970	359	18.9	19.2
24.045	3.69817	379	19.5	19.2
24.055	3.69664	378	19.4	19.2
24.065	3.69512	383	19.6	19.2
24.075	3.69359	383	19.6	19.2
24.085	3.69206	438	20.9	19.2
24.095	3.69054	381	19.5	19.2
24.105	3.68901	380	19.5	19.2
24.115	3.68749	363	19.1	19.2
24.125	3.68597	403	20.1	19.2
24.135	3.68445	364	19.1	19.2
24.146	3.68293	390	19.7	19.2
24.156	3.68142	359	18.9	19.2
24.166	3.67990	378	19.4	19.2
24.176	3.67839	399	20.0	19.2

24.186	3.67687	409	20.2	19.2
24.196	3.67536	376	19.4	19.2
24.206	3.67385	401	20.0	19.2
24.216	3.67234	350	18.7	19.2
24.226	3.67083	397	19.9	19.2
24.236	3.66933	399	20.0	19.2
24.247	3.66782	406	20.1	19.2
24.257	3.66632	356	18.9	19.2
24.267	3.66481	377	19.4	19.2
24.277	3.66331	370	19.2	19.2
24.287	3.66181	392	19.8	19.2
24.297	3.66031	387	19.7	19.2
24.307	3.65881	350	18.7	19.2
24.317	3.65732	419	20.5	19.2
24.327	3.65582	393	19.8	19.2
24.337	3.65433	381	19.5	19.2
24.348	3.65284	374	19.3	19.2
24.358	3.65134	412	20.3	19.2
24.368	3.64985	417	20.4	19.2
24.378	3.64836	414	20.3	19.2
24.388	3.64688	392	19.8	19.2
24.398	3.64539	421	20.5	19.2
24.408	3.64390	398	19.9	19.2
24.418	3.64242	365	19.1	19.2
24.428	3.64094	398	19.9	19.2
24.438	3.63946	417	20.4	19.2
24.449	3.63797	388	19.7	19.2
24.459	3.63650	420	20.5	19.2
24.469	3.63502	372	19.3	19.2
24.479	3.63354	402	20.0	19.2
24.489	3.63206	424	20.6	19.2
24.499	3.63059	366	19.1	19.2
24.509	3.62912	440	21.0	19.2
24.519	3.62765	413	20.3	19.2
24.529	3.62617	393	19.8	19.2
24.539	3.62471	373	19.3	19.2
24.550	3.62324	407	20.2	19.2
24.560	3.62177	437	20.9	19.2
24.570	3.62030	374	19.3	19.2
24.580	3.61884	439	21.0	19.2
24.590	3.61738	417	20.4	19.2
24.600	3.61591	416	20.4	19.2
24.610	3.61445	419	20.5	19.2
24.620	3.61299	397	19.9	19.2
24.630	3.61153	428	20.7	19.2
24.640	3.61008	382	19.5	19.2
24.651	3.60862	425	20.6	19.2
24.661	3.60717	369	19.2	19.2
24.671	3.60571	447	21.1	19.2
24.681	3.60426	393	19.8	19.2

24.691	3.60281	408	20.2	19.2
24.701	3.60136	430	20.7	19.2
24.711	3.59991	410	20.2	19.2
24.721	3.59846	415	20.4	19.2
24.731	3.59701	428	20.7	19.2
24.741	3.59557	426	20.6	19.2
24.752	3.59413	405	20.1	19.2
24.762	3.59268	380	19.5	19.2
24.772	3.59124	453	21.3	19.2
24.782	3.58980	405	20.1	19.2
24.792	3.58836	417	20.4	19.2
24.802	3.58692	416	20.4	19.2
24.812	3.58548	421	20.5	19.2
24.822	3.58405	454	21.3	19.2
24.832	3.58261	465	21.6	19.2
24.842	3.58118	430	20.7	19.2
24.852	3.57975	447	21.1	19.2
24.863	3.57832	432	20.8	19.2
24.873	3.57689	450	21.2	19.2
24.883	3.57546	406	20.1	19.2
24.893	3.57403	425	20.6	19.2
24.903	3.57260	417	20.4	19.2
24.913	3.57118	447	21.1	19.2
24.923	3.56975	444	21.1	19.2
24.933	3.56833	408	20.2	19.2
24.943	3.56691	436	20.9	19.2
24.953	3.56549	456	21.4	19.2
24.964	3.56407	437	20.9	19.2
24.974	3.56265	437	20.9	19.2
24.984	3.56123	428	20.7	19.2
24.994	3.55982	419	20.5	19.2
25.004	3.55840	434	20.8	19.2
25.014	3.55699	420	20.5	19.2
25.024	3.55558	425	20.6	19.2
25.034	3.55416	408	20.2	19.2
25.044	3.55275	413	20.3	19.2
25.054	3.55134	430	20.7	19.2
25.065	3.54994	471	21.7	19.2
25.075	3.54853	430	20.7	19.2
25.085	3.54712	449	21.2	19.2
25.095	3.54572	450	21.2	19.2
25.105	3.54432	450	21.2	19.2
25.115	3.54291	422	20.5	19.2
25.125	3.54151	457	21.4	19.2
25.135	3.54011	461	21.5	19.2
25.145	3.53871	427	20.7	19.2
25.155	3.53732	438	20.9	19.2
25.166	3.53592	461	21.5	19.2
25.176	3.53452	430	20.7	19.2
25.186	3.53313	476	21.8	19.2

25.196	3.53174	473	21.7	19.2
25.206	3.53034	461	21.5	19.2
25.216	3.52895	455	21.3	19.2
25.226	3.52756	488	22.1	19.2
25.236	3.52617	442	21.0	19.2
25.246	3.52479	451	21.2	19.2
25.256	3.52340	436	20.9	19.2
25.267	3.52201	430	20.7	19.2
25.277	3.52063	468	21.6	19.2
25.287	3.51925	452	21.3	19.2
25.297	3.51786	467	21.6	19.2
25.307	3.51648	439	21.0	19.2
25.317	3.51510	458	21.4	19.2
25.327	3.51372	459	21.4	19.2
25.337	3.51235	448	21.2	19.2
25.347	3.51097	454	21.3	19.2
25.357	3.50959	473	21.7	19.2
25.368	3.50822	430	20.7	19.2
25.378	3.50685	446	21.1	19.2
25.388	3.50548	446	21.1	19.2
25.398	3.50410	470	21.7	19.2
25.408	3.50273	444	21.1	19.2
25.418	3.50137	452	21.3	19.2
25.428	3.50000	472	21.7	19.2
25.438	3.49863	475	21.8	19.2
25.448	3.49727	473	21.7	19.2
25.458	3.49590	476	21.8	19.2
25.469	3.49454	524	22.9	19.2
25.479	3.49318	459	21.4	19.2
25.489	3.49181	454	21.3	19.2
25.499	3.49045	542	23.3	19.2
25.509	3.48909	512	22.6	19.2
25.519	3.48774	503	22.4	19.2
25.529	3.48638	501	22.4	19.2
25.539	3.48502	478	21.9	19.2
25.549	3.48367	464	21.5	19.2
25.559	3.48232	465	21.6	19.2
25.570	3.48096	490	22.1	19.2
25.580	3.47961	497	22.3	19.2
25.590	3.47826	489	22.1	19.2
25.600	3.47691	486	22.0	19.2
25.610	3.47556	505	22.5	19.2
25.620	3.47422	492	22.2	19.2
25.630	3.47287	503	22.4	19.2
25.640	3.47153	513	22.6	19.2
25.650	3.47018	532	23.1	19.2
25.660	3.46884	501	22.4	19.2
25.671	3.46750	530	23.0	19.2
25.681	3.46616	527	23.0	19.2
25.691	3.46482	513	22.6	19.2

25.701	3.46348	492	22.2	19.2
25.711	3.46214	541	23.3	19.2
25.721	3.46080	556	23.6	19.2
25.731	3.45947	525	22.9	19.2
25.741	3.45813	579	24.1	19.2
25.751	3.45680	535	23.1	19.2
25.761	3.45547	607	24.6	19.2
25.772	3.45414	552	23.5	19.2
25.782	3.45281	547	23.4	19.2
25.792	3.45148	528	23.0	19.2
25.802	3.45015	573	23.9	19.2
25.812	3.44882	535	23.1	19.2
25.822	3.44750	529	23.0	19.2
25.832	3.44617	546	23.4	19.2
25.842	3.44485	555	23.6	19.2
25.852	3.44353	538	23.2	19.2
25.862	3.44220	487	22.1	19.2
25.873	3.44088	520	22.8	19.2
25.883	3.43956	522	22.8	19.2
25.893	3.43824	505	22.5	19.2
25.903	3.43693	530	23.0	19.2
25.913	3.43561	494	22.2	19.2
25.923	3.43429	487	22.1	19.2
25.933	3.43298	518	22.8	19.2
25.943	3.43167	512	22.6	19.2
25.953	3.43035	465	21.6	19.2
25.963	3.42904	425	20.6	19.2
25.974	3.42773	498	22.3	19.2
25.984	3.42642	480	21.9	19.2
25.994	3.42511	487	22.1	19.2
26.004	3.42381	487	22.1	19.2
26.014	3.42250	496	22.3	19.2
26.024	3.42120	499	22.3	19.2
26.034	3.41989	527	23.0	19.2
26.044	3.41859	471	21.7	19.2
26.054	3.41729	479	21.9	19.2
26.064	3.41599	462	21.5	19.2
26.075	3.41468	493	22.2	19.2
26.085	3.41339	503	22.4	19.2
26.095	3.41209	499	22.3	19.2
26.105	3.41079	495	22.2	19.2
26.115	3.40949	517	22.7	19.2
26.125	3.40820	508	22.5	19.2
26.135	3.40691	484	22.0	19.2
26.145	3.40561	495	22.2	19.2
26.155	3.40432	525	22.9	19.2
26.165	3.40303	542	23.3	19.2
26.175	3.40174	490	22.1	19.2
26.186	3.40045	498	22.3	19.2
26.196	3.39916	500	22.4	19.2

26.206	3.39787	461	21.5	19.2
26.216	3.39659	503	22.4	19.2
26.226	3.39530	542	23.3	19.2
26.236	3.39402	499	22.3	19.2
26.246	3.39274	515	22.7	19.2
26.256	3.39145	498	22.3	19.2
26.266	3.39017	490	22.1	19.2
26.276	3.38889	508	22.5	19.2
26.287	3.38761	487	22.1	19.2
26.297	3.38634	520	22.8	19.2
26.307	3.38506	502	22.4	19.2
26.317	3.38378	506	22.5	19.2
26.327	3.38251	483	22.0	19.2
26.337	3.38123	494	22.2	19.2
26.347	3.37996	510	22.6	19.2
26.357	3.37869	521	22.8	19.2
26.367	3.37742	526	22.9	19.2
26.377	3.37615	549	23.4	19.2
26.388	3.37488	497	22.3	19.2
26.398	3.37361	532	23.1	19.2
26.408	3.37234	526	22.9	19.2
26.418	3.37108	541	23.3	19.2
26.428	3.36981	542	23.3	19.2
26.438	3.36855	547	23.4	19.2
26.448	3.36728	490	22.1	19.2
26.458	3.36602	548	23.4	19.2
26.468	3.36476	510	22.6	19.2
26.478	3.36350	492	22.2	19.2
26.489	3.36224	537	23.2	19.2
26.499	3.36098	529	23.0	19.2
26.509	3.35972	563	23.7	19.2
26.519	3.35847	487	22.1	19.2
26.529	3.35721	493	22.2	19.2
26.539	3.35596	512	22.6	19.2
26.549	3.35470	511	22.6	19.2
26.559	3.35345	525	22.9	19.2
26.569	3.35220	530	23.0	19.2
26.579	3.35095	527	23.0	19.2
26.590	3.34970	530	23.0	19.2
26.600	3.34845	491	22.2	19.2
26.610	3.34720	530	23.0	19.2
26.620	3.34595	544	23.3	19.2
26.630	3.34471	543	23.3	19.2
26.640	3.34346	517	22.7	19.2
26.650	3.34222	515	22.7	19.2
26.660	3.34097	554	23.5	19.2
26.670	3.33973	540	23.2	19.2
26.680	3.33849	519	22.8	19.2
26.691	3.33725	543	23.3	19.2
26.701	3.33601	536	23.2	19.2

26.711	3.33477	564	23.7	19.2
26.721	3.33354	542	23.3	19.2
26.731	3.33230	587	24.2	19.2
26.741	3.33106	539	23.2	19.2
26.751	3.32983	545	23.3	19.2
26.761	3.32860	560	23.7	19.2
26.771	3.32736	535	23.1	19.2
26.781	3.32613	512	22.6	19.2
26.792	3.32490	554	23.5	19.2
26.802	3.32367	552	23.5	19.2
26.812	3.32244	566	23.8	19.2
26.822	3.32121	518	22.8	19.2
26.832	3.31999	502	22.4	19.2
26.842	3.31876	556	23.6	19.2
26.852	3.31753	565	23.8	19.2
26.862	3.31631	528	23.0	19.2
26.872	3.31509	536	23.2	19.2
26.882	3.31386	529	23.0	19.2
26.893	3.31264	579	24.1	19.2
26.903	3.31142	570	23.9	19.2
26.913	3.31020	574	24.0	19.2
26.923	3.30898	585	24.2	19.2
26.933	3.30777	592	24.3	19.2
26.943	3.30655	557	23.6	19.2
26.953	3.30533	497	22.3	19.2
26.963	3.30412	540	23.2	19.2
26.973	3.30290	532	23.1	19.2
26.983	3.30169	544	23.3	19.2
26.994	3.30048	549	23.4	19.2
27.004	3.29927	581	24.1	19.2
27.014	3.29806	533	23.1	19.2
27.024	3.29685	508	22.5	19.2
27.034	3.29564	599	24.5	19.2
27.044	3.29443	544	23.3	19.2
27.054	3.29322	577	24.0	19.2
27.064	3.29202	565	23.8	19.2
27.074	3.29081	576	24.0	19.2
27.084	3.28961	531	23.0	19.2
27.095	3.28840	556	23.6	19.2
27.105	3.28720	502	22.4	19.2
27.115	3.28600	547	23.4	19.2
27.125	3.28480	560	23.7	19.2
27.135	3.28360	564	23.7	19.2
27.145	3.28240	578	24.0	19.2
27.155	3.28120	545	23.3	19.2
27.165	3.28001	532	23.1	19.2
27.175	3.27881	549	23.4	19.2
27.185	3.27762	567	23.8	19.2
27.196	3.27642	572	23.9	19.2
27.206	3.27523	588	24.2	19.2

27.216	3.27404	516	22.7	19.2
27.226	3.27284	543	23.3	19.2
27.236	3.27165	611	24.7	19.2
27.246	3.27046	575	24.0	19.2
27.256	3.26927	561	23.7	19.2
27.266	3.26809	634	25.2	19.2
27.276	3.26690	567	23.8	19.2
27.286	3.26571	535	23.1	19.2
27.297	3.26453	583	24.1	19.2
27.307	3.26334	593	24.4	19.2
27.317	3.26216	591	24.3	19.2
27.327	3.26098	584	24.2	19.2
27.337	3.25980	589	24.3	19.2
27.347	3.25861	539	23.2	19.2
27.357	3.25743	549	23.4	19.2
27.367	3.25626	569	23.9	19.2
27.377	3.25508	565	23.8	19.2
27.387	3.25390	503	22.4	19.2
27.398	3.25272	563	23.7	19.2
27.408	3.25155	578	24.0	19.2
27.418	3.25037	579	24.1	19.2
27.428	3.24920	577	24.0	19.2
27.438	3.24803	549	23.4	19.2
27.448	3.24685	546	23.4	19.2
27.458	3.24568	627	25.0	19.2
27.468	3.24451	604	24.6	19.2
27.478	3.24334	501	22.4	19.2
27.488	3.24217	536	23.2	19.2
27.498	3.24101	557	23.6	19.2
27.509	3.23984	590	24.3	19.2
27.519	3.23867	551	23.5	19.2
27.529	3.23751	582	24.1	19.2
27.539	3.23634	589	24.3	19.2
27.549	3.23518	581	24.1	19.2
27.559	3.23402	559	23.6	19.2
27.569	3.23286	562	23.7	19.2
27.579	3.23169	583	24.1	19.2
27.589	3.23053	609	24.7	19.2
27.599	3.22938	561	23.7	19.2
27.610	3.22822	554	23.5	19.2
27.620	3.22706	599	24.5	19.2
27.630	3.22590	588	24.2	19.2
27.640	3.22475	569	23.9	19.2
27.650	3.22359	616	24.8	19.2
27.660	3.22244	629	25.1	19.2
27.670	3.22128	593	24.4	19.2
27.680	3.22013	576	24.0	19.2
27.690	3.21898	562	23.7	19.2
27.700	3.21783	525	22.9	19.2
27.711	3.21668	622	24.9	19.2

27.721	3.21553	551	23.5	19.2
27.731	3.21438	586	24.2	19.2
27.741	3.21324	612	24.7	19.2
27.751	3.21209	629	25.1	19.2
27.761	3.21094	612	24.7	19.2
27.771	3.20980	573	23.9	19.2
27.781	3.20866	625	25.0	19.2
27.791	3.20751	612	24.7	19.2
27.801	3.20637	580	24.1	19.2
27.812	3.20523	598	24.5	19.2
27.822	3.20409	546	23.4	19.2
27.832	3.20295	594	24.4	19.2
27.842	3.20181	566	23.8	19.2
27.852	3.20067	586	24.2	19.2
27.862	3.19953	598	24.5	19.2
27.872	3.19840	540	23.2	19.2
27.882	3.19726	595	24.4	19.2
27.892	3.19613	611	24.7	19.2
27.902	3.19499	581	24.1	19.2
27.913	3.19386	585	24.2	19.2
27.923	3.19273	579	24.1	19.2
27.933	3.19160	587	24.2	19.2
27.943	3.19047	582	24.1	19.2
27.953	3.18934	591	24.3	19.2
27.963	3.18821	588	24.2	19.2
27.973	3.18708	585	24.2	19.2
27.983	3.18595	546	23.4	19.2
27.993	3.18483	625	25.0	19.2
28.003	3.18370	598	24.5	19.2
28.014	3.18258	626	25.0	19.2
28.024	3.18145	581	24.1	19.2
28.034	3.18033	584	24.2	19.2
28.044	3.17921	602	24.5	19.2
28.054	3.17808	596	24.4	19.2
28.064	3.17696	591	24.3	19.2
28.074	3.17584	589	24.3	19.2
28.084	3.17472	573	23.9	19.2
28.094	3.17361	591	24.3	19.2
28.104	3.17249	619	24.9	19.2
28.115	3.17137	593	24.4	19.2
28.125	3.17026	588	24.2	19.2
28.135	3.16914	597	24.4	19.2
28.145	3.16803	571	23.9	19.2
28.155	3.16691	625	25.0	19.2
28.165	3.16580	647	25.4	19.2
28.175	3.16469	572	23.9	19.2
28.185	3.16358	642	25.3	19.2
28.195	3.16247	610	24.7	19.2
28.205	3.16136	611	24.7	19.2
28.216	3.16025	637	25.2	19.2

28.226	3.15914	637	25.2	19.2
28.236	3.15804	598	24.5	19.2
28.246	3.15693	622	24.9	19.2
28.256	3.15582	650	25.5	19.2
28.266	3.15472	591	24.3	19.2
28.276	3.15362	586	24.2	19.2
28.286	3.15251	619	24.9	19.2
28.296	3.15141	599	24.5	19.2
28.306	3.15031	605	24.6	19.2
28.317	3.14921	605	24.6	19.2
28.327	3.14811	646	25.4	19.2
28.337	3.14701	605	24.6	19.2
28.347	3.14591	626	25.0	19.2
28.357	3.14481	606	24.6	19.2
28.367	3.14372	638	25.3	19.2
28.377	3.14262	644	25.4	19.2
28.387	3.14153	624	25.0	19.2
28.397	3.14043	613	24.8	19.2
28.407	3.13934	677	26.0	19.2
28.418	3.13825	606	24.6	19.2
28.428	3.13715	605	24.6	19.2
28.438	3.13606	605	24.6	19.2
28.448	3.13497	566	23.8	19.2
28.458	3.13388	570	23.9	19.2
28.468	3.13279	642	25.3	19.2
28.478	3.13171	609	24.7	19.2
28.488	3.13062	633	25.2	19.2
28.498	3.12953	633	25.2	19.2
28.508	3.12845	594	24.4	19.2
28.519	3.12736	658	25.7	19.2
28.529	3.12628	614	24.8	19.2
28.539	3.12519	667	25.8	19.2
28.549	3.12411	632	25.1	19.2
28.559	3.12303	660	25.7	19.2
28.569	3.12195	610	24.7	19.2
28.579	3.12087	640	25.3	19.2
28.589	3.11979	615	24.8	19.2
28.599	3.11871	651	25.5	19.2
28.609	3.11763	581	24.1	19.2
28.620	3.11655	591	24.3	19.2
28.630	3.11548	598	24.5	19.2
28.640	3.11440	656	25.6	19.2
28.650	3.11333	644	25.4	19.2
28.660	3.11225	622	24.9	19.2
28.670	3.11118	618	24.9	19.2
28.680	3.11011	586	24.2	19.2
28.690	3.10904	595	24.4	19.2
28.700	3.10796	597	24.4	19.2
28.710	3.10689	625	25.0	19.2
28.721	3.10582	621	24.9	19.2

28.731	3.10476	610	24.7	19.2
28.741	3.10369	651	25.5	19.2
28.751	3.10262	627	25.0	19.2
28.761	3.10155	636	25.2	19.2
28.771	3.10049	628	25.1	19.2
28.781	3.09942	633	25.2	19.2
28.791	3.09836	614	24.8	19.2
28.801	3.09730	561	23.7	19.2
28.811	3.09623	611	24.7	19.2
28.821	3.09517	622	24.9	19.2
28.832	3.09411	614	24.8	19.2
28.842	3.09305	622	24.9	19.2
28.852	3.09199	574	24.0	19.2
28.862	3.09093	627	25.0	19.2
28.872	3.08987	598	24.5	19.2
28.882	3.08881	599	24.5	19.2
28.892	3.08776	615	24.8	19.2
28.902	3.08670	605	24.6	19.2
28.912	3.08565	612	24.7	19.2
28.922	3.08459	618	24.9	19.2
28.933	3.08354	581	24.1	19.2
28.943	3.08249	624	25.0	19.2
28.953	3.08143	698	26.4	19.2
28.963	3.08038	626	25.0	19.2
28.973	3.07933	635	25.2	19.2
28.983	3.07828	696	26.4	19.2
28.993	3.07723	615	24.8	19.2
29.003	3.07618	620	24.9	19.2
29.013	3.07514	600	24.5	19.2
29.023	3.07409	618	24.9	19.2
29.034	3.07304	596	24.4	19.2
29.044	3.07200	685	26.2	19.2
29.054	3.07095	586	24.2	19.2
29.064	3.06991	608	24.7	19.2
29.074	3.06886	622	24.9	19.2
29.084	3.06782	575	24.0	19.2
29.094	3.06678	636	25.2	19.2
29.104	3.06574	638	25.3	19.2
29.114	3.06470	676	26.0	19.2
29.124	3.06366	595	24.4	19.2
29.135	3.06262	698	26.4	19.2
29.145	3.06158	649	25.5	19.2
29.155	3.06054	609	24.7	19.2
29.165	3.05951	650	25.5	19.2
29.175	3.05847	647	25.4	19.2
29.185	3.05743	641	25.3	19.2
29.195	3.05640	672	25.9	19.2
29.205	3.05537	601	24.5	19.2
29.215	3.05433	586	24.2	19.2
29.225	3.05330	612	24.7	19.2

29.236	3.05227	637	25.2	19.2
29.246	3.05124	671	25.9	19.2
29.256	3.05021	627	25.0	19.2
29.266	3.04918	603	24.6	19.2
29.276	3.04815	608	24.7	19.2
29.286	3.04712	604	24.6	19.2
29.296	3.04609	640	25.3	19.2
29.306	3.04507	640	25.3	19.2
29.316	3.04404	593	24.4	19.2
29.326	3.04301	557	23.6	19.2
29.337	3.04199	616	24.8	19.2
29.347	3.04097	686	26.2	19.2
29.357	3.03994	603	24.6	19.2
29.367	3.03892	647	25.4	19.2
29.377	3.03790	635	25.2	19.2
29.387	3.03688	645	25.4	19.2
29.397	3.03586	664	25.8	19.2
29.407	3.03484	646	25.4	19.2
29.417	3.03382	610	24.7	19.2
29.427	3.03280	625	25.0	19.2
29.438	3.03178	641	25.3	19.2
29.448	3.03077	625	25.0	19.2
29.458	3.02975	582	24.1	19.2
29.468	3.02874	609	24.7	19.2
29.478	3.02772	644	25.4	19.2
29.488	3.02671	634	25.2	19.2
29.498	3.02569	623	25.0	19.2
29.508	3.02468	651	25.5	19.2
29.518	3.02367	631	25.1	19.2
29.528	3.02266	616	24.8	19.2
29.539	3.02165	614	24.8	19.2
29.549	3.02064	653	25.6	19.2
29.559	3.01963	617	24.8	19.2
29.569	3.01862	645	25.4	19.2
29.579	3.01761	627	25.0	19.2
29.589	3.01661	611	24.7	19.2
29.599	3.01560	612	24.7	19.2
29.609	3.01459	644	25.4	19.2
29.619	3.01359	644	25.4	19.2
29.629	3.01258	602	24.5	19.2
29.640	3.01158	644	25.4	19.2
29.650	3.01058	611	24.7	19.2
29.660	3.00958	614	24.8	19.2
29.670	3.00857	654	25.6	19.2
29.680	3.00757	626	25.0	19.2
29.690	3.00657	625	25.0	19.2
29.700	3.00557	588	24.2	19.2
29.710	3.00458	613	24.8	19.2
29.720	3.00358	646	25.4	19.2
29.730	3.00258	616	24.8	19.2

29.741	3.00158	639	25.3	19.2
29.751	3.00059	587	24.2	19.2
29.761	2.99959	646	25.4	19.2
29.771	2.99860	618	24.9	19.2
29.781	2.99760	627	25.0	19.2
29.791	2.99661	672	25.9	19.2
29.801	2.99562	667	25.8	19.2
29.811	2.99463	626	25.0	19.2
29.821	2.99364	624	25.0	19.2
29.831	2.99264	606	24.6	19.2
29.842	2.99166	645	25.4	19.2
29.852	2.99067	566	23.8	19.2
29.862	2.98968	636	25.2	19.2
29.872	2.98869	679	26.1	19.2
29.882	2.98770	625	25.0	19.2
29.892	2.98672	647	25.4	19.2
29.902	2.98573	637	25.2	19.2
29.912	2.98475	639	25.3	19.2
29.922	2.98376	621	24.9	19.2
29.932	2.98278	594	24.4	19.2
29.943	2.98179	614	24.8	19.2
29.953	2.98081	657	25.6	19.2
29.963	2.97983	623	25.0	19.2
29.973	2.97885	647	25.4	19.2
29.983	2.97787	564	23.7	19.2
29.993	2.97689	618	24.9	19.2
30.003	2.97591	610	24.7	19.2
30.013	2.97493	603	24.6	19.2
30.023	2.97395	635	25.2	19.2
30.033	2.97298	644	25.4	19.2
30.044	2.97200	601	24.5	19.2
30.054	2.97102	638	25.3	19.2
30.064	2.97005	643	25.4	19.2
30.074	2.96908	623	25.0	19.2
30.084	2.96810	656	25.6	19.2
30.094	2.96713	603	24.6	19.2
30.104	2.96616	607	24.6	19.2
30.114	2.96518	631	25.1	19.2
30.124	2.96421	626	25.0	19.2
30.134	2.96324	595	24.4	19.2
30.144	2.96227	619	24.9	19.2
30.155	2.96130	617	24.8	19.2
30.165	2.96034	598	24.5	19.2
30.175	2.95937	639	25.3	19.2
30.185	2.95840	618	24.9	19.2
30.195	2.95743	654	25.6	19.2
30.205	2.95647	610	24.7	19.2
30.215	2.95550	637	25.2	19.2
30.225	2.95454	653	25.6	19.2
30.235	2.95357	629	25.1	19.2

30.245	2.95261	624	25.0	19.2
30.256	2.95165	647	25.4	19.2
30.266	2.95069	641	25.3	19.2
30.276	2.94973	611	24.7	19.2
30.286	2.94876	598	24.5	19.2
30.296	2.94780	652	25.5	19.2
30.306	2.94685	623	25.0	19.2
30.316	2.94589	603	24.6	19.2
30.326	2.94493	622	24.9	19.2
30.336	2.94397	641	25.3	19.2
30.346	2.94301	603	24.6	19.2
30.357	2.94206	612	24.7	19.2
30.367	2.94110	621	24.9	19.2
30.377	2.94015	610	24.7	19.2
30.387	2.93919	651	25.5	19.2
30.397	2.93824	640	25.3	19.2
30.407	2.93729	623	25.0	19.2
30.417	2.93634	620	24.9	19.2
30.427	2.93538	603	24.6	19.2
30.437	2.93443	687	26.2	19.2
30.447	2.93348	562	23.7	19.2
30.458	2.93253	625	25.0	19.2
30.468	2.93158	613	24.8	19.2
30.478	2.93064	629	25.1	19.2
30.488	2.92969	604	24.6	19.2
30.498	2.92874	586	24.2	19.2
30.508	2.92779	636	25.2	19.2
30.518	2.92685	575	24.0	19.2
30.528	2.92590	599	24.5	19.2
30.538	2.92496	591	24.3	19.2
30.548	2.92401	602	24.5	19.2
30.559	2.92307	651	25.5	19.2
30.569	2.92213	613	24.8	19.2
30.579	2.92119	569	23.9	19.2
30.589	2.92024	618	24.9	19.2
30.599	2.91930	612	24.7	19.2
30.609	2.91836	630	25.1	19.2
30.619	2.91742	587	24.2	19.2
30.629	2.91649	597	24.4	19.2
30.639	2.91555	602	24.5	19.2
30.649	2.91461	599	24.5	19.2
30.660	2.91367	600	24.5	19.2
30.670	2.91274	599	24.5	19.2
30.680	2.91180	614	24.8	19.2
30.690	2.91086	620	24.9	19.2
30.700	2.90993	644	25.4	19.2
30.710	2.90900	613	24.8	19.2
30.720	2.90806	579	24.1	19.2
30.730	2.90713	590	24.3	19.2
30.740	2.90620	585	24.2	19.2

30.750	2.90527	584	24.2	19.2
30.761	2.90434	673	25.9	19.2
30.771	2.90341	656	25.6	19.2
30.781	2.90248	584	24.2	19.2
30.791	2.90155	584	24.2	19.2
30.801	2.90062	630	25.1	19.2
30.811	2.89969	665	25.8	19.2
30.821	2.89876	654	25.6	19.2
30.831	2.89784	576	24.0	19.2
30.841	2.89691	570	23.9	19.2
30.851	2.89599	581	24.1	19.2
30.862	2.89506	655	25.6	19.2
30.872	2.89414	641	25.3	19.2
30.882	2.89321	612	24.7	19.2
30.892	2.89229	587	24.2	19.2
30.902	2.89137	625	25.0	19.2
30.912	2.89045	586	24.2	19.2
30.922	2.88953	604	24.6	19.2
30.932	2.88861	578	24.0	19.2
30.942	2.88769	610	24.7	19.2
30.952	2.88677	612	24.7	19.2
30.963	2.88585	638	25.3	19.2
30.973	2.88493	576	24.0	19.2
30.983	2.88401	620	24.9	19.2
30.993	2.88310	596	24.4	19.2
31.003	2.88218	598	24.5	19.2
31.013	2.88126	601	24.5	19.2
31.023	2.88035	639	25.3	19.2
31.033	2.87943	588	24.2	19.2
31.043	2.87852	592	24.3	19.2
31.053	2.87761	584	24.2	19.2
31.064	2.87670	585	24.2	19.2
31.074	2.87578	579	24.1	19.2
31.084	2.87487	621	24.9	19.2
31.094	2.87396	650	25.5	19.2
31.104	2.87305	646	25.4	19.2
31.114	2.87214	648	25.5	19.2
31.124	2.87123	585	24.2	19.2
31.134	2.87032	630	25.1	19.2
31.144	2.86942	610	24.7	19.2
31.154	2.86851	637	25.2	19.2
31.165	2.86760	628	25.1	19.2
31.175	2.86670	642	25.3	19.2
31.185	2.86579	563	23.7	19.2
31.195	2.86489	601	24.5	19.2
31.205	2.86398	634	25.2	19.2
31.215	2.86308	589	24.3	19.2
31.225	2.86218	594	24.4	19.2
31.235	2.86127	584	24.2	19.2
31.245	2.86037	591	24.3	19.2

31.255	2.85947	574	24.0	19.2
31.266	2.85857	576	24.0	19.2
31.276	2.85767	596	24.4	19.2
31.286	2.85677	603	24.6	19.2
31.296	2.85587	573	23.9	19.2
31.306	2.85497	586	24.2	19.2
31.316	2.85408	601	24.5	19.2
31.326	2.85318	614	24.8	19.2
31.336	2.85228	590	24.3	19.2
31.346	2.85139	619	24.9	19.2
31.356	2.85049	588	24.2	19.2
31.366	2.84960	582	24.1	19.2
31.377	2.84870	590	24.3	19.2
31.387	2.84781	586	24.2	19.2
31.397	2.84692	606	24.6	19.2
31.407	2.84602	615	24.8	19.2
31.417	2.84513	542	23.3	19.2
31.427	2.84424	628	25.1	19.2
31.437	2.84335	579	24.1	19.2
31.447	2.84246	603	24.6	19.2
31.457	2.84157	562	23.7	19.2
31.467	2.84068	578	24.0	19.2
31.478	2.83979	628	25.1	19.2
31.488	2.83890	621	24.9	19.2
31.498	2.83802	600	24.5	19.2
31.508	2.83713	610	24.7	19.2
31.518	2.83624	569	23.9	19.2
31.528	2.83536	540	23.2	19.2
31.538	2.83447	600	24.5	19.2
31.548	2.83359	620	24.9	19.2
31.558	2.83271	575	24.0	19.2
31.568	2.83182	596	24.4	19.2
31.579	2.83094	588	24.2	19.2
31.589	2.83006	610	24.7	19.2
31.599	2.82918	583	24.1	19.2
31.609	2.82830	606	24.6	19.2
31.619	2.82742	589	24.3	19.2
31.629	2.82654	615	24.8	19.2
31.639	2.82566	564	23.7	19.2
31.649	2.82478	563	23.7	19.2
31.659	2.82390	632	25.1	19.2
31.669	2.82302	569	23.9	19.2
31.680	2.82215	572	23.9	19.2
31.690	2.82127	585	24.2	19.2
31.700	2.82039	567	23.8	19.2
31.710	2.81952	571	23.9	19.2
31.720	2.81864	571	23.9	19.2
31.730	2.81777	571	23.9	19.2
31.740	2.81690	583	24.1	19.2
31.750	2.81602	607	24.6	19.2

31.760	2.81515	550	23.5	19.2
31.770	2.81428	554	23.5	19.2
31.781	2.81341	595	24.4	19.2
31.791	2.81254	579	24.1	19.2
31.801	2.81167	596	24.4	19.2
31.811	2.81080	572	23.9	19.2
31.821	2.80993	546	23.4	19.2
31.831	2.80906	592	24.3	19.2
31.841	2.80819	569	23.9	19.2
31.851	2.80732	575	24.0	19.2
31.861	2.80646	596	24.4	19.2
31.871	2.80559	532	23.1	19.2
31.882	2.80473	589	24.3	19.2
31.892	2.80386	604	24.6	19.2
31.902	2.80300	550	23.5	19.2
31.912	2.80213	586	24.2	19.2
31.922	2.80127	592	24.3	19.2
31.932	2.80040	608	24.7	19.2
31.942	2.79954	614	24.8	19.2
31.952	2.79868	571	23.9	19.2
31.962	2.79782	544	23.3	19.2
31.972	2.79696	583	24.1	19.2
31.983	2.79610	568	23.8	19.2
31.993	2.79524	561	23.7	19.2
32.003	2.79438	602	24.5	19.2
32.013	2.79352	579	24.1	19.2
32.023	2.79266	559	23.6	19.2
32.033	2.79181	574	24.0	19.2
32.043	2.79095	553	23.5	19.2
32.053	2.79009	565	23.8	19.2
32.063	2.78924	549	23.4	19.2
32.073	2.78838	561	23.7	19.2
32.084	2.78753	554	23.5	19.2
32.094	2.78667	562	23.7	19.2
32.104	2.78582	564	23.7	19.2
32.114	2.78497	589	24.3	19.2
32.124	2.78411	572	23.9	19.2
32.134	2.78326	564	23.7	19.2
32.144	2.78241	534	23.1	19.2
32.154	2.78156	556	23.6	19.2
32.164	2.78071	566	23.8	19.2
32.174	2.77986	539	23.2	19.2
32.185	2.77901	596	24.4	19.2
32.195	2.77816	590	24.3	19.2
32.205	2.77731	601	24.5	19.2
32.215	2.77647	543	23.3	19.2
32.225	2.77562	548	23.4	19.2
32.235	2.77477	574	24.0	19.2
32.245	2.77393	592	24.3	19.2
32.255	2.77308	571	23.9	19.2

32.265	2.77224	609	24.7	19.2
32.275	2.77139	543	23.3	19.2
32.286	2.77055	625	25.0	19.2
32.296	2.76970	586	24.2	19.2
32.306	2.76886	567	23.8	19.2
32.316	2.76802	619	24.9	19.2
32.326	2.76718	567	23.8	19.2
32.336	2.76634	563	23.7	19.2
32.346	2.76550	580	24.1	19.2
32.356	2.76466	573	23.9	19.2
32.366	2.76382	571	23.9	19.2
32.376	2.76298	587	24.2	19.2
32.387	2.76214	569	23.9	19.2
32.397	2.76130	542	23.3	19.2
32.407	2.76046	550	23.5	19.2
32.417	2.75963	572	23.9	19.2
32.427	2.75879	560	23.7	19.2
32.437	2.75795	570	23.9	19.2
32.447	2.75712	556	23.6	19.2
32.457	2.75628	580	24.1	19.2
32.467	2.75545	555	23.6	19.2
32.477	2.75462	569	23.9	19.2
32.488	2.75378	551	23.5	19.2
32.498	2.75295	546	23.4	19.2
32.508	2.75212	549	23.4	19.2
32.518	2.75129	547	23.4	19.2
32.528	2.75045	569	23.9	19.2
32.538	2.74962	568	23.8	19.2
32.548	2.74879	531	23.0	19.2
32.558	2.74796	524	22.9	19.2
32.568	2.74713	579	24.1	19.2
32.578	2.74631	575	24.0	19.2
32.589	2.74548	632	25.1	19.2
32.599	2.74465	558	23.6	19.2
32.609	2.74382	530	23.0	19.2
32.619	2.74300	548	23.4	19.2
32.629	2.74217	545	23.3	19.2
32.639	2.74135	547	23.4	19.2
32.649	2.74052	555	23.6	19.2
32.659	2.73970	480	21.9	19.2
32.669	2.73887	564	23.7	19.2
32.679	2.73805	541	23.3	19.2
32.689	2.73723	553	23.5	19.2
32.700	2.73640	528	23.0	19.2
32.710	2.73558	548	23.4	19.2
32.720	2.73476	533	23.1	19.2
32.730	2.73394	521	22.8	19.2
32.740	2.73312	596	24.4	19.2
32.750	2.73230	531	23.0	19.2
32.760	2.73148	533	23.1	19.2

32.770	2.73066	536	23.2	19.2
32.780	2.72984	575	24.0	19.2
32.790	2.72903	597	24.4	19.2
32.801	2.72821	505	22.5	19.2
32.811	2.72739	548	23.4	19.2
32.821	2.72658	528	23.0	19.2
32.831	2.72576	577	24.0	19.2
32.841	2.72495	549	23.4	19.2
32.851	2.72413	573	23.9	19.2
32.861	2.72332	536	23.2	19.2
32.871	2.72250	595	24.4	19.2
32.881	2.72169	517	22.7	19.2
32.891	2.72088	588	24.2	19.2
32.902	2.72007	544	23.3	19.2
32.912	2.71925	582	24.1	19.2
32.922	2.71844	622	24.9	19.2
32.932	2.71763	563	23.7	19.2
32.942	2.71682	532	23.1	19.2
32.952	2.71601	547	23.4	19.2
32.962	2.71520	548	23.4	19.2
32.972	2.71439	565	23.8	19.2
32.982	2.71359	556	23.6	19.2
32.992	2.71278	579	24.1	19.2
33.003	2.71197	613	24.8	19.2
33.013	2.71117	583	24.1	19.2
33.023	2.71036	592	24.3	19.2
33.033	2.70955	562	23.7	19.2
33.043	2.70875	587	24.2	19.2
33.053	2.70794	609	24.7	19.2
33.063	2.70714	602	24.5	19.2
33.073	2.70634	596	24.4	19.2
33.083	2.70553	591	24.3	19.2
33.093	2.70473	579	24.1	19.2
33.104	2.70393	587	24.2	19.2
33.114	2.70313	576	24.0	19.2
33.124	2.70233	583	24.1	19.2
33.134	2.70153	567	23.8	19.2
33.144	2.70072	571	23.9	19.2
33.154	2.69993	569	23.9	19.2
33.164	2.69913	555	23.6	19.2
33.174	2.69833	553	23.5	19.2
33.184	2.69753	559	23.6	19.2
33.194	2.69673	570	23.9	19.2
33.205	2.69593	516	22.7	19.2
33.215	2.69514	557	23.6	19.2
33.225	2.69434	558	23.6	19.2
33.235	2.69355	548	23.4	19.2
33.245	2.69275	572	23.9	19.2
33.255	2.69196	510	22.6	19.2
33.265	2.69116	526	22.9	19.2

33.275	2.69037	528	23.0	19.2
33.285	2.68958	537	23.2	19.2
33.295	2.68878	518	22.8	19.2
33.306	2.68799	541	23.3	19.2
33.316	2.68720	596	24.4	19.2
33.326	2.68641	500	22.4	19.2
33.336	2.68562	505	22.5	19.2
33.346	2.68483	550	23.5	19.2
33.356	2.68404	485	22.0	19.2
33.366	2.68325	532	23.1	19.2
33.376	2.68246	489	22.1	19.2
33.386	2.68167	531	23.0	19.2
33.396	2.68088	544	23.3	19.2
33.407	2.68009	541	23.3	19.2
33.417	2.67931	539	23.2	19.2
33.427	2.67852	542	23.3	19.2
33.437	2.67774	518	22.8	19.2
33.447	2.67695	546	23.4	19.2
33.457	2.67616	507	22.5	19.2
33.467	2.67538	518	22.8	19.2
33.477	2.67460	534	23.1	19.2
33.487	2.67381	553	23.5	19.2
33.497	2.67303	547	23.4	19.2
33.508	2.67225	500	22.4	19.2
33.518	2.67147	500	22.4	19.2
33.528	2.67068	532	23.1	19.2
33.538	2.66990	517	22.7	19.2
33.548	2.66912	561	23.7	19.2
33.558	2.66834	512	22.6	19.2
33.568	2.66756	566	23.8	19.2
33.578	2.66678	514	22.7	19.2
33.588	2.66600	485	22.0	19.2
33.598	2.66523	518	22.8	19.2
33.609	2.66445	512	22.6	19.2
33.619	2.66367	510	22.6	19.2
33.629	2.66289	513	22.6	19.2
33.639	2.66212	473	21.7	19.2
33.649	2.66134	513	22.6	19.2
33.659	2.66057	502	22.4	19.2
33.669	2.65979	489	22.1	19.2
33.679	2.65902	530	23.0	19.2
33.689	2.65824	552	23.5	19.2
33.699	2.65747	503	22.4	19.2
33.710	2.65670	513	22.6	19.2
33.720	2.65592	465	21.6	19.2
33.730	2.65515	489	22.1	19.2
33.740	2.65438	496	22.3	19.2
33.750	2.65361	511	22.6	19.2
33.760	2.65284	516	22.7	19.2
33.770	2.65207	502	22.4	19.2

33.780	2.65130	512	22.6	19.2
33.790	2.65053	460	21.4	19.2
33.800	2.64976	493	22.2	19.2
33.811	2.64899	516	22.7	19.2
33.821	2.64822	524	22.9	19.2
33.831	2.64746	524	22.9	19.2
33.841	2.64669	520	22.8	19.2
33.851	2.64592	506	22.5	19.2
33.861	2.64516	488	22.1	19.2
33.871	2.64439	485	22.0	19.2
33.881	2.64363	458	21.4	19.2
33.891	2.64286	520	22.8	19.2
33.901	2.64210	520	22.8	19.2
33.912	2.64133	477	21.8	19.2
33.922	2.64057	497	22.3	19.2
33.932	2.63981	481	21.9	19.2
33.942	2.63904	495	22.2	19.2
33.952	2.63828	480	21.9	19.2
33.962	2.63752	530	23.0	19.2
33.972	2.63676	468	21.6	19.2
33.982	2.63600	474	21.8	19.2
33.992	2.63524	512	22.6	19.2
34.002	2.63448	484	22.0	19.2
34.012	2.63372	484	22.0	19.2
34.023	2.63296	506	22.5	19.2
34.033	2.63220	488	22.1	19.2
34.043	2.63145	512	22.6	19.2
34.053	2.63069	484	22.0	19.2
34.063	2.62993	498	22.3	19.2
34.073	2.62917	455	21.3	19.2
34.083	2.62842	490	22.1	19.2
34.093	2.62766	509	22.6	19.2
34.103	2.62691	492	22.2	19.2
34.113	2.62615	501	22.4	19.2
34.124	2.62540	473	21.7	19.2
34.134	2.62465	488	22.1	19.2
34.144	2.62389	507	22.5	19.2
34.154	2.62314	468	21.6	19.2
34.164	2.62239	465	21.6	19.2
34.174	2.62164	493	22.2	19.2
34.184	2.62088	450	21.2	19.2
34.194	2.62013	507	22.5	19.2
34.204	2.61938	499	22.3	19.2
34.214	2.61863	495	22.2	19.2
34.225	2.61788	483	22.0	19.2
34.235	2.61713	497	22.3	19.2
34.245	2.61639	499	22.3	19.2
34.255	2.61564	457	21.4	19.2
34.265	2.61489	461	21.5	19.2
34.275	2.61414	464	21.5	19.2

34.285	2.61339	506	22.5	19.2
34.295	2.61265	487	22.1	19.2
34.305	2.61190	486	22.0	19.2
34.315	2.61116	461	21.5	19.2
34.326	2.61041	511	22.6	19.2
34.336	2.60967	513	22.6	19.2
34.346	2.60892	499	22.3	19.2
34.356	2.60818	486	22.0	19.2
34.366	2.60744	448	21.2	19.2
34.376	2.60669	488	22.1	19.2
34.386	2.60595	468	21.6	19.2
34.396	2.60521	517	22.7	19.2
34.406	2.60447	487	22.1	19.2
34.416	2.60373	498	22.3	19.2
34.427	2.60298	497	22.3	19.2
34.437	2.60224	533	23.1	19.2
34.447	2.60150	466	21.6	19.2
34.457	2.60077	496	22.3	19.2
34.467	2.60003	433	20.8	19.2
34.477	2.59929	480	21.9	19.2
34.487	2.59855	483	22.0	19.2
34.497	2.59781	498	22.3	19.2
34.507	2.59707	489	22.1	19.2
34.517	2.59634	473	21.7	19.2
34.528	2.59560	447	21.1	19.2
34.538	2.59487	475	21.8	19.2
34.548	2.59413	429	20.7	19.2
34.558	2.59340	489	22.1	19.2
34.568	2.59266	462	21.5	19.2
34.578	2.59193	488	22.1	19.2
34.588	2.59119	492	22.2	19.2
34.598	2.59046	483	22.0	19.2
34.608	2.58973	453	21.3	19.2
34.618	2.58899	489	22.1	19.2
34.629	2.58826	477	21.8	19.2
34.639	2.58753	472	21.7	19.2
34.649	2.58680	441	21.0	19.2
34.659	2.58607	454	21.3	19.2
34.669	2.58534	472	21.7	19.2
34.679	2.58461	484	22.0	19.2
34.689	2.58388	472	21.7	19.2
34.699	2.58315	461	21.5	19.2
34.709	2.58242	455	21.3	19.2
34.719	2.58169	479	21.9	19.2
34.730	2.58097	472	21.7	19.2
34.740	2.58024	470	21.7	19.2
34.750	2.57951	461	21.5	19.2
34.760	2.57879	497	22.3	19.2
34.770	2.57806	422	20.5	19.2
34.780	2.57734	478	21.9	19.2

34.790	2.57661	437	20.9	19.2
34.800	2.57589	496	22.3	19.2
34.810	2.57516	492	22.2	19.2
34.820	2.57444	483	22.0	19.2
34.831	2.57371	461	21.5	19.2
34.841	2.57299	449	21.2	19.2
34.851	2.57227	469	21.7	19.2
34.861	2.57155	453	21.3	19.2
34.871	2.57083	438	20.9	19.2
34.881	2.57010	411	20.3	19.2
34.891	2.56938	499	22.3	19.2
34.901	2.56866	469	21.7	19.2
34.911	2.56794	443	21.0	19.2
34.921	2.56722	432	20.8	19.2
34.932	2.56650	454	21.3	19.2
34.942	2.56579	483	22.0	19.2
34.952	2.56507	420	20.5	19.2
34.962	2.56435	436	20.9	19.2
34.972	2.56363	451	21.2	19.2
34.982	2.56292	466	21.6	19.2
34.992	2.56220	473	21.7	19.2
35.002	2.56148	442	21.0	19.2
35.012	2.56077	451	21.2	19.2
35.022	2.56005	456	21.4	19.2
35.033	2.55934	445	21.1	19.2
35.043	2.55862	443	21.0	19.2
35.053	2.55791	489	22.1	19.2
35.063	2.55719	477	21.8	19.2
35.073	2.55648	467	21.6	19.2
35.083	2.55577	465	21.6	19.2
35.093	2.55506	446	21.1	19.2
35.103	2.55434	458	21.4	19.2
35.113	2.55363	416	20.4	19.2
35.123	2.55292	414	20.3	19.2
35.134	2.55221	477	21.8	19.2
35.144	2.55150	446	21.1	19.2
35.154	2.55079	496	22.3	19.2
35.164	2.55008	434	20.8	19.2
35.174	2.54937	433	20.8	19.2
35.184	2.54866	506	22.5	19.2
35.194	2.54795	431	20.8	19.2
35.204	2.54725	471	21.7	19.2
35.214	2.54654	457	21.4	19.2
35.224	2.54583	422	20.5	19.2
35.235	2.54513	494	22.2	19.2
35.245	2.54442	460	21.4	19.2
35.255	2.54371	435	20.9	19.2
35.265	2.54301	481	21.9	19.2
35.275	2.54230	479	21.9	19.2
35.285	2.54160	466	21.6	19.2

35.295	2.54090	444	21.1	19.2
35.305	2.54019	415	20.4	19.2
35.315	2.53949	409	20.2	19.2
35.325	2.53879	459	21.4	19.2
35.335	2.53808	452	21.3	19.2
35.346	2.53738	461	21.5	19.2
35.356	2.53668	436	20.9	19.2
35.366	2.53598	453	21.3	19.2
35.376	2.53528	469	21.7	19.2
35.386	2.53458	458	21.4	19.2
35.396	2.53388	442	21.0	19.2
35.406	2.53318	434	20.8	19.2
35.416	2.53248	446	21.1	19.2
35.426	2.53178	431	20.8	19.2
35.436	2.53108	433	20.8	19.2
35.447	2.53038	476	21.8	19.2
35.457	2.52969	467	21.6	19.2
35.467	2.52899	432	20.8	19.2
35.477	2.52829	432	20.8	19.2
35.487	2.52759	443	21.0	19.2
35.497	2.52690	433	20.8	19.2
35.507	2.52620	445	21.1	19.2
35.517	2.52551	436	20.9	19.2
35.527	2.52481	494	22.2	19.2
35.537	2.52412	449	21.2	19.2
35.548	2.52342	459	21.4	19.2
35.558	2.52273	481	21.9	19.2
35.568	2.52204	486	22.0	19.2
35.578	2.52135	470	21.7	19.2
35.588	2.52065	437	20.9	19.2
35.598	2.51996	431	20.8	19.2
35.608	2.51927	447	21.1	19.2
35.618	2.51858	465	21.6	19.2
35.628	2.51789	432	20.8	19.2
35.638	2.51720	433	20.8	19.2
35.649	2.51651	450	21.2	19.2
35.659	2.51582	450	21.2	19.2
35.669	2.51513	442	21.0	19.2
35.679	2.51444	452	21.3	19.2
35.689	2.51375	434	20.8	19.2
35.699	2.51306	478	21.9	19.2
35.709	2.51238	431	20.8	19.2
35.719	2.51169	447	21.1	19.2
35.729	2.51100	451	21.2	19.2
35.739	2.51032	440	21.0	19.2
35.750	2.50963	411	20.3	19.2
35.760	2.50894	433	20.8	19.2
35.770	2.50826	469	21.7	19.2
35.780	2.50757	440	21.0	19.2
35.790	2.50689	454	21.3	19.2

35.800	2.50621	435	20.9	19.2
35.810	2.50552	426	20.6	19.2
35.820	2.50484	424	20.6	19.2
35.830	2.50416	462	21.5	19.2
35.840	2.50347	429	20.7	19.2
35.851	2.50279	387	19.7	19.2
35.861	2.50211	467	21.6	19.2
35.871	2.50143	426	20.6	19.2
35.881	2.50075	437	20.9	19.2
35.891	2.50007	444	21.1	19.2
35.901	2.49939	441	21.0	19.2
35.911	2.49871	429	20.7	19.2
35.921	2.49803	411	20.3	19.2
35.931	2.49735	430	20.7	19.2
35.941	2.49667	422	20.5	19.2
35.952	2.49599	445	21.1	19.2
35.962	2.49531	431	20.8	19.2
35.972	2.49464	423	20.6	19.2
35.982	2.49396	429	20.7	19.2
35.992	2.49328	442	21.0	19.2
36.002	2.49261	408	20.2	19.2
36.012	2.49193	460	21.4	19.2
36.022	2.49125	450	21.2	19.2
36.032	2.49058	424	20.6	19.2
36.042	2.48990	417	20.4	19.2
36.053	2.48923	434	20.8	19.2
36.063	2.48856	469	21.7	19.2
36.073	2.48788	388	19.7	19.2
36.083	2.48721	438	20.9	19.2
36.093	2.48654	445	21.1	19.2
36.103	2.48586	413	20.3	19.2
36.113	2.48519	471	21.7	19.2
36.123	2.48452	418	20.4	19.2
36.133	2.48385	457	21.4	19.2
36.143	2.48318	438	20.9	19.2
36.154	2.48251	431	20.8	19.2
36.164	2.48184	457	21.4	19.2
36.174	2.48117	411	20.3	19.2
36.184	2.48050	462	21.5	19.2
36.194	2.47983	447	21.1	19.2
36.204	2.47916	427	20.7	19.2
36.214	2.47849	440	21.0	19.2
36.224	2.47783	428	20.7	19.2
36.234	2.47716	426	20.6	19.2
36.244	2.47649	431	20.8	19.2
36.255	2.47582	442	21.0	19.2
36.265	2.47516	449	21.2	19.2
36.275	2.47449	393	19.8	19.2
36.285	2.47383	436	20.9	19.2
36.295	2.47316	443	21.0	19.2

36.305	2.47250	464	21.5	19.2
36.315	2.47183	419	20.5	19.2
36.325	2.47117	424	20.6	19.2
36.335	2.47050	442	21.0	19.2
36.345	2.46984	452	21.3	19.2
36.356	2.46918	427	20.7	19.2
36.366	2.46852	430	20.7	19.2
36.376	2.46785	405	20.1	19.2
36.386	2.46719	406	20.1	19.2
36.396	2.46653	454	21.3	19.2
36.406	2.46587	422	20.5	19.2
36.416	2.46521	445	21.1	19.2
36.426	2.46455	444	21.1	19.2
36.436	2.46389	408	20.2	19.2
36.446	2.46323	404	20.1	19.2
36.457	2.46257	488	22.1	19.2
36.467	2.46191	416	20.4	19.2
36.477	2.46125	416	20.4	19.2
36.487	2.46059	439	21.0	19.2
36.497	2.45994	384	19.6	19.2
36.507	2.45928	411	20.3	19.2
36.517	2.45862	441	21.0	19.2
36.527	2.45797	407	20.2	19.2
36.537	2.45731	429	20.7	19.2
36.547	2.45665	391	19.8	19.2
36.558	2.45600	409	20.2	19.2
36.568	2.45534	469	21.7	19.2
36.578	2.45469	457	21.4	19.2
36.588	2.45403	438	20.9	19.2
36.598	2.45338	420	20.5	19.2
36.608	2.45273	427	20.7	19.2
36.618	2.45207	404	20.1	19.2
36.628	2.45142	418	20.4	19.2
36.638	2.45077	445	21.1	19.2
36.648	2.45011	439	21.0	19.2
36.658	2.44946	407	20.2	19.2
36.669	2.44881	442	21.0	19.2
36.679	2.44816	390	19.7	19.2
36.689	2.44751	431	20.8	19.2
36.699	2.44686	446	21.1	19.2
36.709	2.44621	431	20.8	19.2
36.719	2.44556	420	20.5	19.2
36.729	2.44491	432	20.8	19.2
36.739	2.44426	430	20.7	19.2
36.749	2.44361	457	21.4	19.2
36.759	2.44296	428	20.7	19.2
36.770	2.44232	435	20.9	19.2
36.780	2.44167	448	21.2	19.2
36.790	2.44102	459	21.4	19.2
36.800	2.44038	457	21.4	19.2

36.810	2.43973	419	20.5	19.2
36.820	2.43908	441	21.0	19.2
36.830	2.43844	421	20.5	19.2
36.840	2.43779	431	20.8	19.2
36.850	2.43715	412	20.3	19.2
36.860	2.43650	434	20.8	19.2
36.871	2.43586	397	19.9	19.2
36.881	2.43522	419	20.5	19.2
36.891	2.43457	437	20.9	19.2
36.901	2.43393	434	20.8	19.2
36.911	2.43329	463	21.5	19.2
36.921	2.43264	421	20.5	19.2
36.931	2.43200	443	21.0	19.2
36.941	2.43136	434	20.8	19.2
36.951	2.43072	463	21.5	19.2
36.961	2.43008	422	20.5	19.2
36.972	2.42944	432	20.8	19.2
36.982	2.42880	458	21.4	19.2
36.992	2.42816	421	20.5	19.2
37.002	2.42752	423	20.6	19.2
37.012	2.42688	432	20.8	19.2
37.022	2.42624	436	20.9	19.2
37.032	2.42560	397	19.9	19.2
37.042	2.42496	388	19.7	19.2
37.052	2.42432	418	20.4	19.2
37.062	2.42369	439	21.0	19.2
37.073	2.42305	428	20.7	19.2
37.083	2.42241	436	20.9	19.2
37.093	2.42178	427	20.7	19.2
37.103	2.42114	430	20.7	19.2
37.113	2.42051	428	20.7	19.2
37.123	2.41987	427	20.7	19.2
37.133	2.41923	449	21.2	19.2
37.143	2.41860	411	20.3	19.2
37.153	2.41797	465	21.6	19.2
37.163	2.41733	440	21.0	19.2
37.174	2.41670	434	20.8	19.2
37.184	2.41607	408	20.2	19.2
37.194	2.41543	417	20.4	19.2
37.204	2.41480	411	20.3	19.2
37.214	2.41417	458	21.4	19.2
37.224	2.41354	440	21.0	19.2
37.234	2.41290	412	20.3	19.2
37.244	2.41227	457	21.4	19.2
37.254	2.41164	407	20.2	19.2
37.264	2.41101	450	21.2	19.2
37.275	2.41038	416	20.4	19.2
37.285	2.40975	477	21.8	19.2
37.295	2.40912	433	20.8	19.2
37.305	2.40849	430	20.7	19.2

37.315	2.40787	423	20.6	19.2
37.325	2.40724	402	20.0	19.2
37.335	2.40661	426	20.6	19.2
37.345	2.40598	390	19.7	19.2
37.355	2.40535	409	20.2	19.2
37.365	2.40473	402	20.0	19.2
37.376	2.40410	431	20.8	19.2
37.386	2.40347	424	20.6	19.2
37.396	2.40285	438	20.9	19.2
37.406	2.40222	412	20.3	19.2
37.416	2.40160	425	20.6	19.2
37.426	2.40097	422	20.5	19.2
37.436	2.40035	438	20.9	19.2
37.446	2.39972	432	20.8	19.2
37.456	2.39910	398	19.9	19.2
37.466	2.39848	384	19.6	19.2
37.477	2.39785	412	20.3	19.2
37.487	2.39723	443	21.0	19.2
37.497	2.39661	389	19.7	19.2
37.507	2.39599	396	19.9	19.2
37.517	2.39537	418	20.4	19.2
37.527	2.39474	410	20.2	19.2
37.537	2.39412	403	20.1	19.2
37.547	2.39350	408	20.2	19.2
37.557	2.39288	408	20.2	19.2
37.567	2.39226	363	19.1	19.2
37.578	2.39164	412	20.3	19.2
37.588	2.39102	420	20.5	19.2
37.598	2.39040	402	20.0	19.2
37.608	2.38978	410	20.2	19.2
37.618	2.38917	427	20.7	19.2
37.628	2.38855	416	20.4	19.2
37.638	2.38793	419	20.5	19.2
37.648	2.38731	400	20.0	19.2
37.658	2.38670	448	21.2	19.2
37.668	2.38608	395	19.9	19.2
37.679	2.38546	427	20.7	19.2
37.689	2.38485	384	19.6	19.2
37.699	2.38423	406	20.1	19.2
37.709	2.38362	426	20.6	19.2
37.719	2.38300	398	19.9	19.2
37.729	2.38239	398	19.9	19.2
37.739	2.38177	402	20.0	19.2
37.749	2.38116	425	20.6	19.2
37.759	2.38054	405	20.1	19.2
37.769	2.37993	402	20.0	19.2
37.780	2.37932	408	20.2	19.2
37.790	2.37871	419	20.5	19.2
37.800	2.37809	422	20.5	19.2
37.810	2.37748	431	20.8	19.2

37.820	2.37687	390	19.7	19.2
37.830	2.37626	459	21.4	19.2
37.840	2.37565	395	19.9	19.2
37.850	2.37504	408	20.2	19.2
37.860	2.37443	416	20.4	19.2
37.870	2.37382	425	20.6	19.2
37.881	2.37321	402	20.0	19.2
37.891	2.37260	414	20.3	19.2
37.901	2.37199	392	19.8	19.2
37.911	2.37138	398	19.9	19.2
37.921	2.37077	437	20.9	19.2
37.931	2.37016	408	20.2	19.2
37.941	2.36956	375	19.4	19.2
37.951	2.36895	416	20.4	19.2
37.961	2.36834	443	21.0	19.2
37.971	2.36773	426	20.6	19.2
37.981	2.36713	400	20.0	19.2
37.992	2.36652	425	20.6	19.2
38.002	2.36592	400	20.0	19.2
38.012	2.36531	394	19.8	19.2
38.022	2.36471	414	20.3	19.2
38.032	2.36410	402	20.0	19.2
38.042	2.36350	374	19.3	19.2
38.052	2.36289	438	20.9	19.2
38.062	2.36229	400	20.0	19.2
38.072	2.36169	390	19.7	19.2
38.082	2.36108	415	20.4	19.2
38.093	2.36048	412	20.3	19.2
38.103	2.35988	422	20.5	19.2
38.113	2.35927	425	20.6	19.2
38.123	2.35867	443	21.0	19.2
38.133	2.35807	404	20.1	19.2
38.143	2.35747	379	19.5	19.2
38.153	2.35687	403	20.1	19.2
38.163	2.35627	437	20.9	19.2
38.173	2.35567	436	20.9	19.2
38.183	2.35507	393	19.8	19.2
38.194	2.35447	427	20.7	19.2
38.204	2.35387	403	20.1	19.2
38.214	2.35327	412	20.3	19.2
38.224	2.35267	395	19.9	19.2
38.234	2.35207	387	19.7	19.2
38.244	2.35148	417	20.4	19.2
38.254	2.35088	421	20.5	19.2
38.264	2.35028	407	20.2	19.2
38.274	2.34969	369	19.2	19.2
38.284	2.34909	413	20.3	19.2
38.295	2.34849	387	19.7	19.2
38.305	2.34790	410	20.2	19.2
38.315	2.34730	420	20.5	19.2

38.325	2.34671	414	20.3	19.2
38.335	2.34611	412	20.3	19.2
38.345	2.34552	422	20.5	19.2
38.355	2.34492	438	20.9	19.2
38.365	2.34433	453	21.3	19.2
38.375	2.34373	421	20.5	19.2
38.385	2.34314	414	20.3	19.2
38.396	2.34255	401	20.0	19.2
38.406	2.34195	384	19.6	19.2
38.416	2.34136	431	20.8	19.2
38.426	2.34077	372	19.3	19.2
38.436	2.34018	398	19.9	19.2
38.446	2.33959	359	18.9	19.2
38.456	2.33900	381	19.5	19.2
38.466	2.33840	423	20.6	19.2
38.476	2.33781	406	20.1	19.2
38.486	2.33722	416	20.4	19.2
38.497	2.33663	392	19.8	19.2
38.507	2.33604	414	20.3	19.2
38.517	2.33545	411	20.3	19.2
38.527	2.33487	407	20.2	19.2
38.537	2.33428	427	20.7	19.2
38.547	2.33369	377	19.4	19.2
38.557	2.33310	439	21.0	19.2
38.567	2.33251	442	21.0	19.2
38.577	2.33193	414	20.3	19.2
38.587	2.33134	434	20.8	19.2
38.598	2.33075	436	20.9	19.2
38.608	2.33017	432	20.8	19.2
38.618	2.32958	444	21.1	19.2
38.628	2.32899	418	20.4	19.2
38.638	2.32841	397	19.9	19.2
38.648	2.32782	411	20.3	19.2
38.658	2.32724	412	20.3	19.2
38.668	2.32665	413	20.3	19.2
38.678	2.32607	399	20.0	19.2
38.688	2.32549	416	20.4	19.2
38.699	2.32490	425	20.6	19.2
38.709	2.32432	452	21.3	19.2
38.719	2.32374	391	19.8	19.2
38.729	2.32315	389	19.7	19.2
38.739	2.32257	422	20.5	19.2
38.749	2.32199	392	19.8	19.2
38.759	2.32141	400	20.0	19.2
38.769	2.32082	404	20.1	19.2
38.779	2.32024	368	19.2	19.2
38.789	2.31966	404	20.1	19.2
38.800	2.31908	403	20.1	19.2
38.810	2.31850	394	19.8	19.2
38.820	2.31792	422	20.5	19.2

38.830	2.31734	416	20.4	19.2
38.840	2.31676	413	20.3	19.2
38.850	2.31618	396	19.9	19.2
38.860	2.31561	410	20.2	19.2
38.870	2.31503	377	19.4	19.2
38.880	2.31445	416	20.4	19.2
38.890	2.31387	447	21.1	19.2
38.901	2.31329	419	20.5	19.2
38.911	2.31272	417	20.4	19.2
38.921	2.31214	435	20.9	19.2
38.931	2.31156	421	20.5	19.2
38.941	2.31099	405	20.1	19.2
38.951	2.31041	440	21.0	19.2
38.961	2.30983	415	20.4	19.2
38.971	2.30926	416	20.4	19.2
38.981	2.30868	420	20.5	19.2
38.991	2.30811	445	21.1	19.2
39.002	2.30754	403	20.1	19.2
39.012	2.30696	414	20.3	19.2
39.022	2.30639	393	19.8	19.2
39.032	2.30581	405	20.1	19.2
39.042	2.30524	419	20.5	19.2
39.052	2.30467	407	20.2	19.2
39.062	2.30410	398	19.9	19.2
39.072	2.30352	409	20.2	19.2
39.082	2.30295	429	20.7	19.2
39.092	2.30238	415	20.4	19.2
39.103	2.30181	404	20.1	19.2
39.113	2.30124	417	20.4	19.2
39.123	2.30067	420	20.5	19.2
39.133	2.30010	412	20.3	19.2
39.143	2.29953	415	20.4	19.2
39.153	2.29896	389	19.7	19.2
39.163	2.29839	382	19.5	19.2
39.173	2.29782	390	19.7	19.2
39.183	2.29725	429	20.7	19.2
39.193	2.29668	412	20.3	19.2
39.204	2.29611	364	19.1	19.2
39.214	2.29554	434	20.8	19.2
39.224	2.29497	408	20.2	19.2
39.234	2.29441	417	20.4	19.2
39.244	2.29384	427	20.7	19.2
39.254	2.29327	421	20.5	19.2
39.264	2.29271	397	19.9	19.2
39.274	2.29214	404	20.1	19.2
39.284	2.29157	378	19.4	19.2
39.294	2.29101	426	20.6	19.2
39.304	2.29044	415	20.4	19.2
39.315	2.28988	407	20.2	19.2
39.325	2.28931	399	20.0	19.2

39.335	2.28875	409	20.2	19.2
39.345	2.28818	378	19.4	19.2
39.355	2.28762	425	20.6	19.2
39.365	2.28706	449	21.2	19.2
39.375	2.28649	420	20.5	19.2
39.385	2.28593	411	20.3	19.2
39.395	2.28537	436	20.9	19.2
39.405	2.28481	412	20.3	19.2
39.416	2.28424	391	19.8	19.2
39.426	2.28368	439	21.0	19.2
39.436	2.28312	424	20.6	19.2
39.446	2.28256	372	19.3	19.2
39.456	2.28200	410	20.2	19.2
39.466	2.28144	397	19.9	19.2
39.476	2.28088	400	20.0	19.2
39.486	2.28032	379	19.5	19.2
39.496	2.27976	442	21.0	19.2
39.506	2.27920	406	20.1	19.2
39.517	2.27864	395	19.9	19.2
39.527	2.27808	399	20.0	19.2
39.537	2.27752	406	20.1	19.2
39.547	2.27696	447	21.1	19.2
39.557	2.27640	431	20.8	19.2
39.567	2.27585	406	20.1	19.2
39.577	2.27529	448	21.2	19.2
39.587	2.27473	393	19.8	19.2
39.597	2.27417	434	20.8	19.2
39.607	2.27362	438	20.9	19.2
39.618	2.27306	399	20.0	19.2
39.628	2.27251	409	20.2	19.2
39.638	2.27195	418	20.4	19.2
39.648	2.27139	406	20.1	19.2
39.658	2.27084	394	19.8	19.2
39.668	2.27028	377	19.4	19.2
39.678	2.26973	359	18.9	19.2
39.688	2.26918	442	21.0	19.2
39.698	2.26862	416	20.4	19.2
39.708	2.26807	403	20.1	19.2
39.719	2.26751	427	20.7	19.2
39.729	2.26696	413	20.3	19.2
39.739	2.26641	385	19.6	19.2
39.749	2.26586	417	20.4	19.2
39.759	2.26530	424	20.6	19.2
39.769	2.26475	407	20.2	19.2
39.779	2.26420	411	20.3	19.2
39.789	2.26365	437	20.9	19.2
39.799	2.26310	415	20.4	19.2
39.809	2.26255	437	20.9	19.2
39.820	2.26200	411	20.3	19.2
39.830	2.26145	402	20.0	19.2

39.840	2.26090	368	19.2	19.2
39.850	2.26035	436	20.9	19.2
39.860	2.25980	437	20.9	19.2
39.870	2.25925	415	20.4	19.2
39.880	2.25870	419	20.5	19.2
39.890	2.25815	428	20.7	19.2
39.900	2.25760	417	20.4	19.2
39.910	2.25705	394	19.8	19.2
39.921	2.25651	427	20.7	19.2
39.931	2.25596	410	20.2	19.2
39.941	2.25541	420	20.5	19.2
39.951	2.25486	421	20.5	19.2
39.961	2.25432	417	20.4	19.2
39.971	2.25377	430	20.7	19.2
39.981	2.25323	427	20.7	19.2
39.991	2.25268	413	20.3	19.2
40.001	2.25213	412	20.3	19.2
40.011	2.25159	426	20.6	19.2
40.022	2.25104	393	19.8	19.2
40.032	2.25050	406	20.1	19.2
40.042	2.24996	397	19.9	19.2
40.052	2.24941	375	19.4	19.2
40.062	2.24887	395	19.9	19.2
40.072	2.24832	361	19.0	19.2
40.082	2.24778	421	20.5	19.2
40.092	2.24724	405	20.1	19.2
40.102	2.24669	404	20.1	19.2
40.112	2.24615	415	20.4	19.2
40.123	2.24561	416	20.4	19.2
40.133	2.24507	415	20.4	19.2
40.143	2.24453	420	20.5	19.2
40.153	2.24399	391	19.8	19.2
40.163	2.24344	402	20.0	19.2
40.173	2.24290	413	20.3	19.2
40.183	2.24236	415	20.4	19.2
40.193	2.24182	391	19.8	19.2
40.203	2.24128	440	21.0	19.2
40.213	2.24074	423	20.6	19.2
40.224	2.24020	396	19.9	19.2
40.234	2.23967	374	19.3	19.2
40.244	2.23913	417	20.4	19.2
40.254	2.23859	412	20.3	19.2
40.264	2.23805	383	19.6	19.2
40.274	2.23751	403	20.1	19.2
40.284	2.23697	419	20.5	19.2
40.294	2.23644	401	20.0	19.2
40.304	2.23590	408	20.2	19.2
40.314	2.23536	437	20.9	19.2
40.325	2.23483	388	19.7	19.2
40.335	2.23429	410	20.2	19.2

40.345	2.23375	399	20.0	19.2
40.355	2.23322	390	19.7	19.2
40.365	2.23268	377	19.4	19.2
40.375	2.23215	380	19.5	19.2
40.385	2.23161	386	19.6	19.2
40.395	2.23108	394	19.8	19.2
40.405	2.23054	367	19.2	19.2
40.415	2.23001	376	19.4	19.2
40.426	2.22948	434	20.8	19.2
40.436	2.22894	411	20.3	19.2
40.446	2.22841	363	19.1	19.2
40.456	2.22788	357	18.9	19.2
40.466	2.22734	398	19.9	19.2
40.476	2.22681	371	19.3	19.2
40.486	2.22628	407	20.2	19.2
40.496	2.22575	415	20.4	19.2
40.506	2.22522	399	20.0	19.2
40.516	2.22468	367	19.2	19.2
40.527	2.22415	474	21.8	19.2
40.537	2.22362	396	19.9	19.2
40.547	2.22309	384	19.6	19.2
40.557	2.22256	377	19.4	19.2
40.567	2.22203	407	20.2	19.2
40.577	2.22150	432	20.8	19.2
40.587	2.22097	419	20.5	19.2
40.597	2.22044	373	19.3	19.2
40.607	2.21991	373	19.3	19.2
40.617	2.21939	365	19.1	19.2
40.627	2.21886	389	19.7	19.2
40.638	2.21833	389	19.7	19.2
40.648	2.21780	409	20.2	19.2
40.658	2.21727	411	20.3	19.2
40.668	2.21675	428	20.7	19.2
40.678	2.21622	413	20.3	19.2
40.688	2.21569	408	20.2	19.2
40.698	2.21517	429	20.7	19.2
40.708	2.21464	387	19.7	19.2
40.718	2.21411	402	20.0	19.2
40.728	2.21359	372	19.3	19.2
40.739	2.21306	406	20.1	19.2
40.749	2.21254	376	19.4	19.2
40.759	2.21201	393	19.8	19.2
40.769	2.21149	375	19.4	19.2
40.779	2.21096	387	19.7	19.2
40.789	2.21044	388	19.7	19.2
40.799	2.20992	400	20.0	19.2
40.809	2.20939	404	20.1	19.2
40.819	2.20887	423	20.6	19.2
40.829	2.20835	397	19.9	19.2
40.840	2.20782	384	19.6	19.2

40.850	2.20730	408	20.2	19.2
40.860	2.20678	394	19.8	19.2
40.870	2.20626	387	19.7	19.2
40.880	2.20573	425	20.6	19.2
40.890	2.20521	407	20.2	19.2
40.900	2.20469	397	19.9	19.2
40.910	2.20417	418	20.4	19.2
40.920	2.20365	376	19.4	19.2
40.930	2.20313	417	20.4	19.2
40.941	2.20261	380	19.5	19.2
40.951	2.20209	372	19.3	19.2
40.961	2.20157	348	18.7	19.2
40.971	2.20105	414	20.3	19.2
40.981	2.20053	413	20.3	19.2
40.991	2.20001	408	20.2	19.2
41.001	2.19949	409	20.2	19.2
41.011	2.19898	385	19.6	19.2
41.021	2.19846	386	19.6	19.2
41.031	2.19794	384	19.6	19.2
41.042	2.19742	387	19.7	19.2
41.052	2.19690	403	20.1	19.2
41.062	2.19639	370	19.2	19.2
41.072	2.19587	407	20.2	19.2
41.082	2.19535	367	19.2	19.2
41.092	2.19484	409	20.2	19.2
41.102	2.19432	388	19.7	19.2
41.112	2.19381	371	19.3	19.2
41.122	2.19329	386	19.6	19.2
41.132	2.19278	403	20.1	19.2
41.143	2.19226	366	19.1	19.2
41.153	2.19175	406	20.1	19.2
41.163	2.19123	412	20.3	19.2
41.173	2.19072	390	19.7	19.2
41.183	2.19020	411	20.3	19.2
41.193	2.18969	407	20.2	19.2
41.203	2.18918	382	19.5	19.2
41.213	2.18866	419	20.5	19.2
41.223	2.18815	406	20.1	19.2
41.233	2.18764	421	20.5	19.2
41.244	2.18713	382	19.5	19.2
41.254	2.18661	407	20.2	19.2
41.264	2.18610	401	20.0	19.2
41.274	2.18559	371	19.3	19.2
41.284	2.18508	377	19.4	19.2
41.294	2.18457	391	19.8	19.2
41.304	2.18406	384	19.6	19.2
41.314	2.18355	414	20.3	19.2
41.324	2.18304	363	19.1	19.2
41.334	2.18253	381	19.5	19.2
41.345	2.18202	377	19.4	19.2

41.355	2.18151	404	20.1	19.2
41.365	2.18100	412	20.3	19.2
41.375	2.18049	368	19.2	19.2
41.385	2.17998	413	20.3	19.2
41.395	2.17947	354	18.8	19.2
41.405	2.17896	390	19.7	19.2
41.415	2.17846	395	19.9	19.2
41.425	2.17795	421	20.5	19.2
41.435	2.17744	363	19.1	19.2
41.446	2.17693	416	20.4	19.2
41.456	2.17643	418	20.4	19.2
41.466	2.17592	415	20.4	19.2
41.476	2.17541	358	18.9	19.2
41.486	2.17491	371	19.3	19.2
41.496	2.17440	421	20.5	19.2
41.506	2.17389	398	19.9	19.2
41.516	2.17339	383	19.6	19.2
41.526	2.17288	388	19.7	19.2
41.536	2.17238	471	21.7	19.2
41.547	2.17187	402	20.0	19.2
41.557	2.17137	403	20.1	19.2
41.567	2.17087	404	20.1	19.2
41.577	2.17036	416	20.4	19.2
41.587	2.16986	368	19.2	19.2
41.597	2.16935	367	19.2	19.2
41.607	2.16885	422	20.5	19.2
41.617	2.16835	409	20.2	19.2
41.627	2.16785	378	19.4	19.2
41.637	2.16734	388	19.7	19.2
41.648	2.16684	389	19.7	19.2
41.658	2.16634	369	19.2	19.2
41.668	2.16584	374	19.3	19.2
41.678	2.16534	377	19.4	19.2
41.688	2.16483	408	20.2	19.2
41.698	2.16433	367	19.2	19.2
41.708	2.16383	381	19.5	19.2
41.718	2.16333	390	19.7	19.2
41.728	2.16283	417	20.4	19.2
41.738	2.16233	381	19.5	19.2
41.749	2.16183	391	19.8	19.2
41.759	2.16133	383	19.6	19.2
41.769	2.16083	370	19.2	19.2
41.779	2.16033	394	19.8	19.2
41.789	2.15984	408	20.2	19.2
41.799	2.15934	369	19.2	19.2
41.809	2.15884	375	19.4	19.2
41.819	2.15834	353	18.8	19.2
41.829	2.15784	405	20.1	19.2
41.839	2.15735	396	19.9	19.2
41.850	2.15685	389	19.7	19.2

41.860	2.15635	341	18.5	19.2
41.870	2.15585	417	20.4	19.2
41.880	2.15536	363	19.1	19.2
41.890	2.15486	373	19.3	19.2
41.900	2.15437	365	19.1	19.2
41.910	2.15387	396	19.9	19.2
41.920	2.15337	382	19.5	19.2
41.930	2.15288	419	20.5	19.2
41.940	2.15238	382	19.5	19.2
41.950	2.15189	384	19.6	19.2
41.961	2.15139	387	19.7	19.2
41.971	2.15090	381	19.5	19.2
41.981	2.15041	381	19.5	19.2
41.991	2.14991	404	20.1	19.2
42.001	2.14942	370	19.2	19.2
42.011	2.14893	391	19.8	19.2
42.021	2.14843	381	19.5	19.2
42.031	2.14794	386	19.6	19.2
42.041	2.14745	341	18.5	19.2
42.051	2.14695	369	19.2	19.2
42.062	2.14646	430	20.7	19.2
42.072	2.14597	388	19.7	19.2
42.082	2.14548	469	21.7	19.2
42.092	2.14499	391	19.8	19.2
42.102	2.14450	402	20.0	19.2
42.112	2.14400	349	18.7	19.2
42.122	2.14351	391	19.8	19.2
42.132	2.14302	384	19.6	19.2
42.142	2.14253	369	19.2	19.2
42.152	2.14204	419	20.5	19.2
42.163	2.14155	386	19.6	19.2
42.173	2.14106	406	20.1	19.2
42.183	2.14058	393	19.8	19.2
42.193	2.14009	372	19.3	19.2
42.203	2.13960	375	19.4	19.2
42.213	2.13911	353	18.8	19.2
42.223	2.13862	362	19.0	19.2
42.233	2.13813	400	20.0	19.2
42.243	2.13764	403	20.1	19.2
42.253	2.13716	395	19.9	19.2
42.264	2.13667	356	18.9	19.2
42.274	2.13618	375	19.4	19.2
42.284	2.13570	391	19.8	19.2
42.294	2.13521	385	19.6	19.2
42.304	2.13472	387	19.7	19.2
42.314	2.13424	390	19.7	19.2
42.324	2.13375	362	19.0	19.2
42.334	2.13327	376	19.4	19.2
42.344	2.13278	351	18.7	19.2
42.354	2.13229	356	18.9	19.2

42.365	2.13181	379	19.5	19.2
42.375	2.13132	399	20.0	19.2
42.385	2.13084	371	19.3	19.2
42.395	2.13036	405	20.1	19.2
42.405	2.12987	385	19.6	19.2
42.415	2.12939	351	18.7	19.2
42.425	2.12890	363	19.1	19.2
42.435	2.12842	375	19.4	19.2
42.445	2.12794	371	19.3	19.2
42.455	2.12746	353	18.8	19.2
42.466	2.12697	376	19.4	19.2
42.476	2.12649	392	19.8	19.2
42.486	2.12601	345	18.6	19.2
42.496	2.12553	368	19.2	19.2
42.506	2.12504	370	19.2	19.2
42.516	2.12456	354	18.8	19.2
42.526	2.12408	359	18.9	19.2
42.536	2.12360	365	19.1	19.2
42.546	2.12312	386	19.6	19.2
42.556	2.12264	397	19.9	19.2
42.567	2.12216	378	19.4	19.2
42.577	2.12168	368	19.2	19.2
42.587	2.12120	390	19.7	19.2
42.597	2.12072	392	19.8	19.2
42.607	2.12024	363	19.1	19.2
42.617	2.11976	357	18.9	19.2
42.627	2.11928	378	19.4	19.2
42.637	2.11880	379	19.5	19.2
42.647	2.11833	382	19.5	19.2
42.657	2.11785	367	19.2	19.2
42.668	2.11737	391	19.8	19.2
42.678	2.11689	355	18.8	19.2
42.688	2.11642	388	19.7	19.2
42.698	2.11594	376	19.4	19.2
42.708	2.11546	380	19.5	19.2
42.718	2.11498	412	20.3	19.2
42.728	2.11451	354	18.8	19.2
42.738	2.11403	352	18.8	19.2
42.748	2.11356	357	18.9	19.2
42.758	2.11308	368	19.2	19.2
42.769	2.11260	386	19.6	19.2
42.779	2.11213	393	19.8	19.2
42.789	2.11165	346	18.6	19.2
42.799	2.11118	373	19.3	19.2
42.809	2.11070	364	19.1	19.2
42.819	2.11023	338	18.4	19.2
42.829	2.10976	355	18.8	19.2
42.839	2.10928	388	19.7	19.2
42.849	2.10881	349	18.7	19.2
42.859	2.10833	373	19.3	19.2

42.870	2.10786	360	19.0	19.2
42.880	2.10739	362	19.0	19.2
42.890	2.10691	370	19.2	19.2
42.900	2.10644	370	19.2	19.2
42.910	2.10597	381	19.5	19.2
42.920	2.10550	363	19.1	19.2
42.930	2.10503	374	19.3	19.2
42.940	2.10455	370	19.2	19.2
42.950	2.10408	349	18.7	19.2
42.960	2.10361	399	20.0	19.2
42.971	2.10314	371	19.3	19.2
42.981	2.10267	351	18.7	19.2
42.991	2.10220	360	19.0	19.2
43.001	2.10173	361	19.0	19.2
43.011	2.10126	390	19.7	19.2
43.021	2.10079	364	19.1	19.2
43.031	2.10032	375	19.4	19.2
43.041	2.09985	401	20.0	19.2
43.051	2.09938	352	18.8	19.2
43.061	2.09891	362	19.0	19.2
43.072	2.09844	376	19.4	19.2
43.082	2.09797	390	19.7	19.2
43.092	2.09751	367	19.2	19.2
43.102	2.09704	366	19.1	19.2
43.112	2.09657	381	19.5	19.2
43.122	2.09610	380	19.5	19.2
43.132	2.09563	378	19.4	19.2
43.142	2.09517	385	19.6	19.2
43.152	2.09470	345	18.6	19.2
43.162	2.09423	338	18.4	19.2
43.172	2.09377	367	19.2	19.2
43.183	2.09330	359	18.9	19.2
43.193	2.09284	336	18.3	19.2
43.203	2.09237	387	19.7	19.2
43.213	2.09190	351	18.7	19.2
43.223	2.09144	363	19.1	19.2
43.233	2.09097	363	19.1	19.2
43.243	2.09051	383	19.6	19.2
43.253	2.09004	363	19.1	19.2
43.263	2.08958	367	19.2	19.2
43.273	2.08911	383	19.6	19.2
43.284	2.08865	379	19.5	19.2
43.294	2.08819	369	19.2	19.2
43.304	2.08772	324	18.0	19.2
43.314	2.08726	398	19.9	19.2
43.324	2.08680	357	18.9	19.2
43.334	2.08633	359	18.9	19.2
43.344	2.08587	370	19.2	19.2
43.354	2.08541	345	18.6	19.2
43.364	2.08495	367	19.2	19.2

43.374	2.08448	364	19.1	19.2
43.385	2.08402	340	18.4	19.2
43.395	2.08356	401	20.0	19.2
43.405	2.08310	357	18.9	19.2
43.415	2.08264	378	19.4	19.2
43.425	2.08218	384	19.6	19.2
43.435	2.08172	362	19.0	19.2
43.445	2.08126	357	18.9	19.2
43.455	2.08080	357	18.9	19.2
43.465	2.08034	365	19.1	19.2
43.475	2.07988	362	19.0	19.2
43.486	2.07942	338	18.4	19.2
43.496	2.07896	362	19.0	19.2
43.506	2.07850	362	19.0	19.2
43.516	2.07804	360	19.0	19.2
43.526	2.07758	365	19.1	19.2
43.536	2.07712	372	19.3	19.2
43.546	2.07666	334	18.3	19.2
43.556	2.07620	367	19.2	19.2
43.566	2.07575	357	18.9	19.2
43.576	2.07529	356	18.9	19.2
43.587	2.07483	362	19.0	19.2
43.597	2.07437	335	18.3	19.2
43.607	2.07392	326	18.1	19.2
43.617	2.07346	332	18.2	19.2
43.627	2.07300	361	19.0	19.2
43.637	2.07255	335	18.3	19.2
43.647	2.07209	345	18.6	19.2
43.657	2.07164	330	18.2	19.2
43.667	2.07118	344	18.5	19.2
43.677	2.07072	336	18.3	19.2
43.688	2.07027	365	19.1	19.2
43.698	2.06981	362	19.0	19.2
43.708	2.06936	340	18.4	19.2
43.718	2.06890	317	17.8	19.2
43.728	2.06845	332	18.2	19.2
43.738	2.06800	360	19.0	19.2
43.748	2.06754	382	19.5	19.2
43.758	2.06709	346	18.6	19.2
43.768	2.06663	349	18.7	19.2
43.778	2.06618	321	17.9	19.2
43.789	2.06573	363	19.1	19.2
43.799	2.06528	339	18.4	19.2
43.809	2.06482	323	18.0	19.2
43.819	2.06437	337	18.4	19.2
43.829	2.06392	346	18.6	19.2
43.839	2.06347	321	17.9	19.2
43.849	2.06301	388	19.7	19.2
43.859	2.06256	348	18.7	19.2
43.869	2.06211	365	19.1	19.2

43.879	2.06166	389	19.7	19.2
43.890	2.06121	353	18.8	19.2
43.900	2.06076	321	17.9	19.2
43.910	2.06031	360	19.0	19.2
43.920	2.05986	363	19.1	19.2
43.930	2.05941	333	18.2	19.2
43.940	2.05896	330	18.2	19.2
43.950	2.05851	318	17.8	19.2
43.960	2.05806	351	18.7	19.2
43.970	2.05761	310	17.6	19.2
43.980	2.05716	363	19.1	19.2
43.991	2.05671	327	18.1	19.2
44.001	2.05626	350	18.7	19.2
44.011	2.05581	347	18.6	19.2
44.021	2.05537	340	18.4	19.2
44.031	2.05492	352	18.8	19.2
44.041	2.05447	349	18.7	19.2
44.051	2.05402	374	19.3	19.2
44.061	2.05357	339	18.4	19.2
44.071	2.05313	328	18.1	19.2
44.081	2.05268	339	18.4	19.2
44.092	2.05223	366	19.1	19.2
44.102	2.05179	322	17.9	19.2
44.112	2.05134	350	18.7	19.2
44.122	2.05089	330	18.2	19.2
44.132	2.05045	327	18.1	19.2
44.142	2.05000	352	18.8	19.2
44.152	2.04956	371	19.3	19.2
44.162	2.04911	313	17.7	19.2
44.172	2.04867	366	19.1	19.2
44.182	2.04822	354	18.8	19.2
44.193	2.04778	347	18.6	19.2
44.203	2.04733	354	18.8	19.2
44.213	2.04689	332	18.2	19.2
44.223	2.04645	322	17.9	19.2
44.233	2.04600	345	18.6	19.2
44.243	2.04556	315	17.7	19.2
44.253	2.04511	345	18.6	19.2
44.263	2.04467	336	18.3	19.2
44.273	2.04423	331	18.2	19.2
44.283	2.04379	343	18.5	19.2
44.294	2.04334	360	19.0	19.2
44.304	2.04290	357	18.9	19.2
44.314	2.04246	355	18.8	19.2
44.324	2.04202	308	17.5	19.2
44.334	2.04157	344	18.5	19.2
44.344	2.04113	359	18.9	19.2
44.354	2.04069	324	18.0	19.2
44.364	2.04025	323	18.0	19.2
44.374	2.03981	335	18.3	19.2

44.384	2.03937	322	17.9	19.2
44.395	2.03893	336	18.3	19.2
44.405	2.03849	324	18.0	19.2
44.415	2.03805	346	18.6	19.2
44.425	2.03761	323	18.0	19.2
44.435	2.03717	334	18.3	19.2
44.445	2.03673	308	17.5	19.2
44.455	2.03629	330	18.2	19.2
44.465	2.03585	321	17.9	19.2
44.475	2.03541	310	17.6	19.2
44.485	2.03497	333	18.2	19.2
44.495	2.03454	337	18.4	19.2
44.506	2.03410	290	17.0	19.2
44.516	2.03366	348	18.7	19.2
44.526	2.03322	308	17.5	19.2
44.536	2.03278	338	18.4	19.2
44.546	2.03235	313	17.7	19.2
44.556	2.03191	317	17.8	19.2
44.566	2.03147	323	18.0	19.2
44.576	2.03104	340	18.4	19.2
44.586	2.03060	325	18.0	19.2
44.596	2.03016	313	17.7	19.2
44.607	2.02973	308	17.5	19.2
44.617	2.02929	324	18.0	19.2
44.627	2.02885	297	17.2	19.2
44.637	2.02842	330	18.2	19.2
44.647	2.02798	320	17.9	19.2
44.657	2.02755	331	18.2	19.2
44.667	2.02711	335	18.3	19.2
44.677	2.02668	343	18.5	19.2
44.687	2.02624	295	17.2	19.2
44.697	2.02581	310	17.6	19.2
44.708	2.02538	326	18.1	19.2
44.718	2.02494	320	17.9	19.2
44.728	2.02451	337	18.4	19.2
44.738	2.02407	348	18.7	19.2
44.748	2.02364	317	17.8	19.2
44.758	2.02321	309	17.6	19.2
44.768	2.02277	324	18.0	19.2
44.778	2.02234	335	18.3	19.2
44.788	2.02191	329	18.1	19.2
44.798	2.02148	317	17.8	19.2
44.809	2.02104	316	17.8	19.2
44.819	2.02061	338	18.4	19.2
44.829	2.02018	322	17.9	19.2
44.839	2.01975	322	17.9	19.2
44.849	2.01932	303	17.4	19.2
44.859	2.01889	359	18.9	19.2
44.869	2.01846	314	17.7	19.2
44.879	2.01802	320	17.9	19.2

44.889	2.01759	307	17.5	19.2
44.899	2.01716	298	17.3	19.2
44.910	2.01673	325	18.0	19.2
44.920	2.01630	284	16.9	19.2
44.930	2.01587	304	17.4	19.2
44.940	2.01544	319	17.9	19.2
44.950	2.01502	339	18.4	19.2
44.960	2.01459	294	17.1	19.2
44.970	2.01416	299	17.3	19.2
44.980	2.01373	359	18.9	19.2
44.990	2.01330	288	17.0	19.2
45.000	2.01287	337	18.4	19.2
45.011	2.01244	289	17.0	19.2
45.021	2.01202	327	18.1	19.2
45.031	2.01159	314	17.7	19.2
45.041	2.01116	307	17.5	19.2
45.051	2.01073	313	17.7	19.2
45.061	2.01031	304	17.4	19.2
45.071	2.00988	311	17.6	19.2
45.081	2.00945	286	16.9	19.2
45.091	2.00902	322	17.9	19.2
45.101	2.00860	321	17.9	19.2
45.112	2.00817	316	17.8	19.2
45.122	2.00775	318	17.8	19.2
45.132	2.00732	325	18.0	19.2
45.142	2.00689	314	17.7	19.2
45.152	2.00647	318	17.8	19.2
45.162	2.00604	310	17.6	19.2
45.172	2.00562	299	17.3	19.2
45.182	2.00519	320	17.9	19.2
45.192	2.00477	289	17.0	19.2
45.202	2.00435	346	18.6	19.2
45.213	2.00392	318	17.8	19.2
45.223	2.00350	299	17.3	19.2
45.233	2.00307	292	17.1	19.2
45.243	2.00265	299	17.3	19.2
45.253	2.00223	315	17.7	19.2
45.263	2.00180	323	18.0	19.2
45.273	2.00138	329	18.1	19.2
45.283	2.00096	337	18.4	19.2
45.293	2.00053	329	18.1	19.2
45.303	2.00011	293	17.1	19.2
45.314	1.99969	302	17.4	19.2
45.324	1.99927	290	17.0	19.2
45.334	1.99885	310	17.6	19.2
45.344	1.99842	317	17.8	19.2
45.354	1.99800	329	18.1	19.2
45.364	1.99758	320	17.9	19.2
45.374	1.99716	313	17.7	19.2
45.384	1.99674	300	17.3	19.2

45.394	1.99632	339	18.4	19.2
45.404	1.99590	277	16.6	19.2
45.415	1.99548	294	17.1	19.2
45.425	1.99506	340	18.4	19.2
45.435	1.99464	310	17.6	19.2
45.445	1.99422	322	17.9	19.2
45.455	1.99380	295	17.2	19.2
45.465	1.99338	322	17.9	19.2
45.475	1.99296	300	17.3	19.2
45.485	1.99254	307	17.5	19.2
45.495	1.99212	295	17.2	19.2
45.505	1.99170	326	18.1	19.2
45.516	1.99128	289	17.0	19.2
45.526	1.99087	291	17.1	19.2
45.536	1.99045	343	18.5	19.2
45.546	1.99003	312	17.7	19.2
45.556	1.98961	286	16.9	19.2
45.566	1.98919	346	18.6	19.2
45.576	1.98878	301	17.3	19.2
45.586	1.98836	258	16.1	19.2
45.596	1.98794	320	17.9	19.2
45.606	1.98753	292	17.1	19.2
45.617	1.98711	286	16.9	19.2
45.627	1.98669	302	17.4	19.2
45.637	1.98628	309	17.6	19.2
45.647	1.98586	299	17.3	19.2
45.657	1.98545	338	18.4	19.2
45.667	1.98503	305	17.5	19.2
45.677	1.98461	292	17.1	19.2
45.687	1.98420	305	17.5	19.2
45.697	1.98378	324	18.0	19.2
45.707	1.98337	315	17.7	19.2
45.718	1.98295	285	16.9	19.2
45.728	1.98254	321	17.9	19.2
45.738	1.98213	301	17.3	19.2
45.748	1.98171	280	16.7	19.2
45.758	1.98130	277	16.6	19.2
45.768	1.98088	305	17.5	19.2
45.778	1.98047	288	17.0	19.2
45.788	1.98006	293	17.1	19.2
45.798	1.97964	342	18.5	19.2
45.808	1.97923	310	17.6	19.2
45.818	1.97882	289	17.0	19.2
45.829	1.97841	287	16.9	19.2
45.839	1.97799	299	17.3	19.2
45.849	1.97758	283	16.8	19.2
45.859	1.97717	302	17.4	19.2
45.869	1.97676	291	17.1	19.2
45.879	1.97635	295	17.2	19.2
45.889	1.97593	273	16.5	19.2

45.899	1.97552	277	16.6	19.2
45.909	1.97511	333	18.2	19.2
45.919	1.97470	292	17.1	19.2
45.930	1.97429	275	16.6	19.2
45.940	1.97388	314	17.7	19.2
45.950	1.97347	274	16.6	19.2
45.960	1.97306	257	16.0	19.2
45.970	1.97265	303	17.4	19.2
45.980	1.97224	316	17.8	19.2
45.990	1.97183	295	17.2	19.2
46.000	1.97142	324	18.0	19.2
46.010	1.97101	285	16.9	19.2
46.020	1.97060	286	16.9	19.2
46.031	1.97019	295	17.2	19.2
46.041	1.96979	271	16.5	19.2
46.051	1.96938	277	16.6	19.2
46.061	1.96897	321	17.9	19.2
46.071	1.96856	309	17.6	19.2
46.081	1.96815	282	16.8	19.2
46.091	1.96774	290	17.0	19.2
46.101	1.96734	303	17.4	19.2
46.111	1.96693	281	16.8	19.2
46.121	1.96652	302	17.4	19.2
46.132	1.96612	316	17.8	19.2
46.142	1.96571	277	16.6	19.2
46.152	1.96530	280	16.7	19.2
46.162	1.96490	306	17.5	19.2
46.172	1.96449	282	16.8	19.2
46.182	1.96408	298	17.3	19.2
46.192	1.96368	257	16.0	19.2
46.202	1.96327	310	17.6	19.2
46.212	1.96287	282	16.8	19.2
46.222	1.96246	291	17.1	19.2
46.233	1.96206	319	17.9	19.2
46.243	1.96165	268	16.4	19.2
46.253	1.96125	288	17.0	19.2
46.263	1.96084	303	17.4	19.2
46.273	1.96044	301	17.3	19.2
46.283	1.96003	293	17.1	19.2
46.293	1.95963	317	17.8	19.2
46.303	1.95922	289	17.0	19.2
46.313	1.95882	299	17.3	19.2
46.323	1.95842	305	17.5	19.2
46.334	1.95801	287	16.9	19.2
46.344	1.95761	310	17.6	19.2
46.354	1.95721	340	18.4	19.2
46.364	1.95680	278	16.7	19.2
46.374	1.95640	291	17.1	19.2
46.384	1.95600	302	17.4	19.2
46.394	1.95560	290	17.0	19.2

46.404	1.95520	286	16.9	19.2
46.414	1.95479	274	16.6	19.2
46.424	1.95439	334	18.3	19.2
46.435	1.95399	290	17.0	19.2
46.445	1.95359	282	16.8	19.2
46.455	1.95319	259	16.1	19.2
46.465	1.95279	285	16.9	19.2
46.475	1.95239	309	17.6	19.2
46.485	1.95198	283	16.8	19.2
46.495	1.95158	274	16.6	19.2
46.505	1.95118	267	16.3	19.2
46.515	1.95078	277	16.6	19.2
46.525	1.95038	318	17.8	19.2
46.536	1.94998	297	17.2	19.2
46.546	1.94958	247	15.7	19.2
46.556	1.94919	273	16.5	19.2
46.566	1.94879	287	16.9	19.2
46.576	1.94839	293	17.1	19.2
46.586	1.94799	287	16.9	19.2
46.596	1.94759	280	16.7	19.2
46.606	1.94719	297	17.2	19.2
46.616	1.94679	264	16.2	19.2
46.626	1.94639	326	18.1	19.2
46.637	1.94600	271	16.5	19.2
46.647	1.94560	268	16.4	19.2
46.657	1.94520	270	16.4	19.2
46.667	1.94480	297	17.2	19.2
46.677	1.94441	289	17.0	19.2
46.687	1.94401	279	16.7	19.2
46.697	1.94361	267	16.3	19.2
46.707	1.94322	292	17.1	19.2
46.717	1.94282	296	17.2	19.2
46.727	1.94242	297	17.2	19.2
46.738	1.94203	277	16.6	19.2
46.748	1.94163	291	17.1	19.2
46.758	1.94123	254	15.9	19.2
46.768	1.94084	255	16.0	19.2
46.778	1.94044	265	16.3	19.2
46.788	1.94005	295	17.2	19.2
46.798	1.93965	261	16.2	19.2
46.808	1.93926	271	16.5	19.2
46.818	1.93886	278	16.7	19.2
46.828	1.93847	277	16.6	19.2
46.839	1.93807	270	16.4	19.2
46.849	1.93768	278	16.7	19.2
46.859	1.93729	282	16.8	19.2
46.869	1.93689	297	17.2	19.2
46.879	1.93650	270	16.4	19.2
46.889	1.93610	306	17.5	19.2
46.899	1.93571	269	16.4	19.2

46.909	1.93532	274	16.6	19.2
46.919	1.93493	307	17.5	19.2
46.929	1.93453	256	16.0	19.2
46.940	1.93414	289	17.0	19.2
46.950	1.93375	295	17.2	19.2
46.960	1.93335	298	17.3	19.2
46.970	1.93296	269	16.4	19.2
46.980	1.93257	276	16.6	19.2
46.990	1.93218	272	16.5	19.2
47.000	1.93179	310	17.6	19.2
47.010	1.93140	285	16.9	19.2
47.020	1.93100	292	17.1	19.2
47.030	1.93061	297	17.2	19.2
47.041	1.93022	281	16.8	19.2
47.051	1.92983	273	16.5	19.2
47.061	1.92944	281	16.8	19.2
47.071	1.92905	298	17.3	19.2
47.081	1.92866	249	15.8	19.2
47.091	1.92827	281	16.8	19.2
47.101	1.92788	292	17.1	19.2
47.111	1.92749	303	17.4	19.2
47.121	1.92710	251	15.8	19.2
47.131	1.92671	251	15.8	19.2
47.141	1.92632	260	16.1	19.2
47.152	1.92593	265	16.3	19.2
47.162	1.92554	285	16.9	19.2
47.172	1.92516	292	17.1	19.2
47.182	1.92477	242	15.6	19.2
47.192	1.92438	270	16.4	19.2
47.202	1.92399	276	16.6	19.2
47.212	1.92360	269	16.4	19.2
47.222	1.92321	270	16.4	19.2
47.232	1.92283	274	16.6	19.2
47.242	1.92244	287	16.9	19.2
47.253	1.92205	308	17.5	19.2
47.263	1.92167	332	18.2	19.2
47.273	1.92128	262	16.2	19.2
47.283	1.92089	273	16.5	19.2
47.293	1.92050	284	16.9	19.2
47.303	1.92012	290	17.0	19.2
47.313	1.91973	281	16.8	19.2
47.323	1.91935	280	16.7	19.2
47.333	1.91896	258	16.1	19.2
47.343	1.91857	281	16.8	19.2
47.354	1.91819	246	15.7	19.2
47.364	1.91780	277	16.6	19.2
47.374	1.91742	283	16.8	19.2
47.384	1.91703	282	16.8	19.2
47.394	1.91665	267	16.3	19.2
47.404	1.91626	257	16.0	19.2

47.414	1.91588	279	16.7	19.2
47.424	1.91549	281	16.8	19.2
47.434	1.91511	293	17.1	19.2
47.444	1.91473	272	16.5	19.2
47.455	1.91434	274	16.6	19.2
47.465	1.91396	243	15.6	19.2
47.475	1.91357	254	15.9	19.2
47.485	1.91319	281	16.8	19.2
47.495	1.91281	279	16.7	19.2
47.505	1.91242	285	16.9	19.2
47.515	1.91204	274	16.6	19.2
47.525	1.91166	256	16.0	19.2
47.535	1.91128	267	16.3	19.2
47.545	1.91089	262	16.2	19.2
47.556	1.91051	284	16.9	19.2
47.566	1.91013	281	16.8	19.2
47.576	1.90975	316	17.8	19.2
47.586	1.90937	267	16.3	19.2
47.596	1.90898	265	16.3	19.2
47.606	1.90860	280	16.7	19.2
47.616	1.90822	280	16.7	19.2
47.626	1.90784	263	16.2	19.2
47.636	1.90746	245	15.7	19.2
47.646	1.90708	302	17.4	19.2
47.657	1.90670	292	17.1	19.2
47.667	1.90632	286	16.9	19.2
47.677	1.90594	283	16.8	19.2
47.687	1.90556	259	16.1	19.2
47.697	1.90518	253	15.9	19.2
47.707	1.90480	261	16.2	19.2
47.717	1.90442	274	16.6	19.2
47.727	1.90404	291	17.1	19.2
47.737	1.90366	275	16.6	19.2
47.747	1.90328	265	16.3	19.2
47.758	1.90290	266	16.3	19.2
47.768	1.90252	259	16.1	19.2
47.778	1.90214	270	16.4	19.2
47.788	1.90177	239	15.5	19.2
47.798	1.90139	257	16.0	19.2
47.808	1.90101	257	16.0	19.2
47.818	1.90063	252	15.9	19.2
47.828	1.90025	239	15.5	19.2
47.838	1.89988	275	16.6	19.2
47.848	1.89950	248	15.7	19.2
47.859	1.89912	282	16.8	19.2
47.869	1.89874	243	15.6	19.2
47.879	1.89837	264	16.2	19.2
47.889	1.89799	279	16.7	19.2
47.899	1.89761	255	16.0	19.2
47.909	1.89724	263	16.2	19.2

47.919	1.89686	277	16.6	19.2
47.929	1.89649	247	15.7	19.2
47.939	1.89611	263	16.2	19.2
47.949	1.89573	264	16.2	19.2
47.960	1.89536	238	15.4	19.2
47.970	1.89498	260	16.1	19.2
47.980	1.89461	242	15.6	19.2
47.990	1.89423	273	16.5	19.2
48.000	1.89386	278	16.7	19.2
48.010	1.89348	248	15.7	19.2
48.020	1.89311	261	16.2	19.2
48.030	1.89273	260	16.1	19.2
48.040	1.89236	280	16.7	19.2
48.050	1.89198	267	16.3	19.2
48.061	1.89161	279	16.7	19.2
48.071	1.89124	257	16.0	19.2
48.081	1.89086	264	16.2	19.2
48.091	1.89049	273	16.5	19.2
48.101	1.89012	289	17.0	19.2
48.111	1.88974	271	16.5	19.2
48.121	1.88937	250	15.8	19.2
48.131	1.88900	255	16.0	19.2
48.141	1.88862	251	15.8	19.2
48.151	1.88825	251	15.8	19.2
48.162	1.88788	262	16.2	19.2
48.172	1.88751	254	15.9	19.2
48.182	1.88714	249	15.8	19.2
48.192	1.88676	285	16.9	19.2
48.202	1.88639	236	15.4	19.2
48.212	1.88602	241	15.5	19.2
48.222	1.88565	250	15.8	19.2
48.232	1.88528	252	15.9	19.2
48.242	1.88491	286	16.9	19.2
48.252	1.88454	236	15.4	19.2
48.263	1.88416	241	15.5	19.2
48.273	1.88379	256	16.0	19.2
48.283	1.88342	252	15.9	19.2
48.293	1.88305	239	15.5	19.2
48.303	1.88268	288	17.0	19.2
48.313	1.88231	289	17.0	19.2
48.323	1.88194	273	16.5	19.2
48.333	1.88157	280	16.7	19.2
48.343	1.88120	272	16.5	19.2
48.353	1.88084	267	16.3	19.2
48.364	1.88047	274	16.6	19.2
48.374	1.88010	279	16.7	19.2
48.384	1.87973	261	16.2	19.2
48.394	1.87936	267	16.3	19.2
48.404	1.87899	268	16.4	19.2
48.414	1.87862	265	16.3	19.2

48.424	1.87825	267	16.3	19.2
48.434	1.87789	276	16.6	19.2
48.444	1.87752	267	16.3	19.2
48.454	1.87715	253	15.9	19.2
48.464	1.87678	288	17.0	19.2
48.475	1.87642	258	16.1	19.2
48.485	1.87605	247	15.7	19.2
48.495	1.87568	266	16.3	19.2
48.505	1.87531	271	16.5	19.2
48.515	1.87495	262	16.2	19.2
48.525	1.87458	245	15.7	19.2
48.535	1.87421	252	15.9	19.2
48.545	1.87385	256	16.0	19.2
48.555	1.87348	269	16.4	19.2
48.565	1.87312	259	16.1	19.2
48.576	1.87275	256	16.0	19.2
48.586	1.87238	247	15.7	19.2
48.596	1.87202	253	15.9	19.2
48.606	1.87165	244	15.6	19.2
48.616	1.87129	272	16.5	19.2
48.626	1.87092	278	16.7	19.2
48.636	1.87056	253	15.9	19.2
48.646	1.87019	269	16.4	19.2
48.656	1.86983	254	15.9	19.2
48.666	1.86946	228	15.1	19.2
48.677	1.86910	262	16.2	19.2
48.687	1.86874	250	15.8	19.2
48.697	1.86837	277	16.6	19.2
48.707	1.86801	264	16.2	19.2
48.717	1.86765	275	16.6	19.2
48.727	1.86728	269	16.4	19.2
48.737	1.86692	261	16.2	19.2
48.747	1.86656	262	16.2	19.2
48.757	1.86619	257	16.0	19.2
48.767	1.86583	267	16.3	19.2
48.778	1.86547	233	15.3	19.2
48.788	1.86510	258	16.1	19.2
48.798	1.86474	275	16.6	19.2
48.808	1.86438	250	15.8	19.2
48.818	1.86402	245	15.7	19.2
48.828	1.86366	249	15.8	19.2
48.838	1.86329	268	16.4	19.2
48.848	1.86293	270	16.4	19.2
48.858	1.86257	236	15.4	19.2
48.868	1.86221	236	15.4	19.2
48.879	1.86185	259	16.1	19.2
48.889	1.86149	255	16.0	19.2
48.899	1.86113	254	15.9	19.2
48.909	1.86077	243	15.6	19.2
48.919	1.86040	257	16.0	19.2

48.929	1.86004	265	16.3	19.2
48.939	1.85968	223	14.9	19.2
48.949	1.85932	269	16.4	19.2
48.959	1.85896	249	15.8	19.2
48.969	1.85860	270	16.4	19.2
48.980	1.85825	235	15.3	19.2
48.990	1.85789	286	16.9	19.2
49.000	1.85753	241	15.5	19.2
49.010	1.85717	250	15.8	19.2
49.020	1.85681	268	16.4	19.2
49.030	1.85645	275	16.6	19.2
49.040	1.85609	273	16.5	19.2
49.050	1.85573	264	16.2	19.2
49.060	1.85537	218	14.8	19.2
49.070	1.85502	262	16.2	19.2
49.081	1.85466	261	16.2	19.2
49.091	1.85430	244	15.6	19.2
49.101	1.85394	235	15.3	19.2
49.111	1.85358	265	16.3	19.2
49.121	1.85323	278	16.7	19.2
49.131	1.85287	254	15.9	19.2
49.141	1.85251	251	15.8	19.2
49.151	1.85216	251	15.8	19.2
49.161	1.85180	250	15.8	19.2
49.171	1.85144	252	15.9	19.2
49.182	1.85108	275	16.6	19.2
49.192	1.85073	229	15.1	19.2
49.202	1.85037	267	16.3	19.2
49.212	1.85002	252	15.9	19.2
49.222	1.84966	291	17.1	19.2
49.232	1.84930	267	16.3	19.2
49.242	1.84895	264	16.2	19.2
49.252	1.84859	245	15.7	19.2
49.262	1.84824	282	16.8	19.2
49.272	1.84788	251	15.8	19.2
49.283	1.84753	251	15.8	19.2
49.293	1.84717	264	16.2	19.2
49.303	1.84682	239	15.5	19.2
49.313	1.84646	253	15.9	19.2
49.323	1.84611	256	16.0	19.2
49.333	1.84575	270	16.4	19.2
49.343	1.84540	260	16.1	19.2
49.353	1.84505	257	16.0	19.2
49.363	1.84469	243	15.6	19.2
49.373	1.84434	225	15.0	19.2
49.384	1.84399	233	15.3	19.2
49.394	1.84363	245	15.7	19.2
49.404	1.84328	276	16.6	19.2
49.414	1.84293	259	16.1	19.2
49.424	1.84257	276	16.6	19.2

49.434	1.84222	246	15.7	19.2
49.444	1.84187	273	16.5	19.2
49.454	1.84151	251	15.8	19.2
49.464	1.84116	254	15.9	19.2
49.474	1.84081	237	15.4	19.2
49.485	1.84046	279	16.7	19.2
49.495	1.84011	261	16.2	19.2
49.505	1.83975	248	15.7	19.2
49.515	1.83940	284	16.9	19.2
49.525	1.83905	251	15.8	19.2
49.535	1.83870	234	15.3	19.2
49.545	1.83835	294	17.1	19.2
49.555	1.83800	257	16.0	19.2
49.565	1.83765	244	15.6	19.2
49.575	1.83730	255	16.0	19.2
49.586	1.83695	246	15.7	19.2
49.596	1.83660	247	15.7	19.2
49.606	1.83625	268	16.4	19.2
49.616	1.83590	249	15.8	19.2
49.626	1.83555	275	16.6	19.2
49.636	1.83520	235	15.3	19.2
49.646	1.83485	233	15.3	19.2
49.656	1.83450	250	15.8	19.2
49.666	1.83415	262	16.2	19.2
49.676	1.83380	244	15.6	19.2
49.687	1.83345	251	15.8	19.2
49.697	1.83310	261	16.2	19.2
49.707	1.83275	266	16.3	19.2
49.717	1.83240	235	15.3	19.2
49.727	1.83205	271	16.5	19.2
49.737	1.83171	243	15.6	19.2
49.747	1.83136	230	15.2	19.2
49.757	1.83101	214	14.6	19.2
49.767	1.83066	240	15.5	19.2
49.777	1.83031	228	15.1	19.2
49.787	1.82997	252	15.9	19.2
49.798	1.82962	272	16.5	19.2
49.808	1.82927	259	16.1	19.2
49.818	1.82892	256	16.0	19.2
49.828	1.82858	261	16.2	19.2
49.838	1.82823	254	15.9	19.2
49.848	1.82788	228	15.1	19.2
49.858	1.82754	283	16.8	19.2
49.868	1.82719	258	16.1	19.2
49.878	1.82684	245	15.7	19.2
49.888	1.82650	244	15.6	19.2
49.899	1.82615	233	15.3	19.2
49.909	1.82581	267	16.3	19.2
49.919	1.82546	247	15.7	19.2
49.929	1.82511	228	15.1	19.2

49.939	1.82477	228	15.1	19.2
49.949	1.82442	263	16.2	19.2
49.959	1.82408	241	15.5	19.2
49.969	1.82373	261	16.2	19.2
49.979	1.82339	243	15.6	19.2
49.989	1.82304	228	15.1	19.2
50.000	1.82270	241	15.5	19.2
50.010	1.82235	232	15.2	19.2
50.020	1.82201	243	15.6	19.2
50.030	1.82167	221	14.9	19.2
50.040	1.82132	260	16.1	19.2
50.050	1.82098	261	16.2	19.2
50.060	1.82063	221	14.9	19.2
50.070	1.82029	250	15.8	19.2
50.080	1.81995	240	15.5	19.2
50.090	1.81960	241	15.5	19.2
50.101	1.81926	264	16.2	19.2
50.111	1.81892	235	15.3	19.2
50.121	1.81858	243	15.6	19.2
50.131	1.81823	237	15.4	19.2
50.141	1.81789	238	15.4	19.2
50.151	1.81755	235	15.3	19.2
50.161	1.81721	223	14.9	19.2
50.171	1.81686	233	15.3	19.2
50.181	1.81652	207	14.4	19.2
50.191	1.81618	232	15.2	19.2
50.202	1.81584	250	15.8	19.2
50.212	1.81550	247	15.7	19.2
50.222	1.81516	253	15.9	19.2
50.232	1.81481	247	15.7	19.2
50.242	1.81447	229	15.1	19.2
50.252	1.81413	249	15.8	19.2
50.262	1.81379	263	16.2	19.2
50.272	1.81345	246	15.7	19.2
50.282	1.81311	261	16.2	19.2
50.292	1.81277	226	15.0	19.2
50.303	1.81243	249	15.8	19.2
50.313	1.81209	265	16.3	19.2
50.323	1.81175	270	16.4	19.2
50.333	1.81141	265	16.3	19.2
50.343	1.81107	243	15.6	19.2
50.353	1.81073	257	16.0	19.2
50.363	1.81039	240	15.5	19.2
50.373	1.81005	226	15.0	19.2
50.383	1.80971	249	15.8	19.2
50.393	1.80937	225	15.0	19.2
50.404	1.80903	225	15.0	19.2
50.414	1.80870	234	15.3	19.2
50.424	1.80836	231	15.2	19.2
50.434	1.80802	274	16.6	19.2

50.444	1.80768	270	16.4	19.2
50.454	1.80734	265	16.3	19.2
50.464	1.80700	254	15.9	19.2
50.474	1.80667	257	16.0	19.2
50.484	1.80633	268	16.4	19.2
50.494	1.80599	245	15.7	19.2
50.505	1.80565	255	16.0	19.2
50.515	1.80532	245	15.7	19.2
50.525	1.80498	247	15.7	19.2
50.535	1.80464	245	15.7	19.2
50.545	1.80430	212	14.6	19.2
50.555	1.80397	267	16.3	19.2
50.565	1.80363	256	16.0	19.2
50.575	1.80330	269	16.4	19.2
50.585	1.80296	203	14.2	19.2
50.595	1.80262	262	16.2	19.2
50.606	1.80229	247	15.7	19.2
50.616	1.80195	248	15.7	19.2
50.626	1.80161	252	15.9	19.2
50.636	1.80128	259	16.1	19.2
50.646	1.80094	247	15.7	19.2
50.656	1.80061	239	15.5	19.2
50.666	1.80027	247	15.7	19.2
50.676	1.79994	287	16.9	19.2
50.686	1.79960	225	15.0	19.2
50.696	1.79927	250	15.8	19.2
50.707	1.79893	241	15.5	19.2
50.717	1.79860	243	15.6	19.2
50.727	1.79826	236	15.4	19.2
50.737	1.79793	202	14.2	19.2
50.747	1.79760	257	16.0	19.2
50.757	1.79726	269	16.4	19.2
50.767	1.79693	245	15.7	19.2
50.777	1.79659	256	16.0	19.2
50.787	1.79626	269	16.4	19.2
50.797	1.79593	273	16.5	19.2
50.808	1.79559	265	16.3	19.2
50.818	1.79526	253	15.9	19.2
50.828	1.79493	248	15.7	19.2
50.838	1.79460	249	15.8	19.2
50.848	1.79426	240	15.5	19.2
50.858	1.79393	258	16.1	19.2
50.868	1.79360	268	16.4	19.2
50.878	1.79327	251	15.8	19.2
50.888	1.79293	265	16.3	19.2
50.898	1.79260	269	16.4	19.2
50.909	1.79227	271	16.5	19.2
50.919	1.79194	253	15.9	19.2
50.929	1.79161	249	15.8	19.2
50.939	1.79127	266	16.3	19.2

50.949	1.79094	275	16.6	19.2
50.959	1.79061	275	16.6	19.2
50.969	1.79028	245	15.7	19.2
50.979	1.78995	257	16.0	19.2
50.989	1.78962	266	16.3	19.2
50.999	1.78929	261	16.2	19.2
51.010	1.78896	283	16.8	19.2
51.020	1.78863	265	16.3	19.2
51.030	1.78830	276	16.6	19.2
51.040	1.78797	258	16.1	19.2
51.050	1.78764	247	15.7	19.2
51.060	1.78731	248	15.7	19.2
51.070	1.78698	280	16.7	19.2
51.080	1.78665	246	15.7	19.2
51.090	1.78632	259	16.1	19.2
51.100	1.78599	278	16.7	19.2
51.110	1.78566	245	15.7	19.2
51.121	1.78533	245	15.7	19.2
51.131	1.78500	287	16.9	19.2
51.141	1.78467	229	15.1	19.2
51.151	1.78434	253	15.9	19.2
51.161	1.78402	251	15.8	19.2
51.171	1.78369	255	16.0	19.2
51.181	1.78336	251	15.8	19.2
51.191	1.78303	271	16.5	19.2
51.201	1.78270	231	15.2	19.2
51.211	1.78238	239	15.5	19.2
51.222	1.78205	249	15.8	19.2
51.232	1.78172	287	16.9	19.2
51.242	1.78139	236	15.4	19.2
51.252	1.78106	235	15.3	19.2
51.262	1.78074	246	15.7	19.2
51.272	1.78041	264	16.2	19.2
51.282	1.78008	229	15.1	19.2
51.292	1.77976	242	15.6	19.2
51.302	1.77943	239	15.5	19.2
51.312	1.77910	235	15.3	19.2
51.323	1.77878	245	15.7	19.2
51.333	1.77845	236	15.4	19.2
51.343	1.77813	219	14.8	19.2
51.353	1.77780	224	15.0	19.2
51.363	1.77747	243	15.6	19.2
51.373	1.77715	269	16.4	19.2
51.383	1.77682	243	15.6	19.2
51.393	1.77650	234	15.3	19.2
51.403	1.77617	257	16.0	19.2
51.413	1.77585	245	15.7	19.2
51.424	1.77552	252	15.9	19.2
51.434	1.77520	226	15.0	19.2
51.444	1.77487	229	15.1	19.2

51.454	1.77455	230	15.2	19.2
51.464	1.77422	262	16.2	19.2
51.474	1.77390	205	14.3	19.2
51.484	1.77357	243	15.6	19.2
51.494	1.77325	257	16.0	19.2
51.504	1.77293	230	15.2	19.2
51.514	1.77260	217	14.7	19.2
51.525	1.77228	232	15.2	19.2
51.535	1.77195	252	15.9	19.2
51.545	1.77163	238	15.4	19.2
51.555	1.77131	216	14.7	19.2
51.565	1.77098	224	15.0	19.2
51.575	1.77066	214	14.6	19.2
51.585	1.77034	247	15.7	19.2
51.595	1.77002	227	15.1	19.2
51.605	1.76969	254	15.9	19.2
51.615	1.76937	225	15.0	19.2
51.626	1.76905	256	16.0	19.2
51.636	1.76873	224	15.0	19.2
51.646	1.76840	250	15.8	19.2
51.656	1.76808	229	15.1	19.2
51.666	1.76776	232	15.2	19.2
51.676	1.76744	210	14.5	19.2
51.686	1.76712	243	15.6	19.2
51.696	1.76680	217	14.7	19.2
51.706	1.76647	234	15.3	19.2
51.716	1.76615	226	15.0	19.2
51.727	1.76583	239	15.5	19.2
51.737	1.76551	259	16.1	19.2
51.747	1.76519	245	15.7	19.2
51.757	1.76487	218	14.8	19.2
51.767	1.76455	224	15.0	19.2
51.777	1.76423	237	15.4	19.2
51.787	1.76391	206	14.4	19.2
51.797	1.76359	244	15.6	19.2
51.807	1.76327	224	15.0	19.2
51.817	1.76295	218	14.8	19.2
51.828	1.76263	260	16.1	19.2
51.838	1.76231	231	15.2	19.2
51.848	1.76199	228	15.1	19.2
51.858	1.76167	223	14.9	19.2
51.868	1.76135	226	15.0	19.2
51.878	1.76103	221	14.9	19.2
51.888	1.76071	242	15.6	19.2
51.898	1.76039	254	15.9	19.2
51.908	1.76007	236	15.4	19.2
51.918	1.75976	242	15.6	19.2
51.929	1.75944	214	14.6	19.2
51.939	1.75912	203	14.2	19.2
51.949	1.75880	209	14.5	19.2

51.959	1.75848	233	15.3	19.2
51.969	1.75816	240	15.5	19.2
51.979	1.75785	237	15.4	19.2
51.989	1.75753	243	15.6	19.2
51.999	1.75721	236	15.4	19.2
52.009	1.75689	250	15.8	19.2
52.019	1.75658	241	15.5	19.2
52.030	1.75626	244	15.6	19.2
52.040	1.75594	210	14.5	19.2
52.050	1.75563	223	14.9	19.2
52.060	1.75531	231	15.2	19.2
52.070	1.75499	217	14.7	19.2
52.080	1.75467	239	15.5	19.2
52.090	1.75436	245	15.7	19.2
52.100	1.75404	240	15.5	19.2
52.110	1.75373	208	14.4	19.2
52.120	1.75341	236	15.4	19.2
52.131	1.75309	211	14.5	19.2
52.141	1.75278	201	14.2	19.2
52.151	1.75246	235	15.3	19.2
52.161	1.75215	243	15.6	19.2
52.171	1.75183	237	15.4	19.2
52.181	1.75152	231	15.2	19.2
52.191	1.75120	248	15.7	19.2
52.201	1.75089	255	16.0	19.2
52.211	1.75057	234	15.3	19.2
52.221	1.75026	226	15.0	19.2
52.232	1.74994	237	15.4	19.2
52.242	1.74963	226	15.0	19.2
52.252	1.74931	247	15.7	19.2
52.262	1.74900	243	15.6	19.2
52.272	1.74868	230	15.2	19.2
52.282	1.74837	232	15.2	19.2
52.292	1.74806	225	15.0	19.2
52.302	1.74774	226	15.0	19.2
52.312	1.74743	218	14.8	19.2
52.322	1.74712	224	15.0	19.2
52.333	1.74680	193	13.9	19.2
52.343	1.74649	240	15.5	19.2
52.353	1.74618	240	15.5	19.2
52.363	1.74586	233	15.3	19.2
52.373	1.74555	233	15.3	19.2
52.383	1.74524	253	15.9	19.2
52.393	1.74492	237	15.4	19.2
52.403	1.74461	239	15.5	19.2
52.413	1.74430	243	15.6	19.2
52.423	1.74399	217	14.7	19.2
52.433	1.74367	247	15.7	19.2
52.444	1.74336	251	15.8	19.2
52.454	1.74305	228	15.1	19.2

52.464	1.74274	222	14.9	19.2
52.474	1.74243	248	15.7	19.2
52.484	1.74212	220	14.8	19.2
52.494	1.74180	207	14.4	19.2
52.504	1.74149	237	15.4	19.2
52.514	1.74118	226	15.0	19.2
52.524	1.74087	221	14.9	19.2
52.534	1.74056	216	14.7	19.2
52.545	1.74025	247	15.7	19.2
52.555	1.73994	242	15.6	19.2
52.565	1.73963	222	14.9	19.2
52.575	1.73932	214	14.6	19.2
52.585	1.73901	224	15.0	19.2
52.595	1.73870	222	14.9	19.2
52.605	1.73839	243	15.6	19.2
52.615	1.73808	221	14.9	19.2
52.625	1.73777	255	16.0	19.2
52.635	1.73746	208	14.4	19.2
52.646	1.73715	204	14.3	19.2
52.656	1.73684	205	14.3	19.2
52.666	1.73653	230	15.2	19.2
52.676	1.73622	214	14.6	19.2
52.686	1.73591	252	15.9	19.2
52.696	1.73560	250	15.8	19.2
52.706	1.73529	217	14.7	19.2
52.716	1.73499	229	15.1	19.2
52.726	1.73468	226	15.0	19.2
52.736	1.73437	202	14.2	19.2
52.747	1.73406	226	15.0	19.2
52.757	1.73375	206	14.4	19.2
52.767	1.73344	220	14.8	19.2
52.777	1.73314	214	14.6	19.2
52.787	1.73283	210	14.5	19.2
52.797	1.73252	219	14.8	19.2
52.807	1.73221	224	15.0	19.2
52.817	1.73191	250	15.8	19.2
52.827	1.73160	227	15.1	19.2
52.837	1.73129	235	15.3	19.2
52.848	1.73098	245	15.7	19.2
52.858	1.73068	220	14.8	19.2
52.868	1.73037	219	14.8	19.2
52.878	1.73006	223	14.9	19.2
52.888	1.72976	218	14.8	19.2
52.898	1.72945	221	14.9	19.2
52.908	1.72914	241	15.5	19.2
52.918	1.72884	229	15.1	19.2
52.928	1.72853	247	15.7	19.2
52.938	1.72823	227	15.1	19.2
52.949	1.72792	211	14.5	19.2
52.959	1.72761	228	15.1	19.2

52.969	1.72731	224	15.0	19.2
52.979	1.72700	233	15.3	19.2
52.989	1.72670	209	14.5	19.2
52.999	1.72639	198	14.1	19.2
53.009	1.72609	193	13.9	19.2
53.019	1.72578	229	15.1	19.2
53.029	1.72548	253	15.9	19.2
53.039	1.72517	224	15.0	19.2
53.050	1.72487	235	15.3	19.2
53.060	1.72456	221	14.9	19.2
53.070	1.72426	240	15.5	19.2
53.080	1.72396	247	15.7	19.2
53.090	1.72365	204	14.3	19.2
53.100	1.72335	255	16.0	19.2
53.110	1.72304	221	14.9	19.2
53.120	1.72274	225	15.0	19.2
53.130	1.72244	216	14.7	19.2
53.140	1.72213	211	14.5	19.2
53.151	1.72183	240	15.5	19.2
53.161	1.72153	222	14.9	19.2
53.171	1.72122	232	15.2	19.2
53.181	1.72092	241	15.5	19.2
53.191	1.72062	222	14.9	19.2
53.201	1.72031	256	16.0	19.2
53.211	1.72001	230	15.2	19.2
53.221	1.71971	256	16.0	19.2
53.231	1.71941	232	15.2	19.2
53.241	1.71910	223	14.9	19.2
53.252	1.71880	217	14.7	19.2
53.262	1.71850	228	15.1	19.2
53.272	1.71820	226	15.0	19.2
53.282	1.71790	229	15.1	19.2
53.292	1.71759	227	15.1	19.2
53.302	1.71729	196	14.0	19.2
53.312	1.71699	217	14.7	19.2
53.322	1.71669	241	15.5	19.2
53.332	1.71639	216	14.7	19.2
53.342	1.71609	239	15.5	19.2
53.353	1.71579	229	15.1	19.2
53.363	1.71548	212	14.6	19.2
53.373	1.71518	214	14.6	19.2
53.383	1.71488	215	14.7	19.2
53.393	1.71458	200	14.1	19.2
53.403	1.71428	227	15.1	19.2
53.413	1.71398	223	14.9	19.2
53.423	1.71368	233	15.3	19.2
53.433	1.71338	235	15.3	19.2
53.443	1.71308	218	14.8	19.2
53.454	1.71278	248	15.7	19.2
53.464	1.71248	211	14.5	19.2

53.474	1.71218	221	14.9	19.2
53.484	1.71188	234	15.3	19.2
53.494	1.71158	220	14.8	19.2
53.504	1.71128	236	15.4	19.2
53.514	1.71099	217	14.7	19.2
53.524	1.71069	192	13.9	19.2
53.534	1.71039	215	14.7	19.2
53.544	1.71009	220	14.8	19.2
53.555	1.70979	206	14.4	19.2
53.565	1.70949	244	15.6	19.2
53.575	1.70919	229	15.1	19.2
53.585	1.70889	230	15.2	19.2
53.595	1.70860	238	15.4	19.2
53.605	1.70830	229	15.1	19.2
53.615	1.70800	233	15.3	19.2
53.625	1.70770	231	15.2	19.2
53.635	1.70740	230	15.2	19.2
53.645	1.70711	238	15.4	19.2
53.656	1.70681	218	14.8	19.2
53.666	1.70651	216	14.7	19.2
53.676	1.70622	206	14.4	19.2
53.686	1.70592	218	14.8	19.2
53.696	1.70562	186	13.6	19.2
53.706	1.70532	232	15.2	19.2
53.716	1.70503	212	14.6	19.2
53.726	1.70473	210	14.5	19.2
53.736	1.70443	209	14.5	19.2
53.746	1.70414	231	15.2	19.2
53.756	1.70384	198	14.1	19.2
53.767	1.70355	199	14.1	19.2
53.777	1.70325	225	15.0	19.2
53.787	1.70295	227	15.1	19.2
53.797	1.70266	227	15.1	19.2
53.807	1.70236	208	14.4	19.2
53.817	1.70207	214	14.6	19.2
53.827	1.70177	205	14.3	19.2
53.837	1.70147	252	15.9	19.2
53.847	1.70118	210	14.5	19.2
53.857	1.70088	219	14.8	19.2
53.868	1.70059	207	14.4	19.2
53.878	1.70029	242	15.6	19.2
53.888	1.70000	211	14.5	19.2
53.898	1.69970	227	15.1	19.2
53.908	1.69941	208	14.4	19.2
53.918	1.69912	227	15.1	19.2
53.928	1.69882	238	15.4	19.2
53.938	1.69853	209	14.5	19.2
53.948	1.69823	211	14.5	19.2
53.958	1.69794	238	15.4	19.2
53.969	1.69765	236	15.4	19.2

53.979	1.69735	222	14.9	19.2
53.989	1.69706	214	14.6	19.2
53.999	1.69676	242	15.6	19.2
54.009	1.69647	213	14.6	19.2
54.019	1.69618	241	15.5	19.2
54.029	1.69588	219	14.8	19.2
54.039	1.69559	216	14.7	19.2
54.049	1.69530	232	15.2	19.2
54.059	1.69501	194	13.9	19.2
54.070	1.69471	186	13.6	19.2
54.080	1.69442	189	13.7	19.2
54.090	1.69413	212	14.6	19.2
54.100	1.69383	209	14.5	19.2
54.110	1.69354	249	15.8	19.2
54.120	1.69325	236	15.4	19.2
54.130	1.69296	244	15.6	19.2
54.140	1.69267	207	14.4	19.2
54.150	1.69237	202	14.2	19.2
54.160	1.69208	222	14.9	19.2
54.171	1.69179	226	15.0	19.2
54.181	1.69150	257	16.0	19.2
54.191	1.69121	205	14.3	19.2
54.201	1.69092	224	15.0	19.2
54.211	1.69063	194	13.9	19.2
54.221	1.69033	214	14.6	19.2
54.231	1.69004	216	14.7	19.2
54.241	1.68975	218	14.8	19.2
54.251	1.68946	219	14.8	19.2
54.261	1.68917	201	14.2	19.2
54.272	1.68888	202	14.2	19.2
54.282	1.68859	258	16.1	19.2
54.292	1.68830	217	14.7	19.2
54.302	1.68801	180	13.4	19.2
54.312	1.68772	210	14.5	19.2
54.322	1.68743	228	15.1	19.2
54.332	1.68714	213	14.6	19.2
54.342	1.68685	224	15.0	19.2
54.352	1.68656	240	15.5	19.2
54.362	1.68627	212	14.6	19.2
54.373	1.68598	200	14.1	19.2
54.383	1.68569	241	15.5	19.2
54.393	1.68540	219	14.8	19.2
54.403	1.68512	198	14.1	19.2
54.413	1.68483	211	14.5	19.2
54.423	1.68454	191	13.8	19.2
54.433	1.68425	197	14.0	19.2
54.443	1.68396	206	14.4	19.2
54.453	1.68367	208	14.4	19.2
54.463	1.68338	242	15.6	19.2
54.474	1.68310	215	14.7	19.2

54.484	1.68281	184	13.6	19.2
54.494	1.68252	204	14.3	19.2
54.504	1.68223	202	14.2	19.2
54.514	1.68194	213	14.6	19.2
54.524	1.68166	224	15.0	19.2
54.534	1.68137	212	14.6	19.2
54.544	1.68108	217	14.7	19.2
54.554	1.68079	212	14.6	19.2
54.564	1.68051	215	14.7	19.2
54.575	1.68022	171	13.1	19.2
54.585	1.67993	217	14.7	19.2
54.595	1.67965	225	15.0	19.2
54.605	1.67936	211	14.5	19.2
54.615	1.67907	223	14.9	19.2
54.625	1.67879	199	14.1	19.2
54.635	1.67850	226	15.0	19.2
54.645	1.67821	211	14.5	19.2
54.655	1.67793	207	14.4	19.2
54.665	1.67764	218	14.8	19.2
54.676	1.67735	244	15.6	19.2
54.686	1.67707	215	14.7	19.2
54.696	1.67678	210	14.5	19.2
54.706	1.67650	204	14.3	19.2
54.716	1.67621	214	14.6	19.2
54.726	1.67593	211	14.5	19.2
54.736	1.67564	188	13.7	19.2
54.746	1.67536	203	14.2	19.2
54.756	1.67507	203	14.2	19.2
54.766	1.67479	236	15.4	19.2
54.777	1.67450	221	14.9	19.2
54.787	1.67422	196	14.0	19.2
54.797	1.67393	216	14.7	19.2
54.807	1.67365	196	14.0	19.2
54.817	1.67336	204	14.3	19.2
54.827	1.67308	226	15.0	19.2
54.837	1.67279	193	13.9	19.2
54.847	1.67251	212	14.6	19.2
54.857	1.67222	203	14.2	19.2
54.867	1.67194	202	14.2	19.2
54.878	1.67166	204	14.3	19.2
54.888	1.67137	210	14.5	19.2
54.898	1.67109	236	15.4	19.2
54.908	1.67081	202	14.2	19.2
54.918	1.67052	205	14.3	19.2
54.928	1.67024	186	13.6	19.2
54.938	1.66996	198	14.1	19.2
54.948	1.66967	191	13.8	19.2
54.958	1.66939	208	14.4	19.2
54.968	1.66911	217	14.7	19.2
54.978	1.66883	227	15.1	19.2

54.989	1.66854	211	14.5	19.2
54.999	1.66826	194	13.9	19.2
55.009	1.66798	206	14.4	19.2
55.019	1.66770	193	13.9	19.2
55.029	1.66741	207	14.4	19.2
55.039	1.66713	225	15.0	19.2
55.049	1.66685	195	14.0	19.2
55.059	1.66657	200	14.1	19.2
55.069	1.66629	215	14.7	19.2
55.079	1.66600	207	14.4	19.2
55.090	1.66572	213	14.6	19.2
55.100	1.66544	201	14.2	19.2
55.110	1.66516	220	14.8	19.2
55.120	1.66488	197	14.0	19.2
55.130	1.66460	222	14.9	19.2
55.140	1.66432	203	14.2	19.2
55.150	1.66404	200	14.1	19.2
55.160	1.66375	205	14.3	19.2
55.170	1.66347	209	14.5	19.2
55.180	1.66319	210	14.5	19.2
55.191	1.66291	184	13.6	19.2
55.201	1.66263	192	13.9	19.2
55.211	1.66235	213	14.6	19.2
55.221	1.66207	191	13.8	19.2
55.231	1.66179	228	15.1	19.2
55.241	1.66151	206	14.4	19.2
55.251	1.66123	189	13.7	19.2
55.261	1.66095	199	14.1	19.2
55.271	1.66067	212	14.6	19.2
55.281	1.66039	219	14.8	19.2
55.292	1.66011	190	13.8	19.2
55.302	1.65984	206	14.4	19.2
55.312	1.65956	218	14.8	19.2
55.322	1.65928	235	15.3	19.2
55.332	1.65900	207	14.4	19.2
55.342	1.65872	212	14.6	19.2
55.352	1.65844	218	14.8	19.2
55.362	1.65816	206	14.4	19.2
55.372	1.65788	212	14.6	19.2
55.382	1.65761	204	14.3	19.2
55.393	1.65733	238	15.4	19.2
55.403	1.65705	216	14.7	19.2
55.413	1.65677	214	14.6	19.2
55.423	1.65649	202	14.2	19.2
55.433	1.65621	206	14.4	19.2
55.443	1.65594	226	15.0	19.2
55.453	1.65566	211	14.5	19.2
55.463	1.65538	232	15.2	19.2
55.473	1.65510	228	15.1	19.2
55.483	1.65483	200	14.1	19.2

55.494	1.65455	233	15.3	19.2
55.504	1.65427	235	15.3	19.2
55.514	1.65400	212	14.6	19.2
55.524	1.65372	194	13.9	19.2
55.534	1.65344	205	14.3	19.2
55.544	1.65316	176	13.3	19.2
55.554	1.65289	202	14.2	19.2
55.564	1.65261	220	14.8	19.2
55.574	1.65234	212	14.6	19.2
55.584	1.65206	207	14.4	19.2
55.595	1.65178	210	14.5	19.2
55.605	1.65151	203	14.2	19.2
55.615	1.65123	197	14.0	19.2
55.625	1.65095	195	14.0	19.2
55.635	1.65068	199	14.1	19.2
55.645	1.65040	199	14.1	19.2
55.655	1.65013	187	13.7	19.2
55.665	1.64985	220	14.8	19.2
55.675	1.64958	198	14.1	19.2
55.685	1.64930	207	14.4	19.2
55.696	1.64903	190	13.8	19.2
55.706	1.64875	215	14.7	19.2
55.716	1.64848	222	14.9	19.2
55.726	1.64820	229	15.1	19.2
55.736	1.64793	199	14.1	19.2
55.746	1.64765	194	13.9	19.2
55.756	1.64738	218	14.8	19.2
55.766	1.64710	215	14.7	19.2
55.776	1.64683	207	14.4	19.2
55.786	1.64656	201	14.2	19.2
55.797	1.64628	192	13.9	19.2
55.807	1.64601	217	14.7	19.2
55.817	1.64573	208	14.4	19.2
55.827	1.64546	199	14.1	19.2
55.837	1.64519	193	13.9	19.2
55.847	1.64491	210	14.5	19.2
55.857	1.64464	171	13.1	19.2
55.867	1.64437	201	14.2	19.2
55.877	1.64409	211	14.5	19.2
55.887	1.64382	203	14.2	19.2
55.898	1.64355	214	14.6	19.2
55.908	1.64327	208	14.4	19.2
55.918	1.64300	223	14.9	19.2
55.928	1.64273	223	14.9	19.2
55.938	1.64245	211	14.5	19.2
55.948	1.64218	196	14.0	19.2
55.958	1.64191	202	14.2	19.2
55.968	1.64164	190	13.8	19.2
55.978	1.64136	237	15.4	19.2
55.988	1.64109	184	13.6	19.2

55.999	1.64082	190	13.8	19.2
56.009	1.64055	184	13.6	19.2
56.019	1.64028	200	14.1	19.2
56.029	1.64001	232	15.2	19.2
56.039	1.63973	214	14.6	19.2
56.049	1.63946	177	13.3	19.2
56.059	1.63919	203	14.2	19.2
56.069	1.63892	202	14.2	19.2
56.079	1.63865	185	13.6	19.2
56.089	1.63838	192	13.9	19.2
56.100	1.63811	191	13.8	19.2
56.110	1.63784	190	13.8	19.2
56.120	1.63756	208	14.4	19.2
56.130	1.63729	218	14.8	19.2
56.140	1.63702	191	13.8	19.2
56.150	1.63675	196	14.0	19.2
56.160	1.63648	218	14.8	19.2
56.170	1.63621	209	14.5	19.2
56.180	1.63594	194	13.9	19.2
56.190	1.63567	217	14.7	19.2
56.201	1.63540	203	14.2	19.2
56.211	1.63513	182	13.5	19.2
56.221	1.63486	206	14.4	19.2
56.231	1.63459	205	14.3	19.2
56.241	1.63432	192	13.9	19.2
56.251	1.63405	234	15.3	19.2
56.261	1.63378	192	13.9	19.2
56.271	1.63351	203	14.2	19.2
56.281	1.63325	238	15.4	19.2
56.291	1.63298	210	14.5	19.2
56.301	1.63271	185	13.6	19.2
56.312	1.63244	212	14.6	19.2
56.322	1.63217	192	13.9	19.2
56.332	1.63190	215	14.7	19.2
56.342	1.63163	214	14.6	19.2
56.352	1.63136	213	14.6	19.2
56.362	1.63110	181	13.5	19.2
56.372	1.63083	211	14.5	19.2
56.382	1.63056	212	14.6	19.2
56.392	1.63029	212	14.6	19.2
56.402	1.63002	196	14.0	19.2
56.413	1.62976	224	15.0	19.2
56.423	1.62949	194	13.9	19.2
56.433	1.62922	197	14.0	19.2
56.443	1.62895	214	14.6	19.2
56.453	1.62868	195	14.0	19.2
56.463	1.62842	198	14.1	19.2
56.473	1.62815	195	14.0	19.2
56.483	1.62788	186	13.6	19.2
56.493	1.62762	210	14.5	19.2

56.503	1.62735	181	13.5	19.2
56.514	1.62708	212	14.6	19.2
56.524	1.62682	193	13.9	19.2
56.534	1.62655	185	13.6	19.2
56.544	1.62628	205	14.3	19.2
56.554	1.62602	222	14.9	19.2
56.564	1.62575	193	13.9	19.2
56.574	1.62548	213	14.6	19.2
56.584	1.62522	222	14.9	19.2
56.594	1.62495	202	14.2	19.2
56.604	1.62469	187	13.7	19.2
56.615	1.62442	188	13.7	19.2
56.625	1.62415	196	14.0	19.2
56.635	1.62389	212	14.6	19.2
56.645	1.62362	209	14.5	19.2
56.655	1.62336	186	13.6	19.2
56.665	1.62309	187	13.7	19.2
56.675	1.62283	204	14.3	19.2
56.685	1.62256	202	14.2	19.2
56.695	1.62230	192	13.9	19.2
56.705	1.62203	207	14.4	19.2
56.716	1.62177	202	14.2	19.2
56.726	1.62150	199	14.1	19.2
56.736	1.62124	232	15.2	19.2
56.746	1.62097	167	12.9	19.2
56.756	1.62071	197	14.0	19.2
56.766	1.62044	173	13.2	19.2
56.776	1.62018	194	13.9	19.2
56.786	1.61992	186	13.6	19.2
56.796	1.61965	209	14.5	19.2
56.806	1.61939	200	14.1	19.2
56.817	1.61912	200	14.1	19.2
56.827	1.61886	218	14.8	19.2
56.837	1.61860	187	13.7	19.2
56.847	1.61833	199	14.1	19.2
56.857	1.61807	191	13.8	19.2
56.867	1.61781	220	14.8	19.2
56.877	1.61754	217	14.7	19.2
56.887	1.61728	220	14.8	19.2
56.897	1.61702	212	14.6	19.2
56.907	1.61675	206	14.4	19.2
56.918	1.61649	217	14.7	19.2
56.928	1.61623	185	13.6	19.2
56.938	1.61596	200	14.1	19.2
56.948	1.61570	222	14.9	19.2
56.958	1.61544	208	14.4	19.2
56.968	1.61518	172	13.1	19.2
56.978	1.61491	206	14.4	19.2
56.988	1.61465	210	14.5	19.2
56.998	1.61439	208	14.4	19.2

57.008	1.61413	189	13.7	19.2
57.019	1.61387	212	14.6	19.2
57.029	1.61360	230	15.2	19.2
57.039	1.61334	221	14.9	19.2
57.049	1.61308	209	14.5	19.2
57.059	1.61282	189	13.7	19.2
57.069	1.61256	196	14.0	19.2
57.079	1.61230	204	14.3	19.2
57.089	1.61204	200	14.1	19.2
57.099	1.61177	183	13.5	19.2
57.109	1.61151	217	14.7	19.2
57.120	1.61125	218	14.8	19.2
57.130	1.61099	226	15.0	19.2
57.140	1.61073	189	13.7	19.2
57.150	1.61047	195	14.0	19.2
57.160	1.61021	215	14.7	19.2
57.170	1.60995	217	14.7	19.2
57.180	1.60969	205	14.3	19.2
57.190	1.60943	234	15.3	19.2
57.200	1.60917	197	14.0	19.2
57.210	1.60891	200	14.1	19.2
57.221	1.60865	183	13.5	19.2
57.231	1.60839	217	14.7	19.2
57.241	1.60813	192	13.9	19.2
57.251	1.60787	204	14.3	19.2
57.261	1.60761	206	14.4	19.2
57.271	1.60735	209	14.5	19.2
57.281	1.60709	209	14.5	19.2
57.291	1.60683	199	14.1	19.2
57.301	1.60657	169	13.0	19.2
57.311	1.60631	215	14.7	19.2
57.322	1.60605	191	13.8	19.2
57.332	1.60580	181	13.5	19.2
57.342	1.60554	228	15.1	19.2
57.352	1.60528	164	12.8	19.2
57.362	1.60502	198	14.1	19.2
57.372	1.60476	180	13.4	19.2
57.382	1.60450	184	13.6	19.2
57.392	1.60424	207	14.4	19.2
57.402	1.60399	209	14.5	19.2
57.412	1.60373	195	14.0	19.2
57.423	1.60347	216	14.7	19.2
57.433	1.60321	180	13.4	19.2
57.443	1.60295	190	13.8	19.2
57.453	1.60270	221	14.9	19.2
57.463	1.60244	185	13.6	19.2
57.473	1.60218	209	14.5	19.2
57.483	1.60192	213	14.6	19.2
57.493	1.60167	208	14.4	19.2
57.503	1.60141	193	13.9	19.2

57.513	1.60115	202	14.2	19.2
57.524	1.60089	210	14.5	19.2
57.534	1.60064	182	13.5	19.2
57.544	1.60038	187	13.7	19.2
57.554	1.60012	181	13.5	19.2
57.564	1.59987	201	14.2	19.2
57.574	1.59961	194	13.9	19.2
57.584	1.59935	209	14.5	19.2
57.594	1.59910	199	14.1	19.2
57.604	1.59884	207	14.4	19.2
57.614	1.59858	199	14.1	19.2
57.624	1.59833	215	14.7	19.2
57.635	1.59807	183	13.5	19.2
57.645	1.59782	244	15.6	19.2
57.655	1.59756	206	14.4	19.2
57.665	1.59730	209	14.5	19.2
57.675	1.59705	193	13.9	19.2
57.685	1.59679	197	14.0	19.2
57.695	1.59654	190	13.8	19.2
57.705	1.59628	187	13.7	19.2
57.715	1.59603	177	13.3	19.2
57.725	1.59577	189	13.7	19.2
57.736	1.59552	224	15.0	19.2
57.746	1.59526	195	14.0	19.2
57.756	1.59501	168	13.0	19.2
57.766	1.59475	192	13.9	19.2
57.776	1.59450	195	14.0	19.2
57.786	1.59424	179	13.4	19.2
57.796	1.59399	210	14.5	19.2
57.806	1.59373	209	14.5	19.2
57.816	1.59348	202	14.2	19.2
57.826	1.59323	194	13.9	19.2
57.837	1.59297	189	13.7	19.2
57.847	1.59272	222	14.9	19.2
57.857	1.59246	203	14.2	19.2
57.867	1.59221	199	14.1	19.2
57.877	1.59196	191	13.8	19.2
57.887	1.59170	205	14.3	19.2
57.897	1.59145	190	13.8	19.2
57.907	1.59119	196	14.0	19.2
57.917	1.59094	180	13.4	19.2
57.927	1.59069	165	12.8	19.2
57.938	1.59043	170	13.0	19.2
57.948	1.59018	179	13.4	19.2
57.958	1.58993	204	14.3	19.2
57.968	1.58968	218	14.8	19.2
57.978	1.58942	189	13.7	19.2
57.988	1.58917	188	13.7	19.2
57.998	1.58892	191	13.8	19.2
58.008	1.58866	201	14.2	19.2

58.018	1.58841	173	13.2	19.2
58.028	1.58816	189	13.7	19.2
58.039	1.58791	191	13.8	19.2
58.049	1.58765	204	14.3	19.2
58.059	1.58740	201	14.2	19.2
58.069	1.58715	194	13.9	19.2
58.079	1.58690	199	14.1	19.2
58.089	1.58665	183	13.5	19.2
58.099	1.58640	188	13.7	19.2
58.109	1.58614	185	13.6	19.2
58.119	1.58589	173	13.2	19.2
58.129	1.58564	185	13.6	19.2
58.140	1.58539	203	14.2	19.2
58.150	1.58514	180	13.4	19.2
58.160	1.58489	175	13.2	19.2
58.170	1.58464	194	13.9	19.2
58.180	1.58438	175	13.2	19.2
58.190	1.58413	216	14.7	19.2
58.200	1.58388	188	13.7	19.2
58.210	1.58363	200	14.1	19.2
58.220	1.58338	212	14.6	19.2
58.230	1.58313	192	13.9	19.2
58.241	1.58288	175	13.2	19.2
58.251	1.58263	194	13.9	19.2
58.261	1.58238	177	13.3	19.2
58.271	1.58213	187	13.7	19.2
58.281	1.58188	180	13.4	19.2
58.291	1.58163	205	14.3	19.2
58.301	1.58138	189	13.7	19.2
58.311	1.58113	179	13.4	19.2
58.321	1.58088	199	14.1	19.2
58.331	1.58063	201	14.2	19.2
58.342	1.58038	173	13.2	19.2
58.352	1.58013	183	13.5	19.2
58.362	1.57988	184	13.6	19.2
58.372	1.57963	220	14.8	19.2
58.382	1.57938	177	13.3	19.2
58.392	1.57913	212	14.6	19.2
58.402	1.57888	172	13.1	19.2
58.412	1.57864	188	13.7	19.2
58.422	1.57839	166	12.9	19.2
58.432	1.57814	165	12.8	19.2
58.443	1.57789	167	12.9	19.2
58.453	1.57764	209	14.5	19.2
58.463	1.57739	179	13.4	19.2
58.473	1.57714	185	13.6	19.2
58.483	1.57690	198	14.1	19.2
58.493	1.57665	207	14.4	19.2
58.503	1.57640	218	14.8	19.2
58.513	1.57615	170	13.0	19.2

58.523	1.57590	196	14.0	19.2
58.533	1.57566	171	13.1	19.2
58.544	1.57541	190	13.8	19.2
58.554	1.57516	209	14.5	19.2
58.564	1.57491	216	14.7	19.2
58.574	1.57467	176	13.3	19.2
58.584	1.57442	213	14.6	19.2
58.594	1.57417	199	14.1	19.2
58.604	1.57392	187	13.7	19.2
58.614	1.57368	180	13.4	19.2
58.624	1.57343	174	13.2	19.2
58.634	1.57318	179	13.4	19.2
58.645	1.57294	183	13.5	19.2
58.655	1.57269	174	13.2	19.2
58.665	1.57244	196	14.0	19.2
58.675	1.57220	196	14.0	19.2
58.685	1.57195	193	13.9	19.2
58.695	1.57170	182	13.5	19.2
58.705	1.57146	185	13.6	19.2
58.715	1.57121	172	13.1	19.2
58.725	1.57096	189	13.7	19.2
58.735	1.57072	187	13.7	19.2
58.746	1.57047	225	15.0	19.2
58.756	1.57023	192	13.9	19.2
58.766	1.56998	160	12.6	19.2
58.776	1.56973	194	13.9	19.2
58.786	1.56949	194	13.9	19.2
58.796	1.56924	179	13.4	19.2
58.806	1.56900	181	13.5	19.2
58.816	1.56875	184	13.6	19.2
58.826	1.56851	190	13.8	19.2
58.836	1.56826	189	13.7	19.2
58.847	1.56802	182	13.5	19.2
58.857	1.56777	186	13.6	19.2
58.867	1.56753	192	13.9	19.2
58.877	1.56728	187	13.7	19.2
58.887	1.56704	177	13.3	19.2
58.897	1.56679	210	14.5	19.2
58.907	1.56655	183	13.5	19.2
58.917	1.56630	183	13.5	19.2
58.927	1.56606	199	14.1	19.2
58.937	1.56582	196	14.0	19.2
58.947	1.56557	195	14.0	19.2
58.958	1.56533	176	13.3	19.2
58.968	1.56508	185	13.6	19.2
58.978	1.56484	182	13.5	19.2
58.988	1.56460	164	12.8	19.2
58.998	1.56435	180	13.4	19.2
59.008	1.56411	195	14.0	19.2
59.018	1.56386	188	13.7	19.2

59.028	1.56362	189	13.7	19.2
59.038	1.56338	193	13.9	19.2
59.048	1.56313	179	13.4	19.2
59.059	1.56289	199	14.1	19.2
59.069	1.56265	205	14.3	19.2
59.079	1.56241	212	14.6	19.2
59.089	1.56216	203	14.2	19.2
59.099	1.56192	196	14.0	19.2
59.109	1.56168	207	14.4	19.2
59.119	1.56143	209	14.5	19.2
59.129	1.56119	197	14.0	19.2
59.139	1.56095	198	14.1	19.2
59.149	1.56071	185	13.6	19.2
59.160	1.56046	182	13.5	19.2
59.170	1.56022	195	14.0	19.2
59.180	1.55998	205	14.3	19.2
59.190	1.55974	179	13.4	19.2
59.200	1.55950	177	13.3	19.2
59.210	1.55925	174	13.2	19.2
59.220	1.55901	197	14.0	19.2
59.230	1.55877	208	14.4	19.2
59.240	1.55853	221	14.9	19.2
59.250	1.55829	203	14.2	19.2
59.261	1.55805	216	14.7	19.2
59.271	1.55780	172	13.1	19.2
59.281	1.55756	175	13.2	19.2
59.291	1.55732	183	13.5	19.2
59.301	1.55708	192	13.9	19.2
59.311	1.55684	175	13.2	19.2
59.321	1.55660	177	13.3	19.2
59.331	1.55636	195	14.0	19.2
59.341	1.55612	164	12.8	19.2
59.351	1.55588	176	13.3	19.2
59.362	1.55564	198	14.1	19.2
59.372	1.55540	212	14.6	19.2
59.382	1.55515	192	13.9	19.2
59.392	1.55491	190	13.8	19.2
59.402	1.55467	184	13.6	19.2
59.412	1.55443	191	13.8	19.2
59.422	1.55419	177	13.3	19.2
59.432	1.55395	186	13.6	19.2
59.442	1.55371	197	14.0	19.2
59.452	1.55347	200	14.1	19.2
59.463	1.55323	190	13.8	19.2
59.473	1.55299	185	13.6	19.2
59.483	1.55276	190	13.8	19.2
59.493	1.55252	196	14.0	19.2
59.503	1.55228	174	13.2	19.2
59.513	1.55204	204	14.3	19.2
59.523	1.55180	182	13.5	19.2

59.533	1.55156	185	13.6	19.2
59.543	1.55132	183	13.5	19.2
59.553	1.55108	190	13.8	19.2
59.564	1.55084	206	14.4	19.2
59.574	1.55060	182	13.5	19.2
59.584	1.55036	186	13.6	19.2
59.594	1.55013	199	14.1	19.2
59.604	1.54989	184	13.6	19.2
59.614	1.54965	192	13.9	19.2
59.624	1.54941	181	13.5	19.2
59.634	1.54917	174	13.2	19.2
59.644	1.54893	197	14.0	19.2
59.654	1.54870	180	13.4	19.2
59.665	1.54846	188	13.7	19.2
59.675	1.54822	196	14.0	19.2
59.685	1.54798	196	14.0	19.2
59.695	1.54774	220	14.8	19.2
59.705	1.54751	192	13.9	19.2
59.715	1.54727	179	13.4	19.2
59.725	1.54703	185	13.6	19.2
59.735	1.54679	180	13.4	19.2
59.745	1.54656	160	12.6	19.2
59.755	1.54632	186	13.6	19.2
59.766	1.54608	190	13.8	19.2
59.776	1.54585	207	14.4	19.2
59.786	1.54561	182	13.5	19.2
59.796	1.54537	177	13.3	19.2
59.806	1.54513	175	13.2	19.2
59.816	1.54490	171	13.1	19.2
59.826	1.54466	193	13.9	19.2
59.836	1.54442	181	13.5	19.2
59.846	1.54419	188	13.7	19.2
59.856	1.54395	185	13.6	19.2
59.867	1.54372	175	13.2	19.2
59.877	1.54348	164	12.8	19.2
59.887	1.54324	172	13.1	19.2
59.897	1.54301	167	12.9	19.2
59.907	1.54277	167	12.9	19.2
59.917	1.54253	180	13.4	19.2
59.927	1.54230	193	13.9	19.2
59.937	1.54206	186	13.6	19.2
59.947	1.54183	173	13.2	19.2
59.957	1.54159	202	14.2	19.2
59.968	1.54136	189	13.7	19.2
59.978	1.54112	188	13.7	19.2
59.988	1.54089	173	13.2	19.2
59.998	1.54065	194	13.9	19.2
60.008	1.54042	195	14.0	19.2
60.018	1.54018	202	14.2	19.2
60.028	1.53995	181	13.5	19.2

60.038	1.53971	206	14.4	19.2
60.048	1.53948	194	13.9	19.2
60.058	1.53924	211	14.5	19.2
60.069	1.53901	202	14.2	19.2
60.079	1.53877	188	13.7	19.2
60.089	1.53854	200	14.1	19.2
60.099	1.53830	181	13.5	19.2
60.109	1.53807	183	13.5	19.2
60.119	1.53783	192	13.9	19.2
60.129	1.53760	186	13.6	19.2
60.139	1.53737	182	13.5	19.2
60.149	1.53713	173	13.2	19.2
60.159	1.53690	175	13.2	19.2
60.170	1.53666	192	13.9	19.2
60.180	1.53643	162	12.7	19.2
60.190	1.53620	174	13.2	19.2
60.200	1.53596	176	13.3	19.2
60.210	1.53573	187	13.7	19.2
60.220	1.53550	195	14.0	19.2
60.230	1.53526	188	13.7	19.2
60.240	1.53503	189	13.7	19.2
60.250	1.53480	193	13.9	19.2
60.260	1.53456	201	14.2	19.2
60.270	1.53433	181	13.5	19.2
60.281	1.53410	176	13.3	19.2
60.291	1.53387	196	14.0	19.2
60.301	1.53363	175	13.2	19.2
60.311	1.53340	194	13.9	19.2
60.321	1.53317	180	13.4	19.2
60.331	1.53293	191	13.8	19.2
60.341	1.53270	160	12.6	19.2
60.351	1.53247	204	14.3	19.2
60.361	1.53224	187	13.7	19.2
60.371	1.53201	187	13.7	19.2
60.382	1.53177	182	13.5	19.2
60.392	1.53154	167	12.9	19.2
60.402	1.53131	201	14.2	19.2
60.412	1.53108	181	13.5	19.2
60.422	1.53085	168	13.0	19.2
60.432	1.53061	166	12.9	19.2
60.442	1.53038	183	13.5	19.2
60.452	1.53015	186	13.6	19.2
60.462	1.52992	169	13.0	19.2
60.472	1.52969	185	13.6	19.2
60.483	1.52946	187	13.7	19.2
60.493	1.52923	178	13.3	19.2
60.503	1.52900	205	14.3	19.2
60.513	1.52876	180	13.4	19.2
60.523	1.52853	177	13.3	19.2
60.533	1.52830	174	13.2	19.2

60.543	1.52807	179	13.4	19.2
60.553	1.52784	183	13.5	19.2
60.563	1.52761	180	13.4	19.2
60.573	1.52738	171	13.1	19.2
60.584	1.52715	176	13.3	19.2
60.594	1.52692	190	13.8	19.2
60.604	1.52669	179	13.4	19.2
60.614	1.52646	177	13.3	19.2
60.624	1.52623	175	13.2	19.2
60.634	1.52600	185	13.6	19.2
60.644	1.52577	194	13.9	19.2
60.654	1.52554	183	13.5	19.2
60.664	1.52531	179	13.4	19.2
60.674	1.52508	172	13.1	19.2
60.685	1.52485	164	12.8	19.2
60.695	1.52462	194	13.9	19.2
60.705	1.52439	179	13.4	19.2
60.715	1.52416	188	13.7	19.2
60.725	1.52393	200	14.1	19.2
60.735	1.52370	200	14.1	19.2
60.745	1.52347	194	13.9	19.2
60.755	1.52324	177	13.3	19.2
60.765	1.52302	176	13.3	19.2
60.775	1.52279	186	13.6	19.2
60.786	1.52256	177	13.3	19.2
60.796	1.52233	208	14.4	19.2
60.806	1.52210	216	14.7	19.2
60.816	1.52187	167	12.9	19.2
60.826	1.52164	164	12.8	19.2
60.836	1.52141	178	13.3	19.2
60.846	1.52119	185	13.6	19.2
60.856	1.52096	159	12.6	19.2
60.866	1.52073	175	13.2	19.2
60.876	1.52050	177	13.3	19.2
60.887	1.52027	185	13.6	19.2
60.897	1.52005	209	14.5	19.2
60.907	1.51982	167	12.9	19.2
60.917	1.51959	183	13.5	19.2
60.927	1.51936	188	13.7	19.2
60.937	1.51913	204	14.3	19.2
60.947	1.51891	184	13.6	19.2
60.957	1.51868	178	13.3	19.2
60.967	1.51845	163	12.8	19.2
60.977	1.51823	182	13.5	19.2
60.988	1.51800	180	13.4	19.2
60.998	1.51777	185	13.6	19.2
61.008	1.51754	184	13.6	19.2
61.018	1.51732	166	12.9	19.2
61.028	1.51709	182	13.5	19.2
61.038	1.51686	168	13.0	19.2

61.048	1.51664	161	12.7	19.2
61.058	1.51641	186	13.6	19.2
61.068	1.51618	180	13.4	19.2
61.078	1.51596	195	14.0	19.2
61.089	1.51573	176	13.3	19.2
61.099	1.51550	202	14.2	19.2
61.109	1.51528	196	14.0	19.2
61.119	1.51505	219	14.8	19.2
61.129	1.51483	175	13.2	19.2
61.139	1.51460	180	13.4	19.2
61.149	1.51437	188	13.7	19.2
61.159	1.51415	189	13.7	19.2
61.169	1.51392	172	13.1	19.2
61.179	1.51370	180	13.4	19.2
61.190	1.51347	183	13.5	19.2
61.200	1.51324	198	14.1	19.2
61.210	1.51302	187	13.7	19.2
61.220	1.51279	181	13.5	19.2
61.230	1.51257	160	12.6	19.2
61.240	1.51234	199	14.1	19.2
61.250	1.51212	207	14.4	19.2
61.260	1.51189	192	13.9	19.2
61.270	1.51167	172	13.1	19.2
61.280	1.51144	171	13.1	19.2
61.291	1.51122	183	13.5	19.2
61.301	1.51099	188	13.7	19.2
61.311	1.51077	175	13.2	19.2
61.321	1.51054	198	14.1	19.2
61.331	1.51032	171	13.1	19.2
61.341	1.51010	190	13.8	19.2
61.351	1.50987	184	13.6	19.2
61.361	1.50965	172	13.1	19.2
61.371	1.50942	184	13.6	19.2
61.381	1.50920	158	12.6	19.2
61.392	1.50897	186	13.6	19.2
61.402	1.50875	173	13.2	19.2
61.412	1.50853	173	13.2	19.2
61.422	1.50830	163	12.8	19.2
61.432	1.50808	188	13.7	19.2
61.442	1.50786	150	12.2	19.2
61.452	1.50763	163	12.8	19.2
61.462	1.50741	193	13.9	19.2
61.472	1.50718	186	13.6	19.2
61.482	1.50696	186	13.6	19.2
61.493	1.50674	156	12.5	19.2
61.503	1.50651	190	13.8	19.2
61.513	1.50629	191	13.8	19.2
61.523	1.50607	157	12.5	19.2
61.533	1.50585	180	13.4	19.2
61.543	1.50562	186	13.6	19.2

61.553	1.50540	183	13.5	19.2
61.563	1.50518	181	13.5	19.2
61.573	1.50495	174	13.2	19.2
61.583	1.50473	151	12.3	19.2
61.593	1.50451	167	12.9	19.2
61.604	1.50429	187	13.7	19.2
61.614	1.50406	172	13.1	19.2
61.624	1.50384	170	13.0	19.2
61.634	1.50362	189	13.7	19.2
61.644	1.50340	186	13.6	19.2
61.654	1.50318	171	13.1	19.2
61.664	1.50295	150	12.2	19.2
61.674	1.50273	168	13.0	19.2
61.684	1.50251	166	12.9	19.2
61.694	1.50229	187	13.7	19.2
61.705	1.50207	172	13.1	19.2
61.715	1.50185	192	13.9	19.2
61.725	1.50162	178	13.3	19.2
61.735	1.50140	178	13.3	19.2
61.745	1.50118	178	13.3	19.2
61.755	1.50096	177	13.3	19.2
61.765	1.50074	165	12.8	19.2
61.775	1.50052	164	12.8	19.2
61.785	1.50030	182	13.5	19.2
61.795	1.50008	177	13.3	19.2
61.806	1.49985	187	13.7	19.2
61.816	1.49963	187	13.7	19.2
61.826	1.49941	178	13.3	19.2
61.836	1.49919	164	12.8	19.2
61.846	1.49897	181	13.5	19.2
61.856	1.49875	197	14.0	19.2
61.866	1.49853	174	13.2	19.2
61.876	1.49831	192	13.9	19.2
61.886	1.49809	160	12.6	19.2
61.896	1.49787	185	13.6	19.2
61.907	1.49765	154	12.4	19.2
61.917	1.49743	185	13.6	19.2
61.927	1.49721	184	13.6	19.2
61.937	1.49699	177	13.3	19.2
61.947	1.49677	176	13.3	19.2
61.957	1.49655	192	13.9	19.2
61.967	1.49633	181	13.5	19.2
61.977	1.49611	171	13.1	19.2
61.987	1.49589	175	13.2	19.2
61.997	1.49567	178	13.3	19.2
62.008	1.49545	173	13.2	19.2
62.018	1.49523	179	13.4	19.2
62.028	1.49501	179	13.4	19.2
62.038	1.49480	181	13.5	19.2
62.048	1.49458	176	13.3	19.2

62.058	1.49436	144	12.0	19.2
62.068	1.49414	179	13.4	19.2
62.078	1.49392	177	13.3	19.2
62.088	1.49370	199	14.1	19.2
62.098	1.49348	177	13.3	19.2
62.109	1.49326	197	14.0	19.2
62.119	1.49305	183	13.5	19.2
62.129	1.49283	177	13.3	19.2
62.139	1.49261	200	14.1	19.2
62.149	1.49239	188	13.7	19.2
62.159	1.49217	180	13.4	19.2
62.169	1.49195	173	13.2	19.2
62.179	1.49174	159	12.6	19.2
62.189	1.49152	183	13.5	19.2
62.199	1.49130	191	13.8	19.2
62.210	1.49108	165	12.8	19.2
62.220	1.49086	192	13.9	19.2
62.230	1.49065	198	14.1	19.2
62.240	1.49043	160	12.6	19.2
62.250	1.49021	186	13.6	19.2
62.260	1.48999	156	12.5	19.2
62.270	1.48978	181	13.5	19.2
62.280	1.48956	191	13.8	19.2
62.290	1.48934	147	12.1	19.2
62.300	1.48912	174	13.2	19.2
62.311	1.48891	172	13.1	19.2
62.321	1.48869	181	13.5	19.2
62.331	1.48847	165	12.8	19.2
62.341	1.48826	177	13.3	19.2
62.351	1.48804	149	12.2	19.2
62.361	1.48782	191	13.8	19.2
62.371	1.48761	174	13.2	19.2
62.381	1.48739	168	13.0	19.2
62.391	1.48717	178	13.3	19.2
62.401	1.48696	182	13.5	19.2
62.412	1.48674	186	13.6	19.2
62.422	1.48652	202	14.2	19.2
62.432	1.48631	182	13.5	19.2
62.442	1.48609	180	13.4	19.2
62.452	1.48588	186	13.6	19.2
62.462	1.48566	188	13.7	19.2
62.472	1.48544	179	13.4	19.2
62.482	1.48523	187	13.7	19.2
62.492	1.48501	178	13.3	19.2
62.502	1.48480	187	13.7	19.2
62.513	1.48458	184	13.6	19.2
62.523	1.48437	157	12.5	19.2
62.533	1.48415	170	13.0	19.2
62.543	1.48394	191	13.8	19.2
62.553	1.48372	164	12.8	19.2

62.563	1.48350	167	12.9	19.2
62.573	1.48329	165	12.8	19.2
62.583	1.48307	164	12.8	19.2
62.593	1.48286	182	13.5	19.2
62.603	1.48264	180	13.4	19.2
62.614	1.48243	157	12.5	19.2
62.624	1.48221	193	13.9	19.2
62.634	1.48200	172	13.1	19.2
62.644	1.48179	152	12.3	19.2
62.654	1.48157	174	13.2	19.2
62.664	1.48136	153	12.4	19.2
62.674	1.48114	170	13.0	19.2
62.684	1.48093	183	13.5	19.2
62.694	1.48071	185	13.6	19.2
62.704	1.48050	174	13.2	19.2
62.715	1.48029	169	13.0	19.2
62.725	1.48007	160	12.6	19.2
62.735	1.47986	168	13.0	19.2
62.745	1.47964	163	12.8	19.2
62.755	1.47943	190	13.8	19.2
62.765	1.47922	190	13.8	19.2
62.775	1.47900	193	13.9	19.2
62.785	1.47879	166	12.9	19.2
62.795	1.47857	180	13.4	19.2
62.805	1.47836	183	13.5	19.2
62.816	1.47815	177	13.3	19.2
62.826	1.47793	179	13.4	19.2
62.836	1.47772	182	13.5	19.2
62.846	1.47751	187	13.7	19.2
62.856	1.47730	209	14.5	19.2
62.866	1.47708	175	13.2	19.2
62.876	1.47687	194	13.9	19.2
62.886	1.47666	162	12.7	19.2
62.896	1.47644	171	13.1	19.2
62.906	1.47623	163	12.8	19.2
62.916	1.47602	165	12.8	19.2
62.927	1.47581	179	13.4	19.2
62.937	1.47559	187	13.7	19.2
62.947	1.47538	177	13.3	19.2
62.957	1.47517	153	12.4	19.2
62.967	1.47496	158	12.6	19.2
62.977	1.47474	176	13.3	19.2
62.987	1.47453	174	13.2	19.2
62.997	1.47432	173	13.2	19.2
63.007	1.47411	156	12.5	19.2
63.017	1.47390	180	13.4	19.2
63.028	1.47368	166	12.9	19.2
63.038	1.47347	161	12.7	19.2
63.048	1.47326	156	12.5	19.2
63.058	1.47305	164	12.8	19.2

63.068	1.47284	159	12.6	19.2
63.078	1.47263	175	13.2	19.2
63.088	1.47241	170	13.0	19.2
63.098	1.47220	179	13.4	19.2
63.108	1.47199	167	12.9	19.2
63.118	1.47178	173	13.2	19.2
63.129	1.47157	169	13.0	19.2
63.139	1.47136	188	13.7	19.2
63.149	1.47115	182	13.5	19.2
63.159	1.47094	167	12.9	19.2
63.169	1.47072	192	13.9	19.2
63.179	1.47051	177	13.3	19.2
63.189	1.47030	169	13.0	19.2
63.199	1.47009	162	12.7	19.2
63.209	1.46988	168	13.0	19.2
63.219	1.46967	158	12.6	19.2
63.230	1.46946	144	12.0	19.2
63.240	1.46925	166	12.9	19.2
63.250	1.46904	157	12.5	19.2
63.260	1.46883	165	12.8	19.2
63.270	1.46862	169	13.0	19.2
63.280	1.46841	181	13.5	19.2
63.290	1.46820	164	12.8	19.2
63.300	1.46799	151	12.3	19.2
63.310	1.46778	164	12.8	19.2
63.320	1.46757	172	13.1	19.2
63.331	1.46736	175	13.2	19.2
63.341	1.46715	187	13.7	19.2
63.351	1.46694	154	12.4	19.2
63.361	1.46673	190	13.8	19.2
63.371	1.46652	161	12.7	19.2
63.381	1.46631	175	13.2	19.2
63.391	1.46610	161	12.7	19.2
63.401	1.46590	181	13.5	19.2
63.411	1.46569	182	13.5	19.2
63.421	1.46548	170	13.0	19.2
63.432	1.46527	175	13.2	19.2
63.442	1.46506	171	13.1	19.2
63.452	1.46485	177	13.3	19.2
63.462	1.46464	160	12.6	19.2
63.472	1.46443	159	12.6	19.2
63.482	1.46422	168	13.0	19.2
63.492	1.46402	169	13.0	19.2
63.502	1.46381	170	13.0	19.2
63.512	1.46360	177	13.3	19.2
63.522	1.46339	171	13.1	19.2
63.533	1.46318	156	12.5	19.2
63.543	1.46297	180	13.4	19.2
63.553	1.46277	173	13.2	19.2
63.563	1.46256	168	13.0	19.2

63.573	1.46235	161	12.7	19.2
63.583	1.46214	162	12.7	19.2
63.593	1.46193	202	14.2	19.2
63.603	1.46173	191	13.8	19.2
63.613	1.46152	162	12.7	19.2
63.623	1.46131	171	13.1	19.2
63.634	1.46110	173	13.2	19.2
63.644	1.46090	151	12.3	19.2
63.654	1.46069	164	12.8	19.2
63.664	1.46048	146	12.1	19.2
63.674	1.46027	189	13.7	19.2
63.684	1.46007	177	13.3	19.2
63.694	1.45986	174	13.2	19.2
63.704	1.45965	191	13.8	19.2
63.714	1.45944	149	12.2	19.2
63.724	1.45924	164	12.8	19.2
63.735	1.45903	176	13.3	19.2
63.745	1.45882	165	12.8	19.2
63.755	1.45862	176	13.3	19.2
63.765	1.45841	185	13.6	19.2
63.775	1.45820	140	11.8	19.2
63.785	1.45800	166	12.9	19.2
63.795	1.45779	169	13.0	19.2
63.805	1.45758	179	13.4	19.2
63.815	1.45738	176	13.3	19.2
63.825	1.45717	169	13.0	19.2
63.836	1.45697	177	13.3	19.2
63.846	1.45676	156	12.5	19.2
63.856	1.45655	174	13.2	19.2
63.866	1.45635	192	13.9	19.2
63.876	1.45614	170	13.0	19.2
63.886	1.45594	177	13.3	19.2
63.896	1.45573	169	13.0	19.2
63.906	1.45552	152	12.3	19.2
63.916	1.45532	137	11.7	19.2
63.926	1.45511	169	13.0	19.2
63.937	1.45491	172	13.1	19.2
63.947	1.45470	166	12.9	19.2
63.957	1.45450	202	14.2	19.2
63.967	1.45429	157	12.5	19.2
63.977	1.45409	153	12.4	19.2
63.987	1.45388	157	12.5	19.2
63.997	1.45368	160	12.6	19.2
64.007	1.45347	181	13.5	19.2
64.017	1.45327	176	13.3	19.2
64.027	1.45306	164	12.8	19.2
64.038	1.45286	178	13.3	19.2
64.048	1.45265	164	12.8	19.2
64.058	1.45245	159	12.6	19.2
64.068	1.45224	174	13.2	19.2

64.078	1.45204	186	13.6	19.2
64.088	1.45183	161	12.7	19.2
64.098	1.45163	168	13.0	19.2
64.108	1.45142	180	13.4	19.2
64.118	1.45122	162	12.7	19.2
64.128	1.45102	159	12.6	19.2
64.139	1.45081	164	12.8	19.2
64.149	1.45061	190	13.8	19.2
64.159	1.45040	158	12.6	19.2
64.169	1.45020	168	13.0	19.2
64.179	1.45000	172	13.1	19.2
64.189	1.44979	166	12.9	19.2
64.199	1.44959	185	13.6	19.2
64.209	1.44939	153	12.4	19.2
64.219	1.44918	170	13.0	19.2
64.229	1.44898	170	13.0	19.2
64.239	1.44878	158	12.6	19.2
64.250	1.44857	173	13.2	19.2
64.260	1.44837	173	13.2	19.2
64.270	1.44817	182	13.5	19.2
64.280	1.44796	175	13.2	19.2
64.290	1.44776	156	12.5	19.2
64.300	1.44756	170	13.0	19.2
64.310	1.44735	178	13.3	19.2
64.320	1.44715	175	13.2	19.2
64.330	1.44695	178	13.3	19.2
64.340	1.44674	152	12.3	19.2
64.351	1.44654	179	13.4	19.2
64.361	1.44634	184	13.6	19.2
64.371	1.44614	141	11.9	19.2
64.381	1.44593	174	13.2	19.2
64.391	1.44573	165	12.8	19.2
64.401	1.44553	186	13.6	19.2
64.411	1.44533	170	13.0	19.2
64.421	1.44513	162	12.7	19.2
64.431	1.44492	158	12.6	19.2
64.441	1.44472	146	12.1	19.2
64.452	1.44452	165	12.8	19.2
64.462	1.44432	147	12.1	19.2
64.472	1.44412	168	13.0	19.2
64.482	1.44391	179	13.4	19.2
64.492	1.44371	169	13.0	19.2
64.502	1.44351	157	12.5	19.2
64.512	1.44331	171	13.1	19.2
64.522	1.44311	143	12.0	19.2
64.532	1.44291	161	12.7	19.2
64.542	1.44270	147	12.1	19.2
64.553	1.44250	194	13.9	19.2
64.563	1.44230	155	12.4	19.2
64.573	1.44210	195	14.0	19.2

64.583	1.44190	163	12.8	19.2
64.593	1.44170	163	12.8	19.2
64.603	1.44150	185	13.6	19.2
64.613	1.44130	162	12.7	19.2
64.623	1.44110	168	13.0	19.2
64.633	1.44089	152	12.3	19.2
64.643	1.44069	183	13.5	19.2
64.654	1.44049	150	12.2	19.2
64.664	1.44029	149	12.2	19.2
64.674	1.44009	166	12.9	19.2
64.684	1.43989	172	13.1	19.2
64.694	1.43969	157	12.5	19.2
64.704	1.43949	167	12.9	19.2
64.714	1.43929	145	12.0	19.2
64.724	1.43909	155	12.4	19.2
64.734	1.43889	152	12.3	19.2
64.744	1.43869	168	13.0	19.2
64.755	1.43849	164	12.8	19.2
64.765	1.43829	159	12.6	19.2
64.775	1.43809	207	14.4	19.2
64.785	1.43789	151	12.3	19.2
64.795	1.43769	157	12.5	19.2
64.805	1.43749	178	13.3	19.2
64.815	1.43729	147	12.1	19.2
64.825	1.43709	175	13.2	19.2
64.835	1.43689	186	13.6	19.2
64.845	1.43669	193	13.9	19.2
64.856	1.43649	173	13.2	19.2
64.866	1.43630	166	12.9	19.2
64.876	1.43610	156	12.5	19.2
64.886	1.43590	166	12.9	19.2
64.896	1.43570	154	12.4	19.2
64.906	1.43550	166	12.9	19.2
64.916	1.43530	157	12.5	19.2
64.926	1.43510	157	12.5	19.2
64.936	1.43490	192	13.9	19.2
64.946	1.43470	148	12.2	19.2
64.957	1.43450	175	13.2	19.2
64.967	1.43431	186	13.6	19.2
64.977	1.43411	160	12.6	19.2
64.987	1.43391	135	11.6	19.2
64.997	1.43371	164	12.8	19.2
65.007	1.43351	174	13.2	19.2
65.017	1.43331	187	13.7	19.2
65.027	1.43312	161	12.7	19.2
65.037	1.43292	173	13.2	19.2
65.047	1.43272	176	13.3	19.2
65.058	1.43252	144	12.0	19.2
65.068	1.43232	166	12.9	19.2
65.078	1.43213	188	13.7	19.2

65.088	1.43193	181	13.5	19.2
65.098	1.43173	189	13.7	19.2
65.108	1.43153	154	12.4	19.2
65.118	1.43134	154	12.4	19.2
65.128	1.43114	172	13.1	19.2
65.138	1.43094	192	13.9	19.2
65.148	1.43074	164	12.8	19.2
65.159	1.43055	150	12.2	19.2
65.169	1.43035	161	12.7	19.2
65.179	1.43015	148	12.2	19.2
65.189	1.42995	164	12.8	19.2
65.199	1.42976	162	12.7	19.2
65.209	1.42956	148	12.2	19.2
65.219	1.42936	156	12.5	19.2
65.229	1.42917	164	12.8	19.2
65.239	1.42897	167	12.9	19.2
65.249	1.42877	182	13.5	19.2
65.260	1.42858	155	12.4	19.2
65.270	1.42838	172	13.1	19.2
65.280	1.42818	162	12.7	19.2
65.290	1.42799	151	12.3	19.2
65.300	1.42779	170	13.0	19.2
65.310	1.42759	175	13.2	19.2
65.320	1.42740	158	12.6	19.2
65.330	1.42720	148	12.2	19.2
65.340	1.42700	172	13.1	19.2
65.350	1.42681	175	13.2	19.2
65.361	1.42661	164	12.8	19.2
65.371	1.42642	181	13.5	19.2
65.381	1.42622	152	12.3	19.2
65.391	1.42603	154	12.4	19.2
65.401	1.42583	171	13.1	19.2
65.411	1.42563	168	13.0	19.2
65.421	1.42544	169	13.0	19.2
65.431	1.42524	177	13.3	19.2
65.441	1.42505	168	13.0	19.2
65.451	1.42485	138	11.7	19.2
65.462	1.42466	156	12.5	19.2
65.472	1.42446	184	13.6	19.2
65.482	1.42427	148	12.2	19.2
65.492	1.42407	152	12.3	19.2
65.502	1.42388	153	12.4	19.2
65.512	1.42368	162	12.7	19.2
65.522	1.42349	161	12.7	19.2
65.532	1.42329	173	13.2	19.2
65.542	1.42310	144	12.0	19.2
65.552	1.42290	204	14.3	19.2
65.562	1.42271	171	13.1	19.2
65.573	1.42251	143	12.0	19.2
65.583	1.42232	158	12.6	19.2

65.593	1.42212	173	13.2	19.2
65.603	1.42193	170	13.0	19.2
65.613	1.42173	140	11.8	19.2
65.623	1.42154	162	12.7	19.2
65.633	1.42134	170	13.0	19.2
65.643	1.42115	166	12.9	19.2
65.653	1.42096	177	13.3	19.2
65.663	1.42076	158	12.6	19.2
65.674	1.42057	186	13.6	19.2
65.684	1.42037	181	13.5	19.2
65.694	1.42018	166	12.9	19.2
65.704	1.41999	171	13.1	19.2
65.714	1.41979	129	11.4	19.2
65.724	1.41960	155	12.4	19.2
65.734	1.41941	168	13.0	19.2
65.744	1.41921	162	12.7	19.2
65.754	1.41902	163	12.8	19.2
65.764	1.41882	161	12.7	19.2
65.775	1.41863	151	12.3	19.2
65.785	1.41844	173	13.2	19.2
65.795	1.41824	152	12.3	19.2
65.805	1.41805	159	12.6	19.2
65.815	1.41786	152	12.3	19.2
65.825	1.41767	177	13.3	19.2
65.835	1.41747	162	12.7	19.2
65.845	1.41728	170	13.0	19.2
65.855	1.41709	157	12.5	19.2
65.865	1.41689	197	14.0	19.2
65.876	1.41670	136	11.7	19.2
65.886	1.41651	154	12.4	19.2
65.896	1.41632	181	13.5	19.2
65.906	1.41612	141	11.9	19.2
65.916	1.41593	167	12.9	19.2
65.926	1.41574	154	12.4	19.2
65.936	1.41555	154	12.4	19.2
65.946	1.41535	161	12.7	19.2
65.956	1.41516	143	12.0	19.2
65.966	1.41497	153	12.4	19.2
65.977	1.41478	143	12.0	19.2
65.987	1.41458	156	12.5	19.2
65.997	1.41439	159	12.6	19.2
66.007	1.41420	170	13.0	19.2
66.017	1.41401	155	12.4	19.2
66.027	1.41382	178	13.3	19.2
66.037	1.41363	164	12.8	19.2
66.047	1.41343	158	12.6	19.2
66.057	1.41324	165	12.8	19.2
66.067	1.41305	151	12.3	19.2
66.078	1.41286	182	13.5	19.2
66.088	1.41267	154	12.4	19.2

66.098	1.41248	147	12.1	19.2
66.108	1.41228	148	12.2	19.2
66.118	1.41209	158	12.6	19.2
66.128	1.41190	158	12.6	19.2
66.138	1.41171	168	13.0	19.2
66.148	1.41152	173	13.2	19.2
66.158	1.41133	166	12.9	19.2
66.168	1.41114	156	12.5	19.2
66.179	1.41095	143	12.0	19.2
66.189	1.41076	158	12.6	19.2
66.199	1.41057	144	12.0	19.2
66.209	1.41038	169	13.0	19.2
66.219	1.41018	184	13.6	19.2
66.229	1.40999	159	12.6	19.2
66.239	1.40980	167	12.9	19.2
66.249	1.40961	173	13.2	19.2
66.259	1.40942	155	12.4	19.2
66.269	1.40923	154	12.4	19.2
66.280	1.40904	161	12.7	19.2
66.290	1.40885	187	13.7	19.2
66.300	1.40866	154	12.4	19.2
66.310	1.40847	170	13.0	19.2
66.320	1.40828	142	11.9	19.2
66.330	1.40809	150	12.2	19.2
66.340	1.40790	149	12.2	19.2
66.350	1.40771	171	13.1	19.2
66.360	1.40752	177	13.3	19.2
66.370	1.40733	175	13.2	19.2
66.381	1.40714	169	13.0	19.2
66.391	1.40695	157	12.5	19.2
66.401	1.40676	178	13.3	19.2
66.411	1.40658	147	12.1	19.2
66.421	1.40639	174	13.2	19.2
66.431	1.40620	156	12.5	19.2
66.441	1.40601	151	12.3	19.2
66.451	1.40582	174	13.2	19.2
66.461	1.40563	153	12.4	19.2
66.471	1.40544	153	12.4	19.2
66.482	1.40525	196	14.0	19.2
66.492	1.40506	176	13.3	19.2
66.502	1.40487	157	12.5	19.2
66.512	1.40468	165	12.8	19.2
66.522	1.40450	173	13.2	19.2
66.532	1.40431	162	12.7	19.2
66.542	1.40412	148	12.2	19.2
66.552	1.40393	150	12.2	19.2
66.562	1.40374	135	11.6	19.2
66.572	1.40355	162	12.7	19.2
66.583	1.40336	148	12.2	19.2
66.593	1.40318	153	12.4	19.2

66.603	1.40299	146	12.1	19.2
66.613	1.40280	158	12.6	19.2
66.623	1.40261	181	13.5	19.2
66.633	1.40242	137	11.7	19.2
66.643	1.40224	146	12.1	19.2
66.653	1.40205	155	12.4	19.2
66.663	1.40186	171	13.1	19.2
66.673	1.40167	159	12.6	19.2
66.684	1.40148	154	12.4	19.2
66.694	1.40130	151	12.3	19.2
66.704	1.40111	143	12.0	19.2
66.714	1.40092	166	12.9	19.2
66.724	1.40073	143	12.0	19.2
66.734	1.40055	173	13.2	19.2
66.744	1.40036	171	13.1	19.2
66.754	1.40017	145	12.0	19.2
66.764	1.39998	150	12.2	19.2
66.774	1.39980	143	12.0	19.2
66.784	1.39961	152	12.3	19.2
66.795	1.39942	169	13.0	19.2
66.805	1.39924	173	13.2	19.2
66.815	1.39905	172	13.1	19.2
66.825	1.39886	165	12.8	19.2
66.835	1.39867	168	13.0	19.2
66.845	1.39849	147	12.1	19.2
66.855	1.39830	140	11.8	19.2
66.865	1.39811	165	12.8	19.2
66.875	1.39793	151	12.3	19.2
66.885	1.39774	154	12.4	19.2
66.896	1.39755	153	12.4	19.2
66.906	1.39737	157	12.5	19.2
66.916	1.39718	143	12.0	19.2
66.926	1.39700	149	12.2	19.2
66.936	1.39681	178	13.3	19.2
66.946	1.39662	171	13.1	19.2
66.956	1.39644	124	11.1	19.2
66.966	1.39625	163	12.8	19.2
66.976	1.39606	169	13.0	19.2
66.986	1.39588	160	12.6	19.2
66.997	1.39569	163	12.8	19.2
67.007	1.39551	170	13.0	19.2
67.017	1.39532	158	12.6	19.2
67.027	1.39514	168	13.0	19.2
67.037	1.39495	152	12.3	19.2
67.047	1.39476	157	12.5	19.2
67.057	1.39458	161	12.7	19.2
67.067	1.39439	174	13.2	19.2
67.077	1.39421	180	13.4	19.2
67.087	1.39402	139	11.8	19.2
67.098	1.39384	158	12.6	19.2

67.108	1.39365	167	12.9	19.2
67.118	1.39347	155	12.4	19.2
67.128	1.39328	162	12.7	19.2
67.138	1.39310	168	13.0	19.2
67.148	1.39291	150	12.2	19.2
67.158	1.39273	162	12.7	19.2
67.168	1.39254	148	12.2	19.2
67.178	1.39236	155	12.4	19.2
67.188	1.39217	134	11.6	19.2
67.199	1.39199	187	13.7	19.2
67.209	1.39180	167	12.9	19.2
67.219	1.39162	171	13.1	19.2
67.229	1.39143	180	13.4	19.2
67.239	1.39125	181	13.5	19.2
67.249	1.39107	179	13.4	19.2
67.259	1.39088	142	11.9	19.2
67.269	1.39070	144	12.0	19.2
67.279	1.39051	178	13.3	19.2
67.289	1.39033	179	13.4	19.2
67.300	1.39014	169	13.0	19.2
67.310	1.38996	128	11.3	19.2
67.320	1.38978	145	12.0	19.2
67.330	1.38959	172	13.1	19.2
67.340	1.38941	155	12.4	19.2
67.350	1.38923	164	12.8	19.2
67.360	1.38904	152	12.3	19.2
67.370	1.38886	157	12.5	19.2
67.380	1.38867	161	12.7	19.2
67.390	1.38849	146	12.1	19.2
67.401	1.38831	157	12.5	19.2
67.411	1.38812	155	12.4	19.2
67.421	1.38794	151	12.3	19.2
67.431	1.38776	161	12.7	19.2
67.441	1.38757	144	12.0	19.2
67.451	1.38739	163	12.8	19.2
67.461	1.38721	135	11.6	19.2
67.471	1.38702	168	13.0	19.2
67.481	1.38684	160	12.6	19.2
67.491	1.38666	143	12.0	19.2
67.502	1.38648	150	12.2	19.2
67.512	1.38629	153	12.4	19.2
67.522	1.38611	145	12.0	19.2
67.532	1.38593	156	12.5	19.2
67.542	1.38574	152	12.3	19.2
67.552	1.38556	175	13.2	19.2
67.562	1.38538	159	12.6	19.2
67.572	1.38520	152	12.3	19.2
67.582	1.38501	194	13.9	19.2
67.592	1.38483	158	12.6	19.2
67.603	1.38465	164	12.8	19.2

67.613	1.38447	160	12.6	19.2
67.623	1.38429	145	12.0	19.2
67.633	1.38410	161	12.7	19.2
67.643	1.38392	145	12.0	19.2
67.653	1.38374	164	12.8	19.2
67.663	1.38356	145	12.0	19.2
67.673	1.38338	145	12.0	19.2
67.683	1.38319	154	12.4	19.2
67.693	1.38301	160	12.6	19.2
67.704	1.38283	174	13.2	19.2
67.714	1.38265	153	12.4	19.2
67.724	1.38247	148	12.2	19.2
67.734	1.38228	154	12.4	19.2
67.744	1.38210	181	13.5	19.2
67.754	1.38192	154	12.4	19.2
67.764	1.38174	172	13.1	19.2
67.774	1.38156	165	12.8	19.2
67.784	1.38138	176	13.3	19.2
67.794	1.38120	151	12.3	19.2
67.805	1.38102	151	12.3	19.2
67.815	1.38083	162	12.7	19.2
67.825	1.38065	158	12.6	19.2
67.835	1.38047	142	11.9	19.2
67.845	1.38029	140	11.8	19.2
67.855	1.38011	182	13.5	19.2
67.865	1.37993	148	12.2	19.2
67.875	1.37975	143	12.0	19.2
67.885	1.37957	152	12.3	19.2
67.895	1.37939	151	12.3	19.2
67.906	1.37921	170	13.0	19.2
67.916	1.37903	143	12.0	19.2
67.926	1.37885	156	12.5	19.2
67.936	1.37867	148	12.2	19.2
67.946	1.37849	150	12.2	19.2
67.956	1.37831	156	12.5	19.2
67.966	1.37813	153	12.4	19.2
67.976	1.37795	148	12.2	19.2
67.986	1.37777	146	12.1	19.2
67.996	1.37759	170	13.0	19.2
68.007	1.37741	160	12.6	19.2
68.017	1.37723	148	12.2	19.2
68.027	1.37705	159	12.6	19.2
68.037	1.37687	168	13.0	19.2
68.047	1.37669	156	12.5	19.2
68.057	1.37651	159	12.6	19.2
68.067	1.37633	167	12.9	19.2
68.077	1.37615	160	12.6	19.2
68.087	1.37597	149	12.2	19.2
68.097	1.37579	161	12.7	19.2
68.107	1.37561	160	12.6	19.2

68.118	1.37543	148	12.2	19.2
68.128	1.37525	135	11.6	19.2
68.138	1.37507	133	11.5	19.2
68.148	1.37489	161	12.7	19.2
68.158	1.37471	180	13.4	19.2
68.168	1.37453	145	12.0	19.2
68.178	1.37435	139	11.8	19.2
68.188	1.37418	169	13.0	19.2
68.198	1.37400	156	12.5	19.2
68.208	1.37382	175	13.2	19.2
68.219	1.37364	139	11.8	19.2
68.229	1.37346	169	13.0	19.2
68.239	1.37328	159	12.6	19.2
68.249	1.37310	122	11.0	19.2
68.259	1.37292	147	12.1	19.2
68.269	1.37275	146	12.1	19.2
68.279	1.37257	169	13.0	19.2
68.289	1.37239	168	13.0	19.2
68.299	1.37221	148	12.2	19.2
68.309	1.37203	150	12.2	19.2
68.320	1.37185	155	12.4	19.2
68.330	1.37168	151	12.3	19.2
68.340	1.37150	144	12.0	19.2
68.350	1.37132	146	12.1	19.2
68.360	1.37114	156	12.5	19.2
68.370	1.37096	150	12.2	19.2
68.380	1.37079	170	13.0	19.2
68.390	1.37061	149	12.2	19.2
68.400	1.37043	164	12.8	19.2
68.410	1.37025	146	12.1	19.2
68.421	1.37008	156	12.5	19.2
68.431	1.36990	154	12.4	19.2
68.441	1.36972	124	11.1	19.2
68.451	1.36954	161	12.7	19.2
68.461	1.36937	150	12.2	19.2
68.471	1.36919	149	12.2	19.2
68.481	1.36901	155	12.4	19.2
68.491	1.36883	142	11.9	19.2
68.501	1.36866	154	12.4	19.2
68.511	1.36848	151	12.3	19.2
68.522	1.36830	179	13.4	19.2
68.532	1.36812	147	12.1	19.2
68.542	1.36795	138	11.7	19.2
68.552	1.36777	144	12.0	19.2
68.562	1.36759	161	12.7	19.2
68.572	1.36742	167	12.9	19.2
68.582	1.36724	167	12.9	19.2
68.592	1.36706	163	12.8	19.2
68.602	1.36689	134	11.6	19.2
68.612	1.36671	150	12.2	19.2

68.623	1.36653	132	11.5	19.2
68.633	1.36636	167	12.9	19.2
68.643	1.36618	176	13.3	19.2
68.653	1.36601	146	12.1	19.2
68.663	1.36583	170	13.0	19.2
68.673	1.36565	144	12.0	19.2
68.683	1.36548	147	12.1	19.2
68.693	1.36530	141	11.9	19.2
68.703	1.36512	166	12.9	19.2
68.713	1.36495	184	13.6	19.2
68.724	1.36477	172	13.1	19.2
68.734	1.36460	139	11.8	19.2
68.744	1.36442	148	12.2	19.2
68.754	1.36424	152	12.3	19.2
68.764	1.36407	159	12.6	19.2
68.774	1.36389	166	12.9	19.2
68.784	1.36372	174	13.2	19.2
68.794	1.36354	177	13.3	19.2
68.804	1.36337	180	13.4	19.2
68.814	1.36319	153	12.4	19.2
68.825	1.36302	165	12.8	19.2
68.835	1.36284	143	12.0	19.2
68.845	1.36267	125	11.2	19.2
68.855	1.36249	160	12.6	19.2
68.865	1.36232	157	12.5	19.2
68.875	1.36214	144	12.0	19.2
68.885	1.36196	154	12.4	19.2
68.895	1.36179	145	12.0	19.2
68.905	1.36161	152	12.3	19.2
68.915	1.36144	145	12.0	19.2
68.926	1.36127	139	11.8	19.2
68.936	1.36109	151	12.3	19.2
68.946	1.36092	153	12.4	19.2
68.956	1.36074	153	12.4	19.2
68.966	1.36057	154	12.4	19.2
68.976	1.36039	176	13.3	19.2
68.986	1.36022	155	12.4	19.2
68.996	1.36004	132	11.5	19.2
69.006	1.35987	149	12.2	19.2
69.016	1.35969	159	12.6	19.2
69.027	1.35952	135	11.6	19.2
69.037	1.35935	141	11.9	19.2
69.047	1.35917	153	12.4	19.2
69.057	1.35900	155	12.4	19.2
69.067	1.35882	155	12.4	19.2
69.077	1.35865	167	12.9	19.2
69.087	1.35848	140	11.8	19.2
69.097	1.35830	165	12.8	19.2
69.107	1.35813	169	13.0	19.2
69.117	1.35795	141	11.9	19.2

69.128	1.35778	151	12.3	19.2
69.138	1.35761	155	12.4	19.2
69.148	1.35743	173	13.2	19.2
69.158	1.35726	148	12.2	19.2
69.168	1.35709	160	12.6	19.2
69.178	1.35691	150	12.2	19.2
69.188	1.35674	142	11.9	19.2
69.198	1.35657	160	12.6	19.2
69.208	1.35639	166	12.9	19.2
69.218	1.35622	149	12.2	19.2
69.229	1.35605	158	12.6	19.2
69.239	1.35587	146	12.1	19.2
69.249	1.35570	131	11.4	19.2
69.259	1.35553	156	12.5	19.2
69.269	1.35535	147	12.1	19.2
69.279	1.35518	129	11.4	19.2
69.289	1.35501	149	12.2	19.2
69.299	1.35484	152	12.3	19.2
69.309	1.35466	165	12.8	19.2
69.319	1.35449	130	11.4	19.2
69.330	1.35432	157	12.5	19.2
69.340	1.35414	146	12.1	19.2
69.350	1.35397	154	12.4	19.2
69.360	1.35380	147	12.1	19.2
69.370	1.35363	168	13.0	19.2
69.380	1.35345	163	12.8	19.2
69.390	1.35328	168	13.0	19.2
69.400	1.35311	147	12.1	19.2
69.410	1.35294	151	12.3	19.2
69.420	1.35277	168	13.0	19.2
69.430	1.35259	158	12.6	19.2
69.441	1.35242	170	13.0	19.2
69.451	1.35225	168	13.0	19.2
69.461	1.35208	157	12.5	19.2
69.471	1.35191	139	11.8	19.2
69.481	1.35173	153	12.4	19.2
69.491	1.35156	166	12.9	19.2
69.501	1.35139	173	13.2	19.2
69.511	1.35122	149	12.2	19.2
69.521	1.35105	139	11.8	19.2
69.531	1.35088	145	12.0	19.2
69.542	1.35070	149	12.2	19.2
69.552	1.35053	148	12.2	19.2
69.562	1.35036	126	11.2	19.2
69.572	1.35019	150	12.2	19.2
69.582	1.35002	170	13.0	19.2
69.592	1.34985	155	12.4	19.2
69.602	1.34968	145	12.0	19.2
69.612	1.34951	150	12.2	19.2
69.622	1.34933	163	12.8	19.2

69.632	1.34916	160	12.6	19.2
69.643	1.34899	156	12.5	19.2
69.653	1.34882	156	12.5	19.2
69.663	1.34865	122	11.0	19.2
69.673	1.34848	159	12.6	19.2
69.683	1.34831	142	11.9	19.2
69.693	1.34814	149	12.2	19.2
69.703	1.34797	175	13.2	19.2
69.713	1.34780	145	12.0	19.2
69.723	1.34763	148	12.2	19.2
69.733	1.34746	147	12.1	19.2
69.744	1.34729	175	13.2	19.2
69.754	1.34712	126	11.2	19.2
69.764	1.34695	170	13.0	19.2
69.774	1.34678	155	12.4	19.2
69.784	1.34660	140	11.8	19.2
69.794	1.34643	136	11.7	19.2
69.804	1.34626	134	11.6	19.2
69.814	1.34609	137	11.7	19.2
69.824	1.34592	152	12.3	19.2
69.834	1.34575	163	12.8	19.2
69.845	1.34558	154	12.4	19.2
69.855	1.34542	153	12.4	19.2
69.865	1.34525	145	12.0	19.2
69.875	1.34508	150	12.2	19.2
69.885	1.34491	170	13.0	19.2
69.895	1.34474	149	12.2	19.2
69.905	1.34457	156	12.5	19.2
69.915	1.34440	163	12.8	19.2
69.925	1.34423	154	12.4	19.2
69.935	1.34406	136	11.7	19.2
69.946	1.34389	156	12.5	19.2
69.956	1.34372	160	12.6	19.2
69.966	1.34355	137	11.7	19.2
69.976	1.34338	137	11.7	19.2
69.986	1.34321	151	12.3	19.2
69.996	1.34304	148	12.2	19.2
70.006	1.34287	171	13.1	19.2
70.016	1.34271	146	12.1	19.2
70.026	1.34254	142	11.9	19.2
70.036	1.34237	157	12.5	19.2
70.047	1.34220	165	12.8	19.2
70.057	1.34203	158	12.6	19.2
70.067	1.34186	171	13.1	19.2
70.077	1.34169	157	12.5	19.2
70.087	1.34152	157	12.5	19.2
70.097	1.34136	148	12.2	19.2
70.107	1.34119	139	11.8	19.2
70.117	1.34102	163	12.8	19.2
70.127	1.34085	151	12.3	19.2

70.137	1.34068	150	12.2	19.2
70.148	1.34051	173	13.2	19.2
70.158	1.34034	149	12.2	19.2
70.168	1.34018	148	12.2	19.2
70.178	1.34001	142	11.9	19.2
70.188	1.33984	184	13.6	19.2
70.198	1.33967	153	12.4	19.2
70.208	1.33950	166	12.9	19.2
70.218	1.33934	170	13.0	19.2
70.228	1.33917	159	12.6	19.2
70.238	1.33900	149	12.2	19.2
70.249	1.33883	124	11.1	19.2
70.259	1.33867	153	12.4	19.2
70.269	1.33850	144	12.0	19.2
70.279	1.33833	162	12.7	19.2
70.289	1.33816	150	12.2	19.2
70.299	1.33800	150	12.2	19.2
70.309	1.33783	165	12.8	19.2
70.319	1.33766	147	12.1	19.2
70.329	1.33749	158	12.6	19.2
70.339	1.33733	143	12.0	19.2
70.350	1.33716	160	12.6	19.2
70.360	1.33699	161	12.7	19.2
70.370	1.33682	157	12.5	19.2
70.380	1.33666	146	12.1	19.2
70.390	1.33649	131	11.4	19.2
70.400	1.33632	145	12.0	19.2
70.410	1.33616	170	13.0	19.2
70.420	1.33599	164	12.8	19.2
70.430	1.33582	170	13.0	19.2
70.440	1.33566	161	12.7	19.2
70.451	1.33549	137	11.7	19.2
70.461	1.33532	152	12.3	19.2
70.471	1.33516	162	12.7	19.2
70.481	1.33499	157	12.5	19.2
70.491	1.33482	149	12.2	19.2
70.501	1.33466	156	12.5	19.2
70.511	1.33449	157	12.5	19.2
70.521	1.33432	143	12.0	19.2
70.531	1.33416	147	12.1	19.2
70.541	1.33399	156	12.5	19.2
70.552	1.33382	143	12.0	19.2
70.562	1.33366	159	12.6	19.2
70.572	1.33349	146	12.1	19.2
70.582	1.33333	154	12.4	19.2
70.592	1.33316	147	12.1	19.2
70.602	1.33299	139	11.8	19.2
70.612	1.33283	168	13.0	19.2
70.622	1.33266	165	12.8	19.2
70.632	1.33250	137	11.7	19.2

70.642	1.33233	165	12.8	19.2
70.653	1.33217	147	12.1	19.2
70.663	1.33200	155	12.4	19.2
70.673	1.33183	138	11.7	19.2
70.683	1.33167	164	12.8	19.2
70.693	1.33150	156	12.5	19.2
70.703	1.33134	175	13.2	19.2
70.713	1.33117	153	12.4	19.2
70.723	1.33101	140	11.8	19.2
70.733	1.33084	161	12.7	19.2
70.743	1.33068	159	12.6	19.2
70.753	1.33051	153	12.4	19.2
70.764	1.33035	156	12.5	19.2
70.774	1.33018	157	12.5	19.2
70.784	1.33002	149	12.2	19.2
70.794	1.32985	144	12.0	19.2
70.804	1.32969	152	12.3	19.2
70.814	1.32952	175	13.2	19.2
70.824	1.32936	150	12.2	19.2
70.834	1.32919	150	12.2	19.2
70.844	1.32903	177	13.3	19.2
70.854	1.32886	152	12.3	19.2
70.865	1.32870	153	12.4	19.2
70.875	1.32853	165	12.8	19.2
70.885	1.32837	145	12.0	19.2
70.895	1.32820	145	12.0	19.2
70.905	1.32804	146	12.1	19.2
70.915	1.32788	144	12.0	19.2
70.925	1.32771	157	12.5	19.2
70.935	1.32755	163	12.8	19.2
70.945	1.32738	138	11.7	19.2
70.955	1.32722	147	12.1	19.2
70.966	1.32705	143	12.0	19.2
70.976	1.32689	135	11.6	19.2
70.986	1.32673	137	11.7	19.2
70.996	1.32656	134	11.6	19.2
71.006	1.32640	147	12.1	19.2
71.016	1.32623	149	12.2	19.2
71.026	1.32607	149	12.2	19.2
71.036	1.32591	130	11.4	19.2
71.046	1.32574	151	12.3	19.2
71.056	1.32558	161	12.7	19.2
71.067	1.32542	147	12.1	19.2
71.077	1.32525	151	12.3	19.2
71.087	1.32509	153	12.4	19.2
71.097	1.32493	156	12.5	19.2
71.107	1.32476	148	12.2	19.2
71.117	1.32460	153	12.4	19.2
71.127	1.32444	158	12.6	19.2
71.137	1.32427	138	11.7	19.2

71.147	1.32411	162	12.7	19.2
71.157	1.32395	155	12.4	19.2
71.168	1.32378	124	11.1	19.2
71.178	1.32362	134	11.6	19.2
71.188	1.32346	141	11.9	19.2
71.198	1.32329	151	12.3	19.2
71.208	1.32313	155	12.4	19.2
71.218	1.32297	152	12.3	19.2
71.228	1.32281	149	12.2	19.2
71.238	1.32264	143	12.0	19.2
71.248	1.32248	164	12.8	19.2
71.258	1.32232	154	12.4	19.2
71.269	1.32216	183	13.5	19.2
71.279	1.32199	132	11.5	19.2
71.289	1.32183	124	11.1	19.2
71.299	1.32167	148	12.2	19.2
71.309	1.32151	155	12.4	19.2
71.319	1.32134	154	12.4	19.2
71.329	1.32118	150	12.2	19.2
71.339	1.32102	174	13.2	19.2
71.349	1.32086	147	12.1	19.2
71.359	1.32069	140	11.8	19.2
71.370	1.32053	147	12.1	19.2
71.380	1.32037	161	12.7	19.2
71.390	1.32021	146	12.1	19.2
71.400	1.32005	136	11.7	19.2
71.410	1.31988	163	12.8	19.2
71.420	1.31972	156	12.5	19.2
71.430	1.31956	179	13.4	19.2
71.440	1.31940	135	11.6	19.2
71.450	1.31924	146	12.1	19.2
71.460	1.31908	142	11.9	19.2
71.471	1.31891	162	12.7	19.2
71.481	1.31875	151	12.3	19.2
71.491	1.31859	155	12.4	19.2
71.501	1.31843	153	12.4	19.2
71.511	1.31827	163	12.8	19.2
71.521	1.31811	149	12.2	19.2
71.531	1.31795	134	11.6	19.2
71.541	1.31778	166	12.9	19.2
71.551	1.31762	130	11.4	19.2
71.561	1.31746	166	12.9	19.2
71.572	1.31730	132	11.5	19.2
71.582	1.31714	145	12.0	19.2
71.592	1.31698	155	12.4	19.2
71.602	1.31682	149	12.2	19.2
71.612	1.31666	139	11.8	19.2
71.622	1.31650	157	12.5	19.2
71.632	1.31634	145	12.0	19.2
71.642	1.31617	137	11.7	19.2

71.652	1.31601	129	11.4	19.2
71.662	1.31585	156	12.5	19.2
71.673	1.31569	162	12.7	19.2
71.683	1.31553	140	11.8	19.2
71.693	1.31537	122	11.0	19.2
71.703	1.31521	146	12.1	19.2
71.713	1.31505	150	12.2	19.2
71.723	1.31489	150	12.2	19.2
71.733	1.31473	132	11.5	19.2
71.743	1.31457	153	12.4	19.2
71.753	1.31441	159	12.6	19.2
71.763	1.31425	138	11.7	19.2
71.774	1.31409	146	12.1	19.2
71.784	1.31393	156	12.5	19.2
71.794	1.31377	149	12.2	19.2
71.804	1.31361	131	11.4	19.2
71.814	1.31345	156	12.5	19.2
71.824	1.31329	160	12.6	19.2
71.834	1.31313	139	11.8	19.2
71.844	1.31297	137	11.7	19.2
71.854	1.31281	144	12.0	19.2
71.864	1.31265	163	12.8	19.2
71.875	1.31249	158	12.6	19.2
71.885	1.31233	157	12.5	19.2
71.895	1.31217	135	11.6	19.2
71.905	1.31201	143	12.0	19.2
71.915	1.31185	139	11.8	19.2
71.925	1.31169	131	11.4	19.2
71.935	1.31154	145	12.0	19.2
71.945	1.31138	156	12.5	19.2
71.955	1.31122	123	11.1	19.2
71.965	1.31106	140	11.8	19.2
71.976	1.31090	147	12.1	19.2
71.986	1.31074	145	12.0	19.2
71.996	1.31058	141	11.9	19.2
72.006	1.31042	147	12.1	19.2
72.016	1.31026	161	12.7	19.2
72.026	1.31010	150	12.2	19.2
72.036	1.30994	141	11.9	19.2
72.046	1.30979	126	11.2	19.2
72.056	1.30963	123	11.1	19.2
72.066	1.30947	145	12.0	19.2
72.076	1.30931	169	13.0	19.2
72.087	1.30915	146	12.1	19.2
72.097	1.30899	170	13.0	19.2
72.107	1.30883	143	12.0	19.2
72.117	1.30868	161	12.7	19.2
72.127	1.30852	142	11.9	19.2
72.137	1.30836	152	12.3	19.2
72.147	1.30820	144	12.0	19.2

72.157	1.30804	156	12.5	19.2
72.167	1.30788	153	12.4	19.2
72.177	1.30773	147	12.1	19.2
72.188	1.30757	155	12.4	19.2
72.198	1.30741	155	12.4	19.2
72.208	1.30725	141	11.9	19.2
72.218	1.30709	155	12.4	19.2
72.228	1.30694	143	12.0	19.2
72.238	1.30678	146	12.1	19.2
72.248	1.30662	157	12.5	19.2
72.258	1.30646	158	12.6	19.2
72.268	1.30631	148	12.2	19.2
72.278	1.30615	130	11.4	19.2
72.289	1.30599	134	11.6	19.2
72.299	1.30583	138	11.7	19.2
72.309	1.30567	152	12.3	19.2
72.319	1.30552	149	12.2	19.2
72.329	1.30536	143	12.0	19.2
72.339	1.30520	150	12.2	19.2
72.349	1.30505	151	12.3	19.2
72.359	1.30489	135	11.6	19.2
72.369	1.30473	126	11.2	19.2
72.379	1.30457	137	11.7	19.2
72.390	1.30442	151	12.3	19.2
72.400	1.30426	126	11.2	19.2
72.410	1.30410	146	12.1	19.2
72.420	1.30395	146	12.1	19.2
72.430	1.30379	125	11.2	19.2
72.440	1.30363	154	12.4	19.2
72.450	1.30347	137	11.7	19.2
72.460	1.30332	156	12.5	19.2
72.470	1.30316	128	11.3	19.2
72.480	1.30300	158	12.6	19.2
72.491	1.30285	145	12.0	19.2
72.501	1.30269	174	13.2	19.2
72.511	1.30253	148	12.2	19.2
72.521	1.30238	147	12.1	19.2
72.531	1.30222	138	11.7	19.2
72.541	1.30207	166	12.9	19.2
72.551	1.30191	147	12.1	19.2
72.561	1.30175	141	11.9	19.2
72.571	1.30160	144	12.0	19.2
72.581	1.30144	141	11.9	19.2
72.592	1.30128	151	12.3	19.2
72.602	1.30113	149	12.2	19.2
72.612	1.30097	143	12.0	19.2
72.622	1.30082	148	12.2	19.2
72.632	1.30066	145	12.0	19.2
72.642	1.30050	138	11.7	19.2
72.652	1.30035	152	12.3	19.2

72.662	1.30019	161	12.7	19.2
72.672	1.30004	165	12.8	19.2
72.682	1.29988	134	11.6	19.2
72.693	1.29972	158	12.6	19.2
72.703	1.29957	159	12.6	19.2
72.713	1.29941	140	11.8	19.2
72.723	1.29926	141	11.9	19.2
72.733	1.29910	164	12.8	19.2
72.743	1.29895	144	12.0	19.2
72.753	1.29879	160	12.6	19.2
72.763	1.29864	136	11.7	19.2
72.773	1.29848	137	11.7	19.2
72.783	1.29833	140	11.8	19.2
72.794	1.29817	136	11.7	19.2
72.804	1.29802	148	12.2	19.2
72.814	1.29786	155	12.4	19.2
72.824	1.29771	149	12.2	19.2
72.834	1.29755	146	12.1	19.2
72.844	1.29740	134	11.6	19.2
72.854	1.29724	130	11.4	19.2
72.864	1.29709	158	12.6	19.2
72.874	1.29693	161	12.7	19.2
72.884	1.29678	148	12.2	19.2
72.895	1.29662	143	12.0	19.2
72.905	1.29647	149	12.2	19.2
72.915	1.29631	151	12.3	19.2
72.925	1.29616	164	12.8	19.2
72.935	1.29600	131	11.4	19.2
72.945	1.29585	134	11.6	19.2
72.955	1.29569	124	11.1	19.2
72.965	1.29554	152	12.3	19.2
72.975	1.29538	150	12.2	19.2
72.985	1.29523	134	11.6	19.2
72.996	1.29508	158	12.6	19.2
73.006	1.29492	144	12.0	19.2
73.016	1.29477	133	11.5	19.2
73.026	1.29461	131	11.4	19.2
73.036	1.29446	133	11.5	19.2
73.046	1.29431	118	10.9	19.2
73.056	1.29415	146	12.1	19.2
73.066	1.29400	150	12.2	19.2
73.076	1.29384	131	11.4	19.2
73.086	1.29369	148	12.2	19.2
73.097	1.29354	144	12.0	19.2
73.107	1.29338	147	12.1	19.2
73.117	1.29323	151	12.3	19.2
73.127	1.29307	146	12.1	19.2
73.137	1.29292	153	12.4	19.2
73.147	1.29277	121	11.0	19.2
73.157	1.29261	138	11.7	19.2

73.167	1.29246	153	12.4	19.2
73.177	1.29231	141	11.9	19.2
73.187	1.29215	143	12.0	19.2
73.198	1.29200	137	11.7	19.2
73.208	1.29185	146	12.1	19.2
73.218	1.29169	149	12.2	19.2
73.228	1.29154	173	13.2	19.2
73.238	1.29139	154	12.4	19.2
73.248	1.29123	142	11.9	19.2
73.258	1.29108	140	11.8	19.2
73.268	1.29093	143	12.0	19.2
73.278	1.29077	135	11.6	19.2
73.288	1.29062	150	12.2	19.2
73.299	1.29047	159	12.6	19.2
73.309	1.29032	146	12.1	19.2
73.319	1.29016	132	11.5	19.2
73.329	1.29001	150	12.2	19.2
73.339	1.28986	168	13.0	19.2
73.349	1.28971	144	12.0	19.2
73.359	1.28955	163	12.8	19.2
73.369	1.28940	149	12.2	19.2
73.379	1.28925	152	12.3	19.2
73.389	1.28910	150	12.2	19.2
73.399	1.28894	155	12.4	19.2
73.410	1.28879	136	11.7	19.2
73.420	1.28864	129	11.4	19.2
73.430	1.28849	151	12.3	19.2
73.440	1.28833	165	12.8	19.2
73.450	1.28818	161	12.7	19.2
73.460	1.28803	142	11.9	19.2
73.470	1.28788	148	12.2	19.2
73.480	1.28772	128	11.3	19.2
73.490	1.28757	149	12.2	19.2
73.500	1.28742	122	11.0	19.2
73.511	1.28727	162	12.7	19.2
73.521	1.28712	165	12.8	19.2
73.531	1.28697	150	12.2	19.2
73.541	1.28681	141	11.9	19.2
73.551	1.28666	161	12.7	19.2
73.561	1.28651	158	12.6	19.2
73.571	1.28636	156	12.5	19.2
73.581	1.28621	158	12.6	19.2
73.591	1.28606	143	12.0	19.2
73.601	1.28590	154	12.4	19.2
73.612	1.28575	147	12.1	19.2
73.622	1.28560	121	11.0	19.2
73.632	1.28545	130	11.4	19.2
73.642	1.28530	148	12.2	19.2
73.652	1.28515	133	11.5	19.2
73.662	1.28500	142	11.9	19.2

73.672	1.28484	160	12.6	19.2
73.682	1.28469	135	11.6	19.2
73.692	1.28454	152	12.3	19.2
73.702	1.28439	162	12.7	19.2
73.713	1.28424	156	12.5	19.2
73.723	1.28409	132	11.5	19.2
73.733	1.28394	146	12.1	19.2
73.743	1.28379	147	12.1	19.2
73.753	1.28364	153	12.4	19.2
73.763	1.28349	137	11.7	19.2
73.773	1.28334	143	12.0	19.2
73.783	1.28318	118	10.9	19.2
73.793	1.28303	141	11.9	19.2
73.803	1.28288	137	11.7	19.2
73.814	1.28273	147	12.1	19.2
73.824	1.28258	146	12.1	19.2
73.834	1.28243	149	12.2	19.2
73.844	1.28228	153	12.4	19.2
73.854	1.28213	134	11.6	19.2
73.864	1.28198	147	12.1	19.2
73.874	1.28183	144	12.0	19.2
73.884	1.28168	137	11.7	19.2
73.894	1.28153	156	12.5	19.2
73.904	1.28138	151	12.3	19.2
73.915	1.28123	165	12.8	19.2
73.925	1.28108	156	12.5	19.2
73.935	1.28093	136	11.7	19.2
73.945	1.28078	124	11.1	19.2
73.955	1.28063	140	11.8	19.2
73.965	1.28048	157	12.5	19.2
73.975	1.28033	143	12.0	19.2
73.985	1.28018	152	12.3	19.2
73.995	1.28003	175	13.2	19.2
74.005	1.27988	156	12.5	19.2
74.016	1.27973	135	11.6	19.2
74.026	1.27958	156	12.5	19.2
74.036	1.27943	145	12.0	19.2
74.046	1.27928	148	12.2	19.2
74.056	1.27913	109	10.4	19.2
74.066	1.27898	142	11.9	19.2
74.076	1.27883	131	11.4	19.2
74.086	1.27868	139	11.8	19.2
74.096	1.27854	159	12.6	19.2
74.106	1.27839	148	12.2	19.2
74.117	1.27824	172	13.1	19.2
74.127	1.27809	150	12.2	19.2
74.137	1.27794	131	11.4	19.2
74.147	1.27779	157	12.5	19.2
74.157	1.27764	138	11.7	19.2
74.167	1.27749	134	11.6	19.2

74.177	1.27734	149	12.2	19.2
74.187	1.27719	137	11.7	19.2
74.197	1.27704	145	12.0	19.2
74.207	1.27690	152	12.3	19.2
74.218	1.27675	129	11.4	19.2
74.228	1.27660	143	12.0	19.2
74.238	1.27645	136	11.7	19.2
74.248	1.27630	146	12.1	19.2
74.258	1.27615	147	12.1	19.2
74.268	1.27600	160	12.6	19.2
74.278	1.27586	139	11.8	19.2
74.288	1.27571	137	11.7	19.2
74.298	1.27556	137	11.7	19.2
74.308	1.27541	145	12.0	19.2
74.319	1.27526	149	12.2	19.2
74.329	1.27511	123	11.1	19.2
74.339	1.27497	143	12.0	19.2
74.349	1.27482	165	12.8	19.2
74.359	1.27467	139	11.8	19.2
74.369	1.27452	143	12.0	19.2
74.379	1.27437	144	12.0	19.2
74.389	1.27423	125	11.2	19.2
74.399	1.27408	147	12.1	19.2
74.409	1.27393	166	12.9	19.2
74.420	1.27378	142	11.9	19.2
74.430	1.27363	154	12.4	19.2
74.440	1.27349	134	11.6	19.2
74.450	1.27334	136	11.7	19.2
74.460	1.27319	144	12.0	19.2
74.470	1.27304	147	12.1	19.2
74.480	1.27290	143	12.0	19.2
74.490	1.27275	148	12.2	19.2
74.500	1.27260	150	12.2	19.2
74.510	1.27245	130	11.4	19.2
74.521	1.27231	137	11.7	19.2
74.531	1.27216	140	11.8	19.2
74.541	1.27201	164	12.8	19.2
74.551	1.27186	130	11.4	19.2
74.561	1.27172	144	12.0	19.2
74.571	1.27157	151	12.3	19.2
74.581	1.27142	132	11.5	19.2
74.591	1.27127	130	11.4	19.2
74.601	1.27113	150	12.2	19.2
74.611	1.27098	146	12.1	19.2
74.622	1.27083	143	12.0	19.2
74.632	1.27069	151	12.3	19.2
74.642	1.27054	141	11.9	19.2
74.652	1.27039	143	12.0	19.2
74.662	1.27025	126	11.2	19.2
74.672	1.27010	146	12.1	19.2

74.682	1.26995	157	12.5	19.2
74.692	1.26981	122	11.0	19.2
74.702	1.26966	158	12.6	19.2
74.712	1.26951	136	11.7	19.2
74.722	1.26937	132	11.5	19.2
74.733	1.26922	143	12.0	19.2
74.743	1.26907	155	12.4	19.2
74.753	1.26893	125	11.2	19.2
74.763	1.26878	145	12.0	19.2
74.773	1.26863	135	11.6	19.2
74.783	1.26849	123	11.1	19.2
74.793	1.26834	139	11.8	19.2
74.803	1.26820	137	11.7	19.2
74.813	1.26805	146	12.1	19.2
74.823	1.26790	133	11.5	19.2
74.834	1.26776	153	12.4	19.2
74.844	1.26761	131	11.4	19.2
74.854	1.26746	127	11.3	19.2
74.864	1.26732	152	12.3	19.2
74.874	1.26717	153	12.4	19.2
74.884	1.26703	152	12.3	19.2
74.894	1.26688	135	11.6	19.2
74.904	1.26674	139	11.8	19.2
74.914	1.26659	152	12.3	19.2
74.924	1.26644	134	11.6	19.2
74.935	1.26630	145	12.0	19.2
74.945	1.26615	134	11.6	19.2
74.955	1.26601	130	11.4	19.2
74.965	1.26586	163	12.8	19.2
74.975	1.26572	150	12.2	19.2
74.985	1.26557	129	11.4	19.2
74.995	1.26543	142	11.9	19.2
75.005	1.26528	141	11.9	19.2
75.015	1.26513	140	11.8	19.2
75.025	1.26499	148	12.2	19.2
75.036	1.26484	138	11.7	19.2
75.046	1.26470	143	12.0	19.2
75.056	1.26455	137	11.7	19.2
75.066	1.26441	133	11.5	19.2
75.076	1.26426	135	11.6	19.2
75.086	1.26412	126	11.2	19.2
75.096	1.26397	138	11.7	19.2
75.106	1.26383	154	12.4	19.2
75.116	1.26368	162	12.7	19.2
75.126	1.26354	124	11.1	19.2
75.137	1.26339	142	11.9	19.2
75.147	1.26325	133	11.5	19.2
75.157	1.26311	157	12.5	19.2
75.167	1.26296	135	11.6	19.2
75.177	1.26282	129	11.4	19.2

75.187	1.26267	134	11.6	19.2
75.197	1.26253	140	11.8	19.2
75.207	1.26238	146	12.1	19.2
75.217	1.26224	150	12.2	19.2
75.227	1.26209	147	12.1	19.2
75.238	1.26195	133	11.5	19.2
75.248	1.26181	140	11.8	19.2
75.258	1.26166	143	12.0	19.2
75.268	1.26152	131	11.4	19.2
75.278	1.26137	148	12.2	19.2
75.288	1.26123	151	12.3	19.2
75.298	1.26108	145	12.0	19.2
75.308	1.26094	143	12.0	19.2
75.318	1.26080	141	11.9	19.2
75.328	1.26065	158	12.6	19.2
75.339	1.26051	130	11.4	19.2
75.349	1.26036	119	10.9	19.2
75.359	1.26022	131	11.4	19.2
75.369	1.26008	146	12.1	19.2
75.379	1.25993	131	11.4	19.2
75.389	1.25979	160	12.6	19.2
75.399	1.25965	135	11.6	19.2
75.409	1.25950	135	11.6	19.2
75.419	1.25936	138	11.7	19.2
75.429	1.25921	151	12.3	19.2
75.440	1.25907	131	11.4	19.2
75.450	1.25893	153	12.4	19.2
75.460	1.25878	148	12.2	19.2
75.470	1.25864	145	12.0	19.2
75.480	1.25850	153	12.4	19.2
75.490	1.25835	142	11.9	19.2
75.500	1.25821	135	11.6	19.2
75.510	1.25807	134	11.6	19.2
75.520	1.25793	128	11.3	19.2
75.530	1.25778	150	12.2	19.2
75.541	1.25764	148	12.2	19.2
75.551	1.25750	161	12.7	19.2
75.561	1.25735	124	11.1	19.2
75.571	1.25721	148	12.2	19.2
75.581	1.25707	134	11.6	19.2
75.591	1.25692	142	11.9	19.2
75.601	1.25678	149	12.2	19.2
75.611	1.25664	136	11.7	19.2
75.621	1.25650	135	11.6	19.2
75.631	1.25635	147	12.1	19.2
75.642	1.25621	138	11.7	19.2
75.652	1.25607	136	11.7	19.2
75.662	1.25593	146	12.1	19.2
75.672	1.25578	158	12.6	19.2
75.682	1.25564	132	11.5	19.2

75.692	1.25550	135	11.6	19.2
75.702	1.25536	161	12.7	19.2
75.712	1.25521	151	12.3	19.2
75.722	1.25507	164	12.8	19.2
75.732	1.25493	157	12.5	19.2
75.743	1.25479	144	12.0	19.2
75.753	1.25464	136	11.7	19.2
75.763	1.25450	144	12.0	19.2
75.773	1.25436	134	11.6	19.2
75.783	1.25422	151	12.3	19.2
75.793	1.25408	127	11.3	19.2
75.803	1.25393	157	12.5	19.2
75.813	1.25379	131	11.4	19.2
75.823	1.25365	156	12.5	19.2
75.833	1.25351	137	11.7	19.2
75.844	1.25337	160	12.6	19.2
75.854	1.25322	139	11.8	19.2
75.864	1.25308	145	12.0	19.2
75.874	1.25294	147	12.1	19.2
75.884	1.25280	147	12.1	19.2
75.894	1.25266	125	11.2	19.2
75.904	1.25252	141	11.9	19.2
75.914	1.25238	131	11.4	19.2
75.924	1.25223	144	12.0	19.2
75.934	1.25209	137	11.7	19.2
75.945	1.25195	149	12.2	19.2
75.955	1.25181	128	11.3	19.2
75.965	1.25167	134	11.6	19.2
75.975	1.25153	158	12.6	19.2
75.985	1.25139	142	11.9	19.2
75.995	1.25124	133	11.5	19.2
76.005	1.25110	126	11.2	19.2
76.015	1.25096	127	11.3	19.2
76.025	1.25082	127	11.3	19.2
76.035	1.25068	120	11.0	19.2
76.045	1.25054	146	12.1	19.2
76.056	1.25040	136	11.7	19.2
76.066	1.25026	129	11.4	19.2
76.076	1.25012	146	12.1	19.2
76.086	1.24998	142	11.9	19.2
76.096	1.24983	151	12.3	19.2
76.106	1.24969	151	12.3	19.2
76.116	1.24955	153	12.4	19.2
76.126	1.24941	140	11.8	19.2
76.136	1.24927	146	12.1	19.2
76.146	1.24913	139	11.8	19.2
76.157	1.24899	143	12.0	19.2
76.167	1.24885	144	12.0	19.2
76.177	1.24871	131	11.4	19.2
76.187	1.24857	157	12.5	19.2

76.197	1.24843	137	11.7	19.2
76.207	1.24829	137	11.7	19.2
76.217	1.24815	144	12.0	19.2
76.227	1.24801	124	11.1	19.2
76.237	1.24787	138	11.7	19.2
76.247	1.24773	161	12.7	19.2
76.258	1.24759	127	11.3	19.2
76.268	1.24745	149	12.2	19.2
76.278	1.24731	151	12.3	19.2
76.288	1.24717	153	12.4	19.2
76.298	1.24703	159	12.6	19.2
76.308	1.24689	138	11.7	19.2
76.318	1.24675	141	11.9	19.2
76.328	1.24661	133	11.5	19.2
76.338	1.24647	151	12.3	19.2
76.348	1.24633	116	10.8	19.2
76.359	1.24619	147	12.1	19.2
76.369	1.24605	156	12.5	19.2
76.379	1.24591	137	11.7	19.2
76.389	1.24577	150	12.2	19.2
76.399	1.24563	138	11.7	19.2
76.409	1.24549	147	12.1	19.2
76.419	1.24535	129	11.4	19.2
76.429	1.24521	154	12.4	19.2
76.439	1.24507	127	11.3	19.2
76.449	1.24493	144	12.0	19.2
76.460	1.24479	133	11.5	19.2
76.470	1.24466	142	11.9	19.2
76.480	1.24452	141	11.9	19.2
76.490	1.24438	137	11.7	19.2
76.500	1.24424	157	12.5	19.2
76.510	1.24410	127	11.3	19.2
76.520	1.24396	142	11.9	19.2
76.530	1.24382	132	11.5	19.2
76.540	1.24368	149	12.2	19.2
76.550	1.24354	142	11.9	19.2
76.561	1.24340	149	12.2	19.2
76.571	1.24327	146	12.1	19.2
76.581	1.24313	150	12.2	19.2
76.591	1.24299	138	11.7	19.2
76.601	1.24285	141	11.9	19.2
76.611	1.24271	136	11.7	19.2
76.621	1.24257	117	10.8	19.2
76.631	1.24243	156	12.5	19.2
76.641	1.24229	146	12.1	19.2
76.651	1.24216	139	11.8	19.2
76.662	1.24202	139	11.8	19.2
76.672	1.24188	157	12.5	19.2
76.682	1.24174	140	11.8	19.2
76.692	1.24160	157	12.5	19.2

76.702	1.24146	141	11.9	19.2
76.712	1.24133	109	10.4	19.2
76.722	1.24119	136	11.7	19.2
76.732	1.24105	139	11.8	19.2
76.742	1.24091	142	11.9	19.2
76.752	1.24077	141	11.9	19.2
76.763	1.24064	131	11.4	19.2
76.773	1.24050	134	11.6	19.2
76.783	1.24036	126	11.2	19.2
76.793	1.24022	141	11.9	19.2
76.803	1.24008	145	12.0	19.2
76.813	1.23995	150	12.2	19.2
76.823	1.23981	143	12.0	19.2
76.833	1.23967	138	11.7	19.2
76.843	1.23953	147	12.1	19.2
76.853	1.23939	145	12.0	19.2
76.864	1.23926	126	11.2	19.2
76.874	1.23912	118	10.9	19.2
76.884	1.23898	162	12.7	19.2
76.894	1.23884	127	11.3	19.2
76.904	1.23871	141	11.9	19.2
76.914	1.23857	141	11.9	19.2
76.924	1.23843	138	11.7	19.2
76.934	1.23829	145	12.0	19.2
76.944	1.23816	154	12.4	19.2
76.954	1.23802	149	12.2	19.2
76.965	1.23788	134	11.6	19.2
76.975	1.23775	134	11.6	19.2
76.985	1.23761	127	11.3	19.2
76.995	1.23747	137	11.7	19.2
77.005	1.23733	127	11.3	19.2
77.015	1.23720	140	11.8	19.2
77.025	1.23706	144	12.0	19.2
77.035	1.23692	134	11.6	19.2
77.045	1.23679	149	12.2	19.2
77.055	1.23665	131	11.4	19.2
77.066	1.23651	150	12.2	19.2
77.076	1.23638	148	12.2	19.2
77.086	1.23624	126	11.2	19.2
77.096	1.23610	124	11.1	19.2
77.106	1.23596	115	10.7	19.2
77.116	1.23583	140	11.8	19.2
77.126	1.23569	149	12.2	19.2
77.136	1.23556	131	11.4	19.2
77.146	1.23542	123	11.1	19.2
77.156	1.23528	169	13.0	19.2
77.167	1.23515	162	12.7	19.2
77.177	1.23501	138	11.7	19.2
77.187	1.23487	138	11.7	19.2
77.197	1.23474	127	11.3	19.2

77.207	1.23460	137	11.7	19.2
77.217	1.23446	175	13.2	19.2
77.227	1.23433	138	11.7	19.2
77.237	1.23419	144	12.0	19.2
77.247	1.23406	136	11.7	19.2
77.257	1.23392	139	11.8	19.2
77.268	1.23378	161	12.7	19.2
77.278	1.23365	123	11.1	19.2
77.288	1.23351	162	12.7	19.2
77.298	1.23338	139	11.8	19.2
77.308	1.23324	152	12.3	19.2
77.318	1.23310	132	11.5	19.2
77.328	1.23297	158	12.6	19.2
77.338	1.23283	139	11.8	19.2
77.348	1.23270	128	11.3	19.2
77.358	1.23256	158	12.6	19.2
77.368	1.23242	141	11.9	19.2
77.379	1.23229	145	12.0	19.2
77.389	1.23215	137	11.7	19.2
77.399	1.23202	143	12.0	19.2
77.409	1.23188	136	11.7	19.2
77.419	1.23175	122	11.0	19.2
77.429	1.23161	120	11.0	19.2
77.439	1.23148	128	11.3	19.2
77.449	1.23134	112	10.6	19.2
77.459	1.23121	141	11.9	19.2
77.469	1.23107	156	12.5	19.2
77.480	1.23093	126	11.2	19.2
77.490	1.23080	131	11.4	19.2
77.500	1.23066	145	12.0	19.2
77.510	1.23053	153	12.4	19.2
77.520	1.23039	139	11.8	19.2
77.530	1.23026	148	12.2	19.2
77.540	1.23012	141	11.9	19.2
77.550	1.22999	128	11.3	19.2
77.560	1.22985	161	12.7	19.2
77.570	1.22972	141	11.9	19.2
77.581	1.22958	151	12.3	19.2
77.591	1.22945	133	11.5	19.2
77.601	1.22932	156	12.5	19.2
77.611	1.22918	125	11.2	19.2
77.621	1.22905	151	12.3	19.2
77.631	1.22891	129	11.4	19.2
77.641	1.22878	148	12.2	19.2
77.651	1.22864	143	12.0	19.2
77.661	1.22851	119	10.9	19.2
77.671	1.22837	131	11.4	19.2
77.682	1.22824	117	10.8	19.2
77.692	1.22810	140	11.8	19.2
77.702	1.22797	124	11.1	19.2

77.712	1.22784	156	12.5	19.2
77.722	1.22770	136	11.7	19.2
77.732	1.22757	128	11.3	19.2
77.742	1.22743	155	12.4	19.2
77.752	1.22730	145	12.0	19.2
77.762	1.22716	128	11.3	19.2
77.772	1.22703	143	12.0	19.2
77.783	1.22690	143	12.0	19.2
77.793	1.22676	145	12.0	19.2
77.803	1.22663	141	11.9	19.2
77.813	1.22649	116	10.8	19.2
77.823	1.22636	151	12.3	19.2
77.833	1.22623	129	11.4	19.2
77.843	1.22609	133	11.5	19.2
77.853	1.22596	142	11.9	19.2
77.863	1.22582	150	12.2	19.2
77.873	1.22569	152	12.3	19.2
77.884	1.22556	136	11.7	19.2
77.894	1.22542	117	10.8	19.2
77.904	1.22529	142	11.9	19.2
77.914	1.22516	153	12.4	19.2
77.924	1.22502	144	12.0	19.2
77.934	1.22489	145	12.0	19.2
77.944	1.22476	149	12.2	19.2
77.954	1.22462	127	11.3	19.2
77.964	1.22449	153	12.4	19.2
77.974	1.22436	133	11.5	19.2
77.985	1.22422	151	12.3	19.2
77.995	1.22409	140	11.8	19.2
78.005	1.22396	148	12.2	19.2
78.015	1.22382	156	12.5	19.2
78.025	1.22369	161	12.7	19.2
78.035	1.22356	158	12.6	19.2
78.045	1.22342	150	12.2	19.2
78.055	1.22329	149	12.2	19.2
78.065	1.22316	139	11.8	19.2
78.075	1.22302	161	12.7	19.2
78.086	1.22289	159	12.6	19.2
78.096	1.22276	123	11.1	19.2
78.106	1.22263	149	12.2	19.2
78.116	1.22249	126	11.2	19.2
78.126	1.22236	147	12.1	19.2
78.136	1.22223	158	12.6	19.2
78.146	1.22210	137	11.7	19.2
78.156	1.22196	125	11.2	19.2
78.166	1.22183	142	11.9	19.2
78.176	1.22170	150	12.2	19.2
78.187	1.22156	143	12.0	19.2
78.197	1.22143	149	12.2	19.2
78.207	1.22130	139	11.8	19.2

78.217	1.22117	145	12.0	19.2
78.227	1.22104	135	11.6	19.2
78.237	1.22090	143	12.0	19.2
78.247	1.22077	153	12.4	19.2
78.257	1.22064	130	11.4	19.2
78.267	1.22051	143	12.0	19.2
78.277	1.22037	140	11.8	19.2
78.288	1.22024	131	11.4	19.2
78.298	1.22011	144	12.0	19.2
78.308	1.21998	154	12.4	19.2
78.318	1.21985	145	12.0	19.2
78.328	1.21971	138	11.7	19.2
78.338	1.21958	135	11.6	19.2
78.348	1.21945	151	12.3	19.2
78.358	1.21932	143	12.0	19.2
78.368	1.21919	134	11.6	19.2
78.378	1.21905	155	12.4	19.2
78.389	1.21892	125	11.2	19.2
78.399	1.21879	124	11.1	19.2
78.409	1.21866	140	11.8	19.2
78.419	1.21853	165	12.8	19.2
78.429	1.21840	140	11.8	19.2
78.439	1.21826	136	11.7	19.2
78.449	1.21813	127	11.3	19.2
78.459	1.21800	139	11.8	19.2
78.469	1.21787	145	12.0	19.2
78.479	1.21774	139	11.8	19.2
78.490	1.21761	144	12.0	19.2
78.500	1.21748	156	12.5	19.2
78.510	1.21734	145	12.0	19.2
78.520	1.21721	140	11.8	19.2
78.530	1.21708	134	11.6	19.2
78.540	1.21695	157	12.5	19.2
78.550	1.21682	138	11.7	19.2
78.560	1.21669	140	11.8	19.2
78.570	1.21656	108	10.4	19.2
78.580	1.21643	104	10.2	19.2
78.591	1.21630	150	12.2	19.2
78.601	1.21616	152	12.3	19.2
78.611	1.21603	144	12.0	19.2
78.621	1.21590	152	12.3	19.2
78.631	1.21577	136	11.7	19.2
78.641	1.21564	149	12.2	19.2
78.651	1.21551	142	11.9	19.2
78.661	1.21538	160	12.6	19.2
78.671	1.21525	150	12.2	19.2
78.681	1.21512	150	12.2	19.2
78.691	1.21499	132	11.5	19.2
78.702	1.21486	133	11.5	19.2
78.712	1.21473	123	11.1	19.2

78.722	1.21460	147	12.1	19.2
78.732	1.21446	154	12.4	19.2
78.742	1.21433	116	10.8	19.2
78.752	1.21420	157	12.5	19.2
78.762	1.21407	145	12.0	19.2
78.772	1.21394	123	11.1	19.2
78.782	1.21381	114	10.7	19.2
78.792	1.21368	132	11.5	19.2
78.803	1.21355	144	12.0	19.2
78.813	1.21342	141	11.9	19.2
78.823	1.21329	125	11.2	19.2
78.833	1.21316	146	12.1	19.2
78.843	1.21303	127	11.3	19.2
78.853	1.21290	135	11.6	19.2
78.863	1.21277	138	11.7	19.2
78.873	1.21264	166	12.9	19.2
78.883	1.21251	135	11.6	19.2
78.893	1.21238	119	10.9	19.2
78.904	1.21225	145	12.0	19.2
78.914	1.21212	133	11.5	19.2
78.924	1.21199	145	12.0	19.2
78.934	1.21186	150	12.2	19.2
78.944	1.21173	127	11.3	19.2
78.954	1.21160	135	11.6	19.2
78.964	1.21147	153	12.4	19.2
78.974	1.21134	134	11.6	19.2
78.984	1.21121	173	13.2	19.2
78.994	1.21109	130	11.4	19.2
79.005	1.21096	140	11.8	19.2
79.015	1.21083	134	11.6	19.2
79.025	1.21070	136	11.7	19.2
79.035	1.21057	151	12.3	19.2
79.045	1.21044	126	11.2	19.2
79.055	1.21031	139	11.8	19.2
79.065	1.21018	146	12.1	19.2
79.075	1.21005	138	11.7	19.2
79.085	1.20992	145	12.0	19.2
79.095	1.20979	134	11.6	19.2
79.106	1.20966	130	11.4	19.2
79.116	1.20953	149	12.2	19.2
79.126	1.20941	120	11.0	19.2
79.136	1.20928	153	12.4	19.2
79.146	1.20915	148	12.2	19.2
79.156	1.20902	114	10.7	19.2
79.166	1.20889	172	13.1	19.2
79.176	1.20876	131	11.4	19.2
79.186	1.20863	130	11.4	19.2
79.196	1.20850	126	11.2	19.2
79.207	1.20837	122	11.0	19.2
79.217	1.20825	147	12.1	19.2

79.227	1.20812	136	11.7	19.2
79.237	1.20799	125	11.2	19.2
79.247	1.20786	131	11.4	19.2
79.257	1.20773	140	11.8	19.2
79.267	1.20760	142	11.9	19.2
79.277	1.20747	132	11.5	19.2
79.287	1.20735	122	11.0	19.2
79.297	1.20722	141	11.9	19.2
79.308	1.20709	141	11.9	19.2
79.318	1.20696	149	12.2	19.2
79.328	1.20683	133	11.5	19.2
79.338	1.20670	146	12.1	19.2
79.348	1.20658	151	12.3	19.2
79.358	1.20645	139	11.8	19.2
79.368	1.20632	150	12.2	19.2
79.378	1.20619	124	11.1	19.2
79.388	1.20606	143	12.0	19.2
79.398	1.20594	132	11.5	19.2
79.409	1.20581	146	12.1	19.2
79.419	1.20568	119	10.9	19.2
79.429	1.20555	123	11.1	19.2
79.439	1.20542	146	12.1	19.2
79.449	1.20530	126	11.2	19.2
79.459	1.20517	147	12.1	19.2
79.469	1.20504	133	11.5	19.2
79.479	1.20491	125	11.2	19.2
79.489	1.20478	136	11.7	19.2
79.499	1.20466	115	10.7	19.2
79.510	1.20453	142	11.9	19.2
79.520	1.20440	150	12.2	19.2
79.530	1.20427	124	11.1	19.2
79.540	1.20415	132	11.5	19.2
79.550	1.20402	122	11.0	19.2
79.560	1.20389	123	11.1	19.2
79.570	1.20376	132	11.5	19.2
79.580	1.20364	110	10.5	19.2
79.590	1.20351	144	12.0	19.2
79.600	1.20338	147	12.1	19.2
79.611	1.20325	140	11.8	19.2
79.621	1.20313	132	11.5	19.2
79.631	1.20300	148	12.2	19.2
79.641	1.20287	156	12.5	19.2
79.651	1.20275	127	11.3	19.2
79.661	1.20262	140	11.8	19.2
79.671	1.20249	155	12.4	19.2
79.681	1.20236	138	11.7	19.2
79.691	1.20224	134	11.6	19.2
79.701	1.20211	150	12.2	19.2
79.712	1.20198	137	11.7	19.2
79.722	1.20186	137	11.7	19.2

79.732	1.20173	150	12.2	19.2
79.742	1.20160	126	11.2	19.2
79.752	1.20148	131	11.4	19.2
79.762	1.20135	147	12.1	19.2
79.772	1.20122	139	11.8	19.2
79.782	1.20110	131	11.4	19.2
79.792	1.20097	171	13.1	19.2
79.802	1.20084	134	11.6	19.2
79.813	1.20072	139	11.8	19.2
79.823	1.20059	149	12.2	19.2
79.833	1.20046	147	12.1	19.2
79.843	1.20034	132	11.5	19.2
79.853	1.20021	155	12.4	19.2
79.863	1.20008	126	11.2	19.2
79.873	1.19996	147	12.1	19.2
79.883	1.19983	146	12.1	19.2
79.893	1.19971	147	12.1	19.2
79.903	1.19958	125	11.2	19.2
79.913	1.19945	139	11.8	19.2
79.924	1.19933	126	11.2	19.2
79.934	1.19920	143	12.0	19.2
79.944	1.19908	132	11.5	19.2
79.954	1.19895	154	12.4	19.2
79.964	1.19882	144	12.0	19.2
79.974	1.19870	134	11.6	19.2
79.984	1.19857	155	12.4	19.2
79.994	1.19845	150	12.2	19.2
80.004	1.19832	126	11.2	19.2
80.014	1.19819	143	12.0	19.2
80.025	1.19807	139	11.8	19.2
80.035	1.19794	133	11.5	19.2
80.045	1.19782	120	11.0	19.2
80.055	1.19769	153	12.4	19.2
80.065	1.19756	143	12.0	19.2
80.075	1.19744	140	11.8	19.2
80.085	1.19731	135	11.6	19.2
80.095	1.19719	156	12.5	19.2
80.105	1.19706	148	12.2	19.2
80.115	1.19694	154	12.4	19.2
80.126	1.19681	139	11.8	19.2
80.136	1.19669	135	11.6	19.2
80.146	1.19656	135	11.6	19.2
80.156	1.19644	140	11.8	19.2
80.166	1.19631	120	11.0	19.2
80.176	1.19618	129	11.4	19.2
80.186	1.19606	156	12.5	19.2
80.196	1.19593	138	11.7	19.2
80.206	1.19581	144	12.0	19.2
80.216	1.19568	125	11.2	19.2
80.227	1.19556	156	12.5	19.2

80.237	1.19543	163	12.8	19.2
80.247	1.19531	145	12.0	19.2
80.257	1.19518	137	11.7	19.2
80.267	1.19506	127	11.3	19.2
80.277	1.19493	131	11.4	19.2
80.287	1.19481	139	11.8	19.2
80.297	1.19468	126	11.2	19.2
80.307	1.19456	120	11.0	19.2
80.317	1.19443	152	12.3	19.2
80.328	1.19431	149	12.2	19.2
80.338	1.19419	126	11.2	19.2
80.348	1.19406	139	11.8	19.2
80.358	1.19394	135	11.6	19.2
80.368	1.19381	140	11.8	19.2
80.378	1.19369	146	12.1	19.2
80.388	1.19356	131	11.4	19.2
80.398	1.19344	125	11.2	19.2
80.408	1.19331	162	12.7	19.2
80.418	1.19319	136	11.7	19.2
80.429	1.19306	150	12.2	19.2
80.439	1.19294	139	11.8	19.2
80.449	1.19282	146	12.1	19.2
80.459	1.19269	139	11.8	19.2
80.469	1.19257	159	12.6	19.2
80.479	1.19244	130	11.4	19.2
80.489	1.19232	133	11.5	19.2
80.499	1.19220	130	11.4	19.2
80.509	1.19207	145	12.0	19.2
80.519	1.19195	139	11.8	19.2
80.530	1.19182	112	10.6	19.2
80.540	1.19170	149	12.2	19.2
80.550	1.19158	149	12.2	19.2
80.560	1.19145	142	11.9	19.2
80.570	1.19133	151	12.3	19.2
80.580	1.19120	133	11.5	19.2
80.590	1.19108	144	12.0	19.2
80.600	1.19096	144	12.0	19.2
80.610	1.19083	131	11.4	19.2
80.620	1.19071	133	11.5	19.2
80.631	1.19058	126	11.2	19.2
80.641	1.19046	145	12.0	19.2
80.651	1.19034	163	12.8	19.2
80.661	1.19021	138	11.7	19.2
80.671	1.19009	142	11.9	19.2
80.681	1.18997	143	12.0	19.2
80.691	1.18984	134	11.6	19.2
80.701	1.18972	136	11.7	19.2
80.711	1.18960	144	12.0	19.2
80.721	1.18947	150	12.2	19.2
80.732	1.18935	131	11.4	19.2

80.742	1.18923	144	12.0	19.2
80.752	1.18910	141	11.9	19.2
80.762	1.18898	119	10.9	19.2
80.772	1.18886	135	11.6	19.2
80.782	1.18873	163	12.8	19.2
80.792	1.18861	137	11.7	19.2
80.802	1.18849	129	11.4	19.2
80.812	1.18836	140	11.8	19.2
80.822	1.18824	143	12.0	19.2
80.833	1.18812	157	12.5	19.2
80.843	1.18800	124	11.1	19.2
80.853	1.18787	133	11.5	19.2
80.863	1.18775	135	11.6	19.2
80.873	1.18763	143	12.0	19.2
80.883	1.18750	143	12.0	19.2
80.893	1.18738	128	11.3	19.2
80.903	1.18726	128	11.3	19.2
80.913	1.18714	135	11.6	19.2
80.923	1.18701	121	11.0	19.2
80.934	1.18689	140	11.8	19.2
80.944	1.18677	138	11.7	19.2
80.954	1.18665	137	11.7	19.2
80.964	1.18652	138	11.7	19.2
80.974	1.18640	139	11.8	19.2
80.984	1.18628	144	12.0	19.2
80.994	1.18616	136	11.7	19.2
81.004	1.18603	137	11.7	19.2
81.014	1.18591	137	11.7	19.2
81.024	1.18579	136	11.7	19.2
81.035	1.18567	150	12.2	19.2
81.045	1.18554	144	12.0	19.2
81.055	1.18542	155	12.4	19.2
81.065	1.18530	141	11.9	19.2
81.075	1.18518	133	11.5	19.2
81.085	1.18506	149	12.2	19.2
81.095	1.18493	151	12.3	19.2
81.105	1.18481	134	11.6	19.2
81.115	1.18469	134	11.6	19.2
81.125	1.18457	146	12.1	19.2
81.136	1.18444	134	11.6	19.2
81.146	1.18432	133	11.5	19.2
81.156	1.18420	136	11.7	19.2
81.166	1.18408	131	11.4	19.2
81.176	1.18396	140	11.8	19.2
81.186	1.18384	139	11.8	19.2
81.196	1.18371	137	11.7	19.2
81.206	1.18359	135	11.6	19.2
81.216	1.18347	138	11.7	19.2
81.226	1.18335	131	11.4	19.2
81.236	1.18323	129	11.4	19.2

81.247	1.18311	137	11.7	19.2
81.257	1.18298	139	11.8	19.2
81.267	1.18286	123	11.1	19.2
81.277	1.18274	129	11.4	19.2
81.287	1.18262	154	12.4	19.2
81.297	1.18250	163	12.8	19.2
81.307	1.18238	140	11.8	19.2
81.317	1.18226	147	12.1	19.2
81.327	1.18213	150	12.2	19.2
81.337	1.18201	146	12.1	19.2
81.348	1.18189	161	12.7	19.2
81.358	1.18177	135	11.6	19.2
81.368	1.18165	134	11.6	19.2
81.378	1.18153	137	11.7	19.2
81.388	1.18141	126	11.2	19.2
81.398	1.18129	127	11.3	19.2
81.408	1.18117	148	12.2	19.2
81.418	1.18104	132	11.5	19.2
81.428	1.18092	135	11.6	19.2
81.438	1.18080	152	12.3	19.2
81.449	1.18068	131	11.4	19.2
81.459	1.18056	151	12.3	19.2
81.469	1.18044	129	11.4	19.2
81.479	1.18032	124	11.1	19.2
81.489	1.18020	147	12.1	19.2
81.499	1.18008	141	11.9	19.2
81.509	1.17996	132	11.5	19.2
81.519	1.17984	132	11.5	19.2
81.529	1.17972	151	12.3	19.2
81.539	1.17960	148	12.2	19.2
81.550	1.17947	135	11.6	19.2
81.560	1.17935	123	11.1	19.2
81.570	1.17923	140	11.8	19.2
81.580	1.17911	131	11.4	19.2
81.590	1.17899	131	11.4	19.2
81.600	1.17887	163	12.8	19.2
81.610	1.17875	125	11.2	19.2
81.620	1.17863	135	11.6	19.2
81.630	1.17851	136	11.7	19.2
81.640	1.17839	150	12.2	19.2
81.651	1.17827	149	12.2	19.2
81.661	1.17815	133	11.5	19.2
81.671	1.17803	146	12.1	19.2
81.681	1.17791	146	12.1	19.2
81.691	1.17779	144	12.0	19.2
81.701	1.17767	144	12.0	19.2
81.711	1.17755	126	11.2	19.2
81.721	1.17743	135	11.6	19.2
81.731	1.17731	141	11.9	19.2
81.741	1.17719	142	11.9	19.2

81.752	1.17707	126	11.2	19.2
81.762	1.17695	130	11.4	19.2
81.772	1.17683	152	12.3	19.2
81.782	1.17671	128	11.3	19.2
81.792	1.17659	143	12.0	19.2
81.802	1.17647	147	12.1	19.2
81.812	1.17635	138	11.7	19.2
81.822	1.17623	125	11.2	19.2
81.832	1.17611	135	11.6	19.2
81.842	1.17599	137	11.7	19.2
81.853	1.17587	141	11.9	19.2
81.863	1.17575	146	12.1	19.2
81.873	1.17563	139	11.8	19.2
81.883	1.17552	127	11.3	19.2
81.893	1.17540	157	12.5	19.2
81.903	1.17528	147	12.1	19.2
81.913	1.17516	146	12.1	19.2
81.923	1.17504	134	11.6	19.2
81.933	1.17492	143	12.0	19.2
81.943	1.17480	143	12.0	19.2
81.954	1.17468	142	11.9	19.2
81.964	1.17456	139	11.8	19.2
81.974	1.17444	140	11.8	19.2
81.984	1.17432	114	10.7	19.2
81.994	1.17420	143	12.0	19.2
82.004	1.17408	129	11.4	19.2
82.014	1.17397	129	11.4	19.2
82.024	1.17385	128	11.3	19.2
82.034	1.17373	124	11.1	19.2
82.044	1.17361	121	11.0	19.2
82.055	1.17349	160	12.6	19.2
82.065	1.17337	140	11.8	19.2
82.075	1.17325	128	11.3	19.2
82.085	1.17313	153	12.4	19.2
82.095	1.17301	117	10.8	19.2
82.105	1.17290	124	11.1	19.2
82.115	1.17278	119	10.9	19.2
82.125	1.17266	133	11.5	19.2
82.135	1.17254	134	11.6	19.2
82.145	1.17242	145	12.0	19.2
82.156	1.17230	130	11.4	19.2
82.166	1.17218	137	11.7	19.2
82.176	1.17207	147	12.1	19.2
82.186	1.17195	121	11.0	19.2
82.196	1.17183	128	11.3	19.2
82.206	1.17171	153	12.4	19.2
82.216	1.17159	108	10.4	19.2
82.226	1.17147	147	12.1	19.2
82.236	1.17136	128	11.3	19.2
82.246	1.17124	139	11.8	19.2

82.257	1.17112	137	11.7	19.2
82.267	1.17100	129	11.4	19.2
82.277	1.17088	129	11.4	19.2
82.287	1.17076	141	11.9	19.2
82.297	1.17065	133	11.5	19.2
82.307	1.17053	171	13.1	19.2
82.317	1.17041	144	12.0	19.2
82.327	1.17029	150	12.2	19.2
82.337	1.17017	138	11.7	19.2
82.347	1.17006	154	12.4	19.2
82.358	1.16994	139	11.8	19.2
82.368	1.16982	146	12.1	19.2
82.378	1.16970	141	11.9	19.2
82.388	1.16959	123	11.1	19.2
82.398	1.16947	129	11.4	19.2
82.408	1.16935	132	11.5	19.2
82.418	1.16923	153	12.4	19.2
82.428	1.16911	128	11.3	19.2
82.438	1.16900	128	11.3	19.2
82.448	1.16888	136	11.7	19.2
82.459	1.16876	143	12.0	19.2
82.469	1.16864	127	11.3	19.2
82.479	1.16853	138	11.7	19.2
82.489	1.16841	135	11.6	19.2
82.499	1.16829	116	10.8	19.2
82.509	1.16817	156	12.5	19.2
82.519	1.16806	154	12.4	19.2
82.529	1.16794	144	12.0	19.2
82.539	1.16782	140	11.8	19.2
82.549	1.16771	148	12.2	19.2
82.559	1.16759	137	11.7	19.2
82.570	1.16747	146	12.1	19.2
82.580	1.16735	124	11.1	19.2
82.590	1.16724	132	11.5	19.2
82.600	1.16712	145	12.0	19.2
82.610	1.16700	135	11.6	19.2
82.620	1.16689	144	12.0	19.2
82.630	1.16677	150	12.2	19.2
82.640	1.16665	136	11.7	19.2
82.650	1.16653	128	11.3	19.2
82.660	1.16642	152	12.3	19.2
82.671	1.16630	137	11.7	19.2
82.681	1.16618	123	11.1	19.2
82.691	1.16607	141	11.9	19.2
82.701	1.16595	133	11.5	19.2
82.711	1.16583	138	11.7	19.2
82.721	1.16572	152	12.3	19.2
82.731	1.16560	149	12.2	19.2
82.741	1.16548	138	11.7	19.2
82.751	1.16537	132	11.5	19.2

82.761	1.16525	144	12.0	19.2
82.772	1.16513	139	11.8	19.2
82.782	1.16502	139	11.8	19.2
82.792	1.16490	138	11.7	19.2
82.802	1.16478	118	10.9	19.2
82.812	1.16467	162	12.7	19.2
82.822	1.16455	166	12.9	19.2
82.832	1.16444	121	11.0	19.2
82.842	1.16432	159	12.6	19.2
82.852	1.16420	129	11.4	19.2
82.862	1.16409	131	11.4	19.2
82.873	1.16397	129	11.4	19.2
82.883	1.16385	140	11.8	19.2
82.893	1.16374	149	12.2	19.2
82.903	1.16362	143	12.0	19.2
82.913	1.16351	152	12.3	19.2
82.923	1.16339	146	12.1	19.2
82.933	1.16327	127	11.3	19.2
82.943	1.16316	140	11.8	19.2
82.953	1.16304	124	11.1	19.2
82.963	1.16293	134	11.6	19.2
82.974	1.16281	140	11.8	19.2
82.984	1.16269	126	11.2	19.2
82.994	1.16258	150	12.2	19.2
83.004	1.16246	141	11.9	19.2
83.014	1.16235	143	12.0	19.2
83.024	1.16223	160	12.6	19.2
83.034	1.16211	139	11.8	19.2
83.044	1.16200	140	11.8	19.2
83.054	1.16188	137	11.7	19.2
83.064	1.16177	129	11.4	19.2
83.075	1.16165	139	11.8	19.2
83.085	1.16154	144	12.0	19.2
83.095	1.16142	151	12.3	19.2
83.105	1.16131	141	11.9	19.2
83.115	1.16119	140	11.8	19.2
83.125	1.16107	137	11.7	19.2
83.135	1.16096	125	11.2	19.2
83.145	1.16084	147	12.1	19.2
83.155	1.16073	138	11.7	19.2
83.165	1.16061	109	10.4	19.2
83.176	1.16050	132	11.5	19.2
83.186	1.16038	148	12.2	19.2
83.196	1.16027	134	11.6	19.2
83.206	1.16015	126	11.2	19.2
83.216	1.16004	136	11.7	19.2
83.226	1.15992	126	11.2	19.2
83.236	1.15981	119	10.9	19.2
83.246	1.15969	156	12.5	19.2
83.256	1.15958	161	12.7	19.2

83.266	1.15946	125	11.2	19.2
83.277	1.15935	124	11.1	19.2
83.287	1.15923	142	11.9	19.2
83.297	1.15912	137	11.7	19.2
83.307	1.15900	133	11.5	19.2
83.317	1.15889	137	11.7	19.2
83.327	1.15877	122	11.0	19.2
83.337	1.15866	139	11.8	19.2
83.347	1.15854	132	11.5	19.2
83.357	1.15843	139	11.8	19.2
83.367	1.15831	134	11.6	19.2
83.378	1.15820	122	11.0	19.2
83.388	1.15808	149	12.2	19.2
83.398	1.15797	150	12.2	19.2
83.408	1.15786	145	12.0	19.2
83.418	1.15774	147	12.1	19.2
83.428	1.15763	151	12.3	19.2
83.438	1.15751	133	11.5	19.2
83.448	1.15740	137	11.7	19.2
83.458	1.15728	156	12.5	19.2
83.468	1.15717	126	11.2	19.2
83.479	1.15706	145	12.0	19.2
83.489	1.15694	140	11.8	19.2
83.499	1.15683	130	11.4	19.2
83.509	1.15671	129	11.4	19.2
83.519	1.15660	134	11.6	19.2
83.529	1.15648	127	11.3	19.2
83.539	1.15637	146	12.1	19.2
83.549	1.15626	135	11.6	19.2
83.559	1.15614	137	11.7	19.2
83.569	1.15603	149	12.2	19.2
83.580	1.15591	143	12.0	19.2
83.590	1.15580	127	11.3	19.2
83.600	1.15569	140	11.8	19.2
83.610	1.15557	125	11.2	19.2
83.620	1.15546	140	11.8	19.2
83.630	1.15534	143	12.0	19.2
83.640	1.15523	140	11.8	19.2
83.650	1.15512	147	12.1	19.2
83.660	1.15500	148	12.2	19.2
83.670	1.15489	132	11.5	19.2
83.681	1.15478	139	11.8	19.2
83.691	1.15466	133	11.5	19.2
83.701	1.15455	139	11.8	19.2
83.711	1.15443	143	12.0	19.2
83.721	1.15432	138	11.7	19.2
83.731	1.15421	121	11.0	19.2
83.741	1.15409	130	11.4	19.2
83.751	1.15398	149	12.2	19.2
83.761	1.15387	130	11.4	19.2

83.771	1.15375	141	11.9	19.2
83.782	1.15364	151	12.3	19.2
83.792	1.15353	137	11.7	19.2
83.802	1.15341	154	12.4	19.2
83.812	1.15330	139	11.8	19.2
83.822	1.15319	124	11.1	19.2
83.832	1.15307	144	12.0	19.2
83.842	1.15296	151	12.3	19.2
83.852	1.15285	147	12.1	19.2
83.862	1.15273	158	12.6	19.2
83.872	1.15262	162	12.7	19.2
83.882	1.15251	140	11.8	19.2
83.893	1.15240	144	12.0	19.2
83.903	1.15228	119	10.9	19.2
83.913	1.15217	132	11.5	19.2
83.923	1.15206	149	12.2	19.2
83.933	1.15194	124	11.1	19.2
83.943	1.15183	137	11.7	19.2
83.953	1.15172	138	11.7	19.2
83.963	1.15161	131	11.4	19.2
83.973	1.15149	153	12.4	19.2
83.983	1.15138	154	12.4	19.2
83.994	1.15127	138	11.7	19.2
84.004	1.15115	149	12.2	19.2
84.014	1.15104	130	11.4	19.2
84.024	1.15093	149	12.2	19.2
84.034	1.15082	133	11.5	19.2
84.044	1.15070	151	12.3	19.2
84.054	1.15059	124	11.1	19.2
84.064	1.15048	131	11.4	19.2
84.074	1.15037	120	11.0	19.2
84.084	1.15025	154	12.4	19.2
84.095	1.15014	146	12.1	19.2
84.105	1.15003	164	12.8	19.2
84.115	1.14992	150	12.2	19.2
84.125	1.14980	120	11.0	19.2
84.135	1.14969	153	12.4	19.2
84.145	1.14958	125	11.2	19.2
84.155	1.14947	136	11.7	19.2
84.165	1.14936	140	11.8	19.2
84.175	1.14924	145	12.0	19.2
84.185	1.14913	129	11.4	19.2
84.196	1.14902	121	11.0	19.2
84.206	1.14891	125	11.2	19.2
84.216	1.14879	134	11.6	19.2
84.226	1.14868	140	11.8	19.2
84.236	1.14857	137	11.7	19.2
84.246	1.14846	130	11.4	19.2
84.256	1.14835	130	11.4	19.2
84.266	1.14824	139	11.8	19.2

84.276	1.14812	124	11.1	19.2
84.286	1.14801	137	11.7	19.2
84.297	1.14790	148	12.2	19.2
84.307	1.14779	136	11.7	19.2
84.317	1.14768	146	12.1	19.2
84.327	1.14756	138	11.7	19.2
84.337	1.14745	136	11.7	19.2
84.347	1.14734	129	11.4	19.2
84.357	1.14723	116	10.8	19.2
84.367	1.14712	135	11.6	19.2
84.377	1.14701	135	11.6	19.2
84.387	1.14689	126	11.2	19.2
84.398	1.14678	141	11.9	19.2
84.408	1.14667	136	11.7	19.2
84.418	1.14656	131	11.4	19.2
84.428	1.14645	148	12.2	19.2
84.438	1.14634	145	12.0	19.2
84.448	1.14623	155	12.4	19.2
84.458	1.14612	142	11.9	19.2
84.468	1.14600	139	11.8	19.2
84.478	1.14589	147	12.1	19.2
84.488	1.14578	128	11.3	19.2
84.499	1.14567	144	12.0	19.2
84.509	1.14556	123	11.1	19.2
84.519	1.14545	146	12.1	19.2
84.529	1.14534	123	11.1	19.2
84.539	1.14523	149	12.2	19.2
84.549	1.14511	115	10.7	19.2
84.559	1.14500	127	11.3	19.2
84.569	1.14489	137	11.7	19.2
84.579	1.14478	144	12.0	19.2
84.589	1.14467	143	12.0	19.2
84.600	1.14456	123	11.1	19.2
84.610	1.14445	154	12.4	19.2
84.620	1.14434	140	11.8	19.2
84.630	1.14423	150	12.2	19.2
84.640	1.14412	128	11.3	19.2
84.650	1.14401	135	11.6	19.2
84.660	1.14390	124	11.1	19.2
84.670	1.14378	144	12.0	19.2
84.680	1.14367	131	11.4	19.2
84.690	1.14356	151	12.3	19.2
84.701	1.14345	150	12.2	19.2
84.711	1.14334	137	11.7	19.2
84.721	1.14323	121	11.0	19.2
84.731	1.14312	121	11.0	19.2
84.741	1.14301	137	11.7	19.2
84.751	1.14290	147	12.1	19.2
84.761	1.14279	147	12.1	19.2
84.771	1.14268	129	11.4	19.2

84.781	1.14257	165	12.8	19.2
84.791	1.14246	145	12.0	19.2
84.802	1.14235	160	12.6	19.2
84.812	1.14224	163	12.8	19.2
84.822	1.14213	132	11.5	19.2
84.832	1.14202	129	11.4	19.2
84.842	1.14191	149	12.2	19.2
84.852	1.14180	136	11.7	19.2
84.862	1.14169	144	12.0	19.2
84.872	1.14158	148	12.2	19.2
84.882	1.14147	141	11.9	19.2
84.892	1.14136	149	12.2	19.2
84.903	1.14125	141	11.9	19.2
84.913	1.14114	123	11.1	19.2
84.923	1.14103	152	12.3	19.2
84.933	1.14092	161	12.7	19.2
84.943	1.14081	142	11.9	19.2
84.953	1.14070	143	12.0	19.2
84.963	1.14059	148	12.2	19.2
84.973	1.14048	139	11.8	19.2
84.983	1.14037	145	12.0	19.2
84.993	1.14026	151	12.3	19.2
85.004	1.14015	135	11.6	19.2
85.014	1.14004	142	11.9	19.2
85.024	1.13993	135	11.6	19.2
85.034	1.13982	124	11.1	19.2
85.044	1.13971	141	11.9	19.2
85.054	1.13960	154	12.4	19.2
85.064	1.13949	145	12.0	19.2
85.074	1.13938	133	11.5	19.2
85.084	1.13927	138	11.7	19.2
85.094	1.13916	149	12.2	19.2
85.105	1.13906	152	12.3	19.2
85.115	1.13895	148	12.2	19.2
85.125	1.13884	114	10.7	19.2
85.135	1.13873	144	12.0	19.2
85.145	1.13862	161	12.7	19.2
85.155	1.13851	126	11.2	19.2
85.165	1.13840	145	12.0	19.2
85.175	1.13829	149	12.2	19.2
85.185	1.13818	142	11.9	19.2
85.195	1.13807	108	10.4	19.2
85.205	1.13796	148	12.2	19.2
85.216	1.13785	131	11.4	19.2
85.226	1.13774	139	11.8	19.2
85.236	1.13764	119	10.9	19.2
85.246	1.13753	142	11.9	19.2
85.256	1.13742	135	11.6	19.2
85.266	1.13731	140	11.8	19.2
85.276	1.13720	151	12.3	19.2

85.286	1.13709	111	10.5	19.2
85.296	1.13698	158	12.6	19.2
85.306	1.13687	140	11.8	19.2
85.317	1.13677	135	11.6	19.2
85.327	1.13666	148	12.2	19.2
85.337	1.13655	120	11.0	19.2
85.347	1.13644	158	12.6	19.2
85.357	1.13633	141	11.9	19.2
85.367	1.13622	130	11.4	19.2
85.377	1.13611	150	12.2	19.2
85.387	1.13600	132	11.5	19.2
85.397	1.13590	152	12.3	19.2
85.407	1.13579	132	11.5	19.2
85.418	1.13568	132	11.5	19.2
85.428	1.13557	133	11.5	19.2
85.438	1.13546	143	12.0	19.2
85.448	1.13535	146	12.1	19.2
85.458	1.13525	124	11.1	19.2
85.468	1.13514	141	11.9	19.2
85.478	1.13503	135	11.6	19.2
85.488	1.13492	144	12.0	19.2
85.498	1.13481	139	11.8	19.2
85.508	1.13470	128	11.3	19.2
85.519	1.13460	162	12.7	19.2
85.529	1.13449	128	11.3	19.2
85.539	1.13438	146	12.1	19.2
85.549	1.13427	147	12.1	19.2
85.559	1.13416	139	11.8	19.2
85.569	1.13406	124	11.1	19.2
85.579	1.13395	128	11.3	19.2
85.589	1.13384	129	11.4	19.2
85.599	1.13373	140	11.8	19.2
85.609	1.13362	132	11.5	19.2
85.620	1.13352	145	12.0	19.2
85.630	1.13341	142	11.9	19.2
85.640	1.13330	142	11.9	19.2
85.650	1.13319	130	11.4	19.2
85.660	1.13309	136	11.7	19.2
85.670	1.13298	156	12.5	19.2
85.680	1.13287	143	12.0	19.2
85.690	1.13276	151	12.3	19.2
85.700	1.13265	149	12.2	19.2
85.710	1.13255	118	10.9	19.2
85.721	1.13244	147	12.1	19.2
85.731	1.13233	130	11.4	19.2
85.741	1.13222	164	12.8	19.2
85.751	1.13212	133	11.5	19.2
85.761	1.13201	135	11.6	19.2
85.771	1.13190	116	10.8	19.2
85.781	1.13179	120	11.0	19.2

85.791	1.13169	144	12.0	19.2
85.801	1.13158	127	11.3	19.2
85.811	1.13147	151	12.3	19.2
85.822	1.13137	132	11.5	19.2
85.832	1.13126	129	11.4	19.2
85.842	1.13115	146	12.1	19.2
85.852	1.13104	145	12.0	19.2
85.862	1.13094	137	11.7	19.2
85.872	1.13083	171	13.1	19.2
85.882	1.13072	144	12.0	19.2
85.892	1.13062	147	12.1	19.2
85.902	1.13051	149	12.2	19.2
85.912	1.13040	147	12.1	19.2
85.923	1.13029	130	11.4	19.2
85.933	1.13019	138	11.7	19.2
85.943	1.13008	137	11.7	19.2
85.953	1.12997	131	11.4	19.2
85.963	1.12987	154	12.4	19.2
85.973	1.12976	140	11.8	19.2
85.983	1.12965	143	12.0	19.2
85.993	1.12955	137	11.7	19.2
86.003	1.12944	133	11.5	19.2
86.013	1.12933	135	11.6	19.2
86.024	1.12923	137	11.7	19.2
86.034	1.12912	140	11.8	19.2
86.044	1.12901	150	12.2	19.2
86.054	1.12891	128	11.3	19.2
86.064	1.12880	133	11.5	19.2
86.074	1.12869	150	12.2	19.2
86.084	1.12859	141	11.9	19.2
86.094	1.12848	151	12.3	19.2
86.104	1.12837	119	10.9	19.2
86.114	1.12827	122	11.0	19.2
86.125	1.12816	138	11.7	19.2
86.135	1.12805	137	11.7	19.2
86.145	1.12795	146	12.1	19.2
86.155	1.12784	135	11.6	19.2
86.165	1.12774	135	11.6	19.2
86.175	1.12763	141	11.9	19.2
86.185	1.12752	145	12.0	19.2
86.195	1.12742	137	11.7	19.2
86.205	1.12731	138	11.7	19.2
86.215	1.12720	138	11.7	19.2
86.226	1.12710	146	12.1	19.2
86.236	1.12699	132	11.5	19.2
86.246	1.12689	144	12.0	19.2
86.256	1.12678	134	11.6	19.2
86.266	1.12667	132	11.5	19.2
86.276	1.12657	139	11.8	19.2
86.286	1.12646	127	11.3	19.2

86.296	1.12636	136	11.7	19.2
86.306	1.12625	134	11.6	19.2
86.316	1.12614	134	11.6	19.2
86.327	1.12604	138	11.7	19.2
86.337	1.12593	130	11.4	19.2
86.347	1.12583	138	11.7	19.2
86.357	1.12572	143	12.0	19.2
86.367	1.12562	126	11.2	19.2
86.377	1.12551	155	12.4	19.2
86.387	1.12540	129	11.4	19.2
86.397	1.12530	135	11.6	19.2
86.407	1.12519	149	12.2	19.2
86.417	1.12509	115	10.7	19.2
86.428	1.12498	122	11.0	19.2
86.438	1.12488	141	11.9	19.2
86.448	1.12477	115	10.7	19.2
86.458	1.12467	138	11.7	19.2
86.468	1.12456	137	11.7	19.2
86.478	1.12445	148	12.2	19.2
86.488	1.12435	138	11.7	19.2
86.498	1.12424	156	12.5	19.2
86.508	1.12414	136	11.7	19.2
86.518	1.12403	146	12.1	19.2
86.528	1.12393	134	11.6	19.2
86.539	1.12382	130	11.4	19.2
86.549	1.12372	157	12.5	19.2
86.559	1.12361	134	11.6	19.2
86.569	1.12351	142	11.9	19.2
86.579	1.12340	148	12.2	19.2
86.589	1.12330	144	12.0	19.2
86.599	1.12319	140	11.8	19.2
86.609	1.12309	141	11.9	19.2
86.619	1.12298	141	11.9	19.2
86.629	1.12288	136	11.7	19.2
86.640	1.12277	129	11.4	19.2
86.650	1.12267	148	12.2	19.2
86.660	1.12256	131	11.4	19.2
86.670	1.12246	130	11.4	19.2
86.680	1.12235	135	11.6	19.2
86.690	1.12225	161	12.7	19.2
86.700	1.12214	154	12.4	19.2
86.710	1.12204	154	12.4	19.2
86.720	1.12193	134	11.6	19.2
86.730	1.12183	142	11.9	19.2
86.741	1.12172	139	11.8	19.2
86.751	1.12162	137	11.7	19.2
86.761	1.12151	142	11.9	19.2
86.771	1.12141	131	11.4	19.2
86.781	1.12131	139	11.8	19.2
86.791	1.12120	148	12.2	19.2

86.801	1.12110	151	12.3	19.2
86.811	1.12099	133	11.5	19.2
86.821	1.12089	155	12.4	19.2
86.831	1.12078	121	11.0	19.2
86.842	1.12068	127	11.3	19.2
86.852	1.12057	148	12.2	19.2
86.862	1.12047	144	12.0	19.2
86.872	1.12037	141	11.9	19.2
86.882	1.12026	129	11.4	19.2
86.892	1.12016	144	12.0	19.2
86.902	1.12005	144	12.0	19.2
86.912	1.11995	134	11.6	19.2
86.922	1.11985	147	12.1	19.2
86.932	1.11974	136	11.7	19.2
86.943	1.11964	134	11.6	19.2
86.953	1.11953	149	12.2	19.2
86.963	1.11943	141	11.9	19.2
86.973	1.11932	143	12.0	19.2
86.983	1.11922	129	11.4	19.2
86.993	1.11912	139	11.8	19.2
87.003	1.11901	126	11.2	19.2
87.013	1.11891	140	11.8	19.2
87.023	1.11881	135	11.6	19.2
87.033	1.11870	140	11.8	19.2
87.044	1.11860	123	11.1	19.2
87.054	1.11849	137	11.7	19.2
87.064	1.11839	132	11.5	19.2
87.074	1.11829	155	12.4	19.2
87.084	1.11818	139	11.8	19.2
87.094	1.11808	135	11.6	19.2
87.104	1.11798	145	12.0	19.2
87.114	1.11787	139	11.8	19.2
87.124	1.11777	127	11.3	19.2
87.134	1.11766	134	11.6	19.2
87.145	1.11756	145	12.0	19.2
87.155	1.11746	138	11.7	19.2
87.165	1.11735	139	11.8	19.2
87.175	1.11725	125	11.2	19.2
87.185	1.11715	131	11.4	19.2
87.195	1.11704	123	11.1	19.2
87.205	1.11694	122	11.0	19.2
87.215	1.11684	134	11.6	19.2
87.225	1.11673	143	12.0	19.2
87.235	1.11663	125	11.2	19.2
87.246	1.11653	121	11.0	19.2
87.256	1.11642	135	11.6	19.2
87.266	1.11632	140	11.8	19.2
87.276	1.11622	142	11.9	19.2
87.286	1.11611	163	12.8	19.2
87.296	1.11601	151	12.3	19.2

87.306	1.11591	132	11.5	19.2
87.316	1.11580	124	11.1	19.2
87.326	1.11570	125	11.2	19.2
87.336	1.11560	111	10.5	19.2
87.347	1.11550	138	11.7	19.2
87.357	1.11539	135	11.6	19.2
87.367	1.11529	165	12.8	19.2
87.377	1.11519	144	12.0	19.2
87.387	1.11508	135	11.6	19.2
87.397	1.11498	144	12.0	19.2
87.407	1.11488	129	11.4	19.2
87.417	1.11478	142	11.9	19.2
87.427	1.11467	124	11.1	19.2
87.437	1.11457	134	11.6	19.2
87.448	1.11447	133	11.5	19.2
87.458	1.11436	150	12.2	19.2
87.468	1.11426	148	12.2	19.2
87.478	1.11416	142	11.9	19.2
87.488	1.11406	170	13.0	19.2
87.498	1.11395	138	11.7	19.2
87.508	1.11385	134	11.6	19.2
87.518	1.11375	136	11.7	19.2
87.528	1.11365	136	11.7	19.2
87.538	1.11354	138	11.7	19.2
87.549	1.11344	138	11.7	19.2
87.559	1.11334	135	11.6	19.2
87.569	1.11324	134	11.6	19.2
87.579	1.11313	138	11.7	19.2
87.589	1.11303	150	12.2	19.2
87.599	1.11293	141	11.9	19.2
87.609	1.11283	135	11.6	19.2
87.619	1.11273	145	12.0	19.2
87.629	1.11262	124	11.1	19.2
87.639	1.11252	128	11.3	19.2
87.650	1.11242	145	12.0	19.2
87.660	1.11232	125	11.2	19.2
87.670	1.11221	118	10.9	19.2
87.680	1.11211	144	12.0	19.2
87.690	1.11201	139	11.8	19.2
87.700	1.11191	135	11.6	19.2
87.710	1.11181	150	12.2	19.2
87.720	1.11170	140	11.8	19.2
87.730	1.11160	142	11.9	19.2
87.740	1.11150	127	11.3	19.2
87.751	1.11140	149	12.2	19.2
87.761	1.11130	150	12.2	19.2
87.771	1.11120	143	12.0	19.2
87.781	1.11109	117	10.8	19.2
87.791	1.11099	114	10.7	19.2
87.801	1.11089	127	11.3	19.2

87.811	1.11079	129	11.4	19.2
87.821	1.11069	152	12.3	19.2
87.831	1.11058	147	12.1	19.2
87.841	1.11048	115	10.7	19.2
87.851	1.11038	110	10.5	19.2
87.862	1.11028	151	12.3	19.2
87.872	1.11018	161	12.7	19.2
87.882	1.11008	141	11.9	19.2
87.892	1.10998	140	11.8	19.2
87.902	1.10987	150	12.2	19.2
87.912	1.10977	144	12.0	19.2
87.922	1.10967	134	11.6	19.2
87.932	1.10957	151	12.3	19.2
87.942	1.10947	144	12.0	19.2
87.952	1.10937	145	12.0	19.2
87.963	1.10927	134	11.6	19.2
87.973	1.10916	117	10.8	19.2
87.983	1.10906	150	12.2	19.2
87.993	1.10896	156	12.5	19.2
88.003	1.10886	156	12.5	19.2
88.013	1.10876	130	11.4	19.2
88.023	1.10866	134	11.6	19.2
88.033	1.10856	142	11.9	19.2
88.043	1.10846	134	11.6	19.2
88.053	1.10835	119	10.9	19.2
88.064	1.10825	122	11.0	19.2
88.074	1.10815	138	11.7	19.2
88.084	1.10805	137	11.7	19.2
88.094	1.10795	146	12.1	19.2
88.104	1.10785	132	11.5	19.2
88.114	1.10775	126	11.2	19.2
88.124	1.10765	145	12.0	19.2
88.134	1.10755	150	12.2	19.2
88.144	1.10745	135	11.6	19.2
88.154	1.10735	133	11.5	19.2
88.165	1.10724	152	12.3	19.2
88.175	1.10714	110	10.5	19.2
88.185	1.10704	133	11.5	19.2
88.195	1.10694	134	11.6	19.2
88.205	1.10684	134	11.6	19.2
88.215	1.10674	118	10.9	19.2
88.225	1.10664	147	12.1	19.2
88.235	1.10654	156	12.5	19.2
88.245	1.10644	129	11.4	19.2
88.255	1.10634	129	11.4	19.2
88.266	1.10624	139	11.8	19.2
88.276	1.10614	138	11.7	19.2
88.286	1.10604	128	11.3	19.2
88.296	1.10594	152	12.3	19.2
88.306	1.10584	145	12.0	19.2

88.316	1.10574	139	11.8	19.2
88.326	1.10564	130	11.4	19.2
88.336	1.10554	149	12.2	19.2
88.346	1.10544	128	11.3	19.2
88.356	1.10534	121	11.0	19.2
88.367	1.10523	152	12.3	19.2
88.377	1.10513	152	12.3	19.2
88.387	1.10503	127	11.3	19.2
88.397	1.10493	123	11.1	19.2
88.407	1.10483	134	11.6	19.2
88.417	1.10473	131	11.4	19.2
88.427	1.10463	152	12.3	19.2
88.437	1.10453	130	11.4	19.2
88.447	1.10443	129	11.4	19.2
88.457	1.10433	132	11.5	19.2
88.468	1.10423	138	11.7	19.2
88.478	1.10413	122	11.0	19.2
88.488	1.10403	135	11.6	19.2
88.498	1.10393	126	11.2	19.2
88.508	1.10383	125	11.2	19.2
88.518	1.10373	113	10.6	19.2
88.528	1.10363	114	10.7	19.2
88.538	1.10354	131	11.4	19.2
88.548	1.10344	129	11.4	19.2
88.558	1.10334	116	10.8	19.2
88.569	1.10324	139	11.8	19.2
88.579	1.10314	124	11.1	19.2
88.589	1.10304	138	11.7	19.2
88.599	1.10294	143	12.0	19.2
88.609	1.10284	123	11.1	19.2
88.619	1.10274	146	12.1	19.2
88.629	1.10264	141	11.9	19.2
88.639	1.10254	102	10.1	19.2
88.649	1.10244	139	11.8	19.2
88.659	1.10234	153	12.4	19.2
88.670	1.10224	134	11.6	19.2
88.680	1.10214	142	11.9	19.2
88.690	1.10204	120	11.0	19.2
88.700	1.10194	140	11.8	19.2
88.710	1.10184	137	11.7	19.2
88.720	1.10174	105	10.2	19.2
88.730	1.10164	131	11.4	19.2
88.740	1.10154	153	12.4	19.2
88.750	1.10145	119	10.9	19.2
88.760	1.10135	131	11.4	19.2
88.771	1.10125	135	11.6	19.2
88.781	1.10115	136	11.7	19.2
88.791	1.10105	149	12.2	19.2
88.801	1.10095	130	11.4	19.2
88.811	1.10085	154	12.4	19.2

88.821	1.10075	140	11.8	19.2
88.831	1.10065	140	11.8	19.2
88.841	1.10055	135	11.6	19.2
88.851	1.10045	120	11.0	19.2
88.861	1.10036	130	11.4	19.2
88.872	1.10026	145	12.0	19.2
88.882	1.10016	123	11.1	19.2
88.892	1.10006	150	12.2	19.2
88.902	1.09996	134	11.6	19.2
88.912	1.09986	136	11.7	19.2
88.922	1.09976	138	11.7	19.2
88.932	1.09966	131	11.4	19.2
88.942	1.09957	158	12.6	19.2
88.952	1.09947	132	11.5	19.2
88.962	1.09937	157	12.5	19.2
88.973	1.09927	154	12.4	19.2
88.983	1.09917	151	12.3	19.2
88.993	1.09907	147	12.1	19.2
89.003	1.09897	130	11.4	19.2
89.013	1.09888	132	11.5	19.2
89.023	1.09878	137	11.7	19.2
89.033	1.09868	137	11.7	19.2
89.043	1.09858	142	11.9	19.2
89.053	1.09848	146	12.1	19.2
89.063	1.09838	133	11.5	19.2
89.074	1.09828	119	10.9	19.2
89.084	1.09819	118	10.9	19.2
89.094	1.09809	125	11.2	19.2
89.104	1.09799	141	11.9	19.2
89.114	1.09789	136	11.7	19.2
89.124	1.09779	133	11.5	19.2
89.134	1.09769	162	12.7	19.2
89.144	1.09760	136	11.7	19.2
89.154	1.09750	127	11.3	19.2
89.164	1.09740	134	11.6	19.2
89.174	1.09730	140	11.8	19.2
89.185	1.09720	126	11.2	19.2
89.195	1.09711	144	12.0	19.2
89.205	1.09701	145	12.0	19.2
89.215	1.09691	146	12.1	19.2
89.225	1.09681	124	11.1	19.2
89.235	1.09671	141	11.9	19.2
89.245	1.09662	137	11.7	19.2
89.255	1.09652	135	11.6	19.2
89.265	1.09642	134	11.6	19.2
89.275	1.09632	133	11.5	19.2
89.286	1.09622	129	11.4	19.2
89.296	1.09613	134	11.6	19.2
89.306	1.09603	140	11.8	19.2
89.316	1.09593	140	11.8	19.2

89.326	1.09583	117	10.8	19.2
89.336	1.09574	126	11.2	19.2
89.346	1.09564	131	11.4	19.2
89.356	1.09554	141	11.9	19.2
89.366	1.09544	138	11.7	19.2
89.376	1.09534	132	11.5	19.2
89.387	1.09525	135	11.6	19.2
89.397	1.09515	140	11.8	19.2
89.407	1.09505	115	10.7	19.2
89.417	1.09495	144	12.0	19.2
89.427	1.09486	120	11.0	19.2
89.437	1.09476	122	11.0	19.2
89.447	1.09466	129	11.4	19.2
89.457	1.09456	142	11.9	19.2
89.467	1.09447	147	12.1	19.2
89.477	1.09437	142	11.9	19.2
89.488	1.09427	143	12.0	19.2
89.498	1.09418	147	12.1	19.2
89.508	1.09408	137	11.7	19.2
89.518	1.09398	137	11.7	19.2
89.528	1.09388	143	12.0	19.2
89.538	1.09379	144	12.0	19.2
89.548	1.09369	131	11.4	19.2
89.558	1.09359	135	11.6	19.2
89.568	1.09350	107	10.3	19.2
89.578	1.09340	143	12.0	19.2
89.589	1.09330	149	12.2	19.2
89.599	1.09320	116	10.8	19.2
89.609	1.09311	134	11.6	19.2
89.619	1.09301	143	12.0	19.2
89.629	1.09291	140	11.8	19.2
89.639	1.09282	143	12.0	19.2
89.649	1.09272	134	11.6	19.2
89.659	1.09262	135	11.6	19.2
89.669	1.09253	143	12.0	19.2
89.679	1.09243	151	12.3	19.2
89.690	1.09233	132	11.5	19.2
89.700	1.09224	123	11.1	19.2
89.710	1.09214	127	11.3	19.2
89.720	1.09204	130	11.4	19.2
89.730	1.09194	110	10.5	19.2
89.740	1.09185	149	12.2	19.2
89.750	1.09175	142	11.9	19.2
89.760	1.09166	148	12.2	19.2
89.770	1.09156	132	11.5	19.2
89.780	1.09146	146	12.1	19.2
89.791	1.09137	139	11.8	19.2
89.801	1.09127	138	11.7	19.2
89.811	1.09117	132	11.5	19.2
89.821	1.09108	149	12.2	19.2

89.831	1.09098	132	11.5	19.2
89.841	1.09088	127	11.3	19.2
89.851	1.09079	148	12.2	19.2
89.861	1.09069	118	10.9	19.2
89.871	1.09059	138	11.7	19.2
89.881	1.09050	131	11.4	19.2
89.892	1.09040	146	12.1	19.2
89.902	1.09030	116	10.8	19.2
89.912	1.09021	154	12.4	19.2
89.922	1.09011	118	10.9	19.2
89.932	1.09002	131	11.4	19.2
89.942	1.08992	142	11.9	19.2
89.952	1.08982	128	11.3	19.2
89.962	1.08973	129	11.4	19.2
89.972	1.08963	127	11.3	19.2
89.982	1.08954	135	11.6	19.2
89.993	1.08944	130	11.4	19.2
90.003	1.08934	135	11.6	19.2
90.013	1.08925	135	11.6	19.2
90.023	1.08915	141	11.9	19.2
90.033	1.08906	129	11.4	19.2
90.043	1.08896	132	11.5	19.2
90.053	1.08886	137	11.7	19.2
90.063	1.08877	140	11.8	19.2
90.073	1.08867	124	11.1	19.2
90.083	1.08858	132	11.5	19.2
90.094	1.08848	145	12.0	19.2
90.104	1.08838	105	10.2	19.2
90.114	1.08829	133	11.5	19.2
90.124	1.08819	121	11.0	19.2
90.134	1.08810	130	11.4	19.2
90.144	1.08800	150	12.2	19.2
90.154	1.08791	135	11.6	19.2
90.164	1.08781	113	10.6	19.2
90.174	1.08772	129	11.4	19.2
90.184	1.08762	140	11.8	19.2
90.195	1.08752	130	11.4	19.2
90.205	1.08743	140	11.8	19.2
90.215	1.08733	136	11.7	19.2
90.225	1.08724	152	12.3	19.2
90.235	1.08714	123	11.1	19.2
90.245	1.08705	163	12.8	19.2
90.255	1.08695	112	10.6	19.2
90.265	1.08686	142	11.9	19.2
90.275	1.08676	142	11.9	19.2
90.285	1.08667	134	11.6	19.2
90.296	1.08657	147	12.1	19.2
90.306	1.08647	142	11.9	19.2
90.316	1.08638	153	12.4	19.2
90.326	1.08628	124	11.1	19.2

90.336	1.08619	142	11.9	19.2
90.346	1.08609	146	12.1	19.2
90.356	1.08600	123	11.1	19.2
90.366	1.08590	142	11.9	19.2
90.376	1.08581	145	12.0	19.2
90.386	1.08571	148	12.2	19.2
90.397	1.08562	142	11.9	19.2
90.407	1.08552	125	11.2	19.2
90.417	1.08543	142	11.9	19.2
90.427	1.08533	148	12.2	19.2
90.437	1.08524	135	11.6	19.2
90.447	1.08514	132	11.5	19.2
90.457	1.08505	124	11.1	19.2
90.467	1.08495	125	11.2	19.2
90.477	1.08486	100	10.0	19.2
90.487	1.08476	142	11.9	19.2
90.497	1.08467	137	11.7	19.2
90.508	1.08458	118	10.9	19.2
90.518	1.08448	124	11.1	19.2
90.528	1.08439	124	11.1	19.2
90.538	1.08429	131	11.4	19.2
90.548	1.08420	153	12.4	19.2
90.558	1.08410	112	10.6	19.2
90.568	1.08401	125	11.2	19.2
90.578	1.08391	148	12.2	19.2
90.588	1.08382	123	11.1	19.2
90.598	1.08372	121	11.0	19.2
90.609	1.08363	135	11.6	19.2
90.619	1.08353	110	10.5	19.2
90.629	1.08344	138	11.7	19.2
90.639	1.08335	126	11.2	19.2
90.649	1.08325	140	11.8	19.2
90.659	1.08316	121	11.0	19.2
90.669	1.08306	132	11.5	19.2
90.679	1.08297	154	12.4	19.2
90.689	1.08287	133	11.5	19.2
90.699	1.08278	145	12.0	19.2
90.710	1.08269	132	11.5	19.2
90.720	1.08259	145	12.0	19.2
90.730	1.08250	127	11.3	19.2
90.740	1.08240	115	10.7	19.2
90.750	1.08231	131	11.4	19.2
90.760	1.08221	123	11.1	19.2
90.770	1.08212	142	11.9	19.2
90.780	1.08203	129	11.4	19.2
90.790	1.08193	132	11.5	19.2
90.800	1.08184	148	12.2	19.2
90.811	1.08174	131	11.4	19.2
90.821	1.08165	133	11.5	19.2
90.831	1.08156	146	12.1	19.2

90.841	1.08146	146	12.1	19.2
90.851	1.08137	126	11.2	19.2
90.861	1.08127	141	11.9	19.2
90.871	1.08118	141	11.9	19.2
90.881	1.08109	120	11.0	19.2
90.891	1.08099	136	11.7	19.2
90.901	1.08090	138	11.7	19.2
90.912	1.08081	140	11.8	19.2
90.922	1.08071	146	12.1	19.2
90.932	1.08062	122	11.0	19.2
90.942	1.08052	134	11.6	19.2
90.952	1.08043	134	11.6	19.2
90.962	1.08034	117	10.8	19.2
90.972	1.08024	124	11.1	19.2
90.982	1.08015	128	11.3	19.2
90.992	1.08006	122	11.0	19.2
91.002	1.07996	136	11.7	19.2
91.013	1.07987	126	11.2	19.2
91.023	1.07978	142	11.9	19.2
91.033	1.07968	132	11.5	19.2
91.043	1.07959	126	11.2	19.2
91.053	1.07950	115	10.7	19.2
91.063	1.07940	133	11.5	19.2
91.073	1.07931	130	11.4	19.2
91.083	1.07922	147	12.1	19.2
91.093	1.07912	117	10.8	19.2
91.103	1.07903	130	11.4	19.2
91.114	1.07894	151	12.3	19.2
91.124	1.07884	129	11.4	19.2
91.134	1.07875	126	11.2	19.2
91.144	1.07866	135	11.6	19.2
91.154	1.07856	139	11.8	19.2
91.164	1.07847	137	11.7	19.2
91.174	1.07838	146	12.1	19.2
91.184	1.07828	137	11.7	19.2
91.194	1.07819	132	11.5	19.2
91.204	1.07810	107	10.3	19.2
91.215	1.07800	116	10.8	19.2
91.225	1.07791	136	11.7	19.2
91.235	1.07782	142	11.9	19.2
91.245	1.07772	124	11.1	19.2
91.255	1.07763	136	11.7	19.2
91.265	1.07754	129	11.4	19.2
91.275	1.07745	134	11.6	19.2
91.285	1.07735	146	12.1	19.2
91.295	1.07726	138	11.7	19.2
91.305	1.07717	131	11.4	19.2
91.316	1.07707	119	10.9	19.2
91.326	1.07698	122	11.0	19.2
91.336	1.07689	123	11.1	19.2

91.346	1.07680	131	11.4	19.2
91.356	1.07670	139	11.8	19.2
91.366	1.07661	135	11.6	19.2
91.376	1.07652	132	11.5	19.2
91.386	1.07643	129	11.4	19.2
91.396	1.07633	109	10.4	19.2
91.406	1.07624	137	11.7	19.2
91.417	1.07615	120	11.0	19.2
91.427	1.07606	141	11.9	19.2
91.437	1.07596	125	11.2	19.2
91.447	1.07587	128	11.3	19.2
91.457	1.07578	117	10.8	19.2
91.467	1.07569	124	11.1	19.2
91.477	1.07559	137	11.7	19.2
91.487	1.07550	134	11.6	19.2
91.497	1.07541	128	11.3	19.2
91.507	1.07532	124	11.1	19.2
91.518	1.07522	116	10.8	19.2
91.528	1.07513	121	11.0	19.2
91.538	1.07504	136	11.7	19.2
91.548	1.07495	128	11.3	19.2
91.558	1.07486	134	11.6	19.2
91.568	1.07476	137	11.7	19.2
91.578	1.07467	119	10.9	19.2
91.588	1.07458	148	12.2	19.2
91.598	1.07449	126	11.2	19.2
91.608	1.07439	142	11.9	19.2
91.619	1.07430	94.0	9.70	19.2
91.629	1.07421	123	11.1	19.2
91.639	1.07412	123	11.1	19.2
91.649	1.07403	105	10.2	19.2
91.659	1.07393	139	11.8	19.2
91.669	1.07384	130	11.4	19.2
91.679	1.07375	143	12.0	19.2
91.689	1.07366	146	12.1	19.2
91.699	1.07357	124	11.1	19.2
91.709	1.07348	122	11.0	19.2
91.719	1.07338	130	11.4	19.2
91.730	1.07329	139	11.8	19.2
91.740	1.07320	140	11.8	19.2
91.750	1.07311	136	11.7	19.2
91.760	1.07302	134	11.6	19.2
91.770	1.07292	141	11.9	19.2
91.780	1.07283	117	10.8	19.2
91.790	1.07274	135	11.6	19.2
91.800	1.07265	110	10.5	19.2
91.810	1.07256	134	11.6	19.2
91.820	1.07247	137	11.7	19.2
91.831	1.07238	128	11.3	19.2
91.841	1.07228	127	11.3	19.2

91.851	1.07219	120	11.0	19.2
91.861	1.07210	116	10.8	19.2
91.871	1.07201	142	11.9	19.2
91.881	1.07192	127	11.3	19.2
91.891	1.07183	126	11.2	19.2
91.901	1.07173	140	11.8	19.2
91.911	1.07164	124	11.1	19.2
91.921	1.07155	124	11.1	19.2
91.932	1.07146	117	10.8	19.2
91.942	1.07137	139	11.8	19.2
91.952	1.07128	134	11.6	19.2
91.962	1.07119	126	11.2	19.2
91.972	1.07110	117	10.8	19.2
91.982	1.07100	122	11.0	19.2
91.992	1.07091	133	11.5	19.2
92.002	1.07082	139	11.8	19.2
92.012	1.07073	115	10.7	19.2
92.022	1.07064	139	11.8	19.2
92.033	1.07055	134	11.6	19.2
92.043	1.07046	140	11.8	19.2
92.053	1.07037	120	11.0	19.2
92.063	1.07028	124	11.1	19.2
92.073	1.07019	126	11.2	19.2
92.083	1.07009	116	10.8	19.2
92.093	1.07000	143	12.0	19.2
92.103	1.06991	153	12.4	19.2
92.113	1.06982	149	12.2	19.2
92.123	1.06973	129	11.4	19.2
92.134	1.06964	151	12.3	19.2
92.144	1.06955	106	10.3	19.2
92.154	1.06946	125	11.2	19.2
92.164	1.06937	135	11.6	19.2
92.174	1.06928	137	11.7	19.2
92.184	1.06919	116	10.8	19.2
92.194	1.06910	127	11.3	19.2
92.204	1.06900	142	11.9	19.2
92.214	1.06891	145	12.0	19.2
92.224	1.06882	121	11.0	19.2
92.235	1.06873	125	11.2	19.2
92.245	1.06864	134	11.6	19.2
92.255	1.06855	129	11.4	19.2
92.265	1.06846	143	12.0	19.2
92.275	1.06837	124	11.1	19.2
92.285	1.06828	136	11.7	19.2
92.295	1.06819	128	11.3	19.2
92.305	1.06810	133	11.5	19.2
92.315	1.06801	119	10.9	19.2
92.325	1.06792	150	12.2	19.2
92.336	1.06783	138	11.7	19.2
92.346	1.06774	139	11.8	19.2

92.356	1.06765	126	11.2	19.2
92.366	1.06756	135	11.6	19.2
92.376	1.06747	135	11.6	19.2
92.386	1.06738	129	11.4	19.2
92.396	1.06729	141	11.9	19.2
92.406	1.06720	122	11.0	19.2
92.416	1.06711	115	10.7	19.2
92.426	1.06702	140	11.8	19.2
92.437	1.06693	129	11.4	19.2
92.447	1.06684	124	11.1	19.2
92.457	1.06675	150	12.2	19.2
92.467	1.06666	120	11.0	19.2
92.477	1.06657	124	11.1	19.2
92.487	1.06648	136	11.7	19.2
92.497	1.06639	128	11.3	19.2
92.507	1.06630	121	11.0	19.2
92.517	1.06621	137	11.7	19.2
92.527	1.06612	142	11.9	19.2
92.538	1.06603	124	11.1	19.2
92.548	1.06594	115	10.7	19.2
92.558	1.06585	138	11.7	19.2
92.568	1.06576	139	11.8	19.2
92.578	1.06567	134	11.6	19.2
92.588	1.06558	122	11.0	19.2
92.598	1.06549	123	11.1	19.2
92.608	1.06540	146	12.1	19.2
92.618	1.06531	119	10.9	19.2
92.628	1.06522	136	11.7	19.2
92.639	1.06513	134	11.6	19.2
92.649	1.06504	122	11.0	19.2
92.659	1.06495	131	11.4	19.2
92.669	1.06486	120	11.0	19.2
92.679	1.06477	124	11.1	19.2
92.689	1.06468	116	10.8	19.2
92.699	1.06459	134	11.6	19.2
92.709	1.06450	120	11.0	19.2
92.719	1.06441	118	10.9	19.2
92.729	1.06432	124	11.1	19.2
92.740	1.06423	129	11.4	19.2
92.750	1.06414	107	10.3	19.2
92.760	1.06405	123	11.1	19.2
92.770	1.06396	123	11.1	19.2
92.780	1.06388	142	11.9	19.2
92.790	1.06379	136	11.7	19.2
92.800	1.06370	128	11.3	19.2
92.810	1.06361	128	11.3	19.2
92.820	1.06352	118	10.9	19.2
92.830	1.06343	122	11.0	19.2
92.841	1.06334	125	11.2	19.2
92.851	1.06325	123	11.1	19.2

92.861	1.06316	139	11.8	19.2
92.871	1.06307	126	11.2	19.2
92.881	1.06298	122	11.0	19.2
92.891	1.06289	128	11.3	19.2
92.901	1.06281	119	10.9	19.2
92.911	1.06272	121	11.0	19.2
92.921	1.06263	114	10.7	19.2
92.931	1.06254	122	11.0	19.2
92.942	1.06245	142	11.9	19.2
92.952	1.06236	142	11.9	19.2
92.962	1.06227	125	11.2	19.2
92.972	1.06218	140	11.8	19.2
92.982	1.06209	125	11.2	19.2
92.992	1.06200	136	11.7	19.2
93.002	1.06192	126	11.2	19.2
93.012	1.06183	117	10.8	19.2
93.022	1.06174	138	11.7	19.2
93.032	1.06165	126	11.2	19.2
93.042	1.06156	145	12.0	19.2
93.053	1.06147	142	11.9	19.2
93.063	1.06138	142	11.9	19.2
93.073	1.06129	116	10.8	19.2
93.083	1.06121	122	11.0	19.2
93.093	1.06112	130	11.4	19.2
93.103	1.06103	131	11.4	19.2
93.113	1.06094	122	11.0	19.2
93.123	1.06085	143	12.0	19.2
93.133	1.06076	145	12.0	19.2
93.143	1.06067	134	11.6	19.2
93.154	1.06059	139	11.8	19.2
93.164	1.06050	154	12.4	19.2
93.174	1.06041	121	11.0	19.2
93.184	1.06032	119	10.9	19.2
93.194	1.06023	140	11.8	19.2
93.204	1.06014	124	11.1	19.2
93.214	1.06006	145	12.0	19.2
93.224	1.05997	126	11.2	19.2
93.234	1.05988	121	11.0	19.2
93.244	1.05979	127	11.3	19.2
93.255	1.05970	122	11.0	19.2
93.265	1.05961	96.0	9.80	19.2
93.275	1.05953	119	10.9	19.2
93.285	1.05944	138	11.7	19.2
93.295	1.05935	143	12.0	19.2
93.305	1.05926	114	10.7	19.2
93.315	1.05917	128	11.3	19.2
93.325	1.05909	135	11.6	19.2
93.335	1.05900	146	12.1	19.2
93.345	1.05891	124	11.1	19.2
93.356	1.05882	127	11.3	19.2

93.366	1.05873	122	11.0	19.2
93.376	1.05865	117	10.8	19.2
93.386	1.05856	124	11.1	19.2
93.396	1.05847	117	10.8	19.2
93.406	1.05838	129	11.4	19.2
93.416	1.05829	156	12.5	19.2
93.426	1.05821	131	11.4	19.2
93.436	1.05812	120	11.0	19.2
93.446	1.05803	113	10.6	19.2
93.457	1.05794	127	11.3	19.2
93.467	1.05785	131	11.4	19.2
93.477	1.05777	135	11.6	19.2
93.487	1.05768	134	11.6	19.2
93.497	1.05759	132	11.5	19.2
93.507	1.05750	133	11.5	19.2
93.517	1.05742	147	12.1	19.2
93.527	1.05733	132	11.5	19.2
93.537	1.05724	120	11.0	19.2
93.547	1.05715	139	11.8	19.2
93.558	1.05707	127	11.3	19.2
93.568	1.05698	114	10.7	19.2
93.578	1.05689	118	10.9	19.2
93.588	1.05680	133	11.5	19.2
93.598	1.05672	106	10.3	19.2
93.608	1.05663	132	11.5	19.2
93.618	1.05654	149	12.2	19.2
93.628	1.05645	123	11.1	19.2
93.638	1.05637	119	10.9	19.2
93.648	1.05628	131	11.4	19.2
93.659	1.05619	113	10.6	19.2
93.669	1.05610	127	11.3	19.2
93.679	1.05602	128	11.3	19.2
93.689	1.05593	137	11.7	19.2
93.699	1.05584	116	10.8	19.2
93.709	1.05576	136	11.7	19.2
93.719	1.05567	124	11.1	19.2
93.729	1.05558	127	11.3	19.2
93.739	1.05549	118	10.9	19.2
93.749	1.05541	122	11.0	19.2
93.760	1.05532	115	10.7	19.2
93.770	1.05523	142	11.9	19.2
93.780	1.05515	125	11.2	19.2
93.790	1.05506	117	10.8	19.2
93.800	1.05497	124	11.1	19.2
93.810	1.05488	143	12.0	19.2
93.820	1.05480	138	11.7	19.2
93.830	1.05471	122	11.0	19.2
93.840	1.05462	138	11.7	19.2
93.850	1.05454	108	10.4	19.2
93.861	1.05445	118	10.9	19.2

93.871	1.05436	125	11.2	19.2
93.881	1.05428	149	12.2	19.2
93.891	1.05419	119	10.9	19.2
93.901	1.05410	139	11.8	19.2
93.911	1.05402	134	11.6	19.2
93.921	1.05393	125	11.2	19.2
93.931	1.05384	125	11.2	19.2
93.941	1.05376	123	11.1	19.2
93.951	1.05367	130	11.4	19.2
93.962	1.05358	136	11.7	19.2
93.972	1.05350	125	11.2	19.2
93.982	1.05341	144	12.0	19.2
93.992	1.05332	124	11.1	19.2
94.002	1.05324	126	11.2	19.2
94.012	1.05315	140	11.8	19.2
94.022	1.05306	127	11.3	19.2
94.032	1.05298	111	10.5	19.2
94.042	1.05289	123	11.1	19.2
94.052	1.05280	146	12.1	19.2
94.063	1.05272	128	11.3	19.2
94.073	1.05263	133	11.5	19.2
94.083	1.05254	130	11.4	19.2
94.093	1.05246	126	11.2	19.2
94.103	1.05237	131	11.4	19.2
94.113	1.05229	111	10.5	19.2
94.123	1.05220	123	11.1	19.2
94.133	1.05211	124	11.1	19.2
94.143	1.05203	132	11.5	19.2
94.153	1.05194	113	10.6	19.2
94.164	1.05185	123	11.1	19.2
94.174	1.05177	121	11.0	19.2
94.184	1.05168	137	11.7	19.2
94.194	1.05160	128	11.3	19.2
94.204	1.05151	127	11.3	19.2
94.214	1.05142	124	11.1	19.2
94.224	1.05134	147	12.1	19.2
94.234	1.05125	132	11.5	19.2
94.244	1.05116	126	11.2	19.2
94.254	1.05108	127	11.3	19.2
94.265	1.05099	118	10.9	19.2
94.275	1.05091	109	10.4	19.2
94.285	1.05082	118	10.9	19.2
94.295	1.05074	119	10.9	19.2
94.305	1.05065	127	11.3	19.2
94.315	1.05056	117	10.8	19.2
94.325	1.05048	141	11.9	19.2
94.335	1.05039	118	10.9	19.2
94.345	1.05031	117	10.8	19.2
94.355	1.05022	132	11.5	19.2
94.365	1.05013	133	11.5	19.2

94.376	1.05005	148	12.2	19.2
94.386	1.04996	158	12.6	19.2
94.396	1.04988	131	11.4	19.2
94.406	1.04979	125	11.2	19.2
94.416	1.04971	140	11.8	19.2
94.426	1.04962	108	10.4	19.2
94.436	1.04953	125	11.2	19.2
94.446	1.04945	122	11.0	19.2
94.456	1.04936	132	11.5	19.2
94.466	1.04928	144	12.0	19.2
94.477	1.04919	130	11.4	19.2
94.487	1.04911	116	10.8	19.2
94.497	1.04902	131	11.4	19.2
94.507	1.04894	132	11.5	19.2
94.517	1.04885	124	11.1	19.2
94.527	1.04876	120	11.0	19.2
94.537	1.04868	122	11.0	19.2
94.547	1.04859	134	11.6	19.2
94.557	1.04851	108	10.4	19.2
94.567	1.04842	131	11.4	19.2
94.578	1.04834	117	10.8	19.2
94.588	1.04825	127	11.3	19.2
94.598	1.04817	119	10.9	19.2
94.608	1.04808	114	10.7	19.2
94.618	1.04800	133	11.5	19.2
94.628	1.04791	121	11.0	19.2
94.638	1.04783	98.0	9.90	19.2
94.648	1.04774	140	11.8	19.2
94.658	1.04766	130	11.4	19.2
94.668	1.04757	124	11.1	19.2
94.679	1.04749	133	11.5	19.2
94.689	1.04740	121	11.0	19.2
94.699	1.04732	129	11.4	19.2
94.709	1.04723	108	10.4	19.2
94.719	1.04715	128	11.3	19.2
94.729	1.04706	133	11.5	19.2
94.739	1.04698	138	11.7	19.2
94.749	1.04689	121	11.0	19.2
94.759	1.04681	141	11.9	19.2
94.769	1.04672	114	10.7	19.2
94.780	1.04664	114	10.7	19.2
94.790	1.04655	121	11.0	19.2
94.800	1.04647	122	11.0	19.2
94.810	1.04638	126	11.2	19.2
94.820	1.04630	105	10.2	19.2
94.830	1.04621	118	10.9	19.2
94.840	1.04613	117	10.8	19.2
94.850	1.04604	121	11.0	19.2
94.860	1.04596	125	11.2	19.2
94.870	1.04587	122	11.0	19.2

94.881	1.04579	116	10.8	19.2
94.891	1.04570	124	11.1	19.2
94.901	1.04562	130	11.4	19.2
94.911	1.04554	109	10.4	19.2
94.921	1.04545	125	11.2	19.2
94.931	1.04537	124	11.1	19.2
94.941	1.04528	110	10.5	19.2
94.951	1.04520	134	11.6	19.2
94.961	1.04511	141	11.9	19.2
94.971	1.04503	157	12.5	19.2
94.982	1.04494	123	11.1	19.2
94.992	1.04486	133	11.5	19.2
95.002	1.04478	124	11.1	19.2
95.012	1.04469	148	12.2	19.2
95.022	1.04461	133	11.5	19.2
95.032	1.04452	137	11.7	19.2
95.042	1.04444	109	10.4	19.2
95.052	1.04435	133	11.5	19.2
95.062	1.04427	129	11.4	19.2
95.072	1.04419	122	11.0	19.2
95.083	1.04410	144	12.0	19.2
95.093	1.04402	152	12.3	19.2
95.103	1.04393	123	11.1	19.2
95.113	1.04385	138	11.7	19.2
95.123	1.04376	117	10.8	19.2
95.133	1.04368	116	10.8	19.2
95.143	1.04360	116	10.8	19.2
95.153	1.04351	103	10.1	19.2
95.163	1.04343	130	11.4	19.2
95.173	1.04334	112	10.6	19.2
95.184	1.04326	122	11.0	19.2
95.194	1.04318	135	11.6	19.2
95.204	1.04309	131	11.4	19.2
95.214	1.04301	123	11.1	19.2
95.224	1.04292	157	12.5	19.2
95.234	1.04284	121	11.0	19.2
95.244	1.04276	128	11.3	19.2
95.254	1.04267	125	11.2	19.2
95.264	1.04259	136	11.7	19.2
95.274	1.04251	120	11.0	19.2
95.285	1.04242	123	11.1	19.2
95.295	1.04234	147	12.1	19.2
95.305	1.04225	112	10.6	19.2
95.315	1.04217	114	10.7	19.2
95.325	1.04209	119	10.9	19.2
95.335	1.04200	131	11.4	19.2
95.345	1.04192	125	11.2	19.2
95.355	1.04184	131	11.4	19.2
95.365	1.04175	131	11.4	19.2
95.375	1.04167	114	10.7	19.2

95.386	1.04158	132	11.5	19.2
95.396	1.04150	131	11.4	19.2
95.406	1.04142	115	10.7	19.2
95.416	1.04133	124	11.1	19.2
95.426	1.04125	134	11.6	19.2
95.436	1.04117	116	10.8	19.2
95.446	1.04108	131	11.4	19.2
95.456	1.04100	129	11.4	19.2
95.466	1.04092	125	11.2	19.2
95.476	1.04083	126	11.2	19.2
95.487	1.04075	150	12.2	19.2
95.497	1.04067	124	11.1	19.2
95.507	1.04058	142	11.9	19.2
95.517	1.04050	151	12.3	19.2
95.527	1.04042	128	11.3	19.2
95.537	1.04033	124	11.1	19.2
95.547	1.04025	117	10.8	19.2
95.557	1.04017	141	11.9	19.2
95.567	1.04008	126	11.2	19.2
95.577	1.04000	129	11.4	19.2
95.588	1.03992	137	11.7	19.2
95.598	1.03984	116	10.8	19.2
95.608	1.03975	111	10.5	19.2
95.618	1.03967	136	11.7	19.2
95.628	1.03959	123	11.1	19.2
95.638	1.03950	120	11.0	19.2
95.648	1.03942	125	11.2	19.2
95.658	1.03934	140	11.8	19.2
95.668	1.03925	122	11.0	19.2
95.678	1.03917	119	10.9	19.2
95.688	1.03909	129	11.4	19.2
95.699	1.03901	103	10.1	19.2
95.709	1.03892	128	11.3	19.2
95.719	1.03884	114	10.7	19.2
95.729	1.03876	119	10.9	19.2
95.739	1.03867	114	10.7	19.2
95.749	1.03859	127	11.3	19.2
95.759	1.03851	117	10.8	19.2
95.769	1.03843	134	11.6	19.2
95.779	1.03834	117	10.8	19.2
95.789	1.03826	142	11.9	19.2
95.800	1.03818	120	11.0	19.2
95.810	1.03809	128	11.3	19.2
95.820	1.03801	113	10.6	19.2
95.830	1.03793	124	11.1	19.2
95.840	1.03785	134	11.6	19.2
95.850	1.03776	122	11.0	19.2
95.860	1.03768	127	11.3	19.2
95.870	1.03760	139	11.8	19.2
95.880	1.03752	118	10.9	19.2

95.890	1.03743	129	11.4	19.2
95.901	1.03735	131	11.4	19.2
95.911	1.03727	125	11.2	19.2
95.921	1.03719	118	10.9	19.2
95.931	1.03710	132	11.5	19.2
95.941	1.03702	125	11.2	19.2
95.951	1.03694	120	11.0	19.2
95.961	1.03686	109	10.4	19.2
95.971	1.03677	119	10.9	19.2
95.981	1.03669	140	11.8	19.2
95.991	1.03661	118	10.9	19.2
96.002	1.03653	124	11.1	19.2
96.012	1.03645	113	10.6	19.2
96.022	1.03636	133	11.5	19.2
96.032	1.03628	124	11.1	19.2
96.042	1.03620	119	10.9	19.2
96.052	1.03612	136	11.7	19.2
96.062	1.03603	121	11.0	19.2
96.072	1.03595	109	10.4	19.2
96.082	1.03587	111	10.5	19.2
96.092	1.03579	121	11.0	19.2
96.103	1.03571	108	10.4	19.2
96.113	1.03562	122	11.0	19.2
96.123	1.03554	135	11.6	19.2
96.133	1.03546	123	11.1	19.2
96.143	1.03538	127	11.3	19.2
96.153	1.03530	108	10.4	19.2
96.163	1.03521	120	11.0	19.2
96.173	1.03513	138	11.7	19.2
96.183	1.03505	113	10.6	19.2
96.193	1.03497	122	11.0	19.2
96.204	1.03489	116	10.8	19.2
96.214	1.03481	115	10.7	19.2
96.224	1.03472	121	11.0	19.2
96.234	1.03464	132	11.5	19.2
96.244	1.03456	136	11.7	19.2
96.254	1.03448	125	11.2	19.2
96.264	1.03440	115	10.7	19.2
96.274	1.03431	126	11.2	19.2
96.284	1.03423	109	10.4	19.2
96.294	1.03415	125	11.2	19.2
96.305	1.03407	139	11.8	19.2
96.315	1.03399	112	10.6	19.2
96.325	1.03391	122	11.0	19.2
96.335	1.03383	151	12.3	19.2
96.345	1.03374	127	11.3	19.2
96.355	1.03366	134	11.6	19.2
96.365	1.03358	108	10.4	19.2
96.375	1.03350	114	10.7	19.2
96.385	1.03342	118	10.9	19.2

96.395	1.03334	139	11.8	19.2
96.406	1.03325	145	12.0	19.2
96.416	1.03317	101	10.0	19.2
96.426	1.03309	125	11.2	19.2
96.436	1.03301	111	10.5	19.2
96.446	1.03293	109	10.4	19.2
96.456	1.03285	125	11.2	19.2
96.466	1.03277	121	11.0	19.2
96.476	1.03269	126	11.2	19.2
96.486	1.03260	133	11.5	19.2
96.496	1.03252	129	11.4	19.2
96.507	1.03244	130	11.4	19.2
96.517	1.03236	143	12.0	19.2
96.527	1.03228	123	11.1	19.2
96.537	1.03220	117	10.8	19.2
96.547	1.03212	131	11.4	19.2
96.557	1.03204	116	10.8	19.2
96.567	1.03195	118	10.9	19.2
96.577	1.03187	128	11.3	19.2
96.587	1.03179	128	11.3	19.2
96.597	1.03171	114	10.7	19.2
96.608	1.03163	135	11.6	19.2
96.618	1.03155	127	11.3	19.2
96.628	1.03147	126	11.2	19.2
96.638	1.03139	134	11.6	19.2
96.648	1.03131	125	11.2	19.2
96.658	1.03123	125	11.2	19.2
96.668	1.03115	108	10.4	19.2
96.678	1.03106	121	11.0	19.2
96.688	1.03098	113	10.6	19.2
96.698	1.03090	127	11.3	19.2
96.709	1.03082	116	10.8	19.2
96.719	1.03074	118	10.9	19.2
96.729	1.03066	128	11.3	19.2
96.739	1.03058	101	10.0	19.2
96.749	1.03050	120	11.0	19.2
96.759	1.03042	135	11.6	19.2
96.769	1.03034	111	10.5	19.2
96.779	1.03026	123	11.1	19.2
96.789	1.03018	140	11.8	19.2
96.799	1.03010	147	12.1	19.2
96.810	1.03001	140	11.8	19.2
96.820	1.02993	106	10.3	19.2
96.830	1.02985	144	12.0	19.2
96.840	1.02977	105	10.2	19.2
96.850	1.02969	127	11.3	19.2
96.860	1.02961	127	11.3	19.2
96.870	1.02953	120	11.0	19.2
96.880	1.02945	121	11.0	19.2
96.890	1.02937	128	11.3	19.2

96.900	1.02929	113	10.6	19.2
96.911	1.02921	131	11.4	19.2
96.921	1.02913	113	10.6	19.2
96.931	1.02905	126	11.2	19.2
96.941	1.02897	108	10.4	19.2
96.951	1.02889	120	11.0	19.2
96.961	1.02881	114	10.7	19.2
96.971	1.02873	135	11.6	19.2
96.981	1.02865	121	11.0	19.2
96.991	1.02857	116	10.8	19.2
97.001	1.02849	127	11.3	19.2
97.011	1.02841	107	10.3	19.2
97.022	1.02833	110	10.5	19.2
97.032	1.02825	138	11.7	19.2
97.042	1.02817	115	10.7	19.2
97.052	1.02809	120	11.0	19.2
97.062	1.02801	112	10.6	19.2
97.072	1.02793	119	10.9	19.2
97.082	1.02785	133	11.5	19.2
97.092	1.02777	125	11.2	19.2
97.102	1.02769	114	10.7	19.2
97.112	1.02761	117	10.8	19.2
97.123	1.02753	134	11.6	19.2
97.133	1.02745	150	12.2	19.2
97.143	1.02737	119	10.9	19.2
97.153	1.02729	120	11.0	19.2
97.163	1.02721	131	11.4	19.2
97.173	1.02713	159	12.6	19.2
97.183	1.02705	122	11.0	19.2
97.193	1.02697	105	10.2	19.2
97.203	1.02689	135	11.6	19.2
97.213	1.02681	126	11.2	19.2
97.224	1.02673	102	10.1	19.2
97.234	1.02665	97.0	9.85	19.2
97.244	1.02657	110	10.5	19.2
97.254	1.02649	134	11.6	19.2
97.264	1.02641	132	11.5	19.2
97.274	1.02633	107	10.3	19.2
97.284	1.02625	122	11.0	19.2
97.294	1.02617	116	10.8	19.2
97.304	1.02609	118	10.9	19.2
97.314	1.02601	123	11.1	19.2
97.325	1.02593	120	11.0	19.2
97.335	1.02585	128	11.3	19.2
97.345	1.02577	125	11.2	19.2
97.355	1.02569	144	12.0	19.2
97.365	1.02561	112	10.6	19.2
97.375	1.02553	144	12.0	19.2
97.385	1.02546	115	10.7	19.2
97.395	1.02538	126	11.2	19.2

97.405	1.02530	125	11.2	19.2
97.415	1.02522	122	11.0	19.2
97.426	1.02514	124	11.1	19.2
97.436	1.02506	120	11.0	19.2
97.446	1.02498	125	11.2	19.2
97.456	1.02490	123	11.1	19.2
97.466	1.02482	124	11.1	19.2
97.476	1.02474	117	10.8	19.2
97.486	1.02466	126	11.2	19.2
97.496	1.02458	106	10.3	19.2
97.506	1.02450	132	11.5	19.2
97.516	1.02442	127	11.3	19.2
97.527	1.02435	121	11.0	19.2
97.537	1.02427	150	12.2	19.2
97.547	1.02419	123	11.1	19.2
97.557	1.02411	144	12.0	19.2
97.567	1.02403	130	11.4	19.2
97.577	1.02395	109	10.4	19.2
97.587	1.02387	114	10.7	19.2
97.597	1.02379	120	11.0	19.2
97.607	1.02371	119	10.9	19.2
97.617	1.02363	124	11.1	19.2
97.628	1.02355	108	10.4	19.2
97.638	1.02348	124	11.1	19.2
97.648	1.02340	125	11.2	19.2
97.658	1.02332	122	11.0	19.2
97.668	1.02324	134	11.6	19.2
97.678	1.02316	139	11.8	19.2
97.688	1.02308	114	10.7	19.2
97.698	1.02300	126	11.2	19.2
97.708	1.02292	128	11.3	19.2
97.718	1.02285	123	11.1	19.2
97.729	1.02277	130	11.4	19.2
97.739	1.02269	136	11.7	19.2
97.749	1.02261	132	11.5	19.2
97.759	1.02253	144	12.0	19.2
97.769	1.02245	130	11.4	19.2
97.779	1.02237	120	11.0	19.2
97.789	1.02229	130	11.4	19.2
97.799	1.02222	122	11.0	19.2
97.809	1.02214	125	11.2	19.2
97.819	1.02206	124	11.1	19.2
97.830	1.02198	119	10.9	19.2
97.840	1.02190	123	11.1	19.2
97.850	1.02182	115	10.7	19.2
97.860	1.02174	112	10.6	19.2
97.870	1.02167	130	11.4	19.2
97.880	1.02159	129	11.4	19.2
97.890	1.02151	104	10.2	19.2
97.900	1.02143	115	10.7	19.2

97.910	1.02135	110	10.5	19.2
97.920	1.02127	125	11.2	19.2
97.931	1.02120	129	11.4	19.2
97.941	1.02112	112	10.6	19.2
97.951	1.02104	115	10.7	19.2
97.961	1.02096	99.0	9.95	19.2
97.971	1.02088	129	11.4	19.2
97.981	1.02080	121	11.0	19.2
97.991	1.02073	127	11.3	19.2
98.001	1.02065	125	11.2	19.2
98.011	1.02057	127	11.3	19.2
98.021	1.02049	114	10.7	19.2
98.032	1.02041	115	10.7	19.2
98.042	1.02034	122	11.0	19.2
98.052	1.02026	115	10.7	19.2
98.062	1.02018	116	10.8	19.2
98.072	1.02010	122	11.0	19.2
98.082	1.02002	114	10.7	19.2
98.092	1.01995	99.0	9.95	19.2
98.102	1.01987	118	10.9	19.2
98.112	1.01979	108	10.4	19.2
98.122	1.01971	139	11.8	19.2
98.133	1.01963	121	11.0	19.2
98.143	1.01956	129	11.4	19.2
98.153	1.01948	109	10.4	19.2
98.163	1.01940	121	11.0	19.2
98.173	1.01932	126	11.2	19.2
98.183	1.01924	124	11.1	19.2
98.193	1.01917	118	10.9	19.2
98.203	1.01909	140	11.8	19.2
98.213	1.01901	116	10.8	19.2
98.223	1.01893	128	11.3	19.2
98.234	1.01885	116	10.8	19.2
98.244	1.01878	113	10.6	19.2
98.254	1.01870	139	11.8	19.2
98.264	1.01862	138	11.7	19.2
98.274	1.01854	128	11.3	19.2
98.284	1.01847	107	10.3	19.2
98.294	1.01839	119	10.9	19.2
98.304	1.01831	117	10.8	19.2
98.314	1.01823	124	11.1	19.2
98.324	1.01816	128	11.3	19.2
98.334	1.01808	133	11.5	19.2
98.345	1.01800	101	10.0	19.2
98.355	1.01792	100	10.0	19.2
98.365	1.01785	126	11.2	19.2
98.375	1.01777	130	11.4	19.2
98.385	1.01769	127	11.3	19.2
98.395	1.01761	131	11.4	19.2
98.405	1.01754	111	10.5	19.2

98.415	1.01746	113	10.6	19.2
98.425	1.01738	130	11.4	19.2
98.435	1.01730	119	10.9	19.2
98.446	1.01723	124	11.1	19.2
98.456	1.01715	129	11.4	19.2
98.466	1.01707	130	11.4	19.2
98.476	1.01699	119	10.9	19.2
98.486	1.01692	127	11.3	19.2
98.496	1.01684	127	11.3	19.2
98.506	1.01676	121	11.0	19.2
98.516	1.01669	116	10.8	19.2
98.526	1.01661	112	10.6	19.2
98.536	1.01653	111	10.5	19.2
98.547	1.01645	122	11.0	19.2
98.557	1.01638	131	11.4	19.2
98.567	1.01630	114	10.7	19.2
98.577	1.01622	146	12.1	19.2
98.587	1.01615	127	11.3	19.2
98.597	1.01607	125	11.2	19.2
98.607	1.01599	125	11.2	19.2
98.617	1.01592	107	10.3	19.2
98.627	1.01584	117	10.8	19.2
98.637	1.01576	126	11.2	19.2
98.648	1.01568	109	10.4	19.2
98.658	1.01561	149	12.2	19.2
98.668	1.01553	132	11.5	19.2
98.678	1.01545	129	11.4	19.2
98.688	1.01538	114	10.7	19.2
98.698	1.01530	102	10.1	19.2
98.708	1.01522	114	10.7	19.2
98.718	1.01515	118	10.9	19.2
98.728	1.01507	124	11.1	19.2
98.738	1.01499	121	11.0	19.2
98.749	1.01492	121	11.0	19.2
98.759	1.01484	114	10.7	19.2
98.769	1.01476	101	10.0	19.2
98.779	1.01469	130	11.4	19.2
98.789	1.01461	117	10.8	19.2
98.799	1.01453	119	10.9	19.2
98.809	1.01446	119	10.9	19.2
98.819	1.01438	125	11.2	19.2
98.829	1.01430	118	10.9	19.2
98.839	1.01423	111	10.5	19.2
98.850	1.01415	115	10.7	19.2
98.860	1.01407	109	10.4	19.2
98.870	1.01400	131	11.4	19.2
98.880	1.01392	121	11.0	19.2
98.890	1.01384	130	11.4	19.2
98.900	1.01377	125	11.2	19.2
98.910	1.01369	129	11.4	19.2

98.920	1.01361	116	10.8	19.2
98.930	1.01354	109	10.4	19.2
98.940	1.01346	118	10.9	19.2
98.951	1.01339	128	11.3	19.2
98.961	1.01331	122	11.0	19.2
98.971	1.01323	137	11.7	19.2
98.981	1.01316	102	10.1	19.2
98.991	1.01308	123	11.1	19.2
99.001	1.01300	129	11.4	19.2
99.011	1.01293	128	11.3	19.2
99.021	1.01285	118	10.9	19.2
99.031	1.01277	111	10.5	19.2
99.041	1.01270	128	11.3	19.2
99.052	1.01262	123	11.1	19.2
99.062	1.01255	116	10.8	19.2
99.072	1.01247	131	11.4	19.2
99.082	1.01239	117	10.8	19.2
99.092	1.01232	124	11.1	19.2
99.102	1.01224	121	11.0	19.2
99.112	1.01217	125	11.2	19.2
99.122	1.01209	112	10.6	19.2
99.132	1.01201	121	11.0	19.2
99.142	1.01194	100	10.0	19.2
99.153	1.01186	125	11.2	19.2
99.163	1.01179	121	11.0	19.2
99.173	1.01171	121	11.0	19.2
99.183	1.01163	124	11.1	19.2
99.193	1.01156	124	11.1	19.2
99.203	1.01148	148	12.2	19.2
99.213	1.01141	123	11.1	19.2
99.223	1.01133	123	11.1	19.2
99.233	1.01125	127	11.3	19.2
99.243	1.01118	104	10.2	19.2
99.254	1.01110	122	11.0	19.2
99.264	1.01103	131	11.4	19.2
99.274	1.01095	108	10.4	19.2
99.284	1.01088	116	10.8	19.2
99.294	1.01080	134	11.6	19.2
99.304	1.01072	110	10.5	19.2
99.314	1.01065	127	11.3	19.2
99.324	1.01057	117	10.8	19.2
99.334	1.01050	144	12.0	19.2
99.344	1.01042	116	10.8	19.2
99.355	1.01035	118	10.9	19.2
99.365	1.01027	121	11.0	19.2
99.375	1.01020	115	10.7	19.2
99.385	1.01012	111	10.5	19.2
99.395	1.01004	137	11.7	19.2
99.405	1.00997	123	11.1	19.2
99.415	1.00989	136	11.7	19.2

99.425	1.00982	105	10.2	19.2
99.435	1.00974	134	11.6	19.2
99.445	1.00967	132	11.5	19.2
99.456	1.00959	108	10.4	19.2
99.466	1.00952	137	11.7	19.2
99.476	1.00944	131	11.4	19.2
99.486	1.00937	107	10.3	19.2
99.496	1.00929	118	10.9	19.2
99.506	1.00922	124	11.1	19.2
99.516	1.00914	117	10.8	19.2
99.526	1.00906	103	10.1	19.2
99.536	1.00899	120	11.0	19.2
99.546	1.00891	114	10.7	19.2
99.557	1.00884	127	11.3	19.2
99.567	1.00876	132	11.5	19.2
99.577	1.00869	124	11.1	19.2
99.587	1.00861	135	11.6	19.2
99.597	1.00854	118	10.9	19.2
99.607	1.00846	118	10.9	19.2
99.617	1.00839	130	11.4	19.2
99.627	1.00831	119	10.9	19.2
99.637	1.00824	128	11.3	19.2
99.647	1.00816	137	11.7	19.2
99.657	1.00809	109	10.4	19.2
99.668	1.00801	127	11.3	19.2
99.678	1.00794	132	11.5	19.2
99.688	1.00786	128	11.3	19.2
99.698	1.00779	124	11.1	19.2
99.708	1.00771	119	10.9	19.2
99.718	1.00764	111	10.5	19.2
99.728	1.00756	111	10.5	19.2
99.738	1.00749	111	10.5	19.2
99.748	1.00741	115	10.7	19.2
99.758	1.00734	108	10.4	19.2
99.769	1.00726	112	10.6	19.2
99.779	1.00719	128	11.3	19.2
99.789	1.00711	128	11.3	19.2
99.799	1.00704	112	10.6	19.2
99.809	1.00697	109	10.4	19.2
99.819	1.00689	137	11.7	19.2
99.829	1.00682	101	10.0	19.2
99.839	1.00674	112	10.6	19.2
99.849	1.00667	131	11.4	19.2
99.859	1.00659	105	10.2	19.2
99.870	1.00652	119	10.9	19.2
99.880	1.00644	117	10.8	19.2
99.890	1.00637	120	11.0	19.2
99.900	1.00629	101	10.0	19.2
99.910	1.00622	134	11.6	19.2
99.920	1.00614	136	11.7	19.2

99.930	1.00607	131	11.4	19.2
99.940	1.00600	115	10.7	19.2
99.950	1.00592	96.0	9.80	19.2
99.960	1.00585	115	10.7	19.2
99.971	1.00577	133	11.5	19.2
99.981	1.00570	124	11.1	19.2
99.991	1.00562	112	10.6	19.2
100.001	1.00555	116	10.8	19.2

2Theta

Show	Icon	Color	Index	Name	Parent	Scan	Pattern #
Yes			0	PDF 53-0447 (Tune Cell)	Pattern List #3	1.raw #1	PDF 53-0447 (Tune Cell)
Yes			1	PDF 51-0768 (Tune Cell)	Pattern List #3	1.raw #1	PDF 51-0768 (Tune Cell)
Yes			2	PDF 52-0233 (Tune Cell)	Pattern List #3	1.raw #1	PDF 52-0233 (Tune Cell)
Yes			3	PDF 88-2402 (Tune Cell)	Pattern List #3	1.raw #1	PDF 88-2402 (Tune Cell)
Yes			4	PDF 88-2401 (Tune Cell)	Pattern List #3	1.raw #1	PDF 88-2401 (Tune Cell)

Compound Name	Formula	Quality	Y-Scale	I/Ic DB	I/Ic User	S-Q
Boron Carbon Nitride	B0.47 C0.23 N0.30	Low precision	34.2899 %	(1)		40.94 %
Boron Carbon Nitride	B2 C5 N2	Blank	11.1898 %	(1)		13.36 %
Boron Nitride Carbide	(B N)0.33 C0.67	Blank	18.7502 %	(1)		22.39 %
Carbon Boron Nitride	(C2).19 (B N).81	Calculated	11.2968 %	1.190		11.34 %
Carbon Boron Nitride	(C2).429 (B N).571	Calculated	11.5292 %	1.150		11.97 %

Added Reference	d x by	Scan WL	Wavelength	System	Space Group	a	b	c
	1.0000	Yes	1.54060	Orthorhombic	P (0)	4.77600	4.58500	3.62900
	1.0000	Yes	1.54060	Cubic	Fd-3m (227)	3.60500		
	1.0000	Yes	1.54060	Hexagonal		2.47500		6.76000
	1.0000	Yes	1.54060	Cubic	F-43m (216)	3.61300		
	1.0000	Yes	1.54060	Cubic	F-43m (216)	3.59600		

alpha	beta	gamma	Z	Volume	Density	Cell Tuned	F (N)
			14	79.47		Automatic	F27= 1.4(0.2280, 86)
			1	46.85	3.888	Automatic	F5= 5.1(0.1950, 5)
				35.86		Automatic	F7= 2.2(0.1010, 31)
			4	47.16	3.473	Automatic	F3= 999.9(0.0000, 3)
			4	46.50	3.496	Automatic	F3= 999.9(0.0000, 3)

Pattern: PDF 53-0447 (Tune Cell)   Radiation: 1.54060   Quality: User modified

**Formula**	B0.47 C0.23 N0.30
**Name**	Boron Carbon Nitride
**Name (mineral)**	
**Name (common)**	

**Lattice:**	Orthorhombic	**Mol. weight =**	12.05
**S.G.:**	P (0)	**Volume [CD] =**	79.47
		**Dx =**	
		**Dm =**	
		**I/Icor =**	-1.000

**a =**	4.77600	**alpha =**	
**b =**	4.58500	**beta =**	
**c =**	3.62900	**gamma =**	
**a/b =**	1.04166	**Z =**	14
**c/b =**	0.79149		

Sample Preparation: A -- precursor was transformed at 1600 C under for 15 minutes. A 3 B2 N4atalyst was added and the mixture was converted at 5.5 GPa and 1600 C
General Comments: Poor fit to proposed cell
General Comments: Unknown impurity phase present

Primary Reference
Publication: Private Communication
Authors: Tian, Y., College of Materials Science and Engineering, Yanshan Univ., Hebei, P.R.China.

**Radiation:**	CuKa1	**Filter:**	Not specified
**Wavelength:**	1.54060	**d-spacing:**	
**SS/FOM:**	1.4 (0.228,86)		

d	2θ	I	h	k	l
4.77600	18.563	3	1	0	0
3.30755	26.935	75	1	1	0
2.88949	30.922	1	1	0	1
2.84554	31.412	1	0	1	1
2.44455	36.735	1	1	1	1
2.38800	37.637	1	2	0	0
2.11795	42.655	1	2	1	0
2.06674	43.766	1	1	2	0
1.99485	45.430	1	2	0	1
1.81450	50.241	1	0	0	2
1.68718	54.331	1	0	1	2
1.65377	55.522	8	2	2	0
1.59200	57.875	1	3	0	0
1.50392	61.620	1	3	1	0
1.44475	64.440	1	2	0	2
1.40852	66.307	1	0	3	1
1.35099	69.525	1	1	3	1
1.28727	73.511	1	2	3	0
1.22227	78.132	1	2	2	2
1.20967	79.105	1	0	0	3
1.18630	80.982	1			
1.17264	82.127	1	1	0	3
1.14625	84.446	1	0	4	0
1.14250	84.788	1			
1.11460	87.435	1	1	4	0
1.11040	87.850	1			
1.08500	90.462	1			
1.06085	93.123	1	3	2	2
1.04399	95.096	1	1	2	3
1.03337	96.391	1	2	4	0
0.90554	116.565	1	5	1	1

Pattern: PDF 51-0768 (Tune Cell)   Radiation: 1.54060   Quality: User modified

**Formula**	B2 C5 N2
**Name**	Boron Carbon Nitride
**Name (mineral)**	
**Name (common)**	

d	2θ	I	h	k	l
2.08135	43.443	24	1	1	1
1.27456	74.366	5	2	2	0
1.08695	90.255	4	3	1	1
0.90125	117.454	1	4	0	0
0.82704	137.305	2	3	3	1

**Lattice:**	Cubic
**S.G.:**	Fd-3m (227)

**Mol. weight =**	109.69
**Volume [CD] =**	46.85
**Dx =**	
**Dm =**	
**I/Icor =**	-1.000

**a =**	3.60500	**alpha =**	
**b =**		**beta =**	
**c =**		**gamma =**	
**a/b =**	1.00000	**Z =**	1
**c/b =**	1.00000		

Sample Preparation: The chemical vapor deposit of H3 C Nnd Cl3as heated, then shock-compressed
General Comments: Intensities recorded with an image plate
Additional Pattern: See 35-1293 for Debye-Scherrer data

Primary Reference
Publication: J. Chem. Soc., Faraday Trans.
Detail: volume 92, page 5067 (1996)
Authors: Komatsu, T., Kakudate, Y., Fujiwara, S.

**Radiation:**	CuKa	**Filter:**	F
**Wavelength:**	1.54060	**d-spacing:**	
**SS/FOM:**	5.1 (0.195,5)		

Pattern: PDF 52-0233 (Tune Cell) Radiation: 1.54060 Quality: User modified

**Formula**	(B N)0.33 C0.67
**Name**	Boron Nitride Carbide
**Name (mineral)**	
**Name (common)**	

d	2θ	I	h	k	l
3.38000	26.347	41	0	0	2
2.14341	42.124	33	1	0	0
2.04317	44.298	20	1	0	1
1.69000	54.233	4	0	0	4
1.23750	76.993	20	1	1	0
1.16900	82.438	20			
1.07171	91.904	4	2	0	0
0.99050	102.099	2			
0.80438	146.525	2	2	1	1

Lattice: Hexagonal
S.G.:

Mol. weight = 16.24
Volume [CD] = 35.86
Dx =
Dm =
I/Icor = -1.000

**a =**	2.47500	**alpha =**	
**b =**		**beta =**	
**c =**	6.76000	**gamma =**	
**a/b =**	1.00000	**Z =**	
**c/b =**	2.73131		

Sample Preparation: Prepared by vaporization and condensation of a 1:1 molar mixture of amorphous Nnd carbon black by shock compression

Primary Reference
Publication: J. Am. Ceram. Soc.
Detail: volume 81, page 1941 (1998)
Authors: Yamada, K.

Radiation: CuKa1
Wavelength: 1.54060
SS/FOM: 2.2 (0.101,31)

Filter: Not specified
d-spacing:

Pattern: PDF 88-2402 (Tune Cell) Radiation: 1.54060 Quality: User modified

**Formula**	(C2).19 (B N).81
**Name**	Carbon Boron Nitride
**Name (mineral)**	
**Name (common)**	

**Lattice:**	Cubic	**Mol. weight =**	24.67
**S.G.:**	F-43m (216)	**Volume [CD] =**	47.16
		**Dx =**	
		**Dm =**	
		**I/Icor =**	1.190

**a =**	3.61300	**alpha =**	
**b =**		**beta =**	
**c =**		**gamma =**	
**a/b =**	1.00000	**Z =**	4
**c/b =**	1.00000		

ICSD Collection Code: 041584
Remark From ICSD/CSD: REM XDP
Remark From ICSD/CSD: REM M Synthesized at at 30 and 50 GPa, cell of BN: 3.617(1)
Remark From ICSD/CSD: REM M PDF 35-1293
Test From ICSD: No R value given
Test From ICSD: At least one TF missing
Remark From ICSD/CSD: REM PRE Mentioned
Article Title: High-pressure synthesis, characterization, and equation of state of cubic C-BN solid solutions

Structure
Publication: Phys. Rev. B: Condens. Matter
Detail: volume 51, page 12149 (1995)
Authors: Knittle, E., Kaner, R.B., Jeanloz, R., Cohen, M.L.
Primary Reference
Publication: Calculated from ICSD using POWD-12++

**Radiation:**	CuKa1	**Filter:**	Not specified
**Wavelength:**	1.54060	**d-spacing:**	
**SS/FOM:**	999.9 (0,3)		

d	2θ	I	h	k	l
2.08597	43.342	25	1	1	1
1.80650	50.479	1	2	0	0
1.27739	74.174	6	2	2	0

Pattern: PDF 88-2401 (Tune Cell) Radiation: 1.54060 Quality: User modified

Formula (C2).429 (B N).571
Name Carbon Boron Nitride
Name (mineral)
Name (common)

Lattice: Cubic
S.G.: F-43m (216)

Mol. weight = 24.48
Volume [CD] = 46.5
Dx =
Dm =
I/Icor = 1.150

a = 3.59600
b =
c =
a/b = 1.00000
c/b = 1.00000

alpha =
beta =
gamma =
Z = 4

ICSD Collection Code: 041583
Remark From ICSD/CSD: REM XDP
Remark From ICSD/CSD: REM M Synthesized at at 30 and 40 GPa, cell of C.5 (BN)
5: 3.602(
Remark From ICSD/CSD: REM M PDF 35-1293
Test From ICSD: No R value given
Test From ICSD: At least one TF missing
Remark From ICSD/CSD: REM PRE Mentioned
Article Title: High-pressure synthesis, characterization, and equation of state of cubic C-BN solid solutions

Structure
Publication: Phys. Rev. B: Condens. Matter
Detail: volume 51, page 12149 (1995)
Authors: Knittle, E., Kaner, R.B., Jeanloz, R., Cohen, M.L.
Primary Reference
Publication: Calculated from ICSD using POWD-12++

Radiation: CuKa1
Wavelength: 1.54060
SS/FOM: 999.9 (0,3)

Filter: Not specified
d-spacing:

d	2θ	I	h	k	l
2.07615	43.557	25	1	1	1
1.79800	50.735	0	2	0	0
1.27138	74.584	7	2	2	0
